# The Bacterial Intimins and Invasins: A Large and Novel Family of Secreted Proteins

**DOI:** 10.1371/journal.pone.0014403

**Published:** 2010-12-22

**Authors:** Jennifer C. Tsai, Ming-Ren Yen, Rostislav Castillo, Denisse L. Leyton, Ian R. Henderson, Milton H. Saier

**Affiliations:** 1 Department of Molecular Biology, University of California at San Diego, La Jolla, California, United States of America; 2 The School of Immunity and Infection, University of Birmingham, Edgbaston, Birmingham, United Kingdom; University of Cambridge, United Kingdom

## Abstract

**Background:**

Gram-negative bacteria have developed a limited repertoire of solutions for secreting proteins from the cytoplasmic compartment to the exterior of the cell. Amongst the spectrum of secreted proteins are the intimins and invasins (the Int/Inv family; TC# 1.B.54) which are characterized by an N-terminal β-barrel domain and a C-terminal surface localized passenger domain. Despite the important role played by members of this family in diseases mediated by several species of the *Enterobacteriaceae*, there has been little appreciation for the distribution and diversity of these proteins amongst Gram-negative bacteria. Furthermore, there is little understanding of the molecular events governing secretion of these proteins to the extracellular milieu.

**Principal Findings:**

*In silico* approaches were used to analyze the domain organization and diversity of members of this secretion family. Proteins belonging to this family are predominantly associated with organisms from the γ-proteobacteria. Whilst proteins from the Chlamydia, γ-, β- and ε-proteobacteria possess β-barrel domains and passenger domains of various sizes, Int/Inv proteins from the α-proteobacteria, cyanobacteria and chlorobi possess only the predicted β-barrel domains. Phylogenetic analyses revealed that with few exceptions these proteins cluster according to organismal type, indicating that divergence occurred contemporaneously with speciation, and that horizontal transfer was limited. Clustering patterns of the β-barrel domains correlate well with those of the full-length proteins although the passenger domains do so with much less consistency. The modular subdomain design of the passenger domains suggests that subdomain duplication and deletion have occurred with high frequency over evolutionary time. However, all repeated subdomains are found in tandem, suggesting that subdomain shuffling occurred rarely if at all. Topological predictions for the β-barrel domains are presented.

**Conclusion:**

Based on our *in silico* analyses we present a model for the biogenesis of these proteins. This study is the first of its kind to describe this unusual family of bacterial adhesins.

## Introduction

Pathogenic Gram-negative bacteria have developed many distinct secretion mechanisms for the efficient surface display of binding domains that specifically interact with their complementary receptors on host cell surfaces [1], [2]. The Intimin/Invasin (Int/Inv) family of adhesins (TC# 1.B.54) consists of outer membrane (OM) proteins that mediate bacterial attachment to and/or invasion of their host cells [3], [4], . The archetypal members of the Int/Inv family are from strains of pathogenic *Escherichia coli* (Int) and *Yersinia* spp. (Inv). Intimins, first described by Jerse et al in enteropathogenic and *E. coli* strains, promote intimate bacterial attachment associated with attaching-effacing lesion formation in the gut mucosa [8], [9]. This intimate adherence to host cells is mediated by interaction of Intimin with Tir, a protein secreted directly from the bacterial cytoplasm into the host cell membrane via a type III protein secretion system, and which results in host cell actin reorganization. [6], [10], [11], [12], [13], [14], [15], [16], [17], [18]. In contrast, Invasin, which was first described by Isberg and coworkers, enhances the ability of *Yersinia* spp. to enter target cells, not by binding to a Tir-like protein, but by binding with high-affinity to multiple members of the β_1_-chain integrin family of mammalian cell receptors. The function of Invasin has been reiewed elsewhere.

The Intimin and Invasin systems have been studied primarily with respect to their contribution to the virulence of Gram-negative pathogens. In contradistinction, little is known of the secretory mechanism of either Intimin or Invasin. These homologous proteins are related to each other both in terms of sequence and predicted structure, possessing a conserved modular organization [2], [3], [19], [20] consisting of (i) an N-terminal signal sequence, (ii) a highly conserved N-terminal β-domain and (iii) a C-terminal surface localized “passenger” domain. The signal peptides which are predicted to mediate translocation from the cytoplasm across the inner membrane (IM) via the General Secretory (Sec)-Translocase (TC# 3.A.5). The proximal β-domains arebelieved to form porin-like β-barrel anchors in the outer membrane [4], [6], [21], [22], which are believed to form pores that are used to export the C-terminal passenger domains across the outer membrane, although this is a contentious issue [3], [19], [23], [24]. The C-terminal passenger domains are composed of repeated **b**acterial **i**mmuno**g**lobulin-like (Big) domains decorated with a C-type lectin-like subdomain (CTLD).

The existence of a domain predicted to form a β-barrel pore has led to the suggestion that passenger domains may be secreted across the outer membrane in a manner similar to Autotransporter mechanisms. Like the Int/Inv family Autotransporters have a modular structure broadly consisting of an N-terminal signal peptide, a passenger domain and a b-barrel pore-forming domain. However, in contrast to the Int/Inv family, the signal peptide is juxtaposed to the passenger domain and the b-barrel is located at the extreme C-terminus. In the case of Autotransporters the b-barrel is proposed to mediate translocation of the passenger domain to the cell surface through the pore. Nevertheless, the evidence for an autotransporter like mechanism for the Int/Inv family of proteins is still equivocal being founded on analogy rather than empiric data [3], [19], [20], [24]. Based on this information, the Int/Inv Family (TC #1.B.54) has been designated, possibly prematurely, the Autotransporter-3 (AT-3) family [25] as it may exhibit functional characteristics of two families of autotransporters, AT-1 (TC #1.B.12) and AT-2 (TC #1.B.40) which also have terminal β-barrel domains linked to multi-sub-domain-containing passenger domains [26], [27].

Here we present evidence that the Int/Inv family is larger and more widely distributed among Gram-negative bacteria than previously appreciated. We demonstrate through phylogenetics that these systems have in general evolved in parallel with the organisms that utilize the systems and that there is only limited evidence for horizontal transfer. Finally, we probe the structures of the systems and propose a model for the biogenesis of Int/Inv secreted proteins.

## Methods

### Computational Methods

**Figure 1:**
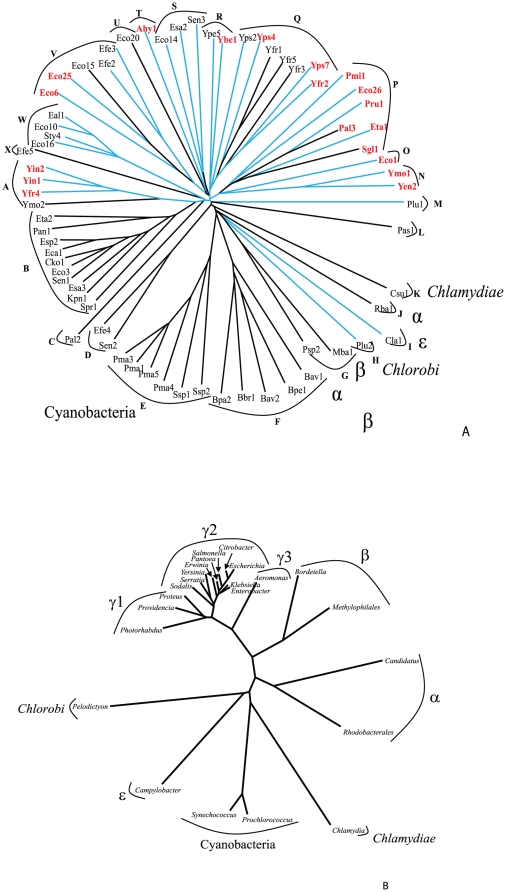
Phylogenetic tree of the full-length Int/Inv proteins. LysM containing proteins are colored in red, and proteins possessing paired cysteines, with the capacity to form disulphide bonds, are indicated with blue branches. Clusters A to X were analyzed for sequence conservation (see text), as indicated in the figures. This tree, and those presented in figures 2 and 3 are based on CLUSTAL-X-derived multiple alignments shown in Figures S1, S2 and S3, respectively. The trees were drawn with the TreeView program [34]. The organismal origins of the proteins are indicated adjacent to the branch/cluster number except for the large majority of proteins from the γ-proteobacteria which are unlabeled. This convention is also used in figures 2 and 3. Using the same program, the tree for the ribosomal RNAs, corresponding to the represented genera, was derived for the second part of this figure.

In this study, intimin-γ of *E. coli* O157:H7 (P43261; TC#1.B.54.1.1, belonging to cluster O in Fig. 1) and the invasin of *Yersinia pseudotuberculosis* (P11922; TC#1.B.54.1.2; in cluster Q in Fig. 1) were used as query sequences in PSI-BLAST searches [25], [28], [29], [30]. All homologues were retrieved from the NCBI database. PSI-BLAST searches with a cut off value of e^−4^ for the initial search and e^−5^ for the second iteration were used to identify distant homologues. Conserved domains within amino acid sequences were identified using the conserved domain database (CDD) and the MakeTable5 program [31], the latter of which incorporates a modified version of the CD-Hit program [32]. The MakeTable5 program also eliminates redundancies, closely similar sequences and fragmentary sequences. The ClustalX program [33] and the TreeView program [34] were used respectively, for multiple alignment of homologous sequences and for construction of the phylogenetic trees. After establishing homology, using BLAST with an e^−5^ cutoff and the GAP program with a 10 standard deviation (S.D.) cutoff [25], [31], [35], the homologues were analyzed topologically and phylogenetically as well as for conserved residues and motifs. For compositional analyses of the C-terminal passenger subdomains, a script was prepared based on BLAST [28], [29] using an e^−5^ cut off to find homologous subdomains.

### Structural Predictions

Topological predictions for individual proteins were made with the WHAT [36] and TMHMM [37] programs. Topological analyses of the N-terminal β-barrel regions were performed using (i) the AveHAS program with an angle of 180° and a window size of 9 residues, as is appropriate for β-structured proteins [38], and (ii) the PRED-TMBB program with default settings [39]. The PsiPred program [40] was used to predict secondary structure elements. Assignment of potential transmembrane segments (TMSs) was based on hydropathy, amphipathicity and similarity analyses as well as transmembrane β-strand predictions. Tertiary structural modeling was performed using Phyre [41] and CPHmodels [42] using default settings.

## Results and Discussion

### Identification of members of the Intimin/Invasin family of secreted proteins

The amino acid sequences of Intimin and Invasin were used to identify members of the Int/Inv family as described in the Methods section. After the initial searches, PSI-BLAST searches were conducted using homologues from distantly related bacterial kingdoms: cyanobacteria, chlorobi, chlamydia, and proteobacteria (α, β and ε). These query sequences have the following gi numbers: 1) α, 71062608; 2) β, 69204798; 3) ε, 222539800; 4) *Chlamydiae*, 69204798; 5) *Chlorobi*, 78186442; and 6) cyanobacteria, 78779562, 148241686 and 148243547. Redundant sequences and closely related sequences (of greater than 90% amino acid identity with a retained sequence) were eliminated, yielding 157 sequence-divergent proteins of sizes ranging from 237 amino acids (the β-barrel domain alone) to 8620 amino acids. These 157 sequences were examined for the presence of the β-barrel domain. As the β-barrel is deemed essential for secretion and therefore a requisite feature of Int/Inv family members, all proteins lacking this domain were eliminated from further analyses. Sixty-nine sequence-divergent proteins resulted, all of which proved to have N-terminal (never internal or C-terminal) β-domains (see Table S1 and Figure S1).

This series of investigations revealed several novel details including: (i) the Int/Inv family is larger than previously appreciated and is not limited to a few strains of *Escherichia* spp., *Yersinia* spp. and *Salmonella* spp.; (ii) only the Int/Inv proteins from γ-, β- and ε-proteobacteria as well as *Chlamydia* possess characteristic passenger domains with Big motifs, albeit these represent the majority of the proteins identified in this study; (iii) the β-barrel domains are conserved in size ranging from about 300–400 amino acids; (iv) in proteins lacking passenger domains, the functions of the β-barrel domains are unknown, but they may play roles in transporting other proteins such as non-covalent passenger domains (see below) across the outer membrane and/or anchoring them to the external surface of the envelope [5], [19] and (v) non Int/Inv proteins from various organisms, including proteins from Gram-positive bacteria (firmicutes) and planctomycetes, contain Big motifs homologous to the passenger domains of Intimins and Invasins. It should be noted that Int/Inv passenger domains show regions of homology with the passenger domains of the AT-1 and AT-2 family members.

### Phylogenetic Analyses of Intimin/Invasin Homologues

**Figure 2:**
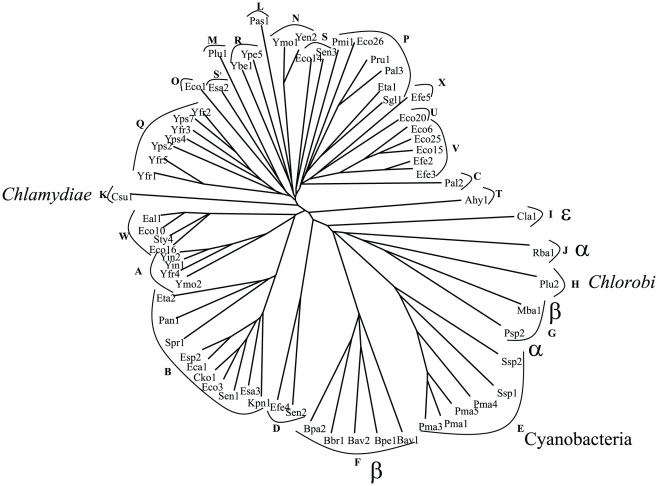
Phylogenetic tree of the N-terminal β-barrel domains. Details are as per Figure 1.

**Figure 3:**
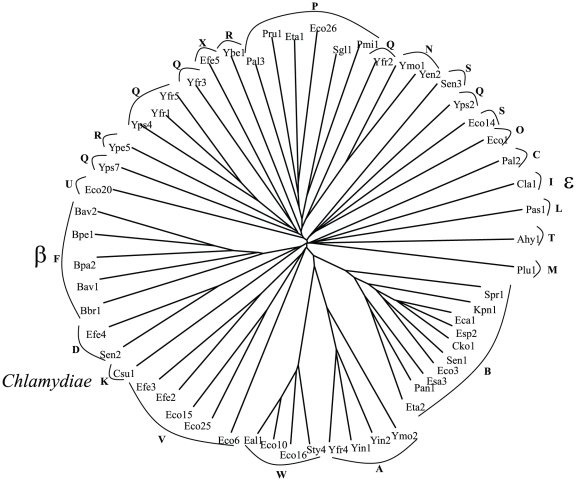
Phylogenetic tree of the C-terminal passenger domains. Details are as per Figure 1. Those proteins lacking a passenger domain were not included.

Phylogenetic analyses of the 69 Int/Inv family members were used to determine the evolutionary history of this putative secretion family. Multiple alignments (see Supplementary Materials Figs. S1, S2A, S2B and S3 at http://www.biology.ucsd.edu/~msaier/supmat/IntInv) and phylogenetic trees (Figs. 1A, 2 and 3) were generated for a) the full-length proteins, b) the β-barrel domains, and c) the passenger domains. To establish the organismal phylogeny, a 16S ribosomal RNA (rRNA) tree was constructed for all species possessing at least one member of the Int/Inv family (Fig. 1B).

The phylogenetic tree for the 69 full-length proteins (Fig. 1) shows 24 clusters, A through X. Cluster E proteins are all derived from cyanobacteria; cluster F proteins derive from β-proteobacteria, branch H is a chlorobi sequence; branch I is an ε-proteobacterial homologue; branch J is an α-proteobacterial protein, and branch K includes the single chlamydial protein. The two cluster G proteins are from one α-proteobacterium and one β-proteobacterium. Thus, only cluster G includes proteins from two phylogenetically distinct groups of organisms. All remaining sequences are from γ-proteobacteria, which form several distinct clusters (Fig. 1).

More detailed analyses of the organismal phylogenies of the larger group of γ-proteobacterial proteins (clusters A and B) revealed that the phylogenetic relationships of the proteins within a cluster in general reflect the organismal phylogenies demonstrated by the 16S rRNA tree (Fig. 1B). The only possible exceptions are Eta2 and Pan1, which cluster loosely together, and the *Yersinia* homologues in cluster A, as they do not branch together with the *Serratia* protein, Spr1. Nevertheless, in both cases, these proteins branch more deeply from the center of the tree than any other member of these clusters.

Having established the phylogenies of the full-length proteins, we next examined the phylogenies of the β-barrel and passenger domains. We observed that the clustering patterns of the β-barrel domains are very similar to those of the full-length proteins. The only significant exception is cluster S, the members of which are found on two branches in the tree representing the β-barrel domain (Fig. 2). This is not entirely surprising since the members of cluster S branch from points near the centers of both trees. The passenger domains follow this pattern to a lesser degree (Fig. 3). The passenger domain tree contains fewer proteins than the other two trees because several of the homologues possess only the β-barrel domain, as noted above.

These data clearly suggest that between bacterial phyla and orders, there has been little or no lateral transfer of genetic material encoding members of the Int/Inv family of secreted proteins, at least over recent evolutionary time (*e.g.*, within the last 1–2 billion years). The only possible exception is cluster G with one α-proteobacterial homologue and one β-proteobacterial homologue. These two proteins do not fall into either the α- or the β-proteobacterial cluster (cluster J or F, respectively) and therefore may have been derived either by early gene duplication events or by lateral transfer from a dissimilar source.

### Domain organization of the Int/Inv family

To probe the structures of members of the Int/Inv family and establish domains that might be relevant to the biogenesis of function, the 69 proteins described above were analyzed for conserved domains and structural motifs.

#### Signal sequences

All proteins were screened using SignalP for the presence of signal peptides. The length of signal peptides ranged from 18 to 65 amino acids, with a mean value of 35 amino acids. This is larger than the mean value (22.5 amino acids) for Sec-secreted proteins from Gram-negative bacteria. Some members of the autotransporter-1 (AT-1; TC# 1.B.12), autotransporter-2 (AT-2; TC# 1.B.40) and the Two Partner Secretion (TPS; TC# 1.B.20) families possess extended signal peptides which adopt an unusual organization consisting of two charged domains, two hydrophobic domains and a signal peptide recognition site [43], [44], [45]. Scrutiny of Int/Inv family members revealed that they all possess signal sequences that adopt the characteristic structure for signal peptides mediating secretion via the post-translational Sec pathway, a tripartite organization consisting of a charged N-domain, a hydrophobic membrane spanning H-domain and a signal peptidase recognition site, the C-domain. The Int/Inv signal peptides do not bear resemblance to the extended signal peptides associated with the autotransporter (AT-1) proteins. For Int/Inv family proteins with extended signal peptides, the additional amino acids could be explained by larger than normal charged N-domains. Furthermore, little or no significant sequence conservation in the Int/Inv family of signal peptides could be discerned.

#### Hydrophilic α-domains

To probe the structural organization of the Int/Inv family of proteins, secondary structure predictions were made using the PsiPred program [40]. These predictions revealed a previously unrecognized hydrophilic subdomain immediately adjacent to the signal peptide cleavage site in all proteins except Plu2 from *Pelodictyon luteolum*. This hydrophilic domain (herein designated the α-domain) consists of one or two α-helical stretches predicted to extend from the outer membrane embedded α-domain into the periplasm.

In the case of 19 proteins (colored red in Fig. 1), including Intimin, but excluding Invasin, there is another conserved structural element harboured between the signal peptide and the α-domain. Pfam analyses [46] revealed that these regions form a LysM domain. This domain, a peptidoglycan-binding domain, is about 45 residues long and is prevalent among, but not restricted to, enzymes implicated in the degradation of peptidoglycan [47], [48], [49]. The LysM domain is present only in Int/Inv family members from γ-proteobacteria.

### Topological Predictions for the β-Barrel domains

Immediately adjacent to the α-domain is a hydrophobic region predicted to reside within the outer membrane. Previous reports have suggested that this portion of the Int/Inv family proteins forms a pore within the outer membrane by adopting a β-barrel conformation, a structure common to most integral outer membrane proteins [19], [24], [50], [51]. Further evidence for a β-barrel conformation was derived from the recent demonstration that members of the Int/Inv family require components of the β-barrel assembly pathway for correct biogenesis [52]. The putative β-barrel domains in the full-length protein alignments begin at alignment position 333 and end with position 979 (Fig. S1).

**Figure 4:**
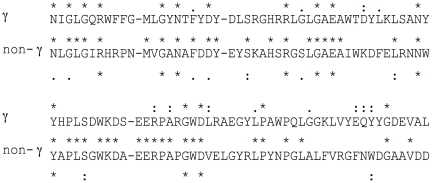
Alignment of the consensus sequences of the γ-proteobacterial (γ) and non-γ-proteobacterial (non-γ) β-barrel domains. The amino acids depicted correspond to amino acids predicted to occur within or immediately adjacent to TMSs 4–8. The positions of the fully conserved residues within each of the two respective consensus sequences are indicated with asterisks (*) above and below the two sequences. Colons (close similarities), and dots (more distant similarities), are as defined for the GAP program. The consensus sequences are based on two separate multiple alignments generated for the β-domains of the 52 γ-proteobacterial proteins and the 17 non-γ-proteobacterial proteins, respectively. Symbols in-between the two consensus sequences indicate similarities and identities between these two consensus sequences.

Examination of alignments of the full-length proteins revealed 10 fully conserved residues (R112, G129, N131, R147, G151, E153, N164, Y166, G211, D213 where the numbers refer to the alignment positions in Fig. S2); all were located within the β-domain. Consensus sequences were separately derived for the 52 γ-proteobacterial proteins and for the 17 non-γ-proteobacterial proteins (see Fig. 4). There are 16 fully conserved residues in the former group of proteins and 10 such residues in the latter group.

**Figure 5:**
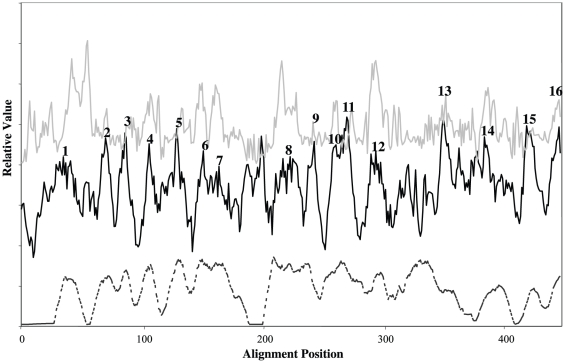
Average hydropathy, amphipathicity, and similarity plots for the β-barrel domains of the 69 Intimin/Invasin proteins included in this study. The plots were generated with the AveHAS program [38]. A window size of 9 residues with the angle set at 180° was used as is appropriate for β-strand analyses. Average hydropathy, dark solid line, middle; Average amphipathicity, faint solid line, top; Average similarity, dashed line, bottom. These plots present relative values as a function of alignment position for all three characteristics.

Despite the amino acid sequence conservation, pore-forming ability and the critical nature of the β-domain for biogenesis, the precise topological organization of the β-barrel domain remains undetermined. The multiple alignment shown in Fig. S2 for the β-barrel domains was used to derive average hydropathy, amphipathicity and similarity plots (Fig. 5). Sixteen peaks of average hydrophobicity coincide with 16 peaks of average similarity, and all of these peaks overlap peaks of amphipathicity (Fig. 5). These characteristics suggest that there may be as many as 16 transmembrane β-strands comprising these barrels.

To further investigate the nature of these transmembrane segments, PRED-TMBB was used to predict the positions of these strands in each of the 69 proteins analyzed (Fig. S3). Some of these 16 peaks of hydrophobicity and similarity proved to be almost universally predicted as transmembrane β-strands, but others were not. Thus, putative TMSs 7–9 were predicted to be transmembrane in every one of the 69 proteins examined, and putative TMSs 10–12 were predicted to be transmembrane in all but one of these proteins. TMSs 1 and 4–6 were predicted to be transmembrane in all but a few of these proteins. The remaining strands were predicted to be transmembrane in less than 50% of the proteins. Thus, these β-barrel domains could consist of as few as 10 β-TMSs and as many as 16. Known β-barrel porins can have as few as 8 and as many as 22 transmembrane β-strands [50], [53].

Interestingly, PsiPred [40] predicted a region between putative β-strands 12 and 13 that has a high propensity for an α-helical conformation and is conserved in all proteins analyzed, (see Fig S3). We have termed this region the α′-domain. Proteins of the AT-1 and AT-2 families possess an α-helical region linking the β-barrel and the passenger domain which spans the pore of the β-barrel and is essential for translocation of the passenger domain to the outside of the cell [54], [55], [56], [57]. The α′-domain may serve a similar function for the Int/Inv family of proteins. If this region acts as a pore domain, analogous to those of the AT-1 and AT-2 autotransporters, β-strands 13, 14, 15 and 16 might be extracellularly localized and not part of the β-barrel *per se*.

We next examined the positions of the fully conserved residues with respect to their positions in the predicted secondary structural model for these β-barrels. All 10 of the fully conserved residues were predicted to occur within or immediately adjacent to putative TMSs 4–8. Examination of this set revealed that 8 of the conserved residues are separated from another fully conserved residue by a single amino acid. The conserved residues are much more hydrophilic than the non-conserved residues that separate them. As these conserved neighboring residues occur on the same side of a β-strand, they presumably form an aqueous channel [24] or the outer surface of a β-turn while the hydrophobic side faces the lipid bilayer. Indeed, the three residues most likely to fit into β-turns (with low propensity for occurrence in an α-helix or a β-strand [58]) are N, G, and D which represent 6 of the 10 fully conserved residues. Consequently, we suggest that some of the fully-conserved residues within these β-barrel domains form surface structures that possibly extend outward on one or the other side of the transmembrane β-barrel. They could play an important role in macromolecular recognition.

The homology observed for this family of β-barrel domains clearly suggests uniform topologies, structures and functions. However, the frequent occurrence of these β-barrel passenger domains may provide a clue as to additional functions these proteins may serve. They probably do not serve only as anchors or transporters, although the possibility of noncovalent protein∶protein interactions mediating anchoring and export of non-attached “passenger” proteins cannot be excluded.

#### Organization of the Passenger Domains

As noted previously, proteins in clusters E, G, H and J are primarily composed of β-barrel domains. While the remaining 48 proteins have C-terminal predicted passenger domains, the Pas1 and Cla1 proteins contain unique passenger domains, different from those in the other 46 passenger domain-containing proteins. The sizes of the proteins vary tremendously (see Table S1). The homologues from clusters A, B, D, E and W show fairly uniform size distributions within each of these clusters, but all remaining clusters show tremendous size variation. Cluster F proteins derive exclusively from one genus in the β-proteobacteria, including four species of *Bordetella*, and they vary in size from 747 amino acids to 937 amino acids (see Table S1 and Figure S1). Branches J and K each includes a single protein, the first from a *Rhodobacter* species, an α-proteobacterium, and the second from *Chlamydia suis*. While the former includes only the β-barrel, the latter has a large passenger domain. All remaining clusters (clusters L-X) include proteins with passenger domains of varying sizes. In these clusters, size variation among the members of each cluster is almost always substantial. We suggest that the latter proteins underwent slow evolutionary subdomain duplication/deletion/insertion compared to the former proteins.

The structures of the C-terminal passenger domains for an intimin and an invasin have been defined by Hamburger et al. (1999) and Luo et al. [5] and shown to consist of repetitive Big motifs which adopt a structure similar to that observed for immunoglobulin domains. For the purposes of this article, we will refer to these smaller repetitive Big elements as subdomains of the larger passenger domain. Thus, in the case of intimins, the subdomains are designated IntD0–D4, and in the case of invasin subdomains, they are designated InvD1–D5. To identify subdomains in the passenger domains of all 48 other proteins, a database containing the passenger domains was constructed and screened using an iterative BLAST (with an e^−5^ cut off), searching for subdomains common to at least two proteins or occurring at least twice within a single protein. Using this method, IntD0 and IntD1, and InvD1, InvD2 and InvD3 were found to be homologous repeats. Thus, for the purposes of this article, all of them are designated D0. The remaining IntD2, IntD3, InvD4 and InvD5 maintain the designations D2, D3, D4 and D5, respectively.

**Figure 6:**
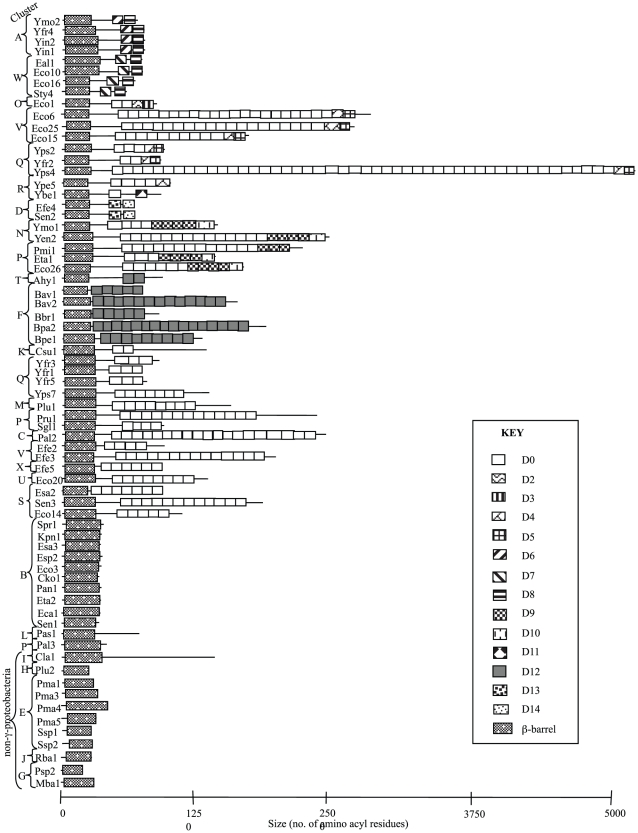
Predicted C-terminal passenger domain compositions. 69 proteins were analyzed for homologous subdomains in the predicted passenger domain regions by BLAST searches with a cutoff value of e^−5^ (see Text).

As a result of this approach, an additional 9 subdomains were identified and designated D6–D14. The positions and numbers of iterations of each subdomain is depicted in Fig. 6. D0 subdomains often occur as repeats. The D0 subdomain is the most common, appearing (usually as internal repeats) in 30 of the 48 proteins and present in the largest homologue (Yps4) 47 times. The remaining domains often show limited distribution reflecting phylogenetic origins.

Tertiary structural predictions revealed that most of these subdomains adopt a structure similar to the Big subdomains (Fig. S4). The passenger subdomains were analyzed for conserved residues by aligning the subdomains, and the positions of these residues were replotted onto the predicted 3-dimensional structures (see Table 1 and Figs. S5, S6, S7, S8, S9, S10, S11, S12, S13, S14, S15, S16). Surprisingly, there was little recognizable amino acid sequence homology between domains that adopt a similar fold. Indeed, no specific amino acid motif could be identified that was common to all subdomains, and those conserved motifs found within specific subdomains (Table 1) generally reflected structural elements within the subdomains, e.g. hydrophobic core residues contributing to the structural integrity of the subdomain. Since these domains exhibit a common fold, it is of considerable interest that they do not exhibit common conserved motifs or show high levels of similarity. This fact may have functional significance.

**Table 1:** Motifs identified in Passenger Sub-domains D0, D4–D10 and D12–D14.

Domain	N-terminal Motif	Central Motif	C-terminal Motif
D0	A(ND)		(LIV)
D4	**FP**XXX**F**	**Y**X(WF)XSS	**G**X(IV)T(FIL)
D5	**W**(V/I/F)	**G**(ST)**L**(WYF)(GS)**E**W**G**(ND)(LM)XXY	**W**
D6	**CP**XXXX**G**XXXX**C**	**EVR**D**G**	**L**V**S**TXX**G**T**F**XXXX**D**XGX**YG**
D7	DGV**V**MDL	LY**D**XX**D**K	**P**
D8			**G**D**QG**(FY)G**L**K**V**
D9			**SY**X**L**X6**P**
D10	**V**XX**L**X**P**XX**IW**		**C**X**K**XXX**G**(ST)**Y**X7**Y**X6**Y**X7**W**X**G**
D12		**V**TVXF**P**D**G**(ST)XK**T**	
D13	See Fig S12		
D14	See Fig S13		

For each motif: boldface, fully-conserved residues.

Conserved motifs identified in passenger subdomains D0, D4–D10 and D12–D14.

Tandem subdomain duplication and deletion is also likely to have occurred with high frequency for some subdomains (e.g., D0, D9 and D12) but not for others (e.g., D4–D8, D10, D11 and D13–D14). These observations are not likely to have arisen by chance and therefore may have both physiological and mechanistic explanations. For example, the C-terminal subdomains in clusters A, N-R, and W are never duplicated. The lack of repetition most likely reflects the functional nature of the final subdomain. In contrast, many of the Big subdomains are repeated, an event which can be explained by the requirement of the Int/Inv protein to span the bacterial surface structures such that the functional domain can be displayed for interaction with the host. Interestingly, the final domain of an Intimin or an Invasin adopts a CLTD fold despite possessing limited amino acid sequence similarity.

**Figure 7:**
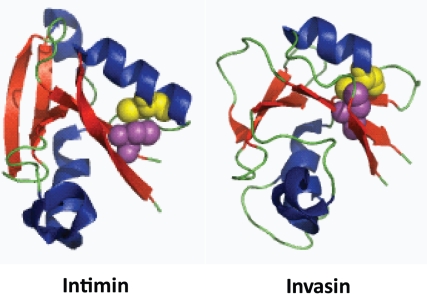
Structures of the C-terminal CLTD subdomains of the Intimin and Invasin passenger domains. The cysteine residues are depicted by spheres with the C-terminal cysteines shown as yellow spheres and the penultimate cysteine as purple spheres. The positioning of the cysteines and the resulting disulphide bonds may stabilize the binding surfaces of both Intimins and Invasins, allowing accurate interactions with their ligands.

In all cases, a pair of disulphide-bonded residues are required to maintain function. In Intimin and Invasin, these have similar spacings (Fig. 7). Examination of the Int/Inv family proteins included in this study revealed paired cysteine residues capable of forming disulphide bonds. Of these proteins, 19 (in clusters A, N, O, P, Q, R, T and V) are predicted to have cysteine residues within the final domain at a spacing similar to that in Intimin and Invasin (67–89 amino acids; proteins indicated in red in Figure 1). They are thus predicted to adopt CTLD folds. The remaining proteins have cysteine residues located in much greater proximity (4–29 amino acids) and are envisaged to adopt different functional folds.

### Genomic Context of Int/Inv Genes

Several of the proteins included in Table S1 are small, lacking a passenger domain or containing only a few repeated sub-domains of typical passenger domains. One such protein is Yfr4 of *Yersinia frederiksenii* in cluster A. Yfr4 proved to be in an operon with three other open reading frames (ORFs). While the invasin has 749 amino acids, the following three ORFs are of 301, 292, and 432 amino acids. TC Blast searches revealed that the first two of these proteins exhibited striking sequence similarity to the repeat units in the passenger domains of AT1 (1.B.12), AT2 (1.B.40), and the Int/Inv (1.B.54) families. The last ORF of 432 amino acids also contained repeats homologous to those described above, but these sequences were far more divergent than the first two ORFs. All four of the encoded proteins possess a signal sequence for export to the periplasm via the general secretory pathway as revealed by the use of SignalP (Bendtsen et al., 2004). It seems reasonable that all four of these encoded proteins represent parts of the passenger domain associated with Yfr4. These observations provide the first evidence that a member of the Int/Inv family may function together with other polypeptide chains that serve to extend the passenger domain. All four of these gene products may play a role in *Yersinia* pathogenesis.

A second example of this type proved to be Ahy1 from *Aeromonas hydrophila* in cluster T. The β-barrel domain protein, Ahy1, is encoded by the first gene of a four cistronic operon. The second and third genes both possess repeat subdomains typical of the Int/Inv family passenger domain. This therefore provides a second example where the two downstream genes probably provide the passenger domain function. Interestingly the fourth gene possesses the GGDEF domain and therefore is likely to be a diguanylate cyclase, which synthesizes cyclic di-GMP. This compound is believed to mediate the transition between planktonic growth and sessile biofilm formation (Ryan et al., 2006; Wolfe and Visick, 2008). It is reasonable to suggest that the first three genes in this operon play an important role in biofilm formation, providing the function of intercellular adhesion. We therefore postulate that the operon is expressed under cyclic di-GMP control and is therefore silent in the planktonic state, but expressed during biofilm generation.

A very dissimilar example proved to be the ten short sequences included in cluster B. These proteins, which show homology with the β-domains of Int/Inv family members, range in size from 417 to 497 amino acids and lack a sizable passenger domain. They thus possess only a β-barrel domain. Surprisingly, we could not identify a signal sequence for these proteins using SignalP, suggesting that if they are exported to the periplasm they must use a pathway other than the general secretory pathway (Saier 2006). Indeed, these proteins lack the first 100 residues that include the signal sequences of other Int/Inv family members that are included in TCDB. This fact raises the possibility that these proteins are cytoplasmic. Genomic context for all ten of these proteins revealed that following the gene for the short putative Int/Inv family member (transcribed in the leftward direction) was a contiguous operon encoding a nitrate/nitrite sensor kinase/response regulator pair (transcribed in the rightward direction), followed by another continguous two cistronic operon encoding an nitrate/nitrite transporter and the α-subunit of a nitrate reductase (transcribed in the leftward direction). In this case we propose that the β-barrel domain serves a totally different function than in the other cases represented. Interestingly Eta2 from *Erwinia tasmaniensis*, also in cluster B, has a signal peptide as well as a passenger domain of moderate size.

Sen2 of *Salmonella enterica* (cluster D) is also of a moderate size (660 amino acids). Consequently, we examined the genomic context of this protein as well. The Int/Inv family protein, Sen2, proved to be in a four cistron operon where the first gene encodes a putative lipoprotein, the second encodes the putative invasin, the third is a hypothetical protein of unknown function, and the fourth possesses a thioredoxin superfamily domain and has been annotated as a putative thiol peroxidase. The operon is preceded by a divergently transcribed *araC*-like gene encoding a transcription factor. The function of the invasin, while difficult to predict, might facilitate lipoprotein export, or serve as an anchor for it. Other short sequences listed in Table S1 were similarly examined for genome context, but in these cases, we were unable to observe relationships that were indicative of function. [59]

### Conclusions and Perspectives

**Figure 8:**
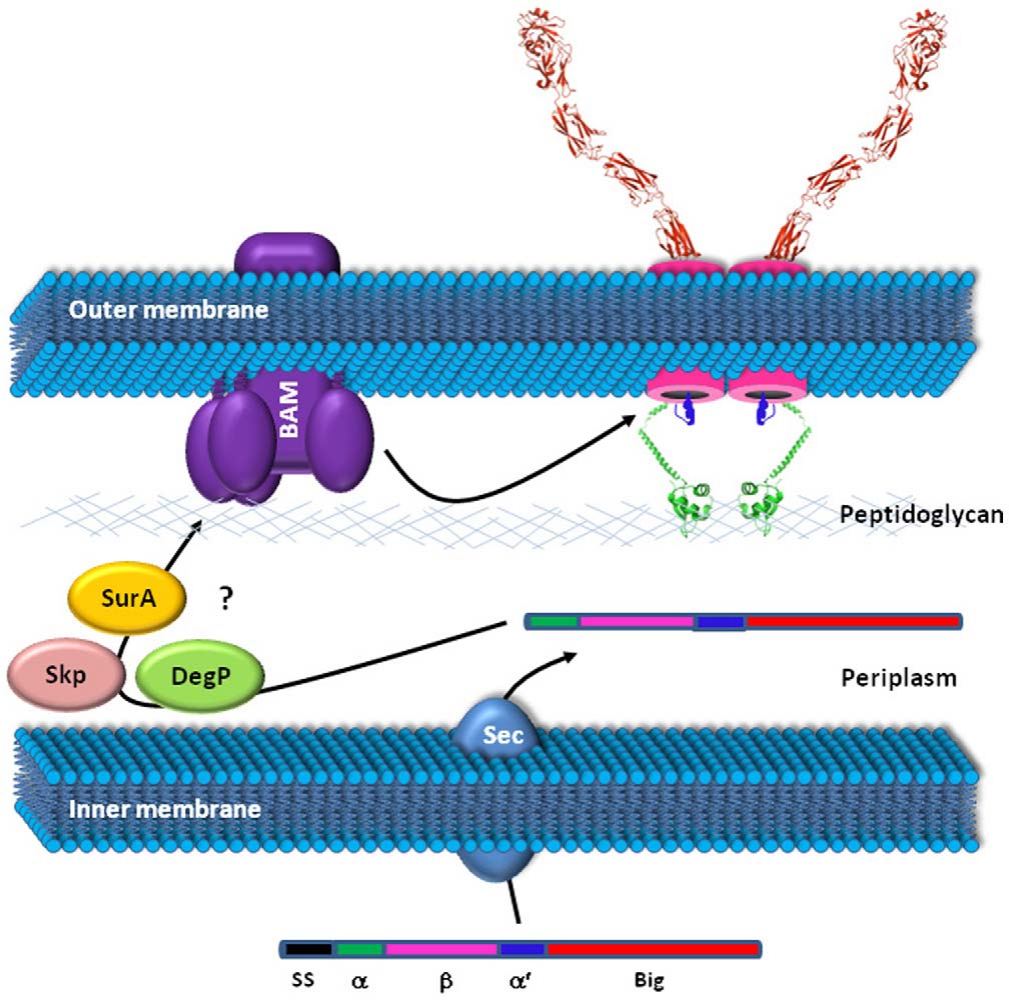
Proposed model for Intimin/Invasin biogenesis. Int/Inv proteins are synthesized as single polypeptides possessing a modular organization consisting of a signal peptide (SS), an α-hydrophilic domain (α), an N-proximal β-domain (β), a second α-helical domain (α′) and a C-terminal passenger domain (Big) which adopts an immunoglobulin-like fold. The signal peptide mediates translocation of the Int/Inv protein across the inner membrane by a post-translational Sec-dependent mechanism. Once periplasmically located, the signal peptide is removed, releasing the remainder of the molecule into the periplasm as an intermediate. The periplasmic intermediate is bound by periplasmic chaperones such as SurA, Skp and DegP and delivered to the β-barrel assembly apparatus (the BAM complex: TC# 1.B.33). The BAM complex facilitates folding of β into a β-barrel structure and insertion of the barrel into the outer membrane, where β adopts a homodimeric conformation. During this process, α remains periplasmically located, and those proteins with a LysM domain interact directly with the peptidoglycan. Such interactions may alter the porosity of the peptidoglycan to allow the bulky disulphide bonded portion of the passenger domain to migrate to the pore formed by β. Once β is inserted correctly into the outer membrane, Big is translocated to the cell surface. It remains unclear whether Big adopts the immunoglobulin-like fold before or after the translocation event. The α′ domain may be inserted into the pore formed by β in a manner analogous to the autotransporters, facilitating translocation of Big to the cell surface and sealing the pore after the translocation event.

The analyses presented here reveal a conserved modular architecture for the Int/Inv family of proteins. Thus, members of this family possess (i) a signal sequence, (ii) a hydrophilic α-domain, sometimes decorated with a LysM domain, (iii) a β-domain with the propensity to adopt a transmembrane β-barrel conformation and (iv) a hydrophilic α′ domain. In addition, the majority of the Int/Inv family members possess a readily identifiable passenger domain that, based on analogy to well characterized Intimin and Invasin, is secreted to the cell surface. These observations allow us to propose a multi-step model for the biogenesis of Int/Inv proteins (Fig. 8). (1) Based on a wealth of scientific endeavours one can reliably predict that the Int/Inv proteins with β-barrel domains studied here are translocated across the inner membrane via the Sec pathway in a post-translational fashion. (2) Recent investigations have demonstrated a role for the outer membrane protein insertion porin BAM complex (TC #1.B.33) and periplasmic chaperones in the biogenesis of Intimin, suggesting that soon after signal sequence cleavage, the Int/Inv proteins are bound by periplasmic chaperones and delivered to the BAM complex. (3) The BAM complex then acts to fold/insert the β-domain into the outer membrane in a β-barrel conformation [60].

Topological predictions for the β-barrel domains suggest that these domains consist of 10 to 16 β-strands, with some being well-conserved and consistently predicted, while others are less certain. Structurally characterized transmembrane β-barrels are of 8 to 22 β-strands [50]. However, multicomponent β-barrels have been demonstrated, e.g. trimeric TolC and the trimeric AT-2 family members. As dimerization of the β-barrel domain has been reported previously, it is possible that the β-barrels form an oligomeric structure spanning the outer membrane [4].

The β-barrel domains have been shown to form transmembrane ion channels and are thus predicted to function in passenger domain secretion across the outer membrane [19], [24]. The mechanism by which this occurs remains stubbornly enigmatic, but, by analogy with the AT-1 and AT-2 families, this translocation event could be mediated by BamA, an essential pore-forming protein in the BAM complex (TC# 1.B.33), or secretion could occur in a vectorial fashion, from the N- to C-terminus, by formation of a hairpin structure that spans the β-barrel pore. Like the AT-1 and AT-2 families of proteins, members of the Int/Inv family possess a conserved α-helical domain (α′) which has the capacity to span the pore formed by the β-barrel. However, in contrast to the AT families, many Int/Inv passenger domains possess cysteine residues which form disulphide bonded loops. Such cysteine bonding arises in the periplasm through interaction with disulphide bond isomerase (DsbA), indicating that the mechanism of passenger domain secretion must be able to accommodate substantially folded elements. From experience with the AT-1 family, this would argue against a monomeric pore formed by the β-barrel domain [6].

It remains unclear what the function of the α-helical α-domains is and what role the LysM domains play. The logical location for these domains is the periplasm. LysM domains are predicted to bind peptidoglycan and are found in peptidoglycan degrading enzymes [47], [48], [49]. It is possible that these domains interact with peptidoglycan to anchor and/or stabilize the β-barrel and the secreted passenger domain in the cell envelope. Alternatively, these domains may form pores through the peptidoglycan allowing the folded elements of the passenger domain to move through the cross-linked peptidoglycan layer so that they can be translocated across the outer membrane. The ability to form pores through the peptidoglycan is essential for some of the other Gram-negative protein secretion machineries, e.g., the Type IV secretion system [7]. It is unclear whether the Big subdomains of the passenger domain are folded into their tertiary conformation prior to translocation across the outer membrane. In the case of the AT-1 proteins, folding of the passenger domain occurs on the cell surface. It is clear that the translocation mechanism can accommodate large folded elements, as noted above, suggesting that the Big subdomains may adopt their tertiary structure before secretion. However, if the α′-domain is a pore spanning domain, the remaining poorly predicted β-strands (those numbered 13–16 in our study) would be located extracellularly, and by analogy with the AT autochaperone domain, they could form a platform for folding of the passenger domains.

The functions of passenger domain-less β-barrels are completely unknown. However, the β-domains undoubtedly provide one or more essential functions such as transport, anchoring, communication via ion channels and/or interaction with cytoskeletal elements underlying the cell membrane. Our genome context analyses suggest that they may be able to translocate/anchor non-covalently linked passenger domains to or on the cell surface as for Yfr4 and Ahy1. Other functions, for example, for cluster B homologues and Sen2 (see section entitled Genomic Context of Int/Inv Genes) seem probable. Perhaps the β-barrels can promote protein-protein interactions on both surfaces of the outer membrane. This possibility could introduce a means of direct communication from the exterior of a two-membrane cell to the periplasm or cytoplasm.

The importance of size, sequence and domain variations in the dissimilar passenger regions of Int/Inv proteins has yet to be studied in detail. It is possible that the multi-subdomain passenger domains merely provide a rigid scaffold for the C-terminal adhesin, but this would not explain the varied compositions of these extracellular structures. Other possibilities include immune evasion for pathogens, escape from predators for free-living organisms, and contribution to their adhesive properties. Comparative analyses of the modular design of passenger subdomains, as recognized previously [61] and as further reported here, suggests that subdomain expansion and contraction has occurred with high frequency over relatively recent evolutionary time. The precise reason for such expansion and contraction can only be guessed at, but it is likely to be due, at least in part, to a requirement to present the C-terminal binding domains beyond the lipopolysaccharide and/or capsular polysaccharide layers of the envelope.

The studies reported here are the first of their kind to describe the unusual family of bacterial intimin and invasin adhesins. We hope it will provide a guide for future studies concerned with the structures, functions, mechanisms of action and evolutionary origins of these proteins.

## Supporting Information

Figure S1 Multiple alignment of the full-length Int/Inv family members.
(5.07 MB PDF)

Figure S2 Multiple alignment of N-terminal β-barrel domains.
(0.37 MB PDF)

Figure S3 Multiple alignment of N-terminal β-barrel domains. Predicted transmembrane β-strands are shaded yellow with predicted β-strands numbered 1 to 16 above the alignment. Fully conserved residues are colored red. Predicted α-helices are shaded blue located between β-strands 12 and 13.
(0.14 MB PDF)

Figure S4 Multiple alignment of C-terminal passenger domains.
(2.82 MB PDF)

Figure S5 Tertiary structural predictions of the Big subdomains comprising the C-terminal passenger domains.
(0.78 MB PDF)

Figure S6 Multiple alignment of passenger subdomain D0.
(0.02 MB PDF)

Figure S7 Multiple alignment of passenger subdomain D4.
(0.01 MB PDF)

Figure S8 Multiple alignment of passenger subdomain D5.
(0.01 MB PDF)

Figure S9 Multiple alignment of passenger subdomain D6.
(0.01 MB PDF)

Figure S10 Multiple alignment of passenger subdomain D7.
(0.01 MB PDF)

Figure S11 Multiple alignment of passenger subdomain D8.
(0.02 MB PDF)

Figure S12 Multiple alignment of passenger subdomain D9.
(0.01 MB PDF)

Figure S13 Multiple alignment of passenger subdomain D10.
(0.01 MB PDF)

Figure S14 Multiple alignment of passenger subdomain D12.
(0.01 MB PDF)

Figure S15 Multiple alignment of passenger subdomain D13.
(0.01 MB PDF)

Figure S16 Multiple alignment of passenger subdomain D14.
(0.01 MB PDF)

Table S1 The sixty-nine proteins of the Intimin/Invasin (Int/Inv) family included in this study, listed according to phylogenetic cluster and position within that cluster. Cluster designations refer to the clustering patterns in the phylogenetic tree shown in Fig 1A. Protein sizes are presented in numbers of amino acyl residues (aas). Greek letters refer to the subcategory (order) of the proteobacteria. Other columns are self-explanatory.
(0.12 MB DOC)
