# Supplementary material for: The Bacterial Intimins and Invasins: A Large and Novel Family of Secreted Proteins
Source: PLoS One. 2010 Dec 22;5(12):e14403. doi: 10.1371/journal.pone.0014403 (PMC3008723; doi:10.1371/journal.pone.0014403)
Supplement: Figure S1 — Multiple alignment of the full-length Int/Inv family members. (5.07 MB PDF) [file pone.0014403.s001.pdf]

CLUSTAL X (1.81.1-alpha) MULTIPLE SEQUENCE ALIGNMENT


```
   Mba1 --------------------------------------------------------------------------------
   Psp2 --------------------------------------------------------------------------------
   Sen2 --------------------------------------------------------------------------------
   Efe4 --------------------------------------------------------------------------------
   Eco3 --------------------------------------------------------------------------------
   Cko1 --------------------------------------------------------------------------------
   Sen1 --------------------------------------------------------------------------------
   Eca1 --------------------------------------------------------------------------------
   Esp2 --------------------------------------------------------------------------------
   Esa3 --------------------------------------------------------------------------------
   Kpn1 --------------------------------------------------------------------------------
   Pan1 --------------------------------------------------------------------------------
   Eta2 --------------------------------------------------------------------------------
   Spr1 --------------------------------------------------------------------------------
   Yin1 --------------------------------------------------------------------------------
   Yin2 --------------------------------------------------------------------------------
   Yfr4 --------------------------------------------------------------------------------
   Ymo2 --------------------------------------------------------------------------------
  Eco10 --------------------------------------------------------------------------------
   Eal1 --------------------------------------------------------------------------------
   Sty4 --------------------------------------------------------------------------------
  Eco16 --------------------------------------------------------------------------------
  Eco25 --------------------------------------------------------------------------------
  Eco15 --------------------------------------------------------------------------------
   Eco6 --------------------------------------------------------------------------------
   Efe2 --------------------------------------------------------------------------------
   Efe3 --------------------------------------------------------------------------------
   Yfr1 --------------------------------------------------------------------------------
   Yfr5 --------------------------------------------------------------------------------
   Yps4 --------------------------------------------------------------------------------
   Yps7 --------------------------------------------------------------------------------
   Yfr2 --------------------------------------------------------------------------------
   Yfr3 --------------------------------------------------------------------------------
   Yps2 --------------------------------------------------------------------------------
   Ype5 --------------------------------------------------------------------------------
   Pru1 --------------------------------------------------------------------------------
   Pal3 --------------------------------------------------------------------------------
   Eta1 --------------------------------------------------------------------------------
   Sgl1 --------------------------------------------------------------------------------
  Eco26 --------------------------------------------------------------------------------
   Pmi1 --------------------------------------------------------------------------------
   Eco1 --------------------------------------------------------------------------------
   Ymo1 --------------------------------------------------------------------------------
   Yen2 --------------------------------------------------------------------------------
   Ybe1 --------------------------------------------------------------------------------
   Esa2 --------------------------------------------------------------------------------
   Sen3 --------------------------------------------------------------------------------
  Eco14 --------------------------------------------------------------------------------
   Bpe1 --------------------------------------------------------------------------------
   Bav2 --------------------------------------------------------------------------------
   Bbr1 --------------------------------------------------------------------------------
   Bpa2 --------------------------------------------------------------------------------
   Bav1 --------------------------------------------------------------------------------
   Pma1 --------------------------------------------------------------------------------
   Pma5 --------------------------------------------------------------------------------
   Pma3 --------------------------------------------------------------------------------
   Pma4 --------------------------------------------------------------------------------
   Ssp1 --------------------------------------------------------------------------------
   Ssp2 --------------------------------------------------------------------------------
  Eco20 --------------------------------------------------------------------------------
   Efe5 --------------------------------------------------------------------------------
   Plu1 --------------------------------------------------------------------------------
   Pal2 --------------------------------------------------------------------------------
   Ahy1 --------------------------------------------------------------------------------
   Csu1 --------------------------------------------------------------------------------
   Plu2 --------------------------------------------------------------------------------
   Rba1 --------------------------------------------------------------------------------
   Pas1 --------------------------------------------------------------------------------
   Cla1 MRTKEGKMKSVIYKKSLKAKYPKKRILRKILLSFLLSSSLINQTHSSVIFDNVDSEIEILSKKIISSTNTDNEILPSSAQ     80
  ruler 1.......10........20........30........40........50........60........70........80
```

CLUSTAL X (1.81.1-alpha) MULTIPLE SEQUENCE ALIGNMENT

File: /Users/saierlab/Desktop/69long.ps **Date: Tue May 12 18:31:19 2009**


```
Mba1   --------------------------------------------------------------------------------
Psp2   --------------------------------------------------------------------------------
Sen2   --------------------------------------------------------------------------------
Efe4   --------------------------------------------------------------------------------
Eco3   --------------------------------------------------------------------------------
Cko1   --------------------------------------------------------------------------------
Sen1   --------------------------------------------------------------------------------
Eca1   ------------------------------------------------------------------------------MV    2
Esp2   ------------------------------------------------------------------------------MV    2
Esa3   ----------------------------------------------------------------------------MRWL    4
Kpn1   -----------------------------------------------------------------------------MPV    3
Pan1   -------------------------------------------------------------------------MIFMPGR    7
Eta2   ---------------------------------------------------------------------------MSPFY    5
Spr1   ------------------------------------------------------------------------MTDVIHKA    8
Yin1   ---------------------------------------------------------------------------MKEPV    5
Yin2   ---------------------------------------------------------------------------MKKPI    5
Yfr4   ---------------------------------------------------------------------------MIKTF    5
Ymo2   ---------------------------------------------------------------------------MENEI    5
Eco10  --------------------------------------------------------------------------------
Eal1   --------------------------------------------------------------------------------
Sty4   --------------------------------------------------------------------------------
Eco16  --------------------------------------------------------------------------------
Eco25  ----------------------------------------------------------------------MATKKRSGEE   10
Eco15  --------------------------------------------------------------------------------
Eco6   ----------------------------------------------------------------------MAGKAHGNGD   10
Efe2   --------------------------------------------------------------------------------
Efe3   --------------------------------------------------------------------------------
Yfr1   --------------------------------------------------------------------------------
Yfr5   --------------------------------------------------------------------------------
Yps4   --------------------------------------------------MLNYFRAILISWKWKLSHHTSRPHDVKEKG   30
Yps7   --------------------------------------------------MSVYMIKYFYFFKKPEPIIGVLPNRQSHHL   30
Yfr2   --------------------------------------------------------------------------------
Yfr3   --------------------------------------------------------------------------------
Yps2   --------------------------------------------------------------------------------
Ype5   --------------------------------------------------------------------------------
Pru1   --------------------------------------------------------------------------MNSGSG    6
Pal3   --------------------------------------------------------------------------------
Eta1   ------------------------------------------------------------------MAFLWSLFMKIKHQ   14
Sgl1   -------------------------------------------------------------------------MKKVYRK    7
Eco26  ------------------------------------------------------------------------MKEIKSSV    8
Pmi1   -----------------------------------------------------------------------MTTLNNVPS    9
Eco1   ----------------------------------------------------------------------------MITH    4
Ymo1   --------------------------------------------------------------------------------
Yen2   ----------------------------------------------------------------------MGSIFKGIER   10
Ybe1   --------------------------------------------------------------------------------
Esa2   --------------------------------------------------------------------------------
Sen3   --------------------------------------------------------------------------------
Eco14  --------------------------------------------------------------------------------
Bpe1   --------------------------------------------------------------------------------
Bav2   --------------------------------------------------------------------------------
Bbr1   --------------------------------------------------------------------------------
Bpa2   --------------------------------------------------------------------------------
Bav1   --------------------------------------------------------------------------------
Pma1   --------------------------------------------------------------------------------
Pma5   --------------------------------------------------------------------------------
Pma3   --------------------------------------------------------------------------------
Pma4   --------------------------------------------------------------------------------
Ssp1   --------------------------------------------------------------------------------
Ssp2   --------------------------------------------------------------------------------
Eco20  --------------------------------------------------------------------------------
Efe5   --------------------------------------------------------------------------------
Plu1   --------------------------------------------------------------------------------
Pal2   --------------------------------------------------------------------------------
Ahy1   ------------------------------------------------------------MNFTPSKVHQFFWTYLFLGQ   20
Csu1   --------------------------------------------------------------------------------
Plu2   --------------------------------------------------------------------------------
Rba1   --------------------------------------------------------------------------------
Pas1   --------------------------------------------------------------------------------
Cla1   NTNTIQSQETQVNEASEIIKEEKDSKELSEQEIYAKYAKINKEIRSIDKELSNNSNNNELIAKKEQLIQEKNNLDNNLKE  160
ruler  ........90.......100.......110.......120.......130.......140.......150.......160
```

```
 Mba1 --------------------------------------------------------------------------------
 Psp2 --------------------------------------------------------------------------------
 Sen2 ---------------------------------------------------------------------------MVFSK     5
 Efe4 ---------------------------------------------------------------------------MKLTP     5
 Eco3 --------------------------------------------------------------------------------
 Cko1 -------------MLLAGGTASAQSSFIQQAEN-----------------------------------------------    20
 Sen1 ----MSRTVFLS-FPLSLLLLIASGTICAQAQD-----------------------------------------------    28
 Eca1 VSPRIRSLFFLP-LLSAGAVYGAPNSFVQQAQN----------------------------------------------    34
 Esp2 AVTRVRNLLLLL-LVPAGAVLAAPNSFMQQAQN----------------------------------------------    34
 Esa3 LVVLPSVVTAAL-LPVRADAASQSDDYVRQAQN----------------------------------------------    36
 Kpn1 SFRLLPTLTFLL-LLPGVPVWALTASDTTRPA------------------------------------------------    34
 Pan1 NDLMFRPVTFSL-AALSALLALPAGALQTDHQVSETPFS-----------------------------------------    45
 Eta2 RCPFLSYLPLAL-LALSGFPSGDAWAFTRQTPVPDAPFA-----------------------------------------    43
 Spr1 RYRLKKVVPFATGCLPAMGLAWLCGALPAYAESPPAP-------------------------------------------    45
 Yin1 RLSIIASSLMGG-IIAFGLTPEIGYAKSSDTRLYKVDSRTSLYQIALQNGLNLRELRKLNNGNLDTRDELNAGESLLLPA    84
 Yin2 RLSIIATLLIGGGVLSCGLIPEMGYAESSPTRIYTVESRTSLYQIALQSGLDLRTLRKLNNGSLDKRDELNAGESLLLPA    85
 Yfr4 RLSVIASLLAGSGMLTLGFLPVPGHAAASSSRIYTVEKPTSLYQLALQSGIQVTELRRLNHGSLDQRDTLEVGESLLLPA    85
 Ymo2 GGTLIN---------------KPGHD------------------------------------------------------    16
Eco10 ---MLRWKRCIILTFISGAAFAAPEIN-----------------------------------------------------    24
 Eal1 ---MVQWMRFIFLTLISGAACAAPEIS-----------------------------------------------------    24
 Sty4 --------------------------------------------------------------------------------
Eco16 --------------------MEPTNIS-----------------------------------------------------     7
Eco25 INDRQILCGMGIKLRRLTAGICLITQLVFPMAAAAQGVVNAATQQPVPAQIAIANANTVPYTLGALESAQSVAERFGISV    90
Eco15 ----------------------------------MWYMLNLRRQFHLPPPLLEN--NTVPYTLGALESAQSVANRFGISL    44
 Eco6 RRGDNTICGLGDRLRRLTAGICLITQTIFPVMAAAPTHINPAHSDTAASLILPN-VKTIPYTLGALESPPTVAARFGITV    89
 Efe2 --------------------------------------MNENTFRHRPLTRAVAFLCIATQVAFPVAATATGTFRVTAPV    42
 Efe3 --------------------------------------MISARFHSSRLTRAVASLCIVTQVLFPVASTAG--HRVAAPQ    40
 Yfr1 ----MRMVALNTLLVQLMLPIVIAFTPLISASARAN-ELDEQMARMRSSGILTEPNTTEPKRGGQSAPILSENPFFAPSL    75
 Yfr5 ------------------------------------------MAKMRSSEILVEPMAVAPKTYSEQPNNHIEKPAVPYYY    38
 Yps4 HPRKIKVVAWITLFFQFAFPLSLSFTPAIAAANTTNSAPTSVITPVNASILPPAARATEPYTLGPGDSIQSIAKKYNITV   110
 Yps7 LPTHIRRVAWGTLLLQLFIPLSVSFSPAIAA------------MKASKADTMVSYSSTEPYVLGSGETVAIVAKKYGITV    98
 Yfr2 ----MKVTAWANILLQLLFPLSLSFTPAIAA------------APKPKATTIS--TTTEPYIIGVGESADTVAKKHGISV    62
 Yfr3 ----------------------------------------------------------MPYTLAPGETVFIVAKKCHLTV    22
 Yps2 ----MVFQPISEFLLIRNAGMSMYFN----------------------KIISFNIISRIVICIFLICGMFMAGASEKYDA    54
 Ype5 -------------------------------------------------MSLYRISSLHQAKQLNKNKQLNKTRISKSVV    31
 Pru1 NHIPHRLKFIAWINIAIQIIFPLASIFTPVAVRAFNEQNDKQHEFLLPKNNRSVFRETQRYTLQVGETAQSIADKYNINL    86
 Pal3 -----------------------------MVASAFTEDRNKHQKPLSQNNKYFFFKETQAYTLQIGETTRSIAEKYHINL    51
 Eta1 QPVPLRIKLFAWVNIGVQSAFPLTMAFTP--AMAGGIDR------HIVETTKKHPPKTRIYILSPGETSVSVAKKFHLTP    86
 Sgl1 KDLSWGLILFTWLNIGIQAIVPLTYAFVPSQASAAGNTK------FLKGSADKATLDTKTYTLATGETTASVAQKYHMSL    81
Eco26 YNHPKMVKMIAILNVLIQLITSVSLTFISYPASSSNFTK------FIPEDKLNTNYKTSNYTLLKGESIRVILERYNIEL    82
 Pmi1 PRPSSITRKVAWLNIFIQLAFPIVTTMPTQTFANENNFK---------KHNISDINQQNTYQVKAGDTPESIAKKFNIKL    80
 Eco1 GFYARTRHKHKLKKTFIMLSAGLGLFFYVNQNSFANGENYFKLGSDSKLLTHDSYQNRLFYTLKTGETVADLSKSQDINL    84
 Ymo1 -----------------------------MSFSTHADSG----------IAKKTALFKRLHTLTATDTLESVASGYGLSV    41
 Yen2 YLCAGFMKKAIAYTQIILQILLGTLPLYSMSFSTQANSD----------ITKKTVLFKQLHTLTPTDTLESVAASYGLSV    80
 Ybe1 ---------------------------MLPRTSQQGDER-------------LAALPTMPYVLKPGETVDMVAKQHNLTL    40
 Esa2 -------------------------------------------------------MHEQSIMEKNTLKISLLKKIVIWAQ    25
 Sen3 ----------------------------------------------------------MRIYLRLTAYFQLVIQVIFLFV    22
Eco14 -------------------------------------------------------------MVKTNPSSSQVRRVAVYGL    19
 Bpe1 ----------------------MNKNIYRVVWSLVRGAWVVAGEWARAGRKSSSPRRQNRQRARRGVAAMVGGSILAQAL    58
 Bav2 ----------------------VNRGMYRVIWSQVRDAWIVVKEGVVSHAKGSG--RNRRRRAQRAASSAVCLSLGMQAA    56
 Bbr1 ----------------------MKQAIHAVAF--------------RHDALARV--GRVHRRR---GAAALAGVLTLQTV    39
 Bpa2 ----------------------MDMNLIPKLT--------------RAHLPARG--RRHWYRRHRAGAAGMSAVLAMQAA    42
 Bav1 -------------------------MLYCSMP----------------------------------SPARLLTLLLCPTL    21
 Pma1 -------------MKISQALTSITL----VFGSILSVSAN--------------EYKFEEIKFNQIPNE-----------    38
 Pma5 -------------MKSPSLLSLVVL----VFGYVLPISAN--------------EYKFEEIKFDQTENK-----------    38
 Pma3 -------------MKIPKRISFLAV----AISSTLSLPAD--------------ELKFKNIEYDQFKEG-----------    38
 Pma4 -------------MKKFSKRSTLALGTLVAFTAFPSAQGDPTLKHKVSALPSNANSDFQEIHIKKPANVNHPADCNIFED    67
 Ssp1 ---MSRTYILFTGLIGWSSLYVPAKAWTQLLNSHDQTKPKQQIEVNNGSAAEQDKPLISEVQLPAEGKAITIGDPLAHQT    77
 Ssp2 -------------MLHRPALALCLAVPLLTSIGTQAAPARPDGPWGRSVSSSIDNDASDRYQWRACKAVAGALEAG----    63
Eco20 ---------------------------------------------------------MSHYKTGHKQPRFRYSVLARCVA    23
 Efe5 -----------------------------------------------------------------------MTMVNKKFK     9
 Plu1 ----MLLKSYPALSEPVKSLIGIENCQEYPEAVIEHN------------------IDSKKQITEQASNKLHENVTYKEKK    58
 Pal2 --------------------------------------------------------------MSSSVSAVKQKLANGFVI    18
 Ahy1 QALFPVVYAAGSLFTFASEQPELIRYLIKKGDTLDSVSRQFGVVPHQLLTLNRHLKLDAPYLVVGETINVPRPQTLPILV   100
 Csu1 --------------------------------------------------------------------------------
 Plu2 --------------------------------------------------------------------------------
 Rba1 --------------------------------------------------------------------------------
 Pas1 ---------------------------------------------------------------MKKKIGYVSAITVALLL    17
 Cla1 INNEEDNTNFQEQTIRKAKVKAHRIISREKPNVKSKQVEIYRENRILDIENIITNETGTKWAKLANKEEYISAKWLEFDD   240
ruler .......170.......180.......190.......200.......210.......220.......230.......240
```

CLUSTAL X (1.81.1-alpha) MULTIPLE SEQUENCE ALIGNMENT


```
Mba1   -----------MNKKQKILLIAPLIVAVSLTQAD---------------------------------------ALKSALE   30
Psp2   --------------------------------------------------------------------------------
Sen2   KPITKYITWAIITSQIS---------LPVIADS---------------------------------------------DN   31
Efe4   TPLARWLAWVLVGTQLLTPAALAQAMLPEITRSG------------------------------------ADSSVDKTDQ   49
Eco3   ---------------------------MAPENHD---------------------------------------GEKHFAE   14
Cko1   ----------PFDNNQDGLPDLG----MTPESHE---------------------------------------GEKQFAE   47
Sen1   ----------PFDQNR--LPDLG----MTPESHE---------------------------------------GEKHFAE   53
Eca1   ----------PFDNNGDSLPDLG----LAKPTSE---------------------------------------GEKHLAE   61
Esp2   ----------PFDNNGDSLPDLG----LAKPNGE---------------------------------------SEKHLAE   61
Esa3   ----------PFDENGDNLPDLG----LAPENNA---------------------------------------AEKHFAR   63
Kpn1   -------------QAQDPLPDMG----IAPQVDD---------------------------------------DARHFAE   58
Pan1   -------DPTRFRQSLDDLPSLG----NVEDNHD---------------------------------------WSKKLAK   75
Eta2   -------DPARFRDMQQHLPELG----KTSDNGQ---------------------------------------LEKKIAE   73
Spr1   --------DSVVQQPANDLPELGG---NASNDAE---------------------------------------REKEWAT   75
Yin1   NSPLFPPEPLGVRVIISDLPEMGMGNAPVPMTEE---------------------------------------NEQKIAA  125
Yin2   NSPLFPLDPLAGKAIASNLPELGMGNDPVPLVSS---------------------------------------GEQKTAA  126
Yfr4   DSPLFPVDASVVAPLANNLPELGMGNDPVSQPDT---------------------------------------DAMKVAG  126
Ymo2   ---------------MPKLPDMAIMAETSGAKPI---------------------------------------SDQQFAD   42
Eco10  -----------VKQNES-LPDLGSQAAQQDEQTN---------------------------------------KGKSLKE   53
Eal1   -----------VGQNESSLPDLGSEAAHQEEQNN---------------------------------------KGKSFKE   54
Sty4   --------------------------------------------------------------------------------
Eco16  -----------STDSPNTLPDLGSSEKSNSKDEK---------------------------------------GALGGKA   37
Eco25  AELRKLNQFRTFARGFDNVRQGDELDVPAQVS----------------------------------ENNLTPPPGNSSG-  135
Eco15  EELRRLNQFRTFARGFDNVRQGEELDVPATTSQK------------------------------SHEQQNAVPPANGEN-   93
Eco6   DELRRLNQFRTFARGFDNVRQGDEIDVPLINSNS------------------------------PEARNLKAMQMERDGK  139
Efe2   ADTTTAATAD----------------------------------------------------------------------   52
Efe3   AAPAVLSEQD----------------------------------------------------------------------   50
Yfr1   PSEAPLNG--STTPLFAPEETSKSITELPDLGS-----------------------------------IQNDIDVNNKLP  118
Yfr5   QPVNPFIG--NEAPVNVPPDS--TLPNLGSENL-----------------------------------PQAESSGANKFN   79
Yps4   DELKKLNAYRTFSKPFASLTTGDEIEVPRKESS-----------------------------------FFSNNPNENNKK  155
Yps7   DELKKINIYRTFSRPFTALTTGDEIDVPHKASP----------------------------------FSVDN--NKDNTL  142
Yfr2   DELKRVNIYRTFSKPFTALTSGDEIDIPRKASP----------------------------------FSIDSEKNKNADV  108
Yfr3   DELKEVNQFRSFSKPFIQLGSGDEIDIPRITPLP-------------------------------EKITTAENAKTVSSS   71
Yps2   NAPQQVQPYSVSSSAFENLHPNNEMESSINPFS----------------------------------ASDTERNAAIIDR  100
Ype5   WANIVIQAIFPLSIAFTPAVMAAETVGASDEKP-------------------------------------------RSAS   68
Pru1   QQLRKLNQFRTFSQN-FENLQPGDELDIPMAPL----------------------------PIVEWDDDKPEIVLPSSAS  137
Pal3   QQLQQLNQFRTFAYS-FENIKPGTEIDIPTSPL----------------------------SPREQENLRP-IVAPEAST  101
Eta1   EALRKLNQRRTFTYG-FDNLQPGDKLNVPAIKLDDEPDVPAARLDNKANLPAARLDNKPDVPAIIWGQEG-SAASALGDD  164
Sgl1   EALRKLNQFRTFARG-FDHLQPGDELDVPLAPL----------------------------PAVTWAEETPVPASASKED  132
Eco26  SELERINQGRVFLNG-IKNIKEGDEINVPVVSF----------------------------APIKWGEEE----TKEQGS  129
Pmi1   FKLIEANPQHVSLTG-ELKINDGTILNIPNEYL----------------------------LKKKWLNNQ----DEAIPS  127
Eco1   STIWSLNKHLYSSESEMMKAAPGQQIILPLKKLPFEYSALPLLGSAPLVAAGGVAG---HTNKLTKMSPDVTKSNMTDDK  161
Ymo1   DELWALNINLYNNRVAFDAIKYGAVVYVPNREE------------------------------------------EQKAT   79
Yen2   DELWALNINLYNNRSAFDAIKYGAVVYVPNQEE------------------------------------------EQQAA  118
Ybe1   AQLKKINQLRTFSKP-FAKLQAGDELEIPQAQS----------------------------------NLGLAPENTALTD   85
Esa2   ILLQIAFPLLVLP--------------AHASSG-------------------------------------------PGAT   48
Sen3   NSFIFSFPAHAATNPDTNQKKPTTEITAQSTAK-------------------------------------------KEED   59
Eco14  AGLQFFFQVTPAFAG--------------------------------------------------------------VFQ   37
Bpe1   LPLSALAQGAPTLRPARVAQ---EEAGQDAAWTR--------------------------------------KLAAQAESL   98
Bav2   APLAVLAQGAPEMTNRPEAG---DIVPSDVLT----------------------------------------QVAVRAQDL   94
Bbr1   AP--AFAQGAPSFSARPAQADRQDAADS---AML--------------------------------------RVAQTARQL   77
Bpa2   APV-AYGQGAPTFSATQVADAASNAVAQPGAVET--------------------------------------RVAQTIQAL   84
Bav1   LPPVAYGS----------------------AIES--------------------------------------EVARNLWTR   42
Pma1   -----------------QNNYEPKDKLDEYIIKG----------------------------------------ANYSTK   61
Pma5   -----------------QNIFEPKDKLDEYIIKA----------------------------------------ATYSTK   61
Pma3   -----------------QSIYEPKDKLEEYIIKG----------------------------------------VTYSTK   61
Pma4   PRSIACIGP-------NDTVYQPKDRLDRIVLKG----------------------------------------ADYAGR  100
Ssp1   WKCDVFMLPRSHACIGPLGIHQPRNLFEHIIQKG----------------------------------------ANYAAL  117
Ssp2   --------QSVRCETLVDADNQSNSTVQKIFVTG----------------------------------------ASYATR   95
Eco20  WANISVQVLFPLAVTFTPVMAARAQHAVQPRLSMG-------------------------------------NTTVTADN   66
Efe5   RKATDAITWSVIATQILSPVSLSLIPANSFASSG---------------------------------------NADIAQT   50
Plu1   RSEPRRCGNFLSKRSVVGKNDSGEKPKILWLDED----------------------------------------SHKDGN   98
Pal2   FTAIWSSAIMPVIPAYAKMLDNKELPSLGSDQIID---------------------------------------ENNTEH   59
Ahy1   SENPLYLKENTESHINVEQKVAQHVTRLGQALTALEEGREAG----------------------FENESEIVKGARRAQR  158
Csu1   ---------------------------------------------------------------------------MCVYR    5
Plu2   -----------------------------------------------------------------------------MGS    3
Rba1   ---------MKQLLLTSTIIALSALPLSAQEVAK----------------------------------------SGKFAT   31
Pas1   ASCYKPNKKKELMVNETQKENLNTIGSEQKRKKR-------------------------------------WLQHQTDDD   60
Cla1   KEVIKEHTHTNFKDKKIIKKPFETVYLENPTNANYYNEN-----------------LKTQKALNDNKKDNNLSKEDQEFS  303
ruler  .......250.......260.......270.......280.......290.......300.......310.......320
```

```
Mba1   MQDAQDKAEIMDLSTMLLAGDVEALKNTAIDGVVEKGVGVTKSFLEQYFP---TVELNFGAQGGSKPSGGLLVVAPLSDP   107
Psp2   ---------------------------------------------MNSYFSDKFP---TAEIGLSTGVTNEVTGSVLVVKPISDP    37
Sen2   EILSWIAGTASSISPHLQEGTLEDYAKGKIKALPGQAANQLVNEGMKSAFPEIIFRGGVNLEDGAKYRSSEFDMFIPVQE   111
Efe4   PEAEWLASRASSLGSLLQEGNISDFAKNQIQALPQTIANDGITSGIKHWLPEAQFRGGITLEDASKYRSAEADLLIPLYQ   129
Eco3   IVKDFGETSM-NDNGLDTGEQAKAFALGKVRDALSQQVNQHVESWLSPWG---NASVDVKVDNEGHFTGSRGSWFVPLQD    90
Cko1   MVKAFGEASM-TDNGLDTGEQARQFAFGQVRDAVSEQVNHQLESWLSPWG---NASVDLRIDNEGSFTGSRGGWFIPWQD   123
Sen1   MAKAFGEASM-KNNALDTGEQARQFAFGQVRDVVSDQVNQQVESWLSAWG---SASVDVKVDNEGHFNGSRGSWFIPLQD   129
Eca1   MAKAFGEASM-TDNGLTTGEQARQFAFGKVRDAVSGEVNEQIESWLSPWG---NASVNVRVDDNGTFNGSSGSWFIPWND   137
Esp2   MAKAFGEASM-TDNGLTTSEQARQFAFGKVRDAVSGEVNEQIESWLSPWG---NASVDLLVDEEGKFNGSSGRWFIPWQD   137
Esa3   VLKAFGEASQ-TDSTLSPGQQARHFAFDRLRDAVSSITSEAESLLSPWG---NATVDLLVDEEGNFNGSSGSLFTPWQD   139
Kpn1   VAKKFGEASM-SDNGLTAGEQAQLFAISKIGNEVS-----HQLESWLSPWG---NANVDLLVDKEGKFTGSKGSWFVPLQD   130
Pan1   AAKSIAEANE-SRSDLTLGEQAGQWAFDRLRDEVATRVESEGQSLLSPYG---NAEFNLNVDMAGNFDGTGGNLFSPLAD   151
Eta2   AAKSIGEASMNSGSDRSLREEAGIWAFNHFRDVAKQRAASEGEQLLSPYG---RASVSLALSDDGSFNGTSAQLLTPWQD   150
Spr1   MAKQLGERNLNNVSSQQVRTRAESYAVG-----QASSVLQQQAQELLSPLG---NAKLSLVMSDQGDFSGSSGQLFSPLYD   148
Yin1   VAQAVGSQNWNTMTGDQMKNQATQWAMGQAKAQAVDPLRQQAQDLLGKFG---KAQVNLAVDDKGSLSKSSFSLFSPWYE   202
Yin2   AAHAVGAQNWNNMTSDQMKNQAESWAKGQAKAQVVDPLRQQAQELLGKFG---KAQVNLAVDDNGSLSKSAFSLFSPWYE   203
Yfr4   TLQNVGAQDWNGMTQDQVKNQAGSWAK----SQAINPLQQQAQDLLGKFG---QAQVSIAVDDHGDFSKSTFSLFTPWYE   199
Ymo2   WGKNLGGQDWNTLNRDKAQSKTTQWAK----EKIISPLQQQAQDLLGRFG---QAQVNLSMDNKGNLNRSTASLFTPWYD   115
Eco10  RGADYVINSATQGFENLTPEALKSQARSYLQSQITSTAQSYIEDTLSPYG---KVRSNLSIGQGGDLDGSSIDYFVPWYD   130
Eal1   QGADYITNSATQGFENLTPEALESQARSYLQSQITSSAQSYIEGALSPYG---KVRSNLSIGQGGDLEGSSIDYFIPWYD   131
Sty4   ---------------------------------------------MSPYG---KIRTSLSVGEGGDLDGSSLDYFVPWYD    32
Eco16  QG--YFISSATQGFENLTPEALKAQAESYAKEQVTSTAQSYLENVLSPYG---SVRTSLSIGEGGDLDGSTFDYFVPWYD   112
Eco25  NLEQQIASTSQQIGSLLAEDMNSEQAANMARGWASSQASGAMTDWLSRFG---TARITLGVDEDFSLKNSQFDFLHPWYE   212
Eco15  TLENQIASTSQRVGTLLSQDMNSEQASGMARGWASSEASGAMTDWLNNFG---TAXISLGVDEDFSLKNSQFDFLHPWYD   170
Eco6   DPQMQVAEVAQQSGTLLARDMDSEQAASMARGWVASSASAQATDWLSRWG---TARVSLGVDEDFSLKSSSFEFLHPWYE   216
Efe2   EDAR-LAGLASQAGSMLTSGVSGDQAADMARGYATGAAQAAFQEWLSQWG---TARVTLSADEHFTLKGSALDLLLPWYD   128
Efe3   ATAAQVAGMTTQAAGMLQSGMNSRQAAEMARGYATSTAQSAFQEWLSQWG---TVRVTLGLDEDFTLKGSAFDLLLPWHD   127
Yfr1   VTEDNVASAATQLWGIMGNDNSSRAAESAVTGVAAGLASQAAADWLGQYG---NARVQLNSNS------IGNADVLIPLTE   190
Yfr5   ITEDNVASTATQFWGVMGNDNSAKAAESAVRGVSSGLAAQAASDWLSQYG---HARVQLNSSG------TGNADILLPLVE   151
Yps4   DVDDLLARNAMGAGKLLSNDNTSDAASNMARSAVTNEINASSQQWLNQFG---TARVQLNVDSDFKLDNSALDLLVPLKD   232
Yps7   SVENTLAGHAVAGATALSNGDVAKSGERMVRSAASNEFNNSAQQWLSQFG---TARVQLNINDDFHLDGSAADVLIPLYD   219
Yfr2   LLENKLASHVQTGATALATSNAAKSSERMIRSAANNEFNSSAQQWLSQFG---TARVQMNVNDDFKLDGSAVDVLVPIYD   185
Yfr3   QYKERLAHNLLKGATVLADDNTPLAAASMARSVAVGEANDAAQHWLSQFG---TARVQLNLDNNLSLKGSAFDMLLPLYD   148
Yps2   ANKEQETEAVNKMISTGARLAASGRASDVAHSMVGDAVNQEIKQWLNRFG---TAQVNLNFDKNFSLKESSLDWLAPWYD   177
Ype5   QAEQSTANAATRLASILTNDDSAKQASSIARGTAANAGNEALQKWFNQFG---SAKVQLNLDEKLSLKGSQLDVLLPLTD   145
Pru1   ENEIRVAQLASQAGKFFSTNPDQEKTKAFARELLTTAASSYAQDWFNRFG---SSQIHLEADKKFSLKNSQIDLLMPWYE   214
Pal3   ENQLILAQFVSKTGQFFTTKPTNEKTKAFARELVTTTASSYLQDWFNHFG---SSQIKLEADKKFSLKNSQIDLLLPLYE   178
Eta1   AGARKMADVASRAGAFLSDNPNGDAALSLARGEVTAEASGQLQQWLNQFG---TARVQLDADEHFSFKNSQFDLLAPLYE   241
Sgl1   LQAQKIAGIASQAGNFLANSPRGDAAASIARGMATGAASTEVQQWLSQFG---TARLQLDVDNKFSLKNSQLDLLIPLYE   209
Eco26  GNLQQIASIATDVGNILSNDNISKNSALLN--KITNKVNSHIQSWFENFG---TAHIQLQVDKNFSLKNSQLELLFPVFE   204
Pmi1   TEGEELAKIIVDNSFLLN---KDIDVTQYAISQISSKSNQKIEQWLNQFG---HARVSLSADKNLTLKNSSAELLIPLYE   201
Eco1   ALNYAAQQAASLGSQLQSRSLNGDYAKDTALGIAGNQASSQLQAWLQHYG---TAEVNLQSGNNFDG--SSLDFLLPFYD   236
Ymo1   QQASLVASHLSQIGSTLSSESRVEAFSRLAKGVLLSSTAKSVEEWLGHIG---KAQVKLQVDDKNDFSGSELHLFVPLYN   156
Yen2   QQASLVASHLSQVGNSLSSENRVDAFSRLAKGILLSSTAKTVEEWLGHIG---QAQVKLQTDDKNDFSGSEIDLFIPLYD   195
Ybe1   TQTTERNLAKTATTSAQMLNSGDKAAARQLRGLAVGNANQAANSWLNNFG---TARLQANVDDRGDLDGSQFDMLMPFYD   162
Esa2   ETDMSDASTLSASLASSAAQNGADAMKNTATHLATTHAASTVEEWLSHFG---TAQVTLDVDDNGNWDNSAFDFLAPLYD   125
Sen3   EAGKNLAAILSSTGSMLSQDNKTDALINSAINNGSAYVTGQIQQWLQQFG---TAKVNLGLDKDLSLDNASLDLLLPLYD   136
Eco14  ADEQSVAQTAMEAGRVLQGSNSGDAARQMLTSQASGQAADAVTQWLNQFG---TAKTQLSVVSDFSLKGSSLDVLLPFYN   114
Bpe1   ARRQAER----QPGARVDGDYLKREAQAQVNDVLRDGVNLARESGLPVFLR---NLQGGLSHDFESGRTSLQLNTIDEVYR   171
Bav2   ARRQADR----REGAQVDADYLKQQGQAQFNQFLQEGVRAANESGLRFLR---NLQGDLRHDFDNGRTSLELRTIDQVYR   167
Bbr1   AQRQAAG---SRASARVDGDLLKGQAEAQANELLQEGVRLANQTELPFLR---RLQGGVNYDFSNKDLSLDLRTIDEVHR   151
Bpa2   AQAREAGGARQDGRASLDGQFLRSQAQAQANVLVQQGVQWANETGLPWLR---RLEGNVSYDFSGRDVAVDVRTIDALHL   161
Bav1   AQHPDTS---------------PGLAQSALDAGVAAGLQASRQTGLPWLR---HLDGGLRYDLDPGRLSFSLRTIDDLMV   104
Pma1   FVPLMNNGAKGDEYTGIMADDLNRLLVDAGFDFANAKANGEIQKIPFFAQT--SVNISGGTESDTSFSINSLMKLGELAK   139
Pma5   FVPLINDGAERNEYTDLIFSDGKRILADAGYDFVNSTANSSIQSIPFFAQT--SLNIGGGTESDTSFSLNSLMKLGELAK   139
Pma3   FVPLINDGAEGSEYTSIMTNDFKKLLVDAGFDLANSTANSQIQKIPFFAQT--NFNISGGSESDTSFSINSLMKLGELAK   139
Pma4   FVPLLNNNAHGSAYTDLIVNDGKSLISDAGYGLVNSYSNQQIQKIPFFAQT--TVSINGTSNSETSFSLDSLMKLREMAT   178
Ssp1   YGPSMVN-SNGVDLGGLIQTELSRTLISSG----VSYANKQIKKIPFFAQT--TLGLDAATSSDLTGYLDSFMRLKTIGY   190
Ssp2   IFPLLNSASLSDGIQKMLWMDSKSFIVSFAHDYLNEYV---LKQIPFLSQT--EFGVGFESDADMTYYLNSLISLAQLGS   170
Eco20  NVEKNVASFAANAGTFLSSQPDSDATRNFITGMATAKANQEIQEWLGKYG---TARVKLNVDKDFSLKDSSLEMLYPIYD   143
Efe5   YANDEHANKLASFAASAGQSLTNNNASSFAVNTLSAQATKEVVDWLQQYG---NARIKLNVDDSFSLKDAEFDFLYPWMD   127
Plu1   HPLPPLILSHGTKILGLLNSDPKKLAQDYIVNKLNSQITSNTQKWLSQFG---TAKINLNVDHRGRLDESSVDLLVPFYD   175
Pal2   LAAEYTKTVGTFLSQKKTMKDLSQIAQDYARNKVSSEATKEIEHWLSKAG---NVKLNIDFDKKFSIKNSQFDWLIPWYD   136
Ahy1   NSIAASGEQVPTSASRYGSEQEVQYWRQQLATQFEEEANAYAASLLGAMG---TARTRVTLDDDFNMVTAEADLLLPLAE   235
Csu1   SKQLFLFLCLNPALLSLLGNEKDISVQNFLTSYSQDNPWSGLGSLRFDFFP---------GDREYPFERTAIDWLFPFYD    76
Plu2   RFIFQVSISRNTLNPTLMKKFIPLSILLATLGATNAQADGQD-----------TPPQATTNAPSWVTSWDVTVPCPVYV    71
Rba1   IVKNIGNALNIGQGEEAVESEVNTLAVDAANAGLDQVEDKVLSTSNFTHFELSVGSDTMGLDKNKSDTKTEAMTVYRLKE   111
Pas1   ATQGGDIPKSAMSGKRWLQHQTNDDVMQGSDISKSGIADMGFAALQPETEKSAGEVRANLPLSDGKLTSGSIDLFYPLYD   140
Cla1   NKVMKVIQTAGAIYDSEDSKSKEEIVKNMASSYLNTSANELAKEFIDSLNTSINTDFSFNYNERSGFSGNAKALLPIVSE   383
ruler  .......330.......340.......350.......360.......370.......380.......390.......400
```

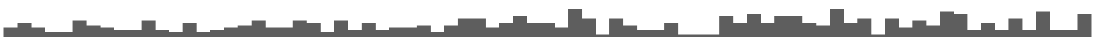

```
                                  . .   *                *   * . .           * . * *
 Mba1 DDIF-NTYFTQGSVFYEDN-----RTTLNLGLGYRKLSDN-KM-----LLTGINAFYDHEFP---YDHGRTSIGLEARTT   172
 Psp2 SDNE-NIIFTQASLFLSDDS----RETINLGFGNRKLIND-DT-----LLVGYNLFYDHELD---YDHQRASIGIEAISS   103
 Sen2 TTS--SLLFGQLGFRDHDSSSFDGRTFANVGMGYRQEVNG--------WLLGVNTFLDADIR---YSHLRGGIGGEVYKE   178
 Efe4 STS--SILFGQLGLRDHDNNSFNGRFFVNTGIGWRQDVGD--------WLLGINSFLDADVR---YDHLRGSLGVELFRD   196
 Eco3 NDR--YLTWSQLGLTQQDD-----GLVSNVGVGQRWARG--N------WLVGYNTFYDNLLD---ENLQRAGFGAEAWGE   152
 Cko1 NTR--YLTWSQLGLTQQDD-----GLVSNVGIGQRWARD--G------WLLGYNTFYDNLLD---ENLQRAGFGAEAWGE   185
 Sen1 KQR--YLTWSLLGLTQQTD-----GLVSNIGVGQRWVQN--G------WLLGYNTFYDNLLD---ENLQRAGVGAEAWGE   191
 Eca1 NNR--YLSWSQLGLTQQTD-----GLVSNAGVGQRWVAG--N------WLLGYNTFYDNLLD---ENLQRAGLGAEAWGQ   199
 Esp2 NNR--YLSWSQLGLTQQTD-----GLVSNAGIGQRWVAG--K------WLLGYNTFYDNLLD---ENLQRAGLGAEAWGE   199
 Esa3 NNR--YLTWSQVGVSQQNQ-----GLVGNAGIGQRWAAG--H------WLLGYNTFYDRLFD---DDTSRAGFGAEAWGD   201
 Kpn1 NDR--YLTWNQYSVTRREH-----DLVGNIGLGQRWRVG--G------WLLGYNSFYDKVLS---ESLARGSVGAEAWGE   192
 Pan1 ENR--YLTFSQLGLHDSVE-----GVVGNAGLGQRWDAG--N------WLLGYNSFIDRSFR---TGLQRASVGTEAWSN   213
 Eta2 NYQ--YLTFSQLGIEQSEY-----GTVGNAGLGQRWIAG--S------WRVGYNAFVDGLLG---SDRQRGSLGAEAWGE   212
 Spr1 VNG--LLTYSQLGLLQQTE-----GSLGNFGLGQRWVAG--D------WLLGYNTVLDSDFE---RHHNRASLGAEAWGD   210
 Yin1 NDA--MVAFSQVGIHGQDG-----RMIGNLGAGVRFDQG--D------WLLGANTFFDQDIS---RNHSRLGLGLEWWAD   264
 Yin2 NDA--MVAFSQVGVHRQDN-----RMIGNLGAGVRFDQG--D------WLFGANTFLDQDIS---RNHSRLGLGLEWWAD   265
 Yfr4 NDA--MVAFSQVGIHDQDS-----RTIGNFGAGLRWDKG--N------WLLGYNTFLDQDFS---RNHSRLGLGAELWTE   261
 Ymo2 SEQ--YLLFSQINIHHQDN-----RKIGNFGLGHRIELPSLN------GLLGYNVFIDHDFS---RGHNRAGIGAEARAD   179
Eco10 NQT--TVYFSQFSAQRKED-----RTIGNIGLGVRYNFD--K------YLLGGNIFYDYDFT---RGHRRLGLGAEAWTD   192
 Eal1 DQS--TVYFSQFSAQRKEE-----RTIGNAGIGVRHNFD--K------WLLGGNIFYDYDFT---RGHRRLGLGTEAWTD   193
 Sty4 NEK--TLLFSQLSAQRKED-----RTIGNFGLGVRQNVG--N------WLLGGNAFYDYDFT---RGHRRLGLGTEAWTD    94
Eco16 NQE--TLVFSQFSAQRKDD-----RTIGNIGVGVRQNIG--S------WLLGGNLFYDYDFT---RGHRRLGLGSEAWTD   174
Eco25 TPD--NLFFSQHTLHRTDE-----RTQINNGLGWRHFTPT--------WMSGINFFFDHDLS---RYHSRAGIGAEYWRD   274
Eco15 TPD--YLLFSQHTLHRTDD-----RTQINTGLGWRHFTSS--------WMSGINLFFDHDLS---RYHSRAGLGAEYWRD   232
 Eco6 TPD--NLVFSQHTLHRTDD-----RTQTNHGIGWRYFTSS--------WMSGVNMFIDHDLT---RYHTRTGMGVEYWRD   278
 Efe2 TPE--NIIFTQHSIHRTDD-----RNQLNTGTGWRHFMPD--------YMTGVNLFFDHDLT---RYHSRMGLGGEYWRD   190
 Efe3 TPE--NLLFTQHSFHRTDD-----RNQLNTGAGWRHFAPD--------YMAGVNLFFDHDLT---RYHSRMGLGGEYWRD   189
 Yfr1 TQN--NLLFGQLGVRYNGE-----RTTNNVGLGVRSFTDS--------WMFGVNTFYDYDLT---GKNSRLGVGGEAWTD   252
 Yfr5 SQD--NLLFGQLGVRYDGY-----RTTNNAGIGVRQFTEN--------WMFGVNSFYDYDLT---GKNSRVGVGAEAWTD   213
 Yps4 SES--SLLFTQLGVRNKDS-----RNTVNIGAGIRQYQGD--------WMYGANTFFDNDLT---GKNRRVGVGAEVATD   294
 Yps7 NEK--SILFTQLGARNKDS-----RNTVNMGAGVRTFQGN--------WMYGANTFFDNDLT---GKNRRIGVGAEAWTD   281
 Yfr2 NQK--SILFTQLGARNKDN-----RNTVNIGAGVRTFQNN--------WMYGVNTFFDNDMT---GKNRRVGVGAEAWTD   247
 Yfr3 DQK--SLLFSQFGLRNHDS-----RNTINIGAGVRTLQDN--------WMYGANVFFDRDIT---GKNNRIGFGAEAWTD   210
 Yps2 SAS--FLFFSQLGIRNKDS-----RNTLNLGVGIRTLENG--------WLYGLNTFYDNDLT---GHNHRIGLGAEAWTD   239
 Ype5 SPD--LLTFTQLGGRYIDD-----RVTLNVGLGQRHFFAQ--------QMLGYNLFIDHDAS---YSHTRIGVGAEYGRD   207
 Pru1 TED--NLIFSQTSLHRKEG-----RIETNLGLGARWYGEG--------QMIGGNTFFDYDIS---RKHSRLGLGVEYRRD   276
 Pal3 TEN--NLLFTQTSLHRKEG-----RIETNLGLGTRWYGEN--------QMIGGNSFFDYDIS---RKHSRLGLGVEYRRD   240
 Eta1 QKD--SLIFTQGSLHRTDD-----RTQVNLGFGLRYFAPS--------YMLGGNIFGDYDLS---RAHSRTGIGMEYWRD   303
 Sgl1 QPD--KLVFTQGSLHRTDD-----RTQTNLGMGMRWFNDG--------YMLGGNTFLDYDLS---RDHARMGMGVEYWRD   271
Eco26 DDE--RLFFSQGGISYIDD-----KFISNIGIGYRAFYDN--------WMLGGNSFIDYDLR---KEHSRLGLGIEYWQD   266
 Pmi1 QKE--KLIFAQTNYHRKDL-----RSQFNYGIGYRYFTEK--------FMVGINGFYDHDLT---HHHNRLGIGAEIWRD   263
 Eco1 SEK--MLAFGQVGARYIDS-----RFTANLGAGQRFFLPE--------NMLGYNVFIDQDFS---GDNTRLGIGGEYWRD   298
 Ymo1 QPE--RLAFSQFGFRRIDQ-----RNIMNIGLGQRHYLSD--------WMLGYNVFLDQQISG--NAHRRLGLGGELARD   219
 Yen2 QPE--KLAFSQFGFRRIDQ-----RNIMNIGLGQRHYVSD--------WMFGYNIFFDQQVSG--NAHRRVGFGGELARD   258
 Ybe1 TPS--QMAFTQFGIRRIDK-----RTTANLGIGIRHFIDD--------WMVGYNLFLDRDIT---RDHTRVGAGAEYARD   224
 Esa2 NKK--SVLFTQLGIRAPDG-----RTTGNIGLGVRTFYVR--D-----WMFGGNVFFDDDFTG---ENRRIGFGAEAWTN   188
 Sen3 DKKQ-NLLFTQWGGRRDDD-----RNIINVGMGYR-YFAD--R-----WMWGINTFYDRQISD--NAHERLGIGGELGWN   200
Eco14 TPK--NVLFTQLGMRDNDG-----RFTTNAGLGHRYFTDN--G-----WMLGYNVFYDVDWRN---TNRRYGIGVEAWRD   177
 Bpe1 AGR--NTGLLQLGAHNQND-----RPTANAGAVYRREVND--A-----LMVGANGFLDYEFG---KQHLRGSVGLEVIAP   234
 Bav2 KGA--NTGLLQLGGHNQNN-----RPTANLGGVYRRDINE--R-----LMLGANAFLDYEFA---KQHLRGSLGVEAIAP   230
 Bbr1 GER--DRVLLQLSGHNRNH-----RPTVNGGVVLRHALNQ--H-----MAVGANAFLDYEFG---KNHLRGSLGGEVIAP   214
 Bpa2 DQD--RALLLQLGGHNQNH-----RPTVNAGVVARSAAGS--S-----LILGGNAFLDYEVG---KRHLRGSLGAEAVAA   224
 Bav1 SER--RALMLQAGLHNQNQ-----RPTANTGIVLRQQASP--G-----LIVGSNAFLDYEFG---KQHVRGSLGLEAIAP   167
 Pma1 DDQG-DLKTLAFSQARFATATNAEGSTINIGLGIRNRPDD--I-----SMVGANAFWDYRMTDYSDAHSRLGLGGEYFWK   211
 Pma5 DDEG-DIKTLAFSQARLATATNAEGSTTNLGLGIRHRPND--V-----SMLGANAFWDYRMTDYSDAHSRLGLGGEYLWK   211
 Pma3 DEEG-DLKTLAFSQARFATATNADGSTFNLGLGIRNRPND--I-----SMVGANAFWDYRMTDYSDAHSRLGLGGEYFWK   211
 Pma4 DDEG-DIKTLLFSQAKLSKTSNSDGTTANLGLGIRHRPNN--E-----SMMGANAFLDYRMTDYSDSHSRLGLGGEYLKN   250
 Ssp1 DNEG-DPMGLMFGQARVTLETSAQ-PQVNVGLGSRFRLGD--E-----AIVGLNGFWDLRTTNYSTAYTRWGIGAEGFWK   261
 Ssp2 DDNG-YPLGLLFAQG-SAKGAYSGSAVTNLGLGLRRRLRD--N-----AMLGANAFWDYRFTNYSSSYSRWGAGAELWWD   241
Eco20 TPT--NMLFTQGAIHRTDD-----RTQSNIGFGWRHFSGN--D-----WMAGVNTFIDHDLS---RSHTRIGVGAEYWRD   206
 Efe5 TKD--YVLFSQTSLHRTDD-----RNQANIGIGVRHFTPD--N-----AMLGANIFYDYDLT---RSHSRAGFGVEYWRD   190
 Plu1 DKDH-WLVYSQYGYRHKDS-----RDTVNLGIGTRLFINN--------WMYGANTFYDNDLT---GNNSRFSLGGELWTN   238
 Pal2 QED--ILLFTQHTLHRYDE-----RFHTNNGIGLRYFHEK--------STIGMNAFIDHDLS---HAHTRVGLGVEYWQD   198
 Ahy1 EQQ--TLLFTQFGLRRNGQD----RTIANLGVGQRHFLDR--------WMLGYNLFADYDLT---NRHWRAGVGAEAWRD   298
 Csu1 SLE--FLFFSQLGFRHNLDR----RDIINLGGGGRFFRKN--------WMLGANAFCDCDLTG---TNTRLGLGLESWTN   139
 Plu2 SENQADNIFFEGGFDYQDA-----RKTVDGALGYRHLMSD-NK-----VMLGANVLYSHEFP---RNHQRISYGAEIRTS   137
 Rba1 TGN--WFLFNQTSAVNFNN-----RTTINTGFGARHINDAN-T-----VITGYNIFYDYELQS---KHERVGAGLELLSS   175
 Pas1 GDS--RLFFGQVGARRFDG-----RNIVNLGIGQRYFQGD--------WALGYNTFYDIQISG--NAHQRLGFGLEYWRD   203
 Cla1 DNPK-ISYFLQSGIGEFAN----DRTIGHFGGGIRYYPNATALNNSGNIMLGLNSVYDHDFS---RGHKRMSLGAEAMVD   455
ruler .......410.......420.......430.......440.......450.......460.......470.......480
```

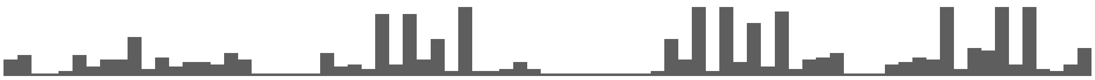

```
             * *                                            * *
 Mba1 VWEINANKYWATTKWKTGK-------------------------NGLEERALDGYDIEAGVPLPYMNWATVFVKNFQWDS 227
 Psp2 VGSLRANQYYGLSGWKSGL-------------------------NNINEKALNGSDVELGMPLPYLPWTNLYYRSFNWEG 158
 Sen2 SLAFSGNYYFPLTGWRTS--AA---------------------HEFHDERPAYGFDLRTKGTLPDFPWFSGELTYEQYYG 235
 Efe4 SMSLAGNWYFPLSDWKAS--KV---------------------QPLHDERPATGIDVRLKGALPSLPWFGAELAFEQYFG 253
 Eco3 YLRLSANFYQPFAAWHEQ-------------------------TATQEQRMARGYDLTARMRMPFYQHLNTSVSVEQYFG 207
 Cko1 YLRLSANYYQPFASWHEQ-------------------------TTALEQRMARGYDVTAQMRMPFYQHLNTSVSVEQYFG 240
 Sen1 YLRLSANYYQPFADWQTH-------------------------TATLEQRMARGYDINAQMRLPFYQHINTSVSLEQYFG 246
 Eca1 NLRLSANYYQPFAGWRDD-------------------------SDVQEQRMARGYDVTAKAWLPWFHHLNTSVSFEQYFG 254
 Esp2 NLRLSANYYQPFASWRDS-------------------------SDLQEQRMARGYDVTAKAWLPYFHHLNTSVSFEQYFG 254
 Esa3 YLRLSANYYQPLGGWQHR-------------------------AGLLEQRMARGYDVTAQAYLPFYQHINTSVSFEQYFG 256
 Kpn1 YLRLSANYYHPLGDWQLRD------------------------NQTQEQRMAAGYDVTAQARLPFYQHINTSVSVEQYFG 248
 Pan1 NLRFSANYYHPLSGWRNR-------------------------GDSQQSRMAEGYDITTQSYLPFYRQLGVSFSYQQYLG 268
 Eta2 YLRLSANYYHPLSGWRNR-------------------------SNSSQMRMARGYDITTRGYLPFYHQLGVTLSYEQYLG 267
 Spr1 FLRFSANYYYPLSALAQQR--D---------------------NAQFLSRPASGYDITTQGYLPFYRQIGGSLSYEQYWG 267
 Yin1 NLKLATNYYHPLSGWKDSK--D---------------------FDDYLERPARGFDVRAQGYLPAYQQLGASVVYEQYYG 321
 Yin2 NLKLASNYYHPLSGWKDSK--D---------------------FDDYLERPARGFDVHAQGYLPAYQQLGASAVYEQYYG 322
 Yfr4 SLRLATNFYHPLSGWKDSK--D---------------------FDDYLERPAKGFDVRLQGYLPAYPHLGASVVYEQYYG 318
 Ymo2 YLKFSANYYHPLSHWKDSP--D---------------------FDDYLERPAKGYDLRSQGYLPAYPQLGVSAVYEHYFG 236
Eco10 YLKFSGNYYHPLSDWKDSE--D---------------------FDFYEERPARGWDIRAEAWLPAYPQLGGKIVFEQYYG 249
 Eal1 YLKFSGNYYHPLSGWKNSE--D---------------------FDFYEERPARGWDIRAEAWLPAYPQLGGKIVFEQYYG 250
 Sty4 YLKFSGNYYHPLSDWKDSE--D---------------------FDFYEERPARGWDIRMESWLPFYPQLGAKLVYEQYYG 151
Eco16 YLKFSGNYYHPLSDWKDSK--D---------------------FDFYEERPARGWDVRAEGWLPAWPQFGAKLVYEQYYG 231
Eco25 YLKLSSNGYLRLTNWRSAP-EL---------------------DNDYEARPANGWDVRAEGWLPAWPHLGGKLVYEQYYG 332
Eco15 YLKLSSNAYIGLTGWRSAP-EL---------------------DNDFEARPANGWDLRAEGWLPAWPQLGGKLVYEQYYG 290
 Eco6 YLKLSGNGYLRLSNWRSAP-EL---------------------DNDYEARPANGWDLRAEGWLPAWPQLGGKLVYEQYYG 336
 Efe2 NLKLGANGYLRLTGWRDAP-EL---------------------DYDYEARPANGWDVRAEGYLPAYPQLGAKLMYEQYYG 248
 Efe3 NLKLGANGYLRLSGWRDAP-EL---------------------DYDYEARPANGWDVRAEGYLPAYPQLGATLMYEQYYG 247
 Yfr1 NLKFSANGYFRLTDWHQSVLAD---------------------MEDYNERPANGFDVRAEAYLPSYPQLGGRLMYEKYFG 311
 Yfr5 NLKFSANGYFRLTDWHQSVLSD---------------------MEDYNERPANGFDVRAAAYLPAYPQLGGSLMYEKYFG 272
 Yps4 YLKFSANTYFGLTGWHQS--RD---------------------FSSYDERPADGFDIRTEAYLPAYPQLGGKLMYEKYRG 351
 Yps7 YLKLSANNYFGITDWHQS--RD---------------------FIDYNERPANGYDLRAEAYLPSYPQLGGKAMYEKYRG 338
 Yfr2 YLKLSANSYIGTSDWHQS--RD---------------------FADYNERPANGYDVRAEAYLPSHPQLGGKLMYEKYRG 304
 Yfr3 YLKLSANSYLRLTDWHQS--RD---------------------FADYNERPANGYDLRVEAYLPAYPQIGTNLKYEQYKG 267
 Yps2 YLQLAANGYFRLNGWHSS--RD---------------------FSDYKERPATGGDLRANAYLPALPQLGGKLMYEQYTG 296
 Ype5 FINLAANGYFGVSGWKNS--PD---------------------LDKYDEKVANGFDLRSEAYLPTLPQLGGKLIYEQYFG 264
 Pru1 FLKLSANSYHRLSGWRSS--RD---------------------LADHSARPSNGWDVRAEGWLPSYPHIGGKLTYEQYYG 333
 Pal3 FLKLSANSYHRLSGWRNS--RD---------------------LADYSTRPANGWDLRAEGWLPIYPHIGGKLTYEQFYG 297
 Eta1 FLKLSANGYLRLSDWNNS--SD---------------------FKDYQERPANGWDIRAQAWLPSLPQLGGKLTYEQYYG 360
 Sgl1 YLKIGANNYLRLTNWRDS--KD---------------------FADYQERPANGWDMSLEGWVPALPQLGGNLKYEQYYG 328
Eco26 NLKLGANSYLRLSNWRNS--SN---------------------IVDYEERPANGLDLNIKSWLPSYPQIGGDIKYEKYYG 323
 Pmi1 YFKLSSNHYHRLSSWRAS--NN---------------------ILDYSERPANGWDIRTEGYFPAYPQLGTKLIFEQYYG 320
 Eco1 YFKSSVNGYFRMSGWHES--YN---------------------KKDYDERPANGFDIRFNGYLPSYPALGARLMYEQYYG 355
 Ymo1 YVKLSANSYYRLGGWKNS--TR---------------------LEDYDERAASGYDIRTEAYLPYYPQLGGKLMYEQYFG 276
 Yen2 YIKLSANSYHRLGGWKNS--TR---------------------LEDYDERAANGYDIRTEAYLPHYPQLGGKLMYEQYFG 315
 Ybe1 YLKLAANGYLRLSDWRDS--PD---------------------FSSYSERPATGFDLRAEAYLPSLPQLGGKLMYEQYFG 281
 Esa2 YLKLSANTYIGTSQWHNS--GD---------------------FDNYNEKPADGYDVRAEGYLPSFPQLGAKLMYEQYYG 245
 Sen3 YFKLSANGYKRLSGWKDS--SE---------------------YEDYQERVANGYDIRAEGYLPAWPQLGAQLVWEQYYG 257
Eco14 YLKLSANGYKRLSDWRQS--PT---------------------VTDYDERPADGWDIRAEGWLPAYPQLGGKLVYEQYYG 234
 Bpe1 EFSLYGNVYAPLSDWKGAK--R---------------------NNRREEKPASGMDVGVGYRPAFAPGLSLSATHFRWNG 291
 Bav2 EFSFYGNVYAPMSGWTGAK--R---------------------DNRREERPASGMDLGMKYSPGFAPGLSLKANYFRWNG 287
 Bbr1 QFTLYGNVYAPMSGWKAAK--R---------------------AERREERPASGWDVGVRLQPEALPGLAIKGQYFRWSG 271
 Bpa2 QFTLYGNVYAPLSGWKAAK--R---------------------AERREERPAAGWDVGFTARPEAVQGLALNAQYFRWRG 281
 Bav1 HYSLYANYYAPLSGWKGAR--R---------------------DSRREERPAAGYDLGGQLS--SDAGLSLQAAYFRWHG 222
 Pma1 DFEFRNNWYMAITNEKDVIIKG----------------------VDYQERVVPGWDLEVGYRLPNNPELAFYIRGFNWDY 269
 Pma5 DFELRNNWYMSITNEKDVTIKG----------------------VSYKERVVPGWDVELGYRLPNNPELAFFVRGFNWDY 269
 Pma3 DFEFRNNWYISITDEKDVTIKG----------------------VSYKERVVPGWDVELGYRLPNNPELAFFVRGFNWDY 269
 Pma4 GWELRNNWYMSMTEQKNVTVNG----------------------TVYTERVVPGWDVEAGYRFPNHPEMAVFVKGFNWDY 308
 Ssp1 SFELRNNWYINGSADKNITINN----------------------IDYVERVVPGWDVEVGYRIPSYPQLAIFVRGFNWDY 319
 Ssp2 DFKLTNNWYIAGTGIKRITTSGRAYTDTTSLAAGTYDETTLLGANTFDERVVPGWDVALNYRLPSYPQLSLGIRGFRWDY 321
Eco20 YLKLSANGYIRASGWKKSP-----------------------DIEDYQERPANGWDIRAEGYLPAWPQLGASLMYEQYYG 263
 Efe5 YLRFGANTYFGLSDWKNSR-----------------------DLADYLERPANGWDVSAEGWLPAYPQLGASVQFEKYYG 247
 Plu1 YLKMSANAYFRLSDWHNAR-----------------------DLVNYYERPANGYDLIADMYLPSMPSLGAKIKYEQYFG 295
 Pal2 YLKLNANSYFGLTSWKSASELN----------------------HDFNAKPAHGWDIQVEGWLPNYPHLGGNLRYEQYYG 256
 Ahy1 YLKLGANFYTPLSSWRDSP-----------------------RFEGMEERAARGMDVRLEAYLPAYPQWSASLTAEQYLG 355
 Csu1 FLQFSMNGYLGLSSPRHSS-----------------------LQPNYQESPAHGLDFRLHYWLPFFPRLGGKIFYEQYFG 196
 Plu2 VFEINSNYYHRLTDWKLTG------------------------VDNNEEKARGGYDVELALAVPYVPSAHFRVKHFCWNG 193
 Rba1 IFEFRANAYQAVSKTLTYN--------------------------GIQETALDGYDAKLTANLPYFYSS-------NLYG 222
 Pas1 YLYLSANGYFGLTDWYSSS-----------------------ALDGYAERAANGYDIRAQGWFPVYPQLSGKLKFEQYFG 260
 Cla1 TLAFNANVYQRLSSWIDSYDFD---------------------KDYVQERPANGWDAKIKYAFPSLINVSFFAKMGQWYG 514
ruler .......490.......500.......510.......520.......530.......540.......550.......560
```

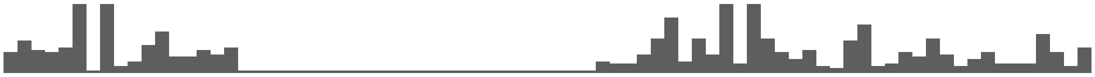

CLUSTAL X (1.81.1-alpha) MULTIPLE SEQUENCE ALIGNMENT

File: /Users/saierlab/Desktop/69long.ps   Date: Tue May 12 18:31:19 2009


```
  Mba1 EISGSKD---------IKGNDLQLRAYIPGITGLEIQAGRTFFSDSSG-TDENYINIFYN----VTQLFAD--KPRYNHQ   291
  Psp2 -ASGAAD---------LEGDEISLEAKLTNFN-IEIGKRS---NDGVT-EDEEFLKITYT----CCNNSN-------NEI   212
  Sen2 DKVDLLG----NGTLSRNPRAAGAALVWNPVPLLEVRAGYRDAGNGGS-QAEGGLRVNYSFGTPLHEQLDY--RNVGAPS   308
  Efe4 DKVDILG----NDSLTRDPAAFTGAITWKPVPLVEIKAGYKDAGSSGS-QTEAGLNLNYTFGVPLRAQLDP--SQVRPAS   326
  Eco3 DRVDLFN----SGTGYHNPVALSLGLNYTPVPLVTVTAQHKQGESGEN-QNNLGLNLNYRFGVPLKKQLSA--GEVAESQ   280
  Cko1 ERVDLFD----SGTGYHNPVAVKLGLNYTPVPLVTVTAQHKQGESGVS-QNNLGLNLNYRFGVPLNKQLSA--SEVAESQ   313
  Sen1 DSVDLFD----SGTGYHNPVALKLGLNYTPVPLLTMTARHKQGESGVS-QNNLGLTLNYRFGVPLKKQLAA--SEVAQSQ   319
  Eca1 DSVDLFN----SGTGYHNPMAVNLGLDYTPVPLVTISAAHKQGESGVS-QNNLGLKLNYRFGVPLAKQLSA--GEVAATR   327
  Esp2 DNVDLFR----SGTGYHNPMAVNLGLDYTPVPLLTFSAAHKQGESGVS-QNNLGMKLNYRFGVPLKKQLSS--EEVATTR   327
  Esa3 DQVELFD----SGTGYHNPVAVKVGLSYTPVPLVTVSAHHRQGESGVS-QNDLGLKLNYRFGVPLTKQLSP--DEVAASR   329
  Kpn1 DSVDLFH----SGTGYHNPVAVSVGLNYTPVPLVTVTAKHKQGENGVS-QNNVGLKLNYRFGVPLKQQLAA--DEVAISN   321
  Pan1 EKVDLFN----SGNRYHNPAALSLGLSYTPVPLVTISASHKTSSAGES-QDQLGLKLNYRFGVALSKQLDA--NNVAEAR   341
  Eta2 DRIDLFN----SGNAVADPSAVSLGINYTPVPLFTLAASRKEGEGGES-QNQFTLKMNYRIGVPLRQQLSS--DNVAEAR   340
  Spr1 ENVDLFG----SGKKQNDPRAMQLGVNYTPVPLVTVKALHKMGEGGVS-QDQVELALNYRLGVPLVKQISP--EYVAQAK   340
  Yin1 DEVALFG----KDNLQKDPSAVTVGVDYTPFPLATLKLIHKRGQQGKN-NTEVGLRVSYQIGTPLDKQLDP--ANVAAMR   394
  Yin2 DEVALFG----KDNLQKDPHAVTVGVDYTPFPLATLKVSHKMGKDGKN-NTELGLQVSYQIGTALEKQLDP--GNVAAMR   395
  Yfr4 DEVALFG----KEHLQKDPRAVTLGLDYTPFPLTTLKVSHKEGQQGQK-EAQVDLQMNYQIGTALSKQLDP--DNVAAMR   391
  Ymo2 DEVALFG----KSHRQKDPRALTLGIDYTPVPLVTLGAKHKYGQQGKK-DTQIDVAFRYQFGSPLSAQLDP--DNVNQLR   309
 Eco10 NEVALFG----TDSLEKDPFAVTLGVKYQPVPLIVVGTDFKAGTGDNT-DLSVNATLNYQFGVPLKDQLDP--DKVSAAH   322
  Eal1 DEVALFG----TDNLEKDPHAVTLGLNYQPVPLITVGTDFKTGTGDNT-DVTVNATLNYQFGTPLKDQLDP--ENVKIAH   323
  Sty4 DEVALFG----TDNLQKDPHAVTLGLEYTPVPLVTVGSDYKAGTGDNN-DFSVKATVNYQIGTPLAAQLDP--ENVKIQH   224
 Eco16 DEVALFG----TDNLEKNPQALTVGLMYNPVPLFTVGSDYKSGTGDNS-DLSVNVSLNYQIGMPLKDQLDP--DNVKAKH   304
 Eco25 DEVALFD----KDDRQSNPHAITAGLNYTPFPLMTFSAEQRQGKQGEN-DTRFAVDFTWQPGSAMQKQLDP--NEVDARR   405
 Eco15 DEVALFD----KNDRQSNPHAITAGLNYTPFPLLTLSAEQRQGKQGEN-DTRFAVDLTWQPSSSMQKQLNP--DEVAGRR   363
  Eco6 DEVALFG----KDERQNDPHAITAGLSYTPVPLISFSAEQRQGKQGEN-DTRIGMELTLQPGHSLQKQLDP--AEVAARR   409
  Efe2 DEVALFG----RDHRQKDPHAFTAGVSYTPVPLVSLSAEQRQGKGGEN-DTRFGLNLSYTPGVSLARQLDP--DAVAYRR   321
  Efe3 DEVALFG----KDKRQQDPHAFTAGLSYTPVPLISLSAEQKQGKGGEN-DTRFALNLTYTPGVSLAHQLDP--DAVAYRR   320
  Yfr1 KGVALNSGSTSPDDLGDSPSAFTVGLNYTPIPLFTVDVAHKKGQNTNN-ELQLGLNFNYRFGVPWVDQINK--NAVGLMR   388
  Yfr5 KGVALNSGSTSPDDLGDSPSAVTLGVNYTPIPLITVDVAHKKGQNTSN-ELQVGLNFNYRFGVPWIDQINK--DTVALMR   349
  Yps4 DEVALFG----KDDRQKDPHAVTLGVNYTPVPLVTIGAEHREGKGNNN-NTSVNVQLNYRMGQPWNDQIDQ--SAVAANR   424
  Yps7 DDVALFG----KDNRQKNPHAITAGVNYTPIPLVTIGAEHRAGKGGQN-DSNINFQLNYRLGETWQSHIDP--SAVAASR   411
  Yfr2 EEVALFG----KDNRQKNPHAVTAGVNYTPIPLLTVGAEHRAGKGSKN-DSSINFQFNYRLGESWQSHINP--SAVAATR   377
  Yfr3 NEVALFG----KDDRQKNPYAFTAGINYTPIPLITIGAEQRAGKGGRN-DTNISIQLNYRLGEPWQSQIDP--SAVAASR   340
  Yps2 ERVALFG----KDNLQRNPYAVTAGINYTPVPLLTVGVDQRMGKSSKH-ETQWNLQMNYRLGESFQSQLSP--SAVAGTR   369
  Ype5 DEVGLFG----VDNRQKNPLAVTLGVNYTPIPLFTVGVDHKMGRAGMN-DTRFNLGFNYAFGTPLTHQLDS--DAVAIKR   337
  Pru1 DSVALFG----TKNLQQNPYSITAGLNYTPIPLVTFNAEHRQGKASKQ-DSRFGLQLNYQFGKTWKQHLDP--GSVTTFR   406
  Pal3 EEAALFG----TKNRQNNPYSITAGINYTPIPLITLNGEHRQGKASKQ-DSRIGLKLSYQFGKSFKQHLDP--DAVGEFR   370
  Eta1 RGVALFG----KENLQQDPRAITAGVNFTPFPLLTLNAEHRQGASGKN-DKRLGVDFSYQLGMPWQQQINP--QAVATMR   433
  Sgl1 KEVALFG----KDNRQKDPHAITVGVNYTPFPLLTFSADQRQGKAGQN-DTRLGVQLNIQLGTPWQHQLDT--SAVGAMR   401
 Eco26 DDVALFG----ENHRQRNPHSTTLGISYTPFPLMSFKAEHKMGSNNIN-DSRIGFEINYQIHTPWESQINP--VLIPAMR   396
  Pmi1 KEVGLFG----KDKRDKNPHTYTLGINYTPIPLVTLNAERRIGLHDRA-DNNLNINLSYRIGESLASQLNP--DNVKAIR   393
  Eco1 DNVALFN----SDKLQSNPGAATVGVNYTPIPLVTMGIDYRHGTGNEN-DLLYSMQFRYQFDKPWSQQIEP--QYVNELR   428
  Ymo1 NEVALFG----LNERQKNPSALTASVSYTPFPLVNLALEHTIGNSGKN-KTGVNLAVNYEINTPWQKQIDP--AAVKATR   349
  Yen2 DEVALFG----INERQKNPSALTAGVSYTPIPLVSLGLDHTIGNGGKK-KTGVNVAVNYEINTPWQQQIDP--AAVQTTR   388
  Ybe1 NDVGLFG----KDNRQQNPAAITAGINYTPIPLVTVGIDRKQGSAGNG-ETLFNLGVNYEVGTPWAKQISP--DAVNARR   354
  Esa2 DNVALFD----KDHLQSNPSAVTVGLNYTPVPLITAGIDYKRGQDSMD-EMKFSLNFHYALDSSWQSQISP--EQVATRR   318
  Sen3 DDVALFDDS--EDDRQRNPYAVTAGVNYTPFPLVSIGLNQKMGKGNHN-DTQIDLAVNWMLGSSLKSQLDS--DAVKARR   332
 Eco14 NEVALFG----ESERQKNPHAITAGVTWTPFSLLTAGVDYRRGKNGAD-DTRLNLGLTYRIGEPLAHQLDS--SRVGAQR   307
  Bpe1 AEVDYFD----NGRTQAGAKGFKVGVEYRPVSLVSVGLEQTKVIGGGR-ETRMQLGLNINLSEPLSKQLRR-DASGTPAF   365
  Bav2 AAVDYFD----NGRTQDRATGFKYGVQYKPVPLLSLGVEQTRVIGGAS-QTSVQLGVALNLSEPLSKQLRR-GGE-TPVF   360
  Bbr1 AAVDYFD----NGRPQRNARGYKYGVEYRPVPLVAVGLEQTKVLGGAR-QTTVQLGVNLSLGEPLSRQLR---HQSGPAF   343
  Bpa2 AQVDYFD----DGRYRRNPSGFKYGIEYRPVPLIGVGVEQARLQSGER-QTSVQLGVRLNLGEPLSRQLRRGAQDTAPPF   356
  Bav1 AGIDVFD----SGRAQRNASGFRYGVAYQPGALFNIGLNQTRTLDGQK-QTSVQLNVRINLQEPPSRQLRR----ESQPF   293
  Pma1 KYTQDNSG--LEGAVSWQATPHVGLEAYVSNEISAASTTANTDLPGTD-ENFFGLRMNIT-GNPVKFEK------SNYKK   339
  Pma5 KNTQDNSG--LEGSVRWQATPHLGWEAYVSNEISAASTTVNTSLPGTD-EIFFGLRVNIT-GNPVTFGK------KDYKK   339
  Pma3 KHTQDNSG--LEGAVSWQATPHVGLEAWVSNEISAASTTVNTSLPGTD-ETFFGLRMNIT-GNPIKFEK------SNYKK   339
  Pma4 KNTQDNSG--IEGSFNWQATPHVNWEAWVSNEISGAKTVKNSDLPKTD-ETFFGLRFRWT-ARPVKFAK------KNLKQ   378
  Ssp1 QDHSDNSG--IEGSVNWQATPHANLELWVSNEIPAYPTDSNDTIGNQP-GPYIGARVRLT-GRPVVFTK------SNTKQ   389
  Ssp2 MRKSDNSG--VEGSVNWQATPHTNLSAWISSEIPAYPAQSNAQLSSGD-DVYVGVRFNVQ-LKPVTYKTGS----NRIRE   393
 Eco20 DEVGLFG----KDKRQKDPHAISAEVTYTPVPLLTLSAGHKQGKSGEN-DTRFGLEVNYRIGEPLAKQLDT--DSIRERR   336
  Efe5 KNVGLFG----SDNLQENPYAVTGEISYTPVPLVKFSAQHRQGQSNTH-DTRFGVEINYRPGVPLSKQLDS--DNVAAMR   320
  Plu1 DNVALFG----KNKRQKDPYAATIGVNYTPIPLITAGIDYKLGKEGKS-DGIFSFNVNYRFGVPLSEQLSP--ENVSSLR   368
  Pal2 DSVALFG----KTKRQKNPNAATIGANWTPFPLFTLNASHKLGSEKQV-ETQAKLQFTWTFGKNLAHHLDP--TKVAETR   329
  Ahy1 ERVGLLD----ADQLERDPHAITAGLHYNPFPLLKMDVEQVEASGRQH-DTRFTLGLEWKLGATLWDMLNP----SSVDK   426
  Csu1 KQVSLFG----RSDLQHDPFVITTGVEFTPFPLLSMEIDHRMSKASRH-YTLFNIRLNYRFGESLSSQLNP--FSVAKMR   269
  Plu2 IASNDSNN---PIDDLKGNTFSVSGSVYDGLSVEVGYIDYTSGNADYS-KAGGERFLKVSYNFDIFGTHVN----KATKP   265
  Rba1 KLSNWKD----AASYETEHYEAGINAEIAPNLTLRVAAQHKKNSNNTE-AVASINYSVPLGGANQPAKVKQDGDWSTKFE   297
  Pas1 DDIALLN----HQNRYKNPYALTMGLEYTPIQLISLGIDRTFSHRGKD-DTKVNLSFNYQLGVPLSQQIDP--TVAPVKR   333
  Cla1 NKVGIFGAN-SVDDLEKNPLIYEGGISYSPFPALTFTLSHSRSAESSKKNTSINANINIPLDEKAMKLAFEP-KLAGISN   592
 ruler .......570.......580.......590.......600.......610.......620.......630.......640
```

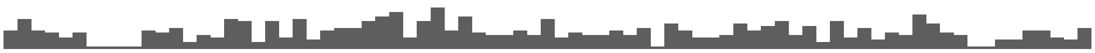

```
  Mba1 WISKDAYKLESMEDRRYEKVRRTN--------------------------------------------------------   315
  Psp2 GISDTAYNLTSVSDQKFAKVRRQN--------------------------------------------------------   236
  Sen2 NTTN-RRAFVDRNYDIVMAYREQAS-------------------------------------------------------   332
  Efe4 NTTN-RTAFVDRNYNIVMEYREQAS-------------------------------------------------------   350
  Eco3 SLRGSRYDNPQRNNLPTLEYRQRK--------------------------------------------------------   304
  Cko1 SLRGSRYDNPQRNNLPTMEYRQRK--------------------------------------------------------   337
  Sen1 SLRGSRYDMPQRNALPTMEYRQRK--------------------------------------------------------   343
  Eca1 SLRGSRYDPAERNSLPVMEFRQRK--------------------------------------------------------   351
  Esp2 SLRGSRYDPVDRTSLPVMEFRQRK--------------------------------------------------------   351
  Esa3 SLRGSRYDRVERTNVPVMEFRQRK--------------------------------------------------------   353
  Kpn1 SLRGSRFDSPERDNLPVVEYRQRK--------------------------------------------------------   345
  Pan1 SLRGSRYDTVTRSQNPVMAYRQRK--------------------------------------------------------   365
  Eta2 SLSGSRYDSVNRDNSPVMAFRQLK--------------------------------------------------------   364
  Spr1 SLRGSRYDNIERKNVPVMAFKQRK--------------------------------------------------------   364
  Yin1 SLKGSRYDLVDRNNDIVLEYKEKA--------------------------------------------------------   418
  Yin2 SLKGSRYDLVDRNYDIVLEYKEKA--------------------------------------------------------   419
  Yfr4 SLMGSRYDRVDRNYDIVLEYKEKAG-------------------------------------------------------   416
  Ymo2 SLKGSRYDLVDRNNDIVLEYKEKQ--------------------------------------------------------   333
 Eco10 SLMGSRHDFVERNNFIVLEYKEKD--------------------------------------------------------   346
  Eal1 SLMGSRHDFVERNNFIVLEYREKD--------------------------------------------------------   347
  Sty4 SLMGSRTDFVDRNNFIILEYREKD--------------------------------------------------------   248
 Eco16 SLMGSRHDFVERNNFIVLEYREQD--------------------------------------------------------   328
 Eco25 SLAGSRFDLVDRNNNIVLEYRKK---------------------------------------------------------   428
 Eco15 SLAGSRYDLIDRNNNIVLEYRKK---------------------------------------------------------   386
  Eco6 SLVGSRYDLVDRNNNIVLEYRKK---------------------------------------------------------   432
  Efe2 SLSGSRHDLVERNNNIVLEYRKK---------------------------------------------------------   344
  Efe3 SLAGSRHDLVERNNNIVLEYRKK---------------------------------------------------------   343
  Yfr1 SLMGSRYDIVDRNYNIVMQYEKQ---------------------------------------------------------   411
  Yfr5 SLAGSRYDLVDRNYNIVMQYEKQ---------------------------------------------------------   372
  Yps4 TLAGSRYDLVERNNNIVLDYKKQ---------------------------------------------------------   447
  Yps7 TLAGSRYDLVERNNHIVLDYQKQ---------------------------------------------------------   434
  Yfr2 TLAGSRYDLVERNNNIVLDYQKQ---------------------------------------------------------   400
  Yfr3 TLAGSRYDLVERNNNIVLEYQKQ---------------------------------------------------------   363
  Yps2 LLAESRYNLVDRNNNIVLEYQKQ---------------------------------------------------------   392
  Ype5 SLMGSRYNLVDRNNQIVMKYRKQ---------------------------------------------------------   360
  Pru1 SLMGNRYDFVSRNNHIVLEYKKN---------------------------------------------------------   429
  Pal3 SLMGNRYDFVSRNNHILLDYKKN---------------------------------------------------------   393
  Eta1 SLAGSRYDLVERNNHILLQYRKK---------------------------------------------------------   456
  Sgl1 TLAGSRYDLVDRNNNIVLEYRKK---------------------------------------------------------   424
 Eco26 KLAGQRYDLVERNNNIILDYRKK---------------------------------------------------------   419
  Pmi1 TLAGSRYDFVNRNNDMILEYKKE---------------------------------------------------------   416
  Eco1 TLSGSRYDLVQRNNNIILEYKKQ---------------------------------------------------------   451
  Ymo1 TLAGSRMDLVDRNNNIVLEYRKQ---------------------------------------------------------   372
  Yen2 TLAGRRMDLVDRNNNIVLEYRKQ---------------------------------------------------------   411
  Ybe1 TLQGSRNDLVERNNQIVLEYKKQ---------------------------------------------------------   377
  Esa2 SLAGSRYDLVDRNNEIILQYKKK---------------------------------------------------------   341
  Sen3 TLLGSRLDLINRNNNIVLEYRKQDLISLKVQNKVTGTESETLPVSVNVKSKYPLDHISWEDDNLVKNGGKISENNGSWSV   412
 Eco14 SLAANRLELVNRNNDVVLEYRKQ---------------------------------------------------------   330
  Bpe1 SPDARRHALVERENRIVLNTRRKE--------------------------------------------------------   389
  Bav2 NLDAHRNALVERENRIVLNTRQKL--------------------------------------------------------   384
  Bbr1 DLQARMGEFVERENRIVLQTRRKH--------------------------------------------------------   367
  Bpa2 DRGARLQDFVRRENRIVLDTRRKK--------------------------------------------------------   380
  Bav1 NLTSRRHQWVERESRIVLNTRRKA--------------------------------------------------------   317
  Pma1 NMVTQMTQPVKRKYDVLLERS-AGG-------------------------------------------------------   363
  Pma5 NMITQMTQPVKREYNVLLERYGANS-------------------------------------------------------   364
  Pma3 NIITQMTQPVKRVNDVLLERAAINSS------------------------------------------------------   365
  Pma4 NLVTQMTQPVRRRYEVLLERSTGG--------------------------------------------------------   402
  Ssp1 NLLTQMTQPVRRRYEVLLERVKEPTK------------------------------------------------------   415
  Ssp2 NLLTQMRQPVQRRNDVLLERWKPKQK------------------------------------------------------   419
 Eco20 VLAGSRYDLVERNNNIVLEYRKSE--------------------------------------------------------   360
  Efe5 EVQNRRYDFVERNNNIVLEYKKKS--------------------------------------------------------   344
  Plu1 SLAGSRYDLVERNNNIILNYLKKQ--------------------------------------------------------   392
  Pal2 RLSGNRYDFVERNNNIILNYQKKT--------------------------------------------------------   353
  Ahy1 SLAGMRHDLIERNNDMVLEYRDK---------------------------------------------------------   449
  Csu1 FLNENRYNFVSRNHVIVFNYSEYT--------------------------------------------------------   293
  Plu2 RFSNTPYEFERMDDRRFEKIRFEQ--------------------------------------------------------   289
  Rba1 PIREKLYRPVQRENRIMKKAIKLG--------------------------------------------------------   321
  Pas1 TLADNRYHLVERNNNIVLKHRERA--------------------------------------------------------   357
  Cla1 TIEGTRTQFIDRDYSMVLEYRATPN-------------------------------------------------------   617
 ruler .......650.......660.......670.......680.......690.......700.......710.......720
```

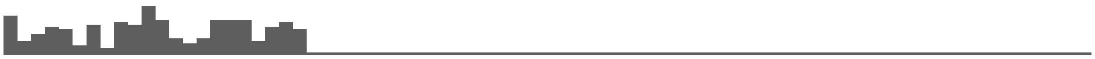

```
  Mba1 --------------------------------------------------------------------------------   315
  Psp2 --------------------------------------------------------------------------------   236
  Sen2 --------------------------------------------------------------------------------   332
  Efe4 --------------------------------------------------------------------------------   350
  Eco3 --------------------------------------------------------------------------------   304
  Cko1 --------------------------------------------------------------------------------   337
  Sen1 --------------------------------------------------------------------------------   343
  Eca1 --------------------------------------------------------------------------------   351
  Esp2 --------------------------------------------------------------------------------   351
  Esa3 --------------------------------------------------------------------------------   353
  Kpn1 --------------------------------------------------------------------------------   345
  Pan1 --------------------------------------------------------------------------------   365
  Eta2 --------------------------------------------------------------------------------   364
  Spr1 --------------------------------------------------------------------------------   364
  Yin1 --------------------------------------------------------------------------------   418
  Yin2 --------------------------------------------------------------------------------   419
  Yfr4 --------------------------------------------------------------------------------   416
  Ymo2 --------------------------------------------------------------------------------   333
 Eco10 --------------------------------------------------------------------------------   346
  Eal1 --------------------------------------------------------------------------------   347
  Sty4 --------------------------------------------------------------------------------   248
 Eco16 --------------------------------------------------------------------------------   328
 Eco25 --------------------------------------------------------------------------------   428
 Eco15 --------------------------------------------------------------------------------   386
  Eco6 --------------------------------------------------------------------------------   432
  Efe2 --------------------------------------------------------------------------------   344
  Efe3 --------------------------------------------------------------------------------   343
  Yfr1 --------------------------------------------------------------------------------   411
  Yfr5 --------------------------------------------------------------------------------   372
  Yps4 --------------------------------------------------------------------------------   447
  Yps7 --------------------------------------------------------------------------------   434
  Yfr2 --------------------------------------------------------------------------------   400
  Yfr3 --------------------------------------------------------------------------------   363
  Yps2 --------------------------------------------------------------------------------   392
  Ype5 --------------------------------------------------------------------------------   360
  Pru1 --------------------------------------------------------------------------------   429
  Pal3 --------------------------------------------------------------------------------   393
  Eta1 --------------------------------------------------------------------------------   456
  Sgl1 --------------------------------------------------------------------------------   424
 Eco26 --------------------------------------------------------------------------------   419
  Pmi1 --------------------------------------------------------------------------------   416
  Eco1 --------------------------------------------------------------------------------   451
  Ymo1 --------------------------------------------------------------------------------   372
  Yen2 --------------------------------------------------------------------------------   411
  Ybe1 --------------------------------------------------------------------------------   377
  Esa2 --------------------------------------------------------------------------------   341
  Sen3 TLPHYQQNSGEKNLYVVSATAWDNQGNKSNASHMTVEVSGFDLHQITSATTTTSATLPADGVSTTQVTVTVTSGNGVKIT   492
 Eco14 --------------------------------------------------------------------------------   330
  Bpe1 --------------------------------------------------------------------------------   389
  Bav2 --------------------------------------------------------------------------------   384
  Bbr1 --------------------------------------------------------------------------------   367
  Bpa2 --------------------------------------------------------------------------------   380
  Bav1 --------------------------------------------------------------------------------   317
  Pma1 --------------------------------------------------------------------------------   363
  Pma5 --------------------------------------------------------------------------------   364
  Pma3 --------------------------------------------------------------------------------   365
  Pma4 --------------------------------------------------------------------------------   402
  Ssp1 --------------------------------------------------------------------------------   415
  Ssp2 --------------------------------------------------------------------------------   419
 Eco20 --------------------------------------------------------------------------------   360
  Efe5 --------------------------------------------------------------------------------   344
  Plu1 --------------------------------------------------------------------------------   392
  Pal2 --------------------------------------------------------------------------------   353
  Ahy1 --------------------------------------------------------------------------------   449
  Csu1 --------------------------------------------------------------------------------   293
  Plu2 --------------------------------------------------------------------------------   289
  Rba1 --------------------------------------------------------------------------------   321
  Pas1 --------------------------------------------------------------------------------   357
  Cla1 --------------------------------------------------------------------------------   617
 ruler .......730.......740.......750.......760.......770.......780.......790.......800
```

```
Mba1   --------------------------------------------------------------------------------   315
Psp2   --------------------------------------------------------------------------------   236
Sen2   --------------------------------------------------------------------------------   332
Efe4   --------------------------------------------------------------------------------   350
Eco3   --------------------------------------------------------------------------------   304
Cko1   --------------------------------------------------------------------------------   337
Sen1   --------------------------------------------------------------------------------   343
Eca1   --------------------------------------------------------------------------------   351
Esp2   --------------------------------------------------------------------------------   351
Esa3   --------------------------------------------------------------------------------   353
Kpn1   --------------------------------------------------------------------------------   345
Pan1   --------------------------------------------------------------------------------   365
Eta2   --------------------------------------------------------------------------------   364
Spr1   --------------------------------------------------------------------------------   364
Yin1   --------------------------------------------------------------------------------   418
Yin2   --------------------------------------------------------------------------------   419
Yfr4   --------------------------------------------------------------------------------   416
Ymo2   --------------------------------------------------------------------------------   333
Eco10  --------------------------------------------------------------------------------   346
Eal1   --------------------------------------------------------------------------------   347
Sty4   --------------------------------------------------------------------------------   248
Eco16  --------------------------------------------------------------------------------   328
Eco25  --------------------------------------------------------------------------------   428
Eco15  --------------------------------------------------------------------------------   386
Eco6   --------------------------------------------------------------------------------   432
Efe2   --------------------------------------------------------------------------------   344
Efe3   --------------------------------------------------------------------------------   343
Yfr1   --------------------------------------------------------------------------------   411
Yfr5   --------------------------------------------------------------------------------   372
Yps4   --------------------------------------------------------------------------------   447
Yps7   --------------------------------------------------------------------------------   434
Yfr2   --------------------------------------------------------------------------------   400
Yfr3   --------------------------------------------------------------------------------   363
Yps2   --------------------------------------------------------------------------------   392
Ype5   --------------------------------------------------------------------------------   360
Pru1   --------------------------------------------------------------------------------   429
Pal3   --------------------------------------------------------------------------------   393
Eta1   --------------------------------------------------------------------------------   456
Sgl1   --------------------------------------------------------------------------------   424
Eco26  --------------------------------------------------------------------------------   419
Pmi1   --------------------------------------------------------------------------------   416
Eco1   --------------------------------------------------------------------------------   451
Ymo1   --------------------------------------------------------------------------------   372
Yen2   --------------------------------------------------------------------------------   411
Ybe1   --------------------------------------------------------------------------------   377
Esa2   ------------------------------------------------------------------------ATSKAVAD   349
Sen3   GLANDLSAQLMRSNSSKGLAADTVQEKISAFKEQSPGVYVSTFTSGTLAGVVTVQPYYNQTSKLSSTTITLTPVSDTARF   572
Eco14  --------------------------------------------------------------------------------   330
Bpe1   --------------------------------------------------------------------------------   389
Bav2   --------------------------------------------------------------------------------   384
Bbr1   --------------------------------------------------------------------------------   367
Bpa2   --------------------------------------------------------------------------------   380
Bav1   --------------------------------------------------------------------------------   317
Pma1   --------------------------------------------------------------------------------   363
Pma5   --------------------------------------------------------------------------------   364
Pma3   --------------------------------------------------------------------------------   365
Pma4   --------------------------------------------------------------------------------   402
Ssp1   --------------------------------------------------------------------------------   415
Ssp2   --------------------------------------------------------------------------------   419
Eco20  --------------------------------------------------------------------------------   360
Efe5   --------------------------------------------------------------------------------   344
Plu1   --------------------------------------------------------------------------------   392
Pal2   --------------------------------------------------------------------------------   353
Ahy1   --------------------------------------------------------------------------------   449
Csu1   --------------------------------------------------------------------------------   293
Plu2   --------------------------------------------------------------------------------   289
Rba1   --------------------------------------------------------------------------------   321
Pas1   --------------------------------------------------------------------------------   357
Cla1   --------------------------------------------------------------------------------   617
ruler  .......810.......820.......830.......840.......850.......860.......870.......880
```

```
Mba1   ---------------------------------------NIVKQIKSLGVKVSGI-------------------------   331
Psp2   ---------------------------------------LIVKQ-KEMDLTVIGF-------------------------   251
Sen2   ----KIRITAMPVSGLSG-----------------TLVTLMATIDSRYPIEKVEWSGDA-------------------EL   372
Efe4   ----RIRVYASPVNGQSG-----------------DTVTLSATINSRYPVERIEWTGDA-------------------EL   390
Eco3   --TLTVFLATPPWDLKPG-----------------ETVPLKLQIRSRYGIRQLIWQGDT----------------QILSL   349
Cko1   --TLTVFLATPPWDLQPG-----------------ETVPLKVQIRSRHGVRHVTWQGDT----------------QALSL   382
Sen1   --TLTVFLATPPWDLTPG-----------------ETVTLKLQVRSVHGIRHLSWQGDT----------------QALSL   388
Eca1   --TLSVYLATPPWDLKGG-----------------ETVMLKLQIRSTHGIRQLHWQGDT----------------QALSL   396
Esp2   --TLSVYLATPPWDLTPG-----------------ETVVLKMQVRSTHGVRQLHWQGDT----------------HALSL   396
Esa3   --TLSVFLATPPWDLSAG-----------------ETVALKLQVRSRHGIRQLSWQGDT----------------QALSL   398
Kpn1   --NLTVYLATPPWDLQSG-----------------ETVQLKLQIHSLHGIKALHWQGDT----------------QALSL   390
Pan1   --TLSVFLATPPWQLNPG-----------------ESVPLKLQLRASHSIKALSWQGDT----------------QALSL   410
Eta2   --TLSVFLATPPWQLQPG-----------------ETLRLKLQIANKNTIKAVSWQGDT----------------QALSL   409
Spr1   --TLQVFLATPPWRLQPG-----------------ETLPLVLEIKTTNKITRVSWQGDT----------------QALSL   409
Yin1   --SLALDLAAVPMTLLEG-----------------DIYLMQPLVRSKYKIVGVTWNGDT----------------VPLSL   463
Yin2   --VLSLDLAAVPMTLLEG-----------------DVYMMQPLVRSKYRITSVSWHGDA----------------VPLLL   464
Yfr4   --LLMLDLAAVPATLLEG-----------------DVYLMQPLVNGKYPIASVSWQGDT----------------VPLAL   461
Ymo2   --VLFADLAAVPDSLMEG-----------------ESYILRPLVKSKYPIIDLIWLGDL----------------LPLQL   378
Eco10  --PLYVTLWLKADVTNEHPECVIKDTPEEAIGLEKCKWTINALINHHYKIVAASWQAKNNAASWQAKNNAARTLVMPVIK   424
Eal1   --HLDVTLWLKADATNEHPECVIKDTPEEAVGLEKCKWTVNALINHHYKIVAASWQAKN---------NAARTLVMPVIK   416
Sty4   --PLDVTLWLKADATNEHPECVIEDTPEAAVGLEKCKWTVNALINHHYKIISASWQAKN---------NAARTLVMPVVK   317
Eco16  --PLDVTLWLKADTANEHPECIVKDTPELAVGLEKCKWTINALINNHYKIVAASWQAKN---------NANRTLVMPVVK   397
Eco25  -ELVRLTLT-DPVTGKSG-----------------EVKSLVSSLQTKYALKGYNVEATALEA--------------AGGK   475
Eco15  -ELIRLSLL-DPVKGKSG-----------------EIKPLVSSLQTKYALKGYNIEAAALEA--------------AGGK   433
Eco6   -ELVRLTLT-DPLKGKPG-----------------EVKSLVSSLQTKYALKGYDIEAASLQS--------------AGGK   479
Efe2   -ELVKLQLN-DPVTGKGG-----------------EQKPLVASLQSKYALKTLQTDAATLTA--------------AGGV   391
Efe3   -ELVKLQLH-DPVTGKGG-----------------EQKPLVASLQSKYALKTLRAEAAELQS--------------AGGV   390
Yfr1   -DLIKLTLP-ETLAAYAI-----------------TNLSLTGNITAKYGAERMEWSAPALMA--------------AGGS   458
Yfr5   -NLINLTLP-ETISAHAL-----------------ENVTLTGTVASKYGTERIEWSAPVLVT--------------TGGA   419
Yps4   -ELIHLVLP-DRISGSGG-----------------GAITLTAQVRAKYGFSRIEWDATPLEN--------------AGGS   494
Yps7   -NLVRLSLP-DSLAGDPF-----------------SQLSVTAQVTATHGLERIDWQSAELMA--------------AGGV   481
Yfr2   -ELIRLSLP-ERVEGKAG-----------------DIATVNAQVTSKYGLERIDWDSAALIA--------------AGGT   447
Yfr3   -DLIQLVLP-NQMTGSAF-----------------EIIKVEAQVTAKYGLKRIDWDTAVIVA--------------AGGV   410
Yps2   -QVVKLTLSPATISGLPG-----------------QVYQVNAQVQGASAVREIVWSDAELIA--------------AGGT   440
Ype5   -NRVTLELP-ARVSGAAR-----------------QTMPLVANATAQQGIDRLEWEASALTL--------------AGGK   407
Pru1   -DVIQLNIA-NSITGYAG-----------------EKIPLSFTVASKYGLSHLKWNAETLVA--------------AGGH   476
Pal3   -DTIYLNMN-ASITGISG-----------------EKIPLKYTINSQYGFNRINWHADSLVA--------------AGGQ   440
Eta1   -EVIRLHTV-GRVTGYAG-----------------ERKSLGVSVNSSYGLERIDWSASSLLA--------------AGGK   503
Sgl1   -EVIHLYTA-DHLAGYAG-----------------EQKSLNVSINTKYGLERIDWSAPELLA--------------AGGK   471
Eco26  -EIIKIDGV-DVISGFSG-----------------EKKRLDIRVNSKYPVDRIDWLANTFIA--------------NGGK   466
Pmi1   -TLVFLSMV-DSINGYAK-----------------EERDLQVQVKTKYPLANIEWSASKLNA--------------QGGQ   463
Eco1   -DILSLNIP-HDINGTER-----------------STQKIQLIVKSKYGLDRIVWDDSSLRS--------------QGGQ   498
Ymo1   -QVVTLNLP-AKVSGKEK-----------------QVLPINYTFNARHGLDRIEWDAADVIN--------------AGGN   419
Yen2   -QVVTLNLP-EKVSGKEK-----------------QVVPINYTFNARHGLDRIEWDAADVIK--------------AGGQ   458
Ybe1   -DVINLYVS-NNVSGRAA-----------------ETKQLVVSVTSKYGLRNIQFDQGALAA--------------AGGK   424
Esa2   MTLATIKNN-SPADGTSA-----------------DTVTLHAVTADGKPAAHAAIVWTVSG---------------NAAL   396
Sen3   ATLSASKTA-ALANARDV-----------------ITLTAHVVDAANNSLQGVAVHWATVNP--------------KAVL   620
Eco14  -TLITLQLP-PDVYGAEL-----------------TTVTLTPQVNAKYGLSRIELDDAELRQ--------------AGGK   377
Bpe1   -IILPLVVSKVSTLQADGRVTVIG----------ATQPFATVTVRMPDGMTGTATADASGRFAYT-----SAGDQPSGVL   453
Bav2   -IILPLTVTTVLTDSVSGRITLVG----------QTQAQATVNWTLPDGSSGQSRADASGVYRIE-----SRKDQPSGPI   448
Bbr1   -VVLPLTIARVDTDPATGRITVTG----------VTEPGAQVSLGLPNGEVVVAQADGSGTYRAT-----SARDMVGGPV   431
Bpa2   -IVLALRIAEVRTDPATGRITVYG----------VTEPLADVQLWLPDGTATSVRANAAGGFEAS-----SAGDMTSGLI   444
Bav1   -ITLPLSIAQLRGDPTNGAIEVSG----------QTEAGARIMLTFPDGSGNWVRADASGRYTAR-----SGPDMPSGTV   381
Pma1   ------------------------------------------VFQNR--AKGV---------------------------   372
Pma5   ------------------------------------------TFTNR--AKGS---------------------------   373
Pma3   ------------------------------------GAA---TFTVRVSAQGT---------------------------   379
Pma4   -------------------------------------------FQNR--ARGI---------------------------   410
Ssp1   ------------------------------------GE----NGSITSIAQGI---------------------------   428
Ssp2   ------------------------------------KTIGGVNGSFINQAAGI---------------------------   436
Eco20  --VIRIALPERIEGKGGQ-----------------TLSLGLVVSKATHGLKNVQWEAPSLLAE--------------GGK   407
Efe5   --TLRISLP-DAIEGESG----------------ATIPVTLNVSHASNGIQSVQWSDSAFAA--------------AGGK   391
Plu1   -QHFRLLVPVIEISSYGG-------------------EVKPIQIQSDTPFKNVTWDIPELFQ-------------KNGGM   439
Pal2   --VLHLSLPSKIQGITG------------------QSVPLVKSFTSKYPLKHIEWQAPEFLA--------------VGGS   399
Ahy1   --VLLKASLNDQYSAVEG-----------------QALTLTLNIQHSRQIASIQWLGDVLG----------------LSG   494
Csu1   ---YDIFVSPASISAIEF-----------------ASSTVSLKCPNTKTLAHFEWEAQNFIEAG-------------GEI   340
Plu2   -----------------------------------------KVISAHNNEQPPA--------------------------   302
Rba1   -----------------------------------------VTVSGY---------------------------------   327
Pas1   --QLSLYLPTGLSGFGG------------------ERKLINFSFNGKYRLKHIQWNDGALRAR--------------GGR   403
Cla1   --KYHISYCGDLGNDKHCILLKNG----------FDEVVKNTPMRVTPTHSCVVFEKGFDYITDN-----------SGKV   674
ruler  .......890.......900.......910.......920.......930.......940.......950.......960
```

```
Mba1   --------------------------------------------------------------------------------   331
Psp2   --------------------------------------------------------------------------------   251
Sen2   IAGLQLQGSLGSGLILPQLPLTATDG---------QEYSLYLTVTDSRGTRVTSERIPVRVTQD----------------   427
Efe4   IGGLQQQGNVNSGLRLPDLSLDVTEN---------KEYSLYLKVTDSRGNSVTSERIPVTVSIN----------------   445
Eco3   TPGAQANSAEGWTLIMPDWQNG---------EGASNHWRLSVVVEDNQGQRVSSNEITLTLVEP----------------   404
Cko1   TAGAKADSAEGWTIIMPAWDSS---------EGATNRWRLSVVVEDEQGQRVSSNEITLSLTEP----------------   437
Sen1   TAGTDTRNTEGWTIIMPAWDNR---------EDATNCWRLSVVVEDEKGQRVSSNEITLALTEP----------------   443
Eca1   TSPANSTSSDGWSIIIPAWDAR---------EGATNRWRLSVVAEDKDGQRVSSNEITLTVVQP----------------   451
Esp2   TSPANANSDEGWSVIMPAWDAS---------EGATNRWKLSVVVEDKDGQRVSSNEITLSLSQP----------------   451
Esa3   TPPLDSTSADGWTVIMPAWDNA---------PGASNSWRLSVTVEDEQGQRVTSNWITLKLSAP----------------   453
Kpn1   TPPVDASSPDGWSIIMPVWNSE---------PGAANRWRLSVVVEDKQGQRVSSNEIALALTEP----------------   445
Pan1   TSPPNNNAIDGWSVIVPPWDST---------PGASNEYRLSVTVEDSKQQRVTSNWITLKVQAP----------------   465
Eta2   TPPPDNSDPQGWSIIIPAWDSQ---------QGASNAWHLSVTLEDSQQQRVTSNWITLKLSPP----------------   464
Spr1   TPSQNSNDPHGWSLIVPQWDDS---------PDAANRYHLSVTLEDDKQQLVTSNWIQLQVTPP----------------   464
Yin1   LATAGATNPQGWQITLPAWDSA---------PGATNLYTLSISIVDEQGRQATSNPVDIKVGQQ----------------   518
Yin2   VPTAGANNPQGWQITLPAWDAT---------PGATNLYTLSISIVDEKGHQATSNDVEIRVGQQ----------------   519
Yfr4   LATAGVDNHQGWQITLPAWNPA---------PAATNRYQLALTVVDERGRQATSNPVDIMVGQQ----------------   516
Ymo2   LATAGSHNPQGWQITLPAWSSV---------AGASNRYQLALSLEDQKNHRVTTNTIEIQVGQR----------------   433
Eco10  ENTLTEGNNNHWNLVLPAWQYSSDQAE----QEKLNTWRVRLALEDEKGNRQNSGVVEITVQQD----------------   484
Eal1   ENTLTEGNNNRWNIVLPAWQYASTEAE----QQKLNTWRVRIALEDEKGNRQNSGVVEITVNQD----------------   476
Sty4   ADALTEGNNNSWNLVLPAWVNADTEAQ----RTALNTWKVRMTLEDEKGNKQNSGVVEITVQQD----------------   377
Eco16  ANALTEGNNNRWNLVLPAWQSASTKEE----QEKVNTWRVRLALEDEKGNRQNSGVVEIVVKQD----------------   457
Eco25  VVTTGK----DILVTLPAYRFTSTPETDN-------TWPIEVTAEDVKGNFSNREQSMVVVQAP----------------   528
Eco15  VSTSGK----DITVTLPGYRFTNTPETDN-------TWSIDVTAEDVKGNLSRHEQSMVVIQAP----------------   486
Eco6   VAVSGK----DIQVTIPPYRFTAMPETDN-------TYPIAVTAEDSKGNFSRREESMVVVEKP----------------   532
Efe2   ISTADN----QVTVTLPAYRYTATPDTDN-------TYRVAVTAEDVKGNRSNREESTVVVQAP----------------   444
Efe3   VNTEAN----QVTVTLPEYRYTATPQTDN-------VYRVAVTAEDEKGNRSNREEASVVVLAP----------------   443
Yfr1   IIPLTM---ESASVTLPPYQQVQ----------TANSYQISAVAYDVRGNRSNTATTTLVVQES----------------   509
Yfr5   LTPLAM---ESAVVTLPAYQPLQ----------NINSYQVSAVAYDIRGNKSNTATTQIIVQES----------------   470
Yps4   TSPLTQ---SSLSVTLPFYQHILR---------TSNTHTISAVAYDAQGNASNRAVTSIEVTRP----------------   546
Yps7   LKQTSK---NGLEITLPEYQMNR-T--------GGNSYILNAIAYDTQGNASSQASMLITVNAQKINIANSTLVAAPVNI   549
Yfr2   LSKGSS---NSISITLPPYQASVGN--------TPNSYTLSAIAFDTQGNRSNSSSTLINVSPQ----------------   500
Yfr3   VTQTSS---QNISIKLPPYTAGS------------NVYMLSAVAYDNQGNTSNHSTTQITVTQQ----------------   459
Yps2   LTPLST---TQFNLVLPPYKRTAQVSRVTD-DLTANFYSLSALAVDHQGNRSNSFTLSVTVQQP----------------   500
Ype5   ITG-SG---NNWQITLPSYLSGGEG---------NNTYRISAIAYDTLGNASPVAYSDLVVDSHG---------------   459
Pru1   IVQE----NGKYSLVLPAYRNDAKSA---------NNYTISAVAIDKKGNISPNTMLRVVVT------------------   525
Pal3   VIDEK---NGAYSIILPTYKSGGKVQ---------NRYTINAVAVDSKGNISPNAILQVQVT------------------   490
Eta1   LVREN---EGSWSVILPEHKPGEAN-----------SWTITGVAVDKKGNVSTGADTQVTVA------------------   551
Sgl1   IVQES---IDNYSIVLPDYNFDSANG---------NVYEISGVAIDTHGNVSKKAKTTLTVT------------------   521
Eco26  IINEG---LHNYSIILPDYRNQENN-----------SYTIDLSAIDIKGHTSNRKTIKIDVL------------------   514
Pmi1   IKHHG---GTHYTVILPQYQIGAIEK---------NSYIISAVAIDTHGNRSAPVQTTVIVD------------------   513
Eco1   IQHSGSQSAQDYQAILPAYVQGGSN-----------VYKVTARAYDRNGNSSNNVQLTITVLSNG---------------   552
Ymo1   ISDQG---NLAYHITFPPYIDGGDN-----------AYVLAGRAVDKKGNYSVSNSTNIYVTG-----------------   468
Yen2   VINQG---NLAYYIAMPPYIDGAVN-----------AYVLSGRAIDKKGNYSVSGSTNVYVTG-----------------   507
Ybe1   IIPQG---PSQFALQLPPQPSGGNN------------WTISAIASDVKGNTSNRAVTLVQLQD-----------------   472
Esa2   SSTNS---VTDANGNTSVNLTNTTA------------GQVIVTATSGSVVRTTSAAFNLLVAN-----------------   444
Sen3   SASTS---NTDAQGNATVSLTSSEI------------MSTTVSAQLDNGQKLDSETLQFTADS-----------------   668
Eco14  IISNT---GNQITLQLPAWSSDRQS------------VTLSGRARDTRGNLSDIARTRILVSP-----------------   425
Bpe1   ALRARNAEGDSSREVTYRYVDEVVLGDLQV-AVMALVPLPADRALEVRGKTEPKVDVKVSFSNG----------------   516
Bav2   RLKAVNQYGDRSREVVYQYEDGLVLGGLEV-KILVMQTLPANGKLKVRGKTEPKMDVRVTFPDG----------------   511
Bbr1   RARATNRHGDRSREVTHHYVDVAVKGEVPL-TLGAVRTHPGTGVVTVTGKTGPGAKVRIDFPDG----------------   494
Bpa2   RARATDRYGDTSQEVTYAYTDTVDKTAPVL-SIAGVATDEATGRVTVTGRSEPDAQVTIRFPGG----------------   507
Bav1   RAQARNVHGDSSPEVSRFYTANPQVEMTAA-PRIERVETLRNGLLRVSGRAAAGADIWVVFPDD----------------   444
Pma1   --------------------------------------------------------------------------------   372
Pma5   --------------------------------------------------------------------------------   373
Pma3   --------------------------------------------------------------------------------   379
Pma4   --------------------------------------------------------------------------------   410
Ssp1   --------------------------------------------------------------------------------   428
Ssp2   --------------------------------------------------------------------------------   436
Eco20  ITGQGS----QWQVTLPAYRPGKDN-----------YYAISAVAYDNKGNASKRVQTEVVITGAG---------------   457
Efe5   ITGSG----TSWQFTLPAYQNGGVN-----------TWTVGATARDSKGNASNHAVITISVTSSN---------------   441
Plu1   INIEST---HGYTIQLPEYQPDGKNDYTITGTSKDDQLRVQIQAHVLQRNISLSVNTTDPLIADG---------------   501
Pal2   ISSDDQ----TATLTLPSYQTSNAAKDVQR----INRYRLRAIAYDIKGNVSPVAETLIEITHSGSISISPRDMKFHGKG   471
Ahy1   LSPADTAGQDKRALTLPSLPTYRIGQ--------SNQYPVVAIVTDIDGHEAIAEGVVAVSEDS----------------   550
Csu1   TLVSAGKSNAEYTVKLPPHKLRLSSQGR---TVPTNEYTLQVFAVDTSGRRSKTGTLSITVSQDPQVSIN----------   407
Plu2   --------------------------------------------------------------------------------   302
Rba1   --------------------------------------------------------------------------------   327
Pas1   IIALSN---NSYVVQFPNYSRQQSN-----------HITISAVAHDEQGNVSNSSEMGVLINVP----------------   453
Cla1   YVQIIRSCTPKTDLNAVAGEANGKFP--------ISIIKLDFKIKADPEKIERFEESLITLAGG----------------   730
ruler  .......970.......980.......990......1000......1010......1020......1030......1040
```

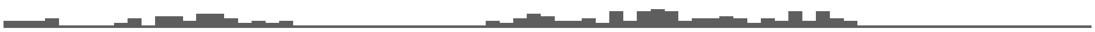

```
   Mba1 --------------------------------------------------------------------------------   331
   Psp2 --------------------------------------------------------------------------------   251
   Sen2 --------------------------------------------------------------------------------   427
   Efe4 --------------------------------------------------------------------------------   445
   Eco3 --------------------------------------------------------------------------------   404
   Cko1 --------------------------------------------------------------------------------   437
   Sen1 --------------------------------------------------------------------------------   443
   Eca1 --------------------------------------------------------------------------------   451
   Esp2 --------------------------------------------------------------------------------   451
   Esa3 --------------------------------------------------------------------------------   453
   Kpn1 --------------------------------------------------------------------------------   445
   Pan1 --------------------------------------------------------------------------------   465
   Eta2 --------------------------------------------------------------------------------   464
   Spr1 --------------------------------------------------------------------------------   464
   Yin1 --------------------------------------------------------------------------------   518
   Yin2 --------------------------------------------------------------------------------   519
   Yfr4 --------------------------------------------------------------------------------   516
   Ymo2 --------------------------------------------------------------------------------   433
  Eco10 --------------------------------------------------------------------------------   484
   Eal1 --------------------------------------------------------------------------------   476
   Sty4 --------------------------------------------------------------------------------   377
  Eco16 --------------------------------------------------------------------------------   457
  Eco25 --------------------------------------------------------------------------------   528
  Eco15 --------------------------------------------------------------------------------   486
   Eco6 --------------------------------------------------------------------------------   532
   Efe2 --------------------------------------------------------------------------------   444
   Efe3 --------------------------------------------------------------------------------   443
   Yfr1 --------------------------------------------------------------------------------   509
   Yfr5 --------------------------------------------------------------------------------   470
   Yps4 --------------------------------------------------------------------------------   546
   Yps7 EANNIDTSVVTLTLKDDNNIPVPGQNVTFLSPLGTLSAMTDSGNGVYTATLTAGTVSGTTAVSSNINGIALDMTPATVTL   629
   Yfr2 --------------------------------------------------------------------------------   500
   Yfr3 --------------------------------------------------------------------------------   459
   Yps2 --------------------------------------------------------------------------------   500
   Ype5 --------------------------------------------------------------------------------   459
   Pru1 --------------------------------------------------------------------------------   525
   Pal3 --------------------------------------------------------------------------------   490
   Eta1 --------------------------------------------------------------------------------   551
   Sgl1 --------------------------------------------------------------------------------   521
  Eco26 --------------------------------------------------------------------------------   514
   Pmi1 --------------------------------------------------------------------------------   513
   Eco1 --------------------------------------------------------------------------------   552
   Ymo1 --------------------------------------------------------------------------------   468
   Yen2 --------------------------------------------------------------------------------   507
   Ybe1 --------------------------------------------------------------------------------   472
   Esa2 --------------------------------------------------------------------------------   444
   Sen3 --------------------------------------------------------------------------------   668
  Eco14 --------------------------------------------------------------------------------   425
   Bpe1 --------------------------------------------------------------------------------   516
   Bav2 --------------------------------------------------------------------------------   511
   Bbr1 --------------------------------------------------------------------------------   494
   Bpa2 --------------------------------------------------------------------------------   507
   Bav1 --------------------------------------------------------------------------------   444
   Pma1 --------------------------------------------------------------------------------   372
   Pma5 --------------------------------------------------------------------------------   373
   Pma3 --------------------------------------------------------------------------------   379
   Pma4 --------------------------------------------------------------------------------   410
   Ssp1 --------------------------------------------------------------------------------   428
   Ssp2 --------------------------------------------------------------------------------   436
  Eco20 --------------------------------------------------------------------------------   457
   Efe5 --------------------------------------------------------------------------------   441
   Plu1 --------------------------------------------------------------------------------   501
   Pal2 LANGNDINGLTAIARDSLG---------------------------------------------------------HAIP   494
   Ahy1 --------------------------------------------------------------------------------   550
   Csu1 --------------------------------------------------------------------------------   407
   Plu2 --------------------------------------------------------------------------------   302
   Rba1 --------------------------------------------------------------------------------   327
   Pas1 --------------------------------------------------------------------------------   453
   Cla1 --------------------------------------------------------------------------------   730
  ruler ......1050......1060......1070......1080......1090......1100......1110......1120
```

```
Mba1   --------------------------------------------------------------------------------   331
Psp2   --------------------------------------------------------------------------------   251
Sen2   ------ETSFRSWINIINDDAQVED-------------------------------------------------------   446
Efe4   ------PESFTPYLNVLHDEVRREE-------------------------------------------------------   464
Eco3   --------FDALSNDEL---------------------------------------------------------------   413
Cko1   --------FMAMPDNDP---------------------------------------------------------------   446
Sen1   --------FITMPDDNP---------------------------------------------------------------   452
Eca1   --------LVVMPDDDP---------------------------------------------------------------   460
Esp2   --------LVALPDDDG---------------------------------------------------------------   460
Esa3   --------VQTLPQDDP---------------------------------------------------------------   462
Kpn1   --------LVKFTTPGV---------------------------------------------------------------   454
Pan1   --------LSMDEPAVEGAVQMPSS-------------------------------------------------------   482
Eta2   --------IGL--PGLD---------------------------------------------------------------   471
Spr1   --------LTVSSEIEQGLPPAKVL-------------------------------------------------------   481
Yin1   --------RSGRLMLESAADVPASG-------------------------------------------------------   535
Yin2   --------RLGRLFIDGDSAMPATG-------------------------------------------------------   536
Yfr4   --------RLGQLVLESAGSVPASG-------------------------------------------------------   533
Ymo2   --------RQGRLQVEGSNAVTASG-------------------------------------------------------   450
Eco10  --------RKIELIVNNIANVPEEN-------------------------------------------------------   501
Eal1   --------RKIELIVNNIANVPEEN-------------------------------------------------------   493
Sty4   --------RKIELIVDNIADN-DRS-------------------------------------------------------   393
Eco16  --------RKIELIVNHVLSVPADN-------------------------------------------------------   474
Eco25  -----TLSQKDSSVSLSSQTLSADS-------------------------------------------------------   548
Eco15  -----TLSQKDSLLSVNPLTVAADK-------------------------------------------------------   506
Eco6   -----TLSLTDSTLSVDQQILLADG-------------------------------------------------------   552
Efe2   -----QLSAEDSEVTSDKPILKPDG-------------------------------------------------------   464
Efe3   -----QLSAQHSQVTSDKTTLKPDG-------------------------------------------------------   463
Yfr1   ------PQQISLTLSGGSNTAVADG-------------------------------------------------------   528
Yfr5   ------PHQIALNVVGSSTSATADG-------------------------------------------------------   489
Yps4   ------ETMVISHLATTVDNATANG-------------------------------------------------------   565
Yps7   NGNSGNLSTTHSTLVAAPVSIEANG-------------------------------------------------------   654
Yfr2   -----NLSTGNSLMTATPSILVADG-------------------------------------------------------   520
Yfr3   -----SVSHLNSTLQVSPTEISADG-------------------------------------------------------   479
Yps2   --------QLTLTAAVIGDGAPANG-------------------------------------------------------   517
Ype5   ------VNTNASGLTAAPEILPANA-------------------------------------------------------   478
Pru1   ---QPAIYPIKSALTPRKIDLIADGKNTKKLSLSIRDKAGNYIDLAANEIGIEKVVANKTAARSVNSANTANLTT-----   597
Pal3   ---QPTIFSANSPLTP-TINLVTNG-------------------------------------------------------   511
Eta1   ---QAAIDASMSPVTPEKITLPADG-------------------------------------------------------   573
Sgl1   ---QPAINTTTSEFTPVKSTLPADT-------------------------------------------------------   543
Eco26  ---YMDIDPTISSLDPKNISLPADG-------------------------------------------------------   536
Pmi1   ---KSLINTRNSLFSPKQSQLFANGEATQKLILSIVDNDNLPVDIDSKEITLQQQSDTEKGNSRISTFSRLAAG------   584
Eco1   ---QVVDQVGVTDFTADKTSAKADG-------------------------------------------------------   574
Ymo1   ----VNINSVKSTITLTPATLPANATSRSTLQLKLNTDAGNAVSGAARQITFAVRDVSGNVPRARSFAQPVVIAD-----   539
Yen2   ----VNINRINSTISLNPATLPANGTSRSTIQLKLNTDAGQAVSGASGQMTFAIRDSSGRVFKARTSLQPVVISD-----   578
Ybe1   -----TPATISGTWTPAASTKPADG-------------------------------------------------------   492
Esa2   ------LDLVVT-----KDNSIADG-------------------------------------------------------   458
Sen3   ------SSATVTGVEVDKKVARADG-------------------------------------------------------   687
Eco14  ------AVQQQLAVSTDKTTATADG-------------------------------------------------------   444
Bpe1   ------ESVLAKADAKGLFTVRSTRKVTQGKVLVQATHPQTRKEARAVADYLPPATLAP---------------------   569
Bav2   ------EVVQLRSADDGSFTALSKQRVPRGVVQVRVTDPSTDQEAVAQDMYEPPAAVAP---------------------   564
Bbr1   ------TFGDVVAGNGGDFTVASKGDVTA---------------------------------------------------   517
Bpa2   ------GRKTVRADANGAYRVRSDGDLPAGVIVVQAADAAGNSTQAQQAYQDTVDRTAPEPVQIALDTDVATGRLTVSGQ   581
Bav1   ------EEVNGKAEADGSFILSSTR-------------------------------------------------------   463
Pma1   --------------------------------------------------------------------------------   372
Pma5   --------------------------------------------------------------------------------   373
Pma3   --------------------------------------------------------------------------------   379
Pma4   --------------------------------------------------------------------------------   410
Ssp1   --------------------------------------------------------------------------------   428
Ssp2   --------------------------------------------------------------------------------   436
Eco20  ---MSADRTALTLDGQSRIQMLANG-------------------------------------------------------   479
Efe5   ---VSAGDSSFTLDGNNNAQISADG-------------------------------------------------------   463
Plu1   ---NAKYVYTATLLGADKKTPIENAKLIWDTD------------------------------------------------   530
Pal2   NTKVVFVLPNALTLASRKTKTTTNSSVQNSLRKKMQTMKVAKPWEYITTTNDKGEALVQFTSEIAGSYEISAYTG-----   569
Ahy1   ------GLQPAIQLAEHFVQLLPG--------------------------------------------------------   568
Csu1   ----RKYFLNGTPVTDFTTPPVAND-------------------------------------------------------   428
Plu2   --------------------------------------------------------------------------------   302
Rba1   --------------------------------------------------------------------------------   327
Pas1   --VALSAPVIGPVEKDEAELLLLTG-------------------------------------------------------   476
Cla1   ---------NLANNLKVVWRLIGKG-------------------------------------------------------   746
ruler  ......1130......1140......1150......1160......1170......1180......1190......1200
```

```
  Mba1 --------------------------------------------------------------------------------  331
  Psp2 --------------------------------------------------------------------------------  251
  Sen2 --------------------------------------------------------------------------------  446
  Efe4 --------------------------------------------------------------------------------  464
  Eco3 --------------------------------------------------------------------------------  413
  Cko1 --------------------------------------------------------------------------------  446
  Sen1 --------------------------------------------------------------------------------  452
  Eca1 --------------------------------------------------------------------------------  460
  Esp2 --------------------------------------------------------------------------------  460
  Esa3 --------------------------------------------------------------------------------  462
  Kpn1 --------------------------------------------------------------------------------  454
  Pan1 --------------------------------------------------------------------------------  482
  Eta2 --------------------------------------------------------------------------------  471
  Spr1 --------------------------------------------------------------------------------  481
  Yin1 --------------------------------------------------------------------------------  535
  Yin2 --------------------------------------------------------------------------------  536
  Yfr4 --------------------------------------------------------------------------------  533
  Ymo2 --------------------------------------------------------------------------------  450
 Eco10 --------------------------------------------------------------------------------  501
  Eal1 --------------------------------------------------------------------------------  493
  Sty4 --------------------------------------------------------------------------------  393
 Eco16 --------------------------------------------------------------------------------  474
 Eco25 --------------------------------------------------------------------------------  548
 Eco15 --------------------------------------------------------------------------------  506
  Eco6 --------------------------------------------------------------------------------  552
  Efe2 --------------------------------------------------------------------------------  464
  Efe3 --------------------------------------------------------------------------------  463
  Yfr1 --------------------------------------------------------------------------------  528
  Yfr5 --------------------------------------------------------------------------------  489
  Yps4 --------------------------------------------------------------------------------  565
  Yps7 --------------------------------------------------------------------------------  654
  Yfr2 --------------------------------------------------------------------------------  520
  Yfr3 --------------------------------------------------------------------------------  479
  Yps2 --------------------------------------------------------------------------------  517
  Ype5 --------------------------------------------------------------------------------  478
  Pru1 ------------------------------------------------------------------------VSGFTRIA  605
  Pal3 --------------------------------------------------------------------------------  511
  Eta1 --------------------------------------------------------------------------------  573
  Sgl1 --------------------------------------------------------------------------------  543
 Eco26 --------------------------------------------------------------------------------  536
  Pmi1 --------------------------------------------------------------------------------  584
  Eco1 --------------------------------------------------------------------------------  574
  Ymo1 ------------------------------------------------------------------------AQEIQTGV  547
  Yen2 ------------------------------------------------------------------------VQEVQTGV  586
  Ybe1 --------------------------------------------------------------------------------  492
  Esa2 --------------------------------------------------------------------------------  458
  Sen3 --------------------------------------------------------------------------------  687
 Eco14 --------------------------------------------------------------------------------  444
  Bpe1 ----------------------------------------------------------------------TIDAVTAQAD  579
  Bav2 ----------------------------------------------------------------------TIDKVTTDPA  574
  Bbr1 --------------------------------------------------------------------------------  517
  Bpa2 AEPGGSVTVTWPDGTTQTVRLDADGGFRVTSAGDAAQGDVVVVVADQAGNRAAPVRAHYADPVDRTAPMTPTVRHATDAQ  661
  Bav1 --------------------------------------------------------------------------------  463
  Pma1 --------------------------------------------------------------------------------  372
  Pma5 --------------------------------------------------------------------------------  373
  Pma3 --------------------------------------------------------------------------------  379
  Pma4 --------------------------------------------------------------------------------  410
  Ssp1 --------------------------------------------------------------------------------  428
  Ssp2 --------------------------------------------------------------------------------  436
 Eco20 --------------------------------------------------------------------------------  479
  Efe5 --------------------------------------------------------------------------------  463
  Plu1 --------------------------------------------------------------------------------  530
  Pal2 --------------------------------------------------------------------------------  569
  Ahy1 --------------------------------------------------------------------------------  568
  Csu1 --------------------------------------------------------------------------------  428
  Plu2 --------------------------------------------------------------------------------  302
  Rba1 --------------------------------------------------------------------------------  327
  Pas1 --------------------------------------------------------------------------------  476
  Cla1 --------------------------------------------------------------------------------  746
 ruler ......1210......1220......1230......1240......1250......1260......1270......1280
```

```
Mba1   --------------------------------------------------------------------------------   331
Psp2   --------------------------------------------------------------------------------   251
Sen2   -------------------------------------------GNFVISTPLPAGEEGKVIEWHYVRERSEEEWASLKPR   483
Efe4   -------------------------------------------GKFVIPSPTVNDDNGSIIEWHYVRVRSKDEWKSLKPE   501
Eco3   -----------------------------------------RWEP-----------------------------------   417
Cko1   -----------------------------------------RWKLLPEE-------------------------------   454
Sen1   -----------------------------------------PWQPFQEQ-------------------------------   460
Eca1   -----------------------------------------RWKLLPDD-------------------------------   468
Esp2   -----------------------------------------GYQLLPNE-------------------------------   468
Esa3   -----------------------------------------RYELLAPVP------------------------------   471
Kpn1   -----------------------------------------SWTDSP---------------------------------   460
Pan1   -----------------------------------------SNSLSFSE-------------------------------   490
Eta2   -----------------------------------------SESLNFTDS------------------------------   480
Spr1   -----------------------------------------QPPTIPAETGPLNGSD-----------------------   497
Yin1   -----------------------------------------RDSDAIKVAGYLVNPADIGVTDSRLVPHWLLTDKATGAA   574
Yin2   -----------------------------------------LDADAVKLSAHLEDHLGKSINDAALAPVWVARSLYSGAV   575
Yfr4   -----------------------------------------LATDAIVLAAHLEDHLGQLINDPQLQPVWQVIDATSGTP   572
Ymo2   -----------------------------------------HDSDVIRLVSYLEDHNGLAINDHDTKPLWLVKSLAR-TP   488
Eco10  -----------------------------------------NHSHEASAQADGVDGVVMDLDVTDSFGDNTDRNGDALPE   540
Eal1   -----------------------------------------NHSHEASAQADGVDGVVMDLDVTDSFGDDTDGKGNVLPE   532
Sty4   -----------------------------------------DHSHEASALADGEDGVVMDLLITDSFGDSTDRNGNELVD   432
Eco16  -----------------------------------------NHSYQAEAKADGSDGVVMDMHITDSFGDNTDKNGNTLPE   513
Eco25  -------------------------------------------HSTATLTFIAHDAAGNPVIGLVLST---RHEGVQDIT   582
Eco15  -------------------------------------------KSTTTLTVTAHDSDGTPVPGLALQT---RSEGVQDIT   540
Eco6   -------------------------------------------KSTSTLTYTARDSSGKPIPGMTLKT---QVKGLQDFA   586
Efe2   -------------------------------------------VDTAHLTFLARDTDGKAVSGLKVTAGFTAPEGLNFSL   501
Efe3   -------------------------------------------TEKAVLTFRAQDAEGKAVSALTVSTSFTAPQGMALVL   500
Yfr1   -------------------------------------------NAPVSYIATVVDTSGT---------------------   544
Yfr5   -------------------------------------------SALVTYRASVVDTANG---------------------   505
Yps4   -------------------------------------------IAANTVQATVTDGDG----------------------   580
Yps7   -------------------------------------------SDTSLVTLTLRDSNN----------------------   669
Yfr2   -------------------------------------------VSTSQITITLRDSHN----------------------   535
Yfr3   -------------------------------------------VAVSLITLNLKDDNN----------------------   494
Yps2   -------------------------------------------KTAITVEFTVADFEG----------------------   532
Ype5   -------------------------------------------SASSVIEFNIKDNANQPITGIADELAFSLE-------   508
Pru1   AGQYEAILTSGTTPENFVLVSKARNAVFPEIKVSVLADGATTQIAKLMTNISQSQPADGKTPLEIKTQLKDANGNPLINT   685
Pal3   --------------------------------------------TPAFTGIKDNKPAS--------KLLKMAD--PYIHN   537
Eta1   -------------------------------------------KTQQLLLLKINDRDG--------KPVDIAE-------   595
Sgl1   -------------------------------------------VAQQTLTLKVKDIQG--------NPVDIGE-------   565
Eco26  -------------------------------------------QSQKKLTLKIKNRNG--------LFEDINP-------   558
Pmi1   -----------------KYQLTVTAGSIPEKLTLTPVFRDNTFNSATVTLIADNQTAHIAKGNLTVTKDNEPADGKSQNK   647
Eco1   --------------------------------------------TEAITYTATVKKNGVAQAN-----------------   593
Ymo1   YETSVTSGNLVGRFEITPTVRGVQLNPIILTQSADATTATINGSANITISTPTITANATDKTHLEVLVTDALGHPVPGVE   627
Yen2   YEASITSGFLTGRFEITPTVRGVQLNPIILTQSADKTTATITDSSAVTISTPSITTNATDKTKLEVQVTDALGHPVPGVE   666
Ybe1   -------------------------------------------NSAVLLTLTLKDAAGNPLIG-----------------   512
Esa2   -------------------------------------------TDQNAAQVQVKDASGKGLSG-----------------   478
Sen3   -------------------------------------------QDTITVQATVMDAEQHPLAN-----------------   707
Eco14  -------------------------------------------ADSVRYTLTVTGSDGKPVSG-----------------   464
Bpe1   TGRVTVTGQAEPGAQVDVHFPDGTAKTVDAGADGAYAATSDGDMVSGDIHAQATDKAGNQSPEATRHYGDTTDITPPAAP   659
Bav2   TGRVTIVGEAEPRSRVEIRFSDGTTEVVDADDEGHYSATSAGDLPSGELRATRLPAAGEQPLSTRREYKDEVDKTAPEAP   654
Bbr1   ---------------------------------------------SGPIVAIARDDDGRESPRRTVQYDDRVNGGGSGAP   552
Bpa2   TGRVTVMGRTEAGALVTVQFPDGSSKTVRAQNDGGYAATSDTDMVSGPIVVSAGDADGNRTPAQQVMYTDTLDKTAPASP   741
Bav1   ------------------------------------------PHASGDILVTASLPDGGVSDASTYAYVKQP----PGEP   497
Pma1   --------------------------------------------------------------------------------   372
Pma5   --------------------------------------------------------------------------------   373
Pma3   --------------------------------------------------------------------------------   379
Pma4   --------------------------------------------------------------------------------   410
Ssp1   --------------------------------------------------------------------------------   428
Ssp2   --------------------------------------------------------------------------------   436
Eco20  -------------------------------------------NEQRPLVLSLRDAEGQPVTGMKDQIKTELAFKPAGNI   516
Efe5   -------------------------------------------QSTYPVTLNLKDSNGKPLTG---------------LE   485
Plu1   ---------------------------------------KKDPGLKLKPDSEATDKNGQQTATLTSTTPLSDIQVSVRIN   571
Pal2   -----------------------------NHQPAKAQVVFKPDSAQASIHSFTITRSGVLADGISTNSVKAHITDKNKNP   620
Ahy1   ------------------------------------AHYQVDWGVVDPRQKPAKARNGIPIESIEGDVEADRDGYQYRYR   612
Csu1   -------------------------------------------QDFVEIEFTVTGMYGPLIGKEVLVRYAQGSEEKYEVL   465
Plu2   --------------------------------------------------------------------------------   302
Rba1   --------------------------------------------------------------------------------   327
Pas1   --------------------------------------------EGGEVPPFLNDREDELLEKQLGQKRLEQERLEQERL   512
Cla1   -------------------------------------------ELIKTNTSDTTDNNGEAIIKYKADDTMKDKEEVKVIA   783
ruler  ......1290......1300......1310......1320......1330......1340......1350......1360
```

```
Mba1 --------------------------------------------------------------------------------  331
Psp2 --------------------------------------------------------------------------------  251
Sen2 NIKYQSDTPGLSFKALGGTERDGHWVERVLVTHVGDDARS----FKLHIEASG---------------------------  532
Efe4 NVEYSTHSPGLSFKSLGGEERDGQWIEKVQLVVKDPTARMTLAAMELNISATG---------------------------  554
Eco3 --------------------------------------------------------------------------------  417
Cko1 --------------------------------------------------------------------------------  454
Sen1 --------------------------------------------------------------------------------  460
Eca1 --------------------------------------------------------------------------------  468
Esp2 --------------------------------------------------------------------------------  468
Esa3 --------------------------------------------------------------------------------  471
Kpn1 --------------------------------------------------------------------------------  460
Pan1 --------------------------------------------------------------------------------  490
Eta2 --------------------------------------------------------------------------------  480
Spr1 --------------------------------------------------------------------------------  497
Yin1 VPLVT-GSACP-LDASGNPEPCIRLKNTQVEVRDGVNYYVLELVSTVMGTFSLQADFNHY--------------------  632
Yin2 VPLVT-GPTCP-TDEDGFPDACLRVVHTATEVRDGITYYVNSLISNQPGTFIITTDLGAY--------------------  633
Yfr4 VAWVAPGSSCP-VDALGLPQPCVQMVQVQTEVRSGINHYVAELVSTQLGTFLVQADLGVY--------------------  631
Ymo2 IELVN-ANQCP-LNAAGKVEPCLRIKDDRTEVRDGVNYHILELVSTLAGNFTISSDMGVY--------------------  546
Eco10 DNLTPQLYDAQDKRVTLTNKPCSTDNPCVFIAKQDKEKGTVTLSSTLPGTYRWKAKAAPY--------------------  600
Eal1 DNLNPQLYDAQDKKVTLANKPCTTEVPCVFIAEKNKEKGTVTLASTLPGTFRWKAKAAPY--------------------  592
Sty4 DAMTPVLYDSNDKKVTLAQTPCTTETPCVFIASRDKEAGTVTLSSTLPGTFRWKAKEDAY--------------------  492
Eco16 TGVTPTLYDENDKKVTLNTKPCVTETPCVFIAKRDKEKGTITLSSTLPGTFRWKVKAEPY--------------------  573
Eco25 LSDWKDNGDGSYTQILTTGAMSGTLTLMPQLNGVDAAKAPAVVNIISVSSSRTHSSIKID--------------------  642
Eco15 LSDWTDNGDGSYTQILTAGTTSGSVTLTPQINGESAVKESIVVNIVPVVSSRDHSSITID--------------------  600
Eco6 LSEWKDNGNGTYTQIVTAGKTSGALSLMPQFNGDDIAKTPALIAIVANTASRADSTIETD--------------------  646
Efe2 SEFFTETKTPGEYTAGLKGSMKGTVSVMPLVDGKPATKAPVTVTLSDVVPVAKYSTITLSSAATRADQ------------  569
Efe3 SDTFTETETKGTYTAELKGTMPGEVRVMPQVAGKDAAKDAVTVTLVNTTPVSEHSSITLSPAVSRADR------------  568
Yfr1 -------VATPLAGMNIAFDS-TVGNVLTPNAVTDNQGKANITIKST---------------------------------  583
Yfr5 -------ANTPMAGMNVAFNA-TIGDVVTPVGTTDNMGRATVSVKST---------------------------------  544
Yps4 ---------QPIIGQIINFAVNTQATLSTTEARTGANGIASTTLTHT---------------------------------  618
Yps7 ---------NPVTGQTVALVSTLGTLGAVTEQASGVYTATLTAGTVA---------------------------------  707
Yfr2 ---------QPVIGASRNIILAS---------------------------------------------------------  549
Yfr3 ---------LPVKGMGDQLTLDL---------------------------------------------------------  508
Yps2 ---------KPLAGQEVVITTNNGALPNKITEKTDANGVARIALTNT---------------------------------  570
Ype5 -LVELPEELAKAKARSVPLKTVSHTLTKITESAPGIYQATLTSGSKP---------------------------------  554
Pru1 RVTWSSDKNPNKVSFSSNTTSTDQNGFASTTVTSTLAGDVLITASAGLGESQDLTVTFIPDIKSAMIRPQNFHASQSHTL  765
Pal3 --------NHHKI-------------------------------------------------------------------  542
Eta1 ----------SEISVLREAKLRTAGSTTITAFSRSAAGEYVATLTAG---------------------------------  632
Sgl1 ----------DEITVTSNNAQENSG-AKVSALQRQDSGIYTLVVTAG---------------------------------  601
Eco26 ----------DDINVLKTSEENFDVSSKITRFSRQEAGIYTATLTAG---------------------------------  595
Pmi1 IKVRVTDSNHNPLAHYPVNFTASNGANVISSRKTDEQGEIIASVTNTQVGSSQIGVQVKGITYSTEVHFSVDNDSAYIPQ  727
Eco1 ----------VPVSFNIVSGTATLGANSAKTDANGKATVTLKSSTPG---------------------------------  630
Ymo1 VTWVSDLNSPGLEHVTSITNEHGIAENNFTSTTAGTANITVQTGTSPPVQAGTIEIKPD---------------------  686
Yen2 VTWVSDLNSPGLEYVTSITNEHGIAENNFSSTVTGTANITVQVGTSAPVQAGKIEIKADNSTMTVNASDFTVTTTPVVAN  746
Ybe1 --------QANNITLKKNTLGGSGSEPTLSALTETGPGIYQTTATAG---------------------------------  551
Esa2 --------VAISWKVGNGAT---IVSSDKATNSSGVATIHLSSTTPG---------------------------------  514
Sen3 --------QPVDWAIRSAASSTHLSDKQSNTNENGVATITLTSAKAG---------------------------------  746
Eco14 --------QAVRWEHNGGTLN-----GENTTNADGVATATLTSQTAG---------------------------------  498
Bpe1 TIANVATDATSGRVTAAGMAEPGANVTVNFPDGTRKTVVAGGDGAYTATSD-----------------------------  710
Bav2 SITKVSTDATTGRVTVSGSAEPDVVVTVMFPDGTEKTVPTNDDGTYRATSD-----------------------------  705
Bbr1 TVV-LHTDGTNGRVTVSGKGRPGDTIRVDFPDGTTKEVVAGPDGTYRVTSD-----------------------------  602
Bpa2 SLT-VREDAASGRATVTGQAEPGAAVRVVFPNGEAQTVTAGSDGAYSVTSA-----------------------------  791
Bav1 TVDAVSADLVG-RLTIQGSSEAGAAIQANFPDGTDANTTADAQGRYTLHSPG----------------------------  548
Pma1 --------------------------------------------------------------------------------  372
Pma5 --------------------------------------------------------------------------------  373
Pma3 --------------------------------------------------------------------------------  379
Pma4 --------------------------------------------------------------------------------  410
Ssp1 --------------------------------------------------------------------------------  428
Ssp2 --------------------------------------------------------------------------------  436
Eco20 VTRSLKATKSQAKPTLGEFTETEAGVYQSVFTTGTQSGEATITVSVDGMSKTVTAELRAT--------------------  576
Efe5 NDIEMSLEFTPDNNTQRSRSVSGPQLGKVQEISAGVYRSMLTAGSQS---------------------------------  532
Plu1 GERVTTDKKVSFTESSSSYRVTGVTVGVDKDKKRYNNGADSYTFTATVVDGHGKPVADKLIDIDWQTDSKVDG-------  644
Pal2 LANQEVTFSATHAKIVDKATTNENGIVEVTLTSLKAGSSEVTISINGQSETKSVQFLSGKLHQVTILDVPEVYAGRESQV  700
Ahy1 LTFQGPGQVLTGRELVAPGQPNPGTRYRLDVEVIFPSGHVARDSMEFEFIDDAT--------------------------  666
Csu1 LTDSQGKIRTSHIKTTLASPVEVDIAVNTVVDAVSVPFGPDTGTARVSGTINILKNNQPAN-------------------  526
Plu2 --------------------------------------------------------------------------------  302
Rba1 --------------------------------------------------------------------------------  327
Pas1 ERERLEQERLGQERLEQERLEQERLGRERLEQERLGQEWLEQERLEQE--------------------------------  560
Cla1 SVNGVEFKAPVQIMVFGNNDNDLSIDKEVIGNKEIAIATYKNLRPNKTNVKWR---------------------------  836
ruler ......1370......1380......1390......1400......1410......1420......1430......1440
```

```
Mba1   --------------------------------------------------------------------------------  331
Psp2   --------------------------------------------------------------------------------  251
Sen2   --------------------------------------------------------------------------------  532
Efe4   --------------------------------------------------------------------------------  554
Eco3   --------------------------------------------------------------------------------  417
Cko1   --------------------------------------------------------------------------------  454
Sen1   --------------------------------------------------------------------------------  460
Eca1   --------------------------------------------------------------------------------  468
Esp2   --------------------------------------------------------------------------------  468
Esa3   --------------------------------------------------------------------------------  471
Kpn1   --------------------------------------------------------------------------------  460
Pan1   --------------------------------------------------------------------------------  490
Eta2   --------------------------------------------------------------------------------  480
Spr1   --------------------------------------------------------------------------------  497
Yin1   --------------------------------------------------------------------------------  632
Yin2   --------------------------------------------------------------------------------  633
Yfr4   --------------------------------------------------------------------------------  631
Ymo2   --------------------------------------------------------------------------------  546
Eco10  --------------------------------------------------------------------------------  600
Eal1   --------------------------------------------------------------------------------  592
Sty4   --------------------------------------------------------------------------------  492
Eco16  --------------------------------------------------------------------------------  573
Eco25  --------------------------------------------------------------------------------  642
Eco15  --------------------------------------------------------------------------------  600
Eco6   --------------------------------------------------------------------------------  646
Efe2   --------------------------------------------------------------------------------  569
Efe3   --------------------------------------------------------------------------------  568
Yfr1   --------------------------------------------------------------------------------  583
Yfr5   --------------------------------------------------------------------------------  544
Yps4   --------------------------------------------------------------------------------  618
Yps7   --------------------------------------------------------------------------------  707
Yfr2   --------------------------------------------------------------------------------  549
Yfr3   --------------------------------------------------------------------------------  508
Yps2   --------------------------------------------------------------------------------  570
Ype5   --------------------------------------------------------------------------------  554
Pru1   ADGITPNTLTVTVTDASGNIIPNVEVQFATDKGQLTNDKVVTNHQGISETQITSTQSGSATITASVGNHTLNKTLLFSAN  845
Pal3   --------------------------------------------------------------------------------  542
Eta1   --------------------------------------------------------------------------------  632
Sgl1   --------------------------------------------------------------------------------  601
Eco26  --------------------------------------------------------------------------------  595
Pmi1   QNFTITPPLSLADGKTEKTISLQVVDKQNNPIPATHVTLSADNQAQLKQTILTTDEQGKATTTMISKVAGTVTVRAKIND  807
Eco1   --------------------------------------------------------------------------------  630
Ymo1   --------------------------------------------------------------------------------  686
Yen2   GTSKAVYKLKVMDKQGNVVPGAAVDWLSNIGTFVQGSTTTTDTNGETFIELVSTKAETAKVTATVGGKPYNAGKVVFVAD  826
Ybe1   --------------------------------------------------------------------------------  551
Esa2   --------------------------------------------------------------------------------  514
Sen3   --------------------------------------------------------------------------------  746
Eco14  --------------------------------------------------------------------------------  498
Bpe1   --------------------------------------------------------------------------------  710
Bav2   --------------------------------------------------------------------------------  705
Bbr1   --------------------------------------------------------------------------------  602
Bpa2   --------------------------------------------------------------------------------  791
Bav1   --------------------------------------------------------------------------------  548
Pma1   --------------------------------------------------------------------------------  372
Pma5   --------------------------------------------------------------------------------  373
Pma3   --------------------------------------------------------------------------------  379
Pma4   --------------------------------------------------------------------------------  410
Ssp1   --------------------------------------------------------------------------------  428
Ssp2   --------------------------------------------------------------------------------  436
Eco20  --------------------------------------------------------------------------------  576
Efe5   --------------------------------------------------------------------------------  532
Plu1   --------------------------------------------------------------------------------  644
Pal2   SFQLLDSHG-----------------------------------------------------------------------  709
Ahy1   --------------------------------------------------------------------------------  666
Csu1   --------------------------------------------------------------------------------  526
Plu2   --------------------------------------------------------------------------------  302
Rba1   --------------------------------------------------------------------------------  327
Pas1   --------------------------------------------------------------------------------  560
Cla1   --------------------------------------------------------------------------------  836
ruler  ......1450......1460......1470......1480......1490......1500......1510......1520
```

```
  Mba1 --------------------------------------------------------------------------------  331
  Psp2 --------------------------------------------------------------------------------  251
  Sen2 --------------------------------------------------------------------------------  532
  Efe4 --------------------------------------------------------------------------------  554
  Eco3 --------------------------------------------------------------------------------  417
  Cko1 --------------------------------------------------------------------------------  454
  Sen1 --------------------------------------------------------------------------------  460
  Eca1 --------------------------------------------------------------------------------  468
  Esp2 --------------------------------------------------------------------------------  468
  Esa3 --------------------------------------------------------------------------------  471
  Kpn1 --------------------------------------------------------------------------------  460
  Pan1 --------------------------------------------------------------------------------  490
  Eta2 --------------------------------------------------------------------------------  480
  Spr1 --------------------------------------------------------------------------------  497
  Yin1 --------------------------------------------------------------------------------  632
  Yin2 --------------------------------------------------------------------------------  633
  Yfr4 --------------------------------------------------------------------------------  631
  Ymo2 --------------------------------------------------------------------------------  546
 Eco10 --------------------------------------------------------------------------------  600
  Eal1 --------------------------------------------------------------------------------  592
  Sty4 --------------------------------------------------------------------------------  492
 Eco16 --------------------------------------------------------------------------------  573
 Eco25 --------------------------------------------------------------------------------  642
 Eco15 --------------------------------------------------------------------------------  600
  Eco6 --------------------------------------------------------------------------------  646
  Efe2 --------------------------------------------------------------------------------  569
  Efe3 --------------------------------------------------------------------------------  568
  Yfr1 --------------------------------------------------------------------------------  583
  Yfr5 --------------------------------------------------------------------------------  544
  Yps4 --------------------------------------------------------------------------------  618
  Yps7 --------------------------------------------------------------------------------  707
  Yfr2 --------------------------------------------------------------------------------  549
  Yfr3 --------------------------------------------------------------------------------  508
  Yps2 --------------------------------------------------------------------------------  570
  Ype5 --------------------------------------------------------------------------------  554
  Pru1 GQTAKVDYVLPEPKASYIADGRTAVTYTAKVLDTNNNPVSDVDVNWLSNLNGSELHFEKETSKTNAQGIATTSIISNKAG  925
  Pal3 --------------------------------------------------------------------------------  542
  Eta1 --------------------------------------------------------------------------------  632
  Sgl1 --------------------------------------------------------------------------------  601
 Eco26 --------------------------------------------------------------------------------  595
  Pmi1 KMTHATTQFIANQAKGVIVSITPSSTTHVADGQTPVFLTALVQDQFGKPLPHAQISWETEHDKSIVNIEHTTMTNEQGIT  887
  Eco1 --------------------------------------------------------------------------------  630
  Ymo1 --------------------------------------------------------------------------------  686
  Yen2 RQSGKITLLPVSKNTAAANGTDSITLNAKILDANGNPIKNEEIEWDAASHKVTFSPATGKTQTNDLGETQITLTSTDVGD  906
  Ybe1 --------------------------------------------------------------------------------  551
  Esa2 --------------------------------------------------------------------------------  514
  Sen3 --------------------------------------------------------------------------------  746
 Eco14 --------------------------------------------------------------------------------  498
  Bpe1 --------------------------------------------------------------------------------  710
  Bav2 --------------------------------------------------------------------------------  705
  Bbr1 --------------------------------------------------------------------------------  602
  Bpa2 --------------------------------------------------------------------------------  791
  Bav1 --------------------------------------------------------------------------------  548
  Pma1 --------------------------------------------------------------------------------  372
  Pma5 --------------------------------------------------------------------------------  373
  Pma3 --------------------------------------------------------------------------------  379
  Pma4 --------------------------------------------------------------------------------  410
  Ssp1 --------------------------------------------------------------------------------  428
  Ssp2 --------------------------------------------------------------------------------  436
 Eco20 --------------------------------------------------------------------------------  576
  Efe5 --------------------------------------------------------------------------------  532
  Plu1 --------------------------------------------------------------------------------  644
  Pal2 --------------------------------------------------------------------------------  709
  Ahy1 --------------------------------------------------------------------------------  666
  Csu1 --------------------------------------------------------------------------------  526
  Plu2 --------------------------------------------------------------------------------  302
  Rba1 --------------------------------------------------------------------------------  327
  Pas1 --------------------------------------------------------------------------------  560
  Cla1 --------------------------------------------------------------------------------  836
 ruler ......1530......1540......1550......1560......1570......1580......1590......1600
```

```
  Mba1 --------------------------------------------------------------------------------   331
  Psp2 --------------------------------------------------------------------------------   251
  Sen2 --------------------------------------------------------------------------------   532
  Efe4 --------------------------------------------------------------------------------   554
  Eco3 --------------------------------------------------------------------------------   417
  Cko1 --------------------------------------------------------------------------------   454
  Sen1 --------------------------------------------------------------------------------   460
  Eca1 --------------------------------------------------------------------------------   468
  Esp2 --------------------------------------------------------------------------------   468
  Esa3 --------------------------------------------------------------------------------   471
  Kpn1 --------------------------------------------------------------------------------   460
  Pan1 --------------------------------------------------------------------------------   490
  Eta2 --------------------------------------------------------------------------------   480
  Spr1 --------------------------------------------------------------------------------   497
  Yin1 --------------------------------------------------------------------------------   632
  Yin2 --------------------------------------------------------------------------------   633
  Yfr4 --------------------------------------------------------------------------------   631
  Ymo2 --------------------------------------------------------------------------------   546
 Eco10 --------------------------------------------------------------------------------   600
  Eal1 --------------------------------------------------------------------------------   592
  Sty4 --------------------------------------------------------------------------------   492
 Eco16 --------------------------------------------------------------------------------   573
 Eco25 --------------------------------------------------------------------------------   642
 Eco15 --------------------------------------------------------------------------------   600
  Eco6 --------------------------------------------------------------------------------   646
  Efe2 --------------------------------------------------------------------------------   569
  Efe3 --------------------------------------------------------------------------------   568
  Yfr1 --------------------------------------------------------------------------------   583
  Yfr5 --------------------------------------------------------------------------------   544
  Yps4 --------------------------------------------------------------------------------   618
  Yps7 --------------------------------------------------------------------------------   707
  Yfr2 --------------------------------------------------------------------------------   549
  Yfr3 --------------------------------------------------------------------------------   508
  Yps2 --------------------------------------------------------------------------------   570
  Ype5 --------------------------------------------------------------------------------   554
  Pru1 IVIVTASTNGNGQLAVPITFVADKNQAKVATLAINKATIIANGNDKALLDVIVTDNFGNPVEGIDVL------LQANNGA   999
  Pal3 --------------------------------------------------------------------------------   542
  Eta1 --------------------------------------------------------------------------------   632
  Sgl1 --------------------------------------------------------------------------------   601
 Eco26 --------------------------------------------------------------------------------   595
  Pmi1 TNKLTSTQALSVRVTARINNNAFTAEPITFIANGQQALISELLVNKNQIVADKQDPAELTAIVTDKLGNQLPDTIVNWQG   967
  Eco1 --------------------------------------------------------------------------------   630
  Ymo1 --------------------------------------------------------------------------------   686
  Yen2 ITLNAQVVKNNLLVNQAGEKLSFTADTVTANISAWSAPSVKTLIADGQAQVIYKVVVKDKNGHVVPNSPVLWETNLGEFV   986
  Ybe1 --------------------------------------------------------------------------------   551
  Esa2 --------------------------------------------------------------------------------   514
  Sen3 --------------------------------------------------------------------------------   746
 Eco14 --------------------------------------------------------------------------------   498
  Bpe1 --------------------------------------------------------------------------------   710
  Bav2 --------------------------------------------------------------------------------   705
  Bbr1 --------------------------------------------------------------------------------   602
  Bpa2 --------------------------------------------------------------------------------   791
  Bav1 --------------------------------------------------------------------------------   548
  Pma1 --------------------------------------------------------------------------------   372
  Pma5 --------------------------------------------------------------------------------   373
  Pma3 --------------------------------------------------------------------------------   379
  Pma4 --------------------------------------------------------------------------------   410
  Ssp1 --------------------------------------------------------------------------------   428
  Ssp2 --------------------------------------------------------------------------------   436
 Eco20 --------------------------------------------------------------------------------   576
  Efe5 --------------------------------------------------------------------------------   532
  Plu1 --------------------------------------------------------------------------------   644
  Pal2 --------------------------------------------------------------------------------   709
  Ahy1 --------------------------------------------------------------------------------   666
  Csu1 --------------------------------------------------------------------------------   526
  Plu2 --------------------------------------------------------------------------------   302
  Rba1 --------------------------------------------------------------------------------   327
  Pas1 --------------------------------------------------------------------------------   560
  Cla1 --------------------------------------------------------------------------------   836
 ruler ......1610......1620......1630......1640......1650......1660......1670......1680
```

```
  Mba1 -------------------------------------------------------------------------------- 331
  Psp2 -------------------------------------------------------------------------------- 251
  Sen2 ------------------------------------------------------PDDKHPVKGSVLLQAQSDS------- 551
  Efe4 ------------------------------------------------------PGGTHPVNGTIRMTPVMN-------- 572
  Eco3 -------------------------------------------------------------------------------- 417
  Cko1 -------------------------------------------------------------------------------- 454
  Sen1 -------------------------------------------------------------------------------- 460
  Eca1 -------------------------------------------------------------------------------- 468
  Esp2 -------------------------------------------------------------------------------- 468
  Esa3 -------------------------------------------------------------------------------- 471
  Kpn1 -------------------------------------------------------------------------------- 460
  Pan1 -------------------------------------------------------------------------------- 490
  Eta2 -------------------------------------------------------------------------------- 480
  Spr1 -------------------------------------------------------------------------------- 497
  Yin1 -------------------------------------------------------GRSNVQIVTFSNPGGRD-NVARAE- 655
  Yin2 -------------------------------------------------------GVTNAKTITFTSASPMETVVARAE- 657
  Yfr4 -------------------------------------------------------GVTAPQTVSFTSSGPTL--VTRAE- 653
  Ymo2 -------------------------------------------------------GVSNSQTFVFNSTISSIENLTGGI- 570
 Eco10 -------------------------------------------------------DDSNYVDVTFLG------AEIGGL- 618
  Eal1 -------------------------------------------------------DDSNYVDVTFLG------SDIGGL- 610
  Sty4 -------------------------------------------------------GDSNYVDVTFIG------DNLSAL- 510
 Eco16 -------------------------------------------------------SDSNFVDVTFTNT-----ANVGGI- 592
 Eco25 -------------------------------------------------KDRYLSGNPIEVTVELRDENDKPVKEQKQQL 673
 Eco15 -------------------------------------------------NVSYYAGDDIKVRVELKDDSNQPVAYQKEEL 631
  Eco6 -------------------------------------------------QDNYVAGKPIVVKVTLRDDNGNGVTGRKELL 677
  Efe2 ------------------------------------------------AGQNFHAGEQVFVTVMLKDTLQHPVSDQKALL 601
  Efe3 ------------------------------------------------AGQAFRAGDAVTATVILRDEQQRPVIHQAALL 600
  Yfr1 ------------------------------------------------------LAGGGHIHGVLDNGNRAQAP------ 603
  Yfr5 ------------------------------------------------------LAGAGQVHAVLDNGNRAQAA------ 564
  Yps4 ------------------------------------------------------VAGVSAVSATLGSSSRSVN------- 637
  Yps7 ------------------------------------------------------GVASLSVSVGGSALGVAPAT------ 727
  Yfr2 -------------------------------------------------------------------------------- 549
  Yfr3 -------------------------------------------------------------------------------- 508
  Yps2 ------------------------------------------------------TDGVTVVTAEVEGQRQSVDT------ 590
  Ype5 ------------------------------------------------------QLINITAQINGVPLADVQTK------ 574
  Pru1 TITTLTPFSKTTVDGRVKAELATNQATGDIMVAANVVGSQSQPVAKHIKALADNNTAKVTIVSSTDRVQISQQTPTVILT 1079
  Pal3 -------------------------------------------------------------------------------- 542
  Eta1 -----------------------------------------------------TLPESFTLIPTARNVRFAP-------- 651
  Sgl1 -----------------------------------------------------TGTDVIKITPSARGANFAS-------- 620
 Eco26 -----------------------------------------------------TKSERFTITPMIYNIKLPPSTVTISKD 622
  Pmi1 SAGTQFAQQQTKTDAQGIAKNTLTTSQSGITTITARLNNGQHAQTNLVAVADLKSATLTLTTLNNKQSAIADNQDSITVV 1047
  Eco1 -----------------------------------------------------------QVVVSAKTAEMTSALN----- 646
  Ymo1 -------------------------------------------------------------------------------- 686
  Yen2 PAQATTTMTSTDSQGEATVVLASIKAGSATVKASVNANKDTSPTQVEFTADSSTATIAITPVTKQVYVANGSEKVTYAVT 1066
  Ybe1 -----------------------------------------------------TNFGVLTLTPEIQGTQLAP-------- 570
  Esa2 ---------------------------------------------------------AIKLSASAGN------------- 524
  Sen3 ---------------------------------------------------------QGIVTASTGNSEPV--------- 760
 Eco14 ---------------------------------------------------------IIRVTATTRN------------- 508
  Bpe1 ---------------------------------------------------RDMVSGDIRVQATDKAGNQSPEATRAYAD 739
  Bav2 ---------------------------------------------------GNMVSGDILAHATDRAKNRSPDTRYAYAD 734
  Bbr1 ---------------------------------------------------RDMTAGDITVSGTDAKGNVGGPVKRPYHD 631
  Bpa2 ---------------------------------------------------ADMVAGEITVVAADASGNQSAPARTVFAD 820
  Bav1 ---------------------------------------------------AVLQSGDIMITASGIDGAVGDAVFKPYTP 577
  Pma1 -------------------------------------------------------------------------------- 372
  Pma5 -------------------------------------------------------------------------------- 373
  Pma3 -------------------------------------------------------------------------------- 379
  Pma4 -------------------------------------------------------------------------------- 410
  Ssp1 -------------------------------------------------------------------------------- 428
  Ssp2 -------------------------------------------------------------------------------- 436
 Eco20 ----------------------------------------------------MMDVANSTLSANEPSGDVVADGQQAYTL 604
  Efe5 --------------------------------------------------------GTARVTAKVLGKTFTLN------- 549
  Plu1 --------------------------------------------LKLTKQNNSVSNAQGQVTATLTSTAAVENVQVSAKT 680
  Pal2 --------------NPIIDAQNDITTIIDKKTESTAIWDTDIDKGIYAAKISGQQPGTHTIQVVMGKTVSQEQKFNTLGT 775
  Ahy1 ------------------------------------------------------VPGAPTLTAADSNGDDKPEVTGKAEP 692
  Csu1 ------------------------------------------------------NQDQAEVIFTIVDAHNNPIPN----- 547
  Plu2 -------------------------------------------------------------------------------- 302
  Rba1 -------------------------------------------------------------------------------- 327
  Pas1 ------------------------------------------------------RLERERLEQERLEQERLE-------- 578
  Cla1 ------------------------------------------------------LDGEDKALFILENENKSSTAK----- 857
 ruler ......1690......1700......1710......1720......1730......1740......1750......1760
```

```
   Mba1 --------------------------------------------------------------------------------  331
   Psp2 --------------------------------------------------------------------------------  251
   Sen2 --------------------------------------------------------------------------------  551
   Efe4 --------------------------------------------------------------------------------  572
   Eco3 --------------------------------------------------------------------------------  417
   Cko1 --------------------------------------------------------------------------------  454
   Sen1 --------------------------------------------------------------------------------  460
   Eca1 --------------------------------------------------------------------------------  468
   Esp2 --------------------------------------------------------------------------------  468
   Esa3 --------------------------------------------------------------------------------  471
   Kpn1 --------------------------------------------------------------------------------  460
   Pan1 --------------------------------------------------------------------------------  490
   Eta2 --------------------------------------------------------------------------------  480
   Spr1 --------------------------------------------------------------------------------  497
   Yin1 --------------------------------------------------------------------------------  655
   Yin2 --------------------------------------------------------------------------------  657
   Yfr4 --------------------------------------------------------------------------------  653
   Ymo2 --------------------------------------------------------------------------------  570
  Eco10 --------------------------------------------------------------------------------  618
   Eal1 --------------------------------------------------------------------------------  610
   Sty4 --------------------------------------------------------------------------------  510
  Eco16 --------------------------------------------------------------------------------  592
  Eco25 N-------------------------------------------------------------------------------  674
  Eco15 V-------------------------------------------------------------------------------  632
   Eco6 K-------------------------------------------------------------------------------  678
   Efe2 QG------------------------------------------------------------------------------  603
   Efe3 TE------------------------------------------------------------------------------  602
   Yfr1 --------------------------------------------------------------------------------  603
   Yfr5 --------------------------------------------------------------------------------  564
   Yps4 --------------------------------------------------------------------------------  637
   Yps7 --------------------------------------------------------------------------------  727
   Yfr2 --------------------------------------------------------------------------------  549
   Yfr3 --------------------------------------------------------------------------------  508
   Yps2 --------------------------------------------------------------------------------  590
   Ype5 --------------------------------------------------------------------------------  574
   Pru1 ATVIDDQQNPLIGTPVTWLTNHNRLSTNTTVTDLRGQAKVELSGYISGETQVTAQLMNRQTAHQNIQFMADIPHQQNSVL 1159
   Pal3 --------------------------------------------------------------------------------  542
   Eta1 --------------------------------------------------------------------------------  651
   Sgl1 --------------------------------------------------------------------------------  620
  Eco26 RQVFLSLSPSVITSG-----------------------------------------------------------------  637
   Pmi1 ANLTDAYHNPLKNIPVYWQSSINHIDETTTQTDEKGRTQVVISGTKAQPTTITAYLSNKEKKSIQVTFVAGAPVQQNSYL 1127
   Eco1 --------------------------------------------------------------------------------  646
   Ymo1 --------------------------------------------------------------------------------  686
   Yen2 VLDANNNPVKAEAINWKSENGHPVKVEPAPSQTDGQGKATVSIGSVKAGDTQIRATLGNNATAIADAITFEADRQTAVVK 1146
   Ybe1 --------------------------------------------------------------------------------  570
   Esa2 --------------------------------------------------------------------------------  524
   Sen3 --------------------------------------------------------------------------------  760
  Eco14 --------------------------------------------------------------------------------  508
   Bpe1 AVDR----------------------------------------------------------------------------  743
   Bav2 AV------------------------------------------------------------------------------  736
   Bbr1 IF------------------------------------------------------------------------------  633
   Bpa2 AVDR----------------------------------------------------------------------------  824
   Bav1 EAP-----------------------------------------------------------------------------  580
   Pma1 --------------------------------------------------------------------------------  372
   Pma5 --------------------------------------------------------------------------------  373
   Pma3 --------------------------------------------------------------------------------  379
   Pma4 --------------------------------------------------------------------------------  410
   Ssp1 --------------------------------------------------------------------------------  428
   Ssp2 --------------------------------------------------------------------------------  436
  Eco20 TLTAVD--------------------------------------------------------------------------  610
   Efe5 --------------------------------------------------------------------------------  549
   Plu1 AS------------------------------------------------------------------------------  682
   Pal2 NAVASVAADGSGPLG-----------------------------------------------------------------  790
   Ahy1 ES------------------------------------------------------------------------------  694
   Csu1 --------------------------------------------------------------------------------  547
   Plu2 --------------------------------------------------------------------------------  302
   Rba1 --------------------------------------------------------------------------------  327
   Pas1 --------------------------------------------------------------------------------  578
   Cla1 --------------------------------------------------------------------------------  857
  ruler ......1770......1780......1790......1800......1810......1820......1830......1840
```

```
   Mba1 --------------------------------------------------------------------------------   331
   Psp2 --------------------------------------------------------------------------------   251
   Sen2 --------------------------------------------------------------------------------   551
   Efe4 --------------------------------------------------------------------------------   572
   Eco3 --------------------------------------------------------------------------------   417
   Cko1 --------------------------------------------------------------------------------   454
   Sen1 --------------------------------------------------------------------------------   460
   Eca1 --------------------------------------------------------------------------------   468
   Esp2 --------------------------------------------------------------------------------   468
   Esa3 --------------------------------------------------------------------------------   471
   Kpn1 --------------------------------------------------------------------------------   460
   Pan1 --------------------------------------------------------------------------------   490
   Eta2 --------------------------------------------------------------------------------   480
   Spr1 --------------------------------------------------------------------------------   497
   Yin1 --------------------------------------------------------------------------------   655
   Yin2 --------------------------------------------------------------------------------   657
   Yfr4 --------------------------------------------------------------------------------   653
   Ymo2 --------------------------------------------------------------------------------   570
  Eco10 --------------------------------------------------------------------------------   618
   Eal1 --------------------------------------------------------------------------------   610
   Sty4 --------------------------------------------------------------------------------   510
  Eco16 --------------------------------------------------------------------------------   592
  Eco25 --------------------------------------------------------------------------------   674
  Eco15 --------------------------------------------------------------------------------   632
   Eco6 --------------------------------------------------------------------------------   678
   Efe2 --------------------------------------------------------------------------------   603
   Efe3 --------------------------------------------------------------------------------   602
   Yfr1 --------------------------------------------------------------------------------   603
   Yfr5 --------------------------------------------------------------------------------   564
   Yps4 --------------------------------------------------------------------------------   637
   Yps7 --------------------------------------------------------------------------------   727
   Yfr2 --------------------------------------------------------------------------------   549
   Yfr3 --------------------------------------------------------------------------------   508
   Yps2 --------------------------------------------------------------------------------   590
   Ype5 --------------------------------------------------------------------------------   574
   Pru1 EIKPQTIVANNHEQATATLILRDQWNNPVLGQNIQWSKNNTGLSLSGARELPNN--------------------------  1213
   Pal3 --------------------------------------------------------------------------------   542
   Eta1 --------------------------------------------------------------------------------   651
   Sgl1 --------------------------------------------------------------------------------   620
  Eco26 --------------------------------------------------------------------------------   637
   Pmi1 TIEPQSIIADGNAFAVGKIDLRDKFDNPVIGRSNDIALIGDNSTIQFSKITETAN-------------------------  1182
   Eco1 --------------------------------------------------------------------------------   646
   Ymo1 --------------------------------------------------------------------------------   686
   Yen2 TVEVTGSKVTAPDGTGSISYVTTVVDANGNPVSGMILSWGSNINNVANPSTTTDINGQSSQTITGTQAGKVEISVALTSG  1226
   Ybe1 --------------------------------------------------------------------------------   570
   Esa2 --------------------------------------------------------------------------------   524
   Sen3 --------------------------------------------------------------------------------   760
  Eco14 --------------------------------------------------------------------------------   508
   Bpe1 --------------------------------------------------------------------------------   743
   Bav2 --------------------------------------------------------------------------------   736
   Bbr1 --------------------------------------------------------------------------------   633
   Bpa2 --------------------------------------------------------------------------------   824
   Bav1 --------------------------------------------------------------------------------   580
   Pma1 --------------------------------------------------------------------------------   372
   Pma5 --------------------------------------------------------------------------------   373
   Pma3 --------------------------------------------------------------------------------   379
   Pma4 --------------------------------------------------------------------------------   410
   Ssp1 --------------------------------------------------------------------------------   428
   Ssp2 --------------------------------------------------------------------------------   436
  Eco20 --------------------------------------------------------------------------------   610
   Efe5 --------------------------------------------------------------------------------   549
   Plu1 --------------------------------------------------------------------------------   682
   Pal2 --------------------------------------------------------------------------------   790
   Ahy1 --------------------------------------------------------------------------------   694
   Csu1 --------------------------------------------------------------------------------   547
   Plu2 --------------------------------------------------------------------------------   302
   Rba1 --------------------------------------------------------------------------------   327
   Pas1 --------------------------------------------------------------------------------   578
   Cla1 --------------------------------------------------------------------------------   857
  ruler ......1850......1860......1870......1880......1890......1900......1910......1920
```

```
  Mba1 --------------------------------------------------------------------------------   331
  Psp2 --------------------------------------------------------------------------------   251
  Sen2 --------------------------------------------------------------------------------   551
  Efe4 --------------------------------------------------------------------------------   572
  Eco3 --------------------------------------------------------------------------------   417
  Cko1 --------------------------------------------------------------------------------   454
  Sen1 --------------------------------------------------------------------------------   460
  Eca1 --------------------------------------------------------------------------------   468
  Esp2 --------------------------------------------------------------------------------   468
  Esa3 --------------------------------------------------------------------------------   471
  Kpn1 --------------------------------------------------------------------------------   460
  Pan1 --------------------------------------------------------------------------------   490
  Eta2 --------------------------------------------------------------------------------   480
  Spr1 --------------------------------------------------------------------------------   497
  Yin1 --------------------------------------------------------------------------------   655
  Yin2 --------------------------------------------------------------------------------   657
  Yfr4 --------------------------------------------------------------------------------   653
  Ymo2 --------------------------------------------------------------------------------   570
 Eco10 --------------------------------------------------------------------------------   618
  Eal1 --------------------------------------------------------------------------------   610
  Sty4 --------------------------------------------------------------------------------   510
 Eco16 --------------------------------------------------------------------------------   592
 Eco25 --------------------------------------------------------------------------------   674
 Eco15 --------------------------------------------------------------------------------   632
  Eco6 --------------------------------------------------------------------------------   678
  Efe2 --------------------------------------------------------------------------------   603
  Efe3 --------------------------------------------------------------------------------   602
  Yfr1 --------------------------------------------------------------------------------   603
  Yfr5 --------------------------------------------------------------------------------   564
  Yps4 --------------------------------------------------------------------------------   637
  Yps7 --------------------------------------------------------------------------------   727
  Yfr2 --------------------------------------------------------------------------------   549
  Yfr3 --------------------------------------------------------------------------------   508
  Yps2 --------------------------------------------------------------------------------   590
  Ype5 --------------------------------------------------------------------------------   574
  Pru1 --------------------------------------------------------------------------------  1213
  Pal3 --------------------------------------------------------------------------------   542
  Eta1 --------------------------------------------------------------------------------   651
  Sgl1 --------------------------------------------------------------------------------   620
 Eco26 --------------------------------------------------------------------------------   637
  Pmi1 --------------------------------------------------------------------------------  1182
  Eco1 --------------------------------------------------------------------------------   646
  Ymo1 --------------------------------------------------------------------------------   686
  Yen2 NNATNPVKNSNNAEFVAVTPVMANADLLLQPNLIIANGKQTATLKFTLRDANHNPVSGLKQRIDVTQSVASHVTIGAVTE  1306
  Ybe1 --------------------------------------------------------------------------------   570
  Esa2 --------------------------------------------------------------------------------   524
  Sen3 --------------------------------------------------------------------------------   760
 Eco14 --------------------------------------------------------------------------------   508
  Bpe1 --------------------------------------------------------------------------------   743
  Bav2 --------------------------------------------------------------------------------   736
  Bbr1 --------------------------------------------------------------------------------   633
  Bpa2 --------------------------------------------------------------------------------   824
  Bav1 --------------------------------------------------------------------------------   580
  Pma1 --------------------------------------------------------------------------------   372
  Pma5 --------------------------------------------------------------------------------   373
  Pma3 --------------------------------------------------------------------------------   379
  Pma4 --------------------------------------------------------------------------------   410
  Ssp1 --------------------------------------------------------------------------------   428
  Ssp2 --------------------------------------------------------------------------------   436
 Eco20 --------------------------------------------------------------------------------   610
  Efe5 --------------------------------------------------------------------------------   549
  Plu1 --------------------------------------------------------------------------------   682
  Pal2 --------------------------------------------------------------------------------   790
  Ahy1 --------------------------------------------------------------------------------   694
  Csu1 --------------------------------------------------------------------------------   547
  Plu2 --------------------------------------------------------------------------------   302
  Rba1 --------------------------------------------------------------------------------   327
  Pas1 --------------------------------------------------------------------------------   578
  Cla1 --------------------------------------------------------------------------------   857
 ruler ......1930......1940......1950......1960......1970......1980......1990......2000
```

```
Mba1   --------------------------------------------------------------------------------   331
Psp2   --------------------------------------------------------------------------------   251
Sen2   --------------------------------------------------------------------------------   551
Efe4   --------------------------------------------------------------------------------   572
Eco3   --------------------------------------------------------------------------------   417
Cko1   --------------------------------------------------------------------------------   454
Sen1   --------------------------------------------------------------------------------   460
Eca1   --------------------------------------------------------------------------------   468
Esp2   --------------------------------------------------------------------------------   468
Esa3   --------------------------------------------------------------------------------   471
Kpn1   --------------------------------------------------------------------------------   460
Pan1   --------------------------------------------------------------------------------   490
Eta2   --------------------------------------------------------------------------------   480
Spr1   --------------------------------------------------------------------------------   497
Yin1   --------------------------------------------------------------------------------   655
Yin2   --------------------------------------------------------------------------------   657
Yfr4   --------------------------------------------------------------------------------   653
Ymo2   --------------------------------------------------------------------------------   570
Eco10  --------------------------------------------------------------------------------   618
Eal1   --------------------------------------------------------------------------------   610
Sty4   --------------------------------------------------------------------------------   510
Eco16  --------------------------------------------------------------------------------   592
Eco25  --------------------------------------------------------------------------------   674
Eco15  --------------------------------------------------------------------------------   632
Eco6   --------------------------------------------------------------------------------   678
Efe2   --------------------------------------------------------------------------------   603
Efe3   --------------------------------------------------------------------------------   602
Yfr1   --------------------------------------------------------------------------------   603
Yfr5   --------------------------------------------------------------------------------   564
Yps4   --------------------------------------------------------------------------------   637
Yps7   --------------------------------------------------------------------------------   727
Yfr2   --------------------------------------------------------------------------------   549
Yfr3   --------------------------------------------------------------------------------   508
Yps2   --------------------------------------------------------------------------------   590
Ype5   --------------------------------------------------------------------------------   574
Pru1   ----GEYQVNISGNLAGTFDISAQTGSVTSQKMIGLIADSSTAHLKNINIVGKTTAPADGTNPITLRATVTDATNNPAPA  1289
Pal3   --------------------------------------------------------------------------------   542
Eta1   --------------------------------------------------------------------------------   651
Sgl1   --------------------------------------------------------------------------------   620
Eco26  --------------------------------------------------------------------------------   637
Pmi1   ----GNYQAHIRGTKAGLSQITATIDSITVTQSLGFLTDNKTVKIHSVKVMAPYTVTANGKDKVIIQAQITDKHNNPVSK  1258
Eco1   --------------------------------------------------------------------------------   646
Ymo1   --------------------------------------------------------------------------------   686
Yen2   TTVKGVYQAAITGMKENSVDLTASVKGTNVRQTRTLTLQADNKTATLKTVTSNIKTAKADGKESITYRAKVIDAQGNASL  1386
Ybe1   --------------------------------------------------------------------------------   570
Esa2   --------------------------------------------------------------------------------   524
Sen3   --------------------------------------------------------------------------------   760
Eco14  --------------------------------------------------------------------------------   508
Bpe1   --------------------------------------------------------------------------------   743
Bav2   --------------------------------------------------------------------------------   736
Bbr1   --------------------------------------------------------------------------------   633
Bpa2   --------------------------------------------------------------------------------   824
Bav1   --------------------------------------------------------------------------------   580
Pma1   --------------------------------------------------------------------------------   372
Pma5   --------------------------------------------------------------------------------   373
Pma3   --------------------------------------------------------------------------------   379
Pma4   --------------------------------------------------------------------------------   410
Ssp1   --------------------------------------------------------------------------------   428
Ssp2   --------------------------------------------------------------------------------   436
Eco20  --------------------------------------------------------------------------------   610
Efe5   --------------------------------------------------------------------------------   549
Plu1   --------------------------------------------------------------------------------   682
Pal2   --------------------------------------------------------------------------------   790
Ahy1   --------------------------------------------------------------------------------   694
Csu1   --------------------------------------------------------------------------------   547
Plu2   --------------------------------------------------------------------------------   302
Rba1   --------------------------------------------------------------------------------   327
Pas1   --------------------------------------------------------------------------------   578
Cla1   --------------------------------------------------------------------------------   857
ruler  ......2010......2020......2030......2040......2050......2060......2070......2080
```

```
  Mba1 --------------------------------------------------------------------------------  331
  Psp2 --------------------------------------------------------------------------------  251
  Sen2 --------------------------------------------------------------------------------  551
  Efe4 --------------------------------------------------------------------------------  572
  Eco3 --------------------------------------------------------------------------------  417
  Cko1 --------------------------------------------------------------------------------  454
  Sen1 --------------------------------------------------------------------------------  460
  Eca1 --------------------------------------------------------------------------------  468
  Esp2 --------------------------------------------------------------------------------  468
  Esa3 --------------------------------------------------------------------------------  471
  Kpn1 --------------------------------------------------------------------------------  460
  Pan1 --------------------------------------------------------------------------------  490
  Eta2 --------------------------------------------------------------------------------  480
  Spr1 --------------------------------------------------------------------------------  497
  Yin1 --------------------------------------------------------------------------------  655
  Yin2 --------------------------------------------------------------------------------  657
  Yfr4 --------------------------------------------------------------------------------  653
  Ymo2 --------------------------------------------------------------------------------  570
 Eco10 --------------------------------------------------------------------------------  618
  Eal1 --------------------------------------------------------------------------------  610
  Sty4 --------------------------------------------------------------------------------  510
 Eco16 --------------------------------------------------------------------------------  592
 Eco25 --------------------------------------------------------------------------------  674
 Eco15 --------------------------------------------------------------------------------  632
  Eco6 --------------------------------------------------------------------------------  678
  Efe2 --------------------------------------------------------------------------------  603
  Efe3 --------------------------------------------------------------------------------  602
  Yfr1 --------------------------------------------------------------------------------  603
  Yfr5 --------------------------------------------------------------------------------  564
  Yps4 --------------------------------------------------------------------------------  637
  Yps7 --------------------------------------------------------------------------------  727
  Yfr2 --------------------------------------------------------------------------------  549
  Yfr3 --------------------------------------------------------------------------------  508
  Yps2 --------------------------------------------------------------------------------  590
  Ype5 --------------------------------------------------------------------------------  574
  Pru1 GIAVGWRSDIGELSQPVSLTDNNGIAQITLTSTVAGKGKVAAIVGQSTKE-TTNQIEFLAGTVSRSASSVTLAVPSIIAA 1368
  Pal3 --------------------------------------------------------------------------------  542
  Eta1 --------------------------------------------------------------------------------  651
  Sgl1 --------------------------------------------------------------------------------  620
 Eco26 --------------------------------------------------------------------------------  637
  Pmi1 GVTVGWISDLGKLAAPLSMTNKQGIAEITLSSTQAGKAKVTAVLNNQQSVNADHIVTFTEGDISASLSTVAIFPDTIVAG 1338
  Eco1 --------------------------------------------------------------------------------  646
  Ymo1 --------------------------------------------------------------------------------  686
  Yen2 DNVSVGWRTTLGELAAITKTDTSGIATVTLTSKQAGSATVTAIVSSTSEMKAAPVNFTAGGISITQSTASLSVKDLVADD 1466
  Ybe1 --------------------------------------------------------------------------------  570
  Esa2 --------------------------------------------------------------------------------  524
  Sen3 --------------------------------------------------------------------------------  760
 Eco14 --------------------------------------------------------------------------------  508
  Bpe1 --------------------------------------------------------------------------------  743
  Bav2 --------------------------------------------------------------------------------  736
  Bbr1 --------------------------------------------------------------------------------  633
  Bpa2 --------------------------------------------------------------------------------  824
  Bav1 --------------------------------------------------------------------------------  580
  Pma1 --------------------------------------------------------------------------------  372
  Pma5 --------------------------------------------------------------------------------  373
  Pma3 --------------------------------------------------------------------------------  379
  Pma4 --------------------------------------------------------------------------------  410
  Ssp1 --------------------------------------------------------------------------------  428
  Ssp2 --------------------------------------------------------------------------------  436
 Eco20 --------------------------------------------------------------------------------  610
  Efe5 --------------------------------------------------------------------------------  549
  Plu1 --------------------------------------------------------------------------------  682
  Pal2 --------------------------------------------------------------TLGVIADIEIDINPNRTD  808
  Ahy1 --------------------------------------------------------------------------------  694
  Csu1 --------------------------------------------------------------------------------  547
  Plu2 --------------------------------------------------------------------------------  302
  Rba1 --------------------------------------------------------------------------------  327
  Pas1 --------------------------------------------------------------------------------  578
  Cla1 --------------------------------------------------------------------------------  857
 ruler ......2090......2100......2110......2120......2130......2140......2150......2160
```

```
Mba1   --------------------------------------------------------------------------------   331
Psp2   --------------------------------------------------------------------------------   251
Sen2   ---------------------------------------------------------------------------IAQKV   556
Efe4   ---------------------------------------------------------------------------LTDKV   577
Eco3   --------------------------------------------------------------------------------   417
Cko1   --------------------------------------------------------------------------------   454
Sen1   --------------------------------------------------------------------------------   460
Eca1   --------------------------------------------------------------------------------   468
Esp2   --------------------------------------------------------------------------------   468
Esa3   --------------------------------------------------------------------------------   471
Kpn1   --------------------------------------------------------------------------------   460
Pan1   --------------------------------------------------------------------------------   490
Eta2   --------------------------------------------------------------------------------   480
Spr1   --------------------------------------------------------------------------------   497
Yin1   ------------------------------------------------------------------IVD-------SAGT   662
Yin2   ------------------------------------------------------------------IRD-------PAGE   664
Yfr4   ------------------------------------------------------------------IQD-------PSGV   660
Ymo2   ------------------------------------------------------------------FLAKDNPTAGTGAT   584
Eco10  ------------------------------------------------------------------NAFIYRVGAAKPSN   632
Eal1   ------------------------------------------------------------------NAFIYCTNDPKPHN   624
Sty4   ------------------------------------------------------------------NAVIYQVKAANPVN   524
Eco16  ------------------------------------------------------------------NGFIYRLEESSPKN   606
Eco25  ----------------------TAVSIDNVKPGVTTDWKETADGVYKATYTAYTKGSGLTAKLLMQNWNEDLHTAGFIID   732
Eco15  ----------------------KAVTVENSKPGATIVWHEEQPGVYAANYPAYKQGTALRAQLSLHNWNAPLQSHIYNIE   690
Eco6   ----------------------QTVKVDNTKADDVSAWTEESEGIYKASYTAHLIGDKLTAQLTMPGWQT-KHSDAFSIA   735
Efe2   ----------------------EVVTVEGMSSKDGAEWQEEEDGTYKMLYVAQGAKEGHRATLKLTDGNK--TTAPYTIH   659
Efe3   ----------------------ESVTVSGMEPAAGAAWQEEDGGIYRMQYIAKNARDAHKATLRLADGSK--STEPYAIH   658
Yfr1   --------------------------------------------------------------------------LIFIAD   609
Yfr5   --------------------------------------------------------------------------VMFMAD   570
Yps4   --------------------------------------------------------------------------TTFVAD   643
Yps7   --------------------------------------------------------------------------VTLNGD   733
Yfr2   --------------------------------------------------------------------------------   549
Yfr3   --------------------------------------------------------------------------------   508
Yps2   --------------------------------------------------------------------------HFVKG-   595
Ype5   --------------------------------------------------------------------------VTLIAD   580
Pru1   TGETNITITLKDNTGNPLTGLANKIILDYSANLSIATPRFNEISKGVYRGKLSGVKAGSTLIKVKANNVTLDNSVSLTIT  1448
Pal3   --------------------------------------------------------------------------------   542
Eta1   --------------------------------------------------------------------------ISVELT   657
Sgl1   --------------------------------------------------------------------------ASVTVE   626
Eco26  VDSATLRVTIKNSAGNIYDGFQDKIKLQYDTDLIATNTAFREIAGGVYETFIQAKKAGTTTITVLIDDNPVPDSKLLTVK   717
Pmi1   ANKATVTITLKDKEGNLLPNLANKITLTPQTNLQTTITPFKEKQKGIYQAQVSALKTGKTSLSAHVAPLAIKQSVSLTVL  1418
Eco1   --------------------------------------------------------------------------ASAVIF   652
Ymo1   --------------------------------------------------------------------------------   686
Yen2   VITTKLTVNIKDDNGNPLTGKGSEISVTATGLAGLKLPTTFVEGPNGVYTATITGTKAGVGDIVTALAGKELAKQQLKVI  1546
Ybe1   -------------------------------------------------------------------------ATITYTD   577
Esa2   ------------------------------------------------------------------------KTDSVNST   532
Sen3   ------------------------------------------------------------------------ESETLTFM   768
Eco14  -------------------------------------------------------------------------QTAKAAD   515
Bpe1   ------------------------------------------------------------------TAPEVPTVTHVATD   757
Bav2   -------------------------------------------------------------------APATPVIRRLTTN   749
Bbr1   -------------------------------------------------------------------VPVPPTV-EVATD   645
Bpa2   ------------------------------------------------------------------TAPAAPTL-ALSEA   837
Bav1   ----------------------------------------------------------------------QATITSVIP-   589
Pma1   --------------------------------------------------------------------------------   372
Pma5   --------------------------------------------------------------------------------   373
Pma3   --------------------------------------------------------------------------------   379
Pma4   --------------------------------------------------------------------------------   410
Ssp1   --------------------------------------------------------------------------------   428
Ssp2   --------------------------------------------------------------------------------   436
Eco20  --------------------------------------------------------------------SEGNPVTGEASR   622
Efe5   ---------------------------------------------------------------------------IKQTV   554
Plu1   ---------------------------------------------------------------QQTAVNANGKVSFTEFS   699
Pal2   FKSGDNPLVMVTLKDSFGNEIENIDPSNIHLGDFKGNDIAWRQDGKYDYIVNLPLTKTGDIDITAHVNGIFSPKTVLTVS   888
Ahy1   -------------------------------------------------------------------------TVTITWP   701
Csu1   ----------------------------------------------------------------------FAVTATASNQ   557
Plu2   --------------------------------------------------------------------------------   302
Rba1   --------------------------------------------------------------------------------   327
Pas1   --------------------------------------------------------------------------RERLDQ   584
Cla1   ------------------------------------------------------------------------ELQLIADN   865
ruler  ......2170......2180......2190......2200......2210......2220......2230......2240
```

```
Mba1   --------------------------------------------------------------------------------    331
Psp2   --------------------------------------------------------------------------------    251
Sen2   TSVEVLFTPGNDEANGSVTAPVVGTEMRARTLCINNTDCTDAFNYQWEISDEMKSWKSVPGATKATWLLPYSLNGESLQN    636
Efe4   SSVQILFTAGTEELNGSTNAPVVGSTLQAKTTCDTDKDCSSLFRYQWEISPDGNRWYDVPGATGQSWLMPAVMDGHSLQN    657
Eco3   --------------------------------------------------------------------------------    417
Cko1   --------------------------------------------------------------------------------    454
Sen1   --------------------------------------------------------------------------------    460
Eca1   --------------------------------------------------------------------------------    468
Esp2   --------------------------------------------------------------------------------    468
Esa3   --------------------------------------------------------------------------------    471
Kpn1   --------------------------------------------------------------------------------    460
Pan1   --------------------------------------------------------------------------------    490
Eta2   --------------------------------------------------------------------------------    480
Spr1   --------------------------------------------------------------------------------    497
Yin1   DVLVSGNHPQVGVTYTVKLYNA-------ANVDITSTLPADSLHWALDGANTAG----CAITLNNFDTG-ATGYSFTPRP    730
Yin2   DLLTTHNAPQVGVTYTVVLFDE-------NDVDVTETLPADEVHWALDGTNTAG----CAITLDNHDTG-MTGYTFTPRP    732
Yfr4   DLLTSGAHPQVGVVYTVKLFDA-------ANTDITATIPATEVHWALDGTNTAG----CAITLNNHDTG-VTGYQFTPRT    728
Ymo2   DYAATGTPLKVGETYRFIAWSDRQGNGKWSEGDEEVTSSLQSIQWYLDGTNSSATGGSSGITLVDHEIAGATTDHYTVAV    664
Eco10  LIGKDKEPLPLNNTYRFVLWRDNNKDGVFQQVEKLTDEEMVQYDYKWEFTGKSINGEVGAQANTSNEDIVIPATNREAAQ    712
Eal1   LAGVD-ERIPTDSTYRFVLWRDKNKDGIFQQSEKLTDEEMAQYDYQWEFTGQSAHGETGAQANTTNEDIVIPATNQEAAQ    703
Sty4   LIGKEDKHPTVNNTYRFLLWRDKNKDGVFQMSEQLTEEEMALYDYQWEFTGQSTNGHTGALANTINEDLVLPVTNKEAAQ    604
Eco16  LAG-DAKPIPLKSGYRFVMWRDSNNDGIYQQSEKLTEEEKARYDYEWEFTGKSAHGSTGAKANTINEDLILPATNKEAAS    685
Eco25  ANPQSAKIATLSASNNGVLANENAANTVSVNVADEGSNPINDHTVTFAVLSGSATSFNNQN--TAKTDVNGLATFDLKSS    810
Eco15  ANQNKARVATLSATNNDVYADKKTFNTLTINVTDESDNPLTNHQVTFKNEKGSA-EFVEPP--QQNTDAYGVATINMVSQ    767
Eco6   GDKDTAKIAAMQITANNAVARR-DHNTVAVTVRDVHQNLLQGQNVTFTVVNGAA-VFADPNGGIVTTDKDGIASVNLASD    813
Efe2   PGDVNIDKSTLGSDKEEIAADGQEEAIITYNAQDSYGNAIDNLDVKAAIDMPVGMTIALQDFVKGAEKGVYTATLKGTSQ    739
Efe3   PGEANPTKSLLGTDKPAILANGDDTATLEFIARDAYDNIVSDLTIDAVAETSQKMDITLSKHFSQEKPGTYTAVLHGTTP    738
Yfr1   SSTAEIVSGNLTVTTDNATANGSDSNAVQVLVTDANGNPVSNEMVSFSVDNGN-LANPNANTGPDGIATMTLTN-TAAGT    687
Yfr5   ESTASILSDNLTVLSNNALANGTATDSVQVMVTDANGNPVPNQLVNFNADNGATLSFSSGITDANGTIIVTAVN-TTTGV    649
Yps4   ESTAEITAANLTVTTNDSVANGSDTNAVRAKVTDAYTNAVANQSVIFSASNGATVIDQTVITNAEGIADSTLTN-TTAGV    722
Yps7   SGNLSTTHSTLVAAPVNIEANGSDTSLVTLTLRDSNNNPVTGQTVALVSTLGT---LGAVTEQASGVYTATLTAGTVAGV    810
Yfr2   ----------------NFVVDGS-----------------TSPIRAAAASEMK---ISDVSEAMGGVYNATLTAGTQPGT    593
Yfr3   ----------------HFTPDTT---------------------ATQPVSYPL---LSTVTETAPGIYTYQLTAGFTQGE    548
Yps2   --TIAADKSTLAAVPTSIIADGLMASTITLELKDTYGDPQAGANVAFDTTLGN---MGVITDHNDGTYSAPLTS-TTLGV    669
Ype5   ESTATLQTSSLQIITNGSLADDTDANQIRAVVVDAYGNKLSGVQVNFTVGNNAKITETTLSDKQGGVTAAITST--KAGT    658
Pru1   PDSQTARVRGGITASKTTETVGRSVTYSAIFEDANSNLLGAGVPVFWVGNDNTLLSDNQTMTDTTGRSAIQVQRDTIGEA   1528
Pal3   --------------------------------------------------------------------------------    542
Eta1   ANDNTIVVKDAISATPTSAVAGDTVTYSAVLTDKQGNPQGAGIPVDWIANEGSTLGAQTSSSDETGTVAVTLTRAQPGTA    737
Sgl1   ADGTTAQIKALEVVSDNAAADG----------------------------------------QATNQVKVTVI-------    659
Eco26  ADNNSATVKGSISANPGVALVGQYTTYKATLVDKNDNIIDAGTTVIWSADTGTVLNTNVVTTDKAGSVSVQATRSQPGIA    797
Pmi1   PNNTTAKVKN-FTISNIQPHAGESITYKAYLVDNHDNPVGMGVPVAWSTNEGSQLETPLTFTDDNGVAIVGLSRRSVGVA   1497
Eco1   VDQTKASITEIKADKTTAVANGKDAVTYTVKVMKNG--LPEKGHVVTFSTDLGKLNLQTVATDKDGFASVTLTSDSVGKA    730
Ymo1   --------------------------------------------------------------------------------    686
Yen2   ADVQTAKIADIKPLKSGSVSVGDKVTYQATLKDANDNLLGAGIPVHWSVNRDTLMSGKLISLTNSAGVAEVEISRDLAGD   1626
Ybe1   PSSTKPSVSNLILLGELAEDSKLSATYTFTQPAG----------------------------------------------    611
Esa2   FVSQTVAAVAVTMTTNNSPADGSTANVAQALVTSASGQPMPGVSVTWNVGTATATTPLN-VTTNASGIATVSLTDTTAET    611
Sen3   PDVKSATLSAITADKTAAQANGADAVTLSVKVEDANHNPIPGAQISWTTTSATAILSASDTSTDAKGNASISVTSTTVEN    848
Eco14  VTFVAAMQGELLADRTQALADGQEAVSYTLTLKTTDGKPLSGKNVTFTTTVGQLSRTQG--TTDQNGQLSVQLTSTRAGQ    593
Bpe1   AKSGRITVTGMAEPGANVTVNFPDGTRKTAVAGGDGAYTATSDGD-MVSGDIRVQATDKAGNRSPEATRAYADTVDTTPP    836
Bav2   STTGQVTAAGTAEPGNQVAVTFPDGTQKTVTADGEGHYTAESEGD-QPSGDVKAQAVDAAGNKSPETSRNYVDTVDKTAP    828
Bbr1   SSSGRVTVSGKATPRAKVKVDFPGGTSKTVTADADGRYRATSDGD-VPGGDIVVTQTGMPGAAGKPVRRPYVDTVAPTP-    723
Bpa2   ADSGRLTVSGRTEPGASVRVTFPDGETVTVTAKADGTYTATSRAD-MIGGNVTVVATDAAGNAAAPVRAAYADTVDRTP-    915
Bav1   STLGELTVSGLTQANAEVYVQFPDGSSTTVNADASGNYTAVSTSKSMPSGEIMVIATGRSAGVGSAATQTYTRNPPTAH-    668
Pma1   --------------------------------------------------------------------------------    372
Pma5   --------------------------------------------------------------------------------    373
Pma3   --------------------------------------------------------------------------------    379
Pma4   --------------------------------------------------------------------------------    410
Ssp1   --------------------------------------------------------------------------------    428
Ssp2   --------------------------------------------------------------------------------    436
Eco20  LRFVPQDTNGVTVGAISEIKPGVYSATVSSTRAGNVVVRAFSEQYQLGTLQQTLKFVAGPLDAAHSSIPLNPDKPVVGGT    702
Efe5   AEEGETETDAVLTAAPAEQVVGYNINLQLVAKDAKGNAITGDKTLRFYALDQAEGVDFGAVTEKEGVYSATVTAKRAGKI    634
Plu1   TSYYVASVTMEVDKDKVRYNNGSDSYTFTATVKDGHGNLVMGQPVNIDWQTDPKADGLKLTKQNNSVSNAQGQVTATLTS    779
Pal2   HNVDISKIQDVVLPPINNTPQAGEIPSISVVLTDSHGNPVNGVKQLEVTIAGTPHTLPATQNPDGSYTVTLPAQHSGKQD    968
Ahy1   DGSTSTTTADVDGNYTLEAPTVQGSGTITATATDKSGNTGPATSVNYIDSTVPGAPTLAATDSNSDNKPEVSGKAEPDST    781
Csu1   ATLDTVPSTTDASGQIRVSLKNNRSGITEVTATSNNSSSTAKLEFLPVLKVDNARVTVTAKSYTAGKNPVSVSSSFSGSP    637
Plu2   --------------------------------------------------------------------------------    302
Rba1   --------------------------------------------------------------------------------    327
Pas1   ERVEQEARDAWLRQEEQERLEQERLEQERLEQERLEQERLDQERVEQEARDAWLRQEEQERLEQERLGRERLEQERLGQE    664
Cla1   KGESKVKIIGLGNNGKVKIFAKNINDESVSEKNKELEVKNYKATMILPTHKHPITNTPFENGTIDYVSEFEVKLTGLMPN    945
ruler  ......2250......2260......2270......2280......2290......2300......2310......2320
```

```
 Mba1 --------------------------------------------------------------------------------   331
 Psp2 --------------------------------------------------------------------------------   251
 Sen2 KYIRVRIISDKENAESNNATSAAN--------------------------------------------------------   660
 Efe4 KQVRVRVVS--ENVPTH---------------------------------------------------------------   672
 Eco3 --------------------------------------------------------------------------------   417
 Cko1 --------------------------------------------------------------------------------   454
 Sen1 --------------------------------------------------------------------------------   460
 Eca1 --------------------------------------------------------------------------------   468
 Esp2 --------------------------------------------------------------------------------   468
 Esa3 --------------------------------------------------------------------------------   471
 Kpn1 --------------------------------------------------------------------------------   460
 Pan1 --------------------------------------------------------------------------------   490
 Eta2 --------------------------------------------------------------------------------   480
 Spr1 --------------------------------------------------------------------------------   497
 Yin1 NGSSNSGVACGDQGFGLKVNY-----------------------------------------------------------   751
 Yin2 NASSNSGVVCGDQGFGLKVNY-----------------------------------------------------------   753
 Yfr4 NGSSNSGVACGDQGFGLKVNW-----------------------------------------------------------   749
 Ymo2 NHASSSGQTAGDQGFKLKVGFH----------------------------------------------------------   686
Eco10 TYGAQAGDGLQGYGLRVLYTKK----------------------------------------------------------   734
 Eal1 KFSAQAGDGVQGYGLQVKYSKK----------------------------------------------------------   725
 Sty4 KFAANVEDGVQGYGIRVTYSQK----------------------------------------------------------   626
Eco16 KFDAEERDGVQGYGIRVVYSEKQKSA------------------------------------------------------   711
Eco25 KQEDNTVEVTLENGVKQTLIVSFVGDSSTAQVDLQKSKNEVVADGN-DSATMTATVRDAKGNLLNDVKVTFNVNSAAAKL   889
Eco15 VAEENTISATLPNGFSQRIIAKFVSDSSTPKFKQLVADPDTIIAGNSQGSTLTAIITDFHNNPLKDMKVNFVAPGGSQLD   847
 Eco6 QAVNSLIKAEIN-GSSQSVEVSFITGDISQLTSTIKTDDVSYTAGG--KIKVSVTLMDEQKNRVKGMASLLAGSSVVEVS   890
 Efe2 GEVSIMPQVGGENAARDAVKVTLSRT-FDETKSSFTTNYSEYEDEE--PIILKLSLKDKDGKPISGMAGSLNKDALLSVE   816
 Efe3 GDVYIMPKVGNLALAGAPVKVQLKSAGASVKESTLAVASTEYRAGD--EMVVALALRDKFGNPLGSEE-ALQTAEKLEVQ   815
 Yfr1 TVVTATVNGSNVSENTTFKA------------------------------------------------------------   707
 Yfr5 TNITATLNGSSQSVPVNFIP------------------------------------------------------------   669
 Yps4 SAVTATLGSQSQQVDTTFKPGSTAAISLVKLADRAVADGIDQNEIQ----------------------------------   768
 Yps7 ASLSVSVGGSALGVAPATVTLNG---------------------------------------------------------   833
 Yfr2 ATITSSINGMALNAVNVIFTAN----------------------------------------------------------   615
 Yfr3 ANIAPTIQNISLASAKVILTASD---------------------------------------------------------   571
 Yps2 ATVTVKVDGAAFSVPSVTVNFTADP-------------------------------------------------------   694
 Ype5 YTVTAELNGVTQQIDVNFIPDAG---------------------------------------------------------   681
 Pru1 LVSLNLISQNSARAPIVAFVQGALDVSKSSLNLTPSTVTAGKPTT----LKLVLKDQFGNPLVNQQTAIHVRKDKNHVVI  1604
 Pal3 --------------------------------------------------------------------------------   542
 Eta1 KVSLILPSGKYS-APDVEFRAGVPDESRSELILTPAVIEAVK--------------------------------------   778
 Sgl1 --------------------------------------------------------------------------------   659
Eco26 KVELLLPSGGKVTAPDVIFNNEDFDEDKSELKLYPETINAGKDFAN---LELVLKDKKGNYLSGQLVHGFSNNTSVIVSD   874
 Pmi1 KVSAILETGTYIADDVHFLTGHIDETMSELSLNPSQIIANGKDKAL---LTFVIKDKNGNIIPNQQVSGFSKNPTIKFSQ  1574
 Eco1 VVS-----------------------------------------------------------------------------   733
 Ymo1 --------------------------------------------------------------------------------   686
 Yen2 ALVTAAVGNNSLQATAVKFISGGVDISKSSMQLLQGNITADNLDIATIQVDIRDSKGNPLPNLASQITTSPKKGEHGLKI  1706
 Ybe1 --------------------------------------------------------------------------------   611
 Esa2 VAVSASAGGKSG--------------------------------------------------------------------   623
 Sen3 VSVVATMKEQAQ--------------------------------------------------------------------   860
Eco14 AVVNASVDSTTI--------------------------------------------------------------------   605
 Bpe1 AVPTITDVTTDATNGRITVTGVAEPGA-----------------------------------------------------   863
 Bav2 AAPTITNVATDAATGHVTVSGRAESDT-----------------------------------------------------   855
 Bbr1 MKVTIDSMRTDGNSGVVTVTGYTVGGS-----------------------------------------------------   750
 Bpa2 PVLDTPVLSVASDSGRVTVTGVTEPGARVQVAIPGESLQTVTADSA----------------------------------   961
 Bav1 -----ISSATADTAGKLSIRGQTEPGL-----------------------------------------------------   690
 Pma1 --------------------------------------------------------------------------------   372
 Pma5 --------------------------------------------------------------------------------   373
 Pma3 --------------------------------------------------------------------------------   379
 Pma4 --------------------------------------------------------------------------------   410
 Ssp1 --------------------------------------------------------------------------------   428
 Ssp2 --------------------------------------------------------------------------------   436
Eco20 VTAIWTAKDAYDNPVTSLTPEAPSLAG-----------------------------------------------------   729
 Efe5 RIGVKSDSHDFSGIEKEISFTEDR--------------------------------------------------------   658
 Plu1 TAAVENVQVSAKTASQQTAVNANGKVSFTEFSTSYYVASVTVEVDKDKAHYNNGSDSYTFTATVKDGHGNLVVGQPVNID   859
 Pal2 IQVSVNGKDSNKETLTVQAPTPIPSKVQGNTGEQGVLETVTLSSAALTGLQSGDTLDLTVTAKDAFKNPLTGLASAIALT  1048
 Ahy1 VTITWPDGTTSTTAADVDGN------------------------------------------------------------   801
 Csu1 LWSIQPSLPPSLQLDNTGKISGTVQSEVG---------------------------------------------------   666
 Plu2 --------------------------------------------------------------------------------   302
 Rba1 --------------------------------------------------------------------------------   327
 Pas1 RLEQERLEQERLERERLEQER-----------------------------------------------------------   685
 Cla1 TKVEWIAKDTQNNKTLRISNNKETTSN-----------------------------------------------------   972
ruler ......2330......2340......2350......2360......2370......2380......2390......2400
```

CLUSTAL X (1.81.1-alpha) MULTIPLE SEQUENCE ALIGNMENT

File: /Users/saierlab/Desktop/69long.ps **Date: Tue May 12 18:31:19 2009**


```
   Mba1 --------------------------------------------------------------------------------   331
   Psp2 --------------------------------------------------------------------------------   251
   Sen2 --------------------------------------------------------------------------------   660
   Efe4 --------------------------------------------------------------------------------   672
   Eco3 --------------------------------------------------------------------------------   417
   Cko1 --------------------------------------------------------------------------------   454
   Sen1 --------------------------------------------------------------------------------   460
   Eca1 --------------------------------------------------------------------------------   468
   Esp2 --------------------------------------------------------------------------------   468
   Esa3 --------------------------------------------------------------------------------   471
   Kpn1 --------------------------------------------------------------------------------   460
   Pan1 --------------------------------------------------------------------------------   490
   Eta2 --------------------------------------------------------------------------------   480
   Spr1 --------------------------------------------------------------------------------   497
   Yin1 --------------------------------------------------------------------------------   751
   Yin2 --------------------------------------------------------------------------------   753
   Yfr4 --------------------------------------------------------------------------------   749
   Ymo2 --------------------------------------------------------------------------------   686
  Eco10 --------------------------------------------------------------------------------   734
   Eal1 --------------------------------------------------------------------------------   725
   Sty4 --------------------------------------------------------------------------------   626
  Eco16 --------------------------------------------------------------------------------   711
  Eco25 SQTEVNS------HDGIATATLTSLKNGD-YTVTASVSSGSQANQQVIFIGDQSTAALTLSVPSGDITVTNT-----APL   957
  Eco15 NTTATTD------QSGIVRVHLTSSKAGS-YSVDASLEVDKNIHQSVT--------------------------------   888
   Eco6 GTDKNETGNWSEESDGVYTTTRTAKIAGDRHYATLKLSTWSSAQQSDAYAIRESGAVLAYSSIVTDKTAYTAGGAIKVTV   970
   Efe2 HALQKNS----WTETGEA--------------------------------------------------------------   830
   Efe3 HAWPLQA----WKEIGEDKYERIYQAQNTGANLQARLTISDGDISSEPYLIIAGDVNAGQSALSANPTSIEA--------   883
   Yfr1 --------------------------------------------------------------------------------   707
   Yfr5 --------------------------------------------------------------------------------   669
   Yps4 --------------------------------------------------------------------------------   768
   Yps7 --------------------------------------------------------------------------------   833
   Yfr2 --------------------------------------------------------------------------------   615
   Yfr3 --------------------------------------------------------------------------------   571
   Yps2 --------------------------------------------------------------------------------   694
   Ype5 --------------------------------------------------------------------------------   681
   Pru1 SPVTVVDDGVYQVSVSSEQADTAILSVDIGTQSLPQTKTLTVQGDTANGSISSLMPSVNHMQAGNQTGVTYY--------  1676
   Pal3 --------------------------------------------------------------------------------   542
   Eta1 --------------------------------------------------------------------------------   778
   Sgl1 --------------------------------------------------------------------------------   659
  Eco26 AREDINKPGHYKMTVTGSKSGIALLSVSVNNKTLPLTKKLVVNGDMDSWEIAEIRTNKNNVIAGDKDGVTYS--------   946
   Pmi1 AQQIS--PGRYEIEITGTQSGTAQIGVMVNGTHFKKQKILQLNADVTTWKIRAVEVDRTTITAGDK-GVNYQ--------  1643
   Eco1 --------------------------------------------------------------------------------   733
   Ymo1 ------------------------------------------------------KVNRTTFKADETDQVTYT--------   704
   Yen2 ETIANPSGDGYLVKMKGTQAGNHTVTVSVAGKPLSAKVDMVLKGDATTAKIESVKSSSPTFKADNVDTVTYT--------  1778
   Ybe1 --------------------------------------------------------------------------------   611
   Esa2 --------------------------------------------------------------------------------   623
   Sen3 --------------------------------------------------------------------------------   860
  Eco14 --------------------------------------------------------------------------------   605
   Bpe1 --------------------------------------------------------------------------------   863
   Bav2 --------------------------------------------------------------------------------   855
   Bbr1 --------------------------------------------------------------------------------   750
   Bpa2 --------------------------------------------------------------------------------   961
   Bav1 --------------------------------------------------------------------------------   690
   Pma1 --------------------------------------------------------------------------------   372
   Pma5 --------------------------------------------------------------------------------   373
   Pma3 --------------------------------------------------------------------------------   379
   Pma4 --------------------------------------------------------------------------------   410
   Ssp1 --------------------------------------------------------------------------------   428
   Ssp2 --------------------------------------------------------------------------------   436
  Eco20 --------------------------------------------------------------------------------   729
   Efe5 --------------------------------------------------------------------------------   658
   Plu1 WQTNPKADG-----------------------------------------------------------------------   868
   Pal2 HGQTGSVTWTDNQDGTYTASLTLSKLGKDSLTATANKVDSNTLKVNVTNRTGHTGVQNVVITPTNKAPQAG---------  1119
   Ahy1 --------------------------------------------------------------------------------   801
   Csu1 --------------------------------------------------------------------------------   666
   Plu2 --------------------------------------------------------------------------------   302
   Rba1 --------------------------------------------------------------------------------   327
   Pas1 --------------------------------------------------------------------------------   685
   Cla1 --------------------------------------------------------------------------------   972
  ruler ......2410......2420......2430......2440......2450......2460......2470......2480
```

```
Mba1   --------------------------------------------------------------------------------   331
Psp2   --------------------------------------------------------------------------------   251
Sen2   --------------------------------------------------------------------------------   660
Efe4   --------------------------------------------------------------------------------   672
Eco3   --------------------------------------------------------------------------------   417
Cko1   --------------------------------------------------------------------------------   454
Sen1   --------------------------------------------------------------------------------   460
Eca1   --------------------------------------------------------------------------------   468
Esp2   --------------------------------------------------------------------------------   468
Esa3   --------------------------------------------------------------------------------   471
Kpn1   --------------------------------------------------------------------------------   460
Pan1   --------------------------------------------------------------------------------   490
Eta2   --------------------------------------------------------------------------------   480
Spr1   --------------------------------------------------------------------------------   497
Yin1   --------------------------------------------------------------------------------   751
Yin2   --------------------------------------------------------------------------------   753
Yfr4   --------------------------------------------------------------------------------   749
Ymo2   --------------------------------------------------------------------------------   686
Eco10  --------------------------------------------------------------------------------   734
Eal1   --------------------------------------------------------------------------------   725
Sty4   --------------------------------------------------------------------------------   626
Eco16  --------------------------------------------------------------------------------   711
Eco25  HMTATLQDKNGNPLKDKEITFSVPNDVASRFSISNSGKGMTDSNGTAIASLTGTLAGTHMITARLANSNVSDTQPMTFVA  1037
Eco15  --------------------------------------------------------------------------------   888
Eco6   TLKDSYENLVGGQRDAINLAIQLPNTKAESIAWNEDQKGIYTATYTALLPGTG-LKAQLQMSGWANALTSNDYSISGDAA  1049
Efe2   --------------------------------------------------------------------------------   830
Efe3   --------------------------------------------------------------------------------   883
Yfr1   --------------------------------------------------------------------------------   707
Yfr5   --------------------------------------------------------------------------------   669
Yps4   --------------------------------------------------------------------------------   768
Yps7   --------------------------------------------------------------------------------   833
Yfr2   --------------------------------------------------------------------------------   615
Yfr3   --------------------------------------------------------------------------------   571
Yps2   --------------------------------------------------------------------------------   694
Ype5   --------------------------------------------------------------------------------   681
Pru1   --------------------------------------------------------------------------------  1676
Pal3   --------------------------------------------------------------------------------   542
Eta1   --------------------------------------------------------------------------------   778
Sgl1   --------------------------------------------------------------------------------   659
Eco26  --------------------------------------------------------------------------------   946
Pmi1   --------------------------------------------------------------------------------  1643
Eco1   --------------------------------------------------------------------------------   733
Ymo1   --------------------------------------------------------------------------------   704
Yen2   --------------------------------------------------------------------------------  1778
Ybe1   --------------------------------------------------------------------------------   611
Esa2   --------------------------------------------------------------------------------   623
Sen3   --------------------------------------------------------------------------------   860
Eco14  --------------------------------------------------------------------------------   605
Bpe1   --------------------------------------------------------------------------------   863
Bav2   --------------------------------------------------------------------------------   855
Bbr1   --------------------------------------------------------------------------------   750
Bpa2   --------------------------------------------------------------------------------   961
Bav1   --------------------------------------------------------------------------------   690
Pma1   --------------------------------------------------------------------------------   372
Pma5   --------------------------------------------------------------------------------   373
Pma3   --------------------------------------------------------------------------------   379
Pma4   --------------------------------------------------------------------------------   410
Ssp1   --------------------------------------------------------------------------------   428
Ssp2   --------------------------------------------------------------------------------   436
Eco20  --------------------------------------------------------------------------------   729
Efe5   --------------------------------------------------------------------------------   658
Plu1   --------------------------------------------------------------------------------   868
Pal2   --------------------------------------------------------------------------------  1119
Ahy1   --------------------------------------------------------------------------------   801
Csu1   --------------------------------------------------------------------------------   666
Plu2   --------------------------------------------------------------------------------   302
Rba1   --------------------------------------------------------------------------------   327
Pas1   --------------------------------------------------------------------------------   685
Cla1   --------------------------------------------------------------------------------   972
ruler  ......2490......2500......2510......2520......2530......2540......2550......2560
```

```
  Mba1 --------------------------------------------------------------------------------   331
  Psp2 --------------------------------------------------------------------------------   251
  Sen2 --------------------------------------------------------------------------------   660
  Efe4 --------------------------------------------------------------------------------   672
  Eco3 --------------------------------------------------------------------------------   417
  Cko1 --------------------------------------------------------------------------------   454
  Sen1 --------------------------------------------------------------------------------   460
  Eca1 --------------------------------------------------------------------------------   468
  Esp2 --------------------------------------------------------------------------------   468
  Esa3 --------------------------------------------------------------------------------   471
  Kpn1 --------------------------------------------------------------------------------   460
  Pan1 --------------------------------------------------------------------------------   490
  Eta2 --------------------------------------------------------------------------------   480
  Spr1 --------------------------------------------------------------------------------   497
  Yin1 --------------------------------------------------------------------------------   751
  Yin2 --------------------------------------------------------------------------------   753
  Yfr4 --------------------------------------------------------------------------------   749
  Ymo2 --------------------------------------------------------------------------------   686
 Eco10 --------------------------------------------------------------------------------   734
  Eal1 --------------------------------------------------------------------------------   725
  Sty4 --------------------------------------------------------------------------------   626
 Eco16 --------------------------------------------------------------------------------   711
 Eco25 DKDRAVVVLQTSKAEIIGNGVDETTLTATVKDPFDNVVKNLSVVFRTSPADTQLSLN----ARNTNENGIAEVTLKGTVL  1113
 Eco15 --------------------------------------------------------------------------------   888
  Eco6 SAQIVAMQVTTGNPDVLANGSDRHTVNVRVEDQFGNVLPEQTVTFTVTKGAAVFANAGQSADIRTDAHGMAEVDLSSTVA  1129
  Efe2 --------------------------------------------------------------------------------   830
  Efe3 --------------------------------------------------------------------------------   883
  Yfr1 --------------------------------------------------------------------------------   707
  Yfr5 --------------------------------------------------------------------------------   669
  Yps4 --------------------------------------------------------------------------------   768
  Yps7 --------------------------------------------------------------------------------   833
  Yfr2 --------------------------------------------------------------------------------   615
  Yfr3 --------------------------------------------------------------------------------   571
  Yps2 --------------------------------------------------------------------------------   694
  Ype5 --------------------------------------------------------------------------------   681
  Pru1 --------------------------------------------------------------------------------  1676
  Pal3 --------------------------------------------------------------------------------   542
  Eta1 --------------------------------------------------------------------------------   778
  Sgl1 --------------------------------------------------------------------------------   659
 Eco26 --------------------------------------------------------------------------------   946
  Pmi1 --------------------------------------------------------------------------------  1643
  Eco1 --------------------------------------------------------------------------------   733
  Ymo1 --------------------------------------------------------------------------------   704
  Yen2 --------------------------------------------------------------------------------  1778
  Ybe1 --------------------------------------------------------------------------------   611
  Esa2 --------------------------------------------------------------------------------   623
  Sen3 --------------------------------------------------------------------------------   860
 Eco14 --------------------------------------------------------------------------------   605
  Bpe1 --------------------------------------------------------------------------------   863
  Bav2 --------------------------------------------------------------------------------   855
  Bbr1 --------------------------------------------------------------------------------   750
  Bpa2 --------------------------------------------------------------------------------   961
  Bav1 --------------------------------------------------------------------------------   690
  Pma1 --------------------------------------------------------------------------------   372
  Pma5 --------------------------------------------------------------------------------   373
  Pma3 --------------------------------------------------------------------------------   379
  Pma4 --------------------------------------------------------------------------------   410
  Ssp1 --------------------------------------------------------------------------------   428
  Ssp2 --------------------------------------------------------------------------------   436
 Eco20 --------------------------------------------------------------------------------   729
  Efe5 --------------------------------------------------------------------------------   658
  Plu1 --------------------------------------------------------------------------------   868
  Pal2 --------------------------------------------------------------------------------  1119
  Ahy1 --------------------------------------------------------------------------------   801
  Csu1 --------------------------------------------------------------------------------   666
  Plu2 --------------------------------------------------------------------------------   302
  Rba1 --------------------------------------------------------------------------------   327
  Pas1 --------------------------------------------------------------------------------   685
  Cla1 --------------------------------------------------------------------------------   972
 ruler ......2570......2580......2590......2600......2610......2620......2630......2640
```

```
   Mba1 --------------------------------------------------------------------------------  331
   Psp2 --------------------------------------------------------------------------------  251
   Sen2 --------------------------------------------------------------------------------  660
   Efe4 --------------------------------------------------------------------------------  672
   Eco3 --------------------------------------------------------------------------------  417
   Cko1 --------------------------------------------------------------------------------  454
   Sen1 --------------------------------------------------------------------------------  460
   Eca1 --------------------------------------------------------------------------------  468
   Esp2 --------------------------------------------------------------------------------  468
   Esa3 --------------------------------------------------------------------------------  471
   Kpn1 --------------------------------------------------------------------------------  460
   Pan1 --------------------------------------------------------------------------------  490
   Eta2 --------------------------------------------------------------------------------  480
   Spr1 --------------------------------------------------------------------------------  497
   Yin1 --------------------------------------------------------------------------------  751
   Yin2 --------------------------------------------------------------------------------  753
   Yfr4 --------------------------------------------------------------------------------  749
   Ymo2 --------------------------------------------------------------------------------  686
  Eco10 --------------------------------------------------------------------------------  734
   Eal1 --------------------------------------------------------------------------------  725
   Sty4 --------------------------------------------------------------------------------  626
  Eco16 --------------------------------------------------------------------------------  711
  Eco25 GVHTAEAILLNGNRDTKIVNIAPDASNAQVTLNIPAQQVVTNNSDSVQLTATVKDPSNHPVAGITVNFTMPQDVAANFTL 1193
  Eco15 --------------------------------------------------------------------------------  888
   Eco6 DASTVEAKINQSSDSKTVNFVADVSTAQVAELVVTQDGSVADGATANTLRARVTDAFGNALAGQTVS------VLAGNGA 1203
   Efe2 --------------------------------------------------------------------------------  830
   Efe3 --------------------------------------------------------------------------------  883
   Yfr1 --------------------------------------------------------------------------------  707
   Yfr5 --------------------------------------------------------------------------------  669
   Yps4 --------------------------------------------------------------------------------  768
   Yps7 --------------------------------------------------------------------------------  833
   Yfr2 --------------------------------------------------------------------------------  615
   Yfr3 --------------------------------------------------------------------------------  571
   Yps2 --------------------------------------------------------------------------------  694
   Ype5 --------------------------------------------------------------------------------  681
   Pru1 -------------------------------------------------------------------------------- 1676
   Pal3 --------------------------------------------------------------------------------  542
   Eta1 --------------------------------------------------------------------------------  778
   Sgl1 --------------------------------------------------------------------------------  659
  Eco26 --------------------------------------------------------------------------------  946
   Pmi1 -------------------------------------------------------------------------------- 1643
   Eco1 --------------------------------------------------------------------------------  733
   Ymo1 --------------------------------------------------------------------------------  704
   Yen2 -------------------------------------------------------------------------------- 1778
   Ybe1 --------------------------------------------------------------------------------  611
   Esa2 --------------------------------------------------------------------------------  623
   Sen3 --------------------------------------------------------------------------------  860
  Eco14 --------------------------------------------------------------------------------  605
   Bpe1 --------------------------------------------------------------------------------  863
   Bav2 --------------------------------------------------------------------------------  855
   Bbr1 --------------------------------------------------------------------------------  750
   Bpa2 --------------------------------------------------------------------------------  961
   Bav1 --------------------------------------------------------------------------------  690
   Pma1 --------------------------------------------------------------------------------  372
   Pma5 --------------------------------------------------------------------------------  373
   Pma3 --------------------------------------------------------------------------------  379
   Pma4 --------------------------------------------------------------------------------  410
   Ssp1 --------------------------------------------------------------------------------  428
   Ssp2 --------------------------------------------------------------------------------  436
  Eco20 --------------------------------------------------------------------------------  729
   Efe5 --------------------------------------------------------------------------------  658
   Plu1 --------------------------------------------------------------------------------  868
   Pal2 -------------------------------------------------------------------------------- 1119
   Ahy1 --------------------------------------------------------------------------------  801
   Csu1 --------------------------------------------------------------------------------  666
   Plu2 --------------------------------------------------------------------------------  302
   Rba1 --------------------------------------------------------------------------------  327
   Pas1 --------------------------------------------------------------------------------  685
   Cla1 --------------------------------------------------------------------------------  972
  ruler ......2650......2660......2670......2680......2690......2700......2710......2720
```

```
Mba1   --------------------------------------------------------------------------------   331
Psp2   --------------------------------------------------------------------------------   251
Sen2   --------------------------------------------------------------------------------   660
Efe4   --------------------------------------------------------------------------------   672
Eco3   --------------------------------------------------------------------------------   417
Cko1   --------------------------------------------------------------------------------   454
Sen1   --------------------------------------------------------------------------------   460
Eca1   --------------------------------------------------------------------------------   468
Esp2   --------------------------------------------------------------------------------   468
Esa3   --------------------------------------------------------------------------------   471
Kpn1   --------------------------------------------------------------------------------   460
Pan1   --------------------------------------------------------------------------------   490
Eta2   --------------------------------------------------------------------------------   480
Spr1   --------------------------------------------------------------------------------   497
Yin1   --------------------------------------------------------------------------------   751
Yin2   --------------------------------------------------------------------------------   753
Yfr4   --------------------------------------------------------------------------------   749
Ymo2   --------------------------------------------------------------------------------   686
Eco10  --------------------------------------------------------------------------------   734
Eal1   --------------------------------------------------------------------------------   725
Sty4   --------------------------------------------------------------------------------   626
Eco16  --------------------------------------------------------------------------------   711
Eco25  ENNGIAITQANGEAHVTLKGKKAGTHTVTATLGNNNASDAQPVTFVADKDSAVVVLQTSKAEIIGNGVDETTLTATVKDP  1273
Eco15  --------------------------------------------------------------------------------   888
Eco6   TTAPTVTTQPDGTVEISVTSQTAGTSVITASVNNSSQS-RNVTFIADVSTAQIADLVVSQDNAVADGATANTLQVRVTDA  1282
Efe2   --------------------------------------------------------------------------------   830
Efe3   ---------------------------------NNKEMPSTLTYKAMDAWKNPVTGLTDNNSLTLKVTGLGGTEFSGFTD   930
Yfr1   --------------------------------------------------------------------------------   707
Yfr5   --------------------------------------------------------------------------------   669
Yps4   --------------------------------------------------------------------------VVLRDG   774
Yps7   --------------------------------------------------------------------------------   833
Yfr2   --------------------------------------------------------------------------------   615
Yfr3   --------------------------------------------------------------------------------   571
Yps2   --------------------------------------------------------------------------------   694
Ype5   --------------------------------------------------------------------------------   681
Pru1   ------------------------------------------------------------------ATVVDQFNNPLPSI  1690
Pal3   --------------------------------------------------------------------------------   542
Eta1   --------------------------------------------------------------------------------   778
Sgl1   --------------------------------------------------------------------------------   659
Eco26  ------------------------------------------------------------------TLVKDKHGNILPGV   960
Pmi1   ------------------------------------------------------------------ATVVDANNNVLPNV  1657
Eco1   --------------------------------------------------------------------------------   733
Ymo1   ------------------------------------------------------------------AKVVDINNNPLENF   718
Yen2   ------------------------------------------------------------------AKVVDANNNLLENI  1792
Ybe1   --------------------------------------------------------------------------------   611
Esa2   --------------------------------------------------------------------------------   623
Sen3   --------------------------------------------------------------------------------   860
Eco14  --------------------------------------------------------------------------------   605
Bpe1   --------------------------------------------------------------------------------   863
Bav2   --------------------------------------------------------------------------------   855
Bbr1   --------------------------------------------------------------------------------   750
Bpa2   --------------------------------------------------------------------------------   961
Bav1   --------------------------------------------------------------------------------   690
Pma1   --------------------------------------------------------------------------------   372
Pma5   --------------------------------------------------------------------------------   373
Pma3   --------------------------------------------------------------------------------   379
Pma4   --------------------------------------------------------------------------------   410
Ssp1   --------------------------------------------------------------------------------   428
Ssp2   --------------------------------------------------------------------------------   436
Eco20  --------------------------------------------------------------------------------   729
Efe5   --------------------------------------------------------------------------------   658
Plu1   --------------------------------------------------------------------------------   868
Pal2   ---------------------------------------------------------ETPTLTVTLTDSNGNPVNDIQQL  1142
Ahy1   --------------------------------------------------------------------------------   801
Csu1   --------------------------------------------------------------------------------   666
Plu2   --------------------------------------------------------------------------------   302
Rba1   --------------------------------------------------------------------------------   327
Pas1   --------------------------------------------------------------------------------   685
Cla1   --------------------------------------------------------------------------------   972
ruler  ......2730......2740......2750......2760......2770......2780......2790......2800
```

```
Mba1   --------------------------------------------------------------------------------   331
Psp2   --------------------------------------------------------------------------------   251
Sen2   --------------------------------------------------------------------------------   660
Efe4   --------------------------------------------------------------------------------   672
Eco3   --------------------------------------------------------------------------------   417
Cko1   --------------------------------------------------------------------------------   454
Sen1   --------------------------------------------------------------------------------   460
Eca1   --------------------------------------------------------------------------------   468
Esp2   --------------------------------------------------------------------------------   468
Esa3   --------------------------------------------------------------------------------   471
Kpn1   --------------------------------------------------------------------------------   460
Pan1   --------------------------------------------------------------------------------   490
Eta2   --------------------------------------------------------------------------------   480
Spr1   --------------------------------------------------------------------------------   497
Yin1   --------------------------------------------------------------------------------   751
Yin2   --------------------------------------------------------------------------------   753
Yfr4   --------------------------------------------------------------------------------   749
Ymo2   --------------------------------------------------------------------------------   686
Eco10  --------------------------------------------------------------------------------   734
Eal1   --------------------------------------------------------------------------------   725
Sty4   --------------------------------------------------------------------------------   626
Eco16  --------------------------------------------------------------------------------   711
Eco25  FDNAVKDLQVTFSTNPADTQLSQSKSNTNDSGVAEVTFKGTVLGVHTAEATLPNGNNDTKIVNIAPDASNAQVTLNIPAQ  1353
Eco15  -------------------------------------------------------------ITVVPNREQSVMTLNAGSG   907
Eco6   FGNALAGQTVSVLADNGATVAPVVTTQPD--GTVEISVTSQTAGSSAVTVSINSSSQSRDVTFIADVRTAQIADLVVIKD  1360
Efe2   --------------------------------------------------------------------------------   830
Efe3   KGEGIYTGTLTGTQSGQASLMPQVNNVDMAPDVTSVTLTAGKAVAKNSRINVGAANFTAGTRFTVTVTLRDASENPVTGA  1010
Yfr1   --------------DATTAEIADT-------------------------------------------------DFTIASG   724
Yfr5   --------------DNGTATIAAG-------------------------------------------------ALTVLTD   686
Yps4   TGNAVPNVPMSIQADNGAIVVASTPNTGVDGTINATFTNLRAGESVVSVTSPALVGMTMTMTFSADQRTAVVSTLAAIDN   854
Yps7   --------------DSGNLSTTHS-------------------------------------------------TLVAAPV   850
Yfr2   -----------------AATFVVD-------------------------------------------------SLTADTH   629
Yfr3   --------------N--ALSPENS-------------------------------------------------LFSVEPT   586
Yps2   -----------------IPDAGRS-------------------------------------------------SFTVSTP   708
Ype5   -----------------TATLDDSDE----------------------------------------------YKLQWVTN   698
Pru1   HVSWHLQGKAEPFAHSTVTNAQGVAHVKVTSHQMGQLVMTAVL-------SSSQNKVATPVQVNAGAVNSQNSTFTASKM  1763
Pal3   --------------------------------------------------------------------------------   542
Eta1   --------------------------------------------------------------------------------   778
Sgl1   --------------------------------------------------------------------------------   659
Eco26  IVSWQLNGNSESFAPVSRTNAEGIATTTVRSNTAGELKMRAYL-------DEANYKDAANVTVIAGDIDSKNSDFSLSKY  1033
Pmi1   IVSWKLLGSADDYHYSTYTNDKGIATNRVTSHVAGRLKMSAYL-------DSNNYKSTQDITVIPAEIDIHKSTFNSHRR  1730
Eco1   --------------------------------------------------------------------------------   733
Ymo1   PVSWRLTQGEGQYKSLSYTGPTGEAETKLSASRLGQYKMEAQIGILNGSPTRIVEKAAPDVSTTAGDIDPSKSDLVVDVN   798
Yen2   AVSWRLAQGEGQYQGQSYTGKTGVATTKLSASRLGTYKMEAQV--------RQQVKAAAGVNSTAGDADPSQSDFVVDVA  1864
Ybe1   --------------------------------------------------------------------------------   611
Esa2   --------------------------------------------------------SASAVFTAVPVNDVAVTMTTNNSP   647
Sen3   --------------------------------------------------------TSPDLQFTVDNATAAVESLNADKT   884
Eco14  --------------------------------------------------------SAAPVTFENRLDSAIVVNKTS---   626
Bpe1   --------------------------------------------------------------------------------   863
Bav2   --------------------------------------------------------------------------------   855
Bbr1   --------------------------------------------------------------------------------   750
Bpa2   ----------GRYRAVSPGDVLRSGTVRVIATDAHGNASAPRDVPYQDAVDRTAPPLTIEAAVEQARTGVVNVSGKTEPN  1031
Bav1   --------------------------------------------------------------------------------   690
Pma1   --------------------------------------------------------------------------------   372
Pma5   --------------------------------------------------------------------------------   373
Pma3   --------------------------------------------------------------------------------   379
Pma4   --------------------------------------------------------------------------------   410
Ssp1   --------------------------------------------------------------------------------   428
Ssp2   --------------------------------------------------------------------------------   436
Eco20  -------------AAAVGSTASGWTNNGDGTWTAQITLGSTAGELEVMPKLNGQDAAANAAKVTVVADALSSNQSKVSVA   796
Efe5   -------------------------------------------------------------------MQFAFSRFEASKN   671
Plu1   ---------LKLTKQSNSVSNAQGQVTATLTSTVAVENVQVSAKTASQQTAVNANGKVSFTEFSTSYYVASVTVEVDKDK   939
Pal2   EVTIAGTTHTLPATQDPDGSYTVTLPAQHSGKQDIQVSVNGKDSNKETLTVQAPTPIPSKVQGNMGEQGVLETVTLSSAA  1222
Ahy1   ------------------------------------------------------------------------------YT   803
Csu1   ------------------------------------------------------IPETNYTVFVRDSLGTQRASTSFSLV   692
Plu2   --------------------------------------------------------------------------------   302
Rba1   --------------------------------------------------------------------------------   327
Pas1   ----------------------------------------------------------------------------LEQE   689
Cla1   ---------------------------------------------------------------------------EKGES   977
ruler  ......2810......2820......2830......2840......2850......2860......2870......2880
```

```
 Mba1 --------------------------------------------------------------------------------  331
 Psp2 --------------------------------------------------------------------------------  251
 Sen2 --------------------------------------------------------------------------------  660
 Efe4 --------------------------------------------------------------------------------  672
 Eco3 --------------------------------------------------------------------------------  417
 Cko1 --------------------------------------------------------------------------------  454
 Sen1 --------------------------------------------------------------------------------  460
 Eca1 --------------------------------------------------------------------------------  468
 Esp2 --------------------------------------------------------------------------------  468
 Esa3 --------------------------------------------------------------------------------  471
 Kpn1 --------------------------------------------------------------------------------  460
 Pan1 --------------------------------------------------------------------------------  490
 Eta2 --------------------------------------------------------------------------------  480
 Spr1 --------------------------------------------------------------------------------  497
 Yin1 --------------------------------------------------------------------------------  751
 Yin2 --------------------------------------------------------------------------------  753
 Yfr4 --------------------------------------------------------------------------------  749
 Ymo2 --------------------------------------------------------------------------------  686
Eco10 --------------------------------------------------------------------------------  734
 Eal1 --------------------------------------------------------------------------------  725
 Sty4 --------------------------------------------------------------------------------  626
Eco16 --------------------------------------------------------------------------------  711
Eco25 QVVTNNSDSVQLTATVKDPSNHPVAGITVNFTMPQDVAANFTLENN---------------------------------- 1399
Eco15 SAIANNTNIVTLTASVKDVYGHPLPDEDVKFTLPASMTGNFTLSS-----------------------------------  952
 Eco6 DSVADGAMANMLRARVTDVFGNALAGQTVSVMADNGAAVASTMTT----------------------------------- 1405
 Efe2 --------------------------GEYTIEYIADSVGNGLMASL----------------------------------  850
 Efe3 EDLLTNETVKVPGATRKGAEWTGNGDGTYRAEWEAEQAGNGYKATL---------------------------------- 1056
 Yfr1 -AVANGSATNALSATVKDAGGNTVPNVSVTFAVTGGATFAG---------------------------------------  764
 Yfr5 NALANGSAPNSVKAVVTDAGGNPIPNFVVSFVGSNGANIAAN--------------------------------------  728
 Yps4 NAKADGTDTNVVRAWVVDANGNSVPGVSVTFDAGNGAVLAQNPVVTDRNGYAENTLTNLAIGTTTVKATTVTDPVGQTVN  934
 Yps7 SIEANGSDTSLVTLTLRDSNNNPVTGQTVALVSTLGTLGAVT--------------------------------------  892
 Yfr2 TTFADGNSLITFTATVKDS-------------------------------------------------------------  648
 Yfr3 EIIADGVQTSVLSFTAQDI-------------------------------------------------------------  605
 Yps2 DILADGTMSSTLSFVPVDKNGHFISGMQGLSFTQNGVPVSIS--------------------------------------  750
 Ype5 GQVADGESTNSVQLTVVDKFGNTVPGVDVAFTTDIGAIISEVTP------------------------------------  742
 Pru1 DIGPDGIEETILTIKLQDDFGNPLSNEIVTINSNAPN--TDFTI------------------------------------ 1805
 Pal3 --------------------------------------------------------------------------------  542
 Eta1 -------EPAMLTLILRDKNGNTLSGNTVRGVSDNSL--VTISN------------------------------------  813
 Sgl1 -----------------DANGNPIPNHVVSFSADNGA--TIIDN------------------------------------  684
Eco26 NIGADGIETSTLTLILKDKYGNSIPGKIVTVNAKTKTGQLSLDN------------------------------------ 1077
 Pmi1 SINADNKDSTLLSVKLMDKYGNTIDGKNVEIKTISGK--PNFSD------------------------------------ 1772
 Eco1 -------------------AKVSEAGSVVNADAVNFFATLSIDN------------------------------------  758
 Ymo1 SIDASGKTKAKLTATLKDKFGNLLKGQTVNVKETQNLTGIKFSV------------------------------------  842
 Yen2 SIDSSGNTKAKLTATLKDKFGNLLSGQKVKLTDSNSLKKITLSA------------------------------------ 1908
 Ybe1 --------------------GPAATDASTYQWGEKGATAANVAN------------------------------------  635
 Esa2 ADGNTAN---VAQALVTSASGQPMPGVSVTWNVGTATATTPLTV------------------------------------  688
 Sen3 QAVANQNDFITLTARVVDANDHPVPDSPLKWQIVEGQATLSATQ------------------------------------  928
Eco14 -AVADGQDSIILTAVIRDAAGVPVAGQAVTWHTDNGQFTQQDAV------------------------------------  669
 Bpe1 -NVTVDFPDGTRKTVVAGGDGAYTATSDRDMA-SGDVRVQAIDKG-----------------------------------  906
 Bav2 -QVTVTFPSGESKQVPADAGGAYRVSSDTDQP-SGEIKAQAADAA-----------------------------------  898
 Bbr1 -TVTVTFPDGTTAGTTANDRGKYTVTSTADIP-AGPIRVSARGPR-----------------------------------  793
 Bpa2 LMVSATFPDGQTAQARADGQGRYQLASPADVARSGRIAVSASDAA----------------------------------- 1076
 Bav1 -SVFVLFPSGDDMTVQADAQGNFHATSSQKET-AGIVSVTVSGKD-----------------------------------  733
 Pma1 --------------------------------------------------------------------------------  372
 Pma5 --------------------------------------------------------------------------------  373
 Pma3 --------------------------------------------------------------------------------  379
 Pma4 --------------------------------------------------------------------------------  410
 Ssp1 --------------------------------------------------------------------------------  428
 Ssp2 --------------------------------------------------------------------------------  436
Eco20 EDHVKAGESTTVTLIAKDAHGNTISGLSLSASLTGTASEGATVSS-----------------------------------  841
 Efe5 NALADGKQQNTVTVSLADRFGNVVPGYAVTLSLPSGITQVGGEHAVS---------------------------------  718
 Plu1 AHYNNGSDSYTFTATVKDGHGNLVVGQPVNIDWQTDPKADGLKLTKQ---------------------------------  986
 Pal2 LTGLQSGDTLDLTVMAKDAFKNPLTGLASAIVLTHGQTGSVVWKDHHDG------------------------------- 1271
 Ahy1 LEAPTVQGSGTITATATDKSGNTGPASSVNYLASLVGVTITGLVDG----------------------------------  849
 Csu1 VNPPFVISQKLYRRVLSTDSQVNIQAFEITGGEPPYTVTVSPTLPSG---------------------------------  739
 Plu2 --------------------------------------------------------------------------------  302
 Rba1 --------------------------------------------------------------------------------  327
 Pas1 RLERERLDQERVEQEARDAWLRQEEQERLEQERLEQERLERERLEQ----------------------------------  735
 Cla1 KVVFEAIKDYNVKNLEITAIYNQSLANKIEVKDNIKLYQYSLAMN----------------------------------- 1022
ruler ......2890......2900......2910......2920......2930......2940......2950......2960
```

```
  Mba1 --------------------------------------------------------------------------------  331
  Psp2 --------------------------------------------------------------------------------  251
  Sen2 --------------------------------------------------------------------------------  660
  Efe4 --------------------------------------------------------------------------------  672
  Eco3 --------------------------------------------------------------------------------  417
  Cko1 --------------------------------------------------------------------------------  454
  Sen1 --------------------------------------------------------------------------------  460
  Eca1 --------------------------------------------------------------------------------  468
  Esp2 --------------------------------------------------------------------------------  468
  Esa3 --------------------------------------------------------------------------------  471
  Kpn1 --------------------------------------------------------------------------------  460
  Pan1 --------------------------------------------------------------------------------  490
  Eta2 --------------------------------------------------------------------------------  480
  Spr1 --------------------------------------------------------------------------------  497
  Yin1 --------------------------------------------------------------------------------  751
  Yin2 --------------------------------------------------------------------------------  753
  Yfr4 --------------------------------------------------------------------------------  749
  Ymo2 --------------------------------------------------------------------------------  686
 Eco10 --------------------------------------------------------------------------------  734
  Eal1 --------------------------------------------------------------------------------  725
  Sty4 --------------------------------------------------------------------------------  626
 Eco16 --------------------------------------------------------------------------------  711
 Eco25 ------------------------------------------------------------GIAITQANGEAHVTLKGKKA  1419
 Eco15 ------------------------------------------------------------ETARTDANGDAVVTLRGTKA  972
  Eco6 -----------------------------------------------------------------KPDGTVEISVTSQTA  1420
  Efe2 ------------------------------------------------------------KLMSWKNE--KTATYNIT-A  867
  Efe3 ------------------------------------------------------------HLAGWGDEEMQSAPYNIQPG  1076
  Yfr1 --------------------------------------------------------------------------------  764
  Yfr5 --------------------------------------------------------------GTTGADGSVTQPLTNTTA  746
  Yps4 THFVAGAVDTITLTVLVNGAVANGVNTNSVQAVVSDSGGNPVNGAAVVFSSANATAQITTVIGTTGVDGIATATLTNTVA  1014
  Yps7 -----------------------------------EQASGVYTATLTAGTVAGVASLSVSVGGSALGVAPATVTLNGDSG  937
  Yfr2 ---------------------------------------------------------------SGHGVPNTDVILN----  661
  Yfr3 --------------------------------------------------------------------------------  605
  Yps2 --------------------------------------------------------------------------------  750
  Ype5 ----------------------------------------------------------------TDANGVATAKIISSQA  758
  Pru1 --------------------------------------------------------------ARITNNQDGSYTSTATAT  1823
  Pal3 --------------------------------------------------------------------------------  542
  Eta1 --------------------------------------------------------------GEEIANKPGNYGMTVTAS  831
  Sgl1 --------------------------------------------------------------ATTNAN--GAAVADLTSV  700
 Eco26 --------------------------------------------------------------NPMKEVGDGIYISNAKSS  1095
  Pmi1 --------------------------------------------------------------NPLKSVGNGEYQTNVTAN  1790
  Eco1 --------------------------------------------------------------NVEIVGTKVRGELPNIWL  776
  Ymo1 --------------------------------------------------------------NPMKDNGDGTYSTEVTST  860
  Yen2 --------------------------------------------------------------NPMKDNGDGTYSTEVTAT  1926
  Ybe1 --------------------------------------------------------------GSAVATSGQVPEYLLKST  653
  Esa2 --------------------------------------------------------------TTN---ASGIATVSLTDA  703
  Sen3 --------------------------------------------------------------TTTDQQGSGSITLTSSTQ  946
 Eco14 --------------------------------------------------------------TNAAG--TASATLTSTQA  685
  Bpe1 --------------------------------------------------------------GNRSPEATRAYADTVDKT  924
  Bav2 --------------------------------------------------------------GNKSPEATKAYDDTVDKT  916
  Bbr1 --------------------------------------------------------------NQQGS-ATDHYLDAWTKQ  810
  Bpa2 --------------------------------------------------------------GNRAA-ASAEFTDQVDKT  1093
  Bav1 --------------------------------------------------------------GGVGVPVEYHYSTA----  747
  Pma1 --------------------------------------------------------------------------------  372
  Pma5 --------------------------------------------------------------------------------  373
  Pma3 --------------------------------------------------------------------------------  379
  Pma4 --------------------------------------------------------------------------------  410
  Ssp1 --------------------------------------------------------------------------------  428
  Ssp2 --------------------------------------------------------------------------------  436
 Eco20 -------------------------------------------------------------WTEKGDCSYVATLTTGGKT  860
  Efe5 ----------------------------------------------------------------TDENGNAVFTLVSSTP  734
  Plu1 ------------------------------------------------------------NNSVSNAQGQVTATLISTAA  1006
  Pal2 -------------------------------------------IYTTSLPLTKLGSDSLTATINTIASQPISITVETQQT  1308
  Ahy1 ----------------------------------------------------------------FPQVGAVLTAVSDCGS  865
  Csu1 --------------------------------------------------------------LSVSNTGVISGQLGTAQT  757
  Plu2 --------------------------------------------------------------------------------  302
  Rba1 --------------------------------------------------------------------------------  327
  Pas1 --------------------------------------------------------------ERLEQERLERERLDQERV  753
  Cla1 --------------------------------------------------------------SQKDKLDAYENGLATKNL  1040
 ruler ......2970......2980......2990......3000......3010......3020......3030......3040
```

```
Mba1   --------------------------------------------------------------------------------  331
Psp2   --------------------------------------------------------------------------------  251
Sen2   --------------------------------------------------------------------------------  660
Efe4   --------------------------------------------------------------------------------  672
Eco3   --------------------------------------------------------------------------------  417
Cko1   --------------------------------------------------------------------------------  454
Sen1   --------------------------------------------------------------------------------  460
Eca1   --------------------------------------------------------------------------------  468
Esp2   --------------------------------------------------------------------------------  468
Esa3   --------------------------------------------------------------------------------  471
Kpn1   --------------------------------------------------------------------------------  460
Pan1   --------------------------------------------------------------------------------  490
Eta2   --------------------------------------------------------------------------------  480
Spr1   --------------------------------------------------------------------------------  497
Yin1   --------------------------------------------------------------------------------  751
Yin2   --------------------------------------------------------------------------------  753
Yfr4   --------------------------------------------------------------------------------  749
Ymo2   --------------------------------------------------------------------------------  686
Eco10  --------------------------------------------------------------------------------  734
Eal1   --------------------------------------------------------------------------------  725
Sty4   --------------------------------------------------------------------------------  626
Eco16  --------------------------------------------------------------------------------  711
Eco25  GTHTVTATLSNNNTSDSQPVTFVADK-TSALVVLQISKNEITGNGVDSATLTATVKDQFDNEVNNLPVTFSTASSGLTLT  1498
Eco15  GEFTVTATLTRNNTVAYQQVT-----------------------------------------------------------  993
Eco6   GVSAVTATIN--SSTQSQNVTFIADVRTAKIADLVVIKDGSEADGSTANTLRARVTDAFGNALAGQTVSVLAGNGATTAP  1498
Efe2   QPRNMDGSIIVGGVGFAS----------------------------------------------------------SQTP  889
Efe3   EPTEAQSHISTDGTIFTTGDAIPVTV-------------------------------------------TLKDAWGNHTP  1113
Yfr1   ------------------------GT------------------------------------------------------  766
Yfr5   GVTHVTASVNGHSQAVDVTFVPDSGT------------------------------------------------------  772
Yps4   GTSNVVATIDTVNANIDTTFVAGAVA------------------------------------------------------  1040
Yps7   NLSTTHSTLVAAPVSIEANGSDTSLV------------------------------------------------------  963
Yfr2   ---TTGGVLSASSVTTDTHG----VA------------------------------------------------------  680
Yfr3   ----HHLPIKGLTVAFDVTG----IP------------------------------------------------------  623
Yps2   -------PITEQPDSYTATVVGNSVG------------------------------------------------------  769
Ype5   KSHTVKATLNRKEQTVEVNFIADTAT------------------------------------------------------  784
Pru1   KQGTVRLTARVGSDIIAN---PLNIK------------------------------------------------------  1846
Pal3   --------------------------------------------------------------------------------  542
Eta1   KAGKAILSVTVNGKPFGQLKTLLTIT------------------------------------------------------  857
Sgl1   KASEVTVTAALDN-------------------------------------------------------------------  713
Eco26  YQGRFELTLDINGNKFSR---SQVLT------------------------------------------------------  1118
Pmi1   TMSDIILTAQAETITIAEP---LTIK------------------------------------------------------  1813
Eco1   RYGQVKLNANGGNG------------------------------------------------------------------  790
Ymo1   TKGNTTFIASINSVDLTQQ---PQML------------------------------------------------------  883
Yen2   AKGNTRFIARVNGVDLTQQ---PQIV------------------------------------------------------  1949
Ybe1   DVGKVMEISVQAKNSLAVTG------------------------------------------------------------  673
Esa2   TAETVAVTASAGGKSGSASAVFTAVP------------------------------------------------------  729
Sen3   GNVVVSVSATAGDAVNSPALLFIADT------------------------------------------------------  972
Eco14  GNAQVSLSLNG---TTTTVSAPRVSF------------------------------------------------------  708
Bpe1   AP------------------------------------------------------------------------------  926
Bav2   APPLSITNVSTAPATGVVTVTGNTEA----------------------------------------------GSSVAVNF  950
Bbr1   TLLGGKIRLLRPVAR-----------------------------------------------------------------  825
Bpa2   APRAPTLQLATDTATGRVTASGKAEP----------------------------------------------GAIVEITW  1127
Bav1   --------------------------------------------------------------------------------  747
Pma1   --------------------------------------------------------------------------------  372
Pma5   --------------------------------------------------------------------------------  373
Pma3   --------------------------------------------------------------------------------  379
Pma4   --------------------------------------------------------------------------------  410
Ssp1   --------------------------------------------------------------------------------  428
Ssp2   --------------------------------------------------------------------------------  436
Eco20  GELRVMPLFNGQPAATEAAQLTVIAG------------------------------------------------------  886
Efe5   GSYVIKAHAGPQDSSELTVTFASNMTG-----------------------------------------------------  761
Plu1   VKDVQVSAKAVANPSWVEADRKVSFEELSLSYQVTG---------------------------------------VTVEV  1047
Pal2   KTSVFDIELHAQHPLISAGQSTTLTLTLRDKYNNNVEKIPSSDIKLEDNKLTLSNIQWKEQGNGVYTTEISFKQLGNHKL  1388
Ahy1   SACRAGLTWQWQIEDGVGSGNFVDIG------------------------------------------------------  891
Csu1   QEQTYTFTVQDQQQESKNISIVLAVVTG----------------------------------------------------  785
Plu2   --------------------------------------------------------------------------------  302
Rba1   --------------------------------------------------------------------------------  327
Pas1   EQEARDAWLRQEEQERLEQERLEQER------------------------------------------------------  779
Cla1   VLDKTEVKVTGGKPNEKVEWDLTGDG------------------------------------------------------  1066
ruler  ......3050......3060......3070......3080......3090......3100......3110......3120
```

```
Mba1   --------------------------------------------------------------------------------  331
Psp2   --------------------------------------------------------------------------------  251
Sen2   --------------------------------------------------------------------------------  660
Efe4   --------------------------------------------------------------------------------  672
Eco3   --------------------------------------------------------------------------------  417
Cko1   --------------------------------------------------------------------------------  454
Sen1   --------------------------------------------------------------------------------  460
Eca1   --------------------------------------------------------------------------------  468
Esp2   --------------------------------------------------------------------------------  468
Esa3   --------------------------------------------------------------------------------  471
Kpn1   --------------------------------------------------------------------------------  460
Pan1   --------------------------------------------------------------------------------  490
Eta2   --------------------------------------------------------------------------------  480
Spr1   --------------------------------------------------------------------------------  497
Yin1   --------------------------------------------------------------------------------  751
Yin2   --------------------------------------------------------------------------------  753
Yfr4   --------------------------------------------------------------------------------  749
Ymo2   --------------------------------------------------------------------------------  686
Eco10  --------------------------------------------------------------------------------  734
Eal1   --------------------------------------------------------------------------------  725
Sty4   --------------------------------------------------------------------------------  626
Eco16  --------------------------------------------------------------------------------  711
Eco25  PGESNTNESGIAQATLAGVAFGEQTVTASLANNGASDNKTVHFIGDTAAAKIIELTPVPDSIIAGTPQNSSGSVITATVV  1578
Eco15  ------------------------------------------FIGDTNSAQLQPLTASLNSIVAG---NSTGSTLTATIL  1028
Eco6   TVTTQPDGTVEISVTSQTAG----ISAVTASINNSSQSRNVTFIADVRTAKIADLVVIKDGSEAD---GSTANTLRARVT  1571
Efe2   ------------------------------------RFPTTGFRG-----------ATFTITG--------------NNF  908
Efe3   GQASYLSTHVVAAHTVPQPTQWQENSDGTYSAQFIAGEPGTGLQVTLNQDSGWKVAAIYSIIAGEPAINNSFVDTDKNNY  1193
Yfr1   ------QTSL----------------------------------------------------------------------  770
Yfr5   ------ATIISGAL------------------------------------------------------------------  780
Yps4   ------TITLTTLVNGAVADGANSNSVQAVVSDSGGNPVTGAAVVFSSANATAQITTVIGTTGVDGIATATLTNTVAGTS  1114
Yps7   ------TLTLRDSNNNPVTGQTVALVSTLGTLGAVTEQASGVYTATLTAGTVAGVASLSVSVGGSALGVAPATVTLNGDS  1037
Yfr2   ------TFTLKSTN-------------------------TGSFSVTAKS------AVNTTDIG-----------------  706
Yfr3   ------DVMLSAVTEN-----------------------NGVYAATLKG-------------------------------  643
Yps2   ------DVTITPQVDTLILS------------------------------------------------------------  783
Ype5   ------AEITANNFTVEVDG------------------------------------------------------------  798
Pru1   ------VDAINPTLRFDNIQKRLTYSSITDNGQVIKGLPAG---AAPVWSSDNTSIATVDNQGKVKLKKAGRTKIWARID  1917
Pal3   --------------------------------------------------------------------------------  542
Eta1   ------GGTTTPELGFATAQHNVTWTKNFSDSQAVSGVPEG---VEQQWSSSDNSVATVNEVGKVTLLKSGQTTITVKTS  928
Sgl1   ------------------------------------QVTKT---VKTTFVSDDKSAMVA----------------ELKVV  738
Eco26  ------VGTINTNLSFDNKTINETYKGRAVLIQQVIGLPESNHPPVTWATSDPLVAEIDSRTGYITMKKAGMVTITASMP  1192
Pmi1   ------VAIPKSEITFEKPIQQEIYKSTVIDALSYKGVPQN--MQVIWSSSDPTVASIDTTSGQISMKKAGTTIITLQTL  1885
Eco1   --------------------------------------------GYSWSSDNPDIASIDANTGIITLNKKGTTVIKVISG  826
Ymo1   ------VGNIIPQLSFDNKNEKQTYRKTPFAKQALKGLP--QSVIAHWNSDNSDVAKIDPATGEIKLLKAGVVNISAVTL  955
Yen2   ------IGNIIPQLSFAKSKEATTYSRKVHKPLSLTGLPSSATLTAHWSSDNSDVATVNPLNGELTLLKAGVVNISVLTL  2023
Ybe1   -------------------------------------------NTLTVDSASADTNTNKTTGGEQGKVTGSTVVTTLETV  710
Esa2   ------VNDVAVTMTTNNSP--ADGSTANVAQALVTSASGQPMPGVSVTWNVGTATATTP-LTVTTNASGVATVSLTDTT  800
Sen3   ------ATAKVSDITVDKSQGVANGNDHITYTAIVTDASGNPVKDQEVTWNATPATANLSSATSTTDSNGNTSVTITTLK  1046
Eco14  ------TQQLYLTLQAGRTTAVADGNDAITYTLNVRDAAGQPVKDKAVQWSTDLG--TLSPLQGTTSAQGEASVTLTSTQ  780
Bpe1   ---------------------------------------------------------AAPTITNVTTDAASGRITVTGMA  949
Bav2   PGGQSVNVTAAADGSYTASSTRDVTQSGDITATARDAAGNVSAPATQAYNDIVDKTPPPVSITNVSTAPGSGIVTVTGVS  1030
Bbr1   --------LLLSPGSMTYTEIAKSFDGSSLDGIVARFEPANGAPPQT----AALLAAIKLHDPNYRLESNKMFIYLDTMN  893
Bpa2   PDQTRVTVVADTSGAYSATSAADMPDG-AITAVAADRAGNTGAPTRQRYIDSVDRTAPTLGTPSVSTAADTGRVSVTGMT  1206
Bav1   --------------------------------------------------------------------------------  747
Pma1   --------------------------------------------------------------------------------  372
Pma5   --------------------------------------------------------------------------------  373
Pma3   --------------------------------------------------------------------------------  379
Pma4   --------------------------------------------------------------------------------  410
Ssp1   --------------------------------------------------------------------------------  428
Ssp2   --------------------------------------------------------------------------------  436
Eco20  ---EMSSANSTLVADNKAPTVKMTTELTFTVKDAYGNPVTGLKPDAPVFSGAASTGSERPSAGNWTEKGNGVYVATLTLG  963
Efe5   --------------------------------ASLSLTPENSSATANIPANGKDAAVLNVQLTNNTNASVNGQKVELITS  809
Plu1   DKDEKRYNNGTDSYTFTATVVDAHGKPVADKPIDIDWQTNPKADGLTLTKQSNPVSNAQGQVTAILTSTAAVKDVQVSAK  1127
Pal2   VSTVGFTNSPSIDIDVIALKGAIHVHHVNLDATPKQFVVGEKIQLKLTLLDQFNNGVTDVLDNSININDDNTRSALNGLQ  1468
Ahy1   ------------------------------------------------------GATSVTYLPAREDQKKRVRVIVAPL-  916
Csu1   -------AKVSVVVPKKMLTKDTSITQGNSSYKPITATPSATDGTVSFTRITPTLPAGINLNSATGEITGTPTEESPLQV  858
Plu2   --------------------------------------------------------------------------------  302
Rba1   --------------------------------------------------------------------------------  327
Pas1   -------------------------------------LEQERLDQERVEQEARDAWLRQEEQERLEQERLEQERLERERL  822
Cla1   -----------------------------TLSNQELSFDQNGEAKALLTSKTPFKTPIKIVTSGVGKNSNKVITYDLRTF  1117
ruler  ......3130......3140......3150......3160......3170......3180......3190......3200
```

```
  Mba1 --------------------------------------------------------------------------------   331
  Psp2 --------------------------------------------------------------------------------   251
  Sen2 --------------------------------------------------------------------------------   660
  Efe4 --------------------------------------------------------------------------------   672
  Eco3 --------------------------------------------------------------------------------   417
  Cko1 --------------------------------------------------------------------------------   454
  Sen1 --------------------------------------------------------------------------------   460
  Eca1 --------------------------------------------------------------------------------   468
  Esp2 --------------------------------------------------------------------------------   468
  Esa3 --------------------------------------------------------------------------------   471
  Kpn1 --------------------------------------------------------------------------------   460
  Pan1 --------------------------------------------------------------------------------   490
  Eta2 --------------------------------------------------------------------------------   480
  Spr1 --------------------------------------------------------------------------------   497
  Yin1 --------------------------------------------------------------------------------   751
  Yin2 --------------------------------------------------------------------------------   753
  Yfr4 --------------------------------------------------------------------------------   749
  Ymo2 --------------------------------------------------------------------------------   686
 Eco10 --------------------------------------------------------------------------------   734
  Eal1 --------------------------------------------------------------------------------   725
  Sty4 --------------------------------------------------------------------------------   626
 Eco16 --------------------------------------------------------------------------------   711
 Eco25 DNNGFPVKGVTVNFTSNA---------ATAEMTNGGQAVTNEQGKATVTYTNTRSS------------------------  1625
 Eco15 DAYQNPLKDQLVTFQSND---------VTLSET---EVTTNTLGQATVTMTSN---------------------------  1069
  Eco6 DAFGNTLAGQTVSVMADNGATTAPTVITEPDGTVEISVTSQTAGTSTVTASINNSSQSRNVTFIADVRTAKIADLVVTRD  1651
  Efe2 IVG-----------------------------------------------------------------------------   911
  Efe3 QAGNNMLISVKLADSRGNALSGREAILNDAVAVGSATRKEGSVWTEDTQHKGTYTA------------------------  1249
  Yfr1 --------------------------------------------------------------------------------   770
  Yfr5 --------------------------------------------------------------------------------   780
  Yps4 NVVATIGSITNNIDTAFVAGAVATITLTTPVNGAVADGANSNSVQAVVTDSGGNPVNG----------------------  1172
  Yps7 -----------------GNLSTTHSTLVAAPVSIEANGSDTSLVTLTLRDSNNNPVTG----------------------  1078
  Yfr2 --------------------QTQNATFIPVFTGIIAGGYDFALN-------DGFPKT-----------------------   736
  Yfr3 ---------------------TVAGTVTVAPT---VNGSAIVGKS-----------------------------------   664
  Yps2 -----------------TLQKKISLFPVPTLTGILVNGQNFATDKG----------------------------------   812
  Ype5 ---------------------QVAGSGTNQVQALVVDKKGNPVANMTVN-------------------------------   826
  Pru1 GNGVYKSAAANYELEVEKARPNLTLTSNSTISATWADNNNHPIQSAFNHTDAA---------------------------  1970
  Pal3 --------------------------------------------------------------------------------   542
  Eta1 GNDQYDPAEASYQLKIDKADPQLQAGDGEPITAEWADGKVWSIASKFGNIDAE---------------------------   981
  Sgl1 SDGATADGKAENQLKVSVTDANGNVIEDKPVTLQAEGGVVLSTNSVTTDKDGT---------------------------   791
 Eco26 GNDKYSPGIASYNLIISKANPQISSDGIINAVWGDKSEKIISVSFNNEDVASN---------------------------  1245
  Pmi1 GNEQYPSAKNSYPLVIEKAPPKLKVTSPHTIQSIWNDGITHQITAEFDNPEVK---------------------------  1938
  Eco1 DKQIATYTIKTPQEIVSLDNSVKVKYDEASGICSNNAAYLSVSVDSLKKLYSQ---------------------------   879
  Ymo1 ADNTYAMGTASYQLEVERADPKLN-FTKRFSNVTWGQVAAKEQPNVGNSDWAN---------------------------  1007
  Yen2 PTDTYTSGTANYQLTVEKADPGIN-FAVAKRDVKWMDSMSPQNFVLSNSDANQ---------------------------  2075
  Ybe1 TDANSMKVGETINLTVTALNKNNLPVSGATIKVQAVSASSRNGQAETATLQVN---------------------------   763
  Esa2 AEAITVSATAGGKTGSAIAVFSVVPVSSVAVTMTTNN----SPADGSTANVAQ---------------------------   849
  Sen3 AGSVNVTAQAGASPAWNAPTTTFVGDKATAQILDLKTNKSTALANGTDSITFT---------------------------  1099
 Eco14 AGQAVVNATVDGK---QISAQSVTFTRTVRGVITVEK----ERVYPGTRQTVT---------------------------   826
  Bpe1 EPGANVAVNFP---------------------------------------------------------------------   960
  Bav2 EPGASVVVNFPGRGTARAEAAPDGGYTVSSTGPVESGAIQAVATDSHDNHSEPVR-------------------------  1085
  Bbr1 SD----------------------PYNRVPNGD-----------------------------------------------   904
  Bpa2 EPGARVQVEIPGEAPRIVTADAAGRYSVVSAGDVLQSGTVRVTATDAHGNASAPAHLR----------------------  1264
  Bav1 --------------------------------------------------------------------------------   747
  Pma1 --------------------------------------------------------------------------------   372
  Pma5 --------------------------------------------------------------------------------   373
  Pma3 --------------------------------------------------------------------------------   379
  Pma4 --------------------------------------------------------------------------------   410
  Ssp1 --------------------------------------------------------------------------------   428
  Ssp2 --------------------------------------------------------------------------------   436
 Eco20 SAAGQLSVMPRVNGQNAVAQPLVLNVAGDASKAEIRDMTVKVNNQLANGQSAN---------------------------  1016
  Efe5 AEGLSVPANIITDAAGRVSVPLTTVKAGEYTVTARVTDGSNSVESGSVKLTFVP--------------------------   863
  Plu1 AAANPSWVDADRKVSFEELSLSYQVTGVTVEVDKDKAHYNNGTDSYTFTATVVDGHG-----------------------  1184
  Pal2 WHHQQKGVYTAETTVTKGGIHSLKATVNSKQDNVLIHAISSVGQKQVSDVQLSTSTASIVAGTPATLILKLNDQHSNPVS  1548
  Ahy1 --------------------------------------------------------------------------------   916
  Csu1 YTMTVRDGKSGVESEVDFELGVAPSFVAQQKTYQKVIGVNSPVSFPICILSGG---------------------------   911
  Plu2 --------------------------------------------------------------------------------   302
  Rba1 --------------------------------------------------------------------------------   327
  Pas1 EQERLEQERLEQERRDKILERRKGTYGDSDDDSDDDTYQESPETTKEPEKKPE---------------------------   875
  Cla1 TPVITYPVFDSTLYGKHEKTIDYDTDYSILVKGLLPDSIIEPITNGVIKPKSN---------------------------  1170
 ruler ......3210......3220......3230......3240......3250......3260......3270......3280
```

```
  Mba1 --------------------------------------------------------------------------------  331
  Psp2 --------------------------------------------------------------------------------  251
  Sen2 --------------------------------------------------------------------------------  660
  Efe4 --------------------------------------------------------------------------------  672
  Eco3 --------------------------------------------------------------------------------  417
  Cko1 --------------------------------------------------------------------------------  454
  Sen1 --------------------------------------------------------------------------------  460
  Eca1 --------------------------------------------------------------------------------  468
  Esp2 --------------------------------------------------------------------------------  468
  Esa3 --------------------------------------------------------------------------------  471
  Kpn1 --------------------------------------------------------------------------------  460
  Pan1 --------------------------------------------------------------------------------  490
  Eta2 --------------------------------------------------------------------------------  480
  Spr1 --------------------------------------------------------------------------------  497
  Yin1 --------------------------------------------------------------------------------  751
  Yin2 --------------------------------------------------------------------------------  753
  Yfr4 --------------------------------------------------------------------------------  749
  Ymo2 --------------------------------------------------------------------------------  686
 Eco10 --------------------------------------------------------------------------------  734
  Eal1 --------------------------------------------------------------------------------  725
  Sty4 --------------------------------------------------------------------------------  626
 Eco16 --------------------------------------------------------------------------------  711
 Eco25 --IESGARPDTVEASLENGS---------------------------------------------------STLSTSINV 1652
 Eco15 ----IAGQHNVVVSRKAQAS---------------------------------------------------DNKTFSLSV 1094
  Eco6 NSVADGAMANTLQVKVTDANGNTLAGQTVSVLADNSATTAPTVITEPDGMVEISVTSQTAGTSAVTASINNSSLSQSVKF 1731
  Efe2 --------VNNYNLRVTCGD---------------------------------------------------KTKDCDWVT  932
  Efe3 --YYMAQHAGQDSVKLTLDD---------------------------------------------------GAKSSDAYT 1276
  Yfr1 --------------------------------------------------------------------------------  770
  Yfr5 --------------------------------------------------------------------------------  780
  Yps4 ------------------------------------------------------------------------------AA 1174
  Yps7 ------------------------------------------------------------------------------QT 1080
  Yfr2 --------------------------------------------------------------------------------  736
  Yfr3 --------------------------------------------------------------------------------  664
  Yps2 --------------------------------------------------------------------------------  812
  Ype5 --------------------------------------------------------------------------------  826
  Pru1 --------------------------------------------------------------------------------  1970
  Pal3 --------------------------------------------------------------------------------  542
  Eta1 --------------------------------------------------------------------------------  981
  Sgl1 --------------------------------------------------------------------------------  791
 Eco26 --------------------------------------------------------------------------------  1245
  Pmi1 --------------------------------------------------------------------------------  1938
  Eco1 --------------------------------------------------------------------------------  879
  Ymo1 --------------------------------------------------------------------------------  1007
  Yen2 --------------------------------------------------------------------------------  2075
  Ybe1 --------------------------------------------------------------------------------  763
  Esa2 --------------------------------------------------------------------------------  849
  Sen3 --------------------------------------------------------------------------------  1099
 Eco14 --------------------------------------------------------------------------------  826
  Bpe1 --------------------------------------------------------------------------------  960
  Bav2 --------------------------------------------------------------------------------  1085
  Bbr1 --------------------------------------------------------------------------------  904
  Bpa2 --------------------------------------------------------------------------------  1264
  Bav1 --------------------------------------------------------------------------------  747
  Pma1 --------------------------------------------------------------------------------  372
  Pma5 --------------------------------------------------------------------------------  373
  Pma3 --------------------------------------------------------------------------------  379
  Pma4 --------------------------------------------------------------------------------  410
  Ssp1 --------------------------------------------------------------------------------  428
  Ssp2 --------------------------------------------------------------------------------  436
 Eco20 --------------------------------------------------------------------------------  1016
  Efe5 --------------------------------------------------------------------------------  863
  Plu1 --------------------------------------------------------------------------------  1184
  Pal2 QVNSADISLENSAGTPLPTTWLEMNNLGIYTAQISLNSVGKQTVTVKVNNISHNATLQVTPPQGASSVATLEISPITTVD 1628
  Ahy1 --------------------------------------------------------------------------------  916
  Csu1 --------------------------------------------------------------------------------  911
  Plu2 --------------------------------------------------------------------------------  302
  Rba1 --------------------------------------------------------------------------------  327
  Pas1 --------------------------------------------------------------------------------  875
  Cla1 --------------------------------------------------------------------------------  1170
 ruler ......3290......3300......3310......3320......3330......3340......3350......3360
```

CLUSTAL X (1.81.1-alpha) MULTIPLE SEQUENCE ALIGNMENT

File: /Users/saierlab/Desktop/69long.ps **Date: Tue May 12 18:31:19 2009**


```
   Mba1 --------------------------------------------------------------------------------  331
   Psp2 --------------------------------------------------------------------------------  251
   Sen2 --------------------------------------------------------------------------------  660
   Efe4 --------------------------------------------------------------------------------  672
   Eco3 --------------------------------------------------------------------------------  417
   Cko1 --------------------------------------------------------------------------------  454
   Sen1 --------------------------------------------------------------------------------  460
   Eca1 --------------------------------------------------------------------------------  468
   Esp2 --------------------------------------------------------------------------------  468
   Esa3 --------------------------------------------------------------------------------  471
   Kpn1 --------------------------------------------------------------------------------  460
   Pan1 --------------------------------------------------------------------------------  490
   Eta2 --------------------------------------------------------------------------------  480
   Spr1 --------------------------------------------------------------------------------  497
   Yin1 --------------------------------------------------------------------------------  751
   Yin2 --------------------------------------------------------------------------------  753
   Yfr4 --------------------------------------------------------------------------------  749
   Ymo2 --------------------------------------------------------------------------------  686
  Eco10 --------------------------------------------------------------------------------  734
   Eal1 --------------------------------------------------------------------------------  725
   Sty4 --------------------------------------------------------------------------------  626
  Eco16 --------------------------------------------------------------------------------  711
  Eco25 NADASTAHLTLLQALFDTVSAG--DTTNLYIEVKDNYGNGVPQQEVTLSVSPSEGVTPSNNAIYTTNHDGNFYASFTATK 1730
  Eco15 LPDESSAKVISITGAEKTITVG--ENITLRILVQDAFNNVIAGQRVRLSAQPTTNITIGDTAY--TDNNGYAYVNLLSTQ 1170
   Eco6 IADVSTAQIAMLEVTQDNAVADGAMANTLQVKVTDAFGNALSGQTVSVLAG--NGATVAPTVI--TEPDGTAEIPVTSQT 1807
   Efe2 VNKQS----DSAIVK--------------------FISNPTSGSK------VRITAIPTVEG------------------  964
   Efe3 IVAAAPVDKNSAIQRGEAATYTAGDTLKLTVTLQDNWGNPVSGMENVLEGSVTLPEAEIQESGWQQTSPGVYEANWTARI 1356
   Yfr1 --------------------------------------------------------------------------------  770
   Yfr5 --------------------------------------------------------------------------------  780
   Yps4 VVFSSANATAQITTVIGTTGADGIATATLTNTVAGTSNVVATVDTVNANIDTTFVAGAVATITLTTPVNGAVADGADSNS 1254
   Yps7 VALVSTLGTLGAVTEQ----------------ASGVYTATLTAGTVAGVASLSVSVGGSA--------LGVAPATVTLNG 1136
   Yfr2 ----------------------------------GFNQATFTIN-MNGTADYSWSSN--------------QPAAVTVNS  767
   Yfr3 ------------------------------------HEVILTANHSTANVDLTVIID------------DAFANGVDVNK  696
   Yps2 -----------------------------------FPKTIFKNATFQLQMDNDVANN---------------TQYEWSSS  842
   Ype5 --------------------------------FTATNGVVVETTSAKTDENGKVTTN-----------LSMTNVGGTIST  863
   Pru1 --------------------------------SIPVEYISQDASIAQIDSASGAITQVKPGVTKLTIRSKETEQFLSESQ 2018
   Pal3 --------------------------------------------------------------------------------  542
   Eta1 --------------------------------NSLTATYTSENTGIVTVAGNGELQAVKPGSTRLTVSTPETDQFKPASV 1029
   Sgl1 --------------------------------ATFNAMSTTSGTFKVTATTNG---------------------------  812
  Eco26 --------------------------------IKPPVSFTSKDPSVVSIDNTGKIKMVKPGSTLIEVRSEETEQFLQSSA 1293
   Pmi1 --------------------------------NTP-IHFSSSDTLVATIDSKGNLTAIKPGKTKIVIQSDATDKFLADSQ 1985
   Eco1 --------------------------------WGPANKYSHYT-------------------------------------  890
   Ymo1 --------------------------------SQPQLKWSSSEPNVAKVDANGNITLLKAGDTSIKATTAQTDQFKAAEA 1055
   Yen2 --------------------------------SDIKTIWQTDSGKIATVDKGGLVTLVKPGTTNVTVSFVGDERFKYGEA 2123
   Ybe1 --------------------------------GKPLTDDVTTDGDGKVQVALTDPTGIGLKTTLTSSTNGSSGTPVSKNN  811
   Esa2 --------------------------------ALVTSASGQPMAGVSVTWNVGTATATTPLTVTTDAS-GLATVSLTDTT  896
   Sen3 --------------------------------GTVIDANNNVLEGVNVAWAVTPTTGVLSTTTSASGSDGKASVSLTSSQ 1147
  Eco14 --------------------------------LTLTDAAGNPVSGERVDWHVD--SGNLWQTQGTTDAQGRTTTTWESTT  872
   Bpe1 ---------------------------------------------------------------------DGTRKTVVADG  971
   Bav2 ---------------------------RDYLDTTAPAAPVISEVTAGPTGLVTVEGRAEVGSTVTVTFPDGASKQVPVAD 1138
   Bbr1 -------------------------------------YPVTLVLEDKATG------------------------------  917
   Bpa2 --------------------------YQDQVDKTAPASPSLTVREDAASGRATVTGQAEPGAAVRVVFPNGEAQTVTAGS 1318
   Bav1 --------------------------------------------------------------------------------  747
   Pma1 --------------------------------------------------------------------------------  372
   Pma5 --------------------------------------------------------------------------------  373
   Pma3 --------------------------------------------------------------------------------  379
   Pma4 --------------------------------------------------------------------------------  410
   Ssp1 --------------------------------------------------------------------------------  428
   Ssp2 --------------------------------------------------------------------------------  436
  Eco20 -----------------------------QITLTVVDSYGNPLQGQEVTLTLPQGVTSKTGNTVTTNAAGKVDIELMSTV 1067
   Efe5 --------------------------------DAASAELTIGASKQQIVADGHENATVDIQMLDANNNAFAGDVNLTITP  911
   Plu1 -------------KPVADKPIDIDWQTEPTVEGLKLTKQNNSVSNAQGQVTATLTSTAAVENVQVSAKTASQKTAENANG 1251
   Pal2 AGHTSSITLIMKDKYGNPVNNVLNRDMTLEIDGVIQSIQLTELGDTGQYSGELPAQQKGQHAVKVTVNGQLASVNWTINN 1708
   Ahy1 --------------------------------------------------------------------------------  916
   Csu1 -----------------------------VPPYNVTITPNLPNGLQLDATTGEISGSAKATSNQTTYTVNVTDANLSPRS  962
   Plu2 --------------------------------------------------------------------------------  302
   Rba1 --------------------------------------------------------------------------------  327
   Pas1 ---------------------------------LQGGNGSKPDIAPNLLLDHPLLNQVGDNSTLYTNNTDPNEEGNETDW  922
   Cla1 -----------------------------------SVQVNNKGEATIAYHKISDFNTKKIKPQFKYIKSGDTKETFTMND 1215
  ruler ......3370......3380......3390......3400......3410......3420......3430......3440
```

```
   Mba1 --------------------------------------------------------------------------------  331
   Psp2 --------------------------------------------------------------------------------  251
   Sen2 --------------------------------------------------------------------------------  660
   Efe4 --------------------------------------------------------------------------------  672
   Eco3 --------------------------------------------------------------------------------  417
   Cko1 --------------------------------------------------------------------------------  454
   Sen1 --------------------------------------------------------------------------------  460
   Eca1 --------------------------------------------------------------------------------  468
   Esp2 --------------------------------------------------------------------------------  468
   Esa3 --------------------------------------------------------------------------------  471
   Kpn1 --------------------------------------------------------------------------------  460
   Pan1 --------------------------------------------------------------------------------  490
   Eta2 --------------------------------------------------------------------------------  480
   Spr1 --------------------------------------------------------------------------------  497
   Yin1 --------------------------------------------------------------------------------  751
   Yin2 --------------------------------------------------------------------------------  753
   Yfr4 --------------------------------------------------------------------------------  749
   Ymo2 --------------------------------------------------------------------------------  686
  Eco10 --------------------------------------------------------------------------------  734
   Eal1 --------------------------------------------------------------------------------  725
   Sty4 --------------------------------------------------------------------------------  626
  Eco16 --------------------------------------------------------------------------------  711
  Eco25 AGVYQVTATLENGDSMQQTVTYVPNVANAEISLAASKDPVIANNNDLTTLTATVADTEGNAIANSEVTFTLPEDVRANFT 1810
  Eco15 PGVYQVTATLDN-------------------------------------------------------------------- 1182
   Eco6 AGVSAVTATINNSSQSRNVTFVADVRTAQIADLVVIKDGSEADGATANTLRARVTDAFGNALAGQTVS------VLADNG 1881
   Efe2 -ALTLMYEFQIRKWFKPDS------------------------------------NSIGNPVG-----------------  990
   Efe3 AGPSLTAKLELEGWRKETAPFVIVAAGPDYENSSLRTSNFAFTAGDDITITVILMDANENPVTGAEGL------------ 1424
   Yfr1 --------------------------------------------------------------------------------  770
   Yfr5 --------------------------------------------------------------------------------  780
   Yps4 VQAVVSDSGGNPVAGAAVVFSSANATAQVTTVIGTTGADGIATATLTNTVAGTSNVVATIGSITNNID------------ 1322
   Yps7 DSGNLSTTHSTLVA------------------------------------------------------------------ 1150
   Yfr2 SG------------------------------------------------------------------------------  769
   Yfr3 IKAH----------------------------------------------------------------------------  700
   Yps2 FTPN----------------------------------------------------------------------------  846
   Ype5 VTATMIN-------------------------------------------------------------------------  870
   Pru1 EVTYNLAKARFNIDFAQ--------------------------------------------------------------- 2035
   Pal3 --------------------------------------------------------------------------------  542
   Eta1 EVAYQLDKRTVDVSFKA--------------------------------------------------------------- 1046
   Sgl1 ---YSKTASVVFVAGGV---------------------------------------------------------------  826
  Eco26 IVTYVLDKGRRNITFKN--------------------------------------------------------------- 1310
   Pmi1 TVDYQQEKAALSYYFKKNY------------------------------------------------------------- 2004
   Eco1 --------------------------------------------------------------------------------  890
   Ymo1 EYVLNINKANLQLSFNAS-------------------------------------------------------------- 1073
   Yen2 SYELNVAKYKPTVSFANS-------------------------------------------------------------- 2141
   Ybe1 DVIFTVFTSPDSPVADYWG-------------------------------------------------------------  830
   Esa2 AETVAVIASAGGKS------------------------------------------------------------------  910
   Sen3 VESYQVTATVNGKDETSGNVSFTADSASASLTSLTSDETSNLIAGNGSATLTAVVQDATGHPIAGAVV------------ 1215
  Eco14 PGTATITADAYGQQYTAP--------------------------------------------------------------  890
   Bpe1 AGAYAADSDGDMVAGDIH--------------------------------------------------------------  989
   Bav2 AGTYRVTSDANQPSGDIK-------------------------------------------------------------- 1156
   Bbr1 -------------AREAT--------------------------------------------------------------  922
   Bpa2 DGAYSVTSAADMVAGEIT-------------------------------------------------------------- 1336
   Bav1 --------------------------------------------------------------------------------  747
   Pma1 --------------------------------------------------------------------------------  372
   Pma5 --------------------------------------------------------------------------------  373
   Pma3 --------------------------------------------------------------------------------  379
   Pma4 --------------------------------------------------------------------------------  410
   Ssp1 --------------------------------------------------------------------------------  428
   Ssp2 --------------------------------------------------------------------------------  436
  Eco20 AGEHSITASVNNAQKTVT-------------------------------------------------------------- 1085
   Efe5 STGASLTSSKLQLDARG---------------------------------------------------------------  928
   Plu1 KVSFTEFSASYYVASVTVEVDKDE-------------------------------------------------------- 1275
   Pal2 PMPIPLSTVDRTGQRGALDTIMLSGSKKMVDSGDKITVTLNLMDKFNNPLTGANSHLKLLTNLSEISQWQDHS------- 1781
   Ahy1 --------------------------------------------------------------------------------  916
   Csu1 PMTLALTVANAPTVTEVES-------------------------------------------------------------  981
   Plu2 --------------------------------------------------------------------------------  302
   Rba1 --------------------------------------------------------------------------------  327
   Pas1 ED------------------------------------------------------------------------------  924
   Cla1 INLYQYKLSISSDKTELVG------------------------------------------------------------- 1234
  ruler ......3450......3460......3470......3480......3490......3500......3510......3520
```

```
  Mba1 --------------------------------------------------------------------------------  331
  Psp2 --------------------------------------------------------------------------------  251
  Sen2 --------------------------------------------------------------------------------  660
  Efe4 --------------------------------------------------------------------------------  672
  Eco3 --------------------------------------------------------------------------------  417
  Cko1 --------------------------------------------------------------------------------  454
  Sen1 --------------------------------------------------------------------------------  460
  Eca1 --------------------------------------------------------------------------------  468
  Esp2 --------------------------------------------------------------------------------  468
  Esa3 --------------------------------------------------------------------------------  471
  Kpn1 --------------------------------------------------------------------------------  460
  Pan1 --------------------------------------------------------------------------------  490
  Eta2 --------------------------------------------------------------------------------  480
  Spr1 --------------------------------------------------------------------------------  497
  Yin1 --------------------------------------------------------------------------------  751
  Yin2 --------------------------------------------------------------------------------  753
  Yfr4 --------------------------------------------------------------------------------  749
  Ymo2 --------------------------------------------------------------------------------  686
 Eco10 --------------------------------------------------------------------------------  734
  Eal1 --------------------------------------------------------------------------------  725
  Sty4 --------------------------------------------------------------------------------  626
 Eco16 --------------------------------------------------------------------------------  711
 Eco25 LGDGGKVVTDTEGKAKVTLKGTKAGAHTVTASMAGGKSEQLVVNFIADTLTAQVNLNVTEDNFIANNVGMTRLQATVTDG 1890
 Eco15 -------------------------------------------------------------------------------- 1182
  Eco6 ATVAPTVTTQPDGTVEISVTSQTAGISAVTATINNSTASQNVTFIADVRTAQIADLVVIKDGSEADGAMANMLRLKITDA 1961
  Efe2 --------------------------------------------------------------------------------  990
  Efe3 -------------------------------------------------------------------------------- 1424
  Yfr1 --------------------------------------------------------------------------------  770
  Yfr5 --------------------------------------------------------------------------------  780
  Yps4 -------------------------------------------------------------------------------- 1322
  Yps7 -------------------------------------------------------------------------------- 1150
  Yfr2 --------------------------------------------------------------------------------  769
  Yfr3 --------------------------------------------------------------------------------  700
  Yps2 --------------------------------------------------------------------------------  846
  Ype5 --------------------------------------------------------------------------------  870
  Pru1 -------------------------------------------------------------------------------- 2035
  Pal3 --------------------------------------------------------------------------------  542
  Eta1 -------------------------------------------------------------------------------- 1046
  Sgl1 --------------------------------------------------------------------------------  826
 Eco26 -------------------------------------------------------------------------------- 1310
  Pmi1 -------------------------------------------------------------------------------- 2004
  Eco1 --------------------------------------------------------------------------------  890
  Ymo1 -------------------------------------------------------------------------------- 1073
  Yen2 -------------------------------------------------------------------------------- 2141
  Ybe1 --------------------------------------------------------------------------------  830
  Esa2 --------------------------------------------------------------------------------  910
  Sen3 -------------------------------------------------------------------------------- 1215
 Eco14 --------------------------------------------------------------------------------  890
  Bpe1 --------------------------------------------------------------------------------  989
  Bav2 -------------------------------------------------------------------------------- 1156
  Bbr1 --------------------------------------------------------------------------------  922
  Bpa2 -------------------------------------------------------------------------------- 1336
  Bav1 --------------------------------------------------------------------------------  747
  Pma1 --------------------------------------------------------------------------------  372
  Pma5 --------------------------------------------------------------------------------  373
  Pma3 --------------------------------------------------------------------------------  379
  Pma4 --------------------------------------------------------------------------------  410
  Ssp1 --------------------------------------------------------------------------------  428
  Ssp2 --------------------------------------------------------------------------------  436
 Eco20 -------------------------------------------------------------------------------- 1085
  Efe5 --------------------------------------------------------------------------------  928
  Plu1 -------------------------------------------------------------------------------- 1275
  Pal2 -------------------------------------------------------------------------------- 1781
  Ahy1 --------------------------------------------------------------------------------  916
  Csu1 --------------------------------------------------------------------------------  981
  Plu2 --------------------------------------------------------------------------------  302
  Rba1 --------------------------------------------------------------------------------  327
  Pas1 --------------------------------------------------------------------------------  924
  Cla1 -------------------------------------------------------------------------------- 1234
 ruler ......3530......3540......3550......3560......3570......3580......3590......3600
```

```
  Mba1 --------------------------------------------------------------------------------  331
  Psp2 --------------------------------------------------------------------------------  251
  Sen2 --------------------------------------------------------------------------------  660
  Efe4 --------------------------------------------------------------------------------  672
  Eco3 --------------------------------------------------------------------------------  417
  Cko1 --------------------------------------------------------------------------------  454
  Sen1 --------------------------------------------------------------------------------  460
  Eca1 --------------------------------------------------------------------------------  468
  Esp2 --------------------------------------------------------------------------------  468
  Esa3 --------------------------------------------------------------------------------  471
  Kpn1 --------------------------------------------------------------------------------  460
  Pan1 --------------------------------------------------------------------------------  490
  Eta2 --------------------------------------------------------------------------------  480
  Spr1 --------------------------------------------------------------------------------  497
  Yin1 --------------------------------------------------------------------------------  751
  Yin2 --------------------------------------------------------------------------------  753
  Yfr4 --------------------------------------------------------------------------------  749
  Ymo2 --------------------------------------------------------------------------------  686
 Eco10 --------------------------------------------------------------------------------  734
  Eal1 --------------------------------------------------------------------------------  725
  Sty4 --------------------------------------------------------------------------------  626
 Eco16 --------------------------------------------------------------------------------  711
 Eco25 NGNPLANEAVTFTLPADVSASFTLGQGGSAITDINGKAEVTLSGTKSGTYPVTVSVNNYGVSDTKQVTLIADAGTAKLAS 1970
 Eco15 -------------------------------------------------------------------------------- 1182
  Eco6 FGNTLAGQTVSVLAGN--------GATTAPTVTTQPDGTVEISVTSQTAGVSAVTATINSSTQSQNVTFIADVRTAQIAE 2033
  Efe2 --------------------------------------------------------------------------------  990
  Efe3 -------------------------------------------------------------------------------- 1424
  Yfr1 --------------------------------------------------------------------------------  770
  Yfr5 --------------------------------------------------------------------------------  780
  Yps4 -------------------------------------------------------------------------------- 1322
  Yps7 -------------------------------------------------------------------------------- 1150
  Yfr2 --------------------------------------------------------------------------------  769
  Yfr3 --------------------------------------------------------------------------------  700
  Yps2 --------------------------------------------------------------------------------  846
  Ype5 --------------------------------------------------------------------------------  870
  Pru1 -------------------------------------------------------------------------------- 2035
  Pal3 --------------------------------------------------------------------------------  542
  Eta1 -------------------------------------------------------------------------------- 1046
  Sgl1 --------------------------------------------------------------------------------  826
 Eco26 -------------------------------------------------------------------------------- 1310
  Pmi1 -------------------------------------------------------------------------------- 2004
  Eco1 --------------------------------------------------------------------------------  890
  Ymo1 -------------------------------------------------------------------------------- 1073
  Yen2 -------------------------------------------------------------------------------- 2141
  Ybe1 --------------------------------------------------------------------------------  830
  Esa2 --------------------------------------------------------------------------------  910
  Sen3 -------------------------------------------------------------------------------- 1215
 Eco14 --------------------------------------------------------------------------------  890
  Bpe1 --------------------------------------------------------------------------------  989
  Bav2 -------------------------------------------------------------------------------- 1156
  Bbr1 --------------------------------------------------------------------------------  922
  Bpa2 -------------------------------------------------------------------------------- 1336
  Bav1 --------------------------------------------------------------------------------  747
  Pma1 --------------------------------------------------------------------------------  372
  Pma5 --------------------------------------------------------------------------------  373
  Pma3 --------------------------------------------------------------------------------  379
  Pma4 --------------------------------------------------------------------------------  410
  Ssp1 --------------------------------------------------------------------------------  428
  Ssp2 --------------------------------------------------------------------------------  436
 Eco20 -------------------------------------------------------------------------------- 1085
  Efe5 --------------------------------------------------------------------------------  928
  Plu1 -------------------------------------------------------------------------------- 1275
  Pal2 -------------------------------------------------------------------------------- 1781
  Ahy1 --------------------------------------------------------------------------------  916
  Csu1 --------------------------------------------------------------------------------  981
  Plu2 --------------------------------------------------------------------------------  302
  Rba1 --------------------------------------------------------------------------------  327
  Pas1 --------------------------------------------------------------------------------  924
  Cla1 -------------------------------------------------------------------------------- 1234
 ruler ......3610......3620......3630......3640......3650......3660......3670......3680
```

```
Mba1   --------------------------------------------------------------------------------   331
Psp2   --------------------------------------------------------------------------------   251
Sen2   --------------------------------------------------------------------------------   660
Efe4   --------------------------------------------------------------------------------   672
Eco3   --------------------------------------------------------------------------------   417
Cko1   --------------------------------------------------------------------------------   454
Sen1   --------------------------------------------------------------------------------   460
Eca1   --------------------------------------------------------------------------------   468
Esp2   --------------------------------------------------------------------------------   468
Esa3   --------------------------------------------------------------------------------   471
Kpn1   --------------------------------------------------------------------------------   460
Pan1   --------------------------------------------------------------------------------   490
Eta2   --------------------------------------------------------------------------------   480
Spr1   --------------------------------------------------------------------------------   497
Yin1   --------------------------------------------------------------------------------   751
Yin2   --------------------------------------------------------------------------------   753
Yfr4   --------------------------------------------------------------------------------   749
Ymo2   --------------------------------------------------------------------------------   686
Eco10  --------------------------------------------------------------------------------   734
Eal1   --------------------------------------------------------------------------------   725
Sty4   --------------------------------------------------------------------------------   626
Eco16  --------------------------------------------------------------------------------   711
Eco25  LTSVYSFVVSTTEGA-TMTASVTDANGNPVEGIKVNFRGTSVTLSSTSVETDDRGFAEILVTSTEVGLKTVSASLADKPT  2049
Eco15  --------------------------------------------------------------------------------  1182
Eco6   LVVIKDGAVADGAMANMLQVKVTDAFGNALAGQTVSVTAGNSATVASTVTTKPDGTVEISVTSQTAGTSTVTASINNSSQ  2113
Efe2   -----------------------------LVDSCITANGKVPEKSELNNEAN----------------------------  1013
Efe3   -----------------------------LTNETVKVPGATRKGTVWTDKGDGTYRAEWEAEQAGNGYKATLHLAGWGDE  1475
Yfr1   --------------------------------------------------------------------------------   770
Yfr5   --------------------------------------------------------------------------------   780
Yps4   --------------------------------------------------------------------------------  1322
Yps7   --------------------------------------------------------------------------------  1150
Yfr2   --------------------------------------------------------------------------------   769
Yfr3   --------------------------------------------------------------------------------   700
Yps2   --------------------------------------------------------------------------------   846
Ype5   --------------------------------------------------------------------------------   870
Pru1   --------------------------------------------------------------------------------  2035
Pal3   --------------------------------------------------------------------------------   542
Eta1   --------------------------------------------------------------------------------  1046
Sgl1   --------------------------------------------------------------------------------   826
Eco26  --------------------------------------------------------------------------------  1310
Pmi1   --------------------------------------------------------------------------------  2004
Eco1   --------------------------------------------------------------------------------   890
Ymo1   --------------------------------------------------------------------------------  1073
Yen2   --------------------------------------------------------------------------------  2141
Ybe1   --------------------------------------------------------------------------------   830
Esa2   --------------------------------------------------------------------------------   910
Sen3   --------------------------------------------------------------------------------  1215
Eco14  --------------------------------------------------------------------------------   890
Bpe1   --------------------------------------------------------------------------------   989
Bav2   --------------------------------------------------------------------------------  1156
Bbr1   --------------------------------------------------------------------------------   922
Bpa2   --------------------------------------------------------------------------------  1336
Bav1   --------------------------------------------------------------------------------   747
Pma1   --------------------------------------------------------------------------------   372
Pma5   --------------------------------------------------------------------------------   373
Pma3   --------------------------------------------------------------------------------   379
Pma4   --------------------------------------------------------------------------------   410
Ssp1   --------------------------------------------------------------------------------   428
Ssp2   --------------------------------------------------------------------------------   436
Eco20  --------------------------------------------------------------------------------  1085
Efe5   --------------------------------------------------------------------------------   928
Plu1   --------------------------------------------------------------------------------  1275
Pal2   --------------------------------------------------------------------------------  1781
Ahy1   --------------------------------------------------------------------------------   916
Csu1   --------------------------------------------------------------------------------   981
Plu2   --------------------------------------------------------------------------------   302
Rba1   --------------------------------------------------------------------------------   327
Pas1   --------------------------------------------------------------------------------   924
Cla1   --------------------------------------------------------------------------------  1234
ruler  ......3690......3700......3710......3720......3730......3740......3750......3760
```

```
 Mba1 --------------------------------------------------------------------------------  331
 Psp2 --------------------------------------------------------------------------------  251
 Sen2 --------------------------------------------------------------------------------  660
 Efe4 --------------------------------------------------------------------------------  672
 Eco3 --------------------------------------------------------------------------------  417
 Cko1 --------------------------------------------------------------------------------  454
 Sen1 --------------------------------------------------------------------------------  460
 Eca1 --------------------------------------------------------------------------------  468
 Esp2 --------------------------------------------------------------------------------  468
 Esa3 --------------------------------------------------------------------------------  471
 Kpn1 --------------------------------------------------------------------------------  460
 Pan1 --------------------------------------------------------------------------------  490
 Eta2 --------------------------------------------------------------------------------  480
 Spr1 --------------------------------------------------------------------------------  497
 Yin1 --------------------------------------------------------------------------------  751
 Yin2 --------------------------------------------------------------------------------  753
 Yfr4 --------------------------------------------------------------------------------  749
 Ymo2 --------------------------------------------------------------------------------  686
Eco10 --------------------------------------------------------------------------------  734
 Eal1 --------------------------------------------------------------------------------  725
 Sty4 --------------------------------------------------------------------------------  626
Eco16 --------------------------------------------------------------------------------  711
Eco25 EVISRLLNAKADINSATITSLEIPEGQVMVAQDVAVKAHVNDQFGNPILNESVTFSAEPPEHMTISQNIVSTDTHGIAEV  2129
Eco15 --------------------------------------------------------------------------------  1182
 Eco6 SQNVTFVPCDASRLTSTVETNK---SNYTVGETITITVTLRDAFDNLVTGAASQLAADG----------VLTVAGTDPSE  2180
 Efe2 ---------------------------------IPKVGTLWAEWG-------------------------KTLVSEGLVS  1035
 Efe3 ERQSDPYNIQPGEPAEAQSGISTDGDTFVAGQFIPVTVTLKDAWGNHVPAQESYLA--------------THVVAAHTVP  1541
 Yfr1 --------------------------------------------------------------------------------  770
 Yfr5 --------------------------------------------------------------------------------  780
 Yps4 -------------------------------------------------TAFVAGAVATITLSVPVNDATADGVDTNQVD  1353
 Yps7 ----------------------------------------------------------------APVNIEANGSDTSLVT  1166
 Yfr2 -------------------------------------------------------------------QVTFNGPPSGTVT  782
 Yfr3 -------------------------------------------------------------------VIDLHGNPVEDVA  713
 Yps2 ----------------------------------------------------------------------VSVNDQGQVT  856
 Ype5 -------------------------------------------------------------------SANVTSTQDKPVI  883
 Pru1 ----------------------------------------------------------------------KEQEITDEKG  2045
 Pal3 --------------------------------------------------------------------------------  542
 Eta1 ----------------------------------------------------------------------AVIKTTDEEE  1056
 Sgl1 ----------------------------------------------------------------------VGVKST----  832
Eco26 ----------------------------------------------------------------------SDVKMTVDEK  1320
 Pmi1 ---------------------------------------------------------------------IVLTTDSAADK  2015
 Eco1 --------------------------------------------------------------------------------  890
 Ymo1 ----------------------------------------------------------------------VKQYSVDSKP  1083
 Yen2 ----------------------------------------------------------------------LLTNKVSEKI  2151
 Ybe1 ---------------------------------------------------------------------HMDETIIVDGV  841
 Esa2 ----------------------------------------------------------------------GNASATFTSV  920
 Sen3 -------------------------------------------------NWSSDNTTGNFSETTSTTNSEGKAIVTFSGT  1246
Eco14 ---------------------------------------------------------------------VITVMPALTVS  901
 Bpe1 -------------------------------------------------------------------VQATDKAGNRSPE  1002
 Bav2 -------------------------------------------------------------------ASATDKARNKSPE  1169
 Bbr1 -------------------------------------------------------------------TMVLKVTGSTYG-  934
 Bpa2 -------------------------------------------------------------------VVAADASGNQSAP  1349
 Bav1 --------------------------------------------------------------------------------  747
 Pma1 --------------------------------------------------------------------------------  372
 Pma5 --------------------------------------------------------------------------------  373
 Pma3 --------------------------------------------------------------------------------  379
 Pma4 --------------------------------------------------------------------------------  410
 Ssp1 --------------------------------------------------------------------------------  428
 Ssp2 --------------------------------------------------------------------------------  436
Eco20 ----------------------------------------------------------------------VKFKADFSTG  1095
 Efe5 ---------------------------------------------------------------------QATTQFTASKA  939
 Plu1 -------------------------------------------------------------------KHYNNGTDSYIFT  1288
 Pal2 ------------------------------DGSYSIDLLMNRLGSQDVQAIVKNILSNKVTLEIKALSGASNVNTTALTI  1831
 Ahy1 --------------------------------------------------------------------------------  916
 Csu1 ---------------------------------------------------------------------NKVAVIGSYVN  992
 Plu2 --------------------------------------------------------------------------------  302
 Rba1 --------------------------------------------------------------------------------  327
 Pas1 --------------------------------------------------------------------------------  924
 Cla1 ------------------------------------------------------------------------DEIFKVTV  1242
ruler ......3770......3780......3790......3800......3810......3820......3830......3840
```

```
Mba1   --------------------------------------------------------------------------------   331
Psp2   --------------------------------------------------------------------------------   251
Sen2   --------------------------------------------------------------------------------   660
Efe4   --------------------------------------------------------------------------------   672
Eco3   --------------------------------------------------------------------------------   417
Cko1   --------------------------------------------------------------------------------   454
Sen1   --------------------------------------------------------------------------------   460
Eca1   --------------------------------------------------------------------------------   468
Esp2   --------------------------------------------------------------------------------   468
Esa3   --------------------------------------------------------------------------------   471
Kpn1   --------------------------------------------------------------------------------   460
Pan1   --------------------------------------------------------------------------------   490
Eta2   --------------------------------------------------------------------------------   480
Spr1   --------------------------------------------------------------------------------   497
Yin1   --------------------------------------------------------------------------------   751
Yin2   --------------------------------------------------------------------------------   753
Yfr4   --------------------------------------------------------------------------------   749
Ymo2   --------------------------------------------------------------------------------   686
Eco10  --------------------------------------------------------------------------------   734
Eal1   --------------------------------------------------------------------------------   725
Sty4   --------------------------------------------------------------------------------   626
Eco16  --------------------------------------------------------------------------------   711
Eco25  TMTPERNGSYMVKASLANGSSYEKDLVVIDQKLTLSASSPLIGVNSPTGATLTATLTSANGTPVEGQVINFSVTPEG--A  2207
Eco15  ------NSSSKVDVNVANG------------KLELTSSKPETTVHNSEGITLTATARNARGELMPGQIITFSVTPEG--A  1242
Eco6   TGSWVESGGVYTTTRMATIASTNQHANLQLQTWSDGVTSDRYDIQSGSPAQATSTIATDKNAYTAGDTITVAVTLKDAHG  2260
Efe2   HPTIWTP---------------------SAQAASGWALLS----------IEN---------------------------  1057
Efe3   QPTQWQENSDGTYSAQFIAGEPGADLQVTLNQDSGWKVAATYSIIDAQPVIDNSFVDTDKDSYQAGDDMLITVKLADSRG  1621
Yfr1   --------------------------------------------------------------------------------   770
Yfr5   --------------------------------------------------------------------------------   780
Yps4   ALVQDANGNAITGAAVVFSSTNGADIIVPTMNTGVNGVASTLLTHTVAGTSNVVATVDTVNANIDTAFVPGAVATITLTT  1433
Yps7   LTLRDSNNNPVTGQTVALVST--LGTLGAVTEQASGIYTATLTAGTLTGTASLSVNVDGNSLGITPATVNVIPAPVDLTV  1244
Yfr2   ITATPNNGG---------------------SPQSYSFTVENWFEGDSTSRNWGNANADCNSR------SEVLPTIAQLTS   835
Yfr3   VKFEADNGATVITPEG--------------ITDTEGIAIVELAN-INPGVTTLNAIIDGHEERVETSFMPEIPLKLLIYR   778
Yps2   ITYQTYSEVAVTAKSK-------------KFPSYSVSYRFYPNRWIYDGGRSLVSSLEASRQCQGSDMSAVLESSR----   919
Ype5   FYPDFTKATLNTPANTYSGFNINSGFPTTGFKNTHFQLSPHGITGANSDYDWVSSHPNVSVSNTGAITLQDNPGGKVTIT   963
Pru1   HFALQNTQITVPSKADIVWTSGADSVVNLEKNGTLK-----DLAKGEANLIMTVKANDYFEQTSGEYRVKVYTKPAISAN  2120
Pal3   --------------------------------------------------------------------------------   542
Eta1   -FTLQLPENALPSDAIFTWESADKKVLDISSSGIVQG----KVSKGKTRLTLSITANDYYSASSNYYDMWIYSKPSVSIG  1131
Sgl1   ---LAADKSLIASN-------------------------------GSSAISLTFTAHDNHNN--AVTGLTVAFEPNGVEG   876
Eco26  -FSLQTPELNNITTSSKIWRSSNPKVIEINQDGSLPSNKETPNDAGYSEISLIIPSDEYYHEERSSYNLHVYGQPAINIG  1399
Pmi1   LLMAQSPTPDVPVEANATWYSSQPNIVEITPDGIIKN-----LNIGQTTLTLEVKNNNYFQDHEQSYNVIIQASPRITIN  2090
Eco1   --------------------------------------------QGTINAWIQQTEQDKKDSVATTYDIVTDNTVRNVNS   926
Ymo1   SNVQSLILPTDLPASAVRWSSSNPSAMNIDSNG-RVSGV----SAGDSNIKIEIDKNDFYAENSAKYLAEVYQKPSVSIS  1158
Yen2   YVQKPDEKLSTYAHLETKWSSSDNAIVEVANDASYMSPK----GPGKARITLQVVGNDWYEEQSSSYEQEVYATPKVSIR  2227
Ybe1   IIKRPKLAAEVAGSQQ--------------------------IDMNNEEWAYANFTNASAHCTLPTKEDLVALYNAYPTG   895
Esa2   SLKAVSVSLSSVGR-----------------------------GNAADPGITTAQVKDINGNPVAGATVIWSTPANGFIH   971
Sen3   HAQLTTITASSVNNSQKTVQVTIAPDTQSAQPVTVVADKHGAIANGADTVTLTATIQDQYGNFINQGDVAWTISPEASYH  1326
Eco14  SVTGIDATGADGKN-----------------------------FGKRVPNSTWPGAKFRIDTENAAGTVTWTASSPAVSI   952
Bpe1   GTRAYVDTVDKTPSAAPTIVRVTTDRSSGVVTVAGTADPDNDVTVQFPDGGRKTVKAGKDGSYSVTSDNDIFSGIIQVSA  1082
Bav2   ATQVYT---DQTAPAVPLISTVNTSPQTGVVTVEGTAEADSQVKVSFPDGTSKTVSADGSGHYTATSDHDQPSGEIKVQA  1246
Bbr1   -----------KAPVVP-----------GANGVLG-TGPGPSLGGSL-----------------------LIGGEGGLLG   968
Bpa2   ARTVFADAVDRTAPAAPTLA-LSEAADSGRLTVSGRTEPGASVRVTFPDGETVTVTAKADGTYTATSRADMIGGNVTVVA  1428
Bav1   --------------------------------------------------------------------------------   747
Pma1   --------------------------------------------------------------------------------   372
Pma5   --------------------------------------------------------------------------------   373
Pma3   --------------------------------------------------------------------------------   379
Pma4   --------------------------------------------------------------------------------   410
Ssp1   --------------------------------------------------------------------------------   428
Ssp2   --------------------------------------------------------------------------------   436
Eco20  QATLEVDGSTPKVANDNDAFTLTATVKDQYGNLLPGAVVVFNLPRGVKPLADGNIMVNADKEGKAELKVVSVTAGTYEIT  1175
Efe5   GKYTIQAEYMLNGKRITASQNIDAVTDVKDAVLEITSDVSSAVVSDTSNLKFTLQLKSKSGEALSGRVVKISTTGPSKNG  1019
Plu1   ATVKDGHGNLVVGQPVDIDWQTDPKADGLKLTKQNNSVSNAQGQVTATLTSTVVAIDVQVSAKTATQQTPVNVDKKVSFI  1368
Pal2   KDATIEAGDTTELTLRLKDLVDNGVTNIQNRDIHLTQKNAKIDKKWLSAMDGIYTTQVQIKQVGVYPLRATVNQQNSRIE  1911
Ahy1   --------------------------------------------------------------------------------   916
Csu1   YTPLKATSAVTGKTVTFISVAPALPSGLSWRSYDGSIYGTPQDVTKTGQETFTFTIRDGGTGAEVQHSFNLSVVPKFTFS  1072
Plu2   --------------------------------------------------------------------------------   302
Rba1   --------------------------------------------------------------------------------   327
Pas1   --------------------------------------------------------------------------------   924
Cla1   RGGKPNASVEWTLTGDGKITSKDSKFNAKGEAYLNGQGKSPFNNSINVEAKAFNQILKASAKISIYESRGILAHPTGNKN  1322
ruler  ......3850......3860......3870......3880......3890......3900......3910......3920
```

CLUSTAL X (1.81.1-alpha) MULTIPLE SEQUENCE ALIGNMENT


```
  Mba1 --------------------------------------------------------------------------------  331
  Psp2 --------------------------------------------------------------------------------  251
  Sen2 --------------------------------------------------------------------------------  660
  Efe4 --------------------------------------------------------------------------------  672
  Eco3 --------------------------------------------------------------------------------  417
  Cko1 --------------------------------------------------------------------------------  454
  Sen1 --------------------------------------------------------------------------------  460
  Eca1 --------------------------------------------------------------------------------  468
  Esp2 --------------------------------------------------------------------------------  468
  Esa3 --------------------------------------------------------------------------------  471
  Kpn1 --------------------------------------------------------------------------------  460
  Pan1 --------------------------------------------------------------------------------  490
  Eta2 --------------------------------------------------------------------------------  480
  Spr1 --------------------------------------------------------------------------------  497
  Yin1 --------------------------------------------------------------------------------  751
  Yin2 --------------------------------------------------------------------------------  753
  Yfr4 --------------------------------------------------------------------------------  749
  Ymo2 --------------------------------------------------------------------------------  686
 Eco10 --------------------------------------------------------------------------------  734
  Eal1 --------------------------------------------------------------------------------  725
  Sty4 --------------------------------------------------------------------------------  626
 Eco16 --------------------------------------------------------------------------------  711
 Eco25 TLS-GGKVRTNSS-----GQAPVVLTSNKVGTYTVTASFHNG------VTIQTQTIVKVTGNSSTAHVASFIADPSTIAA 2275
 Eco15 TLSNTGEVLTDQS-----GQAKVTLTSDKVNVYTVTAIMGKD------VPVQSQVTVAVKADAKTAHVVSVVASPDTITA 1311
  Eco6 NLVEGGESLLSGDNVTVEGAVRSGGRSETAGVYTATWSAQMAGDSHHATLKLSEWGSSKQSESYSIHSGAPVQANSAIRT 2340
  Efe2 NVFPG------------IQEGKFN---------------------------------EFDGELTSANIYGRVCVFEL--- 1089
  Efe3 NALSGREAILNDA----VEVGSATRKEGSIWTEDTQHKGTYTAYYMAQIAGQDSVKLALDDGVKSSDTYTIVAAAPVVKN 1697
  Yfr1 --------------------------------------------------------------------------------  770
  Yfr5 --------------------------------------------------------------------------------  780
  Yps4 PVNGAVADGANSNSVQAVVSDSEGNAVAGAAVVFSSANATAQITTVIGTTGADGIATATLTNTVAGTSNVVATIDTVNAN 1513
  Yps7 SVDNARKNIGQAISLTVTAKYKSTDVVAPNVKMTFEQVAVVNRQNNTVSISGAVQIAGVNYNSFTGMTDANGQLTVS--- 1321
  Yfr2 --------------------------------------------------GEYIRQIGSLFGEWGDMMDYG---------  856
  Yfr3 N-------------------------------------------------GAELIGHPAVGDTLVAVAMCS---------  800
  Yps2 ------------------------------------------------ATNGTRAPDGTLWGEWGSLTAYSS--------  943
  Ype5 ATWKHD--------------------------------------------SSKVFTYDFTLNYWVGLYSSTN--------  991
  Pru1 IITYGNNGNRQD---------------NTSNWSPVYT-DDNLEINWSLNGSSEFDTAATIKAVVTEPN-GNTFEYP-VTV 2182
  Pal3 --------------------------------------------------------------------------------  542
  Eta1 KVTYISKG-VMA---------------DKGVWTPVFT-DDKFSVTWSTESSDELSKAKFVAIYLKDSS-GKALAQKLVDS 1193
  Sgl1 KISTVSE--------------------QDGVYTATFT-STKTGVG-----------------------------------  900
 Eco26 KISYVGNAQVQ----------------NNGRWTPVYA-DDVITINWSVETSNEYKKPKKASIFFKDAANGADIYKKEILH 1462
  Pmi1 SFEFLSAGESKSN-----------THLDDLTWQPLYTSDDQFIVKWSSSDTPKAINFELFDENGTSIALEERQNYAPKHN 2159
  Eco1 TVANAYAVCIK---------------------------------------------------------------------  937
  Ymo1 STNTVSKGVSGS---------------NSSEWKPAFK-GDQMAVNWRVSGSGKFAGPKKVVVQFIEDGSGSKLFEKESTS 1222
  Yen2 ETTAISNSVKKV---------------NERVWSPVFT-NDNFGVTVDNSQS-KYERADSVKVILLDG--TQELASKELG- 2287
  Ybe1 SITNAHGWPSES---------------NILYWSGTTEGSTVWATDMTLGRTVLITKEKIQLVTCL---------------  945
  Esa2 CNSGQSVTDAQG----------------QATQTCYAVSGNVFGFSTTVTVDPQDTVDPSIPVTATNTRDYLL-------- 1027
  Sen3 LSANNQPTNNEGQSIVTLASDDVVSCKGTATFNGLSKSTATIRFTADTTTEKVDTLNASKTENVVAGKDTITLEATVTDE 1406
 Eco14 NGNVMTVKSNPAG-----------VTLTGTDTDGQTVTLNMGGNWFAQSATKYEWLNSDYDGTAIYSCRQLGAQVASSGA 1021
  Bpe1 RNPAGNASPEVRQDYRD---EFVPANAITNIETNSRNGIVTVSGKTSPDATVVVSFPGGDEARTRAKGDGTFSVSSPMDI 1159
  Bav2 TDAANNKSPEATKAYADGVDKTPPVVSISNVQAADATGIVTVTGHTEAGSTVQVTFPDGQAVNATVDQDGSYTARSSKDV 1326
  Bbr1 S-------------------------------------------------------------------------------  969
  Bpa2 TDAAGNAAAPVRAAYADTVDRTPPVLDTPVLSVASDSGRVTVTGVTEPGARVQVAIPGESLQTVTADSAGRYRAVSPGDV 1508
  Bav1 --------------------------------------------------------------------------------  747
  Pma1 --------------------------------------------------------------------------------  372
  Pma5 --------------------------------------------------------------------------------  373
  Pma3 --------------------------------------------------------------------------------  379
  Pma4 --------------------------------------------------------------------------------  410
  Ssp1 --------------------------------------------------------------------------------  428
  Ssp2 --------------------------------------------------------------------------------  436
 Eco20 ASAGNDQPSNAQSVTFVADKTTATISSIEVIGNRAVADGKTKQTYKVTVTDANNNLLKDSDVTLTASSENLVLDPKGTAK 1255
  Efe5 ELVVDQSTVTTDE-------------SGKATVSVHGRTAGSYKLTATLDELGATTSAEKSFSLYADENNGVLSLSMEPGY 1086
  Plu1 SPDELASLTVSPDHVTEGEGEGHTYTFTATVKDFSGQAKSGITVAWSAANSKGVTITDKNLVTQVVGDGKTDADGKAQYQ 1448
  Pal2 TIEVTAPVGSTKVAKAKLASSIVNLDAGNNVELTLELKDQYDNLIIGVNGSDIMLENSYTAETIDNSRLAWRMDSAGIYK 1991
  Ahy1 --------------------------------------------------------------------------------  916
  Csu1 QTTYSKTLPANVAADITVLSITSGSGDYELTAPANFPSGITMSLEGTGAAQIVKVTGTPTSSQSEASYVFQVKDKKSGLS 1152
  Plu2 --------------------------------------------------------------------------------  302
  Rba1 --------------------------------------------------------------------------------  327
  Pas1 --------------------------------------------------------------------------------  924
  Cla1 GGNGRTALLGNDNGEDGQAFGNYTNGYSIFTLVGHNGNFDLGIYGYKNIEDIRNANIKITYTPAYGGNKQWNLNYFISSK 1402
 ruler ......3930......3940......3950......3960......3970......3980......3990......4000
```

```
  Mba1 --------------------------------------------------------------------------------  331
  Psp2 --------------------------------------------------------------------------------  251
  Sen2 --------------------------------------------------------------------------------  660
  Efe4 --------------------------------------------------------------------------------  672
  Eco3 --------------------------------------------------------------------------------  417
  Cko1 --------------------------------------------------------------------------------  454
  Sen1 --------------------------------------------------------------------------------  460
  Eca1 --------------------------------------------------------------------------------  468
  Esp2 --------------------------------------------------------------------------------  468
  Esa3 --------------------------------------------------------------------------------  471
  Kpn1 --------------------------------------------------------------------------------  460
  Pan1 --------------------------------------------------------------------------------  490
  Eta2 --------------------------------------------------------------------------------  480
  Spr1 --------------------------------------------------------------------------------  497
  Yin1 --------------------------------------------------------------------------------  751
  Yin2 --------------------------------------------------------------------------------  753
  Yfr4 --------------------------------------------------------------------------------  749
  Ymo2 --------------------------------------------------------------------------------  686
 Eco10 --------------------------------------------------------------------------------  734
  Eal1 --------------------------------------------------------------------------------  725
  Sty4 --------------------------------------------------------------------------------  626
 Eco16 --------------------------------------------------------------------------------  711
 Eco25 TN-----------SDLSTLKATVEDGSGNLIEGLT-----VYFALKS-GSATLTSLTAVTDQNGIATTSVRGAITGS---  2335
 Eco15 DG-----------IDSSTITSRVEDDYGFPVEGVD-----ISHGLDTKGSPVVNIPTTRTDQSGQVTATITSTLAET---  1372
  Eco6 DKS------AYIAGEPLTVTITLRDEFGNPALGLTSEVIESYIDSFAVGGATPDSMRWVEQNNGEYTIVWTAWVAEENLV  2414
  Efe2 --------------------------------------------------------------------------------  1089
  Efe3 SAIQRGEAATYTAGDTLTLTVTLQDDWGNPVSGMEN----ILRDSVTLPEAGLQERGWQQTPSGEYEANWTAQKAGTS--  1771
  Yfr1 --------------------------------------------------------------------------------  770
  Yfr5 --------------------------------------------------------------------------------  780
  Yps4 IDTAFVPGAVATITLSVLVNDATADGADTNQVDALVQDANGNAITGAAVVFSSANGADIIAPTMNTGVNGVASTLLTHTQ  1593
  Yps7 -----VTDPNGIGVQTTLKVRAESGDTGMENVIFNVLTSPNTS-----LANMWGYMSEVIINNGITFKRPLLKAEDTSGD  1391
  Yfr2 -----FPAAN----------NWSSEQSGTSNYYYVVNSSRGRS-----SQALY-----------------------TPGG  893
  Yfr3 ---------------------IALCNGVPINYQWEIESSVGSGGFIAIAG--------------------------ATSE  833
  Yps2 --------------------DWQSGEYWVKKTSTDFETMNMDTG-------------------------------ALQPG  972
  Ype5 -------------------LSWAQANASCINAGMRLPTNREVSAG-----------------------------QDVRGV  1023
  Pru1 QAGIQKITLPPKREYFSQSGNLKVTISATGKS---SHKLKN---------------EFVVIDTPIQVTSPKEIG--QQTL  2242
  Pal3 --------------------------------------------------------------------------------  542
  Eta1 PAGTVTTTFEPKPRFWNESLHVELVAQGFDKL---TNSEKSPGINVKNLPPNKIWETFTVRSKALIRSDNGNESTCREIA  1270
  Sgl1 ------------------TIGVAVNGTRLEEL---TAVDAG------------------VYSSTLS--------------  927
 Eco26 INGINVTELHANASLLGKKIKVVLVAEGELNL---KSELEDKNEIN-------VYELMPHEIWQNATITTEMSGWCRATW  1532
  Pmi1 IHDPVKIQVPKQYLTSPKQLTLEVTTFDSNNQ---PYVDPKKYKIN-------IDYFPANNAYKIISLDYNSKFIYTDTG  2229
  Eco1 --------------------------------------------------------------------------------  937
  Ymo1 NLASGSTVIEDTNVKNLVGKRGKFVVKTEGTSGLSS-QDQTKIIDVKRLYPDEIWNKVRVKSGHSIYEKGKSVKDCRTSA  1301
  Yen2 --ITTSSSFEFKPKPDWVGKSLKVKVVAKNDVRQENEVTLDHEVRVGTLEPIDIWQNAIFTRNYSLHNNDGSKRDSCPIV  2365
  Ybe1 --------------------------------------------------------------------------------  945
  Esa2 --------------------------------------------------------------------------------  1027
  Sen3 NGHPVADTTVHWGTDNSSGTFQPGDSSVTDSNGVATVTYSATKAVPTLIGAGINQSEKTITVNYIGNADTAKLSNIKPDK  1486
 Eco14 LQG----VISEWGELETYEGWTNLKNNWLYSSTTVISGNGVKSTIAYLFPGGKEGLLTGIRGYYACR-------------  1084
  Bpe1 PRG--VVSLSTEVNGVQTVVATRTYEDKFTKGGLDFTAFLPYTPKFNGVSTSDTSAVLLSIPRYFYSGG-----------  1226
  Bav2 TQSGDITATATDAAGNPSAPATQAYDDTVDRTAPPVPTISSVTGNAAGQVTVVGTAEPGSTVTINFPGGSEKQVQLNADG  1406
  Bbr1 --------------------------------------------------------------------------------  969
  Bpa2 LRSGTVRVIATDAHGNASAPRDVPYQDAVDRTAPPLTIEAAVEQARTGVVNVSGKTEPNLMVSATFPDGQTAQARADGQG  1588
  Bav1 --------------------------------------------------------------------------------  747
  Pma1 --------------------------------------------------------------------------------  372
  Pma5 --------------------------------------------------------------------------------  373
  Pma3 --------------------------------------------------------------------------------  379
  Pma4 --------------------------------------------------------------------------------  410
  Ssp1 --------------------------------------------------------------------------------  428
  Ssp2 --------------------------------------------------------------------------------  436
 Eco20 TNEQGQAVFTGSTTIAATYTLTAKVEQANGQVSTKTAESKFVADDKNAVLAASPERVDSLVADGKTTATMTVTLMAGVNP  1335
  Efe5 VTDDGEPFGFIARLTDKFGNPMTGEAEFTAGNEKYLDSADKITMTPAKVTLSWTGHAYSEIRTYLPGRTWVKVKVTRGDK  1166
  Plu1 VYSKSGGFVAVMVTAKVNDSSVGSKNKTVEIKANEQDVTDFFIMDYDTKDGKGPGRSVPKERMNFAWPKMRFEPEYLPGE  1528
  Pal2 ASLPLTLVGKHKLSAVINKQRTSTADITVNALKGAANVSQVIITTGKNTISVGEKTELALKVQDRFGNEVDDVLASDIDL  2071
  Ahy1 --------------------------------------------------------------------------------  916
  Csu1 TGSRTLKLTIVGGTTWRRQIADSVMLDYIRKHQMQSKMVQDKIPSTVGLGIGGSATFKALSKEDKGKRKQNYTTSSLVSQ  1232
  Plu2 --------------------------------------------------------------------------------  302
  Rba1 --------------------------------------------------------------------------------  327
  Pas1 --------------------------------------------------------------------------------  924
  Cla1 IYYLGNTYLPFDMNHYTYLLFSKEEASIPTKQNNGIYKFEDLNNKYIKPTYLHLKLP-----------------------  1459
 ruler ......4010......4020......4030......4040......4050......4060......4070......4080
```

```
  Mba1 ----------------------------------------------------------------------   331
  Psp2 ----------------------------------------------------------------------   251
  Sen2 ----------------------------------------------------------------------   660
  Efe4 ----------------------------------------------------------------------   672
  Eco3 ----------------------------------------------------------------------   417
  Cko1 ----------------------------------------------------------------------   454
  Sen1 ----------------------------------------------------------------------   460
  Eca1 ----------------------------------------------------------------------   468
  Esp2 ----------------------------------------------------------------------   468
  Esa3 ----------------------------------------------------------------------   471
  Kpn1 ----------------------------------------------------------------------   460
  Pan1 ----------------------------------------------------------------------   490
  Eta2 ----------------------------------------------------------------------   480
  Spr1 ----------------------------------------------------------------------   497
  Yin1 ----------------------------------------------------------------------   751
  Yin2 ----------------------------------------------------------------------   753
  Yfr4 ----------------------------------------------------------------------   749
  Ymo2 ----------------------------------------------------------------------   686
 Eco10 ----------------------------------------------------------------------   734
  Eal1 ----------------------------------------------------------------------   725
  Sty4 ----------------------------------------------------------------------   626
 Eco16 ----------------------------------------------------------------------   711
 Eco25 VTVSAVTTAGGMQTVDITLVAGPADASQSVLKNNRSSLKGDFTDSAELHLVLHDISGNPIKV------SEGLEFVQSGTN  2409
 Eco15 LTVNVQVPGTANQSATITLVAGTADESKSILKSDVDTLKADYQQSAKLTLTLQDKYGNPIVT------SDHLEFVQSGPF  1446
  Eco6 ASLKLKTWATEIKSSLYGIQPGAAAKNQSTIVADKTIYIAG--DSITVTVVLKDAQGNFITDGVVQLNEENVQVRNADPI  2492
  Efe2 ----------------------------------------------------------------------  1089
  Efe3 LTASLELEGWRKETAPFAIVAGTPVQSESSLQTDKESYTVE--DTLELTVTLRDAWKNPVSG--------KLALVNDAVL  1841
  Yfr1 ----------------------------------------------------------------------   770
  Yfr5 ----------------------------------------------------------------------   780
  Yps4 SGVSNVVATIDTVNANIDTTFVAGAVAAITLTTPVDGAVADGTDSNSVQAVVSDSEGNAVAGAAVVFSSANATAQITTVI  1673
  Yps7 SSANSSSNYANEIWAVFTNTAARAVCSSLPTRPELSGLYSNYPTGSLTTTHGWRTDFYRFRSNTPQSTGFSAVNFLNGTF  1471
  Yfr2 TSVLNKSLCVRDL---------------------------------------------------------   906
  Yfr3 TLTVTGDLQKRAIRVVSH----------------------------------------------------   851
  Yps2 PAYLAFPLCALSI---------------------------------------------------------   985
  Ype5 GSLLSLSIILCNCHRIKGDRSGGHRTR-------------------------------------------  1050
  Pru1 GHKVKYFLEGQTSERNICAYYLTDRTIDLVIAPTMNINTNGRRLLAPIYVKHEILSSEYSGTGAA---GISYPEISTPNT  2319
  Pal3 ----------------------------------------------------------------------   542
  Eta1 GPAGKEHWIDAIVDGGQVNFNGKTLISPMTITGLTDGEQNGHYRNGQFPLTQRNIYSDLSVNFGT---KQIARECWKGHD  1347
  Sgl1 ---------------------------------------------------LSVNVSS------------   934
 Eco26 IDAPTSKTMKSNFSISLASGKSLLFPMSVKVKASSNGGYGGFTYGYFTFSGLSEKNIVNSSSWSG---DDKKGECWKDYW  1609
  Pmi1 ALASSCQNGWSTTTTHLILTPRLVFKGEPKNLLYPIYITTELYDVTLNPSDKLIGHINEKLTYPD---KIIFSEHSKKIA  2306
  Eco1 ----------------------------------------------------------------------   937
  Ymo1 GFWNDTHHVNLNWGVGIDLGNNQLLEGMEVKLRIDKVSSSYISPKDIVTRNILKTFDEVSTPSSNYIAETLHTDCYDPHN  1381
  Yen2 NNLFYPNYARLNWRMQLVLNKDMLHP-----MQITKLES-KTSKHGINMTHIDSSTSEIFDSYDNKDDNRLINKCIKEKY  2439
  Ybe1 ----------------------------------------------------------------------   945
  Esa2 ----------------------------------------------------------------------  1027
  Sen3 TKAVADNTELVTWSVDVKDANGNILPGISVNWSSDDPDLTLAASSSVTNEHGVATITGRTLKARDAVVKATLSAGGQMLS  1566
 Eco14 ----------------------------------------------------------------------  1084
  Bpe1 ----------APVAKIMPPEAD--GSEFAKRAAAAMRAQVTP---GRDSSEYDVR----------------VSWPAGTF  1274
  Bav2 QYSVTS-DGHIPTGDIKASATDREGNKSAEVSKAYMSAPPAP---VINSVTTDANTGRVTVSGTAKPNGKVLVTFPGGSQ  1482
  Bbr1 ----------------------------------------------------------------------   969
  Bpa2 RYQLASPADVARSGRIAVSASDAAGNRAAASAEFTDQVDKTAPAVTIRAVTEQAGTGVATVSGATEPGATVSVTFPDGQV  1668
  Bav1 ----------------------------------------------------------------------   747
  Pma1 ----------------------------------------------------------------------   372
  Pma5 ----------------------------------------------------------------------   373
  Pma3 ----------------------------------------------------------------------   379
  Pma4 ----------------------------------------------------------------------   410
  Ssp1 ----------------------------------------------------------------------   428
  Ssp2 ----------------------------------------------------------------------   436
 Eco20 VGGSMWVDIEAPEGVTEKDYQFLPSKADHFSGGKITRTFSTSKPGVYTFTFNALTYGGYEMTPVKVTINAVAAETENGEE  1415
  Efe5 TYEQTEETWVSELPKNPQQ---------------------------------------------------  1185
  Plu1 LTIAGYTGVYDSSNRNVVDVDGHYFQVKKAGTATLTTTFTHPESGRYLKYIIPDVKIDHFVIVDSSSTGPGIGSAYSKDR  1608
  Pal2 TNTDSQIKTSVKWVKSPLTLGTYITDVQFDKVKSHTLIASVNGQTKMLQIHVQPLKGYSNVAAIALQTPAKIEVAEKTKL  2151
  Ahy1 ----------------------------------------------------------------------   916
  Csu1 PRSFVGNFEKAILMMKLGGGITDSEWRVAWGRDVLFTNPLGRMPAWMYTTKGDQPWEEFFRKSGGPFFWTAFK-------  1305
  Plu2 ----------------------------------------------------------------------   302
  Rba1 ----------------------------------------------------------------------   327
  Pas1 ----------------------------------------------------------------------   924
  Cla1 ----------------------------------------------------------------------  1459
 ruler ......4090......4100......4110......4120......4130......4140......4150......4160
```

CLUSTAL X (1.81.1-alpha) MULTIPLE SEQUENCE ALIGNMENT

File: /Users/saierlab/Desktop/69long.ps   Date: Tue May 12 18:31:19 2009


```
  Mba1 --------------------------------------------------------------------------------  331
  Psp2 --------------------------------------------------------------------------------  251
  Sen2 --------------------------------------------------------------------------------  660
  Efe4 --------------------------------------------------------------------------------  672
  Eco3 --------------------------------------------------------------------------------  417
  Cko1 --------------------------------------------------------------------------------  454
  Sen1 --------------------------------------------------------------------------------  460
  Eca1 --------------------------------------------------------------------------------  468
  Esp2 --------------------------------------------------------------------------------  468
  Esa3 --------------------------------------------------------------------------------  471
  Kpn1 --------------------------------------------------------------------------------  460
  Pan1 --------------------------------------------------------------------------------  490
  Eta2 --------------------------------------------------------------------------------  480
  Spr1 --------------------------------------------------------------------------------  497
  Yin1 --------------------------------------------------------------------------------  751
  Yin2 --------------------------------------------------------------------------------  753
  Yfr4 --------------------------------------------------------------------------------  749
  Ymo2 --------------------------------------------------------------------------------  686
 Eco10 --------------------------------------------------------------------------------  734
  Eal1 --------------------------------------------------------------------------------  725
  Sty4 --------------------------------------------------------------------------------  626
 Eco16 --------------------------------------------------------------------------------  711
 Eco25 AP------------------------YVQVSAIDYSKNFSGEYKATVTG--GGEGIATLIPVLNGVHQAGLSTTIQFTRA 2463
 Eco15 VN------------------------FLKLSDIDYSQRNYGEYTVTVTG--GKEGTATLIPMLNGVHQANLSISLNLIQS 1500
  Eco6 QGNNWVYNGNGQYQRQYMAHFAEANLNAQLKMAGWSDANYSNNYTIKPGEVSPLGSQLRIREVLVVEGADLPVSVLLVDD 2572
  Efe2 -------------------------------------------------------------------------------- 1089
  Efe3 TID-------------------------AASATDVFSEGDTAGTYIRHFTLNSWRDSTQVSIRLQTWDRDATSNPYSVER 1896
  Yfr1 --------------------------------------------------------------------------------  770
  Yfr5 --------------------------------------------------------------------------------  780
  Yps4 GTT-------------------------GADGIATATLTNTVAGTSNVAATIGSITDNIDTVFVAGAVATITLSVPVNDA 1728
  Yps7 SSA-------------------------DNTSYDYVSCKG---------------------------------------- 1486
  Yfr2 --------------------------------------------------------------------------------  906
  Yfr3 --------------------------------------------------------------------------------  851
  Yps2 --------------------------------------------------------------------------------  985
  Ype5 -------------------------------------------------------------------------------- 1050
  Pru1 NDD-------------------------YVYSNNDIYRKVLKNNCYENHSGSGNIT-TTLSFLGSSDELKHHFSWNGKNN 2373
  Pal3 --------------------------------------------------------------------------------  542
  Eta1 GSY-------------------------FIGAKVNYNGENFEYWMVDPHNWTGKGVGTNKYYLDTVTKERNRAAINGS-- 1400
  Sgl1 --------------------------------------------------------------------------------  934
 Eco26 GSY-------------------------NTYMEVQYNQKNYIYRSDSARGWQGMDNNNGPYTDNMIYVK----------- 1653
  Pmi1 EIS-------------------------PTSAHYALDEECRINDSGHGTLKANITIGNRSSYLKKEFHWDGQIGGYF--- 2358
  Eco1 --------------------------------------------------------------------------------  937
  Ymo1 DSV-------------------------ELVLDVEYLGKKTMYKAG-SVYWEGLGRTKKDFVEANIQRK----------- 1424
  Yen2 GTY-------------------------KTYMDIKYAGREYKYEAINDLYWEGEGDDRESDKSSGFKKVP---------- 2484
  Ybe1 --------------------------------------------------------------------------------  945
  Esa2 -------------------------------------------------------------------------------- 1027
  Sen3 AAK-------------------------VTFIGDAKTAMLMSLNVDKFNVLANGGDAATYTAYVEDINHNIVPDATVSWR 1621
 Eco14 -------------------------------------------------------------------------------- 1084
  Bpe1 TPADVGQRADLAVKVF----DKG--------------------------------------------------------- 1293
  Bav2 REVSVSAGGDYTATSS----DDEESGEIKAVLLEGAERSRETKRNYADEAVRLSAKDVEIENGDQAGFWLTVTHSVGSYG 1558
  Bbr1 --------------------------------------------------------------------------------  969
  Bpa2 ARATADRSGAYTVHSARDVTARGNISVIATDAAGNASPSVSRQFSNTVADTVPPSLTIAMVTTDPGNGRITVSGVTEAGA 1748
  Bav1 --------------------------------------------------------------------------------  747
  Pma1 --------------------------------------------------------------------------------  372
  Pma5 --------------------------------------------------------------------------------  373
  Pma3 --------------------------------------------------------------------------------  379
  Pma4 --------------------------------------------------------------------------------  410
  Ssp1 --------------------------------------------------------------------------------  428
  Ssp2 --------------------------------------------------------------------------------  436
 Eco20 EMP----------------------------------------------------------------------------- 1418
  Efe5 -------------------------------------------------------------------------------- 1185
  Plu1 IDTSKPLPSCTRGNRIKESDLGGSIDYLVKDLNLNLIDKGLLGDPRASLNSNGITMGGLQINDESIQVHLLKEKDPNAIF 1688
  Pal2 TLTLMDKFNNGVVGVEAQHIELLIGSTLQTATWVDNQDGSYHTEFALNQAGDTPLIVTVNKFTEMNSIHVNSPSGKDKVA 2231
  Ahy1 --------------------------------------------------------------------------------  916
  Csu1 -------------------------------------------------------------------------------- 1305
  Plu2 --------------------------------------------------------------------------------  302
  Rba1 --------------------------------------------------------------------------------  327
  Pas1 --------------------------------------------------------------------------------  924
  Cla1 -------------------------------------------------------------------------------- 1459
 ruler ......4170......4180......4190......4200......4210......4220......4230......4240
```

```
Mba1   --------------------------------------------------------------------------------   331
Psp2   --------------------------------------------------------------------------------   251
Sen2   --------------------------------------------------------------------------------   660
Efe4   --------------------------------------------------------------------------------   672
Eco3   --------------------------------------------------------------------------------   417
Cko1   --------------------------------------------------------------------------------   454
Sen1   --------------------------------------------------------------------------------   460
Eca1   --------------------------------------------------------------------------------   468
Esp2   --------------------------------------------------------------------------------   468
Esa3   --------------------------------------------------------------------------------   471
Kpn1   --------------------------------------------------------------------------------   460
Pan1   --------------------------------------------------------------------------------   490
Eta2   --------------------------------------------------------------------------------   480
Spr1   --------------------------------------------------------------------------------   497
Yin1   --------------------------------------------------------------------------------   751
Yin2   --------------------------------------------------------------------------------   753
Yfr4   --------------------------------------------------------------------------------   749
Ymo2   --------------------------------------------------------------------------------   686
Eco10  --------------------------------------------------------------------------------   734
Eal1   --------------------------------------------------------------------------------   725
Sty4   --------------------------------------------------------------------------------   626
Eco16  --------------------------------------------------------------------------------   711
Eco25  EDKIMSG---------------------------------------------------------------------TVLV   2474
Eco15  -IKEMSG---------------------------------------------------------------------HVTA   1510
Eco6   FGNPVDNGLDLLDDTVYLQNVEKKEGEKWRYVGDGIYERTYMAYQEGENLTSFMEIKGWRIYGQPSYTILPFVEVELLSV   2652
Efe2   --------------------------------------------------------------------------------   1089
Efe3   RRLPMTG---------------------------------------------------------------------TIVA   1907
Yfr1   --------------------------------------------------------------------------------   770
Yfr5   --------------------------------------------------------------------------------   780
Yps4   TADGADTNQVDALVQDVNGNAITGAAVVFSSANGATILSSTVNTGADGIASTTLTHTQSGVSNVVATIDTVNANIDTTFV   1808
Yps7   --------------------------------------------------------------------------------   1486
Yfr2   --------------------------------------------------------------------------------   906
Yfr3   --------------------------------------------------------------------------------   851
Yps2   --------------------------------------------------------------------------------   985
Ype5   --------------------------------------------------------------------------------   1050
Pru1   --------------------------------------------------------------------------------   2373
Pal3   --------------------------------------------------------------------------------   542
Eta1   --------------------------------------------------------------------------------   1400
Sgl1   --------------------------------------------------------------------------------   934
Eco26  --------------------------------------------------------------------------------   1653
Pmi1   --------------------------------------------------------------------------------   2358
Eco1   --------------------------------------------------------------------------------   937
Ymo1   --------------------------------------------------------------------------------   1424
Yen2   --------------------------------------------------------------------------------   2484
Ybe1   --------------------------------------------------------------------------------   945
Esa2   --------------------------------------------------------------------------------   1027
Sen3   TTMNKLSSGTSKTNSSGKATVKLSGNSVGMVTVTATINNSSMSKSGVKFINVIEDTWYVKSGSSTYTSSAIKGYPDLGFV   1701
Eco14  --------------------------------------------------------------------------------   1084
Bpe1   --------TGKYMLLLAP------------------VNFPY---------------------------------------   1308
Bav2   YSLLSIQTSGKVSVKLLPSDPSDRNQQELVKVIALQVSGPYAWSGAVNVSADKYIDQNPPSWVPAGTYPVKIVVTQIATG   1638
Bbr1   --------------------------------------------------------------------------------   969
Bpa2   RVHVVFPGGASDTVTADSKGGYRATSAGDVGSGAVTAQATDASANRSPLKTYAYTDAWQPRQLSAKDFSQSRYFVPLTTR   1828
Bav1   --------------------------------------------------------------------------------   747
Pma1   --------------------------------------------------------------------------------   372
Pma5   --------------------------------------------------------------------------------   373
Pma3   --------------------------------------------------------------------------------   379
Pma4   --------------------------------------------------------------------------------   410
Ssp1   --------------------------------------------------------------------------------   428
Ssp2   --------------------------------------------------------------------------------   436
Eco20  --------------------------------------------------------------------------------   1418
Efe5   --------------------------------------------------------------------------------   1185
Plu1   YVVLCEE-------------------------------------------------------------------------   1695
Pal2   SIQLAATVTQVLPNTSTVLTLTLKDQYGNGVNNVLSKDISLSNSYTPENLTSPNWAEDGKQSGIYTVSVNLQKVTEHTLT   2311
Ahy1   --------------------------------------------------------------------------------   916
Csu1   --------------------------------------------------------------------------------   1305
Plu2   --------------------------------------------------------------------------------   302
Rba1   --------------------------------------------------------------------------------   327
Pas1   --------------------------------------------------------------------------------   924
Cla1   --------------------------------------------------------------------------------   1459
ruler  ......4250......4260......4270......4280......4290......4300......4310......4320
```

```
Mba1    --------------------------------------------------------------------------------  331
Psp2    --------------------------------------------------------------------------------  251
Sen2    --------------------------------------------------------------------------------  660
Efe4    --------------------------------------------------------------------------------  672
Eco3    --------------------------------------------------------------------------------  417
Cko1    --------------------------------------------------------------------------------  454
Sen1    --------------------------------------------------------------------------------  460
Eca1    --------------------------------------------------------------------------------  468
Esp2    --------------------------------------------------------------------------------  468
Esa3    --------------------------------------------------------------------------------  471
Kpn1    --------------------------------------------------------------------------------  460
Pan1    --------------------------------------------------------------------------------  490
Eta2    --------------------------------------------------------------------------------  480
Spr1    --------------------------------------------------------------------------------  497
Yin1    --------------------------------------------------------------------------------  751
Yin2    --------------------------------------------------------------------------------  753
Yfr4    --------------------------------------------------------------------------------  749
Ymo2    --------------------------------------------------------------------------------  686
Eco10   --------------------------------------------------------------------------------  734
Eal1    --------------------------------------------------------------------------------  725
Sty4    --------------------------------------------------------------------------------  626
Eco16   --------------------------------------------------------------------------------  711
Eco25   NGANLPTTT-FPSQGFTGAYYQLNNDNFAPGKTAADYEFSSSASWVDVDATGKVTFKN------VGSKWER-ITATPKTG  2546
Eco15   NNHTFSTAK-FPSEGFAGAYYTLNNDNFEAGKTVDDYMFSSSQGWVSVDASGKVSFAN------IGDQTSVTISAVPRQG  1583
Eco6    NGVKFRATDGFPETGFDGAKFTLL---LTHNMKNTDYNWTAGIYGINVDSNGEVTLS-------VLIRSEVTITGKPKNG  2722
Efe2    --------------------------------------------------------------------------------  1089
Efe3    NDGNFGLDEGFPTTGFIGAWFILSSDMQIEYDKTTCGGNENSCDWLHVNTMSPNSHEVSFIGQPTSETREVTINFISLSD  1987
Yfr1    --------------------------------------------------------------------------------  770
Yfr5    --------------------------------------------------------------------------------  780
Yps4    AGAVATITLSVLVNDATADGADTNQVDALVQDANGNAITGAAVVFSSANGATIIVPTMNTGANGVASTLLTHTVAGTSNV  1888
Yps7    --------------------------------------------------------------------------------  1486
Yfr2    --------------------------------------------------------------------------------  906
Yfr3    --------------------------------------------------------------------------------  851
Yps2    --------------------------------------------------------------------------------  985
Ype5    --------------------------------------------------------------------------------  1050
Pru1    --------------------------------------------------------------------------------  2373
Pal3    --------------------------------------------------------------------------------  542
Eta1    --------------------------------------------------------------------------------  1400
Sgl1    --------------------------------------------------------------------------------  934
Eco26   --------------------------------------------------------------------------------  1653
Pmi1    --------------------------------------------------------------------------------  2358
Eco1    --------------------------------------------------------------------------------  937
Ymo1    --------------------------------------------------------------------------------  1424
Yen2    --------------------------------------------------------------------------------  2484
Ybe1    --------------------------------------------------------------------------------  945
Esa2    --------------------------------------------------------------------------------  1027
Sen3    VASPTTGPTYLEWSPTGTSKVTTPVTLIDDAGQQYTVNLKGNRTSDCSTRPLNAAVGCSSSAGYKAQFTWNINDNKSIPP  1781
Eco14   --------------------------------------------------------------------------------  1084
Bpe1    --------------------------------------------------------------------------------  1308
Bav2    ATVEVSGTIVYSKTAV----------------------------------------------------------------  1654
Bbr1    --------------------------------------------------------------------------------  969
Bpa2    GGTDNEPLLRITNQQIPTAELRVALKPYKTEDRAGRLVREAITLKWGNAPGVGGDARALHFYVPDSAKAKLRKELTEVER  1908
Bav1    --------------------------------------------------------------------------------  747
Pma1    --------------------------------------------------------------------------------  372
Pma5    --------------------------------------------------------------------------------  373
Pma3    --------------------------------------------------------------------------------  379
Pma4    --------------------------------------------------------------------------------  410
Ssp1    --------------------------------------------------------------------------------  428
Ssp2    --------------------------------------------------------------------------------  436
Eco20   --------------------------------------------------------------------------------  1418
Efe5    --------------------------------------------------------------------------------  1185
Plu1    --------------------------------------------------------------------------------  1695
Pal2    AKVNNLDNILKITVQPFTEIQHVNSLELAIDNNNITLGENVMFTLSTKDIYGNNVMIKPADIHLNNANGTVNQPTWKEQN  2391
Ahy1    --------------------------------------------------------------------------------  916
Csu1    --------------------------------------------------------------------------------  1305
Plu2    --------------------------------------------------------------------------------  302
Rba1    --------------------------------------------------------------------------------  327
Pas1    --------------------------------------------------------------------------------  924
Cla1    --------------------------------------------------------------------------------  1459
ruler   ......4330......4340......4350......4360......4370......4380......4390......4400
```

```
  Mba1 --------------------------------------------------------------------------------  331
  Psp2 --------------------------------------------------------------------------------  251
  Sen2 --------------------------------------------------------------------------------  660
  Efe4 --------------------------------------------------------------------------------  672
  Eco3 --------------------------------------------------------------------------------  417
  Cko1 --------------------------------------------------------------------------------  454
  Sen1 --------------------------------------------------------------------------------  460
  Eca1 --------------------------------------------------------------------------------  468
  Esp2 --------------------------------------------------------------------------------  468
  Esa3 --------------------------------------------------------------------------------  471
  Kpn1 --------------------------------------------------------------------------------  460
  Pan1 --------------------------------------------------------------------------------  490
  Eta2 --------------------------------------------------------------------------------  480
  Spr1 --------------------------------------------------------------------------------  497
  Yin1 --------------------------------------------------------------------------------  751
  Yin2 --------------------------------------------------------------------------------  753
  Yfr4 --------------------------------------------------------------------------------  749
  Ymo2 --------------------------------------------------------------------------------  686
 Eco10 --------------------------------------------------------------------------------  734
  Eal1 --------------------------------------------------------------------------------  725
  Sty4 --------------------------------------------------------------------------------  626
 Eco16 --------------------------------------------------------------------------------  711
 Eco25 -GPSYIYEIRVKSWWVNAGD-AFMIYSLAENFCSS--NGYTLPLGDH-LNHSRS-RGIGSLYSEWGDMGHYTTEAGFHSN 2620
 Eco15 -GTTYQTLIKLKGWWVNNGN-HTNIWLAANALCHAKNDGYNLPGITH-LTSGENKRTQGSLYGEWGNVGAFSSNSQFTPG 1660
  Eco6 KGNDVVFKFKIKKWFTSLGATSSNTWDIINTSCSYGQMPSSLELAQR-PSGGVVPRKVGTLWGEYGNLKTYGN--AFSGT 2799
  Efe2 -------------------------------------------------------------------------------- 1089
  Efe3 DNIIYTYTFNLTAWFSTISPVQDTRQQAIDRCGGAENLPLLKDLTSATALGQPGIRGIGNLHGEWGNINRILNTSIFNV- 2066
  Yfr1 --------------------------------------------------------------------------------  770
  Yfr5 --------------------------------------------------------------------------------  780
  Yps4 VATIGSITNNIDTAFVAGAVATITLTTPVNGAVADGANSNSVQAVVSDSEGNAVAGAAVVFSSANATAQITTVIGTTGAD 1968
  Yps7 -------------------------------------------------------------------------------- 1486
  Yfr2 --------------------------------------------------------------------------------  906
  Yfr3 --------------------------------------------------------------------------------  851
  Yps2 --------------------------------------------------------------------------------  985
  Ype5 -------------------------------------------------------------------------------- 1050
  Pru1 -------------------------------------------------------------------------------- 2373
  Pal3 --------------------------------------------------------------------------------  542
  Eta1 -------------------------------------------------------------------------------- 1400
  Sgl1 --------------------------------------------------------------------------------  934
 Eco26 -------------------------------------------------------------------------------- 1653
  Pmi1 -------------------------------------------------------------------------------- 2358
  Eco1 --------------------------------------------------------------------------------  937
  Ymo1 -------------------------------------------------------------------------------- 1424
  Yen2 -------------------------------------------------------------------------------- 2484
  Ybe1 --------------------------------------------------------------------------------  945
  Esa2 -------------------------------------------------------------------------------- 1027
  Sen3 GHYTGLIHFYGKDWHTAWAFEYRLTMDLTIN------------------------------------------------- 1812
 Eco14 -------------------------------------------------------------------------------- 1084
  Bpe1 -------------------------------------------------------------------------------- 1308
  Bav2 -------------------------------------------------------------------------------- 1654
  Bbr1 --------------------------------------------------------------------------------  969
  Bpa2 SNDYKFWIEFTDTRFGGVARLYIKVISTL--------------------------------------------------- 1937
  Bav1 --------------------------------------------------------------------------------  747
  Pma1 --------------------------------------------------------------------------------  372
  Pma5 --------------------------------------------------------------------------------  373
  Pma3 --------------------------------------------------------------------------------  379
  Pma4 --------------------------------------------------------------------------------  410
  Ssp1 --------------------------------------------------------------------------------  428
  Ssp2 --------------------------------------------------------------------------------  436
 Eco20 -------------------------------------------------------------------------------- 1418
  Efe5 -------------------------------------------------------------------------------- 1185
  Plu1 -------------------------------------------------------------------------------- 1695
  Pal2 GEYKGEMILSISGNYVITANVGTQISAPINLTVQAGTPVFATGKSQLSVNRDDLDENSSTNAIVTLELKDANGVAIKGKK 2471
  Ahy1 --------------------------------------------------------------------------------  916
  Csu1 -------------------------------------------------------------------------------- 1305
  Plu2 --------------------------------------------------------------------------------  302
  Rba1 --------------------------------------------------------------------------------  327
  Pas1 --------------------------------------------------------------------------------  924
  Cla1 -------------------------------------------------------------------------------- 1459
 ruler ......4410......4420......4430......4440......4450......4460......4470......4480
```

```
  Mba1 --------------------------------------------------------------------------------  331
  Psp2 --------------------------------------------------------------------------------  251
  Sen2 --------------------------------------------------------------------------------  660
  Efe4 --------------------------------------------------------------------------------  672
  Eco3 --------------------------------------------------------------------------------  417
  Cko1 --------------------------------------------------------------------------------  454
  Sen1 --------------------------------------------------------------------------------  460
  Eca1 --------------------------------------------------------------------------------  468
  Esp2 --------------------------------------------------------------------------------  468
  Esa3 --------------------------------------------------------------------------------  471
  Kpn1 --------------------------------------------------------------------------------  460
  Pan1 --------------------------------------------------------------------------------  490
  Eta2 --------------------------------------------------------------------------------  480
  Spr1 --------------------------------------------------------------------------------  497
  Yin1 --------------------------------------------------------------------------------  751
  Yin2 --------------------------------------------------------------------------------  753
  Yfr4 --------------------------------------------------------------------------------  749
  Ymo2 --------------------------------------------------------------------------------  686
 Eco10 --------------------------------------------------------------------------------  734
  Eal1 --------------------------------------------------------------------------------  725
  Sty4 --------------------------------------------------------------------------------  626
 Eco16 --------------------------------------------------------------------------------  711
 Eco25 MYWSSSPANSNEQYVVSLATGDQSVFEKLGFAYATCYKNL----------------------------------------  2660
 Eco15 AYWTSESDDYSRHYYVQMLTGMTGSDADSSPQLTACRKSL----------------------------------------  1700
  Eco6 DYWTS-TQLMGVHEKFNPETGISELGTGKSSGLCVEYY------------------------------------------  2836
  Efe2 --------------------------------------------------------------------------------  1089
  Efe3 --WTNEIFPPVYYYIVNVSVGIPNMQSENAHINTICRTIL----------------------------------------  2104
  Yfr1 --------------------------------------------------------------------------------  770
  Yfr5 --------------------------------------------------------------------------------  780
  Yps4 GIATATLTNTVAGTSNVVATIGSITDNIDTVFVAGAVATITLTTPVNGAVADGANSNSVQAVVSDSEGNPVTGATVVFSS  2048
  Yps7 --------------------------------------------------------------------------------  1486
  Yfr2 --------------------------------------------------------------------------------  906
  Yfr3 --------------------------------------------------------------------------------  851
  Yps2 --------------------------------------------------------------------------------  985
  Ype5 --------------------------------------------------------------------------------  1050
  Pru1 --------------------------------------------------------------------------------  2373
  Pal3 --------------------------------------------------------------------------------  542
  Eta1 --------------------------------------------------------------------------------  1400
  Sgl1 --------------------------------------------------------------------------------  934
 Eco26 --------------------------------------------------------------------------------  1653
  Pmi1 --------------------------------------------------------------------------------  2358
  Eco1 --------------------------------------------------------------------------------  937
  Ymo1 --------------------------------------------------------------------------------  1424
  Yen2 --------------------------------------------------------------------------------  2484
  Ybe1 --------------------------------------------------------------------------------  945
  Esa2 --------------------------------------------------------------------------------  1027
  Sen3 --------------------------------------------------------------------------------  1812
 Eco14 --------------------------------------------------------------------------------  1084
  Bpe1 --------------------------------------------------------------------------------  1308
  Bav2 --------------------------------------------------------------------------------  1654
  Bbr1 --------------------------------------------------------------------------------  969
  Bpa2 --------------------------------------------------------------------------------  1937
  Bav1 --------------------------------------------------------------------------------  747
  Pma1 --------------------------------------------------------------------------------  372
  Pma5 --------------------------------------------------------------------------------  373
  Pma3 --------------------------------------------------------------------------------  379
  Pma4 --------------------------------------------------------------------------------  410
  Ssp1 --------------------------------------------------------------------------------  428
  Ssp2 --------------------------------------------------------------------------------  436
 Eco20 --------------------------------------------------------------------------------  1418
  Efe5 --------------------------------------------------------------------------------  1185
  Plu1 --------------------------------------------------------------------------------  1695
  Pal2 PHIQATAGKIDRIMIETTDGVYTANFNNPVVGESTIYLDNASIDYSGSTP------------------------------  2521
  Ahy1 --------------------------------------------------------------------------------  916
  Csu1 --------------------------------------------------------------------------------  1305
  Plu2 --------------------------------------------------------------------------------  302
  Rba1 --------------------------------------------------------------------------------  327
  Pas1 --------------------------------------------------------------------------------  924
  Cla1 --------------------------------------------------------------------------------  1459
 ruler ......4490......4500......4510......4520......4530......4540......4550......4560
```

```
Mba1   --------------------------------------------------------------------------------   331
Psp2   --------------------------------------------------------------------------------   251
Sen2   --------------------------------------------------------------------------------   660
Efe4   --------------------------------------------------------------------------------   672
Eco3   --------------------------------------------------------------------------------   417
Cko1   --------------------------------------------------------------------------------   454
Sen1   --------------------------------------------------------------------------------   460
Eca1   --------------------------------------------------------------------------------   468
Esp2   --------------------------------------------------------------------------------   468
Esa3   --------------------------------------------------------------------------------   471
Kpn1   --------------------------------------------------------------------------------   460
Pan1   --------------------------------------------------------------------------------   490
Eta2   --------------------------------------------------------------------------------   480
Spr1   --------------------------------------------------------------------------------   497
Yin1   --------------------------------------------------------------------------------   751
Yin2   --------------------------------------------------------------------------------   753
Yfr4   --------------------------------------------------------------------------------   749
Ymo2   --------------------------------------------------------------------------------   686
Eco10  --------------------------------------------------------------------------------   734
Eal1   --------------------------------------------------------------------------------   725
Sty4   --------------------------------------------------------------------------------   626
Eco16  --------------------------------------------------------------------------------   711
Eco25  --------------------------------------------------------------------------------  2660
Eco15  --------------------------------------------------------------------------------  1700
Eco6   --------------------------------------------------------------------------------  2836
Efe2   --------------------------------------------------------------------------------  1089
Efe3   --------------------------------------------------------------------------------  2104
Yfr1   --------------------------------------------------------------------------------   770
Yfr5   --------------------------------------------------------------------------------   780
Yps4   SNATAQITTVIGTTGADGIATATLTNTVAGTSNVVATIDTVNANIDTTFVPGAVATITLTTPVDGAVADGANSNSVQAVV  2128
Yps7   --------------------------------------------------------------------------------  1486
Yfr2   --------------------------------------------------------------------------------   906
Yfr3   --------------------------------------------------------------------------------   851
Yps2   --------------------------------------------------------------------------------   985
Ype5   --------------------------------------------------------------------------------  1050
Pru1   --------------------------------------------------------------------------------  2373
Pal3   --------------------------------------------------------------------------------   542
Eta1   --------------------------------------------------------------------------------  1400
Sgl1   --------------------------------------------------------------------------------   934
Eco26  --------------------------------------------------------------------------------  1653
Pmi1   --------------------------------------------------------------------------------  2358
Eco1   --------------------------------------------------------------------------------   937
Ymo1   --------------------------------------------------------------------------------  1424
Yen2   --------------------------------------------------------------------------------  2484
Ybe1   --------------------------------------------------------------------------------   945
Esa2   --------------------------------------------------------------------------------  1027
Sen3   --------------------------------------------------------------------------------  1812
Eco14  --------------------------------------------------------------------------------  1084
Bpe1   --------------------------------------------------------------------------------  1308
Bav2   --------------------------------------------------------------------------------  1654
Bbr1   --------------------------------------------------------------------------------   969
Bpa2   --------------------------------------------------------------------------------  1937
Bav1   --------------------------------------------------------------------------------   747
Pma1   --------------------------------------------------------------------------------   372
Pma5   --------------------------------------------------------------------------------   373
Pma3   --------------------------------------------------------------------------------   379
Pma4   --------------------------------------------------------------------------------   410
Ssp1   --------------------------------------------------------------------------------   428
Ssp2   --------------------------------------------------------------------------------   436
Eco20  --------------------------------------------------------------------------------  1418
Efe5   --------------------------------------------------------------------------------  1185
Plu1   --------------------------------------------------------------------------------  1695
Pal2   --------------------------------------------------------------------------------  2521
Ahy1   --------------------------------------------------------------------------------   916
Csu1   --------------------------------------------------------------------------------  1305
Plu2   --------------------------------------------------------------------------------   302
Rba1   --------------------------------------------------------------------------------   327
Pas1   --------------------------------------------------------------------------------   924
Cla1   --------------------------------------------------------------------------------  1459
ruler  ......4570......4580......4590......4600......4610......4620......4630......4640
```

```
  Mba1 --------------------------------------------------------------------------------  331
  Psp2 --------------------------------------------------------------------------------  251
  Sen2 --------------------------------------------------------------------------------  660
  Efe4 --------------------------------------------------------------------------------  672
  Eco3 --------------------------------------------------------------------------------  417
  Cko1 --------------------------------------------------------------------------------  454
  Sen1 --------------------------------------------------------------------------------  460
  Eca1 --------------------------------------------------------------------------------  468
  Esp2 --------------------------------------------------------------------------------  468
  Esa3 --------------------------------------------------------------------------------  471
  Kpn1 --------------------------------------------------------------------------------  460
  Pan1 --------------------------------------------------------------------------------  490
  Eta2 --------------------------------------------------------------------------------  480
  Spr1 --------------------------------------------------------------------------------  497
  Yin1 --------------------------------------------------------------------------------  751
  Yin2 --------------------------------------------------------------------------------  753
  Yfr4 --------------------------------------------------------------------------------  749
  Ymo2 --------------------------------------------------------------------------------  686
 Eco10 --------------------------------------------------------------------------------  734
  Eal1 --------------------------------------------------------------------------------  725
  Sty4 --------------------------------------------------------------------------------  626
 Eco16 --------------------------------------------------------------------------------  711
 Eco25 -------------------------------------------------------------------------------- 2660
 Eco15 -------------------------------------------------------------------------------- 1700
  Eco6 -------------------------------------------------------------------------------- 2836
  Efe2 -------------------------------------------------------------------------------- 1089
  Efe3 -------------------------------------------------------------------------------- 2104
  Yfr1 --------------------------------------------------------------------------------  770
  Yfr5 --------------------------------------------------------------------------------  780
  Yps4 TDSGGNPVTGAAVVFSSANATAQITTVIGTTGADGIATATLTNTVAGTSNVVATVDTVNANIDTTFVAGAVATITLTTPV 2208
  Yps7 -------------------------------------------------------------------------------- 1486
  Yfr2 --------------------------------------------------------------------------------  906
  Yfr3 --------------------------------------------------------------------------------  851
  Yps2 --------------------------------------------------------------------------------  985
  Ype5 -------------------------------------------------------------------------------- 1050
  Pru1 -------------------------------------------------------------------------------- 2373
  Pal3 --------------------------------------------------------------------------------  542
  Eta1 -------------------------------------------------------------------------------- 1400
  Sgl1 --------------------------------------------------------------------------------  934
 Eco26 -------------------------------------------------------------------------------- 1653
  Pmi1 -------------------------------------------------------------------------------- 2358
  Eco1 --------------------------------------------------------------------------------  937
  Ymo1 -------------------------------------------------------------------------------- 1424
  Yen2 -------------------------------------------------------------------------------- 2484
  Ybe1 --------------------------------------------------------------------------------  945
  Esa2 -------------------------------------------------------------------------------- 1027
  Sen3 -------------------------------------------------------------------------------- 1812
 Eco14 -------------------------------------------------------------------------------- 1084
  Bpe1 -------------------------------------------------------------------------------- 1308
  Bav2 -------------------------------------------------------------------------------- 1654
  Bbr1 --------------------------------------------------------------------------------  969
  Bpa2 -------------------------------------------------------------------------------- 1937
  Bav1 --------------------------------------------------------------------------------  747
  Pma1 --------------------------------------------------------------------------------  372
  Pma5 --------------------------------------------------------------------------------  373
  Pma3 --------------------------------------------------------------------------------  379
  Pma4 --------------------------------------------------------------------------------  410
  Ssp1 --------------------------------------------------------------------------------  428
  Ssp2 --------------------------------------------------------------------------------  436
 Eco20 -------------------------------------------------------------------------------- 1418
  Efe5 -------------------------------------------------------------------------------- 1185
  Plu1 -------------------------------------------------------------------------------- 1695
  Pal2 -------------------------------------------------------------------------------- 2521
  Ahy1 --------------------------------------------------------------------------------  916
  Csu1 -------------------------------------------------------------------------------- 1305
  Plu2 --------------------------------------------------------------------------------  302
  Rba1 --------------------------------------------------------------------------------  327
  Pas1 --------------------------------------------------------------------------------  924
  Cla1 -------------------------------------------------------------------------------- 1459
 ruler ......4650......4660......4670......4680......4690......4700......4710......4720
```

```
Mba1   --------------------------------------------------------------------------------  331
Psp2   --------------------------------------------------------------------------------  251
Sen2   --------------------------------------------------------------------------------  660
Efe4   --------------------------------------------------------------------------------  672
Eco3   --------------------------------------------------------------------------------  417
Cko1   --------------------------------------------------------------------------------  454
Sen1   --------------------------------------------------------------------------------  460
Eca1   --------------------------------------------------------------------------------  468
Esp2   --------------------------------------------------------------------------------  468
Esa3   --------------------------------------------------------------------------------  471
Kpn1   --------------------------------------------------------------------------------  460
Pan1   --------------------------------------------------------------------------------  490
Eta2   --------------------------------------------------------------------------------  480
Spr1   --------------------------------------------------------------------------------  497
Yin1   --------------------------------------------------------------------------------  751
Yin2   --------------------------------------------------------------------------------  753
Yfr4   --------------------------------------------------------------------------------  749
Ymo2   --------------------------------------------------------------------------------  686
Eco10  --------------------------------------------------------------------------------  734
Eal1   --------------------------------------------------------------------------------  725
Sty4   --------------------------------------------------------------------------------  626
Eco16  --------------------------------------------------------------------------------  711
Eco25  --------------------------------------------------------------------------------  2660
Eco15  --------------------------------------------------------------------------------  1700
Eco6   --------------------------------------------------------------------------------  2836
Efe2   --------------------------------------------------------------------------------  1089
Efe3   --------------------------------------------------------------------------------  2104
Yfr1   --------------------------------------------------------------------------------  770
Yfr5   --------------------------------------------------------------------------------  780
Yps4   NGAVANGADSNSVQAVVSDSEGNAVAGAAVVFSSANATAQITTVIGTTGADGIATATLINTVAGTSNVVATIDTVNANID  2288
Yps7   --------------------------------------------------------------------------------  1486
Yfr2   --------------------------------------------------------------------------------  906
Yfr3   --------------------------------------------------------------------------------  851
Yps2   --------------------------------------------------------------------------------  985
Ype5   --------------------------------------------------------------------------------  1050
Pru1   --------------------------------------------------------------------------------  2373
Pal3   --------------------------------------------------------------------------------  542
Eta1   --------------------------------------------------------------------------------  1400
Sgl1   --------------------------------------------------------------------------------  934
Eco26  --------------------------------------------------------------------------------  1653
Pmi1   --------------------------------------------------------------------------------  2358
Eco1   --------------------------------------------------------------------------------  937
Ymo1   --------------------------------------------------------------------------------  1424
Yen2   --------------------------------------------------------------------------------  2484
Ybe1   --------------------------------------------------------------------------------  945
Esa2   --------------------------------------------------------------------------------  1027
Sen3   --------------------------------------------------------------------------------  1812
Eco14  --------------------------------------------------------------------------------  1084
Bpe1   --------------------------------------------------------------------------------  1308
Bav2   --------------------------------------------------------------------------------  1654
Bbr1   --------------------------------------------------------------------------------  969
Bpa2   --------------------------------------------------------------------------------  1937
Bav1   --------------------------------------------------------------------------------  747
Pma1   --------------------------------------------------------------------------------  372
Pma5   --------------------------------------------------------------------------------  373
Pma3   --------------------------------------------------------------------------------  379
Pma4   --------------------------------------------------------------------------------  410
Ssp1   --------------------------------------------------------------------------------  428
Ssp2   --------------------------------------------------------------------------------  436
Eco20  --------------------------------------------------------------------------------  1418
Efe5   --------------------------------------------------------------------------------  1185
Plu1   --------------------------------------------------------------------------------  1695
Pal2   --------------------------------------------------------------------------------  2521
Ahy1   --------------------------------------------------------------------------------  916
Csu1   --------------------------------------------------------------------------------  1305
Plu2   --------------------------------------------------------------------------------  302
Rba1   --------------------------------------------------------------------------------  327
Pas1   --------------------------------------------------------------------------------  924
Cla1   --------------------------------------------------------------------------------  1459
ruler  ......4730......4740......4750......4760......4770......4780......4790......4800
```

```
Mba1   --------------------------------------------------------------------------------   331
Psp2   --------------------------------------------------------------------------------   251
Sen2   --------------------------------------------------------------------------------   660
Efe4   --------------------------------------------------------------------------------   672
Eco3   --------------------------------------------------------------------------------   417
Cko1   --------------------------------------------------------------------------------   454
Sen1   --------------------------------------------------------------------------------   460
Eca1   --------------------------------------------------------------------------------   468
Esp2   --------------------------------------------------------------------------------   468
Esa3   --------------------------------------------------------------------------------   471
Kpn1   --------------------------------------------------------------------------------   460
Pan1   --------------------------------------------------------------------------------   490
Eta2   --------------------------------------------------------------------------------   480
Spr1   --------------------------------------------------------------------------------   497
Yin1   --------------------------------------------------------------------------------   751
Yin2   --------------------------------------------------------------------------------   753
Yfr4   --------------------------------------------------------------------------------   749
Ymo2   --------------------------------------------------------------------------------   686
Eco10  --------------------------------------------------------------------------------   734
Eal1   --------------------------------------------------------------------------------   725
Sty4   --------------------------------------------------------------------------------   626
Eco16  --------------------------------------------------------------------------------   711
Eco25  --------------------------------------------------------------------------------  2660
Eco15  --------------------------------------------------------------------------------  1700
Eco6   --------------------------------------------------------------------------------  2836
Efe2   --------------------------------------------------------------------------------  1089
Efe3   --------------------------------------------------------------------------------  2104
Yfr1   --------------------------------------------------------------------------------   770
Yfr5   --------------------------------------------------------------------------------   780
Yps4   TTFVAGAVATITLTTPVDGAVANGADSNSVQAVVSDSEGNAVAGAAVVFSSANATAQITTVIGTTGADGIATATLTNTVA  2368
Yps7   --------------------------------------------------------------------------------  1486
Yfr2   --------------------------------------------------------------------------------   906
Yfr3   --------------------------------------------------------------------------------   851
Yps2   --------------------------------------------------------------------------------   985
Ype5   --------------------------------------------------------------------------------  1050
Pru1   --------------------------------------------------------------------------------  2373
Pal3   --------------------------------------------------------------------------------   542
Eta1   --------------------------------------------------------------------------------  1400
Sgl1   --------------------------------------------------------------------------------   934
Eco26  --------------------------------------------------------------------------------  1653
Pmi1   --------------------------------------------------------------------------------  2358
Eco1   --------------------------------------------------------------------------------   937
Ymo1   --------------------------------------------------------------------------------  1424
Yen2   --------------------------------------------------------------------------------  2484
Ybe1   --------------------------------------------------------------------------------   945
Esa2   --------------------------------------------------------------------------------  1027
Sen3   --------------------------------------------------------------------------------  1812
Eco14  --------------------------------------------------------------------------------  1084
Bpe1   --------------------------------------------------------------------------------  1308
Bav2   --------------------------------------------------------------------------------  1654
Bbr1   --------------------------------------------------------------------------------   969
Bpa2   --------------------------------------------------------------------------------  1937
Bav1   --------------------------------------------------------------------------------   747
Pma1   --------------------------------------------------------------------------------   372
Pma5   --------------------------------------------------------------------------------   373
Pma3   --------------------------------------------------------------------------------   379
Pma4   --------------------------------------------------------------------------------   410
Ssp1   --------------------------------------------------------------------------------   428
Ssp2   --------------------------------------------------------------------------------   436
Eco20  --------------------------------------------------------------------------------  1418
Efe5   --------------------------------------------------------------------------------  1185
Plu1   --------------------------------------------------------------------------------  1695
Pal2   --------------------------------------------------------------------------------  2521
Ahy1   --------------------------------------------------------------------------------   916
Csu1   --------------------------------------------------------------------------------  1305
Plu2   --------------------------------------------------------------------------------   302
Rba1   --------------------------------------------------------------------------------   327
Pas1   --------------------------------------------------------------------------------   924
Cla1   --------------------------------------------------------------------------------  1459
ruler  ......4810......4820......4830......4840......4850......4860......4870......4880
```

```
   Mba1 --------------------------------------------------------------------------------  331
   Psp2 --------------------------------------------------------------------------------  251
   Sen2 --------------------------------------------------------------------------------  660
   Efe4 --------------------------------------------------------------------------------  672
   Eco3 --------------------------------------------------------------------------------  417
   Cko1 --------------------------------------------------------------------------------  454
   Sen1 --------------------------------------------------------------------------------  460
   Eca1 --------------------------------------------------------------------------------  468
   Esp2 --------------------------------------------------------------------------------  468
   Esa3 --------------------------------------------------------------------------------  471
   Kpn1 --------------------------------------------------------------------------------  460
   Pan1 --------------------------------------------------------------------------------  490
   Eta2 --------------------------------------------------------------------------------  480
   Spr1 --------------------------------------------------------------------------------  497
   Yin1 --------------------------------------------------------------------------------  751
   Yin2 --------------------------------------------------------------------------------  753
   Yfr4 --------------------------------------------------------------------------------  749
   Ymo2 --------------------------------------------------------------------------------  686
  Eco10 --------------------------------------------------------------------------------  734
   Eal1 --------------------------------------------------------------------------------  725
   Sty4 --------------------------------------------------------------------------------  626
  Eco16 --------------------------------------------------------------------------------  711
  Eco25 --------------------------------------------------------------------------------  2660
  Eco15 --------------------------------------------------------------------------------  1700
   Eco6 --------------------------------------------------------------------------------  2836
   Efe2 --------------------------------------------------------------------------------  1089
   Efe3 --------------------------------------------------------------------------------  2104
   Yfr1 --------------------------------------------------------------------------------  770
   Yfr5 --------------------------------------------------------------------------------  780
   Yps4 GTSNVVATIGSITNNIDTAFVAGAVATITLTTPVNGAVADGANSNSVQAVVTDSGGNPVNGAAVVFSSANATAQITTVIG  2448
   Yps7 --------------------------------------------------------------------------------  1486
   Yfr2 --------------------------------------------------------------------------------  906
   Yfr3 --------------------------------------------------------------------------------  851
   Yps2 --------------------------------------------------------------------------------  985
   Ype5 --------------------------------------------------------------------------------  1050
   Pru1 --------------------------------------------------------------------------------  2373
   Pal3 --------------------------------------------------------------------------------  542
   Eta1 --------------------------------------------------------------------------------  1400
   Sgl1 --------------------------------------------------------------------------------  934
  Eco26 --------------------------------------------------------------------------------  1653
   Pmi1 --------------------------------------------------------------------------------  2358
   Eco1 --------------------------------------------------------------------------------  937
   Ymo1 --------------------------------------------------------------------------------  1424
   Yen2 --------------------------------------------------------------------------------  2484
   Ybe1 --------------------------------------------------------------------------------  945
   Esa2 --------------------------------------------------------------------------------  1027
   Sen3 --------------------------------------------------------------------------------  1812
  Eco14 --------------------------------------------------------------------------------  1084
   Bpe1 --------------------------------------------------------------------------------  1308
   Bav2 --------------------------------------------------------------------------------  1654
   Bbr1 --------------------------------------------------------------------------------  969
   Bpa2 --------------------------------------------------------------------------------  1937
   Bav1 --------------------------------------------------------------------------------  747
   Pma1 --------------------------------------------------------------------------------  372
   Pma5 --------------------------------------------------------------------------------  373
   Pma3 --------------------------------------------------------------------------------  379
   Pma4 --------------------------------------------------------------------------------  410
   Ssp1 --------------------------------------------------------------------------------  428
   Ssp2 --------------------------------------------------------------------------------  436
  Eco20 --------------------------------------------------------------------------------  1418
   Efe5 --------------------------------------------------------------------------------  1185
   Plu1 --------------------------------------------------------------------------------  1695
   Pal2 --------------------------------------------------------------------------------  2521
   Ahy1 --------------------------------------------------------------------------------  916
   Csu1 --------------------------------------------------------------------------------  1305
   Plu2 --------------------------------------------------------------------------------  302
   Rba1 --------------------------------------------------------------------------------  327
   Pas1 --------------------------------------------------------------------------------  924
   Cla1 --------------------------------------------------------------------------------  1459
  ruler ......4890......4900......4910......4920......4930......4940......4950......4960
```

```
Mba1   --------------------------------------------------------------------------------   331
Psp2   --------------------------------------------------------------------------------   251
Sen2   --------------------------------------------------------------------------------   660
Efe4   --------------------------------------------------------------------------------   672
Eco3   --------------------------------------------------------------------------------   417
Cko1   --------------------------------------------------------------------------------   454
Sen1   --------------------------------------------------------------------------------   460
Eca1   --------------------------------------------------------------------------------   468
Esp2   --------------------------------------------------------------------------------   468
Esa3   --------------------------------------------------------------------------------   471
Kpn1   --------------------------------------------------------------------------------   460
Pan1   --------------------------------------------------------------------------------   490
Eta2   --------------------------------------------------------------------------------   480
Spr1   --------------------------------------------------------------------------------   497
Yin1   --------------------------------------------------------------------------------   751
Yin2   --------------------------------------------------------------------------------   753
Yfr4   --------------------------------------------------------------------------------   749
Ymo2   --------------------------------------------------------------------------------   686
Eco10  --------------------------------------------------------------------------------   734
Eal1   --------------------------------------------------------------------------------   725
Sty4   --------------------------------------------------------------------------------   626
Eco16  --------------------------------------------------------------------------------   711
Eco25  --------------------------------------------------------------------------------  2660
Eco15  --------------------------------------------------------------------------------  1700
Eco6   --------------------------------------------------------------------------------  2836
Efe2   --------------------------------------------------------------------------------  1089
Efe3   --------------------------------------------------------------------------------  2104
Yfr1   --------------------------------------------------------------------------------   770
Yfr5   --------------------------------------------------------------------------------   780
Yps4   TTGADGIATATLTNTVAGTSNVVATVDTVNANIDTTFVAGAVATITLTTPVNGAVADGADSNSVQAVVSDSGGNPVAGAA  2528
Yps7   --------------------------------------------------------------------------------  1486
Yfr2   --------------------------------------------------------------------------------   906
Yfr3   --------------------------------------------------------------------------------   851
Yps2   --------------------------------------------------------------------------------   985
Ype5   --------------------------------------------------------------------------------  1050
Pru1   --------------------------------------------------------------------------------  2373
Pal3   --------------------------------------------------------------------------------   542
Eta1   --------------------------------------------------------------------------------  1400
Sgl1   --------------------------------------------------------------------------------   934
Eco26  --------------------------------------------------------------------------------  1653
Pmi1   --------------------------------------------------------------------------------  2358
Eco1   --------------------------------------------------------------------------------   937
Ymo1   --------------------------------------------------------------------------------  1424
Yen2   --------------------------------------------------------------------------------  2484
Ybe1   --------------------------------------------------------------------------------   945
Esa2   --------------------------------------------------------------------------------  1027
Sen3   --------------------------------------------------------------------------------  1812
Eco14  --------------------------------------------------------------------------------  1084
Bpe1   --------------------------------------------------------------------------------  1308
Bav2   --------------------------------------------------------------------------------  1654
Bbr1   --------------------------------------------------------------------------------   969
Bpa2   --------------------------------------------------------------------------------  1937
Bav1   --------------------------------------------------------------------------------   747
Pma1   --------------------------------------------------------------------------------   372
Pma5   --------------------------------------------------------------------------------   373
Pma3   --------------------------------------------------------------------------------   379
Pma4   --------------------------------------------------------------------------------   410
Ssp1   --------------------------------------------------------------------------------   428
Ssp2   --------------------------------------------------------------------------------   436
Eco20  --------------------------------------------------------------------------------  1418
Efe5   --------------------------------------------------------------------------------  1185
Plu1   --------------------------------------------------------------------------------  1695
Pal2   --------------------------------------------------------------------------------  2521
Ahy1   --------------------------------------------------------------------------------   916
Csu1   --------------------------------------------------------------------------------  1305
Plu2   --------------------------------------------------------------------------------   302
Rba1   --------------------------------------------------------------------------------   327
Pas1   --------------------------------------------------------------------------------   924
Cla1   --------------------------------------------------------------------------------  1459
ruler  ......4970......4980......4990......5000......5010......5020......5030......5040
```

```
Mba1   --------------------------------------------------------------------------------   331
Psp2   --------------------------------------------------------------------------------   251
Sen2   --------------------------------------------------------------------------------   660
Efe4   --------------------------------------------------------------------------------   672
Eco3   --------------------------------------------------------------------------------   417
Cko1   --------------------------------------------------------------------------------   454
Sen1   --------------------------------------------------------------------------------   460
Eca1   --------------------------------------------------------------------------------   468
Esp2   --------------------------------------------------------------------------------   468
Esa3   --------------------------------------------------------------------------------   471
Kpn1   --------------------------------------------------------------------------------   460
Pan1   --------------------------------------------------------------------------------   490
Eta2   --------------------------------------------------------------------------------   480
Spr1   --------------------------------------------------------------------------------   497
Yin1   --------------------------------------------------------------------------------   751
Yin2   --------------------------------------------------------------------------------   753
Yfr4   --------------------------------------------------------------------------------   749
Ymo2   --------------------------------------------------------------------------------   686
Eco10  --------------------------------------------------------------------------------   734
Eal1   --------------------------------------------------------------------------------   725
Sty4   --------------------------------------------------------------------------------   626
Eco16  --------------------------------------------------------------------------------   711
Eco25  --------------------------------------------------------------------------------  2660
Eco15  --------------------------------------------------------------------------------  1700
Eco6   --------------------------------------------------------------------------------  2836
Efe2   --------------------------------------------------------------------------------  1089
Efe3   --------------------------------------------------------------------------------  2104
Yfr1   --------------------------------------------------------------------------------   770
Yfr5   --------------------------------------------------------------------------------   780
Yps4   VVFSSANATAQVTTVIGTTGADGIATATLTNTVAGTSNVVATIGSITNNIDTAFVAGAVATITLTTPVNGAVADGADSNS  2608
Yps7   --------------------------------------------------------------------------------  1486
Yfr2   --------------------------------------------------------------------------------   906
Yfr3   --------------------------------------------------------------------------------   851
Yps2   --------------------------------------------------------------------------------   985
Ype5   --------------------------------------------------------------------------------  1050
Pru1   --------------------------------------------------------------------------------  2373
Pal3   --------------------------------------------------------------------------------   542
Eta1   --------------------------------------------------------------------------------  1400
Sgl1   --------------------------------------------------------------------------------   934
Eco26  --------------------------------------------------------------------------------  1653
Pmi1   --------------------------------------------------------------------------------  2358
Eco1   --------------------------------------------------------------------------------   937
Ymo1   --------------------------------------------------------------------------------  1424
Yen2   --------------------------------------------------------------------------------  2484
Ybe1   --------------------------------------------------------------------------------   945
Esa2   --------------------------------------------------------------------------------  1027
Sen3   --------------------------------------------------------------------------------  1812
Eco14  --------------------------------------------------------------------------------  1084
Bpe1   --------------------------------------------------------------------------------  1308
Bav2   --------------------------------------------------------------------------------  1654
Bbr1   --------------------------------------------------------------------------------   969
Bpa2   --------------------------------------------------------------------------------  1937
Bav1   --------------------------------------------------------------------------------   747
Pma1   --------------------------------------------------------------------------------   372
Pma5   --------------------------------------------------------------------------------   373
Pma3   --------------------------------------------------------------------------------   379
Pma4   --------------------------------------------------------------------------------   410
Ssp1   --------------------------------------------------------------------------------   428
Ssp2   --------------------------------------------------------------------------------   436
Eco20  --------------------------------------------------------------------------------  1418
Efe5   --------------------------------------------------------------------------------  1185
Plu1   --------------------------------------------------------------------------------  1695
Pal2   --------------------------------------------------------------------------------  2521
Ahy1   --------------------------------------------------------------------------------   916
Csu1   --------------------------------------------------------------------------------  1305
Plu2   --------------------------------------------------------------------------------   302
Rba1   --------------------------------------------------------------------------------   327
Pas1   --------------------------------------------------------------------------------   924
Cla1   --------------------------------------------------------------------------------  1459
ruler  ......5050......5060......5070......5080......5090......5100......5110......5120
```

```
  Mba1 --------------------------------------------------------------------------------  331
  Psp2 --------------------------------------------------------------------------------  251
  Sen2 --------------------------------------------------------------------------------  660
  Efe4 --------------------------------------------------------------------------------  672
  Eco3 --------------------------------------------------------------------------------  417
  Cko1 --------------------------------------------------------------------------------  454
  Sen1 --------------------------------------------------------------------------------  460
  Eca1 --------------------------------------------------------------------------------  468
  Esp2 --------------------------------------------------------------------------------  468
  Esa3 --------------------------------------------------------------------------------  471
  Kpn1 --------------------------------------------------------------------------------  460
  Pan1 --------------------------------------------------------------------------------  490
  Eta2 --------------------------------------------------------------------------------  480
  Spr1 --------------------------------------------------------------------------------  497
  Yin1 --------------------------------------------------------------------------------  751
  Yin2 --------------------------------------------------------------------------------  753
  Yfr4 --------------------------------------------------------------------------------  749
  Ymo2 --------------------------------------------------------------------------------  686
 Eco10 --------------------------------------------------------------------------------  734
  Eal1 --------------------------------------------------------------------------------  725
  Sty4 --------------------------------------------------------------------------------  626
 Eco16 --------------------------------------------------------------------------------  711
 Eco25 -------------------------------------------------------------------------------- 2660
 Eco15 -------------------------------------------------------------------------------- 1700
  Eco6 -------------------------------------------------------------------------------- 2836
  Efe2 -------------------------------------------------------------------------------- 1089
  Efe3 -------------------------------------------------------------------------------- 2104
  Yfr1 --------------------------------------------------------------------------------  770
  Yfr5 --------------------------------------------------------------------------------  780
  Yps4 VQAVVSDSEGNAVTGAAVVFSSANATAQITTVIGTTGADGIATATLTNTVAGTSNVVATIGGITNNIDTAFVAGAVATIT 2688
  Yps7 -------------------------------------------------------------------------------- 1486
  Yfr2 --------------------------------------------------------------------------------  906
  Yfr3 --------------------------------------------------------------------------------  851
  Yps2 --------------------------------------------------------------------------------  985
  Ype5 -------------------------------------------------------------------------------- 1050
  Pru1 -------------------------------------------------------------------------------- 2373
  Pal3 --------------------------------------------------------------------------------  542
  Eta1 -------------------------------------------------------------------------------- 1400
  Sgl1 --------------------------------------------------------------------------------  934
 Eco26 -------------------------------------------------------------------------------- 1653
  Pmi1 -------------------------------------------------------------------------------- 2358
  Eco1 --------------------------------------------------------------------------------  937
  Ymo1 -------------------------------------------------------------------------------- 1424
  Yen2 -------------------------------------------------------------------------------- 2484
  Ybe1 --------------------------------------------------------------------------------  945
  Esa2 -------------------------------------------------------------------------------- 1027
  Sen3 -------------------------------------------------------------------------------- 1812
 Eco14 -------------------------------------------------------------------------------- 1084
  Bpe1 -------------------------------------------------------------------------------- 1308
  Bav2 -------------------------------------------------------------------------------- 1654
  Bbr1 --------------------------------------------------------------------------------  969
  Bpa2 -------------------------------------------------------------------------------- 1937
  Bav1 --------------------------------------------------------------------------------  747
  Pma1 --------------------------------------------------------------------------------  372
  Pma5 --------------------------------------------------------------------------------  373
  Pma3 --------------------------------------------------------------------------------  379
  Pma4 --------------------------------------------------------------------------------  410
  Ssp1 --------------------------------------------------------------------------------  428
  Ssp2 --------------------------------------------------------------------------------  436
 Eco20 -------------------------------------------------------------------------------- 1418
  Efe5 -------------------------------------------------------------------------------- 1185
  Plu1 -------------------------------------------------------------------------------- 1695
  Pal2 -------------------------------------------------------------------------------- 2521
  Ahy1 --------------------------------------------------------------------------------  916
  Csu1 -------------------------------------------------------------------------------- 1305
  Plu2 --------------------------------------------------------------------------------  302
  Rba1 --------------------------------------------------------------------------------  327
  Pas1 --------------------------------------------------------------------------------  924
  Cla1 -------------------------------------------------------------------------------- 1459
 ruler ......5130......5140......5150......5160......5170......5180......5190......5200
```

```
Mba1   --------------------------------------------------------------------------------   331
Psp2   --------------------------------------------------------------------------------   251
Sen2   --------------------------------------------------------------------------------   660
Efe4   --------------------------------------------------------------------------------   672
Eco3   --------------------------------------------------------------------------------   417
Cko1   --------------------------------------------------------------------------------   454
Sen1   --------------------------------------------------------------------------------   460
Eca1   --------------------------------------------------------------------------------   468
Esp2   --------------------------------------------------------------------------------   468
Esa3   --------------------------------------------------------------------------------   471
Kpn1   --------------------------------------------------------------------------------   460
Pan1   --------------------------------------------------------------------------------   490
Eta2   --------------------------------------------------------------------------------   480
Spr1   --------------------------------------------------------------------------------   497
Yin1   --------------------------------------------------------------------------------   751
Yin2   --------------------------------------------------------------------------------   753
Yfr4   --------------------------------------------------------------------------------   749
Ymo2   --------------------------------------------------------------------------------   686
Eco10  --------------------------------------------------------------------------------   734
Eal1   --------------------------------------------------------------------------------   725
Sty4   --------------------------------------------------------------------------------   626
Eco16  --------------------------------------------------------------------------------   711
Eco25  --------------------------------------------------------------------------------  2660
Eco15  --------------------------------------------------------------------------------  1700
Eco6   --------------------------------------------------------------------------------  2836
Efe2   --------------------------------------------------------------------------------  1089
Efe3   --------------------------------------------------------------------------------  2104
Yfr1   --------------------------------------------------------------------------------   770
Yfr5   --------------------------------------------------------------------------------   780
Yps4   LTTPVNGAVADGTDSNSVQAVVSDSEGNAVAGAAVVFSSANATAQITTVIGTTGADGIATATLTNTVAGTSNVVATIGSI  2768
Yps7   --------------------------------------------------------------------------------  1486
Yfr2   --------------------------------------------------------------------------------   906
Yfr3   --------------------------------------------------------------------------------   851
Yps2   --------------------------------------------------------------------------------   985
Ype5   --------------------------------------------------------------------------------  1050
Pru1   --------------------------------------------------------------------------------  2373
Pal3   --------------------------------------------------------------------------------   542
Eta1   --------------------------------------------------------------------------------  1400
Sgl1   --------------------------------------------------------------------------------   934
Eco26  --------------------------------------------------------------------------------  1653
Pmi1   --------------------------------------------------------------------------------  2358
Eco1   --------------------------------------------------------------------------------   937
Ymo1   --------------------------------------------------------------------------------  1424
Yen2   --------------------------------------------------------------------------------  2484
Ybe1   --------------------------------------------------------------------------------   945
Esa2   --------------------------------------------------------------------------------  1027
Sen3   --------------------------------------------------------------------------------  1812
Eco14  --------------------------------------------------------------------------------  1084
Bpe1   --------------------------------------------------------------------------------  1308
Bav2   --------------------------------------------------------------------------------  1654
Bbr1   --------------------------------------------------------------------------------   969
Bpa2   --------------------------------------------------------------------------------  1937
Bav1   --------------------------------------------------------------------------------   747
Pma1   --------------------------------------------------------------------------------   372
Pma5   --------------------------------------------------------------------------------   373
Pma3   --------------------------------------------------------------------------------   379
Pma4   --------------------------------------------------------------------------------   410
Ssp1   --------------------------------------------------------------------------------   428
Ssp2   --------------------------------------------------------------------------------   436
Eco20  --------------------------------------------------------------------------------  1418
Efe5   --------------------------------------------------------------------------------  1185
Plu1   --------------------------------------------------------------------------------  1695
Pal2   --------------------------------------------------------------------------------  2521
Ahy1   --------------------------------------------------------------------------------   916
Csu1   --------------------------------------------------------------------------------  1305
Plu2   --------------------------------------------------------------------------------   302
Rba1   --------------------------------------------------------------------------------   327
Pas1   --------------------------------------------------------------------------------   924
Cla1   --------------------------------------------------------------------------------  1459
ruler  ......5210......5220......5230......5240......5250......5260......5270......5280
```

```
Mba1   --------------------------------------------------------------------------------  331
Psp2   --------------------------------------------------------------------------------  251
Sen2   --------------------------------------------------------------------------------  660
Efe4   --------------------------------------------------------------------------------  672
Eco3   --------------------------------------------------------------------------------  417
Cko1   --------------------------------------------------------------------------------  454
Sen1   --------------------------------------------------------------------------------  460
Eca1   --------------------------------------------------------------------------------  468
Esp2   --------------------------------------------------------------------------------  468
Esa3   --------------------------------------------------------------------------------  471
Kpn1   --------------------------------------------------------------------------------  460
Pan1   --------------------------------------------------------------------------------  490
Eta2   --------------------------------------------------------------------------------  480
Spr1   --------------------------------------------------------------------------------  497
Yin1   --------------------------------------------------------------------------------  751
Yin2   --------------------------------------------------------------------------------  753
Yfr4   --------------------------------------------------------------------------------  749
Ymo2   --------------------------------------------------------------------------------  686
Eco10  --------------------------------------------------------------------------------  734
Eal1   --------------------------------------------------------------------------------  725
Sty4   --------------------------------------------------------------------------------  626
Eco16  --------------------------------------------------------------------------------  711
Eco25  --------------------------------------------------------------------------------  2660
Eco15  --------------------------------------------------------------------------------  1700
Eco6   --------------------------------------------------------------------------------  2836
Efe2   --------------------------------------------------------------------------------  1089
Efe3   --------------------------------------------------------------------------------  2104
Yfr1   --------------------------------------------------------------------------------  770
Yfr5   --------------------------------------------------------------------------------  780
Yps4   TNNIDTAFVAGAVATITLTTLVNGAVANGADSNSVQAVVSDSGGNVVAGATVVFSSTNATAQVTTVIGTTGADGIATATL  2848
Yps7   --------------------------------------------------------------------------------  1486
Yfr2   --------------------------------------------------------------------------------  906
Yfr3   --------------------------------------------------------------------------------  851
Yps2   --------------------------------------------------------------------------------  985
Ype5   --------------------------------------------------------------------------------  1050
Pru1   --------------------------------------------------------------------------------  2373
Pal3   --------------------------------------------------------------------------------  542
Eta1   --------------------------------------------------------------------------------  1400
Sgl1   --------------------------------------------------------------------------------  934
Eco26  --------------------------------------------------------------------------------  1653
Pmi1   --------------------------------------------------------------------------------  2358
Eco1   --------------------------------------------------------------------------------  937
Ymo1   --------------------------------------------------------------------------------  1424
Yen2   --------------------------------------------------------------------------------  2484
Ybe1   --------------------------------------------------------------------------------  945
Esa2   --------------------------------------------------------------------------------  1027
Sen3   --------------------------------------------------------------------------------  1812
Eco14  --------------------------------------------------------------------------------  1084
Bpe1   --------------------------------------------------------------------------------  1308
Bav2   --------------------------------------------------------------------------------  1654
Bbr1   --------------------------------------------------------------------------------  969
Bpa2   --------------------------------------------------------------------------------  1937
Bav1   --------------------------------------------------------------------------------  747
Pma1   --------------------------------------------------------------------------------  372
Pma5   --------------------------------------------------------------------------------  373
Pma3   --------------------------------------------------------------------------------  379
Pma4   --------------------------------------------------------------------------------  410
Ssp1   --------------------------------------------------------------------------------  428
Ssp2   --------------------------------------------------------------------------------  436
Eco20  --------------------------------------------------------------------------------  1418
Efe5   --------------------------------------------------------------------------------  1185
Plu1   --------------------------------------------------------------------------------  1695
Pal2   --------------------------------------------------------------------------------  2521
Ahy1   --------------------------------------------------------------------------------  916
Csu1   --------------------------------------------------------------------------------  1305
Plu2   --------------------------------------------------------------------------------  302
Rba1   --------------------------------------------------------------------------------  327
Pas1   --------------------------------------------------------------------------------  924
Cla1   --------------------------------------------------------------------------------  1459
ruler  ......5290......5300......5310......5320......5330......5340......5350......5360
```

```
Mba1   --------------------------------------------------------------------------------   331
Psp2   --------------------------------------------------------------------------------   251
Sen2   --------------------------------------------------------------------------------   660
Efe4   --------------------------------------------------------------------------------   672
Eco3   --------------------------------------------------------------------------------   417
Cko1   --------------------------------------------------------------------------------   454
Sen1   --------------------------------------------------------------------------------   460
Eca1   --------------------------------------------------------------------------------   468
Esp2   --------------------------------------------------------------------------------   468
Esa3   --------------------------------------------------------------------------------   471
Kpn1   --------------------------------------------------------------------------------   460
Pan1   --------------------------------------------------------------------------------   490
Eta2   --------------------------------------------------------------------------------   480
Spr1   --------------------------------------------------------------------------------   497
Yin1   --------------------------------------------------------------------------------   751
Yin2   --------------------------------------------------------------------------------   753
Yfr4   --------------------------------------------------------------------------------   749
Ymo2   --------------------------------------------------------------------------------   686
Eco10  --------------------------------------------------------------------------------   734
Eal1   --------------------------------------------------------------------------------   725
Sty4   --------------------------------------------------------------------------------   626
Eco16  --------------------------------------------------------------------------------   711
Eco25  --------------------------------------------------------------------------------  2660
Eco15  --------------------------------------------------------------------------------  1700
Eco6   --------------------------------------------------------------------------------  2836
Efe2   --------------------------------------------------------------------------------  1089
Efe3   --------------------------------------------------------------------------------  2104
Yfr1   --------------------------------------------------------------------------------   770
Yfr5   --------------------------------------------------------------------------------   780
Yps4   TNTVAGTSNVVATIDTVNANIDTTFVAGAVATITLSVLVNDATADGADTNQVDALVQDANGNAITGAAVVFSSANGATIL  2928
Yps7   --------------------------------------------------------------------------------  1486
Yfr2   --------------------------------------------------------------------------------   906
Yfr3   --------------------------------------------------------------------------------   851
Yps2   --------------------------------------------------------------------------------   985
Ype5   --------------------------------------------------------------------------------  1050
Pru1   --------------------------------------------------------------------------------  2373
Pal3   --------------------------------------------------------------------------------   542
Eta1   --------------------------------------------------------------------------------  1400
Sgl1   --------------------------------------------------------------------------------   934
Eco26  --------------------------------------------------------------------------------  1653
Pmi1   --------------------------------------------------------------------------------  2358
Eco1   --------------------------------------------------------------------------------   937
Ymo1   --------------------------------------------------------------------------------  1424
Yen2   --------------------------------------------------------------------------------  2484
Ybe1   --------------------------------------------------------------------------------   945
Esa2   --------------------------------------------------------------------------------  1027
Sen3   --------------------------------------------------------------------------------  1812
Eco14  --------------------------------------------------------------------------------  1084
Bpe1   --------------------------------------------------------------------------------  1308
Bav2   --------------------------------------------------------------------------------  1654
Bbr1   --------------------------------------------------------------------------------   969
Bpa2   --------------------------------------------------------------------------------  1937
Bav1   --------------------------------------------------------------------------------   747
Pma1   --------------------------------------------------------------------------------   372
Pma5   --------------------------------------------------------------------------------   373
Pma3   --------------------------------------------------------------------------------   379
Pma4   --------------------------------------------------------------------------------   410
Ssp1   --------------------------------------------------------------------------------   428
Ssp2   --------------------------------------------------------------------------------   436
Eco20  --------------------------------------------------------------------------------  1418
Efe5   --------------------------------------------------------------------------------  1185
Plu1   --------------------------------------------------------------------------------  1695
Pal2   --------------------------------------------------------------------------------  2521
Ahy1   --------------------------------------------------------------------------------   916
Csu1   --------------------------------------------------------------------------------  1305
Plu2   --------------------------------------------------------------------------------   302
Rba1   --------------------------------------------------------------------------------   327
Pas1   --------------------------------------------------------------------------------   924
Cla1   --------------------------------------------------------------------------------  1459
ruler  ......5370......5380......5390......5400......5410......5420......5430......5440
```

```
  Mba1 --------------------------------------------------------------------------------  331
  Psp2 --------------------------------------------------------------------------------  251
  Sen2 --------------------------------------------------------------------------------  660
  Efe4 --------------------------------------------------------------------------------  672
  Eco3 --------------------------------------------------------------------------------  417
  Cko1 --------------------------------------------------------------------------------  454
  Sen1 --------------------------------------------------------------------------------  460
  Eca1 --------------------------------------------------------------------------------  468
  Esp2 --------------------------------------------------------------------------------  468
  Esa3 --------------------------------------------------------------------------------  471
  Kpn1 --------------------------------------------------------------------------------  460
  Pan1 --------------------------------------------------------------------------------  490
  Eta2 --------------------------------------------------------------------------------  480
  Spr1 --------------------------------------------------------------------------------  497
  Yin1 --------------------------------------------------------------------------------  751
  Yin2 --------------------------------------------------------------------------------  753
  Yfr4 --------------------------------------------------------------------------------  749
  Ymo2 --------------------------------------------------------------------------------  686
 Eco10 --------------------------------------------------------------------------------  734
  Eal1 --------------------------------------------------------------------------------  725
  Sty4 --------------------------------------------------------------------------------  626
 Eco16 --------------------------------------------------------------------------------  711
 Eco25 -------------------------------------------------------------------------------- 2660
 Eco15 -------------------------------------------------------------------------------- 1700
  Eco6 -------------------------------------------------------------------------------- 2836
  Efe2 -------------------------------------------------------------------------------- 1089
  Efe3 -------------------------------------------------------------------------------- 2104
  Yfr1 --------------------------------------------------------------------------------  770
  Yfr5 --------------------------------------------------------------------------------  780
  Yps4 SSTMNTGVNGVASTLLTHTVAGTSNVVATIDTVNANIDTAFVAGAVATITLTTPVNGAVANGADSNSVQAVVSDSEGNAV 3008
  Yps7 -------------------------------------------------------------------------------- 1486
  Yfr2 --------------------------------------------------------------------------------  906
  Yfr3 --------------------------------------------------------------------------------  851
  Yps2 --------------------------------------------------------------------------------  985
  Ype5 -------------------------------------------------------------------------------- 1050
  Pru1 -------------------------------------------------------------------------------- 2373
  Pal3 --------------------------------------------------------------------------------  542
  Eta1 -------------------------------------------------------------------------------- 1400
  Sgl1 --------------------------------------------------------------------------------  934
 Eco26 -------------------------------------------------------------------------------- 1653
  Pmi1 -------------------------------------------------------------------------------- 2358
  Eco1 --------------------------------------------------------------------------------  937
  Ymo1 -------------------------------------------------------------------------------- 1424
  Yen2 -------------------------------------------------------------------------------- 2484
  Ybe1 --------------------------------------------------------------------------------  945
  Esa2 -------------------------------------------------------------------------------- 1027
  Sen3 -------------------------------------------------------------------------------- 1812
 Eco14 -------------------------------------------------------------------------------- 1084
  Bpe1 -------------------------------------------------------------------------------- 1308
  Bav2 -------------------------------------------------------------------------------- 1654
  Bbr1 --------------------------------------------------------------------------------  969
  Bpa2 -------------------------------------------------------------------------------- 1937
  Bav1 --------------------------------------------------------------------------------  747
  Pma1 --------------------------------------------------------------------------------  372
  Pma5 --------------------------------------------------------------------------------  373
  Pma3 --------------------------------------------------------------------------------  379
  Pma4 --------------------------------------------------------------------------------  410
  Ssp1 --------------------------------------------------------------------------------  428
  Ssp2 --------------------------------------------------------------------------------  436
 Eco20 -------------------------------------------------------------------------------- 1418
  Efe5 -------------------------------------------------------------------------------- 1185
  Plu1 -------------------------------------------------------------------------------- 1695
  Pal2 -------------------------------------------------------------------------------- 2521
  Ahy1 --------------------------------------------------------------------------------  916
  Csu1 -------------------------------------------------------------------------------- 1305
  Plu2 --------------------------------------------------------------------------------  302
  Rba1 --------------------------------------------------------------------------------  327
  Pas1 --------------------------------------------------------------------------------  924
  Cla1 -------------------------------------------------------------------------------- 1459
 ruler ......5450......5460......5470......5480......5490......5500......5510......5520
```

```
  Mba1 --------------------------------------------------------------------------------  331
  Psp2 --------------------------------------------------------------------------------  251
  Sen2 --------------------------------------------------------------------------------  660
  Efe4 --------------------------------------------------------------------------------  672
  Eco3 --------------------------------------------------------------------------------  417
  Cko1 --------------------------------------------------------------------------------  454
  Sen1 --------------------------------------------------------------------------------  460
  Eca1 --------------------------------------------------------------------------------  468
  Esp2 --------------------------------------------------------------------------------  468
  Esa3 --------------------------------------------------------------------------------  471
  Kpn1 --------------------------------------------------------------------------------  460
  Pan1 --------------------------------------------------------------------------------  490
  Eta2 --------------------------------------------------------------------------------  480
  Spr1 --------------------------------------------------------------------------------  497
  Yin1 --------------------------------------------------------------------------------  751
  Yin2 --------------------------------------------------------------------------------  753
  Yfr4 --------------------------------------------------------------------------------  749
  Ymo2 --------------------------------------------------------------------------------  686
 Eco10 --------------------------------------------------------------------------------  734
  Eal1 --------------------------------------------------------------------------------  725
  Sty4 --------------------------------------------------------------------------------  626
 Eco16 --------------------------------------------------------------------------------  711
 Eco25 --------------------------------------------------------------------------------  2660
 Eco15 --------------------------------------------------------------------------------  1700
  Eco6 --------------------------------------------------------------------------------  2836
  Efe2 --------------------------------------------------------------------------------  1089
  Efe3 --------------------------------------------------------------------------------  2104
  Yfr1 --------------------------------------------------------------------------------  770
  Yfr5 --------------------------------------------------------------------------------  780
  Yps4 AGAAVVFSSANATAQITTVIGTTGVDGIATATLTNTVAGTSNVVATVDTVNANIDTAFVAGAVATITLTTPVNGAVANGA  3088
  Yps7 --------------------------------------------------------------------------------  1486
  Yfr2 --------------------------------------------------------------------------------  906
  Yfr3 --------------------------------------------------------------------------------  851
  Yps2 --------------------------------------------------------------------------------  985
  Ype5 --------------------------------------------------------------------------------  1050
  Pru1 --------------------------------------------------------------------------------  2373
  Pal3 --------------------------------------------------------------------------------  542
  Eta1 --------------------------------------------------------------------------------  1400
  Sgl1 --------------------------------------------------------------------------------  934
 Eco26 --------------------------------------------------------------------------------  1653
  Pmi1 --------------------------------------------------------------------------------  2358
  Eco1 --------------------------------------------------------------------------------  937
  Ymo1 --------------------------------------------------------------------------------  1424
  Yen2 --------------------------------------------------------------------------------  2484
  Ybe1 --------------------------------------------------------------------------------  945
  Esa2 --------------------------------------------------------------------------------  1027
  Sen3 --------------------------------------------------------------------------------  1812
 Eco14 --------------------------------------------------------------------------------  1084
  Bpe1 --------------------------------------------------------------------------------  1308
  Bav2 --------------------------------------------------------------------------------  1654
  Bbr1 --------------------------------------------------------------------------------  969
  Bpa2 --------------------------------------------------------------------------------  1937
  Bav1 --------------------------------------------------------------------------------  747
  Pma1 --------------------------------------------------------------------------------  372
  Pma5 --------------------------------------------------------------------------------  373
  Pma3 --------------------------------------------------------------------------------  379
  Pma4 --------------------------------------------------------------------------------  410
  Ssp1 --------------------------------------------------------------------------------  428
  Ssp2 --------------------------------------------------------------------------------  436
 Eco20 --------------------------------------------------------------------------------  1418
  Efe5 --------------------------------------------------------------------------------  1185
  Plu1 --------------------------------------------------------------------------------  1695
  Pal2 --------------------------------------------------------------------------------  2521
  Ahy1 --------------------------------------------------------------------------------  916
  Csu1 --------------------------------------------------------------------------------  1305
  Plu2 --------------------------------------------------------------------------------  302
  Rba1 --------------------------------------------------------------------------------  327
  Pas1 --------------------------------------------------------------------------------  924
  Cla1 --------------------------------------------------------------------------------  1459
 ruler ......5530......5540......5550......5560......5570......5580......5590......5600
```

```
Mba1   --------------------------------------------------------------------------------   331
Psp2   --------------------------------------------------------------------------------   251
Sen2   --------------------------------------------------------------------------------   660
Efe4   --------------------------------------------------------------------------------   672
Eco3   --------------------------------------------------------------------------------   417
Cko1   --------------------------------------------------------------------------------   454
Sen1   --------------------------------------------------------------------------------   460
Eca1   --------------------------------------------------------------------------------   468
Esp2   --------------------------------------------------------------------------------   468
Esa3   --------------------------------------------------------------------------------   471
Kpn1   --------------------------------------------------------------------------------   460
Pan1   --------------------------------------------------------------------------------   490
Eta2   --------------------------------------------------------------------------------   480
Spr1   --------------------------------------------------------------------------------   497
Yin1   --------------------------------------------------------------------------------   751
Yin2   --------------------------------------------------------------------------------   753
Yfr4   --------------------------------------------------------------------------------   749
Ymo2   --------------------------------------------------------------------------------   686
Eco10  --------------------------------------------------------------------------------   734
Eal1   --------------------------------------------------------------------------------   725
Sty4   --------------------------------------------------------------------------------   626
Eco16  --------------------------------------------------------------------------------   711
Eco25  --------------------------------------------------------------------------------   2660
Eco15  --------------------------------------------------------------------------------   1700
Eco6   --------------------------------------------------------------------------------   2836
Efe2   --------------------------------------------------------------------------------   1089
Efe3   --------------------------------------------------------------------------------   2104
Yfr1   --------------------------------------------------------------------------------   770
Yfr5   --------------------------------------------------------------------------------   780
Yps4   DSNSVQAVVSDSGGNVVAGATVVFSSTNTTAQVTTVIGTTGADGIATATLTNTVAGTSNVVATVDTVNANIDTTFVAGAV   3168
Yps7   --------------------------------------------------------------------------------   1486
Yfr2   --------------------------------------------------------------------------------   906
Yfr3   --------------------------------------------------------------------------------   851
Yps2   --------------------------------------------------------------------------------   985
Ype5   --------------------------------------------------------------------------------   1050
Pru1   --------------------------------------------------------------------------------   2373
Pal3   --------------------------------------------------------------------------------   542
Eta1   --------------------------------------------------------------------------------   1400
Sgl1   --------------------------------------------------------------------------------   934
Eco26  --------------------------------------------------------------------------------   1653
Pmi1   --------------------------------------------------------------------------------   2358
Eco1   --------------------------------------------------------------------------------   937
Ymo1   --------------------------------------------------------------------------------   1424
Yen2   --------------------------------------------------------------------------------   2484
Ybe1   --------------------------------------------------------------------------------   945
Esa2   --------------------------------------------------------------------------------   1027
Sen3   --------------------------------------------------------------------------------   1812
Eco14  --------------------------------------------------------------------------------   1084
Bpe1   --------------------------------------------------------------------------------   1308
Bav2   --------------------------------------------------------------------------------   1654
Bbr1   --------------------------------------------------------------------------------   969
Bpa2   --------------------------------------------------------------------------------   1937
Bav1   --------------------------------------------------------------------------------   747
Pma1   --------------------------------------------------------------------------------   372
Pma5   --------------------------------------------------------------------------------   373
Pma3   --------------------------------------------------------------------------------   379
Pma4   --------------------------------------------------------------------------------   410
Ssp1   --------------------------------------------------------------------------------   428
Ssp2   --------------------------------------------------------------------------------   436
Eco20  --------------------------------------------------------------------------------   1418
Efe5   --------------------------------------------------------------------------------   1185
Plu1   --------------------------------------------------------------------------------   1695
Pal2   --------------------------------------------------------------------------------   2521
Ahy1   --------------------------------------------------------------------------------   916
Csu1   --------------------------------------------------------------------------------   1305
Plu2   --------------------------------------------------------------------------------   302
Rba1   --------------------------------------------------------------------------------   327
Pas1   --------------------------------------------------------------------------------   924
Cla1   --------------------------------------------------------------------------------   1459
ruler  ......5610......5620......5630......5640......5650......5660......5670......5680
```

```
   Mba1 --------------------------------------------------------------------------------  331
   Psp2 --------------------------------------------------------------------------------  251
   Sen2 --------------------------------------------------------------------------------  660
   Efe4 --------------------------------------------------------------------------------  672
   Eco3 --------------------------------------------------------------------------------  417
   Cko1 --------------------------------------------------------------------------------  454
   Sen1 --------------------------------------------------------------------------------  460
   Eca1 --------------------------------------------------------------------------------  468
   Esp2 --------------------------------------------------------------------------------  468
   Esa3 --------------------------------------------------------------------------------  471
   Kpn1 --------------------------------------------------------------------------------  460
   Pan1 --------------------------------------------------------------------------------  490
   Eta2 --------------------------------------------------------------------------------  480
   Spr1 --------------------------------------------------------------------------------  497
   Yin1 --------------------------------------------------------------------------------  751
   Yin2 --------------------------------------------------------------------------------  753
   Yfr4 --------------------------------------------------------------------------------  749
   Ymo2 --------------------------------------------------------------------------------  686
  Eco10 --------------------------------------------------------------------------------  734
   Eal1 --------------------------------------------------------------------------------  725
   Sty4 --------------------------------------------------------------------------------  626
  Eco16 --------------------------------------------------------------------------------  711
  Eco25 --------------------------------------------------------------------------------  2660
  Eco15 --------------------------------------------------------------------------------  1700
   Eco6 --------------------------------------------------------------------------------  2836
   Efe2 --------------------------------------------------------------------------------  1089
   Efe3 --------------------------------------------------------------------------------  2104
   Yfr1 --------------------------------------------------------------------------------  770
   Yfr5 --------------------------------------------------------------------------------  780
   Yps4 ATITLSVLVNDATADGADTNQVDALVQDANGNAITGAAVVFSSANGADIIAPTMNTGVNGVASTLLTHTMAGTSNVIATI  3248
   Yps7 --------------------------------------------------------------------------------  1486
   Yfr2 --------------------------------------------------------------------------------  906
   Yfr3 --------------------------------------------------------------------------------  851
   Yps2 --------------------------------------------------------------------------------  985
   Ype5 --------------------------------------------------------------------------------  1050
   Pru1 --------------------------------------------------------------------------------  2373
   Pal3 --------------------------------------------------------------------------------  542
   Eta1 --------------------------------------------------------------------------------  1400
   Sgl1 --------------------------------------------------------------------------------  934
  Eco26 --------------------------------------------------------------------------------  1653
   Pmi1 --------------------------------------------------------------------------------  2358
   Eco1 --------------------------------------------------------------------------------  937
   Ymo1 --------------------------------------------------------------------------------  1424
   Yen2 --------------------------------------------------------------------------------  2484
   Ybe1 --------------------------------------------------------------------------------  945
   Esa2 --------------------------------------------------------------------------------  1027
   Sen3 --------------------------------------------------------------------------------  1812
  Eco14 --------------------------------------------------------------------------------  1084
   Bpe1 --------------------------------------------------------------------------------  1308
   Bav2 --------------------------------------------------------------------------------  1654
   Bbr1 --------------------------------------------------------------------------------  969
   Bpa2 --------------------------------------------------------------------------------  1937
   Bav1 --------------------------------------------------------------------------------  747
   Pma1 --------------------------------------------------------------------------------  372
   Pma5 --------------------------------------------------------------------------------  373
   Pma3 --------------------------------------------------------------------------------  379
   Pma4 --------------------------------------------------------------------------------  410
   Ssp1 --------------------------------------------------------------------------------  428
   Ssp2 --------------------------------------------------------------------------------  436
  Eco20 --------------------------------------------------------------------------------  1418
   Efe5 --------------------------------------------------------------------------------  1185
   Plu1 --------------------------------------------------------------------------------  1695
   Pal2 --------------------------------------------------------------------------------  2521
   Ahy1 --------------------------------------------------------------------------------  916
   Csu1 --------------------------------------------------------------------------------  1305
   Plu2 --------------------------------------------------------------------------------  302
   Rba1 --------------------------------------------------------------------------------  327
   Pas1 --------------------------------------------------------------------------------  924
   Cla1 --------------------------------------------------------------------------------  1459
  ruler ......5690......5700......5710......5720......5730......5740......5750......5760
```

```
   Mba1 --------------------------------------------------------------------------------  331
   Psp2 --------------------------------------------------------------------------------  251
   Sen2 --------------------------------------------------------------------------------  660
   Efe4 --------------------------------------------------------------------------------  672
   Eco3 --------------------------------------------------------------------------------  417
   Cko1 --------------------------------------------------------------------------------  454
   Sen1 --------------------------------------------------------------------------------  460
   Eca1 --------------------------------------------------------------------------------  468
   Esp2 --------------------------------------------------------------------------------  468
   Esa3 --------------------------------------------------------------------------------  471
   Kpn1 --------------------------------------------------------------------------------  460
   Pan1 --------------------------------------------------------------------------------  490
   Eta2 --------------------------------------------------------------------------------  480
   Spr1 --------------------------------------------------------------------------------  497
   Yin1 --------------------------------------------------------------------------------  751
   Yin2 --------------------------------------------------------------------------------  753
   Yfr4 --------------------------------------------------------------------------------  749
   Ymo2 --------------------------------------------------------------------------------  686
  Eco10 --------------------------------------------------------------------------------  734
   Eal1 --------------------------------------------------------------------------------  725
   Sty4 --------------------------------------------------------------------------------  626
  Eco16 --------------------------------------------------------------------------------  711
  Eco25 -------------------------------------------------------------------------------- 2660
  Eco15 -------------------------------------------------------------------------------- 1700
   Eco6 -------------------------------------------------------------------------------- 2836
   Efe2 -------------------------------------------------------------------------------- 1089
   Efe3 -------------------------------------------------------------------------------- 2104
   Yfr1 --------------------------------------------------------------------------------  770
   Yfr5 --------------------------------------------------------------------------------  780
   Yps4 DTVNANIDTTFVAGAVATITLSVPVNDATADGADTNQVDALVQDANGNAITGAAVVFSSANGATILSSTMNTGVNGVAST 3328
   Yps7 -------------------------------------------------------------------------------- 1486
   Yfr2 --------------------------------------------------------------------------------  906
   Yfr3 --------------------------------------------------------------------------------  851
   Yps2 --------------------------------------------------------------------------------  985
   Ype5 -------------------------------------------------------------------------------- 1050
   Pru1 -------------------------------------------------------------------------------- 2373
   Pal3 --------------------------------------------------------------------------------  542
   Eta1 -------------------------------------------------------------------------------- 1400
   Sgl1 --------------------------------------------------------------------------------  934
  Eco26 -------------------------------------------------------------------------------- 1653
   Pmi1 -------------------------------------------------------------------------------- 2358
   Eco1 --------------------------------------------------------------------------------  937
   Ymo1 -------------------------------------------------------------------------------- 1424
   Yen2 -------------------------------------------------------------------------------- 2484
   Ybe1 --------------------------------------------------------------------------------  945
   Esa2 -------------------------------------------------------------------------------- 1027
   Sen3 -------------------------------------------------------------------------------- 1812
  Eco14 -------------------------------------------------------------------------------- 1084
   Bpe1 -------------------------------------------------------------------------------- 1308
   Bav2 -------------------------------------------------------------------------------- 1654
   Bbr1 --------------------------------------------------------------------------------  969
   Bpa2 -------------------------------------------------------------------------------- 1937
   Bav1 --------------------------------------------------------------------------------  747
   Pma1 --------------------------------------------------------------------------------  372
   Pma5 --------------------------------------------------------------------------------  373
   Pma3 --------------------------------------------------------------------------------  379
   Pma4 --------------------------------------------------------------------------------  410
   Ssp1 --------------------------------------------------------------------------------  428
   Ssp2 --------------------------------------------------------------------------------  436
  Eco20 -------------------------------------------------------------------------------- 1418
   Efe5 -------------------------------------------------------------------------------- 1185
   Plu1 -------------------------------------------------------------------------------- 1695
   Pal2 -------------------------------------------------------------------------------- 2521
   Ahy1 --------------------------------------------------------------------------------  916
   Csu1 -------------------------------------------------------------------------------- 1305
   Plu2 --------------------------------------------------------------------------------  302
   Rba1 --------------------------------------------------------------------------------  327
   Pas1 --------------------------------------------------------------------------------  924
   Cla1 -------------------------------------------------------------------------------- 1459
  ruler ......5770......5780......5790......5800......5810......5820......5830......5840
```

```
  Mba1 --------------------------------------------------------------------------------  331
  Psp2 --------------------------------------------------------------------------------  251
  Sen2 --------------------------------------------------------------------------------  660
  Efe4 --------------------------------------------------------------------------------  672
  Eco3 --------------------------------------------------------------------------------  417
  Cko1 --------------------------------------------------------------------------------  454
  Sen1 --------------------------------------------------------------------------------  460
  Eca1 --------------------------------------------------------------------------------  468
  Esp2 --------------------------------------------------------------------------------  468
  Esa3 --------------------------------------------------------------------------------  471
  Kpn1 --------------------------------------------------------------------------------  460
  Pan1 --------------------------------------------------------------------------------  490
  Eta2 --------------------------------------------------------------------------------  480
  Spr1 --------------------------------------------------------------------------------  497
  Yin1 --------------------------------------------------------------------------------  751
  Yin2 --------------------------------------------------------------------------------  753
  Yfr4 --------------------------------------------------------------------------------  749
  Ymo2 --------------------------------------------------------------------------------  686
 Eco10 --------------------------------------------------------------------------------  734
  Eal1 --------------------------------------------------------------------------------  725
  Sty4 --------------------------------------------------------------------------------  626
 Eco16 --------------------------------------------------------------------------------  711
 Eco25 -------------------------------------------------------------------------------- 2660
 Eco15 -------------------------------------------------------------------------------- 1700
  Eco6 -------------------------------------------------------------------------------- 2836
  Efe2 -------------------------------------------------------------------------------- 1089
  Efe3 -------------------------------------------------------------------------------- 2104
  Yfr1 --------------------------------------------------------------------------------  770
  Yfr5 --------------------------------------------------------------------------------  780
  Yps4 LLTHTQSGVSNVVATIDTVNANIDTAFVAGAVATITLTTPVNGAVADGANSNSVQAVVTDSGGNPVNGAAVVFSSANATA 3408
  Yps7 -------------------------------------------------------------------------------- 1486
  Yfr2 --------------------------------------------------------------------------------  906
  Yfr3 --------------------------------------------------------------------------------  851
  Yps2 --------------------------------------------------------------------------------  985
  Ype5 -------------------------------------------------------------------------------- 1050
  Pru1 -------------------------------------------------------------------------------- 2373
  Pal3 --------------------------------------------------------------------------------  542
  Eta1 -------------------------------------------------------------------------------- 1400
  Sgl1 --------------------------------------------------------------------------------  934
 Eco26 -------------------------------------------------------------------------------- 1653
  Pmi1 -------------------------------------------------------------------------------- 2358
  Eco1 --------------------------------------------------------------------------------  937
  Ymo1 -------------------------------------------------------------------------------- 1424
  Yen2 -------------------------------------------------------------------------------- 2484
  Ybe1 --------------------------------------------------------------------------------  945
  Esa2 -------------------------------------------------------------------------------- 1027
  Sen3 -------------------------------------------------------------------------------- 1812
 Eco14 -------------------------------------------------------------------------------- 1084
  Bpe1 -------------------------------------------------------------------------------- 1308
  Bav2 -------------------------------------------------------------------------------- 1654
  Bbr1 --------------------------------------------------------------------------------  969
  Bpa2 -------------------------------------------------------------------------------- 1937
  Bav1 --------------------------------------------------------------------------------  747
  Pma1 --------------------------------------------------------------------------------  372
  Pma5 --------------------------------------------------------------------------------  373
  Pma3 --------------------------------------------------------------------------------  379
  Pma4 --------------------------------------------------------------------------------  410
  Ssp1 --------------------------------------------------------------------------------  428
  Ssp2 --------------------------------------------------------------------------------  436
 Eco20 -------------------------------------------------------------------------------- 1418
  Efe5 -------------------------------------------------------------------------------- 1185
  Plu1 -------------------------------------------------------------------------------- 1695
  Pal2 -------------------------------------------------------------------------------- 2521
  Ahy1 --------------------------------------------------------------------------------  916
  Csu1 -------------------------------------------------------------------------------- 1305
  Plu2 --------------------------------------------------------------------------------  302
  Rba1 --------------------------------------------------------------------------------  327
  Pas1 --------------------------------------------------------------------------------  924
  Cla1 -------------------------------------------------------------------------------- 1459
 ruler ......5850......5860......5870......5880......5890......5900......5910......5920
```

```
   Mba1 --------------------------------------------------------------------------------  331
   Psp2 --------------------------------------------------------------------------------  251
   Sen2 --------------------------------------------------------------------------------  660
   Efe4 --------------------------------------------------------------------------------  672
   Eco3 --------------------------------------------------------------------------------  417
   Cko1 --------------------------------------------------------------------------------  454
   Sen1 --------------------------------------------------------------------------------  460
   Eca1 --------------------------------------------------------------------------------  468
   Esp2 --------------------------------------------------------------------------------  468
   Esa3 --------------------------------------------------------------------------------  471
   Kpn1 --------------------------------------------------------------------------------  460
   Pan1 --------------------------------------------------------------------------------  490
   Eta2 --------------------------------------------------------------------------------  480
   Spr1 --------------------------------------------------------------------------------  497
   Yin1 --------------------------------------------------------------------------------  751
   Yin2 --------------------------------------------------------------------------------  753
   Yfr4 --------------------------------------------------------------------------------  749
   Ymo2 --------------------------------------------------------------------------------  686
  Eco10 --------------------------------------------------------------------------------  734
   Eal1 --------------------------------------------------------------------------------  725
   Sty4 --------------------------------------------------------------------------------  626
  Eco16 --------------------------------------------------------------------------------  711
  Eco25 -------------------------------------------------------------------------------- 2660
  Eco15 -------------------------------------------------------------------------------- 1700
   Eco6 -------------------------------------------------------------------------------- 2836
   Efe2 -------------------------------------------------------------------------------- 1089
   Efe3 -------------------------------------------------------------------------------- 2104
   Yfr1 --------------------------------------------------------------------------------  770
   Yfr5 --------------------------------------------------------------------------------  780
   Yps4 QITTVIGTTGADGIATATLTNTVAGTSNVAATIDTVNANIDTTFVAGAVATITLTTPVNGAVADGANSNSVQAVVSDSEG 3488
   Yps7 -------------------------------------------------------------------------------- 1486
   Yfr2 --------------------------------------------------------------------------------  906
   Yfr3 --------------------------------------------------------------------------------  851
   Yps2 --------------------------------------------------------------------------------  985
   Ype5 -------------------------------------------------------------------------------- 1050
   Pru1 -------------------------------------------------------------------------------- 2373
   Pal3 --------------------------------------------------------------------------------  542
   Eta1 -------------------------------------------------------------------------------- 1400
   Sgl1 --------------------------------------------------------------------------------  934
  Eco26 -------------------------------------------------------------------------------- 1653
   Pmi1 -------------------------------------------------------------------------------- 2358
   Eco1 --------------------------------------------------------------------------------  937
   Ymo1 -------------------------------------------------------------------------------- 1424
   Yen2 -------------------------------------------------------------------------------- 2484
   Ybe1 --------------------------------------------------------------------------------  945
   Esa2 -------------------------------------------------------------------------------- 1027
   Sen3 -------------------------------------------------------------------------------- 1812
  Eco14 -------------------------------------------------------------------------------- 1084
   Bpe1 -------------------------------------------------------------------------------- 1308
   Bav2 -------------------------------------------------------------------------------- 1654
   Bbr1 --------------------------------------------------------------------------------  969
   Bpa2 -------------------------------------------------------------------------------- 1937
   Bav1 --------------------------------------------------------------------------------  747
   Pma1 --------------------------------------------------------------------------------  372
   Pma5 --------------------------------------------------------------------------------  373
   Pma3 --------------------------------------------------------------------------------  379
   Pma4 --------------------------------------------------------------------------------  410
   Ssp1 --------------------------------------------------------------------------------  428
   Ssp2 --------------------------------------------------------------------------------  436
  Eco20 -------------------------------------------------------------------------------- 1418
   Efe5 -------------------------------------------------------------------------------- 1185
   Plu1 -------------------------------------------------------------------------------- 1695
   Pal2 -------------------------------------------------------------------------------- 2521
   Ahy1 --------------------------------------------------------------------------------  916
   Csu1 -------------------------------------------------------------------------------- 1305
   Plu2 --------------------------------------------------------------------------------  302
   Rba1 --------------------------------------------------------------------------------  327
   Pas1 --------------------------------------------------------------------------------  924
   Cla1 -------------------------------------------------------------------------------- 1459
  ruler ......5930......5940......5950......5960......5970......5980......5990......6000
```

```
  Mba1 --------------------------------------------------------------------------------  331
  Psp2 --------------------------------------------------------------------------------  251
  Sen2 --------------------------------------------------------------------------------  660
  Efe4 --------------------------------------------------------------------------------  672
  Eco3 --------------------------------------------------------------------------------  417
  Cko1 --------------------------------------------------------------------------------  454
  Sen1 --------------------------------------------------------------------------------  460
  Eca1 --------------------------------------------------------------------------------  468
  Esp2 --------------------------------------------------------------------------------  468
  Esa3 --------------------------------------------------------------------------------  471
  Kpn1 --------------------------------------------------------------------------------  460
  Pan1 --------------------------------------------------------------------------------  490
  Eta2 --------------------------------------------------------------------------------  480
  Spr1 --------------------------------------------------------------------------------  497
  Yin1 --------------------------------------------------------------------------------  751
  Yin2 --------------------------------------------------------------------------------  753
  Yfr4 --------------------------------------------------------------------------------  749
  Ymo2 --------------------------------------------------------------------------------  686
 Eco10 --------------------------------------------------------------------------------  734
  Eal1 --------------------------------------------------------------------------------  725
  Sty4 --------------------------------------------------------------------------------  626
 Eco16 --------------------------------------------------------------------------------  711
 Eco25 -------------------------------------------------------------------------------- 2660
 Eco15 -------------------------------------------------------------------------------- 1700
  Eco6 -------------------------------------------------------------------------------- 2836
  Efe2 -------------------------------------------------------------------------------- 1089
  Efe3 -------------------------------------------------------------------------------- 2104
  Yfr1 --------------------------------------------------------------------------------  770
  Yfr5 --------------------------------------------------------------------------------  780
  Yps4 NPVNGATVVFSSINATAQITTVIGTTGVDGIATATLTNTVAGTSNVVATIDTVNANIDTTFVAGAVATITLTTLVNGAVA 3568
  Yps7 -------------------------------------------------------------------------------- 1486
  Yfr2 --------------------------------------------------------------------------------  906
  Yfr3 --------------------------------------------------------------------------------  851
  Yps2 --------------------------------------------------------------------------------  985
  Ype5 -------------------------------------------------------------------------------- 1050
  Pru1 -------------------------------------------------------------------------------- 2373
  Pal3 --------------------------------------------------------------------------------  542
  Eta1 -------------------------------------------------------------------------------- 1400
  Sgl1 --------------------------------------------------------------------------------  934
 Eco26 -------------------------------------------------------------------------------- 1653
  Pmi1 -------------------------------------------------------------------------------- 2358
  Eco1 --------------------------------------------------------------------------------  937
  Ymo1 -------------------------------------------------------------------------------- 1424
  Yen2 -------------------------------------------------------------------------------- 2484
  Ybe1 --------------------------------------------------------------------------------  945
  Esa2 -------------------------------------------------------------------------------- 1027
  Sen3 -------------------------------------------------------------------------------- 1812
 Eco14 -------------------------------------------------------------------------------- 1084
  Bpe1 -------------------------------------------------------------------------------- 1308
  Bav2 -------------------------------------------------------------------------------- 1654
  Bbr1 --------------------------------------------------------------------------------  969
  Bpa2 -------------------------------------------------------------------------------- 1937
  Bav1 --------------------------------------------------------------------------------  747
  Pma1 --------------------------------------------------------------------------------  372
  Pma5 --------------------------------------------------------------------------------  373
  Pma3 --------------------------------------------------------------------------------  379
  Pma4 --------------------------------------------------------------------------------  410
  Ssp1 --------------------------------------------------------------------------------  428
  Ssp2 --------------------------------------------------------------------------------  436
 Eco20 -------------------------------------------------------------------------------- 1418
  Efe5 -------------------------------------------------------------------------------- 1185
  Plu1 -------------------------------------------------------------------------------- 1695
  Pal2 -------------------------------------------------------------------------------- 2521
  Ahy1 --------------------------------------------------------------------------------  916
  Csu1 -------------------------------------------------------------------------------- 1305
  Plu2 --------------------------------------------------------------------------------  302
  Rba1 --------------------------------------------------------------------------------  327
  Pas1 --------------------------------------------------------------------------------  924
  Cla1 -------------------------------------------------------------------------------- 1459
 ruler ......6010......6020......6030......6040......6050......6060......6070......6080
```

```
Mba1   --------------------------------------------------------------------------------   331
Psp2   --------------------------------------------------------------------------------   251
Sen2   --------------------------------------------------------------------------------   660
Efe4   --------------------------------------------------------------------------------   672
Eco3   --------------------------------------------------------------------------------   417
Cko1   --------------------------------------------------------------------------------   454
Sen1   --------------------------------------------------------------------------------   460
Eca1   --------------------------------------------------------------------------------   468
Esp2   --------------------------------------------------------------------------------   468
Esa3   --------------------------------------------------------------------------------   471
Kpn1   --------------------------------------------------------------------------------   460
Pan1   --------------------------------------------------------------------------------   490
Eta2   --------------------------------------------------------------------------------   480
Spr1   --------------------------------------------------------------------------------   497
Yin1   --------------------------------------------------------------------------------   751
Yin2   --------------------------------------------------------------------------------   753
Yfr4   --------------------------------------------------------------------------------   749
Ymo2   --------------------------------------------------------------------------------   686
Eco10  --------------------------------------------------------------------------------   734
Eal1   --------------------------------------------------------------------------------   725
Sty4   --------------------------------------------------------------------------------   626
Eco16  --------------------------------------------------------------------------------   711
Eco25  --------------------------------------------------------------------------------  2660
Eco15  --------------------------------------------------------------------------------  1700
Eco6   --------------------------------------------------------------------------------  2836
Efe2   --------------------------------------------------------------------------------  1089
Efe3   --------------------------------------------------------------------------------  2104
Yfr1   --------------------------------------------------------------------------------   770
Yfr5   --------------------------------------------------------------------------------   780
Yps4   DGANSNSVQAVVSDSGGNPVTGAAVVFSSANATAQITTVIGTTGVDGIATATLTNTVAGTSNVVATIGSITNNIDTAFVA  3648
Yps7   --------------------------------------------------------------------------------  1486
Yfr2   --------------------------------------------------------------------------------   906
Yfr3   --------------------------------------------------------------------------------   851
Yps2   --------------------------------------------------------------------------------   985
Ype5   --------------------------------------------------------------------------------  1050
Pru1   --------------------------------------------------------------------------------  2373
Pal3   --------------------------------------------------------------------------------   542
Eta1   --------------------------------------------------------------------------------  1400
Sgl1   --------------------------------------------------------------------------------   934
Eco26  --------------------------------------------------------------------------------  1653
Pmi1   --------------------------------------------------------------------------------  2358
Eco1   --------------------------------------------------------------------------------   937
Ymo1   --------------------------------------------------------------------------------  1424
Yen2   --------------------------------------------------------------------------------  2484
Ybe1   --------------------------------------------------------------------------------   945
Esa2   --------------------------------------------------------------------------------  1027
Sen3   --------------------------------------------------------------------------------  1812
Eco14  --------------------------------------------------------------------------------  1084
Bpe1   --------------------------------------------------------------------------------  1308
Bav2   --------------------------------------------------------------------------------  1654
Bbr1   --------------------------------------------------------------------------------   969
Bpa2   --------------------------------------------------------------------------------  1937
Bav1   --------------------------------------------------------------------------------   747
Pma1   --------------------------------------------------------------------------------   372
Pma5   --------------------------------------------------------------------------------   373
Pma3   --------------------------------------------------------------------------------   379
Pma4   --------------------------------------------------------------------------------   410
Ssp1   --------------------------------------------------------------------------------   428
Ssp2   --------------------------------------------------------------------------------   436
Eco20  --------------------------------------------------------------------------------  1418
Efe5   --------------------------------------------------------------------------------  1185
Plu1   --------------------------------------------------------------------------------  1695
Pal2   --------------------------------------------------------------------------------  2521
Ahy1   --------------------------------------------------------------------------------   916
Csu1   --------------------------------------------------------------------------------  1305
Plu2   --------------------------------------------------------------------------------   302
Rba1   --------------------------------------------------------------------------------   327
Pas1   --------------------------------------------------------------------------------   924
Cla1   --------------------------------------------------------------------------------  1459
ruler  ......6090......6100......6110......6120......6130......6140......6150......6160
```

```
Mba1   --------------------------------------------------------------------------------   331
Psp2   --------------------------------------------------------------------------------   251
Sen2   --------------------------------------------------------------------------------   660
Efe4   --------------------------------------------------------------------------------   672
Eco3   --------------------------------------------------------------------------------   417
Cko1   --------------------------------------------------------------------------------   454
Sen1   --------------------------------------------------------------------------------   460
Eca1   --------------------------------------------------------------------------------   468
Esp2   --------------------------------------------------------------------------------   468
Esa3   --------------------------------------------------------------------------------   471
Kpn1   --------------------------------------------------------------------------------   460
Pan1   --------------------------------------------------------------------------------   490
Eta2   --------------------------------------------------------------------------------   480
Spr1   --------------------------------------------------------------------------------   497
Yin1   --------------------------------------------------------------------------------   751
Yin2   --------------------------------------------------------------------------------   753
Yfr4   --------------------------------------------------------------------------------   749
Ymo2   --------------------------------------------------------------------------------   686
Eco10  --------------------------------------------------------------------------------   734
Eal1   --------------------------------------------------------------------------------   725
Sty4   --------------------------------------------------------------------------------   626
Eco16  --------------------------------------------------------------------------------   711
Eco25  --------------------------------------------------------------------------------  2660
Eco15  --------------------------------------------------------------------------------  1700
Eco6   --------------------------------------------------------------------------------  2836
Efe2   --------------------------------------------------------------------------------  1089
Efe3   --------------------------------------------------------------------------------  2104
Yfr1   --------------------------------------------------------------------------------   770
Yfr5   --------------------------------------------------------------------------------   780
Yps4   GAVATITLTTPVNGAVADGANSNSVQAVVTDSGGNPVNGAAVVFSSANATAQITTVIGTTGADGIATATLTNTVAGTSNV  3728
Yps7   --------------------------------------------------------------------------------  1486
Yfr2   --------------------------------------------------------------------------------   906
Yfr3   --------------------------------------------------------------------------------   851
Yps2   --------------------------------------------------------------------------------   985
Ype5   --------------------------------------------------------------------------------  1050
Pru1   --------------------------------------------------------------------------------  2373
Pal3   --------------------------------------------------------------------------------   542
Eta1   --------------------------------------------------------------------------------  1400
Sgl1   --------------------------------------------------------------------------------   934
Eco26  --------------------------------------------------------------------------------  1653
Pmi1   --------------------------------------------------------------------------------  2358
Eco1   --------------------------------------------------------------------------------   937
Ymo1   --------------------------------------------------------------------------------  1424
Yen2   --------------------------------------------------------------------------------  2484
Ybe1   --------------------------------------------------------------------------------   945
Esa2   --------------------------------------------------------------------------------  1027
Sen3   --------------------------------------------------------------------------------  1812
Eco14  --------------------------------------------------------------------------------  1084
Bpe1   --------------------------------------------------------------------------------  1308
Bav2   --------------------------------------------------------------------------------  1654
Bbr1   --------------------------------------------------------------------------------   969
Bpa2   --------------------------------------------------------------------------------  1937
Bav1   --------------------------------------------------------------------------------   747
Pma1   --------------------------------------------------------------------------------   372
Pma5   --------------------------------------------------------------------------------   373
Pma3   --------------------------------------------------------------------------------   379
Pma4   --------------------------------------------------------------------------------   410
Ssp1   --------------------------------------------------------------------------------   428
Ssp2   --------------------------------------------------------------------------------   436
Eco20  --------------------------------------------------------------------------------  1418
Efe5   --------------------------------------------------------------------------------  1185
Plu1   --------------------------------------------------------------------------------  1695
Pal2   --------------------------------------------------------------------------------  2521
Ahy1   --------------------------------------------------------------------------------   916
Csu1   --------------------------------------------------------------------------------  1305
Plu2   --------------------------------------------------------------------------------   302
Rba1   --------------------------------------------------------------------------------   327
Pas1   --------------------------------------------------------------------------------   924
Cla1   --------------------------------------------------------------------------------  1459
ruler  ......6170......6180......6190......6200......6210......6220......6230......6240
```

```
Mba1   --------------------------------------------------------------------------------   331
Psp2   --------------------------------------------------------------------------------   251
Sen2   --------------------------------------------------------------------------------   660
Efe4   --------------------------------------------------------------------------------   672
Eco3   --------------------------------------------------------------------------------   417
Cko1   --------------------------------------------------------------------------------   454
Sen1   --------------------------------------------------------------------------------   460
Eca1   --------------------------------------------------------------------------------   468
Esp2   --------------------------------------------------------------------------------   468
Esa3   --------------------------------------------------------------------------------   471
Kpn1   --------------------------------------------------------------------------------   460
Pan1   --------------------------------------------------------------------------------   490
Eta2   --------------------------------------------------------------------------------   480
Spr1   --------------------------------------------------------------------------------   497
Yin1   --------------------------------------------------------------------------------   751
Yin2   --------------------------------------------------------------------------------   753
Yfr4   --------------------------------------------------------------------------------   749
Ymo2   --------------------------------------------------------------------------------   686
Eco10  --------------------------------------------------------------------------------   734
Eal1   --------------------------------------------------------------------------------   725
Sty4   --------------------------------------------------------------------------------   626
Eco16  --------------------------------------------------------------------------------   711
Eco25  --------------------------------------------------------------------------------  2660
Eco15  --------------------------------------------------------------------------------  1700
Eco6   --------------------------------------------------------------------------------  2836
Efe2   --------------------------------------------------------------------------------  1089
Efe3   --------------------------------------------------------------------------------  2104
Yfr1   --------------------------------------------------------------------------------   770
Yfr5   --------------------------------------------------------------------------------   780
Yps4   IATIDTVNANIDTTFVAGAVATITLTTPVNGAVADGADSNSVQAVVSDSEGNAVTGAAVVFSSANATAQITTVIGTTGAD  3808
Yps7   --------------------------------------------------------------------------------  1486
Yfr2   --------------------------------------------------------------------------------   906
Yfr3   --------------------------------------------------------------------------------   851
Yps2   --------------------------------------------------------------------------------   985
Ype5   --------------------------------------------------------------------------------  1050
Pru1   --------------------------------------------------------------------------------  2373
Pal3   --------------------------------------------------------------------------------   542
Eta1   --------------------------------------------------------------------------------  1400
Sgl1   --------------------------------------------------------------------------------   934
Eco26  --------------------------------------------------------------------------------  1653
Pmi1   --------------------------------------------------------------------------------  2358
Eco1   --------------------------------------------------------------------------------   937
Ymo1   --------------------------------------------------------------------------------  1424
Yen2   --------------------------------------------------------------------------------  2484
Ybe1   --------------------------------------------------------------------------------   945
Esa2   --------------------------------------------------------------------------------  1027
Sen3   --------------------------------------------------------------------------------  1812
Eco14  --------------------------------------------------------------------------------  1084
Bpe1   --------------------------------------------------------------------------------  1308
Bav2   --------------------------------------------------------------------------------  1654
Bbr1   --------------------------------------------------------------------------------   969
Bpa2   --------------------------------------------------------------------------------  1937
Bav1   --------------------------------------------------------------------------------   747
Pma1   --------------------------------------------------------------------------------   372
Pma5   --------------------------------------------------------------------------------   373
Pma3   --------------------------------------------------------------------------------   379
Pma4   --------------------------------------------------------------------------------   410
Ssp1   --------------------------------------------------------------------------------   428
Ssp2   --------------------------------------------------------------------------------   436
Eco20  --------------------------------------------------------------------------------  1418
Efe5   --------------------------------------------------------------------------------  1185
Plu1   --------------------------------------------------------------------------------  1695
Pal2   --------------------------------------------------------------------------------  2521
Ahy1   --------------------------------------------------------------------------------   916
Csu1   --------------------------------------------------------------------------------  1305
Plu2   --------------------------------------------------------------------------------   302
Rba1   --------------------------------------------------------------------------------   327
Pas1   --------------------------------------------------------------------------------   924
Cla1   --------------------------------------------------------------------------------  1459
ruler  ......6250......6260......6270......6280......6290......6300......6310......6320
```

```
  Mba1 --------------------------------------------------------------------------------  331
  Psp2 --------------------------------------------------------------------------------  251
  Sen2 --------------------------------------------------------------------------------  660
  Efe4 --------------------------------------------------------------------------------  672
  Eco3 --------------------------------------------------------------------------------  417
  Cko1 --------------------------------------------------------------------------------  454
  Sen1 --------------------------------------------------------------------------------  460
  Eca1 --------------------------------------------------------------------------------  468
  Esp2 --------------------------------------------------------------------------------  468
  Esa3 --------------------------------------------------------------------------------  471
  Kpn1 --------------------------------------------------------------------------------  460
  Pan1 --------------------------------------------------------------------------------  490
  Eta2 --------------------------------------------------------------------------------  480
  Spr1 --------------------------------------------------------------------------------  497
  Yin1 --------------------------------------------------------------------------------  751
  Yin2 --------------------------------------------------------------------------------  753
  Yfr4 --------------------------------------------------------------------------------  749
  Ymo2 --------------------------------------------------------------------------------  686
 Eco10 --------------------------------------------------------------------------------  734
  Eal1 --------------------------------------------------------------------------------  725
  Sty4 --------------------------------------------------------------------------------  626
 Eco16 --------------------------------------------------------------------------------  711
 Eco25 -------------------------------------------------------------------------------- 2660
 Eco15 -------------------------------------------------------------------------------- 1700
  Eco6 -------------------------------------------------------------------------------- 2836
  Efe2 -------------------------------------------------------------------------------- 1089
  Efe3 -------------------------------------------------------------------------------- 2104
  Yfr1 --------------------------------------------------------------------------------  770
  Yfr5 --------------------------------------------------------------------------------  780
  Yps4 GIATATLTNTVAGTSNVVATIDTVNANIDTAFVAGELENIVVSIINNNALANGADTNIVEAFVTDRFGNGVANQSLMFGT 3888
  Yps7 -------------------------------------------------------------------------------- 1486
  Yfr2 --------------------------------------------------------------------------------  906
  Yfr3 --------------------------------------------------------------------------------  851
  Yps2 --------------------------------------------------------------------------------  985
  Ype5 -------------------------------------------------------------------------------- 1050
  Pru1 -------------------------------------------------------------------------------- 2373
  Pal3 --------------------------------------------------------------------------------  542
  Eta1 -------------------------------------------------------------------------------- 1400
  Sgl1 --------------------------------------------------------------------------------  934
 Eco26 -------------------------------------------------------------------------------- 1653
  Pmi1 -------------------------------------------------------------------------------- 2358
  Eco1 --------------------------------------------------------------------------------  937
  Ymo1 -------------------------------------------------------------------------------- 1424
  Yen2 -------------------------------------------------------------------------------- 2484
  Ybe1 --------------------------------------------------------------------------------  945
  Esa2 -------------------------------------------------------------------------------- 1027
  Sen3 -------------------------------------------------------------------------------- 1812
 Eco14 -------------------------------------------------------------------------------- 1084
  Bpe1 -------------------------------------------------------------------------------- 1308
  Bav2 -------------------------------------------------------------------------------- 1654
  Bbr1 --------------------------------------------------------------------------------  969
  Bpa2 -------------------------------------------------------------------------------- 1937
  Bav1 --------------------------------------------------------------------------------  747
  Pma1 --------------------------------------------------------------------------------  372
  Pma5 --------------------------------------------------------------------------------  373
  Pma3 --------------------------------------------------------------------------------  379
  Pma4 --------------------------------------------------------------------------------  410
  Ssp1 --------------------------------------------------------------------------------  428
  Ssp2 --------------------------------------------------------------------------------  436
 Eco20 -------------------------------------------------------------------------------- 1418
  Efe5 -------------------------------------------------------------------------------- 1185
  Plu1 -------------------------------------------------------------------------------- 1695
  Pal2 -------------------------------------------------------------------------------- 2521
  Ahy1 --------------------------------------------------------------------------------  916
  Csu1 -------------------------------------------------------------------------------- 1305
  Plu2 --------------------------------------------------------------------------------  302
  Rba1 --------------------------------------------------------------------------------  327
  Pas1 --------------------------------------------------------------------------------  924
  Cla1 -------------------------------------------------------------------------------- 1459
 ruler ......6330......6340......6350......6360......6370......6380......6390......6400
```

```
   Mba1 --------------------------------------------------------------------------------  331
   Psp2 --------------------------------------------------------------------------------  251
   Sen2 --------------------------------------------------------------------------------  660
   Efe4 --------------------------------------------------------------------------------  672
   Eco3 --------------------------------------------------------------------------------  417
   Cko1 --------------------------------------------------------------------------------  454
   Sen1 --------------------------------------------------------------------------------  460
   Eca1 --------------------------------------------------------------------------------  468
   Esp2 --------------------------------------------------------------------------------  468
   Esa3 --------------------------------------------------------------------------------  471
   Kpn1 --------------------------------------------------------------------------------  460
   Pan1 --------------------------------------------------------------------------------  490
   Eta2 --------------------------------------------------------------------------------  480
   Spr1 --------------------------------------------------------------------------------  497
   Yin1 --------------------------------------------------------------------------------  751
   Yin2 --------------------------------------------------------------------------------  753
   Yfr4 --------------------------------------------------------------------------------  749
   Ymo2 --------------------------------------------------------------------------------  686
  Eco10 --------------------------------------------------------------------------------  734
   Eal1 --------------------------------------------------------------------------------  725
   Sty4 --------------------------------------------------------------------------------  626
  Eco16 --------------------------------------------------------------------------------  711
  Eco25 --------------------------------------------------------------------------------  2660
  Eco15 --------------------------------------------------------------------------------  1700
   Eco6 --------------------------------------------------------------------------------  2836
   Efe2 --------------------------------------------------------------------------------  1089
   Efe3 --------------------------------------------------------------------------------  2104
   Yfr1 --------------------------------------------------------------------------------  770
   Yfr5 --------------------------------------------------------------------------------  780
   Yps4 NGASIVGSSTVTTNIDGRVRVSATHTVAGSSNTVFAISGAHQGYTRVTFVADASTAQLKLTSFLDNQLANGKAGNIAQAL  3968
   Yps7 --------------------------------------------------------------------------------  1486
   Yfr2 --------------------------------------------------------------------------------  906
   Yfr3 --------------------------------------------------------------------------------  851
   Yps2 --------------------------------------------------------------------------------  985
   Ype5 --------------------------------------------------------------------------------  1050
   Pru1 --------------------------------------------------------------------------------  2373
   Pal3 --------------------------------------------------------------------------------  542
   Eta1 --------------------------------------------------------------------------------  1400
   Sgl1 --------------------------------------------------------------------------------  934
  Eco26 --------------------------------------------------------------------------------  1653
   Pmi1 --------------------------------------------------------------------------------  2358
   Eco1 --------------------------------------------------------------------------------  937
   Ymo1 --------------------------------------------------------------------------------  1424
   Yen2 --------------------------------------------------------------------------------  2484
   Ybe1 --------------------------------------------------------------------------------  945
   Esa2 --------------------------------------------------------------------------------  1027
   Sen3 --------------------------------------------------------------------------------  1812
  Eco14 --------------------------------------------------------------------------------  1084
   Bpe1 --------------------------------------------------------------------------------  1308
   Bav2 --------------------------------------------------------------------------------  1654
   Bbr1 --------------------------------------------------------------------------------  969
   Bpa2 --------------------------------------------------------------------------------  1937
   Bav1 --------------------------------------------------------------------------------  747
   Pma1 --------------------------------------------------------------------------------  372
   Pma5 --------------------------------------------------------------------------------  373
   Pma3 --------------------------------------------------------------------------------  379
   Pma4 --------------------------------------------------------------------------------  410
   Ssp1 --------------------------------------------------------------------------------  428
   Ssp2 --------------------------------------------------------------------------------  436
  Eco20 --------------------------------------------------------------------------------  1418
   Efe5 --------------------------------------------------------------------------------  1185
   Plu1 --------------------------------------------------------------------------------  1695
   Pal2 --------------------------------------------------------------------------------  2521
   Ahy1 --------------------------------------------------------------------------------  916
   Csu1 --------------------------------------------------------------------------------  1305
   Plu2 --------------------------------------------------------------------------------  302
   Rba1 --------------------------------------------------------------------------------  327
   Pas1 --------------------------------------------------------------------------------  924
   Cla1 --------------------------------------------------------------------------------  1459
  ruler ......6410......6420......6430......6440......6450......6460......6470......6480
```

```
Mba1   --------------------------------------------------------------------------------  331
Psp2   --------------------------------------------------------------------------------  251
Sen2   --------------------------------------------------------------------------------  660
Efe4   --------------------------------------------------------------------------------  672
Eco3   --------------------------------------------------------------------------------  417
Cko1   --------------------------------------------------------------------------------  454
Sen1   --------------------------------------------------------------------------------  460
Eca1   --------------------------------------------------------------------------------  468
Esp2   --------------------------------------------------------------------------------  468
Esa3   --------------------------------------------------------------------------------  471
Kpn1   --------------------------------------------------------------------------------  460
Pan1   --------------------------------------------------------------------------------  490
Eta2   --------------------------------------------------------------------------------  480
Spr1   --------------------------------------------------------------------------------  497
Yin1   --------------------------------------------------------------------------------  751
Yin2   --------------------------------------------------------------------------------  753
Yfr4   --------------------------------------------------------------------------------  749
Ymo2   --------------------------------------------------------------------------------  686
Eco10  --------------------------------------------------------------------------------  734
Eal1   --------------------------------------------------------------------------------  725
Sty4   --------------------------------------------------------------------------------  626
Eco16  --------------------------------------------------------------------------------  711
Eco25  --------------------------------------------------------------------------------  2660
Eco15  --------------------------------------------------------------------------------  1700
Eco6   --------------------------------------------------------------------------------  2836
Efe2   --------------------------------------------------------------------------------  1089
Efe3   --------------------------------------------------------------------------------  2104
Yfr1   --------------------------------------------------------------------------------  770
Yfr5   --------------------------------------------------------------------------------  780
Yps4   VTDAYDNPLANQSVSFALDNGAVIESRGDASSASGIVLMRFNNTLAGMTTVTATLDSTGQTETLEMHFVAGKAASIELTM  4048
Yps7   --------------------------------------------------------------------------------  1486
Yfr2   --------------------------------------------------------------------------------  906
Yfr3   --------------------------------------------------------------------------------  851
Yps2   --------------------------------------------------------------------------------  985
Ype5   --------------------------------------------------------------------------------  1050
Pru1   --------------------------------------------------------------------------------  2373
Pal3   --------------------------------------------------------------------------------  542
Eta1   --------------------------------------------------------------------------------  1400
Sgl1   --------------------------------------------------------------------------------  934
Eco26  --------------------------------------------------------------------------------  1653
Pmi1   --------------------------------------------------------------------------------  2358
Eco1   --------------------------------------------------------------------------------  937
Ymo1   --------------------------------------------------------------------------------  1424
Yen2   --------------------------------------------------------------------------------  2484
Ybe1   --------------------------------------------------------------------------------  945
Esa2   --------------------------------------------------------------------------------  1027
Sen3   --------------------------------------------------------------------------------  1812
Eco14  --------------------------------------------------------------------------------  1084
Bpe1   --------------------------------------------------------------------------------  1308
Bav2   --------------------------------------------------------------------------------  1654
Bbr1   --------------------------------------------------------------------------------  969
Bpa2   --------------------------------------------------------------------------------  1937
Bav1   --------------------------------------------------------------------------------  747
Pma1   --------------------------------------------------------------------------------  372
Pma5   --------------------------------------------------------------------------------  373
Pma3   --------------------------------------------------------------------------------  379
Pma4   --------------------------------------------------------------------------------  410
Ssp1   --------------------------------------------------------------------------------  428
Ssp2   --------------------------------------------------------------------------------  436
Eco20  --------------------------------------------------------------------------------  1418
Efe5   --------------------------------------------------------------------------------  1185
Plu1   --------------------------------------------------------------------------------  1695
Pal2   --------------------------------------------------------------------------------  2521
Ahy1   --------------------------------------------------------------------------------  916
Csu1   --------------------------------------------------------------------------------  1305
Plu2   --------------------------------------------------------------------------------  302
Rba1   --------------------------------------------------------------------------------  327
Pas1   --------------------------------------------------------------------------------  924
Cla1   --------------------------------------------------------------------------------  1459
ruler  ......6490......6500......6510......6520......6530......6540......6550......6560
```

```
  Mba1 --------------------------------------------------------------------------------   331
  Psp2 --------------------------------------------------------------------------------   251
  Sen2 --------------------------------------------------------------------------------   660
  Efe4 --------------------------------------------------------------------------------   672
  Eco3 --------------------------------------------------------------------------------   417
  Cko1 --------------------------------------------------------------------------------   454
  Sen1 --------------------------------------------------------------------------------   460
  Eca1 --------------------------------------------------------------------------------   468
  Esp2 --------------------------------------------------------------------------------   468
  Esa3 --------------------------------------------------------------------------------   471
  Kpn1 --------------------------------------------------------------------------------   460
  Pan1 --------------------------------------------------------------------------------   490
  Eta2 --------------------------------------------------------------------------------   480
  Spr1 --------------------------------------------------------------------------------   497
  Yin1 --------------------------------------------------------------------------------   751
  Yin2 --------------------------------------------------------------------------------   753
  Yfr4 --------------------------------------------------------------------------------   749
  Ymo2 --------------------------------------------------------------------------------   686
 Eco10 --------------------------------------------------------------------------------   734
  Eal1 --------------------------------------------------------------------------------   725
  Sty4 --------------------------------------------------------------------------------   626
 Eco16 --------------------------------------------------------------------------------   711
 Eco25 --------------------------------------------------------------------------------  2660
 Eco15 --------------------------------------------------------------------------------  1700
  Eco6 --------------------------------------------------------------------------------  2836
  Efe2 --------------------------------------------------------------------------------  1089
  Efe3 --------------------------------------------------------------------------------  2104
  Yfr1 --------------------------------------------------------------------------------   770
  Yfr5 --------------------------------------------------------------------------------   780
  Yps4 TKDNAVANNIDTNEVQVLVTDADGNAINGAVVNLTSNSGMNITPNSVTTGSDGTATATLTHTLAGSLPINARIDQVSKTI  4128
  Yps7 --------------------------------------------------------------------------------  1486
  Yfr2 --------------------------------------------------------------------------------   906
  Yfr3 --------------------------------------------------------------------------------   851
  Yps2 --------------------------------------------------------------------------------   985
  Ype5 --------------------------------------------------------------------------------  1050
  Pru1 --------------------------------------------------------------------------------  2373
  Pal3 --------------------------------------------------------------------------------   542
  Eta1 --------------------------------------------------------------------------------  1400
  Sgl1 --------------------------------------------------------------------------------   934
 Eco26 --------------------------------------------------------------------------------  1653
  Pmi1 --------------------------------------------------------------------------------  2358
  Eco1 --------------------------------------------------------------------------------   937
  Ymo1 --------------------------------------------------------------------------------  1424
  Yen2 --------------------------------------------------------------------------------  2484
  Ybe1 --------------------------------------------------------------------------------   945
  Esa2 --------------------------------------------------------------------------------  1027
  Sen3 --------------------------------------------------------------------------------  1812
 Eco14 --------------------------------------------------------------------------------  1084
  Bpe1 --------------------------------------------------------------------------------  1308
  Bav2 --------------------------------------------------------------------------------  1654
  Bbr1 --------------------------------------------------------------------------------   969
  Bpa2 --------------------------------------------------------------------------------  1937
  Bav1 --------------------------------------------------------------------------------   747
  Pma1 --------------------------------------------------------------------------------   372
  Pma5 --------------------------------------------------------------------------------   373
  Pma3 --------------------------------------------------------------------------------   379
  Pma4 --------------------------------------------------------------------------------   410
  Ssp1 --------------------------------------------------------------------------------   428
  Ssp2 --------------------------------------------------------------------------------   436
 Eco20 --------------------------------------------------------------------------------  1418
  Efe5 --------------------------------------------------------------------------------  1185
  Plu1 --------------------------------------------------------------------------------  1695
  Pal2 --------------------------------------------------------------------------------  2521
  Ahy1 --------------------------------------------------------------------------------   916
  Csu1 --------------------------------------------------------------------------------  1305
  Plu2 --------------------------------------------------------------------------------   302
  Rba1 --------------------------------------------------------------------------------   327
  Pas1 --------------------------------------------------------------------------------   924
  Cla1 --------------------------------------------------------------------------------  1459
 ruler ......6570......6580......6590......6600......6610......6620......6630......6640
```

```
  Mba1 --------------------------------------------------------------------------------   331
  Psp2 --------------------------------------------------------------------------------   251
  Sen2 --------------------------------------------------------------------------------   660
  Efe4 --------------------------------------------------------------------------------   672
  Eco3 --------------------------------------------------------------------------------   417
  Cko1 --------------------------------------------------------------------------------   454
  Sen1 --------------------------------------------------------------------------------   460
  Eca1 --------------------------------------------------------------------------------   468
  Esp2 --------------------------------------------------------------------------------   468
  Esa3 --------------------------------------------------------------------------------   471
  Kpn1 --------------------------------------------------------------------------------   460
  Pan1 --------------------------------------------------------------------------------   490
  Eta2 --------------------------------------------------------------------------------   480
  Spr1 --------------------------------------------------------------------------------   497
  Yin1 --------------------------------------------------------------------------------   751
  Yin2 --------------------------------------------------------------------------------   753
  Yfr4 --------------------------------------------------------------------------------   749
  Ymo2 --------------------------------------------------------------------------------   686
 Eco10 --------------------------------------------------------------------------------   734
  Eal1 --------------------------------------------------------------------------------   725
  Sty4 --------------------------------------------------------------------------------   626
 Eco16 --------------------------------------------------------------------------------   711
 Eco25 --------------------------------------------------------------------------------  2660
 Eco15 --------------------------------------------------------------------------------  1700
  Eco6 --------------------------------------------------------------------------------  2836
  Efe2 --------------------------------------------------------------------------------  1089
  Efe3 --------------------------------------------------------------------------------  2104
  Yfr1 --------------------------------------------------------------------------------   770
  Yfr5 --------------------------------------------------------------------------------   780
  Yps4 NATFIADVSTAQIIASDMFIIVNDQVANGQAVNAVQARVTDSYGNPIQGQLVEFVLSNTGTIQYKLEETSVEGGVMVTFT  4208
  Yps7 --------------------------------------------------------------------------------  1486
  Yfr2 --------------------------------------------------------------------------------   906
  Yfr3 --------------------------------------------------------------------------------   851
  Yps2 --------------------------------------------------------------------------------   985
  Ype5 --------------------------------------------------------------------------------  1050
  Pru1 --------------------------------------------------------------------------------  2373
  Pal3 --------------------------------------------------------------------------------   542
  Eta1 --------------------------------------------------------------------------------  1400
  Sgl1 --------------------------------------------------------------------------------   934
 Eco26 --------------------------------------------------------------------------------  1653
  Pmi1 --------------------------------------------------------------------------------  2358
  Eco1 --------------------------------------------------------------------------------   937
  Ymo1 --------------------------------------------------------------------------------  1424
  Yen2 --------------------------------------------------------------------------------  2484
  Ybe1 --------------------------------------------------------------------------------   945
  Esa2 --------------------------------------------------------------------------------  1027
  Sen3 --------------------------------------------------------------------------------  1812
 Eco14 --------------------------------------------------------------------------------  1084
  Bpe1 --------------------------------------------------------------------------------  1308
  Bav2 --------------------------------------------------------------------------------  1654
  Bbr1 --------------------------------------------------------------------------------   969
  Bpa2 --------------------------------------------------------------------------------  1937
  Bav1 --------------------------------------------------------------------------------   747
  Pma1 --------------------------------------------------------------------------------   372
  Pma5 --------------------------------------------------------------------------------   373
  Pma3 --------------------------------------------------------------------------------   379
  Pma4 --------------------------------------------------------------------------------   410
  Ssp1 --------------------------------------------------------------------------------   428
  Ssp2 --------------------------------------------------------------------------------   436
 Eco20 --------------------------------------------------------------------------------  1418
  Efe5 --------------------------------------------------------------------------------  1185
  Plu1 --------------------------------------------------------------------------------  1695
  Pal2 --------------------------------------------------------------------------------  2521
  Ahy1 --------------------------------------------------------------------------------   916
  Csu1 --------------------------------------------------------------------------------  1305
  Plu2 --------------------------------------------------------------------------------   302
  Rba1 --------------------------------------------------------------------------------   327
  Pas1 --------------------------------------------------------------------------------   924
  Cla1 --------------------------------------------------------------------------------  1459
 ruler ......6650......6660......6670......6680......6690......6700......6710......6720
```

```
  Mba1 --------------------------------------------------------------------------------  331
  Psp2 --------------------------------------------------------------------------------  251
  Sen2 --------------------------------------------------------------------------------  660
  Efe4 --------------------------------------------------------------------------------  672
  Eco3 --------------------------------------------------------------------------------  417
  Cko1 --------------------------------------------------------------------------------  454
  Sen1 --------------------------------------------------------------------------------  460
  Eca1 --------------------------------------------------------------------------------  468
  Esp2 --------------------------------------------------------------------------------  468
  Esa3 --------------------------------------------------------------------------------  471
  Kpn1 --------------------------------------------------------------------------------  460
  Pan1 --------------------------------------------------------------------------------  490
  Eta2 --------------------------------------------------------------------------------  480
  Spr1 --------------------------------------------------------------------------------  497
  Yin1 --------------------------------------------------------------------------------  751
  Yin2 --------------------------------------------------------------------------------  753
  Yfr4 --------------------------------------------------------------------------------  749
  Ymo2 --------------------------------------------------------------------------------  686
 Eco10 --------------------------------------------------------------------------------  734
  Eal1 --------------------------------------------------------------------------------  725
  Sty4 --------------------------------------------------------------------------------  626
 Eco16 --------------------------------------------------------------------------------  711
 Eco25 -------------------------------------------------------------------------------- 2660
 Eco15 -------------------------------------------------------------------------------- 1700
  Eco6 -------------------------------------------------------------------------------- 2836
  Efe2 -------------------------------------------------------------------------------- 1089
  Efe3 -------------------------------------------------------------------------------- 2104
  Yfr1 --------------------------------------------------------------------------------  770
  Yfr5 --------------------------------------------------------------------------------  780
  Yps4 NTLAGITNVTATVVSSRSSQNVDTTFIADVTTAHIAESDLMVIVDNAVANNSEKNEVHARVTDAKGNVLSGQTVIFTSGN 4288
  Yps7 -------------------------------------------------------------------------------- 1486
  Yfr2 --------------------------------------------------------------------------------  906
  Yfr3 --------------------------------------------------------------------------------  851
  Yps2 --------------------------------------------------------------------------------  985
  Ype5 -------------------------------------------------------------------------------- 1050
  Pru1 -------------------------------------------------------------------------------- 2373
  Pal3 --------------------------------------------------------------------------------  542
  Eta1 -------------------------------------------------------------------------------- 1400
  Sgl1 --------------------------------------------------------------------------------  934
 Eco26 -------------------------------------------------------------------------------- 1653
  Pmi1 -------------------------------------------------------------------------------- 2358
  Eco1 --------------------------------------------------------------------------------  937
  Ymo1 -------------------------------------------------------------------------------- 1424
  Yen2 -------------------------------------------------------------------------------- 2484
  Ybe1 --------------------------------------------------------------------------------  945
  Esa2 -------------------------------------------------------------------------------- 1027
  Sen3 -------------------------------------------------------------------------------- 1812
 Eco14 -------------------------------------------------------------------------------- 1084
  Bpe1 -------------------------------------------------------------------------------- 1308
  Bav2 -------------------------------------------------------------------------------- 1654
  Bbr1 --------------------------------------------------------------------------------  969
  Bpa2 -------------------------------------------------------------------------------- 1937
  Bav1 --------------------------------------------------------------------------------  747
  Pma1 --------------------------------------------------------------------------------  372
  Pma5 --------------------------------------------------------------------------------  373
  Pma3 --------------------------------------------------------------------------------  379
  Pma4 --------------------------------------------------------------------------------  410
  Ssp1 --------------------------------------------------------------------------------  428
  Ssp2 --------------------------------------------------------------------------------  436
 Eco20 -------------------------------------------------------------------------------- 1418
  Efe5 -------------------------------------------------------------------------------- 1185
  Plu1 -------------------------------------------------------------------------------- 1695
  Pal2 -------------------------------------------------------------------------------- 2521
  Ahy1 --------------------------------------------------------------------------------  916
  Csu1 -------------------------------------------------------------------------------- 1305
  Plu2 --------------------------------------------------------------------------------  302
  Rba1 --------------------------------------------------------------------------------  327
  Pas1 --------------------------------------------------------------------------------  924
  Cla1 -------------------------------------------------------------------------------- 1459
 ruler ......6730......6740......6750......6760......6770......6780......6790......6800
```

```
Mba1    --------------------------------------------------------------------------------   331
Psp2    --------------------------------------------------------------------------------   251
Sen2    --------------------------------------------------------------------------------   660
Efe4    --------------------------------------------------------------------------------   672
Eco3    --------------------------------------------------------------------------------   417
Cko1    --------------------------------------------------------------------------------   454
Sen1    --------------------------------------------------------------------------------   460
Eca1    --------------------------------------------------------------------------------   468
Esp2    --------------------------------------------------------------------------------   468
Esa3    --------------------------------------------------------------------------------   471
Kpn1    --------------------------------------------------------------------------------   460
Pan1    --------------------------------------------------------------------------------   490
Eta2    --------------------------------------------------------------------------------   480
Spr1    --------------------------------------------------------------------------------   497
Yin1    --------------------------------------------------------------------------------   751
Yin2    --------------------------------------------------------------------------------   753
Yfr4    --------------------------------------------------------------------------------   749
Ymo2    --------------------------------------------------------------------------------   686
Eco10   --------------------------------------------------------------------------------   734
Eal1    --------------------------------------------------------------------------------   725
Sty4    --------------------------------------------------------------------------------   626
Eco16   --------------------------------------------------------------------------------   711
Eco25   --------------------------------------------------------------------------------  2660
Eco15   --------------------------------------------------------------------------------  1700
Eco6    --------------------------------------------------------------------------------  2836
Efe2    --------------------------------------------------------------------------------  1089
Efe3    --------------------------------------------------------------------------------  2104
Yfr1    --------------------------------------------------------------------------------   770
Yfr5    --------------------------------------------------------------------------------   780
Yps4    GAAITTVNGISDGDGLTKATLTHTLAGTSVVTARVGNQVQSKDTTFIADRTTATIRASDLTITRSNALADGVATNAARVI  4368
Yps7    --------------------------------------------------------------------------------  1486
Yfr2    --------------------------------------------------------------------------------   906
Yfr3    --------------------------------------------------------------------------------   851
Yps2    --------------------------------------------------------------------------------   985
Ype5    --------------------------------------------------------------------------------  1050
Pru1    --------------------------------------------------------------------------------  2373
Pal3    --------------------------------------------------------------------------------   542
Eta1    --------------------------------------------------------------------------------  1400
Sgl1    --------------------------------------------------------------------------------   934
Eco26   --------------------------------------------------------------------------------  1653
Pmi1    --------------------------------------------------------------------------------  2358
Eco1    --------------------------------------------------------------------------------   937
Ymo1    --------------------------------------------------------------------------------  1424
Yen2    --------------------------------------------------------------------------------  2484
Ybe1    --------------------------------------------------------------------------------   945
Esa2    --------------------------------------------------------------------------------  1027
Sen3    --------------------------------------------------------------------------------  1812
Eco14   --------------------------------------------------------------------------------  1084
Bpe1    --------------------------------------------------------------------------------  1308
Bav2    --------------------------------------------------------------------------------  1654
Bbr1    --------------------------------------------------------------------------------   969
Bpa2    --------------------------------------------------------------------------------  1937
Bav1    --------------------------------------------------------------------------------   747
Pma1    --------------------------------------------------------------------------------   372
Pma5    --------------------------------------------------------------------------------   373
Pma3    --------------------------------------------------------------------------------   379
Pma4    --------------------------------------------------------------------------------   410
Ssp1    --------------------------------------------------------------------------------   428
Ssp2    --------------------------------------------------------------------------------   436
Eco20   --------------------------------------------------------------------------------  1418
Efe5    --------------------------------------------------------------------------------  1185
Plu1    --------------------------------------------------------------------------------  1695
Pal2    --------------------------------------------------------------------------------  2521
Ahy1    --------------------------------------------------------------------------------   916
Csu1    --------------------------------------------------------------------------------  1305
Plu2    --------------------------------------------------------------------------------   302
Rba1    --------------------------------------------------------------------------------   327
Pas1    --------------------------------------------------------------------------------   924
Cla1    --------------------------------------------------------------------------------  1459
ruler   ......6810......6820......6830......6840......6850......6860......6870......6880
```

```
Mba1   --------------------------------------------------------------------------------   331
Psp2   --------------------------------------------------------------------------------   251
Sen2   --------------------------------------------------------------------------------   660
Efe4   --------------------------------------------------------------------------------   672
Eco3   --------------------------------------------------------------------------------   417
Cko1   --------------------------------------------------------------------------------   454
Sen1   --------------------------------------------------------------------------------   460
Eca1   --------------------------------------------------------------------------------   468
Esp2   --------------------------------------------------------------------------------   468
Esa3   --------------------------------------------------------------------------------   471
Kpn1   --------------------------------------------------------------------------------   460
Pan1   --------------------------------------------------------------------------------   490
Eta2   --------------------------------------------------------------------------------   480
Spr1   --------------------------------------------------------------------------------   497
Yin1   --------------------------------------------------------------------------------   751
Yin2   --------------------------------------------------------------------------------   753
Yfr4   --------------------------------------------------------------------------------   749
Ymo2   --------------------------------------------------------------------------------   686
Eco10  --------------------------------------------------------------------------------   734
Eal1   --------------------------------------------------------------------------------   725
Sty4   --------------------------------------------------------------------------------   626
Eco16  --------------------------------------------------------------------------------   711
Eco25  --------------------------------------------------------------------------------  2660
Eco15  --------------------------------------------------------------------------------  1700
Eco6   --------------------------------------------------------------------------------  2836
Efe2   --------------------------------------------------------------------------------  1089
Efe3   --------------------------------------------------------------------------------  2104
Yfr1   --------------------------------------------------------------------------------   770
Yfr5   --------------------------------------------------------------------------------   780
Yps4   VTDAYGNPVPSMLVSYTSENGATLTPTLGSTDSSGMLSTTFTHTIAGISKVTATIVTMGISQAKDAVFIADRTTAHVSAL  4448
Yps7   --------------------------------------------------------------------------------  1486
Yfr2   --------------------------------------------------------------------------------   906
Yfr3   --------------------------------------------------------------------------------   851
Yps2   --------------------------------------------------------------------------------   985
Ype5   --------------------------------------------------------------------------------  1050
Pru1   --------------------------------------------------------------------------------  2373
Pal3   --------------------------------------------------------------------------------   542
Eta1   --------------------------------------------------------------------------------  1400
Sgl1   --------------------------------------------------------------------------------   934
Eco26  --------------------------------------------------------------------------------  1653
Pmi1   --------------------------------------------------------------------------------  2358
Eco1   --------------------------------------------------------------------------------   937
Ymo1   --------------------------------------------------------------------------------  1424
Yen2   --------------------------------------------------------------------------------  2484
Ybe1   --------------------------------------------------------------------------------   945
Esa2   --------------------------------------------------------------------------------  1027
Sen3   --------------------------------------------------------------------------------  1812
Eco14  --------------------------------------------------------------------------------  1084
Bpe1   --------------------------------------------------------------------------------  1308
Bav2   --------------------------------------------------------------------------------  1654
Bbr1   --------------------------------------------------------------------------------   969
Bpa2   --------------------------------------------------------------------------------  1937
Bav1   --------------------------------------------------------------------------------   747
Pma1   --------------------------------------------------------------------------------   372
Pma5   --------------------------------------------------------------------------------   373
Pma3   --------------------------------------------------------------------------------   379
Pma4   --------------------------------------------------------------------------------   410
Ssp1   --------------------------------------------------------------------------------   428
Ssp2   --------------------------------------------------------------------------------   436
Eco20  --------------------------------------------------------------------------------  1418
Efe5   --------------------------------------------------------------------------------  1185
Plu1   --------------------------------------------------------------------------------  1695
Pal2   --------------------------------------------------------------------------------  2521
Ahy1   --------------------------------------------------------------------------------   916
Csu1   --------------------------------------------------------------------------------  1305
Plu2   --------------------------------------------------------------------------------   302
Rba1   --------------------------------------------------------------------------------   327
Pas1   --------------------------------------------------------------------------------   924
Cla1   --------------------------------------------------------------------------------  1459
ruler  ......6890......6900......6910......6920......6930......6940......6950......6960
```

```
Mba1    --------------------------------------------------------------------------------   331
Psp2    --------------------------------------------------------------------------------   251
Sen2    --------------------------------------------------------------------------------   660
Efe4    --------------------------------------------------------------------------------   672
Eco3    --------------------------------------------------------------------------------   417
Cko1    --------------------------------------------------------------------------------   454
Sen1    --------------------------------------------------------------------------------   460
Eca1    --------------------------------------------------------------------------------   468
Esp2    --------------------------------------------------------------------------------   468
Esa3    --------------------------------------------------------------------------------   471
Kpn1    --------------------------------------------------------------------------------   460
Pan1    --------------------------------------------------------------------------------   490
Eta2    --------------------------------------------------------------------------------   480
Spr1    --------------------------------------------------------------------------------   497
Yin1    --------------------------------------------------------------------------------   751
Yin2    --------------------------------------------------------------------------------   753
Yfr4    --------------------------------------------------------------------------------   749
Ymo2    --------------------------------------------------------------------------------   686
Eco10   --------------------------------------------------------------------------------   734
Eal1    --------------------------------------------------------------------------------   725
Sty4    --------------------------------------------------------------------------------   626
Eco16   --------------------------------------------------------------------------------   711
Eco25   --------------------------------------------------------------------------------  2660
Eco15   --------------------------------------------------------------------------------  1700
Eco6    --------------------------------------------------------------------------------  2836
Efe2    --------------------------------------------------------------------------------  1089
Efe3    --------------------------------------------------------------------------------  2104
Yfr1    --------------------------------------------------------------------------------   770
Yfr5    --------------------------------------------------------------------------------   780
Yps4    TVEKNDSLANNSDRNIVQAHIQDAHGNVITGMNVNFSATENVTLAANMVTTNAQGYAENTLRHNAPVTSAVTATVATDLV  4528
Yps7    --------------------------------------------------------------------------------  1486
Yfr2    --------------------------------------------------------------------------------   906
Yfr3    --------------------------------------------------------------------------------   851
Yps2    --------------------------------------------------------------------------------   985
Ype5    --------------------------------------------------------------------------------  1050
Pru1    --------------------------------------------------------------------------------  2373
Pal3    --------------------------------------------------------------------------------   542
Eta1    --------------------------------------------------------------------------------  1400
Sgl1    --------------------------------------------------------------------------------   934
Eco26   --------------------------------------------------------------------------------  1653
Pmi1    --------------------------------------------------------------------------------  2358
Eco1    --------------------------------------------------------------------------------   937
Ymo1    --------------------------------------------------------------------------------  1424
Yen2    --------------------------------------------------------------------------------  2484
Ybe1    --------------------------------------------------------------------------------   945
Esa2    --------------------------------------------------------------------------------  1027
Sen3    --------------------------------------------------------------------------------  1812
Eco14   --------------------------------------------------------------------------------  1084
Bpe1    --------------------------------------------------------------------------------  1308
Bav2    --------------------------------------------------------------------------------  1654
Bbr1    --------------------------------------------------------------------------------   969
Bpa2    --------------------------------------------------------------------------------  1937
Bav1    --------------------------------------------------------------------------------   747
Pma1    --------------------------------------------------------------------------------   372
Pma5    --------------------------------------------------------------------------------   373
Pma3    --------------------------------------------------------------------------------   379
Pma4    --------------------------------------------------------------------------------   410
Ssp1    --------------------------------------------------------------------------------   428
Ssp2    --------------------------------------------------------------------------------   436
Eco20   --------------------------------------------------------------------------------  1418
Efe5    --------------------------------------------------------------------------------  1185
Plu1    --------------------------------------------------------------------------------  1695
Pal2    --------------------------------------------------------------------------------  2521
Ahy1    --------------------------------------------------------------------------------   916
Csu1    --------------------------------------------------------------------------------  1305
Plu2    --------------------------------------------------------------------------------   302
Rba1    --------------------------------------------------------------------------------   327
Pas1    --------------------------------------------------------------------------------   924
Cla1    --------------------------------------------------------------------------------  1459
ruler   ......6970......6980......6990......7000......7010......7020......7030......7040
```

```
Mba1   --------------------------------------------------------------------------------   331
Psp2   --------------------------------------------------------------------------------   251
Sen2   --------------------------------------------------------------------------------   660
Efe4   --------------------------------------------------------------------------------   672
Eco3   --------------------------------------------------------------------------------   417
Cko1   --------------------------------------------------------------------------------   454
Sen1   --------------------------------------------------------------------------------   460
Eca1   --------------------------------------------------------------------------------   468
Esp2   --------------------------------------------------------------------------------   468
Esa3   --------------------------------------------------------------------------------   471
Kpn1   --------------------------------------------------------------------------------   460
Pan1   --------------------------------------------------------------------------------   490
Eta2   --------------------------------------------------------------------------------   480
Spr1   --------------------------------------------------------------------------------   497
Yin1   --------------------------------------------------------------------------------   751
Yin2   --------------------------------------------------------------------------------   753
Yfr4   --------------------------------------------------------------------------------   749
Ymo2   --------------------------------------------------------------------------------   686
Eco10  --------------------------------------------------------------------------------   734
Eal1   --------------------------------------------------------------------------------   725
Sty4   --------------------------------------------------------------------------------   626
Eco16  --------------------------------------------------------------------------------   711
Eco25  --------------------------------------------------------------------------------  2660
Eco15  --------------------------------------------------------------------------------  1700
Eco6   --------------------------------------------------------------------------------  2836
Efe2   --------------------------------------------------------------------------------  1089
Efe3   --------------------------------------------------------------------------------  2104
Yfr1   --------------------------------------------------------------------------------   770
Yfr5   --------------------------------------------------------------------------------   780
Yps4   GLTEDVRFVAGAGARIELFRLNDGAVADGIQTNRVEARVYDVSDNLVPNSNVVFSADNGGQLVQNDVQTDALGSAYVTVS  4608
Yps7   --------------------------------------------------------------------------------  1486
Yfr2   --------------------------------------------------------------------------------   906
Yfr3   --------------------------------------------------------------------------------   851
Yps2   --------------------------------------------------------------------------------   985
Ype5   --------------------------------------------------------------------------------  1050
Pru1   --------------------------------------------------------------------------------  2373
Pal3   --------------------------------------------------------------------------------   542
Eta1   --------------------------------------------------------------------------------  1400
Sgl1   --------------------------------------------------------------------------------   934
Eco26  --------------------------------------------------------------------------------  1653
Pmi1   --------------------------------------------------------------------------------  2358
Eco1   --------------------------------------------------------------------------------   937
Ymo1   --------------------------------------------------------------------------------  1424
Yen2   --------------------------------------------------------------------------------  2484
Ybe1   --------------------------------------------------------------------------------   945
Esa2   --------------------------------------------------------------------------------  1027
Sen3   --------------------------------------------------------------------------------  1812
Eco14  --------------------------------------------------------------------------------  1084
Bpe1   --------------------------------------------------------------------------------  1308
Bav2   --------------------------------------------------------------------------------  1654
Bbr1   --------------------------------------------------------------------------------   969
Bpa2   --------------------------------------------------------------------------------  1937
Bav1   --------------------------------------------------------------------------------   747
Pma1   --------------------------------------------------------------------------------   372
Pma5   --------------------------------------------------------------------------------   373
Pma3   --------------------------------------------------------------------------------   379
Pma4   --------------------------------------------------------------------------------   410
Ssp1   --------------------------------------------------------------------------------   428
Ssp2   --------------------------------------------------------------------------------   436
Eco20  --------------------------------------------------------------------------------  1418
Efe5   --------------------------------------------------------------------------------  1185
Plu1   --------------------------------------------------------------------------------  1695
Pal2   --------------------------------------------------------------------------------  2521
Ahy1   --------------------------------------------------------------------------------   916
Csu1   --------------------------------------------------------------------------------  1305
Plu2   --------------------------------------------------------------------------------   302
Rba1   --------------------------------------------------------------------------------   327
Pas1   --------------------------------------------------------------------------------   924
Cla1   --------------------------------------------------------------------------------  1459
ruler  ......7050......7060......7070......7080......7090......7100......7110......7120
```

```
  Mba1 --------------------------------------------------------------------------------  331
  Psp2 --------------------------------------------------------------------------------  251
  Sen2 --------------------------------------------------------------------------------  660
  Efe4 --------------------------------------------------------------------------------  672
  Eco3 --------------------------------------------------------------------------------  417
  Cko1 --------------------------------------------------------------------------------  454
  Sen1 --------------------------------------------------------------------------------  460
  Eca1 --------------------------------------------------------------------------------  468
  Esp2 --------------------------------------------------------------------------------  468
  Esa3 --------------------------------------------------------------------------------  471
  Kpn1 --------------------------------------------------------------------------------  460
  Pan1 --------------------------------------------------------------------------------  490
  Eta2 --------------------------------------------------------------------------------  480
  Spr1 --------------------------------------------------------------------------------  497
  Yin1 --------------------------------------------------------------------------------  751
  Yin2 --------------------------------------------------------------------------------  753
  Yfr4 --------------------------------------------------------------------------------  749
  Ymo2 --------------------------------------------------------------------------------  686
 Eco10 --------------------------------------------------------------------------------  734
  Eal1 --------------------------------------------------------------------------------  725
  Sty4 --------------------------------------------------------------------------------  626
 Eco16 --------------------------------------------------------------------------------  711
 Eco25 -------------------------------------------------------------------------------- 2660
 Eco15 -------------------------------------------------------------------------------- 1700
  Eco6 -------------------------------------------------------------------------------- 2836
  Efe2 -------------------------------------------------------------------------------- 1089
  Efe3 -------------------------------------------------------------------------------- 2104
  Yfr1 --------------------------------------------------------------------------------  770
  Yfr5 --------------------------------------------------------------------------------  780
  Yps4 NINTGVTKVSVTADGVSASTTTTFIADKDTVTLRADLFLITHDNAVANGVTENRVLLQLLDANDNKVSGVEVNFTATNGA 4688
  Yps7 -------------------------------------------------------------------------------- 1486
  Yfr2 --------------------------------------------------------------------------------  906
  Yfr3 --------------------------------------------------------------------------------  851
  Yps2 --------------------------------------------------------------------------------  985
  Ype5 -------------------------------------------------------------------------------- 1050
  Pru1 -------------------------------------------------------------------------------- 2373
  Pal3 --------------------------------------------------------------------------------  542
  Eta1 -------------------------------------------------------------------------------- 1400
  Sgl1 --------------------------------------------------------------------------------  934
 Eco26 -------------------------------------------------------------------------------- 1653
  Pmi1 -------------------------------------------------------------------------------- 2358
  Eco1 --------------------------------------------------------------------------------  937
  Ymo1 -------------------------------------------------------------------------------- 1424
  Yen2 -------------------------------------------------------------------------------- 2484
  Ybe1 --------------------------------------------------------------------------------  945
  Esa2 -------------------------------------------------------------------------------- 1027
  Sen3 -------------------------------------------------------------------------------- 1812
 Eco14 -------------------------------------------------------------------------------- 1084
  Bpe1 -------------------------------------------------------------------------------- 1308
  Bav2 -------------------------------------------------------------------------------- 1654
  Bbr1 --------------------------------------------------------------------------------  969
  Bpa2 -------------------------------------------------------------------------------- 1937
  Bav1 --------------------------------------------------------------------------------  747
  Pma1 --------------------------------------------------------------------------------  372
  Pma5 --------------------------------------------------------------------------------  373
  Pma3 --------------------------------------------------------------------------------  379
  Pma4 --------------------------------------------------------------------------------  410
  Ssp1 --------------------------------------------------------------------------------  428
  Ssp2 --------------------------------------------------------------------------------  436
 Eco20 -------------------------------------------------------------------------------- 1418
  Efe5 -------------------------------------------------------------------------------- 1185
  Plu1 -------------------------------------------------------------------------------- 1695
  Pal2 -------------------------------------------------------------------------------- 2521
  Ahy1 --------------------------------------------------------------------------------  916
  Csu1 -------------------------------------------------------------------------------- 1305
  Plu2 --------------------------------------------------------------------------------  302
  Rba1 --------------------------------------------------------------------------------  327
  Pas1 --------------------------------------------------------------------------------  924
  Cla1 -------------------------------------------------------------------------------- 1459
 ruler ......7130......7140......7150......7160......7170......7180......7190......7200
```

```
  Mba1 --------------------------------------------------------------------------------   331
  Psp2 --------------------------------------------------------------------------------   251
  Sen2 --------------------------------------------------------------------------------   660
  Efe4 --------------------------------------------------------------------------------   672
  Eco3 --------------------------------------------------------------------------------   417
  Cko1 --------------------------------------------------------------------------------   454
  Sen1 --------------------------------------------------------------------------------   460
  Eca1 --------------------------------------------------------------------------------   468
  Esp2 --------------------------------------------------------------------------------   468
  Esa3 --------------------------------------------------------------------------------   471
  Kpn1 --------------------------------------------------------------------------------   460
  Pan1 --------------------------------------------------------------------------------   490
  Eta2 --------------------------------------------------------------------------------   480
  Spr1 --------------------------------------------------------------------------------   497
  Yin1 --------------------------------------------------------------------------------   751
  Yin2 --------------------------------------------------------------------------------   753
  Yfr4 --------------------------------------------------------------------------------   749
  Ymo2 --------------------------------------------------------------------------------   686
 Eco10 --------------------------------------------------------------------------------   734
  Eal1 --------------------------------------------------------------------------------   725
  Sty4 --------------------------------------------------------------------------------   626
 Eco16 --------------------------------------------------------------------------------   711
 Eco25 --------------------------------------------------------------------------------  2660
 Eco15 --------------------------------------------------------------------------------  1700
  Eco6 --------------------------------------------------------------------------------  2836
  Efe2 --------------------------------------------------------------------------------  1089
  Efe3 --------------------------------------------------------------------------------  2104
  Yfr1 --------------------------------------------------------------------------------   770
  Yfr5 --------------------------------------------------------------------------------   780
  Yps4 SINASAITDTNGLAIGVLTNTLSGPSDVTVTLVTPGGTESLTVTPQFIADINTARIANGDFVIIDDGAVANSVDANEVRA  4768
  Yps7 --------------------------------------------------------------------------------  1486
  Yfr2 --------------------------------------------------------------------------------   906
  Yfr3 --------------------------------------------------------------------------------   851
  Yps2 --------------------------------------------------------------------------------   985
  Ype5 --------------------------------------------------------------------------------  1050
  Pru1 --------------------------------------------------------------------------------  2373
  Pal3 --------------------------------------------------------------------------------   542
  Eta1 --------------------------------------------------------------------------------  1400
  Sgl1 --------------------------------------------------------------------------------   934
 Eco26 --------------------------------------------------------------------------------  1653
  Pmi1 --------------------------------------------------------------------------------  2358
  Eco1 --------------------------------------------------------------------------------   937
  Ymo1 --------------------------------------------------------------------------------  1424
  Yen2 --------------------------------------------------------------------------------  2484
  Ybe1 --------------------------------------------------------------------------------   945
  Esa2 --------------------------------------------------------------------------------  1027
  Sen3 --------------------------------------------------------------------------------  1812
 Eco14 --------------------------------------------------------------------------------  1084
  Bpe1 --------------------------------------------------------------------------------  1308
  Bav2 --------------------------------------------------------------------------------  1654
  Bbr1 --------------------------------------------------------------------------------   969
  Bpa2 --------------------------------------------------------------------------------  1937
  Bav1 --------------------------------------------------------------------------------   747
  Pma1 --------------------------------------------------------------------------------   372
  Pma5 --------------------------------------------------------------------------------   373
  Pma3 --------------------------------------------------------------------------------   379
  Pma4 --------------------------------------------------------------------------------   410
  Ssp1 --------------------------------------------------------------------------------   428
  Ssp2 --------------------------------------------------------------------------------   436
 Eco20 --------------------------------------------------------------------------------  1418
  Efe5 --------------------------------------------------------------------------------  1185
  Plu1 --------------------------------------------------------------------------------  1695
  Pal2 --------------------------------------------------------------------------------  2521
  Ahy1 --------------------------------------------------------------------------------   916
  Csu1 --------------------------------------------------------------------------------  1305
  Plu2 --------------------------------------------------------------------------------   302
  Rba1 --------------------------------------------------------------------------------   327
  Pas1 --------------------------------------------------------------------------------   924
  Cla1 --------------------------------------------------------------------------------  1459
 ruler ......7210......7220......7230......7240......7250......7260......7270......7280
```

```
Mba1   --------------------------------------------------------------------------------   331
Psp2   --------------------------------------------------------------------------------   251
Sen2   --------------------------------------------------------------------------------   660
Efe4   --------------------------------------------------------------------------------   672
Eco3   --------------------------------------------------------------------------------   417
Cko1   --------------------------------------------------------------------------------   454
Sen1   --------------------------------------------------------------------------------   460
Eca1   --------------------------------------------------------------------------------   468
Esp2   --------------------------------------------------------------------------------   468
Esa3   --------------------------------------------------------------------------------   471
Kpn1   --------------------------------------------------------------------------------   460
Pan1   --------------------------------------------------------------------------------   490
Eta2   --------------------------------------------------------------------------------   480
Spr1   --------------------------------------------------------------------------------   497
Yin1   --------------------------------------------------------------------------------   751
Yin2   --------------------------------------------------------------------------------   753
Yfr4   --------------------------------------------------------------------------------   749
Ymo2   --------------------------------------------------------------------------------   686
Eco10  --------------------------------------------------------------------------------   734
Eal1   --------------------------------------------------------------------------------   725
Sty4   --------------------------------------------------------------------------------   626
Eco16  --------------------------------------------------------------------------------   711
Eco25  --------------------------------------------------------------------------------  2660
Eco15  --------------------------------------------------------------------------------  1700
Eco6   --------------------------------------------------------------------------------  2836
Efe2   --------------------------------------------------------------------------------  1089
Efe3   --------------------------------------------------------------------------------  2104
Yfr1   --------------------------------------------------------------------------------   770
Yfr5   --------------------------------------------------------------------------------   780
Yps4   RVTDNQGNAIAGYSVTFASQNGATITTSGITGVDGWASAKLTHTKAGESGILARISRPGSMVQVLTPYFIADVSTATLQL  4848
Yps7   --------------------------------------------------------------------------------  1486
Yfr2   --------------------------------------------------------------------------------   906
Yfr3   --------------------------------------------------------------------------------   851
Yps2   --------------------------------------------------------------------------------   985
Ype5   --------------------------------------------------------------------------------  1050
Pru1   --------------------------------------------------------------------------------  2373
Pal3   --------------------------------------------------------------------------------   542
Eta1   --------------------------------------------------------------------------------  1400
Sgl1   --------------------------------------------------------------------------------   934
Eco26  --------------------------------------------------------------------------------  1653
Pmi1   --------------------------------------------------------------------------------  2358
Eco1   --------------------------------------------------------------------------------   937
Ymo1   --------------------------------------------------------------------------------  1424
Yen2   --------------------------------------------------------------------------------  2484
Ybe1   --------------------------------------------------------------------------------   945
Esa2   --------------------------------------------------------------------------------  1027
Sen3   --------------------------------------------------------------------------------  1812
Eco14  --------------------------------------------------------------------------------  1084
Bpe1   --------------------------------------------------------------------------------  1308
Bav2   --------------------------------------------------------------------------------  1654
Bbr1   --------------------------------------------------------------------------------   969
Bpa2   --------------------------------------------------------------------------------  1937
Bav1   --------------------------------------------------------------------------------   747
Pma1   --------------------------------------------------------------------------------   372
Pma5   --------------------------------------------------------------------------------   373
Pma3   --------------------------------------------------------------------------------   379
Pma4   --------------------------------------------------------------------------------   410
Ssp1   --------------------------------------------------------------------------------   428
Ssp2   --------------------------------------------------------------------------------   436
Eco20  --------------------------------------------------------------------------------  1418
Efe5   --------------------------------------------------------------------------------  1185
Plu1   --------------------------------------------------------------------------------  1695
Pal2   --------------------------------------------------------------------------------  2521
Ahy1   --------------------------------------------------------------------------------   916
Csu1   --------------------------------------------------------------------------------  1305
Plu2   --------------------------------------------------------------------------------   302
Rba1   --------------------------------------------------------------------------------   327
Pas1   --------------------------------------------------------------------------------   924
Cla1   --------------------------------------------------------------------------------  1459
ruler  ......7290......7300......7310......7320......7330......7340......7350......7360
```

```
Mba1   --------------------------------------------------------------------------------  331
Psp2   --------------------------------------------------------------------------------  251
Sen2   --------------------------------------------------------------------------------  660
Efe4   --------------------------------------------------------------------------------  672
Eco3   --------------------------------------------------------------------------------  417
Cko1   --------------------------------------------------------------------------------  454
Sen1   --------------------------------------------------------------------------------  460
Eca1   --------------------------------------------------------------------------------  468
Esp2   --------------------------------------------------------------------------------  468
Esa3   --------------------------------------------------------------------------------  471
Kpn1   --------------------------------------------------------------------------------  460
Pan1   --------------------------------------------------------------------------------  490
Eta2   --------------------------------------------------------------------------------  480
Spr1   --------------------------------------------------------------------------------  497
Yin1   --------------------------------------------------------------------------------  751
Yin2   --------------------------------------------------------------------------------  753
Yfr4   --------------------------------------------------------------------------------  749
Ymo2   --------------------------------------------------------------------------------  686
Eco10  --------------------------------------------------------------------------------  734
Eal1   --------------------------------------------------------------------------------  725
Sty4   --------------------------------------------------------------------------------  626
Eco16  --------------------------------------------------------------------------------  711
Eco25  --------------------------------------------------------------------------------  2660
Eco15  --------------------------------------------------------------------------------  1700
Eco6   --------------------------------------------------------------------------------  2836
Efe2   --------------------------------------------------------------------------------  1089
Efe3   --------------------------------------------------------------------------------  2104
Yfr1   --------------------------------------------------------------------------------  770
Yfr5   --------------------------------------------------------------------------------  780
Yps4   FNFNPIPIIADGVMQFFVLGRVFDANQNPVGGQQVAFSATNEVTLTESNGSISTPEGSVLLSVTSTQAGVHPITGTLVSN  4928
Yps7   --------------------------------------------------------------------------------  1486
Yfr2   --------------------------------------------------------------------------------  906
Yfr3   --------------------------------------------------------------------------------  851
Yps2   --------------------------------------------------------------------------------  985
Ype5   --------------------------------------------------------------------------------  1050
Pru1   --------------------------------------------------------------------------------  2373
Pal3   --------------------------------------------------------------------------------  542
Eta1   --------------------------------------------------------------------------------  1400
Sgl1   --------------------------------------------------------------------------------  934
Eco26  --------------------------------------------------------------------------------  1653
Pmi1   --------------------------------------------------------------------------------  2358
Eco1   --------------------------------------------------------------------------------  937
Ymo1   --------------------------------------------------------------------------------  1424
Yen2   --------------------------------------------------------------------------------  2484
Ybe1   --------------------------------------------------------------------------------  945
Esa2   --------------------------------------------------------------------------------  1027
Sen3   --------------------------------------------------------------------------------  1812
Eco14  --------------------------------------------------------------------------------  1084
Bpe1   --------------------------------------------------------------------------------  1308
Bav2   --------------------------------------------------------------------------------  1654
Bbr1   --------------------------------------------------------------------------------  969
Bpa2   --------------------------------------------------------------------------------  1937
Bav1   --------------------------------------------------------------------------------  747
Pma1   --------------------------------------------------------------------------------  372
Pma5   --------------------------------------------------------------------------------  373
Pma3   --------------------------------------------------------------------------------  379
Pma4   --------------------------------------------------------------------------------  410
Ssp1   --------------------------------------------------------------------------------  428
Ssp2   --------------------------------------------------------------------------------  436
Eco20  --------------------------------------------------------------------------------  1418
Efe5   --------------------------------------------------------------------------------  1185
Plu1   --------------------------------------------------------------------------------  1695
Pal2   --------------------------------------------------------------------------------  2521
Ahy1   --------------------------------------------------------------------------------  916
Csu1   --------------------------------------------------------------------------------  1305
Plu2   --------------------------------------------------------------------------------  302
Rba1   --------------------------------------------------------------------------------  327
Pas1   --------------------------------------------------------------------------------  924
Cla1   --------------------------------------------------------------------------------  1459
ruler  ......7370......7380......7390......7400......7410......7420......7430......7440
```

```
  Mba1 --------------------------------------------------------------------------------  331
  Psp2 --------------------------------------------------------------------------------  251
  Sen2 --------------------------------------------------------------------------------  660
  Efe4 --------------------------------------------------------------------------------  672
  Eco3 --------------------------------------------------------------------------------  417
  Cko1 --------------------------------------------------------------------------------  454
  Sen1 --------------------------------------------------------------------------------  460
  Eca1 --------------------------------------------------------------------------------  468
  Esp2 --------------------------------------------------------------------------------  468
  Esa3 --------------------------------------------------------------------------------  471
  Kpn1 --------------------------------------------------------------------------------  460
  Pan1 --------------------------------------------------------------------------------  490
  Eta2 --------------------------------------------------------------------------------  480
  Spr1 --------------------------------------------------------------------------------  497
  Yin1 --------------------------------------------------------------------------------  751
  Yin2 --------------------------------------------------------------------------------  753
  Yfr4 --------------------------------------------------------------------------------  749
  Ymo2 --------------------------------------------------------------------------------  686
 Eco10 --------------------------------------------------------------------------------  734
  Eal1 --------------------------------------------------------------------------------  725
  Sty4 --------------------------------------------------------------------------------  626
 Eco16 --------------------------------------------------------------------------------  711
 Eco25 -------------------------------------------------------------------------------- 2660
 Eco15 -------------------------------------------------------------------------------- 1700
  Eco6 -------------------------------------------------------------------------------- 2836
  Efe2 -------------------------------------------------------------------------------- 1089
  Efe3 -------------------------------------------------------------------------------- 2104
  Yfr1 --------------------------------------------------------------------------------  770
  Yfr5 --------------------------------------------------------------------------------  780
  Yps4 NYTDTFGATFIANKNTAQLSTLMVVDNNALADGVTRNQVRAHVVDSTGNSVADIAVTFTANHGAQLSHVTVLTDDNGDAV 5008
  Yps7 -------------------------------------------------------------------------------- 1486
  Yfr2 --------------------------------------------------------------------------------  906
  Yfr3 --------------------------------------------------------------------------------  851
  Yps2 --------------------------------------------------------------------------------  985
  Ype5 -------------------------------------------------------------------------------- 1050
  Pru1 -------------------------------------------------------------------------------- 2373
  Pal3 --------------------------------------------------------------------------------  542
  Eta1 -------------------------------------------------------------------------------- 1400
  Sgl1 --------------------------------------------------------------------------------  934
 Eco26 -------------------------------------------------------------------------------- 1653
  Pmi1 -------------------------------------------------------------------------------- 2358
  Eco1 --------------------------------------------------------------------------------  937
  Ymo1 -------------------------------------------------------------------------------- 1424
  Yen2 -------------------------------------------------------------------------------- 2484
  Ybe1 --------------------------------------------------------------------------------  945
  Esa2 -------------------------------------------------------------------------------- 1027
  Sen3 -------------------------------------------------------------------------------- 1812
 Eco14 -------------------------------------------------------------------------------- 1084
  Bpe1 -------------------------------------------------------------------------------- 1308
  Bav2 -------------------------------------------------------------------------------- 1654
  Bbr1 --------------------------------------------------------------------------------  969
  Bpa2 -------------------------------------------------------------------------------- 1937
  Bav1 --------------------------------------------------------------------------------  747
  Pma1 --------------------------------------------------------------------------------  372
  Pma5 --------------------------------------------------------------------------------  373
  Pma3 --------------------------------------------------------------------------------  379
  Pma4 --------------------------------------------------------------------------------  410
  Ssp1 --------------------------------------------------------------------------------  428
  Ssp2 --------------------------------------------------------------------------------  436
 Eco20 -------------------------------------------------------------------------------- 1418
  Efe5 -------------------------------------------------------------------------------- 1185
  Plu1 -------------------------------------------------------------------------------- 1695
  Pal2 -------------------------------------------------------------------------------- 2521
  Ahy1 --------------------------------------------------------------------------------  916
  Csu1 -------------------------------------------------------------------------------- 1305
  Plu2 --------------------------------------------------------------------------------  302
  Rba1 --------------------------------------------------------------------------------  327
  Pas1 --------------------------------------------------------------------------------  924
  Cla1 -------------------------------------------------------------------------------- 1459
 ruler ......7450......7460......7470......7480......7490......7500......7510......7520
```

```
Mba1   --------------------------------------------------------------------------------   331
Psp2   --------------------------------------------------------------------------------   251
Sen2   --------------------------------------------------------------------------------   660
Efe4   --------------------------------------------------------------------------------   672
Eco3   --------------------------------------------------------------------------------   417
Cko1   --------------------------------------------------------------------------------   454
Sen1   --------------------------------------------------------------------------------   460
Eca1   --------------------------------------------------------------------------------   468
Esp2   --------------------------------------------------------------------------------   468
Esa3   --------------------------------------------------------------------------------   471
Kpn1   --------------------------------------------------------------------------------   460
Pan1   --------------------------------------------------------------------------------   490
Eta2   --------------------------------------------------------------------------------   480
Spr1   --------------------------------------------------------------------------------   497
Yin1   --------------------------------------------------------------------------------   751
Yin2   --------------------------------------------------------------------------------   753
Yfr4   --------------------------------------------------------------------------------   749
Ymo2   --------------------------------------------------------------------------------   686
Eco10  --------------------------------------------------------------------------------   734
Eal1   --------------------------------------------------------------------------------   725
Sty4   --------------------------------------------------------------------------------   626
Eco16  --------------------------------------------------------------------------------   711
Eco25  --------------------------------------------------------------------------------  2660
Eco15  --------------------------------------------------------------------------------  1700
Eco6   --------------------------------------------------------------------------------  2836
Efe2   --------------------------------------------------------------------------------  1089
Efe3   --------------------------------------------------------------------------------  2104
Yfr1   --------------------------------------------------------------------------------   770
Yfr5   --------------------------------------------------------------------------------   780
Yps4   NTLTNSLVGVTVVTAKLGTAGTPLTVDTVFTAGPLATLTLVTMVDNAFADNSATNTVQATLKDATGNPIVGEVVAFAASN  5088
Yps7   --------------------------------------------------------------------------------  1486
Yfr2   --------------------------------------------------------------------------------   906
Yfr3   --------------------------------------------------------------------------------   851
Yps2   --------------------------------------------------------------------------------   985
Ype5   --------------------------------------------------------------------------------  1050
Pru1   --------------------------------------------------------------------------------  2373
Pal3   --------------------------------------------------------------------------------   542
Eta1   --------------------------------------------------------------------------------  1400
Sgl1   --------------------------------------------------------------------------------   934
Eco26  --------------------------------------------------------------------------------  1653
Pmi1   --------------------------------------------------------------------------------  2358
Eco1   --------------------------------------------------------------------------------   937
Ymo1   --------------------------------------------------------------------------------  1424
Yen2   --------------------------------------------------------------------------------  2484
Ybe1   --------------------------------------------------------------------------------   945
Esa2   --------------------------------------------------------------------------------  1027
Sen3   --------------------------------------------------------------------------------  1812
Eco14  --------------------------------------------------------------------------------  1084
Bpe1   --------------------------------------------------------------------------------  1308
Bav2   --------------------------------------------------------------------------------  1654
Bbr1   --------------------------------------------------------------------------------   969
Bpa2   --------------------------------------------------------------------------------  1937
Bav1   --------------------------------------------------------------------------------   747
Pma1   --------------------------------------------------------------------------------   372
Pma5   --------------------------------------------------------------------------------   373
Pma3   --------------------------------------------------------------------------------   379
Pma4   --------------------------------------------------------------------------------   410
Ssp1   --------------------------------------------------------------------------------   428
Ssp2   --------------------------------------------------------------------------------   436
Eco20  --------------------------------------------------------------------------------  1418
Efe5   --------------------------------------------------------------------------------  1185
Plu1   --------------------------------------------------------------------------------  1695
Pal2   --------------------------------------------------------------------------------  2521
Ahy1   --------------------------------------------------------------------------------   916
Csu1   --------------------------------------------------------------------------------  1305
Plu2   --------------------------------------------------------------------------------   302
Rba1   --------------------------------------------------------------------------------   327
Pas1   --------------------------------------------------------------------------------   924
Cla1   --------------------------------------------------------------------------------  1459
ruler  ......7530......7540......7550......7560......7570......7580......7590......7600
```

```
  Mba1 --------------------------------------------------------------------------------  331
  Psp2 --------------------------------------------------------------------------------  251
  Sen2 --------------------------------------------------------------------------------  660
  Efe4 --------------------------------------------------------------------------------  672
  Eco3 --------------------------------------------------------------------------------  417
  Cko1 --------------------------------------------------------------------------------  454
  Sen1 --------------------------------------------------------------------------------  460
  Eca1 --------------------------------------------------------------------------------  468
  Esp2 --------------------------------------------------------------------------------  468
  Esa3 --------------------------------------------------------------------------------  471
  Kpn1 --------------------------------------------------------------------------------  460
  Pan1 --------------------------------------------------------------------------------  490
  Eta2 --------------------------------------------------------------------------------  480
  Spr1 --------------------------------------------------------------------------------  497
  Yin1 --------------------------------------------------------------------------------  751
  Yin2 --------------------------------------------------------------------------------  753
  Yfr4 --------------------------------------------------------------------------------  749
  Ymo2 --------------------------------------------------------------------------------  686
 Eco10 --------------------------------------------------------------------------------  734
  Eal1 --------------------------------------------------------------------------------  725
  Sty4 --------------------------------------------------------------------------------  626
 Eco16 --------------------------------------------------------------------------------  711
 Eco25 -------------------------------------------------------------------------------- 2660
 Eco15 -------------------------------------------------------------------------------- 1700
  Eco6 -------------------------------------------------------------------------------- 2836
  Efe2 -------------------------------------------------------------------------------- 1089
  Efe3 -------------------------------------------------------------------------------- 2104
  Yfr1 --------------------------------------------------------------------------------  770
  Yfr5 --------------------------------------------------------------------------------  780
  Yps4 GATITATDGGVSNANGIVLATLTNGAAGVSTVTATIETLTATTETTFIAMKNLDVTVGDTTFDGDAGFPTTGFVGAAFKV 5168
  Yps7 -------------------------------------------------------------------------------- 1486
  Yfr2 --------------------------------------------------------------------------------  906
  Yfr3 --------------------------------------------------------------------------------  851
  Yps2 --------------------------------------------------------------------------------  985
  Ype5 -------------------------------------------------------------------------------- 1050
  Pru1 -------------------------------------------------------------------------------- 2373
  Pal3 --------------------------------------------------------------------------------  542
  Eta1 -------------------------------------------------------------------------------- 1400
  Sgl1 --------------------------------------------------------------------------------  934
 Eco26 -------------------------------------------------------------------------------- 1653
  Pmi1 -------------------------------------------------------------------------------- 2358
  Eco1 --------------------------------------------------------------------------------  937
  Ymo1 -------------------------------------------------------------------------------- 1424
  Yen2 -------------------------------------------------------------------------------- 2484
  Ybe1 --------------------------------------------------------------------------------  945
  Esa2 -------------------------------------------------------------------------------- 1027
  Sen3 -------------------------------------------------------------------------------- 1812
 Eco14 -------------------------------------------------------------------------------- 1084
  Bpe1 -------------------------------------------------------------------------------- 1308
  Bav2 -------------------------------------------------------------------------------- 1654
  Bbr1 --------------------------------------------------------------------------------  969
  Bpa2 -------------------------------------------------------------------------------- 1937
  Bav1 --------------------------------------------------------------------------------  747
  Pma1 --------------------------------------------------------------------------------  372
  Pma5 --------------------------------------------------------------------------------  373
  Pma3 --------------------------------------------------------------------------------  379
  Pma4 --------------------------------------------------------------------------------  410
  Ssp1 --------------------------------------------------------------------------------  428
  Ssp2 --------------------------------------------------------------------------------  436
 Eco20 -------------------------------------------------------------------------------- 1418
  Efe5 -------------------------------------------------------------------------------- 1185
  Plu1 -------------------------------------------------------------------------------- 1695
  Pal2 -------------------------------------------------------------------------------- 2521
  Ahy1 --------------------------------------------------------------------------------  916
  Csu1 -------------------------------------------------------------------------------- 1305
  Plu2 --------------------------------------------------------------------------------  302
  Rba1 --------------------------------------------------------------------------------  327
  Pas1 --------------------------------------------------------------------------------  924
  Cla1 -------------------------------------------------------------------------------- 1459
 ruler ......7610......7620......7630......7640......7650......7660......7670......7680
```

```
  Mba1 --------------------------------------------------------------------------------   331
  Psp2 --------------------------------------------------------------------------------   251
  Sen2 --------------------------------------------------------------------------------   660
  Efe4 --------------------------------------------------------------------------------   672
  Eco3 --------------------------------------------------------------------------------   417
  Cko1 --------------------------------------------------------------------------------   454
  Sen1 --------------------------------------------------------------------------------   460
  Eca1 --------------------------------------------------------------------------------   468
  Esp2 --------------------------------------------------------------------------------   468
  Esa3 --------------------------------------------------------------------------------   471
  Kpn1 --------------------------------------------------------------------------------   460
  Pan1 --------------------------------------------------------------------------------   490
  Eta2 --------------------------------------------------------------------------------   480
  Spr1 --------------------------------------------------------------------------------   497
  Yin1 --------------------------------------------------------------------------------   751
  Yin2 --------------------------------------------------------------------------------   753
  Yfr4 --------------------------------------------------------------------------------   749
  Ymo2 --------------------------------------------------------------------------------   686
 Eco10 --------------------------------------------------------------------------------   734
  Eal1 --------------------------------------------------------------------------------   725
  Sty4 --------------------------------------------------------------------------------   626
 Eco16 --------------------------------------------------------------------------------   711
 Eco25 --------------------------------------------------------------------------------  2660
 Eco15 --------------------------------------------------------------------------------  1700
  Eco6 --------------------------------------------------------------------------------  2836
  Efe2 --------------------------------------------------------------------------------  1089
  Efe3 --------------------------------------------------------------------------------  2104
  Yfr1 --------------------------------------------------------------------------------   770
  Yfr5 --------------------------------------------------------------------------------   780
  Yps4 NSGGDNSLYDWSSSAPALVSVSGEGVVTFNAVFPTGTPAITISATPKGGGSPLSYSFRVNQWFINNNGVALNRADAATYC  5248
  Yps7 --------------------------------------------------------------------------------  1486
  Yfr2 --------------------------------------------------------------------------------   906
  Yfr3 --------------------------------------------------------------------------------   851
  Yps2 --------------------------------------------------------------------------------   985
  Ype5 --------------------------------------------------------------------------------  1050
  Pru1 --------------------------------------------------------------------------------  2373
  Pal3 --------------------------------------------------------------------------------   542
  Eta1 --------------------------------------------------------------------------------  1400
  Sgl1 --------------------------------------------------------------------------------   934
 Eco26 --------------------------------------------------------------------------------  1653
  Pmi1 --------------------------------------------------------------------------------  2358
  Eco1 --------------------------------------------------------------------------------   937
  Ymo1 --------------------------------------------------------------------------------  1424
  Yen2 --------------------------------------------------------------------------------  2484
  Ybe1 --------------------------------------------------------------------------------   945
  Esa2 --------------------------------------------------------------------------------  1027
  Sen3 --------------------------------------------------------------------------------  1812
 Eco14 --------------------------------------------------------------------------------  1084
  Bpe1 --------------------------------------------------------------------------------  1308
  Bav2 --------------------------------------------------------------------------------  1654
  Bbr1 --------------------------------------------------------------------------------   969
  Bpa2 --------------------------------------------------------------------------------  1937
  Bav1 --------------------------------------------------------------------------------   747
  Pma1 --------------------------------------------------------------------------------   372
  Pma5 --------------------------------------------------------------------------------   373
  Pma3 --------------------------------------------------------------------------------   379
  Pma4 --------------------------------------------------------------------------------   410
  Ssp1 --------------------------------------------------------------------------------   428
  Ssp2 --------------------------------------------------------------------------------   436
 Eco20 --------------------------------------------------------------------------------  1418
  Efe5 --------------------------------------------------------------------------------  1185
  Plu1 --------------------------------------------------------------------------------  1695
  Pal2 --------------------------------------------------------------------------------  2521
  Ahy1 --------------------------------------------------------------------------------   916
  Csu1 --------------------------------------------------------------------------------  1305
  Plu2 --------------------------------------------------------------------------------   302
  Rba1 --------------------------------------------------------------------------------   327
  Pas1 --------------------------------------------------------------------------------   924
  Cla1 --------------------------------------------------------------------------------  1459
 ruler ......7690......7700......7710......7720......7730......7740......7750......7760
```

```
   Mba1 --------------------------------------------------------------------------------  331
   Psp2 --------------------------------------------------------------------------------  251
   Sen2 --------------------------------------------------------------------------------  660
   Efe4 --------------------------------------------------------------------------------  672
   Eco3 --------------------------------------------------------------------------------  417
   Cko1 --------------------------------------------------------------------------------  454
   Sen1 --------------------------------------------------------------------------------  460
   Eca1 --------------------------------------------------------------------------------  468
   Esp2 --------------------------------------------------------------------------------  468
   Esa3 --------------------------------------------------------------------------------  471
   Kpn1 --------------------------------------------------------------------------------  460
   Pan1 --------------------------------------------------------------------------------  490
   Eta2 --------------------------------------------------------------------------------  480
   Spr1 --------------------------------------------------------------------------------  497
   Yin1 --------------------------------------------------------------------------------  751
   Yin2 --------------------------------------------------------------------------------  753
   Yfr4 --------------------------------------------------------------------------------  749
   Ymo2 --------------------------------------------------------------------------------  686
  Eco10 --------------------------------------------------------------------------------  734
   Eal1 --------------------------------------------------------------------------------  725
   Sty4 --------------------------------------------------------------------------------  626
  Eco16 --------------------------------------------------------------------------------  711
  Eco25 -------------------------------------------------------------------------------- 2660
  Eco15 -------------------------------------------------------------------------------- 1700
   Eco6 -------------------------------------------------------------------------------- 2836
   Efe2 -------------------------------------------------------------------------------- 1089
   Efe3 -------------------------------------------------------------------------------- 2104
   Yfr1 --------------------------------------------------------------------------------  770
   Yfr5 --------------------------------------------------------------------------------  780
   Yps4 ANAGYTTVSSSQVTNAIVWGMGTRAMGNLWSEWGDFNNYNVPGWEPAEFFWLSDNYNATDGLAASLSHGVLTTMGDPMAM 5328
   Yps7 -------------------------------------------------------------------------------- 1486
   Yfr2 --------------------------------------------------------------------------------  906
   Yfr3 --------------------------------------------------------------------------------  851
   Yps2 --------------------------------------------------------------------------------  985
   Ype5 -------------------------------------------------------------------------------- 1050
   Pru1 -------------------------------------------------------------------------------- 2373
   Pal3 --------------------------------------------------------------------------------  542
   Eta1 -------------------------------------------------------------------------------- 1400
   Sgl1 --------------------------------------------------------------------------------  934
  Eco26 -------------------------------------------------------------------------------- 1653
   Pmi1 -------------------------------------------------------------------------------- 2358
   Eco1 --------------------------------------------------------------------------------  937
   Ymo1 -------------------------------------------------------------------------------- 1424
   Yen2 -------------------------------------------------------------------------------- 2484
   Ybe1 --------------------------------------------------------------------------------  945
   Esa2 -------------------------------------------------------------------------------- 1027
   Sen3 -------------------------------------------------------------------------------- 1812
  Eco14 -------------------------------------------------------------------------------- 1084
   Bpe1 -------------------------------------------------------------------------------- 1308
   Bav2 -------------------------------------------------------------------------------- 1654
   Bbr1 --------------------------------------------------------------------------------  969
   Bpa2 -------------------------------------------------------------------------------- 1937
   Bav1 --------------------------------------------------------------------------------  747
   Pma1 --------------------------------------------------------------------------------  372
   Pma5 --------------------------------------------------------------------------------  373
   Pma3 --------------------------------------------------------------------------------  379
   Pma4 --------------------------------------------------------------------------------  410
   Ssp1 --------------------------------------------------------------------------------  428
   Ssp2 --------------------------------------------------------------------------------  436
  Eco20 -------------------------------------------------------------------------------- 1418
   Efe5 -------------------------------------------------------------------------------- 1185
   Plu1 -------------------------------------------------------------------------------- 1695
   Pal2 -------------------------------------------------------------------------------- 2521
   Ahy1 --------------------------------------------------------------------------------  916
   Csu1 -------------------------------------------------------------------------------- 1305
   Plu2 --------------------------------------------------------------------------------  302
   Rba1 --------------------------------------------------------------------------------  327
   Pas1 --------------------------------------------------------------------------------  924
   Cla1 -------------------------------------------------------------------------------- 1459
  ruler ......7770......7780......7790......7800......7810......7820......7830......7840
```

```
  Mba1 ---------   331
  Psp2 ---------   251
  Sen2 ---------   660
  Efe4 ---------   672
  Eco3 ---------   417
  Cko1 ---------   454
  Sen1 ---------   460
  Eca1 ---------   468
  Esp2 ---------   468
  Esa3 ---------   471
  Kpn1 ---------   460
  Pan1 ---------   490
  Eta2 ---------   480
  Spr1 ---------   497
  Yin1 ---------   751
  Yin2 ---------   753
  Yfr4 ---------   749
  Ymo2 ---------   686
 Eco10 ---------   734
  Eal1 ---------   725
  Sty4 ---------   626
 Eco16 ---------   711
 Eco25 ---------  2660
 Eco15 ---------  1700
  Eco6 ---------  2836
  Efe2 ---------  1089
  Efe3 ---------  2104
  Yfr1 ---------   770
  Yfr5 ---------   780
  Yps4 IHVMCTRPI  5337
  Yps7 ---------  1486
  Yfr2 ---------   906
  Yfr3 ---------   851
  Yps2 ---------   985
  Ype5 ---------  1050
  Pru1 ---------  2373
  Pal3 ---------   542
  Eta1 ---------  1400
  Sgl1 ---------   934
 Eco26 ---------  1653
  Pmi1 ---------  2358
  Eco1 ---------   937
  Ymo1 ---------  1424
  Yen2 ---------  2484
  Ybe1 ---------   945
  Esa2 ---------  1027
  Sen3 ---------  1812
 Eco14 ---------  1084
  Bpe1 ---------  1308
  Bav2 ---------  1654
  Bbr1 ---------   969
  Bpa2 ---------  1937
  Bav1 ---------   747
  Pma1 ---------   372
  Pma5 ---------   373
  Pma3 ---------   379
  Pma4 ---------   410
  Ssp1 ---------   428
  Ssp2 ---------   436
 Eco20 ---------  1418
  Efe5 ---------  1185
  Plu1 ---------  1695
  Pal2 ---------  2521
  Ahy1 ---------   916
  Csu1 ---------  1305
  Plu2 ---------   302
  Rba1 ---------   327
  Pas1 ---------   924
  Cla1 ---------  1459
 ruler .........
```
