# Supplementary material for: The Bacterial Intimins and Invasins: A Large and Novel Family of Secreted Proteins
Source: PLoS One. 2010 Dec 22;5(12):e14403. doi: 10.1371/journal.pone.0014403 (PMC3008723; doi:10.1371/journal.pone.0014403)

CLUSTAL X (1.81.1-alpha) MULTIPLE SEQUENCE ALIGNMENT

File: /Users/saierlab/Desktop/69BBcut.ps   Date: Tue May 12 18:56:54 2009


```
Mba1   ---------------------------------FGAQGGSKP-----------------------SGGLLVVAPLSDPDD   24
Psp2   ---------------------------------LSTGVTNEV-----------------------TGSVLVVKPISDPSD   24
Ybe1   --------------------------------WLNNFGTARLQANVDDRG------------DLDGSQFDMLMPFYDTPS   36
Ype5   --------------------------------WFNQFGSAKVQLNLDEKL------------SLKGSQLDVLLPLTDSPD   36
Eco14  --------------------------------WLNQFGTAKTQLSVVSDF------------SLKGSSLDVLLPFYNTPK   36
Sen3   --------------------------------WLQQFGTAKVNLGLDKDL------------SLDNASLDLLLPLYDDKK   36
Ymo1   --------------------------------WLGHIGKAQVKLQVDDKN------------DFSGSELHLFVPLYNQPE   36
Yen2   --------------------------------WLGHIGQAQVKLQTDDKN------------DFSGSEIDLFIPLYDQPE   36
Pas1   -------------------NDDVMQGSDISKSGIADMGFAALQPETEKSAGEVRANLPLSDGKLTSGSIDLFYPLYDGDS   61
Yfr1   --------------------------------WLGQYGNARVQLNSNS-----------------IGNADVLIPLTETQ-   30
Yfr5   --------------------------------WLSQFGHARVQLNSSG-----------------TGNADILLPLVESQ-   30
Yps7   --------------------------------WLSQFGTARVQLNINDDF------------HLDGSAADVLIPLYDNE-   35
Yfr2   --------------------------------WLSQFGTARVQMNVNDDF------------KLDGSAVDVLVPIYDNQ-   35
Yfr3   --------------------------------WLSQFGTARVQLNLDNNL------------SLKGSAFDMLLPLYDDQ-   35
Yps4   --------------------------------WLNQFGTARVQLNVDSDF------------KLDNSALDLLVPLKDSE-   35
Yps2   --------------------------------WLNRFGTAQVNLNFDKNF------------SLKESSLDWLAPWYDSA-   35
Esa2   --------------------------------WLSHFGTAQVTLDVDDNG------------NWDNSAFDFLAPLYDNK-   35
Plu1   --------------------------------WLSQFGTAKINLNVDHRG------------RLDESSVDLLVPFYDDKD   36
Eco1   --------------------------------WLQHYGTAEVNLQSGNN--------------FDGSSLDFLLPFYDSE-   33
Pru1   --------------------------------WFNRFGSSQIHLEADKKF------------SLKNSQIDLLMPWYETE-   35
Pal3   --------------------------------WFNHFGSSQIKLEADKKF------------SLKNSQIDLLLPLYETE-   35
Eta1   --------------------------------WLNQFGTARVQLDADEHF------------SFKNSQFDLLAPLYEQK-   35
Sgl1   --------------------------------WLSQFGTARLQLDVDNKF------------SLKNSQLDLLIPLYEQP-   35
Eco26  --------------------------------WFENFGTAHIQLQVDKNF------------SLKNSQLELLFPVFEDD-   35
Pmi1   --------------------------------WLNQFGHARVSLSADKNL------------TLKNSSAELLIPLYEQK-   35
Efe5   --------------------------------WLQQYGNARIKLNVDDSF------------SLKDAEFDFLYPWMDTK-   35
Eco20  --------------------------------WLGKYGTARVKLNVDKDF------------SLKDSSLEMLYPIYDTP-   35
Eco25  --------------------------------WLSRFGTARITLGVDEDF------------SLKNSQFDFLHPWYETP-   35
Eco15  --------------------------------WLNNFGTAXISLGVDEDF------------SLKNSQFDFLHPWYDTP-   35
Eco6   --------------------------------WLSRWGTARVSLGVDEDF------------SLKSSSFEFLHPWYETP-   35
Efe2   --------------------------------WLSQWGTARVTLSADEHF------------TLKGSALDLLLPWYDTP-   35
Efe3   --------------------------------WLSQWGTVRVTLGLDEDF------------TLKGSAFDLLLPWHDTP-   35
Pal2   --------------------------------WLSKAGNVKLNIDFDKKF------------SIKNSQFDWLIPWYDQE-   35
Csu1   ---------------------------------------LRFDFFPGDRE-----------YPFERTAIDWLFPFYDSL-   29
Eco3   --------------------------------WLSPWGNASVDVKVDNEG------------HFTGSRGSWFVPLQDND-   35
Cko1   --------------------------------WLSPWGNASVDLRIDNEG------------SFTGSRGGWFIPWQDNT-   35
Sen1   --------------------------------WLSAWGSASVDVKVDNEG------------HFNGSRGSWFIPLQDKQ-   35
Eca1   --------------------------------WLSPWGNASVNVRVDDNG------------TFNGSSGSWFIPWNDNN-   35
Esp2   --------------------------------WLSPWGNASVDLLVDEEG------------KFNGSSGRWFIPWQDNN-   35
Esa3   --------------------------------LLSPWGNATVDLLVDEEG------------NFNGSSGSLFTPWQDNN-   35
Kpn1   --------------------------------WLSPWGNANVDLLVDKEG------------KFTGSKGSWFVPLQDND-   35
Pan1   --------------------------------LLSPYGNAEFNLNVDMAG------------NFDGTGGNLFSPLADEN-   35
Eta2   --------------------------------LLSPYGRASVSLALSDDG------------SFNGTSAQLLTPWQDNY-   35
Spr1   --------------------------------LLSPLGNAKLSLVMSDQG------------DFSGSSGQLFSPLYDVN-   35
Yin1   --------------------------------LLGKFGKAQVNLAVDDKG------------SLSKSSFSLFSPWYEND-   35
Yin2   --------------------------------LLGKFGKAQVNLAVDDNG------------SLSKSAFSLFSPWYEND-   35
Yfr4   --------------------------------LLGKFGQAQVSIAVDDHG------------DFSKSTFSLFTPWYEND-   35
Ymo2   --------------------------------LLGRFGQAQVNLSMDNKG------------NLNRSTASLFTPWYDSE-   35
Eco10  --------------------------------TLSPYGKVRSNLSIGQGG------------DLDGSSIDYFVPWYDNQ-   35
Eal1   --------------------------------ALSPYGKVRSNLSIGQGG------------DLEGSSIDYFIPWYDDQ-   35
Sty4   ---------------------------------MSPYGKIRTSLSVGEGG------------DLDGSSLDYFVPWYDNE-   34
Eco16  --------------------------------VLSPYGSVRTSLSIGEGG------------DLDGSTFDYFVPWYDNQ-   35
Ahy1   SASRYGSEQEVQYWRQQLATQFEEEANAYAASLLGAMGTARTRVTLDDDF------------NMVTAEADLLLPLAEEQ-   67
Sen2   -------------------------------------IIFRGGVNLEDGA------------KYRSSEFDMFIPVQETT-   30
Efe4   -------------------------------------AQFRGGITLEDAS------------KYRSAEADLLIPLYQST-   30
Bpe1   --------------------------------GLPFLRNLQGGLSHDFES------------GRTSLQLNTIDEVYRAG-   35
Bav2   --------------------------------GLRFLRNLQGDLRHDFDN------------GRTSLELRTIDQVYRKG-   35
Bbr1   --------------------------------ELPFLRRLQGGVNYDFSN------------KDLSLDLRTIDEVHRGE-   35
Bpa2   --------------------------------GLPWLRRLEGNVSYDFSG------------RDVAVDVRTIDALHLDQ-   35
Bav1   --------------------------------GLPWLRHLDGGLRYDLDP------------GRLSFSLRTIDDLMVSE-   35
Pma1   ---------------------------------AQTSVNISGGTESDTSF------------SINSLMKLGELAKDDQGD   35
Pma3   ---------------------------------AQTNFNISGGSESDTSF------------SINSLMKLGELAKDEEGD   35
Pma5   ---------------------------------AQTSLNIGGGTESDTSF------------SLNSLMKLGELAKDDEGD   35
Pma4   ---------------------------------AQTTVSINGTSNSETSF------------SLDSLMKLREMATDDEGD   35
Ssp1   ---------------------------------AQTTLGLDAATSSDLTG------------YLDSFMRLKTIGYDNEGD   35
Ssp2   ---------------------------------SQTEFGVGFESDADMTY------------YLNSLISLAQLGSDDNGY   35
Plu2   ----------------------------------TNAPS-----------------------WVTSWDVTVPCPVYVSEN   23
Cla1   ---------SKSKEEIVKNMASSYLNTSANELAKEFIDSLNTSINTDFSFN-------YNERSGFSGNAKALLPIVSEDN   64
Rba1   ----------------------------------LSVGSDTMGLDKNKSD--------------TKTEAMTVYRLKETG-   31
ruler  1.......10........20........30........40........50........60........70........80
```

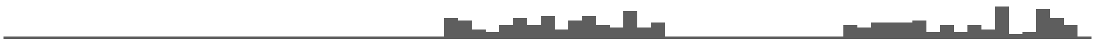

```
                               .  .   *                .         * * . .           * . * *
Mba1   IFNTYFTQGSVFY----EDN-RTTLNLGLGYRK------LSDNKMLLTGINAFYDHEFP---YDHGRTSIGLEARTTVWE   90
Psp2   NENIIFTQASLFL----SDDSRETINLGFGNRK------LINDDTLLVGYNLFYDHELD---YDHQRASIGIEAISSVGS   91
Ybe1   --QMAFTQFGIRR-----IDKRTTANLGIGIR-------HFIDD-WMVGYNLFLDRDIT---RDHTRVGAGAEYARDYLK   98
Ype5   --LLTFTQLGGRY-----IDDRVTLNVGLGQR-------HFFAQ-QMLGYNLFIDHDAS---YSHTRIGVGAEYGRDFIN   98
Eco14  --NVLFTQLGMRD-----NDGRFTTNAGLGHR-------YFTDNGWMLGYNVFYDVDWR---NTNRRYGIGVEAWRDYLK   99
Sen3   -QNLLFTQWGGRR-----DDDRNIINVGMGYR-------YFADR-WMWGINTFYDRQISD--NAHERLGIGGELGWNYFK  100
Ymo1   --RLAFSQFGFRR-----IDQRNIMNIGLGQR-------HYLSD-WMLGYNVFLDQQISG--NAHRRLGLGGELARDYVK   99
Yen2   --KLAFSQFGFRR-----IDQRNIMNIGLGQR-------HYVSD-WMFGYNIFFDQQVSG--NAHRRVGFGGELARDYIK   99
Pas1   --RLFFGQVGARR-----FDGRNIVNLGIGQR-------YFQGD-WALGYNTFYDIQISG--NAHQRLGFGLEYWRDYLY  124
Yfr1   -NNLLFGQLGVRY-----NGERTTNNVGLGVR-------SFTDS-WMFGVNTFYDYDLT---GKNSRLGVGGEAWTDNLK   93
Yfr5   -DNLLFGQLGVRY-----DGYRTTNNAGIGVR-------QFTEN-WMFGVNSFYDYDLT---GKNSRVGVGAEAWTDNLK   93
Yps7   -KSILFTQLGARN-----KDSRNTVNMGAGVR-------TFQGN-WMYGANTFFDNDLT---GKNRRIGVGAEAWTDYLK   98
Yfr2   -KSILFTQLGARN-----KDNRNTVNIGAGVR-------TFQNN-WMYGVNTFFDNDMT---GKNRRVGVGAEAWTDYLK   98
Yfr3   -KSLLFSQFGLRN-----HDSRNTINIGAGVR-------TLQDN-WMYGANVFFDRDIT---GKNNRIGFGAEAWTDYLK   98
Yps4   -SSLLFTQLGVRN-----KDSRNTVNIGAGIR-------QVQGD-WMYGANTFFDNDLT---GKNRRVGVGAEVATDYLK   98
Yps2   -SFLFFSQLGIRN-----KDSRNTLNLGVGIR-------TLENG-WLYGLNTFYDNDLT---GHNHRIGLGAEAWTDYLQ   98
Esa2   -KSVLFTQLGIRA-----PDGRTTGNIGLGVRT------FYVRD-WMFGGNVFFDDDFT---GENRRIGFGAEAWTNYLK   99
Plu1   -HWLVYSQYGYRH-----KDSRDTVNLGIGTR-------LFINN-WMYGANTFYDNDLT---GNNSRFSLGGELWTNYLK   99
Eco1   -KMLAFGQVGARY-----IDSRFTANLGAGQR-------FFLPE-NMLGYNVFIDQDFS---GDNTRLGIGGEYWRDYFK   96
Pru1   -DNLIFSQTSLHR-----KEGRIETNLGLGAR-------WYGE-GQMIGGNTFFDYDIS---RKHSRLGLGVEYRRDFLK   98
Pal3   -NNLLFTQTSLHR-----KEGRIETNLGLGTR-------WYGE-NQMIGGNSFFDYDIS---RKHSRLGLGVEYRRDFLK   98
Eta1   -DSLIFTQGSLHR-----TDDRTQVNLGFGLR-------YFAP-SYMLGGNIFGDYDLS---RAHSRTGIGMEYWRDFLK   98
Sgl1   -DKLVFTQGSLHR-----TDDRTQTNLGMGMR-------WFND-GYMLGGNTFLDYDLS---RDHARMGMGVEYWRDYLK   98
Eco26  -ERLFFSQGGISY-----IDDKFISNIGIGYR-------AFYD-NWMLGGNSFIDYDLR---KEHSRLGLGIEYWQDNLK   98
Pmi1   -EKLIFAQTNYHR-----KDLRSQFNYGIGYR-------YFTE-KFMVGINGFYDHDLT---HHHNRLGIGAEIWRDYFK   98
Efe5   -DYVLFSQTSLHR-----TDDRNQANIGIGVR-------HFTPDNAMLGANIFYDYDLT---RSHSRAGFGVEYWRDYLR   99
Eco20  -TNMLFTQGAIHR-----TDDRTQSNIGFGWR-------HFSGNDWMAGVNTFIDHDLS---RSHTRIGVGAEYWRDYLK   99
Eco25  -DNLFFSQHTLHR-----TDERTQINNGLGWR-------HFTP-TWMSGINFFFDHDLS---RYHSRAGIGAEYWRDYLK   98
Eco15  -DYLLFSQHTLHR-----TDDRTQINTGLGWR-------HFTS-SWMSGINLFFDHDLS---RYHSRAGLGAEYWRDYLK   98
Eco6   -DNLVFSQHTLHR-----TDDRTQTNHGIGWR-------YFTS-SWMSGVNMFIDHDLT---RYHTRTGMGVEYWRDYLK   98
Efe2   -ENIIFTQHSIHR-----TDDRNQLNTGTGWR-------HFMP-DYMTGVNLFFDHDLT---RYHSRMGLGGEYWRDNLK   98
Efe3   -ENLLFTQHSFHR-----TDDRNQLNTGAGWR-------HFAP-DYMAGVNLFFDHDLT---RYHSRMGLGGEYWRDNLK   98
Pal2   -DILLFTQHTLHR-----YDERFHTNNGIGLR-------YFHE-KSTIGMNAFIDHDLS---HAHTRVGLGVEYWQDYLK   98
Csu1   -EFLFFSQLGFRHN----LDRRDIINLGGGGR--------FFRKNWMLGANAFCDCDLT---GTNTRLGLGLESWTNFLQ   93
Eco3   -RYLTWSQLGLTQ-----QDDGLVSNVGVGQRW------ARG--NWLVGYNTFYDNLLD---ENLQRAGFGAEAWGEYLR   98
Cko1   -RYLTWSQLGLTQ-----QDDGLVSNVGIGQRW------ARD--GWLLGYNTFYDNLLD---ENLQRAGFGAEAWGEYLR   98
Sen1   -RYLTWSLLGLTQ-----QTDGLVSNIGVGQRW------VQN--GWLLGYNTFYDNLLD---ENLQRAGVGAEAWGEYLR   98
Eca1   -RYLSWSQLGLTQ-----QTDGLVSNAGVGQRW------VAG--NWLLGYNTFYDNLLD---ENLQRAGLGAEAWGQNLR   98
Esp2   -RYLSWSQLGLTQ-----QTDGLVSNAGIGQRW------VAG--KWLLGYNTFYDNLLD---ENLQRAGLGAEAWGENLR   98
Esa3   -RYLTWSQVGVSQ-----QNQGLVGNAGIGQRW------AAG--HWLLGYNTFYDRLFD---DDTSRAGFGAEAWGDYLR   98
Kpn1   -RYLTWNQYSVTR-----REHDLVGNIGLGQRW------RVG--GWLLGYNSFYDKVLS---ESLARGSVGAEAWGEYLR   98
Pan1   -RYLTFSQLGLHD-----SVEGVVGNAGLGQRW------DAG--NWLLGYNSFIDRSFR---TGLQRASVGTEAWSNNLR   98
Eta2   -QYLTFSQLGIEQ-----SEYGTVGNAGLGQRW------IAG--SWRVGYNAFVDGLLG---SDRQRGSLGAEAWGEYLR   98
Spr1   -GLLTYSQLGLLQ-----QTEGSLGNFGLGQRW------VAG--DWLLGYNTVLDSDFE---RHHNRASLGAEAWGDFLR   98
Yin1   -AMVAFSQVGIHG-----QDGRMIGNLGAGVRF------DQG--DWLLGANTFFDQDIS---RNHSRLGLGLEWWADNLK   98
Yin2   -AMVAFSQVGVHR-----QDNRMIGNLGAGVRF------DQG--DWLFGANTFLDQDIS---RNHSRLGLGLEWWADNLK   98
Yfr4   -AMVAFSQVGIHD-----QDSRTIGNFGAGLRW------DKG--NWLLGYNTFLDQDFS---RNHSRLGLGAELWTESLR   98
Ymo2   -QYLLFSQINIHH-----QDNRKIGNFGLGHRI------ELPSLNGLLGYNVFIDHDFS---RGHNRAGIGAEARADYLK  100
Eco10  -TTVYFSQFSAQR-----KEDRTIGNIGLGVRY------NFD--KYLLGGNIFYDYDFT---RGHRRLGLGAEAWTDYLK   98
Eal1   -STVYFSQFSAQR-----KEERTIGNAGIGVRH------NFD--KWLLGGNIFYDYDFT---RGHRRLGLGTEAWTDYLK   98
Sty4   -KTLLFSQLSAQR-----KEDRTIGNFGLGVRQ------NVG--NWLLGGNAFYDYDFT---RGHRRLGLGTEAWTDYLK   97
Eco16  -ETLVFSQFSAQR-----KDDRTIGNIGVGVRQ------NIG--SWLLGGNLFYDYDFT---RGHRRLGLGSEAWTDYLK   98
Ahy1   -QTLLFTQFGLRRN----GQDRTIANLGVGQRH------FLDR--WMLGYNLFADYDLT---NRHWRAGVGAEAWRDYLK  131
Sen2   -SSLLFGQLGFRDHDSSSFDGRTFANVGMGYRQ------EVNG--WLLGVNTFLDADIR---YSHLRGGIGGEVYKESLA   98
Efe4   -SSILFGQLGLRDHDNNSFNGRFFVNTGIGWRQ------DVGD--WLLGINSFLDADVR---YDHLRGSLGVELFRDSMS   98
Bpe1   -RNTGLLQLGAHN-----QNDRPTANAGAVYR-------REVNDALMVGANGFLDYEFG---KQHLRGSVGLEVIAPEFS   99
Bav2   -ANTGLLQLGGHN-----QNNRPTANLGGVYR-------RDINERLMLGANAFLDYEFA---KQHLRGSLGVEAIAPEFS   99
Bbr1   -RDRVLLQLSGHN-----RNHRPTVNGGVVLR-------HALNQHMAVGANAFLDYEFG---KNHLRGSLGGEVIAPQFT   99
Bpa2   -DRALLLQLGGHN-----QNHRPTVNAGVVAR-------SAAGSSLILGGNAFLDYEVG---KRHLRGSLGAEAVAAQFT   99
Bav1   -RRALMLQAGLHN-----QNQRPTANTGIVLR-------QQASPGLIVGSNAFLDYEFG---KQHVRGSLGLEAIAPHYS   99
Pma1   LKTLAFSQARFATAT---NAEGSTINIGLGIR-------NRPDDISMVGANAFWDYRMTDYSDAHSRLGLGGEYFWKDFE  105
Pma3   LKTLAFSQARFATAT---NADGSTFNLGLGIR-------NRPNDISMVGANAFWDYRMTDYSDAHSRLGLGGEYFWKDFE  105
Pma5   IKTLAFSQARLATAT---NAEGSTTNLGLGIR-------HRPNDVSMLGANAFWDYRMTDYSDAHSRLGLGGEYLWKDFE  105
Pma4   IKTLLFSQAKLSKTS---NSDGTTANLGLGIR-------HRPNNESMMGANAFLDYRMTDYSDSHSRLGLGGEYLKNGWE  105
Ssp1   PMGLMFGQARVTLET---SAQ-PQVNVGLGSR-------FRLGDEAIVGLNGFWDLRTTNYSTAYTRWGIGAEGFWKSFE  104
Ssp2   PLGLLFAQG-SAKGA---YSGSAVTNLGLGLR-------RRLRDNAMLGANAFWDYRFTNYSSSYSRWGAGAELWWDDFK  104
Plu2   QADNIFFEGGFDY-----QDARKTVDGALGYRH------LMSDNKVMLGANVLYSHEFP---RNHQRISYGAEIRTSVFE   89
Cla1   PKISYFLQSGIGEF----ANDRTIGHFGGGIRYYPNATALNNSGNIMLGLNSVYDHDFS---RGHKRMSLGAEAMVDTLA  137
Rba1   -NWFLFNQTSAVN-----FNNRTTINTGFGARH------INDANTVITGYNIFYDYELQ---SKHERVGAGLELLSSIFE   96
ruler  ........90.......100.......110.......120.......130.......140.......150.......160
```

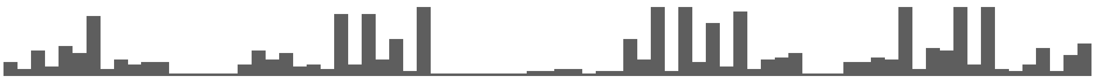

```
              * *                                                         * *
Mba1   INANKYWATTKWKTGK------NGLE---------------------ERALDGYDIEAGVPLPYMNWATVFVKNFQWDSEIS   145
Psp2   LRANQYYGLSGWKSGL------NNIN---------------------EKALNGSDVELGMPLPYLPWTNLYYRSFNWEG-AS   145
Ybe1   LAANGYLRLSDWRDS--PDFS-SYS---------------------ERPATGFDLRAEAYLPSLPQLGGKLMYEQYFGNDV   155
Ype5   LAANGYFGVSGWKNS--PDLD-KYD---------------------EKVANGFDLRSEAYLPTLPQLGGKLIYEQYFGDEV   155
Eco14  LSANGYKRLSDWRQS--PTVT-DYD---------------------ERPADGWDIRAEGWLPAYPQLGGKLVYEQYYGNEV   156
Sen3   LSANGYKRLSGWKDS--SEYE-DYQ---------------------ERVANGYDIRAEGYLPAWPQLGAQLVWEQYYGDDV   157
Ymo1   LSANSYYRLGGWKNS--TRLE-DYD---------------------ERAASGYDIRTEAYLPYYPQLGGKLMYEQYFGNEV   156
Yen2   LSANSYHRLGGWKNS--TRLE-DYD---------------------ERAANGYDIRTEAYLPHYPQLGGKLMYEQYFGDEV   156
Pas1   LSANGYFGLTDWYSS--SALD-GYA---------------------ERAANGYDIRAQGWFPVYPQLSGKLKFEQYFGDDI   181
Yfr1   FSANGYFRLTDWHQSVLADME-DYN---------------------ERPANGFDVRAEAYLPSYPQLGGRLMYEKYFGKGV   152
Yfr5   FSANGYFRLTDWHQSVLSDME-DYN---------------------ERPANGFDVRAAAYLPAYPQLGGSLMYEKYFGKGV   152
Yps7   LSANNYFGITDWHQS--RDFI-DYN---------------------ERPANGYDLRAEAYLPSYPQLGGKAMYEKYRGDDV   155
Yfr2   LSANSYIGTSDWHQS--RDFA-DYN---------------------ERPANGYDVRAEAYLPSHPQLGGKLMYEKYRGEEV   155
Yfr3   LSANSYLRLTDWHQS--RDFA-DYN---------------------ERPANGYDLRVEAYLPAYPQIGTNLKYEQYKGNEV   155
Yps4   FSANTYFGLTGWHQS--RDFS-SYD---------------------ERPADGFDIRTEAYLPAYPQLGGKLMYEKYRGDEV   155
Yps2   LAANGYFRLNGWHSS--RDFS-DYK---------------------ERPATGGDLRANAYLPALPQLGGKLMYEQYTGERV   155
Esa2   LSANTYIGTSQWHNS--GDFD-NYN---------------------EKPADGYDVRAEGYLPSFPQLGAKLMYEQYYGDNV   156
Plu1   MSANAYFRLSDWHNA--RDLV-NYY---------------------ERPANGYDLIADMYLPSMPSLGAKIKYEQYFGDNV   156
Eco1   SSVNGYFRMSGWHES--YNKK-DYD---------------------ERPANGFDIRFNGYLPSYPALGARLMYEQYYGDNV   153
Pru1   LSANSYHRLSGWRSS--RDLA-DHS---------------------ARPSNGWDVRAEGWLPSYPHIGGKLTYEQYYGDSV   155
Pal3   LSANSYHRLSGWRNS--RDLA-DYS---------------------TRPANGWDLRAEGWLPIYPHIGGKLTYEQFYGEEA   155
Eta1   LSANGYLRLSDWNNS--SDFK-DYQ---------------------ERPANGYDIRAQAWLPSLPQLGGKLTYEQYYGRGV   155
Sgl1   IGANNYLRLTNWRDS--KDFA-DYQ---------------------ERPANGWDMSLEGWVPALPQLGGNLKYEQYYGKEV   155
Eco26  LGANSYLRLSNWRNS--SNIV-DYE---------------------ERPANGLDLNIKSWLPSYPQIGGDIKYEKYYGDDV   155
Pmi1   LSSNHYHRLSSWRAS--NNIL-DYS---------------------ERPANGWDIRTEGYFPAYPQLGTKLIFEQYYGKEV   155
Efe5   FGANTYFGLSDWKNS--RDLA-DYL---------------------ERPANGWDVSAEGWLPAYPQLGASVQFEKYYGKNV   156
Eco20  LSANGYIRASGWKKS--PDIE-DYQ---------------------ERPANGWDIRAEGYLPAWPQLGASLMYEQYYGDEV   156
Eco25  LSSNGYLRLTNWRSA--PELDNDYE---------------------ARPANGWDVRAEGWLPAWPHLGGKLVYEQYYGDEV   156
Eco15  LSSNAYIGLTGWRSA--PELDNDFE---------------------ARPANGWDLRAEGWLPAWPQLGGKLVYEQYYGDEV   156
Eco6   LSGNGYLRLSNWRSA--PELDNDYE---------------------ARPANGWDLRAEGWLPAWPQLGGKLVYEQYYGDEV   156
Efe2   LGANGYLRLTGWRDA--PELDYDYE---------------------ARPANGWDVRAEGYLPAYPQLGAKLMYEQYYGDEV   156
Efe3   LGANGYLRLSGWRDA--PELDYDYE---------------------ARPANGWDVRAEGYLPAYPQLGATLMYEQYYGDEV   156
Pal2   LNANSYFGLTSWKSA--SELNHDFN---------------------AKPAHGWDIQVEGWLPNYPHLGGNLRYEQYYGDSV   156
Csu1   FSMNGYLGLSSPRHS---SLQPNYQ---------------------ESPAHGLDFRLHYWLPFFPRLGGKIFYEQYFGKQV   150
Eco3   LSANFYQPFAAWHEQ----TATQE---------------------QRMARGYDLTARMRMPFYQHLNTSVSVEQYFGDRV   153
Cko1   LSANYYQPFASWHEQ----TTALE---------------------QRMARGYDVTAQMRMPFYQHLNTSVSVEQYFGERV   153
Sen1   LSANYYQPFADWQTH----TATLE---------------------QRMARGYDINAQMRLPFYQHINTSVSLEQYFGDSV   153
Eca1   LSANYYQPFAGWRDD----SDVQE---------------------QRMARGYDVTAKAWLPWFHHLNTSVSFEQYFGDSV   153
Esp2   LSANYYQPFASWRDS----SDLQE---------------------QRMARGYDVTAKAWLPYFHHLNTSVSFEQYFGDNV   153
Esa3   LSANYYQPLGGWQHR----AGLLE---------------------QRMARGYDVTAQAYLPFYQHINTSVSFEQYFGDQV   153
Kpn1   LSANYYHPLGDWQLRD---NQTQE---------------------QRMAAGYDVTAQARLPFYQHINTSVSVEQYFGDSV   154
Pan1   FSANYYHPLSGWRNRG---DSQQ----------------------SRMAEGYDITTQSYLPFYRQLGVSFSYQQYLGEKV   153
Eta2   LSANYYHPLSGWRNRS---NSSQ----------------------MRMARGYDITTRGYLPFYHQLGVTLSYEQYLGDRI   153
Spr1   FSANYYYPLSALAQQR---DNAQFL--------------------SRPASGYDITTQGYLPFYRQIGGSLSYEQYWGENV   155
Yin1   LATNYYHPLSGWKDSK---DFDDYL--------------------ERPARGFDVRAQGYLPAYQQLGASVVYEQYYGDEV   155
Yin2   LASNYYHPLSGWKDSK---DFDDYL--------------------ERPARGFDVHAQGYLPAYQQLGASAVYEQYYGDEV   155
Yfr4   LATNFYHPLSGWKDSK---DFDDYL--------------------ERPAKGFDVRLQGYLPAYPHLGASVVYEQYYGDEV   155
Ymo2   FSANYYHPLSHWKDSP---DFDDYL--------------------ERPAKGYDLRSQGYLPAYPQLGVSAVYEHYFGDEV   157
Eco10  FSGNYYHPLSDWKDSE---DFDFYE--------------------ERPARGWDIRAEAWLPAYPQLGGKIVFEQYYGNEV   155
Eal1   FSGNYYHPLSGWKNSE---DFDFYE--------------------ERPARGWDIRAEAWLPAYPQLGGKIVFEQYYGDEV   155
Sty4   FSGNYYHPLSDWKDSE---DFDFYE--------------------ERPARGWDIRMESWLPFYPQLGAKLVYEQYYGDEV   154
Eco16  FSGNYYHPLSDWKDSK---DFDFYE--------------------ERPARGWDVRAEGWLPAWPQFGAKLVYEQYYGDEV   155
Ahy1   LGANFYTPLSSWRDSP---RFEGME--------------------ERAARGMDVRLEAYLPAYPQWSASLTAEQYLGERV   188
Sen2   FSGNYYFPLTGWRTS---AAHEFHD--------------------ERPAYGFDLRTKGTLPDFPWFSGELTYEQYYGDKV   155
Efe4   LAGNWYFPLSDWKAS---KVQPLHD--------------------ERPATGIDVRLKGALPSLPWFGAELAFEQYFGDKV   155
Bpe1   LYGNVYAPLSDWKGAK---RNNRRE--------------------EKPASGMDVGVGYRPAFAPGLSLSATHFRWNGAEV   156
Bav2   FYGNVYAPMSGWTGAK---RDNRRE--------------------ERPASGMDLGMKYSPGFAPGLSLKANYFRWNGAAV   156
Bbr1   LYGNVYAPMSGWKAAK---RAERRE--------------------ERPASGWDVGVRLQPEALPGLAIKGQYFRWSGAAV   156
Bpa2   LYGNVYAPLSGWKAAK---RAERRE--------------------ERPAAGWDVGFTARPEAVQGLALNAQYFRWRGAQV   156
Bav1   LYANYYAPLSGWKGAR---RDSRRE--------------------ERPAAGYDLGGQLS--SDAGLSLQAAYFRWHGAGI   154
Pma1   FRNNWYMAITNEKDVIIKGVDYQ----------------------ERVVPGWDLEVGYRLPNNPELAFYIRGFNWDYKYT   163
Pma3   FRNNWYISITDEKDVTIKGVSYK----------------------ERVVPGWDVELGYRLPNNPELAFFVRGFNWDYKHT   163
Pma5   LRNNWYMSITNEKDVTIKGVSYK----------------------ERVVPGWDVELGYRLPNNPELAFFVRGFNWDYKNT   163
Pma4   LRNNWYMSMTEQKNVTVNGTVYT----------------------ERVVPGWDVEAGYRFPNHPEMAVFVKGFNWDYKNT   163
Ssp1   LRNNWYINGSADKNITINNIDYV----------------------ERVVPGWDVEVGYRIPSYPQLAIFVRGFNWDYQDH   162
Ssp2   LTNNWYIAGTGIKRITTSGRAYTDTTSLAAGTYDETTLLGANTFDERVVPGWDVALNYRLPSYPQLSLGIRGFRWDYMRK   184
Plu2   INSNYYHRLTDWKLTG---VDNNE---------------------EKARGGYDVELALAVPYVPSAHFRVKHFCWNGIAS   145
Cla1   FNANVYQRLSSWIDSY--DFDKDYVQ-------------------ERPANGWDAKIKYAFPSLINVSFFAKMGQWYGNKV   196
Rba1   FRANAYQAVSKTLTYN------GIQ--------------------ETALDGYDAKLTANLPYFYSS-------NLYGKLS   143
ruler  .......170.......180.......190.......200.......210.......220.......230.......240
```

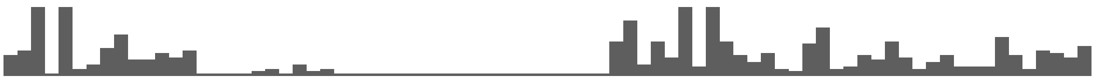

CLUSTAL X (1.81.1-alpha) MULTIPLE SEQUENCE ALIGNMENT


```
Mba1   GSKD---------IKGNDLQLRAYIPGITGLEIQAGRTFFSDSSGTD--ENYINIFYNVTQLFADKPRY-----------N   204
Psp2   GAAD---------LEGDEISLEAKLTNFN-IEIGK-RS--NDGVTED--EEFLKITYTCCNNSN----------------N   195
Ybe1   GLFG--KDN--RQQNPAAITAGINYTPIPLVTVGIDRKQGSAGNGET--LFNLGVNYEVGTPWAK----QISP--DAVNA   223
Ype5   GLFG--VDN--RQKNPLAVTLGVNYTPIPLFTVGVDHKMGRAGMNDT--RFNLGFNYAFGTPLTH----QLDS--DAVAI   223
Eco14  ALFG--ESE--RQKNPHAITAGVTWTPFSLLTAGVDYRRGKNGADDT--RLNLGLTYRIGEPLAH----QLDS--SRVGA   224
Sen3   ALFD--DSEDDRQRNPYAVTAGVNYTPFPLVSIGLNQKMGKGNHNDT--QIDLAVNWMLGSSLKS----QLDS--DAVKA   227
Ymo1   ALFG--LNE--RQKNPSALTASVSYTPFPLVNLALEHTIGNSGKNKT--GVNLAVNYEINTPWQK----QIDP--AAVKA   224
Yen2   ALFG--INE--RQKNPSALTAGVSYTPIPLVSLGLDHTIGNGGKKKT--GVNVAVNYEINTPWQQ----QIDP--AAVQT   224
Pas1   ALLN--HQN--RYKNPYALTMGLEYTPIQLISLGIDRTFSHRGKDDT--KVNLSFNYQLGVPLSQ----QIDP--TVAPV   249
Yfr1   ALNSGSTSPDDLGDSPSAFTVGLNYTPIPLFTVDVAHKKGQNTNNEL--QLGLNFNYRFGVPWVD----QINK--NAVGL   224
Yfr5   ALNSGSTSPDDLGDSPSAVTLGVNYTPIPLITVDVAHKKGQNTSNEL--QVGLNFNYRFGVPWID----QINK--DTVAL   224
Yps7   ALFG----KDNRQKNPHAITAGVNYTPIPLVTIGAEHRAGKGGQNDS--NINFQLNYRLGETWQS----HIDP--SAVAA   223
Yfr2   ALFG----KDNRQKNPHAVTAGVNYTPIPLLTVGAEHRAGKGSKNDS--SINFQFNYRLGESWQS----HINP--SAVAA   223
Yfr3   ALFG----KDDRQKNPYAFTAGINYTPIPLITIGAEQRAGKGGRNDT--NISIQLNYRLGEPWQS----QIDP--SAVAA   223
Yps4   ALFG----KDDRQKDPHAVTLGVNYTPVPLVTIGAEHREGKGNNNNT--SVNVQLNYRMGQPWND----QIDQ--SAVAA   223
Yps2   ALFG----KDNLQRNPYAVTAGINYTPVPLLTVGVDQRMGKSSKHET--QWNLQMNYRLGESFQS----QLSP--SAVAG   223
Esa2   ALFD----KDHLQSNPSAVTVGLNYTPVPLITAGIDYKRGQDSMDEM--KFSLNFHYALDSSWQS----QISP--EQVAT   224
Plu1   ALFG----KNKRQKDPYAATIGVNYTPIPLITAGIDYKLGKEGKSDG--IFSFNVNYRFGVPLSE----QLSP--ENVSS   224
Eco1   ALFN----SDKLQSNPGAATVGVNYTPIPLVTMGIDYRHGTGNENDL--LYSMQFRYQFDKPWSQ----QIEP--QYVNE   221
Pru1   ALFG----TKNLQQNPYSITAGLNYTPIPLVTFNAEHRQGKASKQDS--RFGLQLNYQFGKTWKQ----HLDP--GSVTT   223
Pal3   ALFG----TKNRQNNPYSITAGINYTPIPLITLNGEHRQGKASKQDS--RIGLKLSYQFGKSFKQ----HLDP--DAVGE   223
Eta1   ALFG----KENLQQDPRAITAGVNFTPFPLLTLNAEHRQGASGKNDK--RLGVDFSYQLGMPWQQ----QINP--QAVAT   223
Sgl1   ALFG----KDNRQKDPHAITVGVNYTPFPLLTFSADQRQGKAGQNDT--RLGVQLNIQLGTPWQH----QLDT--SAVGA   223
Eco26  ALFG----ENHRQRNPHSTTLGISYTPFPLMSFKAEHKMGSNNINDS--RIGFEINYQIHTPWES----QINP--VLIPA   223
Pmi1   GLFG----KDKRDKNPHTYTLGINYTPIPLVTLNAERRIGLHDRADN--NLNINLSYRIGESLAS----QLNP--DNVKA   223
Efe5   GLFG----SDNLQENPYAVTGEISYTPVPLVKFSAQHRQGQSNTHDT--RFGVEINYRPGVPLSK----QLDS--DNVAA   224
Eco20  GLFG----KDKRQKDPHAISAEVTYTPVPLLTLSAGHKQGKSGENDT--RFGLEVNYRIGEPLAK----QLDT--DSIRE   224
Eco25  ALFD----KDDRQSNPHAITAGLNYTPFPLMTFSAEQRQGKQGENDT--RFAVDFTWQPGSAMQK----QLDP--NEVDA   224
Eco15  ALFD----KNDRQSNPHAITAGLNYTPFPLLTLSAEQRQGKQGENDT--RFAVDLTWQPSSSMQK----QLNP--DEVAG   224
Eco6   ALFG----KDERQNDPHAITAGLSYTPVPLISFSAEQRQGKQGENDT--RIGMELTLQPGHSLQK----QLDP--AEVAA   224
Efe2   ALFG----RDHRQKDPHAFTAGVSYTPVPLVSLSAEQRQGKGGENDT--RFGLNLSYTPGVSLAR----QLDP--DAVAY   224
Efe3   ALFG----KDKRQQDPHAFTAGLSYTPVPLISLSAEQKQGKGGENDT--RFALNLTYTPGVSLAH----QLDP--DAVAY   224
Pal2   ALFG----KTKRQKNPNAATIGANWTPFPLFTLNASHKLGSEKQVET--QAKLQFTWTFGKNLAH----HLDP--TKVAE   224
Csu1   SLFG----RSDLQHDPFVITTGVEFTPFPLLSMEIDHRMSKASRHYT--LFNIRLNYRFGESLSS----QLNP--FSVAK   218
Eco3   DLFN----SGTGYHNPVALSLGLNYTPVPLVTVTAQHKQGESGENQN--NLGLNLNYRFGVPLKK----QLSA--GEVAE   221
Cko1   DLFD----SGTGYHNPVAVKLGLNYTPVPLVTVTAQHKQGESGVSQN--NLGLNLNYRFGVPLNK----QLSA--SEVAE   221
Sen1   DLFD----SGTGYHNPVALKLGLNYTPVPLLTMTARHKQGESGVSQN--NLGLTLNYRFGVPLKK----QLAA--SEVAQ   221
Eca1   DLFN----SGTGYHNPMAVNLGLDYTPVPLVTISAAHKQGESGVSQN--NLGLKLNYRFGVPLAK----QLSA--GEVAA   221
Esp2   DLFR----SGTGYHNPMAVNLGLDYTPVPLLTFSAAHKQGESGVSQN--NLGMKLNYRFGVPLKK----QLSS--EEVAT   221
Esa3   ELFD----SGTGYHNPVAVKVGLSYTPVPLVTVSAHHRQGESGVSQN--DLGLKLNYRFGVPLTK----QLSP--DEVAA   221
Kpn1   DLFH----SGTGYHNPVAVSVGLNYTPVPLVTVTAKHKQGENGVSQN--NVGLKLNYRFGVPLKQ----QLAA--DEVAI   222
Pan1   DLFN----SGNRYHNPAALSLGLSYTPVPLVTISASHKTSSAGESQD--QLGLKLNYRFGVALSK----QLDA--NNVAE   221
Eta2   DLFN----SGNAVADPSAVSLGINYTPVPLFTLAASRKEGEGGESQN--QFTLKMNYRIGVPLRQ----QLSS--DNVAE   221
Spr1   DLFG----SGKKQNDPRAMQLGVNYTPVPLVTVKALHKMGEGGVSQD--QVELALNYRLGVPLVK----QISP--EYVAQ   223
Yin1   ALFG----KDNLQKDPSAVTVGVDYTPFPLATLKLIHKRGQQGKNNT--EVGLRVSYQIGTPLDK----QLDP--ANVAA   223
Yin2   ALFG----KDNLQKDPHAVTVGVDYTPFPLATLKVSHKMGKDGKNNT--ELGLQVSYQIGTALEK----QLDP--GNVAA   223
Yfr4   ALFG----KEHLQKDPRAVTLGLDYTPFPLTTLKVSHKEGQQGQKEA--QVDLQMNYQIGTALSK----QLDP--DNVAA   223
Ymo2   ALFG----KSHRQKDPRALTLGIDYTPVPLVTLGAKHKYGQQGKKDT--QIDVAFRYQFGSPLSA----QLDP--DNVNQ   225
Eco10  ALFG----TDSLEKDPFAVTLGVKYQPVPLIVVGTDFKAGTGDNTDL--SVNATLNYQFGVPLKD----QLDP--DKVSA   223
Eal1   ALFG----TDNLEKDPHAVTLGLNYQPVPLITVGTDFKTGTGDNTDV--TVNATLNYQFGTPLKD----QLDP--ENVKI   223
Sty4   ALFG----TDNLQKDPHAVTLGLEYTPVPLVTVGSDYKAGTGDNNDF--SVKATVNYQIGTPLAA----QLDP--ENVKI   222
Eco16  ALFG----TDNLEKNPQALTVGLMYNPVPLFTVGSDYKSGTGDNSDL--SVNVSLNYQIGMPLKD----QLDP--DNVKA   223
Ahy1   GLLD----ADQLERDPHAITAGLHYNPFPLLKMDVEQVEASGRQHDT--RFTLGLEWKLGATLWD----MLNP--SSVD-   255
Sen2   DLLG----NGTLSRNPRAAGAALVWNPVPLLEVRAGYRDAGNGGSQA--EGGLRVNYSFGTPLHE----QLDY--RNVGA   223
Efe4   DILG----NDSLTRDPAAFTGAITWKPVPLVEIKAGYKDAGSSGSQT--EAGLNLNYTFGVPLRA----QLDP--SQVRP   223
Bpe1   DYFD----NGRTQAGAKGFKVGVEYRPVSLVSVGLEQTKVIGGGRET--RMQLGLNINLSEPLSK----QLRR-DASGTP   225
Bav2   DYFD----NGRTQDRATGFKYGVQYKPVPLLSLGVEQTRVIGGASQT--SVQLGVALNLSEPLSK----QLRR-GGE-TP   224
Bbr1   DYFD----NGRPQRNARGYKYGVEYRPVPLVAVGLEQTKVLGGARQT--TVQLGVNLSLGEPLSR----QLR---HQSGP   223
Bpa2   DYFD----DGRYRRNPSGFKYGIEYRPVPLIGVGVEQARLQSGERQT--SVQLGVRLNLGEPLSR----QLRRGAQDTAP   226
Bav1   DVFD----SGRAQRNASGFRYGVAYQPGALFNIGLNQTRTLDGQKQT--SVQLNVRINLQEPPSR----QLRR----ESQ   220
Pma1   QDNS--GLEGAVSWQATPHVGLEAYVSNEISAASTTANTDLPGTDEN--FFGLRMNITGNPVKFEK-----------SNY   228
Pma3   QDNS--GLEGAVSWQATPHVGLEAWVSNEISAASTTVNTSLPGTDET--FFGLRMNITGNPIKFEK-----------SNY   228
Pma5   QDNS--GLEGSVRWQATPHLGWEAYVSNEISAASTTVNTSLPGTDEI--FFGLRVNITGNPVTFGK-----------KDY   228
Pma4   QDNS--GIEGSFNWQATPHVNWEAWVSNEISGAKTVKNSDLPKTDET--FFGLRFRWTARPVKFAK-----------KNL   228
Ssp1   SDNS--GIEGSVNWQATPHANLELWVSNEIPAYPTDSNDTIGNQPGP--YIGARVRLTGRPVVFTK-----------SNT   227
Ssp2   SDNS--GVEGSVNWQATPHTNLSAWISSEIPAYPAQSNAQLSSGDDV--YVGVRFNVQLKPVTYKTG----------SNRI   251
Plu2   NDSN---NPIDDLKGNTFSVSGSVYDGLSVEVGYIDYTSGNADYSKAGGERFLKVSYNFDIFGTH-----------VNKAT   212
Cla1   GIFG-ANSVDDLEKNPLIYEGGISYSPFPALTFTLSHSRSAESSKKN-TSINANINIPLDEKAMK---LAFEP--KLAGI   269
Rba1   NWKD----AASYETEHYEAGINAEIAPNLTLRVAAQHKKNSNNTEAV---ASINYSVPLGGANQP---AKVKQDGDWSTK   213
ruler  .......250.......260.......270.......280.......290.......300.......310.......320
```

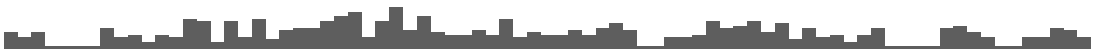

```
Mba1   HQWISKDAYKLESMEDRRYEKVRRTN-------------------------------------NIVKQIKSLGVKVSGI-   246
Psp2   EIGISDTAYNLTSVSDQKFAKVRRQN-------------------------------------LIVKQ-KEMDLTVIGF-   236
Ybe1   RRTLQGSRNDLVERNNQIVLEYKKQD-VINLYV-SNNVSGRAA------ET-----------KQLVVSV-TSKYGLRNIQ   283
Ype5   KRSLMGSRYNLVDRNNQIVMKYRKQN-RVTLEL-PARVSGAAR------QT-----------MPLVANA-TAQQGIDRLE   283
Eco14  QRSLAANRLELVNRNNDVVLEYRKQT-LITLQL-PPDVYGAEL------TT-----------VTLTPQV-NAKYGLSRIE   284
Sen3   RRTLLGSRLDLINRNNNIVLEYRKQD-LISLKV-QNKVTGTES------ET-----------LPVSVNV-KSKYPLDHIS   287
Ymo1   TRTLAGSRMDLVDRNNNIVLEYRKQQ-VVTLNL-PAKVSGKEK------QV-----------LPINYTF-NARHGLDRIE   284
Yen2   TRTLAGRRMDLVDRNNNIVLEYRKQQ-VVTLNL-PEKVSGKEK------QV-----------VPINYTF-NARHGLDRIE   284
Pas1   KRTLADNRYHLVERNNNIVLKHRERA-QLSLYL-PTGLSGFGG------ER-----------KLINFSF-NGKYRLKHIQ   309
Yfr1   MRSLMGSRYDIVDRNYNIVMQYEKQD-LIKLTL-PETLAAYAI------TN-----------LSLTGNI-TAKYGAERME   284
Yfr5   MRSLAGSRYDLVDRNYNIVMQYEKQN-LINLTL-PETISAHAL------EN-----------VTLTGTV-ASKYGTERIE   284
Yps7   SRTLAGSRYDLVERNNHIVLDYQKQN-LVRLSL-PDSLAGDPF------SQ-----------LSVTAQV-TATHGLERID   283
Yfr2   TRTLAGSRYDLVERNNNIVLDYQKQE-LIRLSL-PERVEGKAG------DI-----------ATVNAQV-TSKYGLERID   283
Yfr3   SRTLAGSRYDLVERNNNIVLEYQKQD-LIQLVL-PNQMTGSAF------EI-----------IKVEAQV-TAKYGLKRID   283
Yps4   NRTLAGSRYDLVERNNNIVLDYKKQE-LIHLVL-PDRISGSGG------GA-----------ITLTAQV-RAKYGFSRIE   283
Yps2   TRLLAESRYNLVDRNNNIVLEYQKQQ-VVKLTLSPATISGLPG------QV-----------YQVNAQV-QGASAVREIV   284
Esa2   RRSLAGSRYDLVDRNNEIILQYKKK-------------------------------------------------------   249
Plu1   LRSLAGSRYDLVERNNNIILNYLKKQ-QHFRLL-VPVIEISSY------GG-----------EVKPIQI-QSDTPFKNVT   284
Eco1   LRTLSGSRYDLVQRNNNIILEYKKQD-ILSLNI-PHDINGTER------ST-----------QKIQLIV-KSKYGLDRIV   281
Pru1   FRSLMGNRYDFVSRNNHIVLEYKKND-VIQLNI-ANSITGYAG------EK-----------IPLSFTV-ASKYGLSHLK   283
Pal3   FRSLMGNRYDFVSRNNHILLDYKKND-TIYLNM-NASITGISG------EK-----------IPLKYTI-NSQYGFNRIN   283
Eta1   MRSLAGSRYDLVERNNHILLQYRKKE-VIRLHT-VGRVTGYAG------ER-----------KSLGVSV-NSSYGLERID   283
Sgl1   MRTLAGSRYDLVDRNNNIVLEYRKKE-VIHLYT-ADHLAGYAG------EQ-----------KSLNVSI-NTKYGLERID   283
Eco26  MRKLAGQRYDLVERNNNIILDYRKKE-IIKIDG-VDVISGFSG------EK-----------KRLDIRV-NSKYPVDRID   283
Pmi1   IRTLAGSRYDFVNRNNDMILEYKKET-LVFLSM-VDSINGYAK------EE-----------RDLQVQV-KTKYPLANIE   283
Efe5   MREVQNRRYDFVERNNNIVLEYKKKS-TLRISL-PDAIEGESG------AT-----------IPVTLNVSHASNGIQSVQ   285
Eco20  RRVLAGSRYDLVERNNNIVLEYRKSE-VIRIAL-PERIEGKGG------QT-----------LSLGLVVSKATHGLKNVQ   285
Eco25  RRSLAGSRFDLVDRNNNIVLEYRKKE-LVRLTL-TDPVTGKSG------EV-----------KSLVSSL-QTKYALKGYN   284
Eco15  RRSLAGSRYDLIDRNNNIVLEYRKKE-LIRLSL-LDPVKGKSG------EI-----------KPLVSSL-QTKYALKGYN   284
Eco6   RRSLVGSRYDLVDRNNNIVLEYRKKE-LVRLTL-TDPLKGKPG------EV-----------KSLVSSL-QTKYALKGYD   284
Efe2   RRSLSGSRHDLVERNNNIVLEYRKKE-LVKLQL-NDPVTGKGG------EQ-----------KPLVASL-QSKYALKTLQ   284
Efe3   RRSLAGSRHDLVERNNNIVLEYRKKE-LVKLQL-HDPVTGKGG------EQ-----------KPLVASL-QSKYALKTLR   284
Pal2   TRRLSGNRYDFVERNNNIILNYQKKT-VLHLSL-PSKIQGITG------QS-----------VPLVKSF-TSKYPLKHIE   284
Csu1   MRFLNENRYNFVSRNHVIVFNYSEYT-YDIFVS-PASISAIEF------AS-----------STVSLKCPNTKT-LAHFE   278
Eco3   SQSLRGSRYDNPQRNNLPTLEYRQRK-TLTVFLATPPWDLKPG------ET-----------VPLKLQI-RSRYGIRQLI   282
Cko1   SQSLRGSRYDNPQRNNLPTMEYRQRK-TLTVFLATPPWDLQPG------ET-----------VPLKVQI-RSRHGVRHVT   282
Sen1   SQSLRGSRYDMPQRNALPTMEYRQRK-TLTVFLATPPWDLTPG------ET-----------VTLKLQV-RSVHGIRHLS   282
Eca1   TRSLRGSRYDPAERNSLPVMEFRQRK-TLSVYLATPPWDLKGG------ET-----------VMLKLQI-RSTHGIRQLH   282
Esp2   TRSLRGSRYDPVDRTSLPVMEFRQRK-TLSVYLATPPWDLTPG------ET-----------VVLKMQV-RSTHGVRQLH   282
Esa3   SRSLRGSRYDRVERTNVPVMEFRQRK-TLSVFLATPPWDLSAG------ET-----------VALKLQV-RSRHGIRQLS   282
Kpn1   SNSLRGSRFDSPERDNLPVVEYRQRK-NLTVYLATPPWDLQSG------ET-----------VQLKLQI-HSLHGIKALH   283
Pan1   ARSLRGSRYDTVTRSQNPVMAYRQRK-TLSVFLATPPWQLNPG------ES-----------VPLKLQL-RASHSIKALS   282
Eta2   ARSLSGSRYDSVNRDNSPVMAFRQLK-TLSVFLATPPWQLQPG------ET-----------LRLKLQI-ANKNTIKAVS   282
Spr1   AKSLRGSRYDNIERKNVPVMAFKQRK-TLQVFLATPPWRLQPG------ET-----------LPLVLEI-KTTNKITRVS   284
Yin1   MRSLKGSRYDLVDRNNDIVLEYKEKA-SLALDLAAVPMTLLEG------DI-----------YLMQPLV-RSKYKIVGVT   284
Yin2   MRSLKGSRYDLVDRNYDIVLEYKEKA-VLSLDLAAVPMTLLEG------DV-----------YMMQPLV-RSKYRITSVS   284
Yfr4   MRSLMGSRYDRVDRNYDIVLEYKEKAGLLMLDLAAVPATLLEG------DV-----------YLMQPLV-NGKYPIASVS   285
Ymo2   LRSLKGSRYDLVDRNNDIVLEYKEKQ-VLFADLAAVPDSLMEG------ES-----------YILRPLV-KSKYPIIDLI   286
Eco10  AHSLMGSRHDFVERNNFIVLEYKEKD-PLYVTLWLKADVTNEHPECVIKDTPEEAIGLEKCKWTINALI-NHHYKIVAAS   301
Eal1   AHSLMGSRHDFVERNNFIVLEYREKD-HLDVTLWLKADATNEHPECVIKDTPEEAVGLEKCKWTVNALI-NHHYKIVAAS   301
Sty4   QHSLMGSRTDFVDRNNFIILEYREKD-PLDVTLWLKADATNEHPECVIEDTPEAAVGLEKCKWTVNALI-NHHYKIISAS   300
Eco16  KHSLMGSRHDFVERNNFIVLEYREQD-PLDVTLWLKADTANEHPECIVKDTPELAVGLEKCKWTINALI-NNHYKIVAAS   301
Ahy1   -KSLAGMRHDLIERNNDMVLEYRDKV-LLKASL-NDQYSAVEG------QA-----------LTLTLNI-QHSRQIASIQ   314
Sen2   PSNTTN-RRAFVDRNYDIVMAYREQA--SKIRITAMPVSGLSG------------------TLVTLMATIDSRYPIEKVE   282
Efe4   ASNTTN-RTAFVDRNYNIVMEYREQA--SRIRVYASPVNGQSG------------------DTVTLSATINSRYPVERIE   282
Bpe1   AFSPDARRHALVERENRIVLNTRRKEIILPLVVSKVSTLQADG--------------------RVTVIGATQPFATVTVR   285
Bav2   VFNLDAHRNALVERENRIVLNTRQKLIILPLTVTTVLTDSVSG--------------------RITLVGQTQAQATVNWT   284
Bbr1   AFDLQARMGEFVERENRIVLQTRRKHVVLPLTIARVDTDPATG--------------------RITVTGVTEPGAQVSLG   283
Bpa2   PFDRGARLQDFVRRENRIVLDTRRKKIVLALRIAEVRTDPATG--------------------RITVYGVTEPLADVQLW   286
Bav1   PFNLTSRRHQWVERESRIVLNTRRKAITLPLSIAQLRGDPTNG--------------------AIEVSGQTEAGARIMLT   280
Pma1   KKNMVTQMTQPVKRKYDVLLERSAG----------GVFQNR-------------------------------AKGV----   263
Pma3   KKNIITQMTQPVKRVNDVLLERAAINSS-----GAATFTVRVS-----------------------------AQGT----   270
Pma5   KKNMITQMTQPVKREYNVLLERYGAN---------STFTNR-------------------------------AKGS----   264
Pma4   KQNLVTQMTQPVRRRYEVLLERSTGG-----------FQNR-------------------------------ARGI----   262
Ssp1   KQNLLTQMTQPVRRRYEVLLERVKEPTK-----GENGSITS-I-----------------------------AQGI----   268
Ssp2   RENLLTQMRQPVQRRNDVLLERWKPKQKKTIGGVNGSFINQ-------------------------------AAGI----   296
Plu2   KPRFSNTPYEFERMDDRRFEKIRFEQKVISAHNNEQPPA-----------------------------------------   251
Cla1   SNTIEGTRTQFIDRDYSMVLEYRATPNKYHISYCGDLGNDKHCILLKN-----------GFDEVVKNTPMRVTPTHSCVV   338
Rba1   FEPIREKLYRPVQRENRIMKKAIKLG---------VTVSGY---------------------------------------   245
ruler  .......330.......340.......350.......360.......370.......380.......390.......400
```

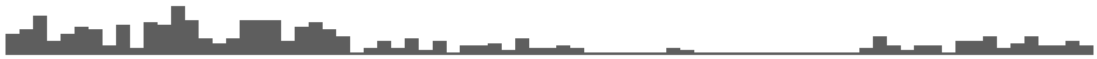

CLUSTAL X (1.81.1-alpha) MULTIPLE SEQUENCE ALIGNMENT

File: /Users/saierlab/Desktop/69BBcut.ps   Date: Tue May 12 18:56:54 2009


```
  Mba1 ------------------------------------------------   246
  Psp2 ------------------------------------------------   236
  Ybe1 FDQGA----------------LAAAGGKIIP----QGPSQFALQLPPQ   311
  Ype5 WEASA----------------LTLAGGKITG----SG-NNWQITLPSY   310
 Eco14 LDDAE----------------LRQAGGKIIS----NTGNQITLQLPAW   312
  Sen3 WEDDN----------------LVKNGGKISE----NNG-SWSVTLPHY   314
  Ymo1 WDAAD----------------VINAGGNISD----QGNLAYHITFPPY   312
  Yen2 WDAAD----------------VIKAGGQVIN----QGNLAYYIAMPPY   312
  Pas1 WNDGA----------------LRARGGRIIA----LSNNSYVVQFPNY   337
  Yfr1 WSAPA----------------LMAAGGSIIP----LTMESASVTLPPY   312
  Yfr5 WSAPV----------------LVTTGGALTP----LAMESAVVTLPAY   312
  Yps7 WQSAE----------------LMAAGGVLKQ----TSKNGLEITLPEY   311
  Yfr2 WDSAA----------------LIAAGGTLSK----GSSNSISITLPPY   311
  Yfr3 WDTAV----------------IVAAGGVVTQ----TSSQNISIKLPPY   311
  Yps4 WDATP----------------LENAGGSTSP----LTQSSLSVTLPFY   311
  Yps2 WSDAE----------------LIAAGGTLTP----LSTTQFNLVLPPY   312
  Esa2 ------------------------------------------------   249
  Plu1 WDIPE----------------LFQKNGGMIN--I-ESTHGYTIQLPEY   313
  Eco1 WDDSS----------------LRSQGGQIQHSGS-QSAQDYQAILPAY   312
  Pru1 WNAET----------------LVAAGGHIVQE-----NGKYSLVLPAY   310
  Pal3 WHADS----------------LVAAGGQVIDEK----NGAYSIILPTY   311
  Eta1 WSASS----------------LLAAGGKLVREN----EGSWSVILPEH   311
  Sgl1 WSAPE----------------LLAAGGKIVQES----IDNYSIVLPDY   311
 Eco26 WLANT----------------FIANGGKIINEG----LHNYSIILPDY   311
  Pmi1 WSASK----------------LNAQGGQIKHHG----GTHYTVILPQY   311
  Efe5 WSDSA----------------FAAAGGKITGS-----GTSWQFTLPAY   312
 Eco20 WEAPS----------------LLAEGGKITGQ-----GSQWQVTLPAY   312
 Eco25 VEATA----------------LEAAGGKVVTT-----GKDILVTLPAY   311
 Eco15 IEAAA----------------LEAAGGKVSTS-----GKDITVTLPGY   311
  Eco6 IEAAS----------------LQSAGGKVAVS-----GKDIQVTIPPY   311
  Efe2 TDAAT----------------LTAAGGVISTA-----DNQVTVTLPAY   311
  Efe3 AEAAE----------------LQSAGGVVNTE-----ANQVTVTLPEY   311
  Pal2 WQAPE----------------FLAVGGSISSD-----DQTATLTLPSY   311
  Csu1 WEAQN----------------FIEAGGEITLVSAGKSNAEYTVKLPPH   310
  Eco3 WQGDT------------QILSLTPGAQANSAEG-------WTLIMPDW   311
  Cko1 WQGDT------------QALSLTAGAKADSAEG-------WTIIMPAW   311
  Sen1 WQGDT------------QALSLTAGTDTRNTEG-------WTIIMPAW   311
  Eca1 WQGDT------------QALSLTSPANSTSSDG-------WSIIIPAW   311
  Esp2 WQGDT------------HALSLTSPANANSDEG-------WSVIMPAW   311
  Esa3 WQGDT------------QALSLTPPLDSTSADG-------WTVIMPAW   311
  Kpn1 WQGDT------------QALSLTPPVDASSPDG-------WSIIMPVW   312
  Pan1 WQGDT------------QALSLTSPPNNNAIDG-------WSVIVPPW   311
  Eta2 WQGDT------------QALSLTPPPDNSDPQG-------WSIIIPAW   311
  Spr1 WQGDT------------QALSLTPSQNSNDPHG-------WSLIVPQW   313
  Yin1 WNGDT------------VPLSLLATAGATNPQG-------WQITLPAW   313
  Yin2 WHGDA------------VPLLLVPTAGANNPQG-------WQITLPAW   313
  Yfr4 WQGDT------------VPLALLATAGVDNHQG-------WQITLPAW   314
  Ymo2 WLGDL------------LPLQLLATAGSHNPQG-------WQITLPAW   315
 Eco10 WQAKNNAASWQAKNNAARTLVMPVIKENTLTEGN---NNHWNLVLPAW   346
  Eal1 WQAKN---------NAARTLVMPVIKENTLTEGN---NNRWNIVLPAW   337
  Sty4 WQAKN---------NAARTLVMPVVKADALTEGN---NNSWNLVLPAW   336
 Eco16 WQAKN---------NANRTLVMPVVKANALTEGN---NNRWNLVLPAW   337
  Ahy1 WLGDVLG--------------LSGLSPADTAGQ-----DKRALTLPSL   343
  Sen2 WSGDA-----------------ELIAGLQLQGS-----LGSGLILPQL   308
  Efe4 WTGDA-----------------ELIGGLQQQGN-----VNSGLRLPDL   308
  Bpe1 MPDGM-------------TGTATADASGRFAYTSAGDQPSGVLALRAR   320
  Bav2 LPDGS-------------SGQSRADASGVYRIESRKDQPSGPIRLKAV   319
  Bbr1 LPNGE-------------VVVAQADGSGTYRATSARDMVGGPVRARAT   318
  Bpa2 LPDGT-------------ATSVRANAAGGFEASSAGDMTSGLIRARAT   321
  Bav1 FPDGS-------------GNWVRADASGRYTARSGPDMPSGTVRAQAR   315
  Pma1 ------------------------------------------------   263
  Pma3 ------------------------------------------------   270
  Pma5 ------------------------------------------------   264
  Pma4 ------------------------------------------------   262
  Ssp1 ------------------------------------------------   268
  Ssp2 ------------------------------------------------   296
  Plu2 ------------------------------------------------   251
  Cla1 FEKGFD--------------YITDNSGKVYVQIIRSCTPKTDLNAVAG   372
  Rba1 ------------------------------------------------   245
 ruler .......410.......420.......430.......440........
```

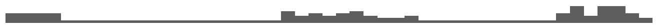

Supplement: Figure S2 — Multiple alignment of N-terminal β-barrel domains. (0.37 MB PDF) [file pone.0014403.s002.pdf]
