# Supplementary material for: The Bacterial Intimins and Invasins: A Large and Novel Family of Secreted Proteins
Source: PLoS One. 2010 Dec 22;5(12):e14403. doi: 10.1371/journal.pone.0014403 (PMC3008723; doi:10.1371/journal.pone.0014403)
Supplement: Figure S3 — Multiple alignment of N-terminal β-barrel domains. Predicted transmembrane β-strands are shaded yellow with predicted β-strands numbered 1 to 16 above the alignment. Fully conserved residues are colored red. Predicted α-helices are shaded blue located between β-strands 12 and 13. (0.14 MB PDF) [file pone.0014403.s003.pdf]

1

```
Mba1      ---------------------------------FGAQGGSKP------------------
Psp2      ---------------------------------LSTGVTNEV------------------
Ybe1      --------------------------------WLNNFGTARLQANVDDRG----------
Ype5      --------------------------------WFNQFGSAKVQLNLDEKL----------
Eco14     --------------------------------WLNQFGTAKTQLSVVSDF----------
Sen3      --------------------------------WLQQFGTAKVNLGLDKDL----------
Ymo1      --------------------------------WLGHIGKAQVKLQVDDKN----------
Yen2      --------------------------------WLGHIGQAQVKLQTDDKN----------
Pas1      -------------------NDDVMQGSDISKSGIADMGFAALQPETEKSAGEVRANLPLS
Yfr1      --------------------------------WLGQYGNARVQLNSNS------------
Yfr5      --------------------------------WLSQFGHARVQLNSSG------------
Yps7      --------------------------------WLSQFGTARVQLNINDDF----------
Yfr2      --------------------------------WLSQFGTARVQMNVNDDF----------
Yfr3      --------------------------------WLSQFGTARVQLNLDNNL----------
Yps4      --------------------------------WLNQFGTARVQLNVDSDF----------
Yps2      --------------------------------WLNRFGTAQVNLNFDKNF----------
Esa2      --------------------------------WLSHFGTAQVTLDVDDNG----------
Plu1      --------------------------------WLSQFGTAKINLNVDHRG----------
Eco1      --------------------------------WLQHYGTAEVNLQSGNN-----------
Pru1      --------------------------------WFNRFGSSQIHLEADKKF----------
Pal3      --------------------------------WFNHFGSSQIKLEADKKF----------
Eta1      --------------------------------WLNQFGTARVQLDADEHF----------
Sgl1      --------------------------------WLSQFGTARLQLDVDNKF----------
Eco26     --------------------------------WFENFGTAHIQLQVDKNF----------
Pmi1      --------------------------------WLNQFGHARVSLSADKNL----------
```

```
Efe5     --------------------------------WLQQYGNARIKLNVDDSF----------
Eco20    --------------------------------WLGKYGTARVKLNVDKDF----------
Eco25    --------------------------------WLSRFGTARITLGVDEDF----------
Eco15    --------------------------------WLNNFGTAXISLGVDEDF----------
Eco6     --------------------------------WLSRWGTARVSLGVDEDF----------
Efe2     --------------------------------WLSQWGTARVTLSADEHF----------
Efe3     --------------------------------WLSQWGTVRVTLGLDEDF----------
Pal2     --------------------------------WLSKAGNVKLNIDFDKKF----------
Csu1     ---------------------------------------LRFDFFPGDRE----------
Eco3     --------------------------------WLSPWGNASVDVKVDNEG----------
Cko1     --------------------------------WLSPWGNASVDLRIDNEG----------
Sen1     --------------------------------WLSAWGSASVDVKVDNEG----------
Eca1     --------------------------------WLSPWGNASVNVRVDDNG----------
Esp2     --------------------------------WLSPWGNASVDLLVDEEG----------
Esa3     --------------------------------LLSPWGNATVDLLVDEEG----------
Kpn1     --------------------------------WLSPWGNANVDLLVDKEG----------
Pan1     --------------------------------LLSPYGNAEFNLNVDMAG----------
Eta2     --------------------------------LLSPYGRASVSLALSDDG----------
Spr1     --------------------------------LLSPLGNAKLSLVMSDQG----------
Yin1     --------------------------------LLGKFGKAQVNLAVDDKG----------
Yin2     --------------------------------LLGKFGKAQVNLAVDDNG----------
Yfr4     --------------------------------LLGKFGQAQVSIAVDDHG----------
Ymo2     --------------------------------LLGRFGQAQVNLSMDNKG----------
Eco10    --------------------------------TLSPYGKVRSNLSIGQGG----------
Eal1     --------------------------------ALSPYGKVRSNLSIGQGG----------
Sty4     ---------------------------------MSPYGKIRTSLSVGEGG----------
Eco16    --------------------------------VLSPYGSVRTSLSIGEGG----------
```

```
Ahy1      SASRYGSEQEVQYWRQQLATQFEEEANAYAASLLGAMGTARTRVTLDDDF----------
Sen2      -------------------------------------IIFRGGVNLEDGA----------
Efe4      -------------------------------------AQFRGGITLEDAS----------
Bpe1      --------------------------------GLPFLRNLQGGLSHDFES----------
Bav2      --------------------------------GLRFLRNLQGDLRHDFDN----------
Bbr1      --------------------------------ELPFLRRLQGGVNYDFSN----------
Bpa2      --------------------------------GLPWLRRLEGNVSYDFSG----------
Bav1      --------------------------------GLPWLRHLDGGLRYDLDP----------
Pma1      ---------------------------------AQTSVNISGGTESDTSF----------
Pma3      ---------------------------------AQTNFNISGGSESDTSF----------
Pma5      ---------------------------------AQTSLNIGGGTESDTSF----------
Pma4      ---------------------------------AQTTVSINGTSNSETSF----------
Ssp1      ---------------------------------AQTTLGLDAATSSDLTG----------
Ssp2      ---------------------------------SQTEFGVGFESDADMTY----------
Plu2      ----------------------------------TNAPS---------------------
Cla1      ---------SKSKEEIVKNMASSYLNTSANELAKEFIDSLNTSINTDFSFN-------YN
Rba1      ----------------------------------LSVGSDTMGLDKNKSD----------
```

2 **3** **4**

```
Mba1      -----SGGLLVVAPLSDPDDIFNTYFTQGSVFY----EDN-RTTLNLGLGYRK------L
```

Psp2	-----TGSVLVVKPISDPSDNENIIFTQASLFL----SDDSRETINLGFGNRK------L
Ybe1	--DLDGSQFDMLMPFYDTPS--QMAFTQFGIRR-----IDKRTTANLGIGIR-------H
Ype5	--SLKGSQLDVLLPLTDSPD--LLTFTQLGGRY-----IDDRVTLNVGLGQR-------H
Eco14	--SLKGSSLDVLLPFYNTPK--NVLFTQLGMRD-----NDGRFTTNAGLGHR-------Y
Sen3	--SLDNASLDLLLPLYDDKK-QNLLFTQWGGRR-----DDDRNIINVGMGYR-------Y
Ymo1	--DFSGSELHLFVPLYNQPE--RLAFSQFGFRR-----IDQRNIMNIGLGQR-------H
Yen2	--DFSGSEIDLFIPLYDQPE--KLAFSQFGFRR-----IDQRNIMNIGLGQR-------H
Pas1	DGKLTSGSIDLFYPLYDGDS--RLFFGQVGARR-----FDGRNIVNLGIGQR-------Y
Yfr1	-----IGNADVLIPLTETQ--NNLLFGQLGVRY-----NGERTTNNVGLGVR-------S
Yfr5	-----TGNADILLPLVESQ--DNLLFGQLGVRY-----DGYRTTNNAGIGVR-------Q
Yps7	--HLDGSAADVLIPLYDNE--KSILFTQLGARN-----KDSRNTVNMGAGVR-------T
Yfr2	--KLDGSAVDVLVPIYDNQ--KSILFTQLGARN-----KDNRNTVNIGAGVR-------T
Yfr3	--SLKGSAFDMLLPLYDDQ--KSLLFSQFGLRN-----HDSRNTINIGAGVR-------T
Yps4	--KLDNSALDLLVPLKDSE--SSLLFTQLGVRN-----KDSRNTVNIGAGIR-------Q
Yps2	--SLKESSLDWLAPWYDSA--SFLFFSQLGIRN-----KDSRNTLNLGVGIR-------T
Esa2	--NWDNSAFDFLAPLYDNK--KSVLFTQLGIRA-----PDGRTTGNIGLGVRT------F
Plu1	--RLDESSVDLLVPFYDDKD-HWLVYSQYGYRH-----KDSRDTVNLGIGTR-------L
Eco1	---FDGSSLDFLLPFYDSE--KMLAFGQVGARY-----IDSRFTANLGAGQR-------F
Pru1	--SLKNSQIDLLMPWYETE--DNLIFSQTSLHR-----KEGRIETNLGLGAR-------W
Pal3	--SLKNSQIDLLLPLYETE--NNLLFTQTSLHR-----KEGRIETNLGLGTR-------W
Eta1	--SFKNSQFDLLAPLYEQK--DSLIFTQGSLHR-----TDDRTQVNLGFGLR-------Y
Sgl1	--SLKNSQLDLLIPLYEQP--DKLVFTQGSLHR-----TDDRTQTNLGMGMR-------W
Eco26	--SLKNSQLELLFPVFEDD--ERLFFSQGGISY-----IDDKFISNIGIGYR-------A
Pmi1	--TLKNSSAELLIPLYEQK--EKLIFAQTNYHR-----KDLRSQFNYGIGYR-------Y
Efe5	--SLKDAEFDFLYPWMDTK--DYVLFSQTSLHR-----TDDRNQANIGIGVR-------H
Eco20	--SLKDSSLEMLYPIYDTP--TNMLFTQGAIHR-----TDDRTQSNIGFGWR-------H
Eco25	--SLKNSQFDFLHPWYETP--DNLFFSQHTLHR-----TDERTQINNGLGWR-------H

Eco15	--SLKNSQFDFLHPWYDTP--DYLLFSQHTLHR-----TDDRTQINTGLGWR-------H
Eco6	--SLKSSSFEFLHPWYETP--DNLVFSQHTLHR-----TDDRTQTNHGIGWR-------Y
Efe2	--TLKGSALDLLLPWYDTP--ENIIFTQHSIHR-----TDDRNQLNTGTGWR-------H
Efe3	--TLKGSAFDLLLPWHDTP--ENLLFTQHSFHR-----TDDRNQLNTGAGWR-------H
Pal2	--SIKNSQFDWLIPWYDQE--DILLFTQHTLHR-----YDERFHTNNGIGLR-------Y
Csu1	-YPFERTAIDWLFPFYDSL--EFLFFSQLGFRHN----LDRRDIINLGGGGR--------
Eco3	--HFTGSRGSWFVPLQDND--RYLTWSQLGLTQ-----QDDGLVSNVGVGQRW------A
Cko1	--SFTGSRGGWFIPWQDNT--RYLTWSQLGLTQ-----QDDGLVSNVGIGQRW------A
Sen1	--HFNGSRGSWFIPLQDKQ--RYLTWSLLGLTQ-----QTDGLVSNIGVGQRW------V
Eca1	--TFNGSSGSWFIPWNDNN--RYLSWSQLGLTQ-----QTDGLVSNAGVGQRW------V
Esp2	--KFNGSSGRWFIPWQDNN--RYLSWSQLGLTQ-----QTDGLVSNAGIGQRW------V
Esa3	--NFNGSSGSLFTPWQDNN--RYLTWSQVGVSQ-----QNQGLVGNAGIGQRW------A
Kpn1	--KFTGSKGSWFVPLQDND--RYLTWNQYSVTR-----REHDLVGNIGLGQRW------R
Pan1	--NFDGTGGNLFSPLADEN--RYLTFSQLGLHD-----SVEGVVGNAGLGQRW------D
Eta2	--SFNGTSAQLLTPWQDNY--QYLTFSQLGIEQ-----SEYGTVGNAGLGQRW------I
Spr1	--DFSGSSGQLFSPLYDVN--GLLTYSQLGLLQ-----QTEGSLGNFGLGQRW------V
Yin1	--SLSKSSFSLFSPWYEND--AMVAFSQVGIHG-----QDGRMIGNLGAGVRF------D
Yin2	--SLSKSAFSLFSPWYEND--AMVAFSQVGVHR-----QDNRMIGNLGAGVRF------D
Yfr4	--DFSKSTFSLFTPWYEND--AMVAFSQVGIHD-----QDSRTIGNFGAGLRW------D
Ymo2	--NLNRSTASLFTPWYDSE--QYLLFSQINIHH-----QDNRKIGNFGLGHRI------E
Eco10	--DLDGSSIDYFVPWYDNQ--TTVYFSQFSAQR-----KEDRTIGNIGLGVRY------N
Eal1	--DLEGSSIDYFIPWYDDQ--STVYFSQFSAQR-----KEERTIGNAGIGVRH------N
Sty4	--DLDGSSLDYFVPWYDNE--KTLLFSQLSAQR-----KEDRTIGNFGLGVRQ------N
Eco16	--DLDGSTFDYFVPWYDNQ--ETLVFSQFSAQR-----KDDRTIGNIGVGVRQ------N
Ahy1	--NMVTAEADLLLPLAEEQ--QTLLFTQFGLRRN----GQDRTIANLGVGQRH------F
Sen2	--KYRSSEFDMFIPVQETT--SSLLFGQLGFRDHDSSSFDGRTFANVGMGYRQ------E
Efe4	--KYRSAEADLLIPLYQST--SSILFGQLGLRDHDNNSFNGRFFVNTGIGWRQ------D

```
Bpe1    --GRTSLQLNTIDEVYRAG--RNTGLLQLGAHN-----QNDRPTANAGAVYR-------R
Bav2    --GRTSLELRTIDQVYRKG--ANTGLLQLGGHN-----QNNRPTANLGGVYR-------R
Bbr1    --KDLSLDLRTIDEVHRGE--RDRVLLQLSGHN-----RNHRPTVNGGVVLR-------H
Bpa2    --RDVAVDVRTIDALHLDQ--DRALLLQLGGHN-----QNHRPTVNAGVVAR-------S
Bav1    --GRLSFSLRTIDDLMVSE--RRALMLQAGLHN-----QNQRPTANTGIVLR-------Q
Pma1    --SINSLMKLGELAKDDQGDLKTLAFSQARFATAT---NAEGSTINIGLGIR-------N
Pma3    --SINSLMKLGELAKDEEGDLKTLAFSQARFATAT---NADGSTFNLGLGIR-------N
Pma5    --SLNSLMKLGELAKDDEGDIKTLAFSQARLATAT---NAEGSTTNLGLGIR-------H
Pma4    --SLDSLMKLREMATDDEGDIKTLLFSQAKLSKTS---NSDGTTANLGLGIR-------H
Ssp1    --YLDSFMRLKTIGYDNEGDPMGLMFGQARVTLET---SAQ-PQVNVGLGSR-------F
Ssp2    --YLNSLISLAQLGSDDNGYPLGLLFAQG-SAKGA---YSGSAVTNLGLGLR-------R
Plu2    --WVTSWDVTVPCPVYVSENQADNIFFEGGFDY-----QDARKTVDGALGYRH------L
Cla1    ERSGFSGNAKALLPIVSEDNPKISYFLQSGIGEF----ANDRTIGHFGGGIRYYPNATAL
Rba1    ----TKTEAMTVYRLKETG--NWFLFNQTSAVN-----FNNRTTINTGFGARH------I
                                                     . .   *
```

5 **6** **7**

```
Mba1    SDNKMLLTGINAFYDHEFP---YDHGRTSIGLEARTTVWEINANKYWATTKWKTGK----
Psp2    INDDTLLVGYNLFYDHELD---YDHQRASIGIEAISSVGSLRANQYYGLSGWKSGL----
Ybe1    FIDD-WMVGYNLFLDRDIT---RDHTRVGAGAEYARDYLKLAANGYLRLSDWRDS--PDF
Ype5    FFAQ-QMLGYNLFIDHDAS---YSHTRIGVGAEYGRDFINLAANGYFGVSGWKNS--PDL
```

Eco14	FTDNGWMLGYNVFYDVDWR---NTNRRYGIGVEAWRDYLKLSANGYKRLSDWRQS--PTV
Sen3	FADR-WMWGINTFYDRQISD--NAHERLGIGGELGWNYFKLSANGYKRLSGWKDS--SEY
Ymo1	YLSD-WMLGYNVFLDQQISG--NAHRRLGLGGELARDYVKLSANSYYRLGGWKNS--TRL
Yen2	YVSD-WMFGYNIFFDQQVSG--NAHRRVGFGGELARDYIKLSANSYHRLGGWKNS--TRL
Pas1	FQGD-WALGYNTFYDIQISG--NAHQRLGFGLEYWRDYLYLSANGYFGLTDWYSS--SAL
Yfr1	FTDS-WMFGVNTFYDYDLT---GKNSRLGVGGEAWTDNLKFSANGYFRLTDWHQSVLADM
Yfr5	FTEN-WMFGVNSFYDYDLT---GKNSRVGVGAEAWTDNLKFSANGYFRLTDWHQSVLSDM
Yps7	FQGN-WMYGANTFFDNDLT---GKNRRIGVGAEAWTDYLKLSANNYFGITDWHQS--RDF
Yfr2	FQNN-WMYGVNTFFDNDMT---GKNRRVGVGAEAWTDYLKLSANSYIGTSDWHQS--RDF
Yfr3	LQDN-WMYGANVFFDRDIT---GKNNRIGFGAEAWTDYLKLSANSYLRLTDWHQS--RDF
Yps4	YQGD-WMYGANTFFDNDLT---GKNRRVGVGAEVATDYLKFSANTYFGLTGWHQS--RDF
Yps2	LENG-WLYGLNTFYDNDLT---GHNHRIGLGAEAWTDYLQLAANGYFRLNGWHSS--RDF
Esa2	YVRD-WMFGGNVFFDDDFT---GENRRIGFGAEAWTNYLKLSANTYIGTSQWHNS--GDF
Plu1	FINN-WMYGANTFYDNDLT---GNNSRFSLGGELWTNYLKMSANAYFRLSDWHNA--RDL
Eco1	FLPE-NMLGYNVFIDQDFS---GDNTRLGIGGEYWRDYFKSSVNGYFRMSGWHES--YNK
Pru1	YGE-GQMIGGNTFFDYDIS---RKHSRLGLGVEYRRDFLKLSANSYHRLSGWRSS--RDL
Pal3	YGE-NQMIGGNSFFDYDIS---RKHSRLGLGVEYRRDFLKLSANSYHRLSGWRNS--RDL
Eta1	FAP-SYMLGGNIFGDYDLS---RAHSRTGIGMEYWRDFLKLSANGYLRLSDWNNS--SDF
Sgl1	FND-GYMLGGNTFLDYDLS---RDHARMGMGVEYWRDYLKIGANNYLRLTNWRDS--KDF
Eco26	FYD-NWMLGGNSFIDYDLR---KEHSRLGLGIEYWQDNLKLGANSYLRLSNWRNS--SNI
Pmi1	FTE-KFMVGINGFYDHDLT---HHHNRLGIGAEIWRDYFKLSSNHYHRLSSWRAS--NNI
Efe5	FTPDNAMLGANIFYDYDLT---RSHSRAGFGVEYWRDYLRFGANTYFGLSDWKNS--RDL
Eco20	FSGNDWMAGVNTFIDHDLS---RSHTRIGVGAEYWRDYLKLSANGYIRASGWKKS--PDI
Eco25	FTP-TWMSGINFFFDHDLS---RYHSRAGIGAEYWRDYLKLSSNGYLRLTNWRSA--PEL
Eco15	FTS-SWMSGINLFFDHDLS---RYHSRAGLGAEYWRDYLKLSSNAYIGLTGWRSA--PEL
Eco6	FTS-SWMSGVNMFIDHDLT---RYHTRTGMGVEYWRDYLKLSGNGYLRLSNWRSA--PEL
Efe2	FMP-DYMTGVNLFFDHDLT---RYHSRMGLGGEYWRDNLKLGANGYLRLTGWRDA--PEL

```
Efe3    FAP-DYMAGVNLFFDHDLT---RYHSRMGLGGEYWRDNLKLGANGYLRLSGWRDA--PEL
Pal2    FHE-KSTIGMNAFIDHDLS---HAHTRVGLGVEYWQDYLKLNANSYFGLTSWKSA--SEL
Csu1    FFRKNWMLGANAFCDCDLT---GTNTRLGLGLESWTNFLQFSMNGYLGLSSPRHS---SL
Eco3    RG--NWLVGYNTFYDNLLD---ENLQRAGFGAEAWGEYLRLSANFYQPFAAWHEQ----T
Cko1    RD--GWLLGYNTFYDNLLD---ENLQRAGFGAEAWGEYLRLSANYYQPFASWHEQ----T
Sen1    QN--GWLLGYNTFYDNLLD---ENLQRAGVGAEAWGEYLRLSANYYQPFADWQTH----T
Eca1    AG--NWLLGYNTFYDNLLD---ENLQRAGLGAEAWGQNLRLSANYYQPFAGWRDD----S
Esp2    AG--KWLLGYNTFYDNLLD---ENLQRAGLGAEAWGENLRLSANYYQPFASWRDS----S
Esa3    AG--HWLLGYNTFYDRLFD---DDTSRAGFGAEAWGDYLRLSANYYQPLGGWQHR----A
Kpn1    VG--GWLLGYNSFYDKVLS---ESLARGSVGAEAWGEYLRLSANYYHPLGDWQLRD---N
Pan1    AG--NWLLGYNSFIDRSFR---TGLQRASVGTEAWSNNLRFSANYYHPLSGWRNRG---D
Eta2    AG--SWRVGYNAFVDGLLG---SDRQRGSLGAEAWGEYLRLSANYYHPLSGWRNRS---N
Spr1    AG--DWLLGYNTVLDSDFE---RHHNRASLGAEAWGDFLRFSANYYYPLSALAQQR---D
Yin1    QG--DWLLGANTFFDQDIS---RNHSRLGLGLEWWADNLKLATNYYHPLSGWKDSK---D
Yin2    QG--DWLFGANTFLDQDIS---RNHSRLGLGLEWWADNLKLASNYYHPLSGWKDSK---D
Yfr4    KG--NWLLGYNTFLDQDFS---RNHSRLGLGAELWTESLRLATNFYHPLSGWKDSK---D
Ymo2    LPSLNGLLGYNVFIDHDFS---RGHNRAGIGAEARADYLKFSANYYHPLSHWKDSP---D
Eco10   FD--KYLLGGNIFYDYDFT---RGHRRLGLGAEAWTDYLKFSGNYYHPLSDWKDSE---D
Eal1    FD--KWLLGGNIFYDYDFT---RGHRRLGLGTEAWTDYLKFSGNYYHPLSGWKNSE---D
Sty4    VG--NWLLGGNAFYDYDFT---RGHRRLGLGTEAWTDYLKFSGNYYHPLSDWKDSE---D
Eco16   IG--SWLLGGNLFYDYDFT---RGHRRLGLGSEAWTDYLKFSGNYYHPLSDWKDSK---D
Ahy1    LDR--WMLGYNLFADYDLT---NRHWRAGVGAEAWRDYLKLGANFYTPLSSWRDSP---R
Sen2    VNG--WLLGVNTFLDADIR---YSHLRGGIGGEVYKESLAFSGNYYFPLTGWRTS---AA
Efe4    VGD--WLLGINSFLDADVR---YDHLRGSLGVELFRDSMSLAGNWYFPLSDWKAS---KV
Bpe1    EVNDALMVGANGFLDYEFG---KQHLRGSVGLEVIAPEFSLYGNVYAPLSDWKGAK---R
Bav2    DINERLMLGANAFLDYEFA---KQHLRGSLGVEAIAPEFSFYGNVYAPMSGWTGAK---R
Bbr1    ALNQHMAVGANAFLDYEFG---KNHLRGSLGGEVIAPQFTLYGNVYAPMSGWKAAK---R
```

```
Bpa2            AAGSSLILGGNAFLDYEVG---KRHLRGSLGAEAVAAQFTLYGNVYAPLSGWKAAK---R

Bav1            QASPGLIVGSNAFLDYEFG---KQHVRGSLGLEAIAPHYSLYANYYAPLSGWKGAR---R

Pma1            RPDDISMVGANAFWDYRMTDYSDAHSRLGLGGEYFWKDFEFRNNWYMAITNEKDVIIKGV

Pma3            RPNDISMVGANAFWDYRMTDYSDAHSRLGLGGEYFWKDFEFRNNWYISITDEKDVTIKGV

Pma5            RPNDVSMLGANAFWDYRMTDYSDAHSRLGLGGEYLWKDFELRNNWYMSITNEKDVTIKGV

Pma4            RPNNESMMGANAFLDYRMTDYSDSHSRLGLGGEYLKNGWELRNNWYMSMTEQKNVTVNGT

Ssp1            RLGDEAIVGLNGFWDLRTTNYSTAYTRWGIGAEGFWKSFELRNNWYINGSADKNITINNI

Ssp2            RLRDNAMLGANAFWDYRFTNYSSSYSRWGAGAELWWDDFKLTNNWYIAGTGIKRITTSGR

Plu2            MSDNKVMLGANVLYSHEFP---RNHQRISYGAEIRTSVFEINSNYYHRLTDWKLTG---V

Cla1            NNSGNIMLGLNSVYDHDFS---RGHKRMSLGAEAMVDTLAFNANVYQRLSSWIDSY--DF

Rba1            NDANTVITGYNIFYDYELQ---SKHERVGAGLELLSSIFEFRANAYQAVSKTLTYN----

                        * * . .           * . * *          * *
```

```
                                   8          9

Mba1            -NGLE--------------------ERALDGYDIEAGVPLPYMNWATVFVKNFQWDSEIS

Psp2            -NNIN--------------------EKALNGSDVELGMPLPYLPWTNLYYRSFNWEG-AS

Ybe1            S-SYS--------------------ERPATGFDLRAEAYLPSLPQLGGKLMYEQYFGNDV

Ype5            D-KYD--------------------EKVANGFDLRSEAYLPTLPQLGGKLIYEQYFGDEV

Eco14           T-DYD--------------------ERPADGWDIRAEGWLPAYPQLGGKLVYEQYYGNEV

Sen3            E-DYQ--------------------ERVANGYDIRAEGYLPAWPQLGAQLVWEQYYGDDV

Ymo1            E-DYD--------------------ERAASGYDIRTEAYLPYYPQLGGKLMYEQYFGNEV
```

Yen2	E-DYD--------------------ERAANGYDIRTEAYLPHYPQLGGKLMYEQYFGDEV
Pas1	D-GYA--------------------ERAANGYDIRAQGWFPVYPQLSGKLKFEQYFGDDI
Yfr1	E-DYN--------------------ERPANGFDVRAEAYLPSYPQLGGRLMYEKYFGKGV
Yfr5	E-DYN--------------------ERPANGFDVRAAAYLPAYPQLGGSLMYEKYFGKGV
Yps7	I-DYN--------------------ERPANGYDLRAEAYLPSYPQLGGKAMYEKYRGDDV
Yfr2	A-DYN--------------------ERPANGYDVRAEAYLPSHPQLGGKLMYEKYRGEEV
Yfr3	A-DYN--------------------ERPANGYDLRVEAYLPAYPQIGTNLKYEQYKGNEV
Yps4	S-SYD--------------------ERPADGFDIRTEAYLPAYPQLGGKLMYEKYRGDEV
Yps2	S-DYK--------------------ERPATGGDLRANAYLPALPQLGGKLMYEQYTGERV
Esa2	D-NYN--------------------EKPADGYDVRAEGYLPSFPQLGAKLMYEQYYGDNV
Plu1	V-NYY--------------------ERPANGYDLIADMYLPSMPSLGAKIKYEQYFGDNV
Eco1	K-DYD--------------------ERPANGFDIRFNGYLPSYPALGARLMYEQYYGDNV
Pru1	A-DHS--------------------ARPSNGWDVRAEGWLPSYPHIGGKLTYEQYYGDSV
Pal3	A-DYS--------------------TRPANGWDLRAEGWLPIYPHIGGKLTYEQFYGEEA
Eta1	K-DYQ--------------------ERPANGWDIRAQAWLPSLPQLGGKLTYEQYYGRGV
Sgl1	A-DYQ--------------------ERPANGWDMSLEGWVPALPQLGGNLKYEQYYGKEV
Eco26	V-DYE--------------------ERPANGLDLNIKSWLPSYPQIGGDIKYEKYYGDDV
Pmi1	L-DYS--------------------ERPANGWDIRTEGYFPAYPQLGTKLIFEQYYGKEV
Efe5	A-DYL--------------------ERPANGWDVSAEGWLPAYPQLGASVQFEKYYGKNV
Eco20	E-DYQ--------------------ERPANGWDIRAEGYLPAWPQLGASLMYEQYYGDEV
Eco25	DNDYE--------------------ARPANGWDVRAEGWLPAWPHLGGKLVYEQYYGDEV
Eco15	DNDFE--------------------ARPANGWDLRAEGWLPAWPQLGGKLVYEQYYGDEV
Eco6	DNDYE--------------------ARPANGWDLRAEGWLPAWPQLGGKLVYEQYYGDEV
Efe2	DYDYE--------------------ARPANGWDVRAEGYLPAYPQLGAKLMYEQYYGDEV
Efe3	DYDYE--------------------ARPANGWDVRAEGYLPAYPQLGATLMYEQYYGDEV
Pal2	NHDFN--------------------AKPAHGWDIQVEGWLPNYPHLGGNLRYEQYYGDSV
Csu1	QPNYQ--------------------ESPAHGLDFRLHYWLPFFPRLGGKIFYEQYFGKQV

```
Eco3     ATQE---------------------QRMARGYDLTARMRMPFYQHLNTSVSVEQYFGDRV
Cko1     TALE---------------------QRMARGYDVTAQMRMPFYQHLNTSVSVEQYFGERV
Sen1     ATLE---------------------QRMARGYDINAQMRLPFYQHINTSVSLEQYFGDSV
Eca1     DVQE---------------------QRMARGYDVTAKAWLPWFHHLNTSVSFEQYFGDSV
Esp2     DLQE---------------------QRMARGYDVTAKAWLPYFHHLNTSVSFEQYFGDNV
Esa3     GLLE---------------------QRMARGYDVTAQAYLPFYQHINTSVSFEQYFGDQV
Kpn1     QTQE---------------------QRMAAGYDVTAQARLPFYQHINTSVSVEQYFGDSV
Pan1     SQQ----------------------SRMAEGYDITTQSYLPFYRQLGVSFSYQQYLGEKV
Eta2     SSQ----------------------MRMARGYDITTRGYLPFYHQLGVTLSYEQYLGDRI
Spr1     NAQFL--------------------SRPASGYDITTQGYLPFYRQIGGSLSYEQYWGENV
Yin1     FDDYL--------------------ERPARGFDVRAQGYLPAYQQLGASVVYEQYYGDEV
Yin2     FDDYL--------------------ERPARGFDVHAQGYLPAYQQLGASAVYEQYYGDEV
Yfr4     FDDYL--------------------ERPAKGFDVRLQGYLPAYPHLGASVVYEQYYGDEV
Ymo2     FDDYL--------------------ERPAKGYDLRSQGYLPAYPQLGVSAVYEHYFGDEV
Eco10    FDFYE--------------------ERPARGWDIRAEAWLPAYPQLGGKIVFEQYYGNEV
Eal1     FDFYE--------------------ERPARGWDIRAEAWLPAYPQLGGKIVFEQYYGDEV
Sty4     FDFYE--------------------ERPARGWDIRMESWLPFYPQLGAKLVYEQYYGDEV
Eco16    FDFYE--------------------ERPARGWDVRAEGWLPAWPQFGAKLVYEQYYGDEV
Ahy1     FEGME--------------------ERAARGMDVRLEAYLPAYPQWSASLTAEQYLGERV
Sen2     HEFHD--------------------ERPAYGFDLRTKGTLPDFPWFSGELTYEQYYGDKV
Efe4     QPLHD--------------------ERPATGIDVRLKGALPSLPWFGAELAFEQYFGDKV
Bpe1     NNRRE--------------------EKPASGMDVGVGYRPAFAPGLSLSATHFRWNGAEV
Bav2     DNRRE--------------------ERPASGMDLGMKYSPGFAPGLSLKANYFRWNGAAV
Bbr1     AERRE--------------------ERPASGWDVGVRLQPEALPGLAIKGQYFRWSGAAV
Bpa2     AERRE--------------------ERPAAGWDVGFTARPEAVQGLALNAQYFRWRGAQV
Bav1     DSRRE--------------------ERPAAGYDLGGQLS--SDAGLSLQAAYFRWHGAGI
Pma1     DYQ----------------------ERVVPGWDLEVGYRLPNNPELAFYIRGFNWDYKYT
```

```
Pma3     SYK-----------------------ERVVPGWDVELGYRLPNNPELAFFVRGFNWDYKHT

Pma5     SYK-----------------------ERVVPGWDVELGYRLPNNPELAFFVRGFNWDYKNT

Pma4     VYT-----------------------ERVVPGWDVEAGYRFPNHPEMAVFVKGFNWDYKNT

Ssp1     DYV-----------------------ERVVPGWDVEVGYRIPSYPQLAIFVRGFNWDYQDH

Ssp2     AYTDTTSLAAGTYDETTLLGANTFDERVVPGWDVALNYRLPSYPQLSLGIRGFRWDYMRK

Plu2     DNNE----------------------EKARGGYDVELALAVPYVPSAHFRVKHFCWNGIAS

Cla1     DKDYVQ--------------------ERPANGWDAKIKYAFPSLINVSFFAKMGQWYGNKV

Rba1     --GIQ---------------------ETALDGYDAKLTANLPYFYSS-------NLYGKLS

                                        *  *
```

10 11 12

```
Mba1     GSKD---------IKGNDLQLRAYIPGITGLEIQAGRTFFSDSSGTD--ENYINIFYNVT

Psp2     GAAD---------LEGDEISLEAKLTNFN-IEIGK-RS--NDGVTED--EEFLKITYTCC

Ybe1     GLFG--KDN--RQQNPAAITAGINYTPIPLVTVGIDRKQGSAGNGET--LFNLGVNYEVG

Ype5     GLFG--VDN--RQKNPLAVTLGVNYTPIPLFTVGVDHKMGRAGMNDT--RFNLGFNYAFG

Eco14    ALFG--ESE--RQKNPHAITAGVTWTPFSLLTAGVDYRRGKNGADDT--RLNLGLTYRIG

Sen3     ALFD--DSEDDRQRNPYAVTAGVNYTPFPLVSIGLNQKMGKGNHNDT--QIDLAVNWMLG

Ymo1     ALFG--LNE--RQKNPSALTASVSYTPFPLVNLALEHTIGNSGKNKT--GVNLAVNYEIN

Yen2     ALFG--INE--RQKNPSALTAGVSYTPIPLVSLGLDHTIGNGGKKKT--GVNVAVNYEIN

Pas1     ALLN--HQN--RYKNPYALTMGLEYTPIQLISLGIDRTFSHRGKDDT--KVNLSFNYQLG

Yfr1     ALNSGSTSPDDLGDSPSAFTVGLNYTPIPLFTVDVAHKKGQNTNNEL--QLGLNFNYRFG
```

```
Yfr5    ALNSGSTSPDDLGDSPSAVTLGVNYTPIPLITVDVAHKKGQNTSNEL--QVGLNFNYRFG
Yps7    ALFG----KDNRQKNPHAITAGVNYTPIPLVTIGAEHRAGKGGQNDS--NINFQLNYRLG
Yfr2    ALFG----KDNRQKNPHAVTAGVNYTPIPLLTVGAEHRAGKGSKNDS--SINFQFNYRLG
Yfr3    ALFG----KDDRQKNPYAFTAGINYTPIPLITIGAEQRAGKGGRNDT--NISIQLNYRLG
Yps4    ALFG----KDDRQKDPHAVTLGVNYTPVPLVTIGAEHREGKGNNNNT--SVNVQLNYRMG
Yps2    ALFG----KDNLQRNPYAVTAGINYTPVPLLTVGVDQRMGKSSKHET--QWNLQMNYRLG
Esa2    ALFD----KDHLQSNPSAVTVGLNYTPVPLITAGIDYKRGQDSMDEM--KFSLNFHYALD
Plu1    ALFG----KNKRQKDPYAATIGVNYTPIPLITAGIDYKLGKEGKSDG--IFSFNVNYRFG
Eco1    ALFN----SDKLQSNPGAATVGVNYTPIPLVTMGIDYRHGTGNENDL--LYSMQFRYQFD
Pru1    ALFG----TKNLQQNPYSITAGLNYTPIPLVTFNAEHRQGKASKQDS--RFGLQLNYQFG
Pal3    ALFG----TKNRQNNPYSITAGINYTPIPLITLNGEHRQGKASKQDS--RIGLKLSYQFG
Eta1    ALFG----KENLQQDPRAITAGVNFTPFPLLTLNAEHRQGASGKNDK--RLGVDFSYQLG
Sgl1    ALFG----KDNRQKDPHAITVGVNYTPFPLLTFSADQRQGKAGQNDT--RLGVQLNIQLG
Eco26   ALFG----ENHRQRNPHSTTLGISYTPFPLMSFKAEHKMGSNNINDS--RIGFEINYQIH
Pmi1    GLFG----KDKRDKNPHTYTLGINYTPIPLVTLNAERRIGLHDRADN--NLNINLSYRIG
Efe5    GLFG----SDNLQENPYAVTGEISYTPVPLVKFSAQHRQGQSNTHDT--RFGVEINYRPG
Eco20   GLFG----KDKRQKDPHAISAEVTYTPVPLLTLSAGHKQGKSGENDT--RFGLEVNYRIG
Eco25   ALFD----KDDRQSNPHAITAGLNYTPFPLMTFSAEQRQGKQGENDT--RFAVDFTWQPG
Eco15   ALFD----KNDRQSNPHAITAGLNYTPFPLLTLSAEQRQGKQGENDT--RFAVDLTWQPS
Eco6    ALFG----KDERQNDPHAITAGLSYTPVPLISFSAEQRQGKQGENDT--RIGMELTLQPG
Efe2    ALFG----RDHRQKDPHAFTAGVSYTPVPLVSLSAEQRQGKGGENDT--RFGLNLSYTPG
Efe3    ALFG----KDKRQQDPHAFTAGLSYTPVPLISLSAEQKQGKGGENDT--RFALNLTYTPG
Pal2    ALFG----KTKRQKNPNAATIGANWTPFPLFTLNASHKLGSEKQVET--QAKLQFTWTFG
Csu1    SLFG----RSDLQHDPFVITTGVEFTPFPLLSMEIDHRMSKASRHYT--LFNIRLNYRFG
Eco3    DLFN----SGTGYHNPVALSLGLNYTPVPLVTVTAQHKQGESGENQN--NLGLNLNYRFG
Cko1    DLFD----SGTGYHNPVAVKLGLNYTPVPLVTVTAQHKQGESGVSQN--NLGLNLNYRFG
Sen1    DLFD----SGTGYHNPVALKLGLNYTPVPLLTMTARHKQGESGVSQN--NLGLTLNYRFG
```

Eca1	DLFN----SGTGYHNPMAVNLGLDYTPVPLVTISAAHKQGESGVSQN--NLGLKLNYRFG
Esp2	DLFR----SGTGYHNPMAVNLGLDYTPVPLLTFSAAHKQGESGVSQN--NLGMKLNYRFG
Esa3	ELFD----SGTGYHNPVAVKVGLSYTPVPLVTVSAHHRQGESGVSQN--DLGLKLNYRFG
Kpn1	DLFH----SGTGYHNPVAVSVGLNYTPVPLVTVTAKHKQGENGVSQN--NVGLKLNYRFG
Pan1	DLFN----SGNRYHNPAALSLGLSYTPVPLVTISASHKTSSAGESQD--QLGLKLNYRFG
Eta2	DLFN----SGNAVADPSAVSLGINYTPVPLFTLAASRKEGEGGESQN--QFTLKMNYRIG
Spr1	DLFG----SGKKQNDPRAMQLGVNYTPVPLVTVKALHKMGEGGVSQD--QVELALNYRLG
Yin1	ALFG----KDNLQKDPSAVTVGVDYTPFPLATLKLIHKRGQQGKNNT--EVGLRVSYQIG
Yin2	ALFG----KDNLQKDPHAVTVGVDYTPFPLATLKVSHKMGKDGKNNT--ELGLQVSYQIG
Yfr4	ALFG----KEHLQKDPRAVTLGLDYTPFPLTTLKVSHKEGQQGQKEA--QVDLQMNYQIG
Ymo2	ALFG----KSHRQKDPRALTLGIDYTPVPLVTLGAKHKYGQQGKKDT--QIDVAFRYQFG
Eco10	ALFG----TDSLEKDPFAVTLGVKYQPVPLIVVGTDFKAGTGDNTDL--SVNATLNYQFG
Eal1	ALFG----TDNLEKDPHAVTLGLNYQPVPLITVGTDFKTGTGDNTDV--TVNATLNYQFG
Sty4	ALFG----TDNLQKDPHAVTLGLEYTPVPLVTVGSDYKAGTGDNNDF--SVKATVNYQIG
Eco16	ALFG----TDNLEKNPQALTVGLMYNPVPLFTVGSDYKSGTGDNSDL--SVNVSLNYQIG
Ahy1	GLLD----ADQLERDPHAITAGLHYNPFPLLKMDVEQVEASGRQHDT--RFTLGLEWKLG
Sen2	DLLG----NGTLSRNPRAAGAALVWNPVPLLEVRAGYRDAGNGGSQA--EGGLRVNYSFG
Efe4	DILG----NDSLTRDPAAFTGAITWKPVPLVEIKAGYKDAGSSGSQT--EAGLNLNYTFG
Bpe1	DYFD----NGRTQAGAKGFKVGVEYRPVSLVSVGLEQTKVIGGGRET--RMQLGLNINLS
Bav2	DYFD----NGRTQDRATGFKYGVQYKPVPLLSLGVEQTRVIGGASQT--SVQLGVALNLS
Bbr1	DYFD----NGRPQRNARGYKYGVEYRPVPLVAVGLEQTKVLGGARQT--TVQLGVNLSLG
Bpa2	DYFD----DGRYRRNPSGFKYGIEYRPVPLIGVGVEQARLQSGERQT--SVQLGVRLNLG
Bav1	DVFD----SGRAQRNASGFRYGVAYQPGALFNIGLNQTRTLDGQKQT--SVQLNVRINLQ
Pma1	QDNS--GLEGAVSWQATPHVGLEAYVSNEISAASTTANTDLPGTDEN--FFGLRMNITGN
Pma3	QDNS--GLEGAVSWQATPHVGLEAWVSNEISAASTTVNTSLPGTDET--FFGLRMNITGN
Pma5	QDNS--GLEGSVRWQATPHLGWEAYVSNEISAASTTVNTSLPGTDEI--FFGLRVNITGN
Pma4	QDNS--GIEGSFNWQATPHVNWEAWVSNEISGAKTVKNSDLPKTDET--FFGLRFRWTAR

Ssp1	SDNS--GIEGSVNWQATPHANLELWVSNEIPAYPTDSNDTIGNQPGP--YIGARVRLTGR
Ssp2	SDNS--GVEGSVNWQATPHTNLSAWISSEIPAYPAQSNAQLSSGDDV--YVGVRFNVQLK
Plu2	NDSN---NPIDDLKGNTFSVSGSVYDGLSVEVGYIDYTSGNADYSKAGGERFLKVSYNFD
Cla1	GIFG-ANSVDDLEKNPLIYEGGISYSPFPALTFTLSHSRSAESSKKN-TSINANINIPLD
Rba1	NWKD----AASYETEHYEAGINAEIAPNLTLRVAAQHKKNSNNTEAV---ASINYSVPLG

13

Mba1	QLFADKPRY----------NHQWISKDAYKLESMEDRRYEKVRRTN--------------
Psp2	NNSN---------------NEIGISDTAYNLTSVSDQKFAKVRRQN--------------
Ybe1	TPWAK----QISP--DAVNARRTLQGSRNDLVERNNQIVLEYKKQD-VINLYV-SNNVSG
Ype5	TPLTH----QLDS--DAVAIKRSLMGSRYNLVDRNNQIVMKYRKQN-RVTLEL-PARVSG
Eco14	EPLAH----QLDS--SRVGAQRSLAANRLELVNRNNDVVLEYRKQT-LITLQL-PPDVYG
Sen3	SSLKS----QLDS--DAVKARRTLLGSRLDLINRNNNIVLEYRKQD-LISLKV-QNKVTG
Ymo1	TPWQK----QIDP--AAVKATRTLAGSRMDLVDRNNNIVLEYRKQQ-VVTLNL-PAKVSG
Yen2	TPWQQ----QIDP--AAVQTTRTLAGRRMDLVDRNNNIVLEYRKQQ-VVTLNL-PEKVSG
Pas1	VPLSQ----QIDP--TVAPVKRTLADNRYHLVERNNNIVLKHRERA-QLSLYL-PTGLSG
Yfr1	VPWVD----QINK--NAVGLMRSLMGSRYDIVDRNYNIVMQYEKQD-LIKLTL-PETLAA
Yps7	ETWQS----HIDP--SAVAASRTLAGSRYDLVERNNHIVLDYQKQN-LVRLSL-PDSLAG
Yfr2	ESWQS----HINP--SAVAATRTLAGSRYDLVERNNNIVLDYQKQE-LIRLSL-PERVEG
Yfr3	EPWQS----QIDP--SAVAASRTLAGSRYDLVERNNNIVLEYQKQD-LIQLVL-PNQMTG

Yps4	QPWND----QIDQ--SAVAANRTLAGSRYDLVERNNNIVLDYKKQE-LIHLVL-PDRISG
Yps2	ESFQS----QLSP--SAVAGTRLLAESRYNLVDRNNNIVLEYQKQQ-VVKLTLSPATISG
Esa2	SSWQS----QISP--EQVATRRSLAGSRYDLVDRNNEIILQYKKK---------------
Plu1	VPLSE----QLSP--ENVSSLRSLAGSRYDLVERNNNIILNYLKKQ-QHFRLL-VPVIEI
Eco1	KPWSQ----QIEP--QYVNELRTLSGSRYDLVQRNNNIILEYKKQD-ILSLNI-PHDING
Pru1	KTWKQ----HLDP--GSVTTFRSLMGNRYDFVSRNNNHIVLEYKKND-VIQLNI-ANSITG
Pal3	KSFKQ----HLDP--DAVGEFRSLMGNRYDFVSRNNHILLDYKKND-TIYLNM-NASITG
Eta1	MPWQQ----QINP--QAVATMRSLAGSRYDLVERNNHILLQYRKKE-VIRLHT-VGRVTG
Sgl1	TPWQH----QLDT--SAVGAMRTLAGSRYDLVDRNNNIVLEYRKKE-VIHLYT-ADHLAG
Eco26	TPWES----QINP--VLIPAMRKLAGQRYDLVERNNNIILDYRKKE-IIKIDG-VDVISG
Pmi1	ESLAS----QLNP--DNVKAIRTLAGSRYDFVNRNNDMILEYKKET-LVFLSM-VDSING
Efe5	VPLSK----QLDS--DNVAAMREVQNRRYDFVERNNNIVLEYKKKS-TLRISL-PDAIEG
Eco20	EPLAK----QLDT--DSIRERRVLAGSRYDLVERNNNIVLEYRKSE-VIRIAL-PERIEG
Eco25	SAMQK----QLDP--NEVDARRSLAGSRFDLVDRNNNIVLEYRKKE-LVRLTL-TDPVTG
Eco6	HSLQK----QLDP--AEVAARRSLVGSRYDLVDRNNNIVLEYRKKE-LVRLTL-TDPLKG
Efe2	VSLAR----QLDP--DAVAYRRSLSGSRHDLVERNNNIVLEYRKKE-LVKLQL-NDPVTG
Efe3	VSLAH----QLDP--DAVAYRRSLAGSRHDLVERNNNIVLEYRKKE-LVKLQL-HDPVTG
Pal2	KNLAH----HLDP--TKVAETRRLSGNRYDFVERNNNIILNYQKKT-VLHLSL-PSKIQG
Csu1	ESLSS----QLNP--FSVAKMRFLNENRYNFVSRNHVIVFNYSEYT-YDIFVS-PASISA
Eco3	VPLKK----QLSA--GEVAESQSLRGSRYDNPQRNNLPTLEYRQRK-TLTVFLATPPWDL
Cko1	VPLNK----QLSA--SEVAESQSLRGSRYDNPQRNNLPTMEYRQRK-TLTVFLATPPWDL
Sen1	VPLKK----QLAA--SEVAQSQSLRGSRYDMPQRNALPTMEYRQRK-TLTVFLATPPWDL
Eca1	VPLAK----QLSA--GEVAATRSLRGSRYDPAERNSLPVMEFRQRK-TLSVYLATPPWDL
Esp2	VPLKK----QLSS--EEVATTRSLRGSRYDPVDRTSLPVMEFRQRK-TLSVYLATPPWDL
Esa3	VPLTK----QLSP--DEVAASRSLRGSRYDRVERTNVPVMEFRQRK-TLSVFLATPPWDL
Kpn1	VPLKQ----QLAA--DEVAISNSLRGSRFDSPERDNLPVVEYRQRK-NLTVYLATPPWDL
Pan1	VALSK----QLDA--NNVAEARSLRGSRYDTVTRSQNPVMAYRQRK-TLSVFLATPPWQL

Eta2	VPLRQ----QLSS--DNVAEARSLSGSRYDSVNRDNSPVMAFRQLK-TLSVFLATPPWQL
Spr1	VPLVK----QISP--EYVAQAKSLRGSRYDNIERKNVPVMAFKQRK-TLQVFLATPPWRL
Yin1	TPLDK----QLDP--ANVAAMRSLKGSRYDLVDRNNDIVLEYKEKA-SLALDLAAVPMTL
Yin2	TALEK----QLDP--GNVAAMRSLKGSRYDLVDRNYDIVLEYKEKA-VLSLDLAAVPMTL
Yfr4	TALSK----QLDP--DNVAAMRSLMGSRYDRVDRNYDIVLEYKEKAGLLMLDLAAVPATL
Eco10	VPLKD----QLDP--DKVSAAHSLMGSRHDFVERNNFIVLEYKEKD-PLYVTLWLKADVT
Eal1	TPLKD----QLDP--ENVKIAHSLMGSRHDFVERNNFIVLEYREKD-HLDVTLWLKADAT
Sty4	TPLAA----QLDP--ENVKIQHSLMGSRTDFVDRNNFIILEYREKD-PLDVTLWLKADAT
Eco16	MPLKD----QLDP--DNVKAKHSLMGSRHDFVERNNFIVLEYREQD-PLDVTLWLKADTA
Ahy1	ATLWD----MLNP--SSVD--KSLAGMRHDLIERNNDMVLEYRDKV-LLKASL-NDQYSA
Sen2	TPLHE----QLDY--RNVGAPSNTTN-RRAFVDRNYDIVMAYREQA--SKIRITAMPVSG
Efe4	VPLRA----QLDP--SQVRPASNTTN-RTAFVDRNYNIVMEYREQA--SRIRVYASPVNG
Bpe1	EPLSK----QLRR-DASGTPAFSPDARRHALVERENRIVLNTRRKEIILPLVVSKVSTLQ
Bbr1	EPLSR----QLR---HQSGPAFDLQARMGEFVERENRIVLQTRRKHVVLPLTIARVDTDP
Bpa2	EPLSR----QLRRGAQDTAPPFDRGARLQDFVRRENRIVLDTRRKKIVLALRIAEVRTDP
Bav1	EPPSR----QLRR----ESQPFNLTSRRHQWVERESRIVLNTRRKAITLPLSIAQLRGDP
Pma1	PVKFEK-----------SNYKKNMVTQMTQPVKRKYDVLLERSAG----------GVFQN
Pma3	PIKFEK-----------SNYKKNIITQMTQPVKRVNDVLLERAAINSS-----GAATFTV
Pma5	PVTFGK-----------KDYKKNMITQMTQPVKREYNVLLERYGAN---------STFTN
Pma4	PVKFAK-----------KNLKQNLVTQMTQPVRRRYEVLLERSTGG-----------FQN
Ssp1	PVVFTK-----------SNTKQNLLTQMTQPVRRRYEVLLERVKEPTK-----GENGSIT
Ssp2	PVTYKTG---------SNRIRENLLTQMRQPVQRRNDVLLERWKPKQKKTIGGVNGSFIN
Plu2	IFGTH----------VNKATKPRFSNTPYEFERMDDRRFEKIRFEQKVISAHNNEQPPA-
Cla1	EKAMK---LAFEP--KLAGISNTIEGTRTQFIDRDYSMVLEYRATPNKYHISYCGDLGND
Rba1	GANQP---AKVKQDGDWSTKFEPIREKLYRPVQRENRIMKKAIKLG---------VTVSG

14

```
Mba1     -----------------------NIVKQIKSLGVKVSGI---------------------
Psp2     -----------------------LIVKQ-KEMDLTVIGF---------------------
Ybe1     RAA------ET-----------KQLVVSV-TSKYGLRNIQFDQGA---------------
Ype5     AAR------QT-----------MPLVANA-TAQQGIDRLEWEASA---------------
Eco14    AEL------TT-----------VTLTPQV-NAKYGLSRIELDDAE---------------
Sen3     TES------ET-----------LPVSVNV-KSKYPLDHISWEDDN---------------
Ymo1     KEK------QV-----------LPINYTF-NARHGLDRIEWDAAD---------------
Yen2     KEK------QV-----------VPINYTF-NARHGLDRIEWDAAD---------------
Pas1     FGG------ER-----------KLINFSF-NGKYRLKHIQWNDGA---------------
Yfr1     YAI------TN-----------LSLTGNI-TAKYGAERMEWSAPA---------------
Yfr5     HAL------EN-----------VTLTGTV-ASKYGTERIEWSAPV---------------
Yps7     DPF------SQ-----------LSVTAQV-TATHGLERIDWQSAE---------------
Yfr2     KAG------DI-----------ATVNAQV-TSKYGLERIDWDSAA---------------
Yfr3     SAF------EI-----------IKVEAQV-TAKYGLKRIDWDTAV---------------
Yps4     SGG------GA-----------ITLTAQV-RAKYGFSRIEWDATP---------------
Yps2     LPG------QV-----------YQVNAQV-QGASAVREIVWSDAE---------------
Esa2     ------------------------------------------------------------
Plu1     SSY------GG-----------EVKPIQI-QSDTPFKNVTWDIPE---------------
Eco1     TER------ST-----------QKIQLIV-KSKYGLDRIVWDDSS---------------
Pru1     YAG------EK-----------IPLSFTV-ASKYGLSHLKWNAET---------------
```

```
Pal3     ISG------EK-----------IPLKYTI-NSQYGFNRINWHADS---------------
Eta1     YAG------ER-----------KSLGVSV-NSSYGLERIDWSASS---------------
Sgl1     YAG------EQ-----------KSLNVSI-NTKYGLERIDWSAPE---------------
Eco26    FSG------EK-----------KRLDIRV-NSKYPVDRIDWLANT---------------
Pmi1     YAK------EE-----------RDLQVQV-KTKYPLANIEWSASK---------------
Efe5     ESG------AT-----------IPVTLNVSHASNGIQSVQWSDSA---------------
Eco20    KGG------QT-----------LSLGLVVSKATHGLKNVQWEAPS---------------
Eco25    KSG------EV-----------KSLVSSL-QTKYALKGYNVEATA---------------
Eco15    KSG------EI-----------KPLVSSL-QTKYALKGYNIEAAA---------------
Eco6     KPG------EV-----------KSLVSSL-QTKYALKGYDIEAAS---------------
Efe2     KGG------EQ-----------KPLVASL-QSKYALKTLQTDAAT---------------
Efe3     KGG------EQ-----------KPLVASL-QSKYALKTLRAEAAE---------------
Pal2     ITG------QS-----------VPLVKSF-TSKYPLKHIEWQAPE---------------
Csu1     IEF------AS-----------STVSLKCPNTKT-LAHFEWEAQN---------------
Eco3     KPG------ET-----------VPLKLQI-RSRYGIRQLIWQGDT------------QIL
Cko1     QPG------ET-----------VPLKVQI-RSRHGVRHVTWQGDT------------QAL
Sen1     TPG------ET-----------VTLKLQV-RSVHGIRHLSWQGDT------------QAL
Eca1     KGG------ET-----------VMLKLQI-RSTHGIRQLHWQGDT------------QAL
Esp2     TPG------ET-----------VVLKMQV-RSTHGVRQLHWQGDT------------HAL
Esa3     SAG------ET-----------VALKLQV-RSRHGIRQLSWQGDT------------QAL
Kpn1     QSG------ET-----------VQLKLQI-HSLHGIKALHWQGDT------------QAL
Pan1     NPG------ES-----------VPLKLQL-RASHSIKALSWQGDT------------QAL
Eta2     QPG------ET-----------LRLKLQI-ANKNTIKAVSWQGDT------------QAL
Spr1     QPG------ET-----------LPLVLEI-KTTNKITRVSWQGDT------------QAL
Yin1     LEG------DI-----------YLMQPLV-RSKYKIVGVTWNGDT------------VPL
Yin2     LEG------DV-----------YMMQPLV-RSKYRITSVSWHGDA------------VPL
Yfr4     LEG------DV-----------YLMQPLV-NGKYPIASVSWQGDT------------VPL
```

```
Ymo2     MEG------ES-----------YILRPLV-KSKYPIIDLIWLGDL------------LPL
Eco10    NEHPECVIKDTPEEAIGLEKCKWTINALI-NHHYKIVAASWQAKNNAASWQAKNNAARTL
Eal1     NEHPECVIKDTPEEAVGLEKCKWTVNALI-NHHYKIVAASWQAKN---------NAARTL
Sty4     NEHPECVIEDTPEAAVGLEKCKWTVNALI-NHHYKIISASWQAKN---------NAARTL
Eco16    NEHPECIVKDTPELAVGLEKCKWTINALI-NNHYKIVAASWQAKN---------NANRTL
Ahy1     VEG------QA-----------LTLTLNI-QHSRQIASIQWLGDVLG-------------
Sen2     LSG------------------TLVTLMATIDSRYPIEKVEWSGDA---------------
Efe4     QSG------------------DTVTLSATINSRYPVERIEWTGDA---------------
Bpe1     ADG--------------------RVTVIGATQPFATVTVRMPDGM-------------TG
Bav2     VSG--------------------RITLVGQTQAQATVNWTLPDGS-------------SG
Bbr1     ATG--------------------RITVTGVTEPGAQVSLGLPNGE-------------VV
Bpa2     ATG--------------------RITVYGVTEPLADVQLWLPDGT-------------AT
Bav1     TNG--------------------AIEVSGQTEAGARIMLTFPDGS-------------GN
Pma1     R-------------------------------AKGV------------------------
Pma3     RVS-----------------------------AQGT------------------------
Pma5     R-------------------------------AKGS------------------------
Pma4     R-------------------------------ARGI------------------------
Ssp1     S-I-----------------------------AQGI------------------------
Ssp2     Q-------------------------------AAGI------------------------
Plu2     ------------------------------------------------------------
Cla1     KHCILLKN-----------GFDEVVKNTPMRVTPTHSCVVFEKGFD--------------
Rba1     Y-----------------------------------------------------------
```

	15	16
Mba1	----------------------------	
Psp2	----------------------------	
Ybe1	-LAAAGGKIIP----QGPSQFALQLPPQ	
Ype5	-LTLAGGKITG----SG-NNWQITLPSY	
Eco14	-LRQAGGKIIS----NTGNQITLQLPAW	
Sen3	-LVKNGGKISE----NNG-SWSVTLPHY	
Ymo1	-VINAGGNISD----QGNLAYHITFPPY	
Yen2	-VIKAGGQVIN----QGNLAYYIAMPPY	
Pas1	-LRARGGRIIA----LSNNSYVVQFPNY	
Yfr1	-LMAAGGSIIP----LTMESASVTLPPY	
Yfr5	-LVTTGGALTP----LAMESAVVTLPAY	
Yps7	-LMAAGGVLKQ----TSKNGLEITLPEY	
Yfr2	-LIAAGGTLSK----GSSNSISITLPPY	
Yfr3	-IVAAGGVVTQ----TSSQNISIKLPPY	
Yps4	-LENAGGSTSP----LTQSSLSVTLPFY	
Yps2	-LIAAGGTLTP----LSTTQFNLVLPPY	
Esa2	----------------------------	
Plu1	-LFQKNGGMIN--I-ESTHGYTIQLPEY	
Eco1	-LRSQGGQIQHSGS-QSAQDYQAILPAY	
Pru1	-LVAAGGHIVQE-----NGKYSLVLPAY	
Pal3	-LVAAGGQVIDEK----NGAYSIILPTY	
Eta1	-LLAAGGKLVREN----EGSWSVILPEH	
Sgl1	-LLAAGGKIVQES----IDNYSIVLPDY	
Eco26	-FIANGGKIINEG----LHNYSIILPDY	

Pmi1	-LNAQGGQIKHHG----GTHYTVILPQY
Efe5	-FAAAGGKITGS-----GTSWQFTLPAY
Eco20	-LLAEGGKITGQ-----GSQWQVTLPAY
Eco25	-LEAAGGKVVTT-----GKDILVTLPAY
Eco15	-LEAAGGKVSTS-----GKDITVTLPGY
Eco6	-LQSAGGKVAVS-----GKDIQVTIPPY
Efe2	-LTAAGGVISTA-----DNQVTVTLPAY
Efe3	-LQSAGGVVNTE-----ANQVTVTLPEY
Pal2	-FLAVGGSISSD-----DQTATLTLPSY
Csu1	-FIEAGGEITLVSAGKSNAEYTVKLPPH
Eco3	SLTPGAQANSAEG-------WTLIMPDW
Cko1	SLTAGAKADSAEG-------WTIIMPAW
Sen1	SLTAGTDTRNTEG-------WTIIMPAW
Eca1	SLTSPANSTSSDG-------WSIIIPAW
Esp2	SLTSPANANSDEG-------WSVIMPAW
Esa3	SLTPPLDSTSADG-------WTVIMPAW
Kpn1	SLTPPVDASSPDG-------WSIIMPVW
Pan1	SLTSPPNNNAIDG-------WSVIVPPW
Eta2	SLTPPPDNSDPQG-------WSIIIPAW
Spr1	SLTPSQNSNDPHG-------WSLIVPQW
Yin1	SLLATAGATNPQG-------WQITLPAW
Yin2	LLVPTAGANNPQG-------WQITLPAW
Yfr4	ALLATAGVDNHQG-------WQITLPAW
Ymo2	QLLATAGSHNPQG-------WQITLPAW
Eco10	VMPVIKENTLTEGN---NNHWNLVLPAW
Eal1	VMPVIKENTLTEGN---NNRWNIVLPAW
Sty4	VMPVVKADALTEGN---NNSWNLVLPAW

Eco16	VMPVVKANALTEGN---NNRWNLVLPAW
Ahy1	-LSGLSPADTAGQ-----DKRALTLPSL
Sen2	--ELIAGLQLQGS-----LGSGLILPQL
Efe4	--ELIGGLQQQGN-----VNSGLRLPDL
Bpe1	TATADASGRFAYTSAGDQPSGVLALRAR
Bav2	QSRADASGVYRIESRKDQPSGPIRLKAV
Bbr1	VAQADGSGTYRATSARDMVGGPVRARAT
Bpa2	SVRANAAGGFEASSAGDMTSGLIRARAT
Bav1	WVRADASGRYTARSGPDMPSGTVRAQAR
Pma1	----------------------------
Pma3	----------------------------
Pma5	----------------------------
Pma4	----------------------------
Ssp1	----------------------------
Ssp2	----------------------------
Plu2	----------------------------
Cla1	YITDNSGKVYVQIIRSCTPKTDLNAVAG
Rba1	----------------------------
