# Supplementary material for: The Bacterial Intimins and Invasins: A Large and Novel Family of Secreted Proteins
Source: PLoS One. 2010 Dec 22;5(12):e14403. doi: 10.1371/journal.pone.0014403 (PMC3008723; doi:10.1371/journal.pone.0014403)
Supplement: Figure S4 — Multiple alignment of C-terminal passenger domains. (2.82 MB PDF) [file pone.0014403.s004.pdf]

CLUSTAL X (1.81.1-alpha) MULTIPLE SEQUENCE ALIGNMENT


```
   Eca1 --------DAREGATNRWRLSVVAEDKDGQRVSSNEITLTVV--------QPLVV-----------------------MP     41
   Esp2 --------DASEGATNRWKLSVVVEDKDGQRVSSNEITLSLS--------QPLVA-----------------------LP     41
   Cko1 --------DSSEGATNRWRLSVVVEDEQGQRVSSNEITLSLT--------EPFMA-----------------------MP     41
   Sen1 --------DNREDATNCWRLSVVVEDEKGQRVSSNEITLALT--------EPFIT-----------------------MP     41
   Eco3 --------QNGEGASNHWRLSVVVEDNQGQRVSSNEITLTLV--------EPFDA-----------------------LS     41
   Esa3 --------DNAPGASNSWRLSVTVEDEQGQRVTSNWITLKLS--------APVQT-----------------------LP     41
   Kpn1 --------NSEPGAANRWRLSVVVEDKQGQRVSSNEIALALT--------EPLVK-----------------------FT     41
   Pan1 --------DSTPGASNEYRLSVTVEDSKQQRVTSNWITLKVQ--------APLSMD--------EPAVEGA-----VQMP     51
   Eta2 --------DSQQGASNAWHLSVTLEDSQQQRVTSNWITLKLS--------PPIGL----------PGLD-----------     43
   Spr1 --------DDSPDAANRYHLSVTLEDDKQQLVTSNWIQLQVT--------PPLTVSSEI-----EQGLPPAKVLQPPTIP     59
  Eco25 ------RFTSTPETDNTWPIEVTAEDVKGNFSNREQSMVVVQAPTLSQKDSSVSLSSQT-----LSADSHSTATLTFIAH     69
  Eco15 ------RFTNTPETDNTWSIDVTAEDVKGNLSRHEQSMVVIQAPTLSQKDSLLSVNPLT-----VAADKKSTTTLTVTAH     69
   Yfr1 ---------QQVQTANSYQISAVAYDVRGNRSNTATTTLVVQESPQ-QISLTLSGGSNT-----AVADGNAPVSYIATVV     65
   Yfr5 ---------QPLQNINSYQVSAVAYDIRGNKSNTATTQIIVQESPH-QIALNVVGSSTS-----ATADGSALVTYRASVV     65
   Yps4 --------QHILRTSNTHTISAVAYDAQGNASNRAVTSIEVTRPET-MVISHLATTVDN-----ATANGIAANTVQATVT     66
   Efe2 ------RYTATPDTDNTYRVAVTAEDVKGNRSNREESTVVVQAPQLSAEDSEVTSDKPI-----LKPDGVDTAHLTFLAR     69
   Efe3 ------RYTATPQTDNVYRVAVTAEDEKGNRSNREEASVVVLAPQLSAQHSQVTSDKTT-----LKPDGTEKAVLTFRAQ     69
   Eta1 ----------KPGEANSWTITGVAVDKKGNVSTGADTQVTVAQAAIDASMSPVTPEKIT-----LPADGKTQQLLLLKIN     65
  Eco26 ----------RNQENNSYTIDLSAIDIKGHTSNRKTIKIDVLYMDIDPTISSLDPKNIS-----LPADGQSQKKLTLKIK     65
   Pru1 --------RNDAKSANNYTISAVAIDKKGNISPNTMLRVVVTQPAIYPIKSALTPRKID-----LIADGKNTKKLSLSIR     67
   Sgl1 --------NFDSANGNVYEISGVAIDTHGNVSKKAKTTLTVTQPAINTTTSEFTPVKST-----LPADTVAQQTLTLKVK     67
   Pmi1 --------QIGAIEKNSYIISAVAIDTHGNRSAPVQTTVIVDKSLINTRNSLFSPKQSQ-----LFANGEATQKLILSIV     67
   Ymo1 ----------IDGGDNAYVLAGRAVDKKGNYSVSNSTNIYVTGVNINSVKSTITLTPAT-----LPANATSRSTLQLKLN     65
   Yen2 ----------IDGAVNAYVLSGRAIDKKGNYSVSGSTNVYVTGVNINRINSTISLNPAT-----LPANGTSRSTIQLKLN     65
   Sen2 ----PLTATDGQEYSLYLTVTD----SRGTRVTSERIPVRVTQDETSFRSWIN----II-----NDDAQVEDGNFVISTP     63
   Efe4 ----SLDVTENKEYSLYLKVTD----SRGNSVTSERIPVTVSINPESFTPYLN----VL-----HDEVRREEGKFVIPSP     63
   Bpe1 VDEVVLGDLQVAVMALVPLPADRALEVRGKTEPKVDVKVSFSNGESVLAKADAKGLFTV-----RSTRKVTQGKVLVQAT     75
   Bav2 EDGLVLGGLEVKILVMQTLPANGKLKVRGKTEPKMDVRVTFPDGEVVQLRSADDGSFTA-----LSKQRVPRGVVQVRVT     75
   Bpa2 TDTVDKTAPVLSIAGVATDEATGRVTVTGRSEPDAQVTIRFPGGGRKTVRADANGAYRV-----RSDGDLPAGVIVVQAA     75
   Bav1 TANPQVEMTAAPRIERVETLRNGLLRVSGRAAAGADIWVVFPDDEEVNGKAEADGSFIL-----SSTRPHASGDILVTAS     75
   Yin1 --------DSAPGATNLYTLSISIVDEQGRQATSNPVDIKVG---QQRSGRLMLESAAD-----VPASGRDSDAIKVAGY     64
   Yfr4 --------NPAPAATNRYQLALTVVDERGRQATSNPVDIMVG---QQRLGQLVLESAGS-----VPASGLATDAIVLAAH     64
   Yin2 --------DATPGATNLYTLSISIVDEKGHQATSNDVEIRVG---QQRLGRLFIDGDSA-----MPATGLDADAVKLSAH     64
   Ymo2 --------SSVAGASNRYQLALSLEDQKNHRVTTNTIEIQVG---QRRQGRLQVEGSNA-----VTASGHDSDVIRLVSY     64
  Eco10 ---QYSSDQAEQEKLNTWRVRLALEDEKGNRQNSGVVEITVQ---QDRKIELIVNNIAN-----VPEENNHS---HEASA     66
   Eal1 ---QYASTEAEQQKLNTWRVRIALEDEKGNRQNSGVVEITVN---QDRKIELIVNNIAN-----VPEENNHS---HEASA     66
  Eco16 ---QSASTKEEQEKVNTWRVRLALEDEKGNRQNSGVVEIVVK---QDRKIELIVNHVLS-----VPADNNHS---YQAEA     66
   Sty4 ---VNADTEAQRTALNTWKVRMTLEDEKGNKQNSGVVEITVQ---QDRKIELIVDNIAD-----N-DRSDHS---HEASA     65
   Yfr2 -------QASVGNTPNSYTLSAIAFDTQGNRSNSSSTLINVSPQNLSTGNSLMTATPSI-----LVADGVSTSQITITLR     68
   Ype5 --------LSGGEGNNTYRISAIAYDTLGNASPVAYSDLVVDSHGVNTNASGLTAAPEI-----LPANASASSVIEFNIK     67
   Eco6 ------RFTAMPETDNTYPIAVTAEDSKGNFSRREESMVVVEKPTLSLTDSTLSVDQQI-----LLADGKSTSTLTYTAR     69
   Bbr1 VDVAVKGEVPLTLGAVRTHPGTGVVTVTGKTGPGAKVRIDFPDGTFGDVVAGNGGDFTV-----ASKGDVTASGPIVAIA     75
   Yps2 KRTAQVSRVTDDLTANFYSLSALAVDHQGNRSNSFTLSVTVQQPQLTLTAAVIGDG--------APANGKTAITVEFTVA     72
  Eco14 -----------SSDRQSVTLSGRARDTRGNLSDIARTRILVSP--AVQQQLAVSTDKTT-----ATADGADSVRYTLTVT     62
   Sen3 -------------LTSSEIMSTTVSAQLDNGQKLDSETLQFTADSSSATVTGVEVDKKV-----ARADGQDTITVQATVM     62
   Pal3 --------KSGGKVQNRYTINAVAVDSKGNISPNAILQVQVTQPTIFSANSPLTPTINL-----VTNGTPAFTG--IKDN     65
   Ybe1 -----------PSGGNNWTISAIASDVKGNTSNRAVTLVQLQDT-PATISGTWTPAAST-----KPADGNSAVLLTLTLK     63
   Pal2 ---QTSNAAKDVQRINRYRLRAIAYDIKGNVSPVAETLIEITHSGSISISPRDMKFHGK-----GLANGNDINGLTAIAR     72
   Eco1 ----------VQGGSNVYKVTARAYDRNGNSSNNVQLTITVLSNGQVVDQVGVTDFTAD-----KTSAKADGTEAITYTA     65
   Yps7 --------QMNRTGGNSYILNAIAYDTQGNASSQASMLITVNAQKINIANSTLVAAPVN-----IEANNIDTSVVTLTLK     67
   Yfr3 -----------TAGSNVYMLSAVAYDNQGNTSNHSTTQITVTQQSVSHLNSTLQVSPTE-----ISADGVAVSLITLNLK     64
  Eco20 ----------RPGKDNYYAISAVAYDNKGNASKRVQTEVVITGAGMSADRTALTLDGQSRI--QMLANGNEQRPLVLSLR     68
   Efe5 ----------QNGGVNTWTVGATARDSKGNASNHAVITISVTSSNVSAGDSSFTLDGNNNA--QISADGQSTYPVTLNLK     68
   Csu1 --KLRLSSQGRTVPTNEYTLQVFAVDTSGRRSKTGTLSITVSQDPQVSINRKYFLNGTPVT--DFTTPPVANDQDFVEIE     76
   Ahy1 -------PTYRIGQSNQYPVVAIVTDIDGHEAIAEGVVAVSEDSGLQPAIQLAEHFVQLLPGAHYQVDWGVVDPRQKPAK     73
   Plu1 ----------QPDGKNDYTITGTSKDDQLRVQIQAHVLQRNISLSVNTTDPLIADGNAK---YVYTATLLGADKKTPIEN     67
   Cla1 ------------EANGKFPISIIKLDFKIKADPEKIERFEESLITLAGGNLANNLKVVWR----LIGKGELIKTNTSDTT     64
   Pas1 ----------SRQQSNHITISAVAHDEQGNVSNSSEMGVLINVPVALSAPVIGPVEKDEAELLLLTGEGGEVPPFLNDRE     70
  ruler 1.......10........20........30........40........50........60........70........80
```

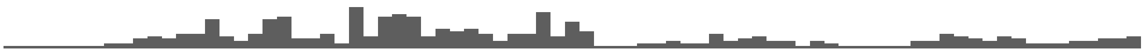

```
Eca1  DDDPRWKLLPDD--------------------------------------------------------------------  53
Esp2  DDDGGYQLLPNE--------------------------------------------------------------------  53
Cko1  DNDPRWKLLPEE--------------------------------------------------------------------  53
Sen1  DDNPPWQPFQEQ--------------------------------------------------------------------  53
Eco3  NDELRWEP------------------------------------------------------------------------  49
Esa3  QDDPRYELLAPVP-------------------------------------------------------------------  54
Kpn1  TPGVSWTDSP----------------------------------------------------------------------  51
Pan1  SSSNSLSFSE----------------------------------------------------------------------  61
Eta2  --SESLNFTDS---------------------------------------------------------------------  52
Spr1  AETGPLNGSD----------------------------------------------------------------------  69
Eco25 DAAG----NPVIGLVLSTRHE---------------GVQDITLSDWKDNGDG-SYTQILTTGAMSGTLTLMPQLNGVDAA  129
Eco15 DSDG----TPVPGLALQTRSE---------------GVQDITLSDWTDNGDG-SYTQILTAGTTSGSVTLTPQINGESAV  129
Yfr1  DTSGT-VATPLAGMNIAFDS----------------TVGNVLTPNAVTDNQGKANITIKSTLAGGGHIHGVLDNGNRAQA  128
Yfr5  DTANG-ANTPMAGMNVAFNA----------------TIGDVVTPVGTTDNMGRATVSVKSTLAGAGQVHAVLDNGNRAQA  128
Yps4  DGDG----QPIIGQIINFAVN---------------TQATLSTTEARTGANGIASTTLTHTVAGVSAVSATLGSSSRS-V  126
Efe2  DTDG--KAVSGLKVTAGFTAP---------------EGLNFSLSEFFTETKTPGEYTAGLKGSMKGTVSVMPLVDGKPAT  132
Efe3  DAEG--KAVSALTVSTSFTAP---------------QGMALVLSDTFTETETKGTYTAELKGTMPGEVRVMPQVAGKDAA  132
Eta1  DRDGKPVDIAESEISVLREAK--------------LRTAGSTTITAFSRSAAGEYVATLTAGTLPESFTLIPTARNVRFA  131
Eco26 NRNGLFEDINPDDINVLKTSE--------------ENFDVSSKITRFSRQEAGIYTATLTAGTKSERFTITPMIYNIKLP  131
Pru1  DKAGNYIDLAANEIGIEKVVANK----TAARSVNSANTANLTTVSGFTRIAAGQYEAILTSGTTPENFVLVSKARNAVFP  143
Sgl1  DIQGNPVDIGEDEITVTSNNA---------------QENSGAKVSALQRQDSGIYTLVVTAGTGTDVIKITPSARGANFA  132
Pmi1  DNDNLPVDIDSKEITLQQQSD---------------TEKGNSRISTFSRLAAGKYQLTVTAGSIPEKLTLTPVFRDNTFN  132
Ymo1  TDAGNAVSGAARQITFAVRDV-------SGNVPRARSFAQPVVIADAQEIQTGVYETSVTSGNLVGRFEITPTVRGVQLN  138
Yen2  TDAGQAVSGASGQMTFAIRDS-------SGRVFKARTSLQPVVISDVQEVQTGVYEASITSGFLTGRFEITPTVRGVQLN  138
Sen2  LPAGEEGKVIEWHYVRERSEE--------------EWASLKPRNIKYQSDTPGLSFKALGGTERDGHWVERVLVTHVGDD  129
Efe4  TVNDDNGSIIEWHYVRVRSKD--------------EWKSLKPENVEYSTHSPGLSFKSLGGEERDGQWIEKVQLVVKDPT  129
Bpe1  HPQ--TRKEARAVADYLPPAT--------------LAPTIDAVTAQADTGRVTVTGQAEPGAQVDVHFPDGTAKTVDAGA  139
Bav2  DPS--TDQEAVAQDMYEPPAA--------------VAPTIDKVTTDPATGRVTIVGEAEPRSRVEIRFSDGTTEVVDADD  139
Bpa2  DAAGNSTQAQQAYQDTVDRTA--------------PEPVQIALDTDVATGRLTVSGQAEPGGSVTVTWPDGTTQTVRLDA  141
Bav1  LPD--GGVSDASTYAYVKQPP--------------GEPTVDAVSADLVG-RLTIQGSSEAGAAIQANFPDGTDANTTADA  138
Yin1  LVNPADIGVTDSRLVPHWLLT--------------DKATGAAVPLVT-GSACPLDASGNP----EPCIRLKNTQVEVRDG  125
Yfr4  LEDHLGQLINDPQLQPVWQVI--------------DATSGTPVAWVAPGSSCPVDALGLP----QPCVQMVQVQTEVRSG  126
Yin2  LEDHLGKSINDAALAPVWVAR--------------SLYSGAVVPLVT-GPTCPTDEDGFP----DACLRVVHTATEVRDG  125
Ymo2  LEDHNGLAINDHDTKPLWLVK--------------SLAR-TPIELVN-ANQCPLNAAGKV----EPCLRIKDDRTEVRDG  124
Eco10 QADGVDGVVMDLDVTDSFGDN--------------TDRNGDALPEDNLTPQLYDAQDKRVTLTNKPCSTDNPCVFIAKQD  132
Eal1  QADGVDGVVMDLDVTDSFGDD--------------TDGKGNVLPEDNLNPQLYDAQDKKVTLANKPCTTEVPCVFIAEKN  132
Eco16 KADGSDGVVMDMHITDSFGDN--------------TDKNGNTLPETGVTPTLYDENDKKVTLNTKPCVTETPCVFIAKRD  132
Sty4  LADGEDGVVMDLLITDSFGDS--------------TDRNGNELVDDAMTPVLYDSNDKKVTLAQTPCTTETPCVFIASRD  131
Yfr2  DSHNQPVIGASRNIILASNFVVD----G-STSPIRAAAASEMKISDVSEAMGGVYNATLTAGTQPGTATITSSINGMALN  143
Ype5  DNANQPITGIADELAFSLELVELPEELAKAKARSVPLKTVSHTLTKITESAPGIYQATLTSGSKPQLINITAQINGVPLA  147
Eco6  DSSGKPIPGMTLKTQVKGLQD--------------------FALSEWKDNGNGTYTQIVTAGKTSGALSLMPQFNGDDIA  129
Bbr1  RDDDGRESPRRTVQYDDRVNGGG------------SGAPTVVLHTDGTNGRVTVSGKGRPGDTIRVDFPDGTTKEVVAGP  143
Yps2  DFEGKPLAGQEVVITTNNGALPN--------KITEKTDANGVARIALTNTTDGVTVVTAEVEGQRQSVDTHFVKGTIAAD  144
Eco14 GSDGKPVSGQAVRWEHNGGTLNG----------ENTTNADGVATATLTSQTAGIIRVTATTRNQTAKAADVTFVAAMQGE  132
Sen3  DAEQHPLANQPVDWAIRSAAS-----------------STHLSDKQSNTNENGVATITLTSAKAGQGIVTASTGNSEPVE  125
Pal3  KPASKLLKMADPYIHNNHHKI-----------------------------------------------------------  86
Ybe1  DAAGNPLIGQANNITLKKNTLG--------------GSGSEPTLSALTETGPGIYQTTATAGTNFGVLTLTPEIQGTQLA  129
Pal2  DSLGHAIPNTKVVFVLPNALT----------------LASRKTKTTTNSSVQNSLRKKMQTMKVAKPWEYITTTNDKGEA  136
Eco1  TVKKNGVAQANVPVSFNIVSG--------------TATLGANSAKTDANGKATVTLKSSTPGQVVVSAKTAEMTSALNAS  131
Yps7  DDNNIPVPGQNVTFLS-----------------------PLGTLSAMTDSGNGVYTATLTAGTVSGTTAVSSNINGIALD  124
Yfr3  DDNNLPVKGMGDQLTLDLHFTP---------DTTATQPVSYPLLSTVTETAPGIYTYQLTAGFTQGEANIAPTIQNISLA  135
Eco20 DAEGQPVTGMKDQIKTELAFKPAG--NIVTRSLKATKSQAKPTLGEFTETEAGVYQSVFTTGTQSGEATITVSVDGMSKT  146
Efe5  DSNGKPLTGLENDIEMSLEFTPDN-------NTQRSRSVSGPQLGKVQEISAGVYRSMLTAGSQSGTARVTAKVLGKTFT  141
Csu1  FTVTGMYGPLIGKEVLVRYAQG-------------SEEKYEVLLTDSQGKIRTSHIKTTLASPVEVDIAVNTVVDAVSVP  143
Ahy1  ARNGIPIESIEGDVEADRDGYQYR-----------YRLTFQGPGQVLTGRELVAPGQPNPGTRYRLDVEVIFPSGHVARD  142
Plu1  AKLIWDTDKKDPGLKLKPDSEATDKNGQQTATLTSTTPLSDIQVSVRINGERVTTDKKVSFTESSSSYRVTGVTVGVDKD  147
Cla1  DNNGEAIIKYKADDTMKDKEE-------------------VKVIASVNGVEFKAPVQIMVFGNNDNDLSIDKEVIGNKEI  125
Pas1  DELLEKQLGQKRLEQERLEQE---------------RLERERLEQERLGQERLEQERLEQERLGRERLEQERLGQEWLEQ  135
ruler ........90.......100.......110.......120.......130.......140.......150.......160
```

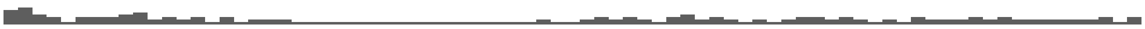

```
 Eca1 --------------------------------------------------------------------------------  53
 Esp2 --------------------------------------------------------------------------------  53
 Cko1 --------------------------------------------------------------------------------  53
 Sen1 --------------------------------------------------------------------------------  53
 Eco3 --------------------------------------------------------------------------------  49
 Esa3 --------------------------------------------------------------------------------  54
 Kpn1 --------------------------------------------------------------------------------  51
 Pan1 --------------------------------------------------------------------------------  61
 Eta2 --------------------------------------------------------------------------------  52
 Spr1 --------------------------------------------------------------------------------  69
Eco25 KAPAVVNIISVS-------------------------------------------------------------------- 141
Eco15 KESIVVNIVPVV-------------------------------------------------------------------- 141
 Yfr1 PLIFIADSSTAE-------------------------------------------------------------------- 140
 Yfr5 AVMFMADESTAS-------------------------------------------------------------------- 140
 Yps4 NTTFVADESTAE-------------------------------------------------------------------- 138
 Efe2 KAPVTVTLSDVV-------------------------------------------------------------------- 144
 Efe3 KDAVTVTLVNTT-------------------------------------------------------------------- 144
 Eta1 PISVELT------------------------------------------------------------------------- 138
Eco26 PSTVTISKD----------------------------------------------------------------------- 140
 Pru1 EIKVSVLADGATTQIAKLMTNISQSQPADGKTPLEIKTQLKDANGNPLINTRVTWSSDKNPNKVSFSSNTTSTDQNGFAS 223
 Sgl1 SASVTVEADG---------------------------------------------------------------------- 142
 Pmi1 SATVTLIADN---------------------------------------------------------------------- 142
 Ymo1 PIILTQSADATT-------------------------------------------------------------------- 150
 Yen2 PIILTQSADKTT-------------------------------------------------------------------- 150
 Sen2 ARS----------------------------------------------------------------------------- 132
 Efe4 ARMTLAA------------------------------------------------------------------------- 136
 Bpe1 DGAYAATSDGDMV------------------------------------------------------------------- 152
 Bav2 EGHYSATSAGDLP------------------------------------------------------------------- 152
 Bpa2 DGGFRVTSAGDAA------------------------------------------------------------------- 154
 Bav1 QGRYTLHSPGAVLQ------------------------------------------------------------------ 152
 Yin1 VNYYVLELVSTV-------------------------------------------------------------------- 137
 Yfr4 INHYVAELVSTQ-------------------------------------------------------------------- 138
 Yin2 ITYYVNSLISNQ-------------------------------------------------------------------- 137
 Ymo2 VNYHILELVSTL-------------------------------------------------------------------- 136
Eco10 KEKGTVTLSSTL-------------------------------------------------------------------- 144
 Eal1 KEKGTVTLASTL-------------------------------------------------------------------- 144
Eco16 KEKGTITLSSTL-------------------------------------------------------------------- 144
 Sty4 KEAGTVTLSSTL-------------------------------------------------------------------- 143
 Yfr2 AVNVIFTANAAT-------------------------------------------------------------------- 155
 Ype5 DVQTKVTLIADES------------------------------------------------------------------- 160
 Eco6 KTPALIAIVANT-------------------------------------------------------------------- 141
 Bbr1 DGTYRVTSDRDMT------------------------------------------------------------------- 156
 Yps2 KSTLAAVPTSIIADG----------------------------------------------------------------- 159
Eco14 LLADRTQALADG-------------------------------------------------------------------- 144
 Sen3 SETLTFMPDVKS-------------------------------------------------------------------- 137
 Pal3 --------------------------------------------------------------------------------  86
 Ybe1 PATITYTDPSST-------------------------------------------------------------------- 141
 Pal2 LVQFTSEIAGSYEISAYTG------------------------------------------------------------- 155
 Eco1 AVIFVDQTKASIT------------------------------------------------------------------- 144
 Yps7 MTPATVTLNGNSG------------------------------------------------------------------- 137
 Yfr3 SAKVILTASDNALS------------------------------------------------------------------ 149
Eco20 VTAELRATMMDVAN------------------------------------------------------------------ 160
 Efe5 LNIKQTVAEEGE-------------------------------------------------------------------- 153
 Csu1 FGPDTGTARVSG-------------------------------------------------------------------- 155
 Ahy1 SMEFEFIDDATVPG------------------------------------------------------------------ 156
 Plu1 KKRYNNGADSYT-------------------------------------------------------------------- 159
 Cla1 AIATYKNLRPNK-------------------------------------------------------------------- 137
 Pas1 ERLEQERLERER-------------------------------------------------------------------- 147
ruler .......170.......180.......190.......200.......210.......220.......230.......240
```

```
Eca1   --------------------------------------------------------------------------------    53
Esp2   --------------------------------------------------------------------------------    53
Cko1   --------------------------------------------------------------------------------    53
Sen1   --------------------------------------------------------------------------------    53
Eco3   --------------------------------------------------------------------------------    49
Esa3   --------------------------------------------------------------------------------    54
Kpn1   --------------------------------------------------------------------------------    51
Pan1   --------------------------------------------------------------------------------    61
Eta2   --------------------------------------------------------------------------------    52
Spr1   --------------------------------------------------------------------------------    69
Eco25  --------------------------------------------------------------------------------   141
Eco15  --------------------------------------------------------------------------------   141
Yfr1   --------------------------------------------------------------------------------   140
Yfr5   --------------------------------------------------------------------------------   140
Yps4   --------------------------------------------------------------------------------   138
Efe2   --------------------------------------------------------------------------------   144
Efe3   --------------------------------------------------------------------------------   144
Eta1   --------------------------------------------------------------------------------   138
Eco26  --------------------------------------------------------------------------------   140
Pru1   TTVTSTLAGDVLITASAGLGESQDLTVTFIPDIKSAMIRPQNFHASQSHTLADGITPNTLTVTVTDASGNIIPNVEVQFA   303
Sgl1   --------------------------------------------------------------------------------   142
Pmi1   --------------------------------------------------------------------------------   142
Ymo1   --------------------------------------------------------------------------------   150
Yen2   --------------------------------------------------------------------------------   150
Sen2   --------------------------------------------------------------------------------   132
Efe4   --------------------------------------------------------------------------------   136
Bpe1   --------------------------------------------------------------------------------   152
Bav2   --------------------------------------------------------------------------------   152
Bpa2   --------------------------------------------------------------------------------   154
Bav1   --------------------------------------------------------------------------------   152
Yin1   --------------------------------------------------------------------------------   137
Yfr4   --------------------------------------------------------------------------------   138
Yin2   --------------------------------------------------------------------------------   137
Ymo2   --------------------------------------------------------------------------------   136
Eco10  --------------------------------------------------------------------------------   144
Eal1   --------------------------------------------------------------------------------   144
Eco16  --------------------------------------------------------------------------------   144
Sty4   --------------------------------------------------------------------------------   143
Yfr2   --------------------------------------------------------------------------------   155
Ype5   --------------------------------------------------------------------------------   160
Eco6   --------------------------------------------------------------------------------   141
Bbr1   --------------------------------------------------------------------------------   156
Yps2   --------------------------------------------------------------------------------   159
Eco14  --------------------------------------------------------------------------------   144
Sen3   --------------------------------------------------------------------------------   137
Pal3   --------------------------------------------------------------------------------    86
Ybe1   --------------------------------------------------------------------------------   141
Pal2   --------------------------------------------------------------------------------   155
Eco1   --------------------------------------------------------------------------------   144
Yps7   --------------------------------------------------------------------------------   137
Yfr3   --------------------------------------------------------------------------------   149
Eco20  --------------------------------------------------------------------------------   160
Efe5   --------------------------------------------------------------------------------   153
Csu1   --------------------------------------------------------------------------------   155
Ahy1   --------------------------------------------------------------------------------   156
Plu1   --------------------------------------------------------------------------------   159
Cla1   --------------------------------------------------------------------------------   137
Pas1   --------------------------------------------------------------------------------   147
ruler  .......250.......260.......270.......280.......290.......300.......310.......320
```

```
  Eca1 --------------------------------------------------------------------------------  53
  Esp2 --------------------------------------------------------------------------------  53
  Cko1 --------------------------------------------------------------------------------  53
  Sen1 --------------------------------------------------------------------------------  53
  Eco3 --------------------------------------------------------------------------------  49
  Esa3 --------------------------------------------------------------------------------  54
  Kpn1 --------------------------------------------------------------------------------  51
  Pan1 --------------------------------------------------------------------------------  61
  Eta2 --------------------------------------------------------------------------------  52
  Spr1 --------------------------------------------------------------------------------  69
 Eco25 -------------------------------------------------------------------------------- 141
 Eco15 -------------------------------------------------------------------------------- 141
  Yfr1 -------------------------------------------------------------------------------- 140
  Yfr5 -------------------------------------------------------------------------------- 140
  Yps4 -------------------------------------------------------------------------------- 138
  Efe2 -------------------------------------------------------------------------------- 144
  Efe3 -------------------------------------------------------------------------------- 144
  Eta1 -------------------------------------------------------------------------------- 138
 Eco26 -------------------------------------------------------------------------------- 140
  Pru1 TDKGQLTNDKVVTNHQGISETQITSTQSGSATITASVGNHTLNKTLLFSANGQTAKVDYVLPEPKASYIADGRTAVTYTA 383
  Sgl1 -------------------------------------------------------------------------------- 142
  Pmi1 -------------------------------------------------------------------------------- 142
  Ymo1 -------------------------------------------------------------------------------- 150
  Yen2 -------------------------------------------------------------------------------- 150
  Sen2 -------------------------------------------------------------------------------- 132
  Efe4 -------------------------------------------------------------------------------- 136
  Bpe1 -------------------------------------------------------------------------------- 152
  Bav2 -------------------------------------------------------------------------------- 152
  Bpa2 -------------------------------------------------------------------------------- 154
  Bav1 -------------------------------------------------------------------------------- 152
  Yin1 -------------------------------------------------------------------------------- 137
  Yfr4 -------------------------------------------------------------------------------- 138
  Yin2 -------------------------------------------------------------------------------- 137
  Ymo2 -------------------------------------------------------------------------------- 136
 Eco10 -------------------------------------------------------------------------------- 144
  Eal1 -------------------------------------------------------------------------------- 144
 Eco16 -------------------------------------------------------------------------------- 144
  Sty4 -------------------------------------------------------------------------------- 143
  Yfr2 -------------------------------------------------------------------------------- 155
  Ype5 -------------------------------------------------------------------------------- 160
  Eco6 -------------------------------------------------------------------------------- 141
  Bbr1 -------------------------------------------------------------------------------- 156
  Yps2 -------------------------------------------------------------------------------- 159
 Eco14 -------------------------------------------------------------------------------- 144
  Sen3 -------------------------------------------------------------------------------- 137
  Pal3 --------------------------------------------------------------------------------  86
  Ybe1 -------------------------------------------------------------------------------- 141
  Pal2 -------------------------------------------------------------------------------- 155
  Eco1 -------------------------------------------------------------------------------- 144
  Yps7 -------------------------------------------------------------------------------- 137
  Yfr3 -------------------------------------------------------------------------------- 149
 Eco20 -------------------------------------------------------------------------------- 160
  Efe5 -------------------------------------------------------------------------------- 153
  Csu1 -------------------------------------------------------------------------------- 155
  Ahy1 -------------------------------------------------------------------------------- 156
  Plu1 -------------------------------------------------------------------------------- 159
  Cla1 -------------------------------------------------------------------------------- 137
  Pas1 -------------------------------------------------------------------------------- 147
 ruler .......330.......340.......350.......360.......370.......380.......390.......400
```

```
Eca1   --------------------------------------------------------------------------------   53
Esp2   --------------------------------------------------------------------------------   53
Cko1   --------------------------------------------------------------------------------   53
Sen1   --------------------------------------------------------------------------------   53
Eco3   --------------------------------------------------------------------------------   49
Esa3   --------------------------------------------------------------------------------   54
Kpn1   --------------------------------------------------------------------------------   51
Pan1   --------------------------------------------------------------------------------   61
Eta2   --------------------------------------------------------------------------------   52
Spr1   --------------------------------------------------------------------------------   69
Eco25  --------------------------------------------------------------------------------  141
Eco15  --------------------------------------------------------------------------------  141
Yfr1   --------------------------------------------------------------------------------  140
Yfr5   --------------------------------------------------------------------------------  140
Yps4   --------------------------------------------------------------------------------  138
Efe2   --------------------------------------------------------------------------------  144
Efe3   --------------------------------------------------------------------------------  144
Eta1   --------------------------------------------------------------------------------  138
Eco26  --------------------------------------------------------------------------------  140
Pru1   KVLDTNNNPVSDVDVNWLSNLNGSELHFEKETSKTNAQGIATTSIISNKAGIVIVTASTNGNGQLAVPITFVADKNQAKV  463
Sgl1   --------------------------------------------------------------------------------  142
Pmi1   --------------------------------------------------------------------------------  142
Ymo1   --------------------------------------------------------------------------------  150
Yen2   --------------------------------------------------------------------------------  150
Sen2   --------------------------------------------------------------------------------  132
Efe4   --------------------------------------------------------------------------------  136
Bpe1   --------------------------------------------------------------------------------  152
Bav2   --------------------------------------------------------------------------------  152
Bpa2   --------------------------------------------------------------------------------  154
Bav1   --------------------------------------------------------------------------------  152
Yin1   --------------------------------------------------------------------------------  137
Yfr4   --------------------------------------------------------------------------------  138
Yin2   --------------------------------------------------------------------------------  137
Ymo2   --------------------------------------------------------------------------------  136
Eco10  --------------------------------------------------------------------------------  144
Eal1   --------------------------------------------------------------------------------  144
Eco16  --------------------------------------------------------------------------------  144
Sty4   --------------------------------------------------------------------------------  143
Yfr2   --------------------------------------------------------------------------------  155
Ype5   --------------------------------------------------------------------------------  160
Eco6   --------------------------------------------------------------------------------  141
Bbr1   --------------------------------------------------------------------------------  156
Yps2   --------------------------------------------------------------------------------  159
Eco14  --------------------------------------------------------------------------------  144
Sen3   --------------------------------------------------------------------------------  137
Pal3   --------------------------------------------------------------------------------   86
Ybe1   --------------------------------------------------------------------------------  141
Pal2   --------------------------------------------------------------------------------  155
Eco1   --------------------------------------------------------------------------------  144
Yps7   --------------------------------------------------------------------------------  137
Yfr3   --------------------------------------------------------------------------------  149
Eco20  --------------------------------------------------------------------------------  160
Efe5   --------------------------------------------------------------------------------  153
Csu1   --------------------------------------------------------------------------------  155
Ahy1   --------------------------------------------------------------------------------  156
Plu1   --------------------------------------------------------------------------------  159
Cla1   --------------------------------------------------------------------------------  137
Pas1   --------------------------------------------------------------------------------  147
ruler  .......410.......420.......430.......440.......450.......460.......470.......480
```

```
Eca1   --------------------------------------------------------------------------------   53
Esp2   --------------------------------------------------------------------------------   53
Cko1   --------------------------------------------------------------------------------   53
Sen1   --------------------------------------------------------------------------------   53
Eco3   --------------------------------------------------------------------------------   49
Esa3   --------------------------------------------------------------------------------   54
Kpn1   --------------------------------------------------------------------------------   51
Pan1   --------------------------------------------------------------------------------   61
Eta2   --------------------------------------------------------------------------------   52
Spr1   --------------------------------------------------------------------------------   69
Eco25  --------------------------------------------------------------------------------  141
Eco15  --------------------------------------------------------------------------------  141
Yfr1   --------------------------------------------------------------------------------  140
Yfr5   --------------------------------------------------------------------------------  140
Yps4   --------------------------------------------------------------------------------  138
Efe2   --------------------------------------------------------------------------------  144
Efe3   --------------------------------------------------------------------------------  144
Eta1   --------------------------------------------------------------------------------  138
Eco26  --------------------------------------------------------------------------------  140
Pru1   ATLAINKATIIANGNDKALLDVIVTDNFGNPVEGIDVLLQANNGATITTLTPFSKTTVDGRVKAELATNQATGDIMVAAN  543
Sgl1   --------------------------------------------------------------------------------  142
Pmi1   --------------------------------------------------------------------------------  142
Ymo1   --------------------------------------------------------------------------------  150
Yen2   --------------------------------------------------------------------------------  150
Sen2   --------------------------------------------------------------------------------  132
Efe4   --------------------------------------------------------------------------------  136
Bpe1   --------------------------------------------------------------------------------  152
Bav2   --------------------------------------------------------------------------------  152
Bpa2   --------------------------------------------------------------------------------  154
Bav1   --------------------------------------------------------------------------------  152
Yin1   --------------------------------------------------------------------------------  137
Yfr4   --------------------------------------------------------------------------------  138
Yin2   --------------------------------------------------------------------------------  137
Ymo2   --------------------------------------------------------------------------------  136
Eco10  --------------------------------------------------------------------------------  144
Eal1   --------------------------------------------------------------------------------  144
Eco16  --------------------------------------------------------------------------------  144
Sty4   --------------------------------------------------------------------------------  143
Yfr2   --------------------------------------------------------------------------------  155
Ype5   --------------------------------------------------------------------------------  160
Eco6   --------------------------------------------------------------------------------  141
Bbr1   --------------------------------------------------------------------------------  156
Yps2   --------------------------------------------------------------------------------  159
Eco14  --------------------------------------------------------------------------------  144
Sen3   --------------------------------------------------------------------------------  137
Pal3   --------------------------------------------------------------------------------   86
Ybe1   --------------------------------------------------------------------------------  141
Pal2   --------------------------------------------------------------------------------  155
Eco1   --------------------------------------------------------------------------------  144
Yps7   --------------------------------------------------------------------------------  137
Yfr3   --------------------------------------------------------------------------------  149
Eco20  --------------------------------------------------------------------------------  160
Efe5   --------------------------------------------------------------------------------  153
Csu1   --------------------------------------------------------------------------------  155
Ahy1   --------------------------------------------------------------------------------  156
Plu1   --------------------------------------------------------------------------------  159
Cla1   --------------------------------------------------------------------------------  137
Pas1   --------------------------------------------------------------------------------  147
ruler  .......490.......500.......510.......520.......530.......540.......550.......560
```

```
 Eca1  --------------------------------------------------------------------------------   53
 Esp2  --------------------------------------------------------------------------------   53
 Cko1  --------------------------------------------------------------------------------   53
 Sen1  --------------------------------------------------------------------------------   53
 Eco3  --------------------------------------------------------------------------------   49
 Esa3  --------------------------------------------------------------------------------   54
 Kpn1  --------------------------------------------------------------------------------   51
 Pan1  --------------------------------------------------------------------------------   61
 Eta2  --------------------------------------------------------------------------------   52
 Spr1  --------------------------------------------------------------------------------   69
Eco25  --------------------------------------------------------------------------------  141
Eco15  --------------------------------------------------------------------------------  141
 Yfr1  --------------------------------------------------------------------------------  140
 Yfr5  --------------------------------------------------------------------------------  140
 Yps4  --------------------------------------------------------------------------------  138
 Efe2  --------------------------------------------------------------------------------  144
 Efe3  --------------------------------------------------------------------------------  144
 Eta1  --------------------------------------------------------------------------------  138
Eco26  --------------------------------------------------------------------------------  140
 Pru1  VVGSQSQPVAKHIKALADNNTAKVTIVSSTDRVQISQQTPTVILTATVIDDQQNPLIGTPVTWLTNHNRLSTNTTVTDLR  623
 Sgl1  --------------------------------------------------------------------------------  142
 Pmi1  --------------------------------------------------------------------------------  142
 Ymo1  --------------------------------------------------------------------------------  150
 Yen2  --------------------------------------------------------------------------------  150
 Sen2  --------------------------------------------------------------------------------  132
 Efe4  --------------------------------------------------------------------------------  136
 Bpe1  --------------------------------------------------------------------------------  152
 Bav2  --------------------------------------------------------------------------------  152
 Bpa2  --------------------------------------------------------------------------------  154
 Bav1  --------------------------------------------------------------------------------  152
 Yin1  --------------------------------------------------------------------------------  137
 Yfr4  --------------------------------------------------------------------------------  138
 Yin2  --------------------------------------------------------------------------------  137
 Ymo2  --------------------------------------------------------------------------------  136
Eco10  --------------------------------------------------------------------------------  144
 Eal1  --------------------------------------------------------------------------------  144
Eco16  --------------------------------------------------------------------------------  144
 Sty4  --------------------------------------------------------------------------------  143
 Yfr2  --------------------------------------------------------------------------------  155
 Ype5  --------------------------------------------------------------------------------  160
 Eco6  --------------------------------------------------------------------------------  141
 Bbr1  --------------------------------------------------------------------------------  156
 Yps2  --------------------------------------------------------------------------------  159
Eco14  --------------------------------------------------------------------------------  144
 Sen3  --------------------------------------------------------------------------------  137
 Pal3  --------------------------------------------------------------------------------   86
 Ybe1  --------------------------------------------------------------------------------  141
 Pal2  --------------------------------------------------------------------------------  155
 Eco1  --------------------------------------------------------------------------------  144
 Yps7  --------------------------------------------------------------------------------  137
 Yfr3  --------------------------------------------------------------------------------  149
Eco20  --------------------------------------------------------------------------------  160
 Efe5  --------------------------------------------------------------------------------  153
 Csu1  --------------------------------------------------------------------------------  155
 Ahy1  --------------------------------------------------------------------------------  156
 Plu1  --------------------------------------------------------------------------------  159
 Cla1  --------------------------------------------------------------------------------  137
 Pas1  --------------------------------------------------------------------------------  147
ruler  .......570.......580.......590.......600.......610.......620.......630.......640
```

```
  Eca1 --------------------------------------------------------------------------------  53
  Esp2 --------------------------------------------------------------------------------  53
  Cko1 --------------------------------------------------------------------------------  53
  Sen1 --------------------------------------------------------------------------------  53
  Eco3 --------------------------------------------------------------------------------  49
  Esa3 --------------------------------------------------------------------------------  54
  Kpn1 --------------------------------------------------------------------------------  51
  Pan1 --------------------------------------------------------------------------------  61
  Eta2 --------------------------------------------------------------------------------  52
  Spr1 --------------------------------------------------------------------------------  69
 Eco25 -------------------------------------------------------------------------------- 141
 Eco15 -------------------------------------------------------------------------------- 141
  Yfr1 -------------------------------------------------------------------------------- 140
  Yfr5 -------------------------------------------------------------------------------- 140
  Yps4 -------------------------------------------------------------------------------- 138
  Efe2 -------------------------------------------------------------------------------- 144
  Efe3 -------------------------------------------------------------------------------- 144
  Eta1 -------------------------------------------------------------------------------- 138
 Eco26 -------------------------------------------------------------------------------- 140
  Pru1 GQAKVELSGYISGETQVTAQLMNRQTAHQNIQFMADIPHQQNSVLEIKPQTIVANNHEQATATLILRDQWNNPVLGQNIQ 703
  Sgl1 -------------------------------------------------------------------------------- 142
  Pmi1 -------------------------------------------------------------------------------- 142
  Ymo1 -------------------------------------------------------------------------------- 150
  Yen2 -------------------------------------------------------------------------------- 150
  Sen2 -------------------------------------------------------------------------------- 132
  Efe4 -------------------------------------------------------------------------------- 136
  Bpe1 -------------------------------------------------------------------------------- 152
  Bav2 -------------------------------------------------------------------------------- 152
  Bpa2 -------------------------------------------------------------------------------- 154
  Bav1 -------------------------------------------------------------------------------- 152
  Yin1 -------------------------------------------------------------------------------- 137
  Yfr4 -------------------------------------------------------------------------------- 138
  Yin2 -------------------------------------------------------------------------------- 137
  Ymo2 -------------------------------------------------------------------------------- 136
 Eco10 -------------------------------------------------------------------------------- 144
  Eal1 -------------------------------------------------------------------------------- 144
 Eco16 -------------------------------------------------------------------------------- 144
  Sty4 -------------------------------------------------------------------------------- 143
  Yfr2 -------------------------------------------------------------------------------- 155
  Ype5 -------------------------------------------------------------------------------- 160
  Eco6 -------------------------------------------------------------------------------- 141
  Bbr1 -------------------------------------------------------------------------------- 156
  Yps2 -------------------------------------------------------------------------------- 159
 Eco14 -------------------------------------------------------------------------------- 144
  Sen3 -------------------------------------------------------------------------------- 137
  Pal3 --------------------------------------------------------------------------------  86
  Ybe1 -------------------------------------------------------------------------------- 141
  Pal2 -------------------------------------------------------------------------------- 155
  Eco1 -------------------------------------------------------------------------------- 144
  Yps7 -------------------------------------------------------------------------------- 137
  Yfr3 -------------------------------------------------------------------------------- 149
 Eco20 -------------------------------------------------------------------------------- 160
  Efe5 -------------------------------------------------------------------------------- 153
  Csu1 -------------------------------------------------------------------------------- 155
  Ahy1 -------------------------------------------------------------------------------- 156
  Plu1 -------------------------------------------------------------------------------- 159
  Cla1 -------------------------------------------------------------------------------- 137
  Pas1 -------------------------------------------------------------------------------- 147
 ruler .......650.......660.......670.......680.......690.......700.......710.......720
```

```
  Eca1 --------------------------------------------------------------------------------   53
  Esp2 --------------------------------------------------------------------------------   53
  Cko1 --------------------------------------------------------------------------------   53
  Sen1 --------------------------------------------------------------------------------   53
  Eco3 --------------------------------------------------------------------------------   49
  Esa3 --------------------------------------------------------------------------------   54
  Kpn1 --------------------------------------------------------------------------------   51
  Pan1 --------------------------------------------------------------------------------   61
  Eta2 --------------------------------------------------------------------------------   52
  Spr1 --------------------------------------------------------------------------------   69
 Eco25 --------------------------------------------------------------------------------  141
 Eco15 --------------------------------------------------------------------------------  141
  Yfr1 --------------------------------------------------------------------------------  140
  Yfr5 --------------------------------------------------------------------------------  140
  Yps4 --------------------------------------------------------------------------------  138
  Efe2 --------------------------------------------------------------------------------  144
  Efe3 --------------------------------------------------------------------------------  144
  Eta1 --------------------------------------------------------------------------------  138
 Eco26 --------------------------------------------------------------------------------  140
  Pru1 WSKNNTGLSLSGARELPNNGEYQVNISGNLAGTFDISAQTGSVTSQKMIGLIADSSTAHLKNINIVGKTTAPADGTNPIT  783
  Sgl1 --------------------------------------------------------------------------------  142
  Pmi1 --------------------------------------------------------------------------------  142
  Ymo1 --------------------------------------------------------------------------------  150
  Yen2 --------------------------------------------------------------------------------  150
  Sen2 --------------------------------------------------------------------------------  132
  Efe4 --------------------------------------------------------------------------------  136
  Bpe1 --------------------------------------------------------------------------------  152
  Bav2 --------------------------------------------------------------------------------  152
  Bpa2 --------------------------------------------------------------------------------  154
  Bav1 --------------------------------------------------------------------------------  152
  Yin1 --------------------------------------------------------------------------------  137
  Yfr4 --------------------------------------------------------------------------------  138
  Yin2 --------------------------------------------------------------------------------  137
  Ymo2 --------------------------------------------------------------------------------  136
 Eco10 --------------------------------------------------------------------------------  144
  Eal1 --------------------------------------------------------------------------------  144
 Eco16 --------------------------------------------------------------------------------  144
  Sty4 --------------------------------------------------------------------------------  143
  Yfr2 --------------------------------------------------------------------------------  155
  Ype5 --------------------------------------------------------------------------------  160
  Eco6 --------------------------------------------------------------------------------  141
  Bbr1 --------------------------------------------------------------------------------  156
  Yps2 --------------------------------------------------------------------------------  159
 Eco14 --------------------------------------------------------------------------------  144
  Sen3 --------------------------------------------------------------------------------  137
  Pal3 --------------------------------------------------------------------------------   86
  Ybe1 --------------------------------------------------------------------------------  141
  Pal2 --------------------------------------------------------------------------------  155
  Eco1 --------------------------------------------------------------------------------  144
  Yps7 --------------------------------------------------------------------------------  137
  Yfr3 --------------------------------------------------------------------------------  149
 Eco20 --------------------------------------------------------------------------------  160
  Efe5 --------------------------------------------------------------------------------  153
  Csu1 --------------------------------------------------------------------------------  155
  Ahy1 --------------------------------------------------------------------------------  156
  Plu1 --------------------------------------------------------------------------------  159
  Cla1 --------------------------------------------------------------------------------  137
  Pas1 --------------------------------------------------------------------------------  147
 ruler .......730.......740.......750.......760.......770.......780.......790.......800
```

```
 Eca1 --------------------------------------------------------------------------------   53
 Esp2 --------------------------------------------------------------------------------   53
 Cko1 --------------------------------------------------------------------------------   53
 Sen1 --------------------------------------------------------------------------------   53
 Eco3 --------------------------------------------------------------------------------   49
 Esa3 --------------------------------------------------------------------------------   54
 Kpn1 --------------------------------------------------------------------------------   51
 Pan1 --------------------------------------------------------------------------------   61
 Eta2 --------------------------------------------------------------------------------   52
 Spr1 --------------------------------------------------------------------------------   69
Eco25 ----------------------------------------------------------------------------SSRT  145
Eco15 ----------------------------------------------------------------------------SSRD  145
 Yfr1 ----------------------------------------------------------------------------IVSG  144
 Yfr5 ----------------------------------------------------------------------------ILSD  144
 Yps4 ----------------------------------------------------------------------------ITAA  142
 Efe2 ----------------------------------------------------------------------------PVAK  148
 Efe3 ----------------------------------------------------------------------------PVSE  148
 Eta1 --------------------------------------------------------------------------------  138
Eco26 -------------------------------------------------------------------------------R  141
 Pru1 LRATVTDATNNPAPAGIAVGWRSDIGELSQPVSLTDNNGIAQITLTSTVAGKGKVAAIVGQSTKETTNQIEFLAGTVSRS  863
 Sgl1 ----------------------------------------------------------------------------TTAQ  146
 Pmi1 ----------------------------------------------------------------------------QTAH  146
 Ymo1 ---------------------------------------------------------------------------ATING  155
 Yen2 ---------------------------------------------------------------------------ATITD  155
 Sen2 -----------------------------------------------------------------------------FKL  135
 Efe4 -----------------------------------------------------------------------------MEL  139
 Bpe1 ----------------------------------------------------------------------------SGDI  156
 Bav2 ----------------------------------------------------------------------------SGEL  156
 Bpa2 ----------------------------------------------------------------------------QGDV  158
 Bav1 ----------------------------------------------------------------------------SGDI  156
 Yin1 ------------------------------------------------------------------------------MG  139
 Yfr4 ------------------------------------------------------------------------------LG  140
 Yin2 ------------------------------------------------------------------------------PG  139
 Ymo2 ------------------------------------------------------------------------------AG  138
Eco10 ------------------------------------------------------------------------------PG  146
 Eal1 ------------------------------------------------------------------------------PG  146
Eco16 ------------------------------------------------------------------------------PG  146
 Sty4 ------------------------------------------------------------------------------PG  145
 Yfr2 ----------------------------------------------------------------------------FVVD  159
 Ype5 -----------------------------------------------------------------------------TAT  163
 Eco6 ------------------------------------------------------------------------------AS  143
 Bbr1 ----------------------------------------------------------------------------AGDI  160
 Yps2 ----------------------------------------------------------------------------LMAS  163
Eco14 ------------------------------------------------------------------------------QE  146
 Sen3 ----------------------------------------------------------------------------ATLS  141
 Pal3 --------------------------------------------------------------------------------   86
 Ybe1 -----------------------------------------------------------------------------KPS  144
 Pal2 --------------------------------------------------------------------NHQPAKAQVVFK  167
 Eco1 ---------------------------------------------------------------------------EIKAD  149
 Yps7 -----------------------------------------------------------------------------NLS  140
 Yfr3 ----------------------------------------------------------------------------PENS  153
Eco20 -----------------------------------------------------------------------------STL  163
 Efe5 ---------------------------------------------------------------------------TETDA  158
 Csu1 ----------------------------------------------------------------------------TINI  159
 Ahy1 ------------------------------------------------------------------------------AP  158
 Plu1 -----------------------------------------------------------------------------FTA  162
 Cla1 -----------------------------------------------------------------------------TNV  140
 Pas1 ------------------------------------------------------------------------------LE  149
ruler .......810.......820.......830.......840.......850.......860.......870.......880
```

CLUSTAL X (1.81.1-alpha) MULTIPLE SEQUENCE ALIGNMENT

File: /Users/saierlab/Desktop/58cterm.ps Date: Tue May 12 18:49:09 2009


```
Eca1   --------------------------------------------------------------------------------   53
Esp2   --------------------------------------------------------------------------------   53
Cko1   --------------------------------------------------------------------------------   53
Sen1   --------------------------------------------------------------------------------   53
Eco3   --------------------------------------------------------------------------------   49
Esa3   --------------------------------------------------------------------------------   54
Kpn1   --------------------------------------------------------------------------------   51
Pan1   --------------------------------------------------------------------------------   61
Eta2   --------------------------------------------------------------------------------   52
Spr1   --------------------------------------------------------------------------------   69
Eco25  HSSIKIDKDR---------YLSGNPIEVTVELRDENDKPVKEQKQQLNTA-VSIDNVKPGVTTDWKETADGVYKATYTAY  215
Eco15  HSSITIDNVS---------YYAGDDIKVRVELKDDSNQPVAYQKEELVKA-VTVENSKPGATIVWHEEQPGVYAANYPAY  215
Yfr1   NLTVTTDNAT---------ANGSDSNAVQVLVTDANGNPVSNEMVSFSVD-NGN-LANPNANTGPDGIATMTLTNTAAGT  213
Yfr5   NLTVLSNNAL---------ANGTATDSVQVMVTDANGNPVPNQLVNFNAD-NGATLSFSSGITDANGTIIVTAVNTTTGV  214
Yps4   NLTVTTNDSV---------ANGSDTNAVRAKVTDAYTNAVANQSVIFSAS-NGATVIDQTVITNAEGIADSTLTNTTAGV  212
Efe2   YSTITLSSAATRADQAGQNFHAGEQVFVTVMLKDTLQHPVSDQKALLQGEVVTVEGMSSKDGAEWQEEEDGTYKMLYVAQ  228
Efe3   HSSITLSPAVSRADRAGQAFRAGDAVTATVILRDEQQRPVIHQAALLTEESVTVSGMEPAAGAAWQEEDGGIYRMQYIAK  228
Eta1   --------------------------------------------------------------------------------  138
Eco26  QVFLSLSPSVI-------TSGVDSATLRVTIKNSAGNIYDGFQDKIKLQYDTDLIATNTAFREIAGGVYETFIQAKKAGT  214
Pru1   ASSVTLAVPSI-------IAATGETNITITLKDNTGNPLTGLANKIILDYSANLSIATPRFNEISKGVYRGKLSGVKAGS  936
Sgl1   IKA--LEVVSD-------NAAADGQATNQVKVTVIDANGNPIPNHVVSFSADNGATIIDNATTNANGAAVADLTSVKASE  217
Pmi1   IAKGNLTVTKD-------NEPADGKSQNKIKVRVTDSNHNPLAHYPVNFTASNGANVISSRKTDEQGEIIASVTNTQVGS  219
Ymo1   SANITISTPTI-------TANATDKTHLEVLVTDALGHPVPGVEVTWVSDLNSPGLEHVTSITNEHGIAENNFTSTTAGT  228
Yen2   SSAVTISTPSI-------TTNATDKTKLEVQVTDALGHPVPGVEVTWVSDLNSPGLEYVTSITNEHGIAENNFSSTVTGT  228
Sen2   HIEASGPDDKHP------------------------VKGSVLLQAQSDSIAQKVTSVEVLFTPGN---------------  176
Efe4   NISATGPGGTHP------------------------VNGTIRMTPVMN-LTDKVSSVQILFTAGT---------------  179
Bpe1   HAQATDKAGNQSPEATRHYGDTTDITPPAAPTIANVATDATSGRVTAAGMAEPGANVTVNFPDGTRKTVVAGGDGAYTAT  236
Bav2   RATRLPAAGEQPLSTRREYKDEVDKTAPEAPSITKVSTDATTGRVTVSGSAEPDVVVTVMFPDGTEKTVPTNDDGTYRAT  236
Bpa2   VVVVADQAGNRAAPVRAHYADPVDRTAPMTPTVR-HATDAQTGRVTVMGRTEAGALVTVQFPDGSSKTVRAQNDGGYAAT  237
Bav1   MITASGIDGAVGDAVFKPYTPEAPQATITS------VIPSTLGELTVSGLTQANAEVYVQFPDGSSTTVNADASGNYTAV  230
Yin1   TFSLQADFNHYG-----------RSNVQIVTFS---NPGGRDNVAR----AEIVDSAG---TDVLVSGNHPQVGVTYTVK  198
Yfr4   TFLVQADLGVYG-----------VTAPQTVSFT---SSG-PTLVTR----AEIQDPSG---VDLLTSGAHPQVGVVYTVK  198
Yin2   TFIITTDLGAYG-----------VTNAKTITFTS--ASPMETVVAR----AEIRDPAG---EDLLTTHNAPQVGVTYTVV  199
Ymo2   NFTISSDMGVYG-----------VSNSQTFVFNS--TISSIENLTGGIFLAKDNPTAGTGATDYAATGTPLKVGETYRFI  205
Eco10  TYRWKAKAAPYD-----------DSNYVDVTFLG-AEIGGLNAFIYRVGAAKPSNLIGK-------DKEPLPLNNTYRFV  207
Eal1   TFRWKAKAAPYD-----------DSNYVDVTFLG-SDIGGLNAFIYCTNDPKPHNLAGV-------D-ERIPTDSTYRFV  206
Eco16  TFRWKVKAEPYS-----------DSNFVDVTFTNTANVGGINGFIYRLEESSPKNLAG--------DAKPIPLKSGYRFV  207
Sty4   TFRWKAKEDAYG-----------DSNYVDVTFIG-DNLSALNAVIYQVKAANPVNLIGK-------EDKHPTVNNTYRFL  206
Yfr2   SLTADTHTTFAD---------GNSLITFTATVKDSSGHGVPNTDVILNTTGGVLSASSVTTDTHGVATFTLKSTNTGSFS  230
Ype5   LQTSSLQIITNG-------SLADDTDANQIRAVVVDAYGNKLSGVQVNFTVGNNAKITETTLSDKQGGVTAAITSTKAGT  236
Eco6   RADSTIETDQDN--------YVAGKPIVVKVTLRDDNGNGVTGRKELLKQTVKVDNTKADDVSAWTEESEGIYKASYTAH  215
Bbr1   TVSGTDAKGNVG----GPVKRPYHDIFVPVPPTVEVATDSSSGRVTVSGKATPRAKVKVDFPGGTSKTVTADADGRYRAT  236
Yps2   TITLELKDTYGD-----------PQAGANVAFDTTLGNMGVITDHNDGTYSAPLTSTTLGVATVTVKVDGAAFSVPSVTV  232
Eco14  AVSYTLTLKTTD---------GKPLSGKNVTFTTTVGQLSRTQGTTDQNGQLSVQLTSTRAGQAVVNASVDSTTISAAPV  217
Sen3   AITADKTAAQAN---------GADAVTLSVKVEDANHNPIPGAQISWTTTSATAILSASDTSTDAKGNASISVTSTTVEN  212
Pal3   --------------------------------------------------------------------------------   86
Ybe1   VSNLILLGELAE----------DSKLSATYTFTQPAGGPAATDASTYQWGEKGATAANVANGSAVATSGQVPEYLLKSTD  214
Pal2   PDSAQASIHSFTITRSGVLADGISTNSVKAHITDKNKNPLANQEVTFSATHAKIVDKATTNENGIVEVTLTSLKAGSSEV  247
Eco1   KTTAVANGKDAVTYTVKVMKNGLPEKGHVVTFSTDLGKLNLQTVATDKDGFASVTLTSDSVGKAVVSAKVSEAGSVVNAD  229
Yps7   TTHSTLVAAPVS-----IEANGSDTSLVTLTLRDSNNNPVTGQTVALVSTLGTLGAVTEQASGVYTATLTAGTVAGVASL  215
Yfr3   LFSVEPTEIIADG-----------VQTSVLSFTAQDIHHLPIKGLTVAFDVTGIPDVMLSAVTENNGVYAATLKGTVAGT  222
Eco20  SANEPSGDVVAD---------GQQAYTLTLTAVDSEGNPVTGEASRLRFVPQDTNGVTVGAISEIKPGVYSATVSSTRAG  234
Efe5   VLTAAPAEQVVG-----------YNINLQLVAKDAKGNAITGDKTLRFYALDQAEGVDFGAVTEKEGVYSATVTAKRAGK  227
Csu1   LKNNQPANNQDQAEVIFTIVDAHNNPIPNFAVTATASNQATLDTVPSTTDASGQIRVSLKNNRSGITEVTATSNNSSSTA  239
Ahy1   TLTAADSNGDDKPEVTGKAEPESTVTITWPDGSTSTTTADVDGNYTLEAPTVQGSGTITATATDKSGNTGPATSVNYIDS  238
Plu1   TVVDGHGKPVADKLIDIDWQTDSKVDGLKLTKQNNSVSNAQGQVTATLTSTAAVENVQVSAKTASQQTAVNANGKVSFTE  242
Cla1   KWRLDGEDKALFILENENKSSTAKELQLIADNKGESKVKIIGLGNNGKVKIFAKNINDESVSEKNKELEVKNYKATMILP  220
Pas1   QERLEQERLERERLDQERVEQEARDAWLRQEEQERLEQERLEQERLEQERLEQERLDQERVEQEARDAWLRQEEQERLEQ  229
ruler  .......890.......900.......910.......920.......930.......940.......950.......960
```

```
Eca1    --------------------------------------------------------------------------------   53
Esp2    --------------------------------------------------------------------------------   53
Cko1    --------------------------------------------------------------------------------   53
Sen1    --------------------------------------------------------------------------------   53
Eco3    --------------------------------------------------------------------------------   49
Esa3    --------------------------------------------------------------------------------   54
Kpn1    --------------------------------------------------------------------------------   51
Pan1    --------------------------------------------------------------------------------   61
Eta2    --------------------------------------------------------------------------------   52
Spr1    --------------------------------------------------------------------------------   69
Eco25   TKGSGLTAKLLMQNWNEDLH----------------------------------------------------TAGFIIDA  243
Eco15   KQGTALRAQLSLHNWNAPLQ----------------------------------------------------SHIYNIEA  243
Yfr1    TVVTATVNGSNVS-----ENTTFKA----------------------------------------------DATTAEIAD  242
Yfr5    TNITATLNGSSQS-----VPVNFIP----------------------------------------------DNGTATIAA  243
Yps4    SAVTATLGSQSQQ-----VDTTFKPGSTAAISLVKLADRAVADGIDQNEIQVVLRDGTGNAVPNVPMSIQADNGAIVVAS  287
Efe2    GAKEGHRATLKLTDGNK------------------------------------------------------TTAPYTIHP  254
Efe3    NARDAHKATLRLADGSK------------------------------------------------------STEPYAIHP  254
Eta1    -------------------------------------------------------------------------------A  139
Eco26   TTITVLIDDNPVP----------------------------------------------------------DSKLLTVKA  236
Pru1    TLIKVKANNVTLD----------------------------------------------------------NSVSLTITP  958
Sgl1    VTVTAALDNQVTK----------------------------------------------------------TVKTTFVSD  239
Pmi1    SQIGVQVKG-ITY----------------------------------------------------------STEVHFSVD  240
Ymo1    ANITVQTGTSPPV----------------------------------------------------------QAGTIEIKP  250
Yen2    ANITVQVGTSAPV----------------------------------------------------------QAGKIEIKA  250
Sen2    --------------------------------------------------------------------------------  176
Efe4    --------------------------------------------------------------------------------  179
Bpe1    SDRDMVSGDIRVQATDK-------------------------------------------------AGNQSPEATRAYAD  267
Bav2    SDGNMVSGDILAHATDR-------------------------------------------------AKNRSPDTRYAYAD  267
Bpa2    SDTDMVSGPIVVSAGDA-------------------------------------------------DGNRTPAQQVMYTD  268
Bav1    S-------------------------------------------------------------------------------  231
Yin1    LYNA----------------------------------------------------------------------------  202
Yfr4    LFDA----------------------------------------------------------------------------  202
Yin2    LFDE----------------------------------------------------------------------------  203
Ymo2    AWSDRQGNGKW---------------------------------------------------------------------  216
Eco10   LWRDNNKDGVFQQ-------------------------------------------------------------------  220
Eal1    LWRDKNKDGIFQQ-------------------------------------------------------------------  219
Eco16   MWRDSNNDGIYQQ-------------------------------------------------------------------  220
Sty4    LWRDKNKDGVFQM-------------------------------------------------------------------  219
Yfr2    VTAKSAVNTTDIG-------------------------------------------------------------------  243
Ype5    YTVTAELNGVTQQIDVN---------------------------------------------------FIPDAGTATLDD  265
Eco6    LIGDKLTAQLTMPG-----------------------------------------------------WQTKHSDAFSIAG  242
Bbr1    S-------------------------------------------------------------------------------  237
Yps2    NFTADPIPDAGRS-------------------------------------------------------------------  245
Eco14   TFENRLDSAIVVN-------------------------------------------------------------------  230
Sen3    VSVVATMKEQAQTS---------------------------------------------------------PDLQFTVDN  235
Pal3    --------------------------------------------------------------------------------   86
Ybe1    VGKVMEISVQAKN-------------------------------------------------------------------  227
Pal2    TISINGQSETKSVQFLSG--------------------------------------------------------------  265
Eco1    AVNFFATLSIDNN-------------------------------------------------------------------  242
Yps7    SVSVGGSALGVAP------------------------------------------------------------ATVTLNG  235
Yfr3    VTVAPTVNGSAIVG---------------------------------------------------------KSHEVILTA  245
Eco20   NVVVRAFSEQYQLG------------------------------------------------------------------  248
Efe5    IRIGVKSDSHDFSG---------------------------------------------------------IEKEISFTE  250
Csu1    KLEFLPVLKVDNAR------------------------------------------------------------------  253
Ahy1    TVPGAPTLAATDSN------------------------------------------------------------------  252
Plu1    FSTSYYVASVTMEVD-----------------------------------------------------------------  257
Cla1    THKHPITNTPFENG------------------------------------------------------------------  234
Pas1    ERLGRERLEQERLG------------------------------------------------------------------  243
ruler   .......970.......980.......990......1000......1010......1020......1030......1040
```

```
 Eca1 --------------------------------------------------------------------------------  53
 Esp2 --------------------------------------------------------------------------------  53
 Cko1 --------------------------------------------------------------------------------  53
 Sen1 --------------------------------------------------------------------------------  53
 Eco3 --------------------------------------------------------------------------------  49
 Esa3 --------------------------------------------------------------------------------  54
 Kpn1 --------------------------------------------------------------------------------  51
 Pan1 --------------------------------------------------------------------------------  61
 Eta2 --------------------------------------------------------------------------------  52
 Spr1 --------------------------------------------------------------------------------  69
Eco25 NPQS------------------------------------------AKIATLSASNNGVLANENAANTVSVNVADEGSNP 281
Eco15 NQNK------------------------------------------ARVATLSATNNDVYADKKTFNTLTINVTDESDNP 281
 Yfr1 T-------------------------------------------------DFTIASG-AVANGSATNALSATVKDAGGNT 272
 Yfr5 G-------------------------------------------------ALTVLTDNALANGSAPNSVKAVVTDAGGNP 274
 Yps4 TPNTGVDGTINATFTNLRAGESVVSVTSPALVGMTMTMTFSADQRTAVVSTLAAIDNNAKADGTDTNVVRAWVVDANGNS 367
 Efe2 GDVN------------------------------------------IDKSTLGSDKEEIAADGQEEAIITYNAQDSYGNA 292
 Efe3 GEAN------------------------------------------PTKSLLGTDKPAILANGDDTATLEFIARDAYDNI 292
 Eta1 NDNT-----------------------------------------IVVKDAISATPTSAVAG--DTVTYSAVLTDKQGNP 176
Eco26 DNNS-----------------------------------------ATVKGSISANPGVALVG--QYTTYKATLVDKNDNI 273
 Pru1 DSQT-----------------------------------------ARVRGGITASKTTETVG--RSVTYSAIFEDANSNL 995
 Sgl1 DKSA-----------------------------------------MVAELKVVSDG--ATADGKAENQLKVSVTDANGNV 276
 Pmi1 NDSA-----------------------------------------YIPQQNFTITPPLSLADGKTEKTISLQVVDKQNNP 279
 Ymo1 D------------------------------------------------------------------------------- 251
 Yen2 DNST-----------------------------------------MTVNASDFTVTTTPVVANGTSKAVYKLKVMDKQGN 289
 Sen2 --------------------------------------------------------------DEANGSVTAPVVGTEMRA 194
 Efe4 --------------------------------------------------------------EELNGSTNAPVVGSTLQA 197
 Bpe1 AVDRTAPEVPTVTHVATDAKSGRITVTGMAEPGANVTVNFPDGTRKTAVAGGDGAYTATSDGDMVSGDIRVQATDKAGNR 347
 Bav2 AV---APATPVIRRLTTNSTTGQVTAAGTAEPGNQVAVTFPDGTQKTVTADGEGHYTAESEGDQPSGDVKAQAVDAAGNK 344
 Bpa2 TLDKTAPASPSLT-VREDAASGRATVTGQAEPGAAVRVVFPNGEAQTVTAGSDGAYSVTSAADMVAGEITVVAADASGNQ 347
 Bav1 -----------------------------------------------------------TSKSMPSGEIMVIATGRSAGV 252
 Yin1 -------------------------------------------------------ANVDITSTLPADSLHWALDGAN--- 224
 Yfr4 -------------------------------------------------------ANTDITATIPATEVHWALDGTN--- 224
 Yin2 -------------------------------------------------------NDVDVTETLPADEVHWALDGTN--- 225
 Ymo2 -------------------------------------------------------SEGDEEVTSSLQSIQWYLDGTNSSA 241
Eco10 -------------------------------------------------------VEKLTDEEMVQYDYKWEFTGKS--- 242
 Eal1 -------------------------------------------------------SEKLTDEEMAQYDYQWEFTGQS--- 241
Eco16 -------------------------------------------------------SEKLTEEEKARYDYEWEFTGKS--- 242
 Sty4 -------------------------------------------------------SEQLTEEEMALYDYQWEFTGQS--- 241
 Yfr2 --------------------------------------------------QTQNATFIPVFTGIIAGGYDFALNDGFPKT 273
 Ype5 SDE----------------------------------------------YKLQWVTNGQVADGESTNSVQLTVVDKFGNT 299
 Eco6 DKDT-------------------------------------------AKIAAMQITANNAVARRDHNTVAVTVRDVHQNL 279
 Bbr1 ------------------------------------------------------------DGDVPGGDIVVTQTGMPGAA 257
 Yps2 --------------------------------------------------SFTVSTPDILADGTMSSTLSFVPVDKNGHF 275
Eco14 -------------------------------------------------------KTSAVADGQDSIILTAVIRDAAGVP 255
 Sen3 --------------------------------------------ATAAVESLNADKTQAVANQNDFITLTARVVDANDHP 271
 Pal3 --------------------------------------------------------------------------------  86
 Ybe1 --------------------------------------------------SLAVTGNTLTVDSASADTNTNKTTGGEQGK 257
 Pal2 ------------------------------------------------KLHQVTILDVPEVYAGRESQVSFQLLDSHGNP 297
 Eco1 ----------------------------------------------------VEIVGTKVRGELPNIWLRYGQVKLNANG 270
 Yps7 DSGN----------------------------------------LSTTHSTLVAAPVNIEANGSDTSLVTLTLRDSNNNP 275
 Yfr3 NHSTAN-------------------------------------------VDLTVIIDDAFANGVDVNKIKAHVIDLHGNP 282
Eco20 ----------------------------------------------------TLQQTLKFVAGPLDAAHSSIPLNPDKPV 276
 Efe5 DR------------------------------------------MQFAFSRFEASKNNALADGKQQNTVTVSLADRFGNV 288
 Csu1 ----------------------------------------------------VTVTAKSYTAGKNPVSVSSSFSGSPLWS 281
 Ahy1 ----------------------------------------------------SDNKPEVSGKAEPDSTVTITWPDGTTST 280
 Plu1 ------------------------------------------------------KDKVRYNNGSDSYTFTATVKDGHGNL 283
 Cla1 ------------------------------------------------TIDYVSEFEVKLTGLMPNTKVEWIAKDTQNNK 266
 Pas1 -------------------------------------------------------QERLEQERLEQERLERERLEQERLE 268
ruler ......1050......1060......1070......1080......1090......1100......1110......1120
```

```
Eca1   --------------------------------------------------------------------------------   53
Esp2   --------------------------------------------------------------------------------   53
Cko1   --------------------------------------------------------------------------------   53
Sen1   --------------------------------------------------------------------------------   53
Eco3   --------------------------------------------------------------------------------   49
Esa3   --------------------------------------------------------------------------------   54
Kpn1   --------------------------------------------------------------------------------   51
Pan1   --------------------------------------------------------------------------------   61
Eta2   --------------------------------------------------------------------------------   52
Spr1   --------------------------------------------------------------------------------   69
Eco25  INDHTVTFAVLSGSATSFNNQNT---------------------------------------------------------  304
Eco15  LTNHQVTFKNEKGSA-EFVEPPQ---------------------------------------------------------  303
Yfr1   VPNVSVTFAVTGGATFAG--------------------------------------------------------------  290
Yfr5   IPNFVVSFVGSNGANIAAN-------------------------------------------------------------  293
Yps4   VPGVSVTFDAGNGAVLAQNPVVTDRNGYAENTLTNLAIGTTTVKATTVTDPVGQTVNTHFVAGAVDTITLTVLVNGAVAN  447
Efe2   IDNLDVKAAIDMPVGMTIALQDFVK-------------------------------------------------------  317
Efe3   VSDLTIDAVAETSQKMDITLSKHFS-------------------------------------------------------  317
Eta1   QGAGIPVDWIANEGSTLGAQTSS---------------------------------------------------------  199
Eco26  IDAGTTVIWSADTGTVLNTNVVT---------------------------------------------------------  296
Pru1   LGAGVPVFWVGNDNTLLSDNQTM--------------------------------------------------------- 1018
Sgl1   IED-KPVTLQAEGGVVLSTNSVT---------------------------------------------------------  298
Pmi1   IPA-THVTLSADNQAQLKQTILT---------------------------------------------------------  301
Ymo1   --------------------------------------------------------------------------------  251
Yen2   VVPGAAVDWLSNIGTFVQGSTTT---------------------------------------------------------  312
Sen2   RTLCIN--------------------------------------------------------------------------  200
Efe4   KTTCDT--------------------------------------------------------------------------  203
Bpe1   SPEATRAYADTVDTTPPAVPTITD--------------------------------------------------------  371
Bav2   SPETSRNYVDTVDKTAPAAPTITN--------------------------------------------------------  368
Bpa2   SAPARTVFADAVDRTAPAAPTLA---------------------------------------------------------  370
Bav1   GSAATQTYTRNPPTAHISS-------------------------------------------------------------  271
Yin1   TAG-CAITLNNFDTG-ATGYSFT---------------------------------------------------------  245
Yfr4   TAG-CAITLNNHDTG-VTGYQFT---------------------------------------------------------  245
Yin2   TAG-CAITLDNHDTG-MTGYTFT---------------------------------------------------------  246
Ymo2   TGGSSGITLVDHEIAGATTDHYT---------------------------------------------------------  264
Eco10  INGEVGAQANTSNED-IVIPATN---------------------------------------------------------  264
Eal1   AHGETGAQANTTNED-IVIPATN---------------------------------------------------------  263
Eco16  AHGSTGAKANTINED-LILPATN---------------------------------------------------------  264
Sty4   TNGHTGALANTINED-LVLPVTN---------------------------------------------------------  263
Yfr2   GFNQATFTINMNGTADYSWSSNQP--------------------------------------------------------  297
Ype5   VPGVDVAFTTDIGAIISEVTPTD---------------------------------------------------------  322
Eco6   LQGQNVTFTVVNGAAVFADPNGG---------------------------------------------------------  302
Bbr1   GKPVRRPYVDTVAPTPMKVTIDS---------------------------------------------------------  280
Yps2   ISGMQGLSFTQNGVPVSISPITE---------------------------------------------------------  298
Eco14  VAGQAVTWHTDNGQFTQQDAVTN---------------------------------------------------------  278
Sen3   VPDSPLKWQIVEGQATLSATQTT---------------------------------------------------------  294
Pal3   --------------------------------------------------------------------------------   86
Ybe1   VTGSTVVTTLETVTDANSMKVGETIN------------------------------------------------------  283
Pal2   IIDAQNDITTIIDKKTESTAIWDT--------------------------------------------------------  321
Eco1   GNGGYSWSSDNPDIASIDANTGIIT-------------------------------------------------------  295
Yps7   VTGQTVALVSTLGTLGAVTEQAS---------------------------------------------------------  298
Yfr3   VEDVAVKFEADNGATVITPEGIT---------------------------------------------------------  305
Eco20  VGGTVTAIWTAKDAYDNPVTSLTPEAPSLAG-------------------------------------------------  307
Efe5   VPGYAVTLSLPSGITQVGGEHAVS--------------------------------------------------------  312
Csu1   IQPSLPPSLQLDNTGKISGTVQSEVG------------------------------------------------------  307
Ahy1   TAADVDGNYTLEAPTVQGSGTITAT-------------------------------------------------------  305
Plu1   VMGQPVNIDWQTDPKADGLKLTKQNNS-----------------------------------------------------  310
Cla1   TLRISNNKETTSNEKGESKVVFEA--------------------------------------------------------  290
Pas1   QERLERERLDQERVEQEARDAWLRQ-------------------------------------------------------  293
ruler  ......1130......1140......1150......1160......1170......1180......1190......1200
```

```
Eca1   --------------------------------------------------------------------------------   53
Esp2   --------------------------------------------------------------------------------   53
Cko1   --------------------------------------------------------------------------------   53
Sen1   --------------------------------------------------------------------------------   53
Eco3   --------------------------------------------------------------------------------   49
Esa3   --------------------------------------------------------------------------------   54
Kpn1   --------------------------------------------------------------------------------   51
Pan1   --------------------------------------------------------------------------------   61
Eta2   --------------------------------------------------------------------------------   52
Spr1   --------------------------------------------------------------------------------   69
Eco25  ---------------------------------------AKTDVNGLATFDLKSSKQEDNTVEVTLEN------------  333
Eco15  ---------------------------------------QNTDAYGVATINMVSQVAEENTISATLPN------------  332
Yfr1   --------------------------------------------------------------------------------  290
Yfr5   ---------------------------------------GTTGADGSVTQPLTNTTAGVTHVTASVNGHSQAVDVTFVP-  333
Yps4   GVNTNSVQAVVSDSGGNPVNGAAVVFSSANATAQITTVIGTTGVDGIATATLTNTVAGTSNVVATIDTVNANIDTTFVAG  527
Efe2   -----------------------------------------GAEKGVYTATLKGTSQGEVSIMPQVGGEN----------  346
Efe3   -----------------------------------------QEKPGTYTAVLHGTTPGDVYIMPKVGNLA----------  346
Eta1   -----------------------------------------SDETGTVAVTLTRAQPGTAKVSLILPSG-KYSAPDVEFR  237
Eco26  -----------------------------------------TDKAGSVSVQATRSQPGIAKVELLLPSGGKVTAPDVIFN  335
Pru1   -----------------------------------------TDTTGRSAIQVQRDTIGEALVSLNLISQNSARAPIVAFV 1057
Sgl1   -----------------------------------------TDKDGTATFNAMSTTSGTFKVTATTNGYSKTASVVFVAG  337
Pmi1   -----------------------------------------TDEQGKATTTMISKVAGTVTVRAKINDKMTHATTQFIAN  340
Ymo1   --------------------------------------------------------------------------------  251
Yen2   -----------------------------------------TDTNGETFIELVSTKAETAKVTATVGGKPYNAGKVVFVA  351
Sen2   ------------------------------------------NTDCTDAFNYQWEISDEMKSWKSVPGATKAT-------  231
Efe4   ------------------------------------------DKDCSSLFRYQWEISPDGNRWYDVPGATGQS-------  234
Bpe1   ----------------------------------------VTTDATNGRITVTGVAEPGANVTVDFPDGTRKT-------  404
Bav2   ----------------------------------------VATDAATGHVTVSGRAESDTQVTVTFPSGESKQ-------  401
Bpa2   ----------------------------------------LSEAADSGRLTVSGRTEPGASVRVTFPDGETVT-------  403
Bav1   -----------------------------------------ATADTAGKLSIRGQTEPGLSVFVLFPSGDDMT-------  303
Yin1   ------------------------------------------PRPNGSSNSGVACGDQGFGLKVNY--------------  269
Yfr4   ------------------------------------------PRTNGSSNSGVACGDQGFGLKVNW--------------  269
Yin2   ------------------------------------------PRPNASSNSGVVCGDQGFGLKVNY--------------  270
Ymo2   ------------------------------------------VAVNHASSSGQTAGDQGFKLKVGFH-------------  289
Eco10  ------------------------------------------REAAQTYGAQAGDGLQGYGLRVLYTKK-----------  291
Eal1   ------------------------------------------QEAAQKFSAQAGDGVQGYGLQVKYSKK-----------  290
Eco16  ------------------------------------------KEAASKFDAEERDGVQGYGIRVVYSEKQKSA-------  295
Sty4   ------------------------------------------KEAAQKFAANVEDGVQGYGIRVTYSQK-----------  290
Yfr2   ---------------------------------------AAVTVNSSGQVTFNGPPSGTVTITATPNNGGSPQSYSFTVE  338
Ype5   -------------------------------------------ANGVATAKIISSQAKSHTVKATLNRKEQ---------  350
Eco6   --------------------------------------IVTTDKDGIASVNLASDQAVNSLIKAEINGSSQSVEVSFITG  344
Bbr1   ----------------------------------------MRTDGNSGVVTVTGYTVGGSTVTVTFPDGTTAG-------  313
Yps2   -------------------------------------------QPDSYTATVVGNSVGDVTITPQVDTLILST-------  328
Eco14  -------------------------------------------AAGTASATLTSTQAGNAQVSLSLNGTTTTVSAPRVS-  314
Sen3   -----------------------------------------TDQQGSGSITLTSSTQGNVVVSVSATAGDAVNSPALLFI  333
Pal3   --------------------------------------------------------------------------------   86
Ybe1   ---------------------------------------LTVTALNKNNLPVSGATIKVQAVSASSRNGQAET-------  317
Pal2   -----------------------------------------DIDKGIYAAKISGQQPGTHTIQVVMGKTVSQEQKFNTLG  360
Eco1   -----------------------------------------LNKKGTTVIKVISGDKQIATYTIKTPQEIVSLDN-----  329
Yps7   --------------------------------------------GVYTATLTAGTVAGVASLSVSVGGSALG--------  326
Yfr3   ------------------------------------------DTEGIAIVELANINPGVTTLNAIIDGHEERVETSFMPE  343
Eco20  -----------------------------------------AAAVGSTASGWTNNGDGTWTAQITLGSTAGELEVMPKLN  346
Efe5   -----------------------------------------TDENGNAVFTLVSSTPGSYVIKAHAGPQDSSE-------  344
Csu1   -----------------------------------------IPETNYTVFVRDSLGTQRASTSFSLVVNPPFVISQKLYR  346
Ahy1   -----------------------------------------ATDKSGNTGPASSVNYLASLVGVTITGLVDGFP------  338
Plu1   ---------------------------------------VSNAQGQVTATLTSTAAVENVQVSAKTASQQTAVNANGKVS  351
Cla1   -----------------------------------------IKDYNVKNLEITAIYNQSLANKIEVKDNIKLYQYS----  325
Pas1   -----------------------------------------EEQERLEQERLEQERLERERLEQERLEQERLERE-----  327
ruler  ......1210......1220......1230......1240......1250......1260......1270......1280
```

```
Eca1  --------------------------------------------------------------------------------   53
Esp2  --------------------------------------------------------------------------------   53
Cko1  --------------------------------------------------------------------------------   53
Sen1  --------------------------------------------------------------------------------   53
Eco3  --------------------------------------------------------------------------------   49
Esa3  --------------------------------------------------------------------------------   54
Kpn1  --------------------------------------------------------------------------------   51
Pan1  --------------------------------------------------------------------------------   61
Eta2  --------------------------------------------------------------------------------   52
Spr1  --------------------------------------------------------------------------------   69
Eco25 -------------------------------------GVKQTLIVSFVGDSSTAQVDLQKSKNEVVADGNDSATMTATVR  376
Eco15 -------------------------------------GFSQRIIAKFVSDSSTPKFKQLVADPDTIIAGN----------  365
Yfr1  -------------------------------------GTQTSL-------------------------------------  296
Yfr5  -----------------------------------DSGTATIISGAL---------------------------------  345
Yps4  AVATITLTTLVNGAVADGANSNSVQAVVSDSGGNPVTGAAVVFSSANATAQITTVIGTTGVDGIATATLTNTVAGTSNVV  607
Efe2  -------------------------------------AARDAVKVTLSRT-FDETKSSFTTNYSEYEDEE----------  378
Efe3  -------------------------------------LAGAPVKVQLKSAGASVKESTLAVASTEYRAGD----------  379
Eta1  A------------------------------------GVPDESRSELILTPAVIEAVKEPAMLTLILRDKNG--------  273
Eco26 N------------------------------------EDFDEDKSELKLYPETINAGKDFANLELVLKDKKG--------  371
Pru1  Q------------------------------------GALDVSKSSLNLTPSTVTAG-KPTTLKLVLKDQFG-------- 1092
Sgl1  -------------------------------------GVVGVKSTLAADKSLIASNGSSAISLTFTAHDNH---------  371
Pmi1  -------------------------------------QAKGVIVSITPSSTTHVADGQTPVFLTALVQDQFGKPLPHAQI  383
Ymo1  --------------------------------------------------------------------------------  251
Yen2  D------------------------------------RQSGKITLLPVSKNTAAANGTDSITLNAKILDANGNPIKNEEI  395
Sen2  -------------------------------------WLLP-----YSLNGESLQNKYIRVRIISDKENAES--------  261
Efe4  -------------------------------------WLMP-----AVMDGHSLQNKQVRVRVVS--ENVPT--------  262
Bpe1  -------------------------------------VVAGGDGAYTATSDRDMASGDVRVQAIDKGGNRSP--------  439
Bav2  -------------------------------------VPADAGGAYRVSSDTDQPSGEIKAQAADAAGNKSP--------  436
Bpa2  -------------------------------------VTAKADGTYTATSRADMIGGNVTVVATDAAGNAAA--------  438
Bav1  -------------------------------------VQADAQGNFHATSSQKETAGIVSVTVSGKDGGVGV--------  338
Yin1  --------------------------------------------------------------------------------  269
Yfr4  --------------------------------------------------------------------------------  269
Yin2  --------------------------------------------------------------------------------  270
Ymo2  --------------------------------------------------------------------------------  289
Eco10 --------------------------------------------------------------------------------  291
Eal1  --------------------------------------------------------------------------------  290
Eco16 --------------------------------------------------------------------------------  295
Sty4  --------------------------------------------------------------------------------  290
Yfr2  N---------------------------------WFEGDSTSRNWGNANADCNSRSEVLPTIAQLTSGEYIRQIG-----  380
Ype5  -------------------------------------TVEVNFIADTATAEITANNFTVEVDGQVAGSGTN---------  384
Eco6  DISQLTSTIKTDDVSYTAG------------------GKIKVSVTLMDEQKNRVKGMASLLAGSSVVEVSGTDKNETGNW  406
Bbr1  -------------------------------------TTANDRGKYTVTSTADIPAGPIRVSARGPRNQQGS--------  348
Yps2  -------------------------------------LQKKISLFPVPTLTGILVNGQNFATDKGFPKTIFKN-------  364
Eco14 -------------------------------------FTQQLYLTLQAGRTTAVADGNDAITYTLNVRDAAG--------  349
Sen3  AD-----------------------------------TATAKVSDITVDKSQGVANGNDHITYTAIVTDASGNPVKDQEV  378
Pal3  --------------------------------------------------------------------------------   86
Ybe1  -------------------------------------ATLQVNGKPLTDDVTTDGDGKVQVALTDPTGIG----------  350
Pal2  TN---------------------------------AVASVAADGSGPLGTLGVIADIEIDINPNRTDFKSGDNPLVMVTL  407
Eco1  -------------------------------------SVKVKYDEASGICSNNAAYLSVSVDSLKKLYSQWG--------  364
Yps7  --------------------------------------VAPATVTLNGDSGNLSTTHSTLVAAPVSIEANGSD-------  361
Yfr3  IP-----------------------------------LKLLIYRNGAELIGHPAVGDTLVAVAMCSIALCNGVPIN----  384
Eco20 GQ----------------------------------DAAANAAKVTVVADALSSNQSKVSVAEDHVKAGES---------  383
Efe5  --------------------------------------LTVTFASNMTGASLSLTPENSSATANIPANGKD---------  377
Csu1  R----------------------------------VLSTDSQVNIQAFEITGGEPPYTVTVSPTLPSGLSVSN-------  385
Ahy1  --------------------------------------QVGAVLTAVSDCGSSACRAGLTWQWQIEDGVGSG--------  372
Plu1  FTEFS------------------------------TSYYVASVTVEVDKDKAHYNNGSDSYTFTATVKDGHGNLVVGQPV  401
Cla1  -------------------------------------LAMNSQKDKLDAYENGLATKNLVLDKTEVKVTGGKP-------  361
Pas1  -------------------------------------RLDQERVEQEARDAWLRQEEQERLEQERLEQERLEQ-------  363
ruler ......1290......1300......1310......1320......1330......1340......1350......1360
```

```
   Eca1 --------------------------------------------------------------------------------   53
   Esp2 --------------------------------------------------------------------------------   53
   Cko1 --------------------------------------------------------------------------------   53
   Sen1 --------------------------------------------------------------------------------   53
   Eco3 --------------------------------------------------------------------------------   49
   Esa3 --------------------------------------------------------------------------------   54
   Kpn1 --------------------------------------------------------------------------------   51
   Pan1 --------------------------------------------------------------------------------   61
   Eta2 --------------------------------------------------------------------------------   52
   Spr1 --------------------------------------------------------------------------------   69
  Eco25 DAKGNLLNDVKVTFNVNSAAAKLSQTEVN---------------------------------------------SHDGIA  411
  Eco15 --------------------------------------------------------------------------------  365
   Yfr1 --------------------------------------------------------------------------------  296
   Yfr5 --------------------------------------------------------------------------------  345
   Yps4 ATIGSITNNIDTAFVAGAVATITLTTPVNGAVADGANSNSVQAVVTDSGGNPVNGAAVVFSSANATAQITTVIGTTGADG  687
   Efe2 --------------------------------------------------------------------------------  378
   Efe3 --------------------------------------------------------------------------------  379
   Eta1 -------------------------------------------------------NTLSGNTVRGVSDNSLVTISNGEEI  298
  Eco26 -------------------------------------------------------NYLSGQLVHGFSNNTSVIVSDARED  396
   Pru1 -------------------------------------------------------NPLVNQQTAIHVRKDKNHVVISPVT 1117
   Sgl1 ------------------------------------------------------NNAVTGLTVAFEPNGVEGKISTVSEQ  397
   Pmi1 SWETEHDKSIV----------NIEHTTMTNEQGITTNKLTSTQALSVRVTARINNNAFTAEPITFIANGQQALISELLVN  453
   Ymo1 --------------------------------------------------------------------------------  251
   Yen2 EWDAASHKVTFSPATGKTQTNDLGETQITLTSTDVGDITLNAQVVKNNLLVNQAGEKLSFTADTVTANISAWSAPSVKTL  475
   Sen2 --------------------------------------------------------------------------------  261
   Efe4 --------------------------------------------------------------------------------  262
   Bpe1 --------------------------------------------------------------------------------  439
   Bav2 --------------------------------------------------------------------------------  436
   Bpa2 --------------------------------------------------------------------------------  438
   Bav1 --------------------------------------------------------------------------------  338
   Yin1 --------------------------------------------------------------------------------  269
   Yfr4 --------------------------------------------------------------------------------  269
   Yin2 --------------------------------------------------------------------------------  270
   Ymo2 --------------------------------------------------------------------------------  289
  Eco10 --------------------------------------------------------------------------------  291
   Eal1 --------------------------------------------------------------------------------  290
  Eco16 --------------------------------------------------------------------------------  295
   Sty4 --------------------------------------------------------------------------------  290
   Yfr2 --------------------------------------------------------------------------------  380
   Ype5 --------------------------------------------------------------------------------  384
   Eco6 SEESDGVYTTTRTAKIAGDRHYATLKLSTWSSAQQSDAYAIRESGAVLAYSSIVTDKTAYTAGGAIKVTVTLKDSYENLV  486
   Bbr1 --------------------------------------------------------------------------------  348
   Yps2 --------------------------------------------------------------------------------  364
  Eco14 --------------------------------------------------------------------------------  349
   Sen3 TWN-----------------------------------------------------------------------------  381
   Pal3 --------------------------------------------------------------------------------   86
   Ybe1 --------------------------------------------------------------------------------  350
   Pal2 KDSFGNEIENIDPSNIHLGDFKGNDIAWRQDGKYDYIVNLPLTKTGDIDITAHVNGIFSPKTVLTVSHNVDISKIQDVVL  487
   Eco1 --------------------------------------------------------------------------------  364
   Yps7 --------------------------------------------------------------------------------  361
   Yfr3 --------------------------------------------------------------------------------  384
  Eco20 --------------------------------------------------------------------------------  383
   Efe5 --------------------------------------------------------------------------------  377
   Csu1 --------------------------------------------------------------------------------  385
   Ahy1 --------------------------------------------------------------------------------  372
   Plu1 NIDWQTN-------------------------------------------------------------------------  408
   Cla1 --------------------------------------------------------------------------------  361
   Pas1 --------------------------------------------------------------------------------  363
  ruler ......1370......1380......1390......1400......1410......1420......1430......1440
```

```
   Eca1 --------------------------------------------------------------------------------   53
   Esp2 --------------------------------------------------------------------------------   53
   Cko1 --------------------------------------------------------------------------------   53
   Sen1 --------------------------------------------------------------------------------   53
   Eco3 --------------------------------------------------------------------------------   49
   Esa3 --------------------------------------------------------------------------------   54
   Kpn1 --------------------------------------------------------------------------------   51
   Pan1 --------------------------------------------------------------------------------   61
   Eta2 --------------------------------------------------------------------------------   52
   Spr1 --------------------------------------------------------------------------------   69
  Eco25 TATLTSLKNGDYTVTASVSSGSQANQQVIFIGDQSTAALTLSVPSGDITVTNTAPLHMTATLQDKNGNPLKDKEITFSVP  491
  Eco15 ------------------SQGS----------------------------------TLTAIITDFHNNPLKDMKVNFVAP  393
   Yfr1 --------------------------------------------------------------------------------  296
   Yfr5 --------------------------------------------------------------------------------  345
   Yps4 IATATLTNTVAGTSNVVATVDTVNANIDTTFVAGAVATITLTTPVNGAVADGADSNSVQAVVSDSGGNPVAGAAVVFSSA  767
   Efe2 ------------------------------------------------------PIILKLSLKDKDGKPISGMAG-----  399
   Efe3 ------------------------------------------------------EMVVALALRDKFGNPLGSEE------  399
   Eta1 ANK-----------------------------------------------PGNYGMTVTASKAGKAILSVTVNGKPFGQL  331
  Eco26 INK-----------------------------------------------PGHYKMTVTGSKSGIALLSVSVNNKTLPLT  429
   Pru1 VVD-----------------------------------------------DGVYQVSVSSEQADTAILSVDIGTQSLPQT 1150
   Sgl1 --------------------------------------------------DGVYTATFTSTKTGVGTIGVAVN-GTRLEE  426
   Pmi1 KNQIVADKQDPAELTAIVTDKLGNQLPDTIVNWQGSAGTQFAQQQTKTDAQGIAKNTLTTSQSGITTITARLNNGQHAQT  533
   Ymo1 --------------------------------------------------------------------------------  251
   Yen2 IADGQAQVIYKVVVKDKNGHVVPNSPVLWETNLGEFVPAQATTTMTSTDSQGEATVVLASIKAGSATVKASVNANKDTSP  555
   Sen2 --------------------------------------------------------NNATSAAN----------------  269
   Efe4 --------------------------------------------------------H-----------------------  263
   Bpe1 --------------------------------------------------------EATRAYADTVDKTAP---------  454
   Bav2 --------------------------------------------------------EATKAYDDTVDKTAPPLSITNVST  460
   Bpa2 --------------------------------------------------------PVRAAYADTVDRTPPVLDTPVLSV  462
   Bav1 --------------------------------------------------------PVEYHYSTA---------------  347
   Yin1 --------------------------------------------------------------------------------  269
   Yfr4 --------------------------------------------------------------------------------  269
   Yin2 --------------------------------------------------------------------------------  270
   Ymo2 --------------------------------------------------------------------------------  289
  Eco10 --------------------------------------------------------------------------------  291
   Eal1 --------------------------------------------------------------------------------  290
  Eco16 --------------------------------------------------------------------------------  295
   Sty4 --------------------------------------------------------------------------------  290
   Yfr2 --------------------------------------------------------SLFGEWGDMMDYGFPAANNWSSEQ  404
   Ype5 --------------------------------------------------------QVQALVVDKKGNPVANMTVNFTAT  408
   Eco6 GGQRDAINLAIQLPNTKAESIAWNEDQKGIYTATYTALLPGTGLKAQLQMSGWANALTSNDYSISGDAASAQIVAMQVTT  566
   Bbr1 --------------------------------------------------------------------------------  348
   Yps2 --------------------------------------------------------------------------------  364
  Eco14 -----------------------------------------------------QPVKDKAVQWSTDLGTLSPLQGTTSAQ  376
   Sen3 --------------------------------------------------ATPATANLSSATSTTDSNGNTSVTITTLKA  411
   Pal3 --------------------------------------------------------------------------------   86
   Ybe1 --------------------------------------------------------------------------------  350
   Pal2 PPINNTPQAGEIPSISVVLTDSHGNPVNGVKQLEVTIAGTPHTLPATQNPDGSYTVTLPAQHSGKQDIQVSVNGKDSNKE  567
   Eco1 ------------------------------------------------------PANKYSHYTQGTINAWIQQTEQDKKD  390
   Yps7 ------------------------------------------------------TSLVTLTLRDSNNNPVTGQTVALVST  387
   Yfr3 -----------------------------------------------------YQWEIESSVGSGGFIAIAGATSETLTV  411
  Eco20 -------------------------------------------------------TTVTLIAKDAHGNTISGLSLSASLT  408
   Efe5 ------------------------------------------------------AAVLNVQLTNNTNASVNGQKVELITS  403
   Csu1 --------------------------------------------------TGVISGQLGTAQTQEQTYTFTVQDQQQESK  415
   Ahy1 --------------------------------------------------------NFVDIGGATSVTYLPAREDQKKRV  396
   Plu1 -----------------------------------------------PKADGLKLTKQSNSVSNAQGQVTATLTSTVAVE  441
   Cla1 --------------------------------------------------------NEKVEWDLTGDGTLSNQELSFDQN  385
   Pas1 --------------------------------------------------------------------------------  363
  ruler ......1450......1460......1470......1480......1490......1500......1510......1520
```

```
Eca1   --------------------------------------------------------------------------------   53
Esp2   --------------------------------------------------------------------------------   53
Cko1   --------------------------------------------------------------------------------   53
Sen1   --------------------------------------------------------------------------------   53
Eco3   --------------------------------------------------------------------------------   49
Esa3   --------------------------------------------------------------------------------   54
Kpn1   --------------------------------------------------------------------------------   51
Pan1   --------------------------------------------------------------------------------   61
Eta2   --------------------------------------------------------------------------------   52
Spr1   --------------------------------------------------------------------------------   69
Eco25  NDVASRFSISNSGKGMTDSNGTAIASLTGTLAGTHMI-------------------------------------------  528
Eco15  --------------------------------------------------------------------------------  393
Yfr1   --------------------------------------------------------------------------------  296
Yfr5   --------------------------------------------------------------------------------  345
Yps4   NATAQVTTVIGTTGADGIATATLTNTVAGTSNVVATIGSITNNIDTAFVAGAVATITLSVPVNDATADGVDTNQVDALVQ  847
Efe2   --------------------------------------------------------------------------------  399
Efe3   --------------------------------------------------------------------------------  399
Eta1   KTLLT---------------------------------------------------------------------------  336
Eco26  KKLVVNGDMDSWEIAEIRTNKNNVIAGDKDGVTYST--------------------------------------------  465
Pru1   KTLTVQGDTANGSISSLMPSVNHMQAGNQTGVTYYA-------------------------------------------- 1186
Sgl1   LTAVDAGVYSSTLSLSVNVSS-----------------------------------------------------------  447
Pmi1   NLVAVADLKSATLTLTTLNNKQSAIADNQDSITVVANLTDAYHNPLKNIPVYWQSSINHIDETTTQTDEKGRTQVVISGT  613
Ymo1   --------------------------------------------------------------------------------  251
Yen2   TQVEFTADSSTATIAITPVTKQVYVANGSEKVTYAVTVLDANNNPVKAEAINWKSENGHPVKVEPAPSQTDGQGKATVSI  635
Sen2   --------------------------------------------------------------------------------  269
Efe4   --------------------------------------------------------------------------------  263
Bpe1   ---------------------------------AAP--------------------------------------------  457
Bav2   APATGVVTVTGNTEAGSSVAVNFPGGQSVNVTAAADG-------------------------------------------  497
Bpa2   ASDSGRVTVTGVTEPGARVQVAIPGESLQTVTADSAG-------------------------------------------  499
Bav1   --------------------------------------------------------------------------------  347
Yin1   --------------------------------------------------------------------------------  269
Yfr4   --------------------------------------------------------------------------------  269
Yin2   --------------------------------------------------------------------------------  270
Ymo2   --------------------------------------------------------------------------------  289
Eco10  --------------------------------------------------------------------------------  291
Eal1   --------------------------------------------------------------------------------  290
Eco16  --------------------------------------------------------------------------------  295
Sty4   --------------------------------------------------------------------------------  290
Yfr2   SGTSNYYYVVNSSRGRSSQALYTPGGTSVLNKSLCVRDL-----------------------------------------  443
Ype5   NG------------------------------------------------------------------------------  410
Eco6   GNPDVLANGSDRHTVNVRVEDQFGNVLPEQTVTFTVTKGAAVFANAGQSADIRTDAHGMAEVDLSSTVADASTVEAKINQ  646
Bbr1   --------------------------------------------------------------------------------  348
Yps2   --------------------------------------------------------------------------------  364
Eco14  GEAS----------------------------------------------------------------------------  380
Sen3   GSVNVTAQAGASPAWNAPTTTFVGDKATAQILDLKTN-------------------------------------------  448
Pal3   --------------------------------------------------------------------------------   86
Ybe1   --------------------------------------------------------------------------------  350
Pal2   TLTVQAPTPIPSKVQGNTGEQGVLETVTLSSAALTGLQSGDTLDLTVTAKDAFKNPLTGLASAIALTHGQTGSVTWTDNQ  647
Eco1   SVATTYDIVTDNTVRNVNSTVANAYAVCIK--------------------------------------------------  420
Yps7   LGTLGAVTEQASG-------------------------------------------------------------------  400
Yfr3   TGDLQKRAIRVVSH------------------------------------------------------------------  425
Eco20  GTASEGATVSSWTEKG----------------------------------------------------------------  424
Efe5   AEG-----------------------------------------------------------------------------  406
Csu1   NISIVLAVVTG---------------------------------------------------------------------  426
Ahy1   RVIVAPL-------------------------------------------------------------------------  403
Plu1   NVQVSAKTASQQTAVNANGKVSFTEFSTSYYVASVTVEVD----------------------------------------  481
Cla1   GE------------------------------------------------------------------------------  387
Pas1   --------------------------------------------------------------------------------  363
ruler  ......1530......1540......1550......1560......1570......1580......1590......1600
```

```
Eca1   --------------------------------------------------------------------------------   53
Esp2   --------------------------------------------------------------------------------   53
Cko1   --------------------------------------------------------------------------------   53
Sen1   --------------------------------------------------------------------------------   53
Eco3   --------------------------------------------------------------------------------   49
Esa3   --------------------------------------------------------------------------------   54
Kpn1   --------------------------------------------------------------------------------   51
Pan1   --------------------------------------------------------------------------------   61
Eta2   --------------------------------------------------------------------------------   52
Spr1   --------------------------------------------------------------------------------   69
Eco25  ----------------------------------------------TARLANSNVSDTQPMTFVADKDRAVVVLQTSKAE  562
Eco15  --------------------------------------------------------------------------------  393
Yfr1   --------------------------------------------------------------------------------  296
Yfr5   --------------------------------------------------------------------------------  345
Yps4   DANGNAITGAAVVFSSTNGADIIVPTMNTGVNGVASTLLTHTVAGTSNVVATVDTVNANIDTAFVPGAVATITLTTPVNG  927
Efe2   --------------------------------------------------------------------------------  399
Efe3   --------------------------------------------------------------------------------  399
Eta1   --------------------------------------------------------------------------------  336
Eco26  --------------------------------------------------------------------------------  465
Pru1   -------------------------------------------------------------------------------- 1186
Sgl1   --------------------------------------------------------------------------------  447
Pmi1   KAQPTTITAYLSNKEKKSIQVTFVAGAPVQQNSYLTIEPQSIIADGNAFAVGKIDLRDKFDNPVIGRSNDIALIGDNSTI  693
Ymo1   --------------------------------------------------------------------------------  251
Yen2   GSVKAGDTQIRATLGNNATAIADAITFEADRQTAVVKTVEVTGSKVTAPDGTGSISYVTTVVDANGNPVSGMILSWGSNI  715
Sen2   --------------------------------------------------------------------------------  269
Efe4   --------------------------------------------------------------------------------  263
Bpe1   --------------------------------------------------------------------------------  457
Bav2   --------------------------------------------------------------------------------  497
Bpa2   --------------------------------------------------------------------------------  499
Bav1   --------------------------------------------------------------------------------  347
Yin1   --------------------------------------------------------------------------------  269
Yfr4   --------------------------------------------------------------------------------  269
Yin2   --------------------------------------------------------------------------------  270
Ymo2   --------------------------------------------------------------------------------  289
Eco10  --------------------------------------------------------------------------------  291
Eal1   --------------------------------------------------------------------------------  290
Eco16  --------------------------------------------------------------------------------  295
Sty4   --------------------------------------------------------------------------------  290
Yfr2   --------------------------------------------------------------------------------  443
Ype5   --------------------------------------------------------------------------------  410
Eco6   SSDSKTVNFVADVSTAQVAELVVTQDGSVADGATANTLRARVTDAFGNALAGQTVSVLAGNGATTAPTVTTQPDGTVEIS  726
Bbr1   --------------------------------------------------------------------------------  348
Yps2   --------------------------------------------------------------------------------  364
Eco14  --------------------------------------------------------------------------------  380
Sen3   --------------------------------------------------------------------------------  448
Pal3   --------------------------------------------------------------------------------   86
Ybe1   --------------------------------------------------------------------------------  350
Pal2   DGTYTASLTLSKLGKDSLTATANKVDSNTLKVNVTNRTGHTGVQNVVITPTNKAPQAGETPTLTVTLTDSNGNPVNDIQQ  727
Eco1   --------------------------------------------------------------------------------  420
Yps7   --------------------------------------------------------------------------------  400
Yfr3   --------------------------------------------------------------------------------  425
Eco20  --------------------------------------------------------------------------------  424
Efe5   --------------------------------------------------------------------------------  406
Csu1   --------------------------------------------------------------------------------  426
Ahy1   --------------------------------------------------------------------------------  403
Plu1   --------------------------------------------------------------------------------  481
Cla1   --------------------------------------------------------------------------------  387
Pas1   --------------------------------------------------------------------------------  363
ruler  ......1610......1620......1630......1640......1650......1660......1670......1680
```

```
Eca1   --------------------------------------------------------------------------------   53
Esp2   --------------------------------------------------------------------------------   53
Cko1   --------------------------------------------------------------------------------   53
Sen1   --------------------------------------------------------------------------------   53
Eco3   --------------------------------------------------------------------------------   49
Esa3   --------------------------------------------------------------------------------   54
Kpn1   --------------------------------------------------------------------------------   51
Pan1   --------------------------------------------------------------------------------   61
Eta2   --------------------------------------------------------------------------------   52
Spr1   --------------------------------------------------------------------------------   69
Eco25  IIGNGVDETTLTATVKDPFDNVVKNLSVVFRTSPADTQLSLNARNTNENGIAEVTLKGTVLGVHTAEAILLNGNRDTKIV  642
Eco15  ----------------------------------GGSQLDNTTATTDQSGIVRVHLT-----------------------  416
Yfr1   --------------------------------------------------------------------------------  296
Yfr5   --------------------------------------------------------------------------------  345
Yps4   AVADGANSNSVQAVVSDSEGNAVAGAAVVFSSANATAQITTVIGTTGADGIATATLTNTVAGTSNVVATIDTVNANIDTA 1007
Efe2   -----------------------------------SLNKDALLSVEHALQKNSWTETGEA--------------------  424
Efe3   -----------------------------------ALQTAEKLEVQHAWPLQAWKEIGEDKYERIYQAQNTGANLQARLT  444
Eta1   --------------------------------------------------------------------------------  336
Eco26  --------------------------------------------------------------------------------  465
Pru1   -------------------------------------------------------------------------------- 1186
Sgl1   --------------------------------------------------------------------------------  447
Pmi1   QFSKITETANGNYQAHIRGTKAGLSQITATIDSITVTQSLGFLTDNKTVKIHSVKVMAPYTVTANGKDKVIIQAQITDKH  773
Ymo1   --------------------------------------------------------------------------------  251
Yen2   NNVANPSTTTDINGQSSQTITGTQAGKVEISVALTSGNNATNPVKNSNNAEFVAVTPVMANADLLLQPNLIIANGKQTAT  795
Sen2   --------------------------------------------------------------------------------  269
Efe4   --------------------------------------------------------------------------------  263
Bpe1   --------------------------------------------------------------------------------  457
Bav2   --------------------------------------------------------------------------------  497
Bpa2   --------------------------------------------------------------------------------  499
Bav1   --------------------------------------------------------------------------------  347
Yin1   --------------------------------------------------------------------------------  269
Yfr4   --------------------------------------------------------------------------------  269
Yin2   --------------------------------------------------------------------------------  270
Ymo2   --------------------------------------------------------------------------------  289
Eco10  --------------------------------------------------------------------------------  291
Eal1   --------------------------------------------------------------------------------  290
Eco16  --------------------------------------------------------------------------------  295
Sty4   --------------------------------------------------------------------------------  290
Yfr2   --------------------------------------------------------------------------------  443
Ype5   --------------------------------------------------------------------------------  410
Eco6   VTSQTAGTSVITASVNNSSQSRNVTFIADVSTAQIADLVVSQDNAVADGATANTLQVRVTDAFGNALAGQTVSVLADNGA  806
Bbr1   --------------------------------------------------------------------------------  348
Yps2   --------------------------------------------------------------------------------  364
Eco14  --------------------------------------------------------------------------------  380
Sen3   --------------------------------------------------------------------------------  448
Pal3   --------------------------------------------------------------------------------   86
Ybe1   --------------------------------------------------------------------------------  350
Pal2   LEVTIAGTTHTLPATQDPDGSYTVTLPAQHSGKQDIQVSVNGKDSNKETLTVQAPTPIPSKVQGNMGEQGVLETVTLSSA  807
Eco1   --------------------------------------------------------------------------------  420
Yps7   --------------------------------------------------------------------------------  400
Yfr3   --------------------------------------------------------------------------------  425
Eco20  --------------------------------------------------------------------------------  424
Efe5   --------------------------------------------------------------------------------  406
Csu1   --------------------------------------------------------------------------------  426
Ahy1   --------------------------------------------------------------------------------  403
Plu1   --------------------------------------------------------------------------------  481
Cla1   --------------------------------------------------------------------------------  387
Pas1   --------------------------------------------------------------------------------  363
ruler  ......1690......1700......1710......1720......1730......1740......1750......1760
```

```
  Eca1 --------------------------------------------------------------------------------   53
  Esp2 --------------------------------------------------------------------------------   53
  Cko1 --------------------------------------------------------------------------------   53
  Sen1 --------------------------------------------------------------------------------   53
  Eco3 --------------------------------------------------------------------------------   49
  Esa3 --------------------------------------------------------------------------------   54
  Kpn1 --------------------------------------------------------------------------------   51
  Pan1 --------------------------------------------------------------------------------   61
  Eta2 --------------------------------------------------------------------------------   52
  Spr1 --------------------------------------------------------------------------------   69
 Eco25 NIAPDASNAQVTLNIPAQQVVTNNSDSVQLTATVKDPSNHPVAGITVNFTMPQDVAANFTLENNGIAITQANGEAHVTLK  722
 Eco15 --------------------------------------------------------------------------------  416
  Yfr1 --------------------------------------------------------------------------------  296
  Yfr5 --------------------------------------------------------------------------------  345
  Yps4 FVPGAVATITLSVLVNDATADGADTNQVDALVQDANGNAITGAAVVFSSANGADIIAPTMNTGVNGVASTLLTHTQSGVS 1087
  Efe2 --------------------------------------------------------------------------------  424
  Efe3 ISDGDISSEPYLIIAGDVNAGQSALSANPTSIEANNKEMPSTLTYKAMDAWKNPVTGLTDNNSLTLKVTGLGGTEFSGFT  524
  Eta1 --------------------------------------------------------------------------------  336
 Eco26 --------------------------------------------------------------------------------  465
  Pru1 -------------------------------------------------------------------------------- 1186
  Sgl1 --------------------------------------------------------------------------------  447
  Pmi1 NNPVSKGVTVGWISDLGKLAAPLSMTNKQGIAEITLSSTQAGKAKVTAVLNNQQSVNADHIVTFTEGDISASLSTVAIFP  853
  Ymo1 --------------------------------------------------------------------------------  251
  Yen2 LKFTLRDANHNPVSGLKQRIDVTQSVASHVTIGAVTETTVKGVYQAAITGMKENSVDLTASVKGTNVRQTRTLTLQADNK  875
  Sen2 --------------------------------------------------------------------------------  269
  Efe4 --------------------------------------------------------------------------------  263
  Bpe1 --------------------------------------------------------------------------------  457
  Bav2 --------------------------------------------------------------------------------  497
  Bpa2 --------------------------------------------------------------------------------  499
  Bav1 --------------------------------------------------------------------------------  347
  Yin1 --------------------------------------------------------------------------------  269
  Yfr4 --------------------------------------------------------------------------------  269
  Yin2 --------------------------------------------------------------------------------  270
  Ymo2 --------------------------------------------------------------------------------  289
 Eco10 --------------------------------------------------------------------------------  291
  Eal1 --------------------------------------------------------------------------------  290
 Eco16 --------------------------------------------------------------------------------  295
  Sty4 --------------------------------------------------------------------------------  290
  Yfr2 --------------------------------------------------------------------------------  443
  Ype5 --------------------------------------------------------------------------------  410
  Eco6 TVAPVVTTQPDGTVEISVTSQTAGSSAVTVSINSSSQSRDVTFIADVRTAQIADLVVIKDDSVADGAMANMLRARVTDVF  886
  Bbr1 --------------------------------------------------------------------------------  348
  Yps2 --------------------------------------------------------------------------------  364
 Eco14 --------------------------------------------------------------------------------  380
  Sen3 --------------------------------------------------------------------------------  448
  Pal3 --------------------------------------------------------------------------------   86
  Ybe1 --------------------------------------------------------------------------------  350
  Pal2 ALTGLQSGDTLDLTVMAKDAFKNPLTGLASAIVLTHGQTGSVVWKDHHDGIYTTSLPLTKLGSDSLTATINTIASQPISI  887
  Eco1 --------------------------------------------------------------------------------  420
  Yps7 --------------------------------------------------------------------------------  400
  Yfr3 --------------------------------------------------------------------------------  425
 Eco20 --------------------------------------------------------------------------------  424
  Efe5 --------------------------------------------------------------------------------  406
  Csu1 --------------------------------------------------------------------------------  426
  Ahy1 --------------------------------------------------------------------------------  403
  Plu1 --------------------------------------------------------------------------------  481
  Cla1 --------------------------------------------------------------------------------  387
  Pas1 --------------------------------------------------------------------------------  363
 ruler ......1770......1780......1790......1800......1810......1820......1830......1840
```

```
 Eca1 --------------------------------------------------------------------------------   53
 Esp2 --------------------------------------------------------------------------------   53
 Cko1 --------------------------------------------------------------------------------   53
 Sen1 --------------------------------------------------------------------------------   53
 Eco3 --------------------------------------------------------------------------------   49
 Esa3 --------------------------------------------------------------------------------   54
 Kpn1 --------------------------------------------------------------------------------   51
 Pan1 --------------------------------------------------------------------------------   61
 Eta2 --------------------------------------------------------------------------------   52
 Spr1 --------------------------------------------------------------------------------   69
Eco25 GKKAGTHTVTATLGNNNASDAQPVTFVADKDSAVVVLQTSKAEIIGNGVDETTLTATVKDPFDNAVKDLQVTFSTNPADT  802
Eco15 SSKAGSYSVDASL---------------------------------------------------------------EVDK  433
 Yfr1 --------------------------------------------------------------------------------  296
 Yfr5 --------------------------------------------------------------------------------  345
 Yps4 NVVATIDTVNANIDTTFVAG----------AVAAITLTTPVDGAVADGTDSNSVQAVVSDSEGNAVAGAAVVFSSANATA 1157
 Efe2 --------------------------------------------------------------------------------  424
 Efe3 DKGEGIYTGTLTGTQSGQASL---------MPQVNNVDMAPDVTSVTLTAGKAVAKNSRINVGAANFTAGTRFTVTVTLR  595
 Eta1 --------------------------------------------------------------------------------  336
Eco26 --------------------------------------------------------------------------------  465
 Pru1 -------------------------------------------------------------------------------- 1186
 Sgl1 --------------------------------------------------------------------------------  447
 Pmi1 DTIVAGANKATVTITLKDKEGNLLPNLANKITLTPQTNLQTTITPFKEKQKGIYQAQVSALKTGKTSLSAHVAPLAIKQS  933
 Ymo1 --------------------------------------------------------------------------------  251
 Yen2 TATLKTVTSNIKTAKADGKESITYRAKVIDAQGNASLDNVSVGWRTTLGELAAITKTDTSGIATVTLTSKQAGSATVTAI  955
 Sen2 --------------------------------------------------------------------------------  269
 Efe4 --------------------------------------------------------------------------------  263
 Bpe1 --------------------------------------------------------------------------------  457
 Bav2 --------------------------------------------------------------------------------  497
 Bpa2 --------------------------------------------------------------------------------  499
 Bav1 --------------------------------------------------------------------------------  347
 Yin1 --------------------------------------------------------------------------------  269
 Yfr4 --------------------------------------------------------------------------------  269
 Yin2 --------------------------------------------------------------------------------  270
 Ymo2 --------------------------------------------------------------------------------  289
Eco10 --------------------------------------------------------------------------------  291
 Eal1 --------------------------------------------------------------------------------  290
Eco16 --------------------------------------------------------------------------------  295
 Sty4 --------------------------------------------------------------------------------  290
 Yfr2 --------------------------------------------------------------------------------  443
 Ype5 --------------------------------------------------------------------------------  410
 Eco6 GNALAGQTVSVMADNGAAVASTMTTKPDGTVEISVTSQTAGVSAVTATINSSTQSQNVTFIADVRTAKIADLVVIKDGSE  966
 Bbr1 --------------------------------------------------------------------------------  348
 Yps2 --------------------------------------------------------------------------------  364
Eco14 --------------------------------------------------------------------------------  380
 Sen3 --------------------------------------------------------------------------------  448
 Pal3 --------------------------------------------------------------------------------   86
 Ybe1 --------------------------------------------------------------------------------  350
 Pal2 TVETQQTKTSVFDIELHAQHPLISAGQSTTLTLTLRDKYNNNVEKIPSSDIKLEDNKLTLSNIQWKEQGNGVYTTEISFK  967
 Eco1 --------------------------------------------------------------------------------  420
 Yps7 --------------------------------------------------------------------------------  400
 Yfr3 --------------------------------------------------------------------------------  425
Eco20 --------------------------------------------------------------------------------  424
 Efe5 --------------------------------------------------------------------------------  406
 Csu1 --------------------------------------------------------------------------------  426
 Ahy1 --------------------------------------------------------------------------------  403
 Plu1 --------------------------------------------------------------------------------  481
 Cla1 --------------------------------------------------------------------------------  387
 Pas1 --------------------------------------------------------------------------------  363
ruler ......1850......1860......1870......1880......1890......1900......1910......1920
```

```
  Eca1 --------------------------------------------------------------------------------   53
  Esp2 --------------------------------------------------------------------------------   53
  Cko1 --------------------------------------------------------------------------------   53
  Sen1 --------------------------------------------------------------------------------   53
  Eco3 --------------------------------------------------------------------------------   49
  Esa3 --------------------------------------------------------------------------------   54
  Kpn1 --------------------------------------------------------------------------------   51
  Pan1 --------------------------------------------------------------------------------   61
  Eta2 --------------------------------------------------------------------------------   52
  Spr1 --------------------------------------------------------------------------------   69
 Eco25 QLSQSKSNTNDSGVAEVTFKGTVLGVHTAEATLPNGNNDTKIVNIAPDASNAQVTLNIPAQQVVTNNSDSVQLTATVKDP  882
 Eco15 NIHQSVT-----------------------------------ITVVPNREQSVMTLNAGSGSAIANNTNIVTLTASVKDV  478
  Yfr1 --------------------------------------------------------------------------------  296
  Yfr5 --------------------------------------------------------------------------------  345
  Yps4 QITTVIGTTGADGIATATLTNTVAGTSNVAATIGSITDN--IDTVFVAGAVATITLSVPVNDATADGADTNQVDALVQDV 1235
  Efe2 ------------------------------------GEYTIEYIADSVGNGLMASLKLMSWKNE--KTATYNIT-AQPRN  465
  Efe3 DASENPVTGAEDLLTNETVKVPGATRKGAEWTGNGDGTYRAEWEAEQAGNGYKATLHLAGWGDEEMQSAPYNIQPGEPTE  675
  Eta1 --------------------------------------------------------------------------------  336
 Eco26 --------------------------------------------------------------------------------  465
  Pru1 -------------------------------------------------------------------------------- 1186
  Sgl1 --------------------------------------------------------------------------------  447
  Pmi1 VSLTVLPNNTTAKVKNFTISNIQPHAGESITYKAYLVDNHDNPVGMGVPVAWSTNEGSQLETPLTFTDDNGVAIVGLSRR 1013
  Ymo1 --------------------------------------------------------------------------------  251
  Yen2 VSSTSEMKAAPVNFTAGGISITQSTASLSVKDLVADDVITTKLTVNIKDDNGNPLTGKGSEISVTATGLAGLKLPTTFVE 1035
  Sen2 --------------------------------------------------------------------------------  269
  Efe4 --------------------------------------------------------------------------------  263
  Bpe1 --------------------------------------------------------------------------------  457
  Bav2 --------------------------------------------------------------------------------  497
  Bpa2 --------------------------------------------------------------------------------  499
  Bav1 --------------------------------------------------------------------------------  347
  Yin1 --------------------------------------------------------------------------------  269
  Yfr4 --------------------------------------------------------------------------------  269
  Yin2 --------------------------------------------------------------------------------  270
  Ymo2 --------------------------------------------------------------------------------  289
 Eco10 --------------------------------------------------------------------------------  291
  Eal1 --------------------------------------------------------------------------------  290
 Eco16 --------------------------------------------------------------------------------  295
  Sty4 --------------------------------------------------------------------------------  290
  Yfr2 --------------------------------------------------------------------------------  443
  Ype5 --------------------------------------------------------------------------------  410
  Eco6 ADGSTANTLRARVTDAFGNALAGQTVSVLAGNGATTAPTVTTQPDGTVEISVTSQTAGISAVTASINNSSQSRNVTFIAD 1046
  Bbr1 --------------------------------------------------------------------------------  348
  Yps2 --------------------------------------------------------------------------------  364
 Eco14 --------------------------------------------------------------------------------  380
  Sen3 --------------------------------------------------------------------------------  448
  Pal3 --------------------------------------------------------------------------------   86
  Ybe1 --------------------------------------------------------------------------------  350
  Pal2 QLGNHKLVSTVGFTNSPSIDIDVIALKGAIHVHHVNLDATPKQFVVGEKIQLKLTLLDQFNNGVTDVLDNSININDDNTR 1047
  Eco1 --------------------------------------------------------------------------------  420
  Yps7 --------------------------------------------------------------------------------  400
  Yfr3 --------------------------------------------------------------------------------  425
 Eco20 --------------------------------------------------------------------------------  424
  Efe5 --------------------------------------------------------------------------------  406
  Csu1 --------------------------------------------------------------------------------  426
  Ahy1 --------------------------------------------------------------------------------  403
  Plu1 --------------------------------------------------------------------------------  481
  Cla1 --------------------------------------------------------------------------------  387
  Pas1 --------------------------------------------------------------------------------  363
 ruler ......1930......1940......1950......1960......1970......1980......1990......2000
```

```
Eca1   --------------------------------------------------------------------------------   53
Esp2   --------------------------------------------------------------------------------   53
Cko1   --------------------------------------------------------------------------------   53
Sen1   --------------------------------------------------------------------------------   53
Eco3   --------------------------------------------------------------------------------   49
Esa3   --------------------------------------------------------------------------------   54
Kpn1   --------------------------------------------------------------------------------   51
Pan1   --------------------------------------------------------------------------------   61
Eta2   --------------------------------------------------------------------------------   52
Spr1   --------------------------------------------------------------------------------   69
Eco25  SNHPVAGITVNFTMP-------------------QDVAANFTLENNGIAITQANGEAHVTLKGKKAGTHTVTATLSNNNT  943
Eco15  YGHPLPDEDVKFTLP-------------------ASMTGNFTLSS-ETARTDANGDAVVTLRGTKAGEFTVTATLTRNNT  538
Yfr1   --------------------------------------------------------------------------------  296
Yfr5   --------------------------------------------------------------------------------  345
Yps4   NGNAITGAAVVFSSANGATILSSTVNTGADGIASTTLTHTQSGVSNVVATIDTVNANIDTTFVAGAVATITLSVLVNDAT 1315
Efe2   MDGSIIVGGVGFAS---------------SQTP------------------------------------RFPTTGFRG--  492
Efe3   AQSHISTDGTIFTTGDAIPVTVTLKDAWGNHTPGQASYLSTHVVAAHTVPQPTQWQENSDGTYSAQFIAGEPGTGLQVTL  755
Eta1   --------------------------------------------------------------------------------  336
Eco26  --------------------------------------------------------------------------------  465
Pru1   -------------------------------------------------------------------------------- 1186
Sgl1   --------------------------------------------------------------------------------  447
Pmi1   SVGVAKVSAILETGTYIADDVHFLTGHIDETMSELSLNPSQIIANGKDKALLTFVIKDKNGNIIPNQQVSGFSKNPTIKF 1093
Ymo1   --------------------------------------------------------------------------------  251
Yen2   GPNGVYTATITGTKAGVGDIVTALAGKELAKQQLKVIADVQTAKIADIKPLKSGSVSVGDKVTYQATLKDANDNLLGAGI 1115
Sen2   --------------------------------------------------------------------------------  269
Efe4   --------------------------------------------------------------------------------  263
Bpe1   --------------------------------------------------------------------------------  457
Bav2   ----------------------------------------------------------------------SYTASSTRDV  507
Bpa2   ----------------------------------------------------------------------RYRAVSPGDV  509
Bav1   --------------------------------------------------------------------------------  347
Yin1   --------------------------------------------------------------------------------  269
Yfr4   --------------------------------------------------------------------------------  269
Yin2   --------------------------------------------------------------------------------  270
Ymo2   --------------------------------------------------------------------------------  289
Eco10  --------------------------------------------------------------------------------  291
Eal1   --------------------------------------------------------------------------------  290
Eco16  --------------------------------------------------------------------------------  295
Sty4   --------------------------------------------------------------------------------  290
Yfr2   --------------------------------------------------------------------------------  443
Ype5   --------------------------------------------------------------------------------  410
Eco6   VRTAKIADLVVIKDGSEADGSTANTLRARVTDAFGNTLAGQTVSVMADNGATTAPTVITEPDGTVEISVTSQTAGTSTVT 1126
Bbr1   --------------------------------------------------------------------------------  348
Yps2   --------------------------------------------------------------------------------  364
Eco14  --------------------------------------------------------------------------------  380
Sen3   --------------------------------------------------------------------------------  448
Pal3   --------------------------------------------------------------------------------   86
Ybe1   --------------------------------------------------------------------------------  350
Pal2   SALNGLQWHHQQKGVYTAETTVTKGGIHSLKATVNSKQDNVLIHAISSVGQKQVSDVQLSTSTASIVAGTPATLILKLND 1127
Eco1   --------------------------------------------------------------------------------  420
Yps7   --------------------------------------------------------------------------------  400
Yfr3   --------------------------------------------------------------------------------  425
Eco20  --------------------------------------------------------------------------------  424
Efe5   --------------------------------------------------------------------------------  406
Csu1   --------------------------------------------------------------------------------  426
Ahy1   --------------------------------------------------------------------------------  403
Plu1   --------------------------------------------------------------------------------  481
Cla1   --------------------------------------------------------------------------------  387
Pas1   --------------------------------------------------------------------------------  363
ruler  ......2010......2020......2030......2040......2050......2060......2070......2080
```

```
Eca1   --------------------------------------------------------------------------------   53
Esp2   --------------------------------------------------------------------------------   53
Cko1   --------------------------------------------------------------------------------   53
Sen1   --------------------------------------------------------------------------------   53
Eco3   --------------------------------------------------------------------------------   49
Esa3   --------------------------------------------------------------------------------   54
Kpn1   --------------------------------------------------------------------------------   51
Pan1   --------------------------------------------------------------------------------   61
Eta2   --------------------------------------------------------------------------------   52
Spr1   --------------------------------------------------------------------------------   69
Eco25  SDSQPVTFVADKTSALVVLQISKNEITGNGVDSATLTATVKDQFDNEVNNLPVTFSTASSGLTLTPGESNTNESGIAQAT   1023
Eco15  VAYQQVT-------------------------------------------------------------------------   545
Yfr1   --------------------------------------------------------------------------------   296
Yfr5   --------------------------------------------------------------------------------   345
Yps4   ADGADTNQVDALVQDANGNAITGAAVVFSSANGATIIVPTMNTGANGVASTLLTHTVAGTSNVVATIGSITNNIDTAFVA   1395
Efe2   ---------ATFTITG--------------------NNFIVGVNNYN---------------------------------   510
Efe3   NQDSGWKVAAIYSIIAGEPAINNSFVDTDKNNYQAGNNMLISVKLADSRGNALSGREAILNDAVAVGSATRKEGSVWTED   835
Eta1   --------------------------------------------------------------------------------   336
Eco26  --------------------------------------------------------------------------------   465
Pru1   --------------------------------------------------------------------------------   1186
Sgl1   --------------------------------------------------------------------------------   447
Pmi1   SQAQQISPGRYEIEITGTQSGTAQIGVMVNGTHFKKQKILQLNADVTTWKIRA---------------------------   1146
Ymo1   --------------------------------------------------------------------------------   251
Yen2   PVHWSVNRDTLMSGKLISLTNSAGVAEVEISRDLAGDALVTAAVGNNSLQATAVKFISGGVDISKSSMQLLQGNITADNL   1195
Sen2   --------------------------------------------------------------------------------   269
Efe4   --------------------------------------------------------------------------------   263
Bpe1   --------------------------------------------------------------------------------   457
Bav2   TQS-----------------------------------------------------------------------------   510
Bpa2   LRSGTVRVIATDAHGNASAPRDVPYQDAVDRTAPPLTIEAAVEQARTGVVNVSGKTEPNLMVSATFPDGQTAQARADGQG   589
Bav1   --------------------------------------------------------------------------------   347
Yin1   --------------------------------------------------------------------------------   269
Yfr4   --------------------------------------------------------------------------------   269
Yin2   --------------------------------------------------------------------------------   270
Ymo2   --------------------------------------------------------------------------------   289
Eco10  --------------------------------------------------------------------------------   291
Eal1   --------------------------------------------------------------------------------   290
Eco16  --------------------------------------------------------------------------------   295
Sty4   --------------------------------------------------------------------------------   290
Yfr2   --------------------------------------------------------------------------------   443
Ype5   --------------------------------------------------------------------------------   410
Eco6   ASINNSSQSRNVTFIADVRTAKIADLVVTRDNSVADGAMANTLQVKVTDANGNTLAGQTVSVLADNSATTAPTVITEPDG   1206
Bbr1   --------------------------------------------------------------------------------   348
Yps2   --------------------------------------------------------------------------------   364
Eco14  --------------------------------------------------------------------------------   380
Sen3   --------------------------------------------------------------------------------   448
Pal3   --------------------------------------------------------------------------------   86
Ybe1   --------------------------------------------------------------------------------   350
Pal2   QHSNPVSQVNSADISLENSAGTPLPTTWLEMNNLGIYTAQISLNSVGKQTVTVKVNNISHNATLQVTPPQGASSVATLEI   1207
Eco1   --------------------------------------------------------------------------------   420
Yps7   --------------------------------------------------------------------------------   400
Yfr3   --------------------------------------------------------------------------------   425
Eco20  --------------------------------------------------------------------------------   424
Efe5   --------------------------------------------------------------------------------   406
Csu1   --------------------------------------------------------------------------------   426
Ahy1   --------------------------------------------------------------------------------   403
Plu1   --------------------------------------------------------------------------------   481
Cla1   --------------------------------------------------------------------------------   387
Pas1   --------------------------------------------------------------------------------   363
ruler  ......2090......2100......2110......2120......2130......2140......2150......2160
```

```
  Eca1 --------------------------------------------------------------------------------   53
  Esp2 --------------------------------------------------------------------------------   53
  Cko1 --------------------------------------------------------------------------------   53
  Sen1 --------------------------------------------------------------------------------   53
  Eco3 --------------------------------------------------------------------------------   49
  Esa3 --------------------------------------------------------------------------------   54
  Kpn1 --------------------------------------------------------------------------------   51
  Pan1 --------------------------------------------------------------------------------   61
  Eta2 --------------------------------------------------------------------------------   52
  Spr1 --------------------------------------------------------------------------------   69
 Eco25 LAGVAFGEQTVTASLANNGASDNKTVHFIGDTAAAKII------------------------------------------ 1061
 Eco15 ---------------------------FIGDTNSAQLQ------------------------------------------  556
  Yfr1 --------------------------------------------------------------------------------  296
  Yfr5 --------------------------------------------------------------------------------  345
  Yps4 GAVATITLTTPVNGAVADGANSNSVQAVVSDSEGNAVA------------------------------------------ 1433
  Efe2 --------------------LRVTCGDKTKDCDWVTVN------------------------------------------  528
  Efe3 TQHKGTYTAYYMAQHAGQDSVKLTLDDGAKSSDAYTIV------------------------------------------  873
  Eta1 --------------------------------------------------------------------------------  336
 Eco26 --------------------------------------------------------------------------------  465
  Pru1 -------------------------------------------------------------------------------- 1186
  Sgl1 --------------------------------------------------------------------------------  447
  Pmi1 -------------------------------------------------------------------------------- 1146
  Ymo1 --------------------------------------------------------------------------------  251
  Yen2 DIATIQVDIRDSKGNPLPNLASQITTSPKKGEHGLKIETIANPSGDGYLVKMKGTQAGNHTVTVSVAGKPLSAKVDMVLK 1275
  Sen2 --------------------------------------------------------------------------------  269
  Efe4 --------------------------------------------------------------------------------  263
  Bpe1 --------------------------------------------------------------------------------  457
  Bav2 --------------------------------------------------------------------------------  510
  Bpa2 RYQLASPADVARSGRIAVSASDAAGNRAAASAEFTDQVDKTAPRAPTLQLATDTATGRVTASGKAEPGAIVEITWPDQTR  669
  Bav1 --------------------------------------------------------------------------------  347
  Yin1 --------------------------------------------------------------------------------  269
  Yfr4 --------------------------------------------------------------------------------  269
  Yin2 --------------------------------------------------------------------------------  270
  Ymo2 --------------------------------------------------------------------------------  289
 Eco10 --------------------------------------------------------------------------------  291
  Eal1 --------------------------------------------------------------------------------  290
 Eco16 --------------------------------------------------------------------------------  295
  Sty4 --------------------------------------------------------------------------------  290
  Yfr2 --------------------------------------------------------------------------------  443
  Ype5 --------------------------------------------------------------------------------  410
  Eco6 MVEISVTSQTAGTSAVTASINNSSLSQSVKFIADVSTAQIAMLEVTQDNAVADGAMANTLQVKVTDAFGNALSGQTVSVL 1286
  Bbr1 --------------------------------------------------------------------------------  348
  Yps2 --------------------------------------------------------------------------------  364
 Eco14 --------------------------------------------------------------------------------  380
  Sen3 --------------------------------------------------------------------------------  448
  Pal3 --------------------------------------------------------------------------------   86
  Ybe1 --------------------------------------------------------------------------------  350
  Pal2 SPITTVDAGHTSSITLIMKDKYGNPVNNVLNRDMTLEIDGVIQSIQLTELGDTGQYSG---------------------- 1265
  Eco1 --------------------------------------------------------------------------------  420
  Yps7 --------------------------------------------------------------------------------  400
  Yfr3 --------------------------------------------------------------------------------  425
 Eco20 --------------------------------------------------------------------------------  424
  Efe5 --------------------------------------------------------------------------------  406
  Csu1 --------------------------------------------------------------------------------  426
  Ahy1 --------------------------------------------------------------------------------  403
  Plu1 --------------------------------------------------------------------------------  481
  Cla1 --------------------------------------------------------------------------------  387
  Pas1 --------------------------------------------------------------------------------  363
 ruler ......2170......2180......2190......2200......2210......2220......2230......2240
```

```
Eca1   --------------------------------------------------------------------------------   53
Esp2   --------------------------------------------------------------------------------   53
Cko1   --------------------------------------------------------------------------------   53
Sen1   --------------------------------------------------------------------------------   53
Eco3   --------------------------------------------------------------------------------   49
Esa3   --------------------------------------------------------------------------------   54
Kpn1   --------------------------------------------------------------------------------   51
Pan1   --------------------------------------------------------------------------------   61
Eta2   --------------------------------------------------------------------------------   52
Spr1   --------------------------------------------------------------------------------   69
Eco25  ------ELTPVPDSIIAGTPQNSSGSVITATVVDNNGFPVKGVTVNFTSNAATAEMTNGGQAVTNEQGKATVTYTNTRSS   1135
Eco15  ------PLTASLNSIVAG---NSTGSTLTATILDAYQNPLKDQLVTFQSNDVTLSET---EVTTNTLGQATVTMT---SN   621
Yfr1   --------------------------------------------------------------------------------   296
Yfr5   --------------------------------------------------------------------------------   345
Yps4   ------GAAVVFSSANATAQITTVIGTTGADGIATATLTNTVAGTSNVVATIGSITDNIDTVFVAGAVATITLTTPVNGA   1507
Efe2   ------KQS----DSAIVK-----------------------FISNPTSGSK------VRITAIPTVEG--------------   558
Efe3   ------AAAPVDKNSAIQRGEAATYTAGDTLKLTVTLQDNWGNPVSGMENVLEGSVTLPEAEIQESGWQQTSPGVYEANW   947
Eta1   --------------------------------------------------------------------------------   336
Eco26  ------------------------------LVKDKHGNILPGVIVSWQLNGNSESFAPVSRTNAEGIATTTVRSNTAGEL   515
Pru1   ------------------------------TVVDQFNNPLPSIHVSWHLQGKAEPFAHSTVTNAQGVAHVKVTSHQMGQL   1236
Sgl1   --------------------------------------------------------------------------------   447
Pmi1   -----------VEVDRTTITAGDKGVNYQATVVDANNNVLPNVIVSWKLLGSADDYHYSTYTNDKGIATNRVTSHVAGRL   1215
Ymo1   -----------KVNRTTFKADETDQVTYTAKVVDINNNPLENFPVSWRLTQGEGQYKSLSYTGPTGEAETKLSASRLGQY   320
Yen2   GDATTAKIESVKSSSPTFKADNVDTVTYTAKVVDANNNLLENIAVSWRLAQGEGQYQGQSYTGKTGVATTKLSASRLGTY   1355
Sen2   --------------------------------------------------------------------------------   269
Efe4   --------------------------------------------------------------------------------   263
Bpe1   ------------------------------------------------------TITNVTTDAASGRITVTGMAEPGANV   483
Bav2   ---------------------GDITATARDAAGNVSAPATQAYNDIVDKTPPPVSITNVSTAPGSGIVTVTGVSEPGASV   569
Bpa2   VTVVADTSGAYSATSAADMPDGAITAVAADRAGNTGAPTRQRYIDSVDRTAPTLGTPSVSTAADTGRVSVTGMTEPGARV   749
Bav1   --------------------------------------------------------------------------------   347
Yin1   --------------------------------------------------------------------------------   269
Yfr4   --------------------------------------------------------------------------------   269
Yin2   --------------------------------------------------------------------------------   270
Ymo2   --------------------------------------------------------------------------------   289
Eco10  --------------------------------------------------------------------------------   291
Eal1   --------------------------------------------------------------------------------   290
Eco16  --------------------------------------------------------------------------------   295
Sty4   --------------------------------------------------------------------------------   290
Yfr2   --------------------------------------------------------------------------------   443
Ype5   --------------------------------------------------------------------------------   410
Eco6   AGNGATVAPTVITEPDGTAEIPVTSQTAGVSAVTATINNSSQSRNVTFVADVRTAQIADLVVIKDGSEADGATANTLRAR   1366
Bbr1   --------------------------------------------------------------------------------   348
Yps2   --------------------------------------------------------------------------------   364
Eco14  --------------------------------------------------------------------------------   380
Sen3   --------------KSTALANGTDSITFTGTVIDANNNVLEGVNVAWAVTPTTGVLSTTTSASGSDGKASVSLTSSQVES   514
Pal3   --------------------------------------------------------------------------------   86
Ybe1   --------------------------------------------------------------------------------   350
Pal2   -----ELPAQQKGQHAVKVTVNGQLASVNWTINNPMPIPLSTVDRTGQRGALDTIMLSGSKKMVDSGDKITVTLNLMDKF   1340
Eco1   --------------------------------------------------------------------------------   420
Yps7   --------------------------------------------------------------------------------   400
Yfr3   --------------------------------------------------------------------------------   425
Eco20  --------------------------------------------------------------------------------   424
Efe5   --------------------------------------------------------------------------------   406
Csu1   --------------------------------------------------------------------------------   426
Ahy1   --------------------------------------------------------------------------------   403
Plu1   -----------KDKAHYNNGSDSYTFTATVKDGHGNLVVGQPVNIDWQTDPKADGLKLTKQNNSVSNAQGQVTATLISTA   550
Cla1   --------------------------------------------------------------------------------   387
Pas1   --------------------------------------------------------------------------------   363
ruler  ......2250......2260......2270......2280......2290......2300......2310......2320
```

```
 Eca1 --------------------------------------------------------------------------------   53
 Esp2 --------------------------------------------------------------------------------   53
 Cko1 --------------------------------------------------------------------------------   53
 Sen1 --------------------------------------------------------------------------------   53
 Eco3 --------------------------------------------------------------------------------   49
 Esa3 --------------------------------------------------------------------------------   54
 Kpn1 --------------------------------------------------------------------------------   51
 Pan1 --------------------------------------------------------------------------------   61
 Eta2 --------------------------------------------------------------------------------   52
 Spr1 --------------------------------------------------------------------------------   69
Eco25 IESGARPDTVEASLENGSSTLSTSINVNADASTAHLTLLQALFDTVSAG------------------------------- 1184
Eco15 IAG--QHNVVVSRKAQASDNKTFSLSVLPDESSAKVISITGAEKTITVG-------------------------------  668
 Yfr1 --------------------------------------------------------------------------------  296
 Yfr5 --------------------------------------------------------------------------------  345
 Yps4 VADGANSNSVQAVVSDSEGNPVTGATVVFSSSNATAQITTVIGTTGADGIATATLTNTVAGTSNVVATIDTVNANIDTTF 1587
 Efe2 -----ALTLMYEFQIRKWFKPDS---------------------------------------------------------  576
 Efe3 TARIAGPSLTAKLELEGWRKETAPFVIVAAGPDYENSSLRTSNFAFTAG-------------------------------  996
 Eta1 --------------------------------------------------------------------------------  336
Eco26 KMRAYL-------DEANYKDAANVTVIAGDIDSKNSDFSLSKYNIGADGI------------------------------  558
 Pru1 VMTAVL-------SSSQNKVATPVQVNAGAVNSQNSTFTASKMDIGPDGI------------------------------ 1279
 Sgl1 --------------------------------------------------------------------------------  447
 Pmi1 KMSAYL-------DSNNYKSTQDITVIPAEIDIHKSTFNSHRRSINADNK------------------------------ 1258
 Ymo1 KMEAQIGILNGSPTRIVEKAAPDVSTTAGDIDPSKSDLVVDVNSIDASGK------------------------------  370
 Yen2 KMEAQV--------RQQVKAAAGVNSTAGDADPSQSDFVVDVASIDSSGN------------------------------ 1397
 Sen2 --------------------------------------------------------------------------------  269
 Efe4 --------------------------------------------------------------------------------  263
 Bpe1 AVNFP---------------------------------------------------------------------------  488
 Bav2 VVNFPGRGTARAEAAPDGGYTVSST-GPVESGAIQAVATDSHDNHSEPVRR---DYLDTTAPAAPVISEVTAGPTGLVTV  645
 Bpa2 QVEIPGEAPRIVTADAAGRYSVVSAGDVLQSGTVRVTATDAHGNASAPAHLRYQDQVDKTAPASPSLTVREDAASGRATV  829
 Bav1 --------------------------------------------------------------------------------  347
 Yin1 --------------------------------------------------------------------------------  269
 Yfr4 --------------------------------------------------------------------------------  269
 Yin2 --------------------------------------------------------------------------------  270
 Ymo2 --------------------------------------------------------------------------------  289
Eco10 --------------------------------------------------------------------------------  291
 Eal1 --------------------------------------------------------------------------------  290
Eco16 --------------------------------------------------------------------------------  295
 Sty4 --------------------------------------------------------------------------------  290
 Yfr2 --------------------------------------------------------------------------------  443
 Ype5 --------------------------------------------------------------------------------  410
 Eco6 VTDAFGNALAGQTVSVLADNGATVAPTVTTQPDGTVEISVTSQTAGISAVTATINNSTASQNVTFIADVRTAQIADLVVI 1446
 Bbr1 --------------------------------------------------------------------------------  348
 Yps2 --------------------------------------------------------------------------------  364
Eco14 --------------------------------------------------------------------------------  380
 Sen3 YQVTATVNGKDETSGNVSFTADSASASLTSLTSDETSNLIAGNG------------------------------------  558
 Pal3 --------------------------------------------------------------------------------   86
 Ybe1 --------------------------------------------------------------------------------  350
 Pal2 NNPLTGANSHLKLLTNLSEISQWQDHSDGSYSIDLLMNRLGSQDVQAIVKNILSNKVTLEIKALSGASNVNTTALTIKDA 1420
 Eco1 --------------------------------------------------------------------------------  420
 Yps7 --------------------------------------------------------------------------------  400
 Yfr3 --------------------------------------------------------------------------------  425
Eco20 --------------------------------------------------------------------------------  424
 Efe5 --------------------------------------------------------------------------------  406
 Csu1 --------------------------------------------------------------------------------  426
 Ahy1 --------------------------------------------------------------------------------  403
 Plu1 AVKDVQVSAKAVANPSWVEADRKVSFEELSLSYQVTGVTVEVDKDEKRYNNG----------------------------  602
 Cla1 --------------------------------------------------------------------------------  387
 Pas1 --------------------------------------------------------------------------------  363
ruler ......2330......2340......2350......2360......2370......2380......2390......2400
```

```
Eca1   --------------------------------------------------------------------------------   53
Esp2   --------------------------------------------------------------------------------   53
Cko1   --------------------------------------------------------------------------------   53
Sen1   --------------------------------------------------------------------------------   53
Eco3   --------------------------------------------------------------------------------   49
Esa3   --------------------------------------------------------------------------------   54
Kpn1   --------------------------------------------------------------------------------   51
Pan1   --------------------------------------------------------------------------------   61
Eta2   --------------------------------------------------------------------------------   52
Spr1   --------------------------------------------------------------------------------   69
Eco25  -----DTTNLYIEVKDNYGNGVPQQEVTLSVSPSEGVTPSNNAIYTTNHDGNFYASFTATKAGVYQVTATLENGDSMQQT 1259
Eco15  -----ENITLRILVQDAFNNVIAGQRVRLSAQPTTNITIGDTAY--TDNNGYAYVNLLSTQPGVYQVTATLDN-------  734
Yfr1   --------------------------------------------------------------------------------  296
Yfr5   --------------------------------------------------------------------------------  345
Yps4   VPGAVATITLTTPVDGAVADGANSNSVQAVVTDSGGNPVTGAAVVFSSANATAQITTVIGTTGADGIATATLTNTVAGTS 1667
Efe2   ---------------NSIGNPVGLVDSCIT-ANGKVP---------------------EKSELNN---------------  604
Efe3   -----DDITITVILMDANENPVTGAEGLLTNETVKVPGATRKGTVWTDKGDGTYRAEWEAEQAGNGYKATLHLAGWGDEE 1071
Eta1   --------------------------------------------------------------------------------  336
Eco26  -----ETSTLTLILKDKYGNSIPGKIVTVNAKTKTGQLSLDNNPMKEVGDGIYISNAKSSYQGRFELTLDINGNKFSRSQ  633
Pru1   -----EETILTIKLQDDFGNPLSNEIVTINSNAPNTDFTIARITNNQ--DGSYTSTATATKQGTVRLTARVGSDIIANPL 1352
Sgl1   --------------------------------------------------------------------------------  447
Pmi1   -----DSTLLSVKLMDKYGNTIDGKNVEIKTISGKPNFSDNPLKSVG--NGEYQTNVTANTMSDIILTAQAETITIAEPL 1331
Ymo1   -----TKAKLTATLKDKFGNLLKGQTVNVKETQNLTGIKFSVNPMKDNGDGTYSTEVTSTTKGNTTFIASINSVDLTQQP  445
Yen2   -----TKAKLTATLKDKFGNLLSGQKVKLTDSNSLKKITLSANPMKDNGDGTYSTEVTATAKGNTRFIARVNGVDLTQQP 1472
Sen2   --------------------------------------------------------------------------------  269
Efe4   --------------------------------------------------------------------------------  263
Bpe1   ---------------DGTRKTVVADGAGAYAADSDGDMVAGDIHVQATDKAGNRSPEGTRAYVDTVDKTPSAAP------  547
Bav2   EGRAEVGSTVTVTFPDGASKQVPVADAGTYRVTSDANQPSGDIKASATDKARNKSPEATQVYTD---QTAPAVP------  716
Bpa2   TGQAEPGAAVRVVFPNGEAQTVTAGSDGAYSVTSAADMVAGEITVVAADASGNQSAPARTVFADAVDRTAPAAP------  903
Bav1   --------------------------------------------------------------------------------  347
Yin1   --------------------------------------------------------------------------------  269
Yfr4   --------------------------------------------------------------------------------  269
Yin2   --------------------------------------------------------------------------------  270
Ymo2   --------------------------------------------------------------------------------  289
Eco10  --------------------------------------------------------------------------------  291
Eal1   --------------------------------------------------------------------------------  290
Eco16  --------------------------------------------------------------------------------  295
Sty4   --------------------------------------------------------------------------------  290
Yfr2   --------------------------------------------------------------------------------  443
Ype5   --------------------------------------------------------------------------------  410
Eco6   KDGSEADGAMANMLRLKITDAFGNTLAGQTVSVLAGNGATTAPTVTTQPDGTVEISVTSQTAGVSAVTATINSSTQSQNV 1526
Bbr1   --------------------------------------------------------------------------------  348
Yps2   --------------------------------------------------------------------------------  364
Eco14  --------------------------------------------------------------------------------  380
Sen3   ------SATLTAVVQDATGHPIAGAVVNWSSDNTTGNFSETTSTTNSEGKAIVTFSGTHAQLTTITASSVNNSQKTVQVT  632
Pal3   --------------------------------------------------------------------------------   86
Ybe1   --------------------------------------------------------------------------------  350
Pal2   TIEAGDTTELTLRLKDLVDNGVTNIQNRDIHLTQKNAKIDKKWLSAMDGIYTTQVQIKQVGVYPLRATVNQQNSRIETIE 1500
Eco1   --------------------------------------------------------------------------------  420
Yps7   ------------VYTATLTAGTVAGVASLSVSVGGSALGVAPATVTLNGDSGNLSTTHSTLVAAP---------------  453
Yfr3   --------------------------------------------------------------------------------  425
Eco20  ------------DCSYVATLTTGGKTGELRVMPLFNGQPAATEAAQLTVIAGEMSSANSTLVADN---------------  477
Efe5   --------------------------------------------------------------------------------  406
Csu1   --------------------------------------------------------------------------------  426
Ahy1   --------------------------------------------------------------------------------  403
Plu1   ----TDSYTFTATVVDAHGKPVADKPIDIDWQTNPKADGLTLTKQSNPVSNAQGQVTAILTSTAAVKDVQVSAKAAAN--  676
Cla1   --------------------------------------------------------------------------------  387
Pas1   --------------------------------------------------------------------------------  363
ruler  ......2410......2420......2430......2440......2450......2460......2470......2480
```

```
Eca1   --------------------------------------------------------------------------------   53
Esp2   --------------------------------------------------------------------------------   53
Cko1   --------------------------------------------------------------------------------   53
Sen1   --------------------------------------------------------------------------------   53
Eco3   --------------------------------------------------------------------------------   49
Esa3   --------------------------------------------------------------------------------   54
Kpn1   --------------------------------------------------------------------------------   51
Pan1   --------------------------------------------------------------------------------   61
Eta2   --------------------------------------------------------------------------------   52
Spr1   --------------------------------------------------------------------------------   69
Eco25  VTYVPNVANAEISLAASKDPVIANNNDLTTLTATVADTEGNAIANSEVTFTLPEDVRANFTLGDGGKVVTDTEGKAKVTL 1339
Eco15  --------------------------------------------------------------------------------  734
Yfr1   --------------------------------------------------------------------------------  296
Yfr5   --------------------------------------------------------------------------------  345
Yps4   NVVATVDTVNANIDTTFVAGAVATITLTTPVNGAVANGADSNSVQAVVSDSEGNAVAGAAVVFSSANATAQITTVIGTTG 1747
Efe2   --------------------------------------------------------------------------------  604
Efe3   RQSDPY-------------------------------------------------------------------------- 1077
Eta1   -ITGGTT-------------------------------------------------------------------------  342
Eco26  VLTVGTI-------------------------------------------------------------------------  640
Pru1   NIKVDAI------------------------------------------------------------------------- 1359
Sgl1   --------------------------------------------------------------------------------  447
Pmi1   TIKVAIP------------------------------------------------------------------------- 1338
Ymo1   QMLVGNI-------------------------------------------------------------------------  452
Yen2   QIVIGNI------------------------------------------------------------------------- 1479
Sen2   --------------------------------------------------------------------------------  269
Efe4   --------------------------------------------------------------------------------  263
Bpe1   --------------------------------------------------------------------------------  547
Bav2   --------------------------------------------------------------------------------  716
Bpa2   --------------------------------------------------------------------------------  903
Bav1   --------------------------------------------------------------------------------  347
Yin1   --------------------------------------------------------------------------------  269
Yfr4   --------------------------------------------------------------------------------  269
Yin2   --------------------------------------------------------------------------------  270
Ymo2   --------------------------------------------------------------------------------  289
Eco10  --------------------------------------------------------------------------------  291
Eal1   --------------------------------------------------------------------------------  290
Eco16  --------------------------------------------------------------------------------  295
Sty4   --------------------------------------------------------------------------------  290
Yfr2   --------------------------------------------------------------------------------  443
Ype5   --------------------------------------------------------------------------------  410
Eco6   TFIADVRTAQIAELVVIKDGAVADGAMANMLQVKVTDAFGNALAGQTVSVTAGNSATVASTVTTKPDGTVEISVTSQTAG 1606
Bbr1   --------------------------------------------------------------------------------  348
Yps2   --------------------------------------------------------------------------------  364
Eco14  --------------------------------------------------------------------------------  380
Sen3   IAPDTQS-------------------------------------------------------------------------  639
Pal3   --------------------------------------------------------------------------------   86
Ybe1   --------------------------------------------------------------------------------  350
Pal2   VTAPVGS------------------------------------------------------------------------- 1507
Eco1   --------------------------------------------------------------------------------  420
Yps7   --------------------------------------------------------------------------------  453
Yfr3   --------------------------------------------------------------------------------  425
Eco20  --------------------------------------------------------------------------------  477
Efe5   --------------------------------------------------------------------------------  406
Csu1   --------------------------------------------------------------------------------  426
Ahy1   --------------------------------------------------------------------------------  403
Plu1   --------------------------------------------------------------------------------  676
Cla1   --------------------------------------------------------------------------------  387
Pas1   --------------------------------------------------------------------------------  363
ruler  ......2490......2500......2510......2520......2530......2540......2550......2560
```

```
Eca1   --------------------------------------------------------------------------------   53
Esp2   --------------------------------------------------------------------------------   53
Cko1   --------------------------------------------------------------------------------   53
Sen1   --------------------------------------------------------------------------------   53
Eco3   --------------------------------------------------------------------------------   49
Esa3   --------------------------------------------------------------------------------   54
Kpn1   --------------------------------------------------------------------------------   51
Pan1   --------------------------------------------------------------------------------   61
Eta2   --------------------------------------------------------------------------------   52
Spr1   --------------------------------------------------------------------------------   69
Eco25  KGTKAGAHTVTASMAGGKSEQLVVNFIADTLTAQVNLNVTEDNFIANNVGMTRLQATVTDGNGNPLANEAVTFTLPADVS 1419
Eco15  --------------------------------------------------------------------------------  734
Yfr1   --------------------------------------------------------------------------------  296
Yfr5   --------------------------------------------------------------------------------  345
Yps4   ADGIATATLINTVAGTSNVVATIDTVNANIDTTFVAGAVATITLTTPVDGAVANGADSNSVQAVVSDSEGNAVAGAAVVF 1827
Efe2   --------------------------------------------------------------------------------  604
Efe3   -------------------------------------------------------------------------------- 1077
Eta1   --------------------------------------------------------------------------------  342
Eco26  --------------------------------------------------------------------------------  640
Pru1   -------------------------------------------------------------------------------- 1359
Sgl1   --------------------------------------------------------------------------------  447
Pmi1   -------------------------------------------------------------------------------- 1338
Ymo1   --------------------------------------------------------------------------------  452
Yen2   -------------------------------------------------------------------------------- 1479
Sen2   --------------------------------------------------------------------------------  269
Efe4   --------------------------------------------------------------------------------  263
Bpe1   --------------------------------------------------------------------------------  547
Bav2   --------------------------------------------------------------------------------  716
Bpa2   --------------------------------------------------------------------------------  903
Bav1   --------------------------------------------------------------------------------  347
Yin1   --------------------------------------------------------------------------------  269
Yfr4   --------------------------------------------------------------------------------  269
Yin2   --------------------------------------------------------------------------------  270
Ymo2   --------------------------------------------------------------------------------  289
Eco10  --------------------------------------------------------------------------------  291
Eal1   --------------------------------------------------------------------------------  290
Eco16  --------------------------------------------------------------------------------  295
Sty4   --------------------------------------------------------------------------------  290
Yfr2   --------------------------------------------------------------------------------  443
Ype5   --------------------------------------------------------------------------------  410
Eco6   -------------------------------------------------------------------------------- 1606
Bbr1   --------------------------------------------------------------------------------  348
Yps2   --------------------------------------------------------------------------------  364
Eco14  --------------------------------------------------------------------------------  380
Sen3   --------------------------------------------------------------------------------  639
Pal3   --------------------------------------------------------------------------------   86
Ybe1   --------------------------------------------------------------------------------  350
Pal2   -------------------------------------------------------------------------------- 1507
Eco1   --------------------------------------------------------------------------------  420
Yps7   --------------------------------------------------------------------------------  453
Yfr3   --------------------------------------------------------------------------------  425
Eco20  --------------------------------------------------------------------------------  477
Efe5   --------------------------------------------------------------------------------  406
Csu1   --------------------------------------------------------------------------------  426
Ahy1   --------------------------------------------------------------------------------  403
Plu1   --------------------------------------------------------------------------------  676
Cla1   --------------------------------------------------------------------------------  387
Pas1   --------------------------------------------------------------------------------  363
ruler  ......2570......2580......2590......2600......2610......2620......2630......2640
```

```
Eca1   --------------------------------------------------------------------------------   53
Esp2   --------------------------------------------------------------------------------   53
Cko1   --------------------------------------------------------------------------------   53
Sen1   --------------------------------------------------------------------------------   53
Eco3   --------------------------------------------------------------------------------   49
Esa3   --------------------------------------------------------------------------------   54
Kpn1   --------------------------------------------------------------------------------   51
Pan1   --------------------------------------------------------------------------------   61
Eta2   --------------------------------------------------------------------------------   52
Spr1   --------------------------------------------------------------------------------   69
Eco25  ASFTLGQGGSAITDINGKAEVTLSGTKSGTYPVTVSVNNYGVSDTKQVTLIADAGTAKLASLTSVYSFVVSTTEGATMTA 1499
Eco15  --------------------------------------------------------------------------------  734
Yfr1   --------------------------------------------------------------------------------  296
Yfr5   --------------------------------------------------------------------------------  345
Yps4   SSANATAQITTVIGTTGADGIATATLTNTVAGTSNVVATIGSITNNIDTAFVAGAVATITLTTPVNGAVADGANSNSVQA 1907
Efe2   --------------------------------------------------------------------------------  604
Efe3   -------------------------------------------------------------------------------- 1077
Eta1   --------------------------------------------------------------------------------  342
Eco26  --------------------------------------------------------------------------------  640
Pru1   -------------------------------------------------------------------------------- 1359
Sgl1   --------------------------------------------------------------------------------  447
Pmi1   -------------------------------------------------------------------------------- 1338
Ymo1   --------------------------------------------------------------------------------  452
Yen2   -------------------------------------------------------------------------------- 1479
Sen2   --------------------------------------------------------------------------------  269
Efe4   --------------------------------------------------------------------------------  263
Bpe1   --------------------------------------------------------------------------------  547
Bav2   --------------------------------------------------------------------------------  716
Bpa2   --------------------------------------------------------------------------------  903
Bav1   --------------------------------------------------------------------------------  347
Yin1   --------------------------------------------------------------------------------  269
Yfr4   --------------------------------------------------------------------------------  269
Yin2   --------------------------------------------------------------------------------  270
Ymo2   --------------------------------------------------------------------------------  289
Eco10  --------------------------------------------------------------------------------  291
Eal1   --------------------------------------------------------------------------------  290
Eco16  --------------------------------------------------------------------------------  295
Sty4   --------------------------------------------------------------------------------  290
Yfr2   --------------------------------------------------------------------------------  443
Ype5   --------------------------------------------------------------------------------  410
Eco6   -------------------------------------------------------------------------------- 1606
Bbr1   --------------------------------------------------------------------------------  348
Yps2   --------------------------------------------------------------------------------  364
Eco14  --------------------------------------------------------------------------------  380
Sen3   --------------------------------------------------------------------------------  639
Pal3   --------------------------------------------------------------------------------   86
Ybe1   --------------------------------------------------------------------------------  350
Pal2   -------------------------------------------------------------------------------- 1507
Eco1   --------------------------------------------------------------------------------  420
Yps7   --------------------------------------------------------------------------------  453
Yfr3   --------------------------------------------------------------------------------  425
Eco20  --------------------------------------------------------------------------------  477
Efe5   --------------------------------------------------------------------------------  406
Csu1   --------------------------------------------------------------------------------  426
Ahy1   --------------------------------------------------------------------------------  403
Plu1   --------------------------------------------------------------------------------  676
Cla1   --------------------------------------------------------------------------------  387
Pas1   --------------------------------------------------------------------------------  363
ruler  ......2650......2660......2670......2680......2690......2700......2710......2720
```

CLUSTAL X (1.81.1-alpha) MULTIPLE SEQUENCE ALIGNMENT

File: /Users/saierlab/Desktop/58cterm.ps   Date: Tue May 12 18:49:09 2009


```
  Eca1 --------------------------------------------------------------------------------   53
  Esp2 --------------------------------------------------------------------------------   53
  Cko1 --------------------------------------------------------------------------------   53
  Sen1 --------------------------------------------------------------------------------   53
  Eco3 --------------------------------------------------------------------------------   49
  Esa3 --------------------------------------------------------------------------------   54
  Kpn1 --------------------------------------------------------------------------------   51
  Pan1 --------------------------------------------------------------------------------   61
  Eta2 --------------------------------------------------------------------------------   52
  Spr1 --------------------------------------------------------------------------------   69
 Eco25 SVTDANGNPVEGIKVNFRGTSVTLSSTSVETDDRGFAEILVTSTEVGLKTVSASLADKPTEVISRLLNAKADINSATITS 1579
 Eco15 --------------------------------------------------------------------------------  734
  Yfr1 --------------------------------------------------------------------------------  296
  Yfr5 --------------------------------------------------------------------------------  345
  Yps4 VVTDSGGNPVNGAAVVFSSANATAQITTVIGTTGADGIATATLTNTVAGTSNVVATVDTVNANIDTTFVAGAVATITLTT 1987
  Efe2 ----------------------------------------------------------------------EAN-------  607
  Efe3 --------------------------------------------------------------NIQPGEPAEAQSGISTDG 1095
  Eta1 --------------------------------------------------------------------------------  342
 Eco26 --------------------------------------------------------------------------------  640
  Pru1 -------------------------------------------------------------------------------- 1359
  Sgl1 --------------------------------------------------------------------------------  447
  Pmi1 -------------------------------------------------------------------------------- 1338
  Ymo1 --------------------------------------------------------------------------------  452
  Yen2 -------------------------------------------------------------------------------- 1479
  Sen2 --------------------------------------------------------------------------------  269
  Efe4 --------------------------------------------------------------------------------  263
  Bpe1 --------------------------------------------------------------------------------  547
  Bav2 --------------------------------------------------------------------------------  716
  Bpa2 --------------------------------------------------------------------------------  903
  Bav1 --------------------------------------------------------------------------------  347
  Yin1 --------------------------------------------------------------------------------  269
  Yfr4 --------------------------------------------------------------------------------  269
  Yin2 --------------------------------------------------------------------------------  270
  Ymo2 --------------------------------------------------------------------------------  289
 Eco10 --------------------------------------------------------------------------------  291
  Eal1 --------------------------------------------------------------------------------  290
 Eco16 --------------------------------------------------------------------------------  295
  Sty4 --------------------------------------------------------------------------------  290
  Yfr2 --------------------------------------------------------------------------------  443
  Ype5 --------------------------------------------------------------------------------  410
  Eco6 ---------------TSTVTASINNSSQSQNVTFVPCDASRLTSTVETNKSNYTVGETITITVTLRDAFDNLVTGAASQL 1671
  Bbr1 --------------------------------------------------------------------------------  348
  Yps2 --------------------------------------------------------------------------------  364
 Eco14 --------------------------------------------------------------------------------  380
  Sen3 --------------------------------------------------------------------------------  639
  Pal3 --------------------------------------------------------------------------------   86
  Ybe1 --------------------------------------------------------------------------------  350
  Pal2 -------------------------------------------------------------------------------- 1507
  Eco1 --------------------------------------------------------------------------------  420
  Yps7 --------------------------------------------------------------------------------  453
  Yfr3 --------------------------------------------------------------------------------  425
 Eco20 --------------------------------------------------------------------------------  477
  Efe5 --------------------------------------------------------------------------------  406
  Csu1 --------------------------------------------------------------------------------  426
  Ahy1 --------------------------------------------------------------------------------  403
  Plu1 --------------------------------------------------------------------------------  676
  Cla1 --------------------------------------------------------------------------------  387
  Pas1 --------------------------------------------------------------------------------  363
 ruler ......2730......2740......2750......2760......2770......2780......2790......2800
```

```
Eca1   --------------------------------------------------------------------------------   53
Esp2   --------------------------------------------------------------------------------   53
Cko1   --------------------------------------------------------------------------------   53
Sen1   --------------------------------------------------------------------------------   53
Eco3   --------------------------------------------------------------------------------   49
Esa3   --------------------------------------------------------------------------------   54
Kpn1   --------------------------------------------------------------------------------   51
Pan1   --------------------------------------------------------------------------------   61
Eta2   --------------------------------------------------------------------------------   52
Spr1   --------------------------------------------------------------------------------   69
Eco25  LEIPEGQVMVAQDVAVKAHVNDQFGNPILNESVTFSAEPPEHMTISQNIVSTDTHGIAEVTMTPERNGSYMVKASLANGS 1659
Eco15  ------------------------------------------------------------------NSSSKVDVNVANG-  747
Yfr1   --------------------------------------------------------------------------------  296
Yfr5   --------------------------------------------------------------------------------  345
Yps4   PVNGAVADGADSNSVQAVVSDSGGNPVAGAAVVFSSANATAQVTTVIGTTGADGIATATLTNTVAGTSNVVATIGSITNN 2067
Efe2   --------IPKVGTLWAEWG-----------KTLVSEGLVSHPTIWTP---------------------SAQAASGWALL  647
Efe3   DTFVAGQFIPVTVTLKDAWGNHVPAQESYLATHVVAAHTVPQPTQWQENSDGTYSAQFIAGEPGADLQVTLNQDSGWKVA 1175
Eta1   ------------------------------TPELGFATAQHNVTWTKNFSDSQAVSGVPEG--VEQQWSSSDNSVATVN-  389
Eco26  ------------------------------NTNLSFDNKTINETYKGRAVLIQQVIGLPESNHPPVTWATSDPLVAEIDS  690
Pru1   ------------------------------NPTLRFDNIQKRLTYSSITDNGQVIKGLPAG--AAPVWSSDNTSIATVD- 1406
Sgl1   --------------------------------------------------------------------------------  447
Pmi1   ------------------------------KSEITFEKPIQQEIYKSTVIDALSYKGVPQN--MQVIWSSSDPTVASIDT 1386
Ymo1   ------------------------------IPQLSFDNKNEKQTYRKTPFAKQALKGLP--QSVIAHWNSDNSDVAKIDP  500
Yen2   ------------------------------IPQLSFAKSKEATTYSRKVHKPLSLTGLPSSATLTAHWSSDNSDVATVNP 1529
Sen2   --------------------------------------------------------------------------------  269
Efe4   --------------------------------------------------------------------------------  263
Bpe1   ---------------------------------TIVRVTTDRSSGVVTVAGTADPDNDVTVQFPDGGRKTVKAGKDGSYS  594
Bav2   ---------------------------------LISTVNTSPQTGVVTVEGTAEADSQVKVSFPDGTSKTVSADGSGHYT  763
Bpa2   ---------------------------------TLA-LSEAADSGRLTVSGRTEPGASVRVTFPDGETVTVTAKADGTYT  949
Bav1   --------------------------------------------------------------------------------  347
Yin1   --------------------------------------------------------------------------------  269
Yfr4   --------------------------------------------------------------------------------  269
Yin2   --------------------------------------------------------------------------------  270
Ymo2   --------------------------------------------------------------------------------  289
Eco10  --------------------------------------------------------------------------------  291
Eal1   --------------------------------------------------------------------------------  290
Eco16  --------------------------------------------------------------------------------  295
Sty4   --------------------------------------------------------------------------------  290
Yfr2   --------------------------------------------------------------------------------  443
Ype5   --------------------------------------------------------------------------------  410
Eco6   AADGVLTVAGTDPSETGSWVESGGVYTTTRMATIASTNQHANLQLQTWSDGVTSDRYDIQSGSPAQATSTIATDKNAYTA 1751
Bbr1   --------------------------------------------------------------------------------  348
Yps2   --------------------------------------------------------------------------------  364
Eco14  --------------------------------------------------------------------------------  380
Sen3   ----------------------------AQPVTVVADKHGAIANGADTVTLTATIQDQYGNFINQGDVAWTISPEASYHL  691
Pal3   --------------------------------------------------------------------------------   86
Ybe1   --------------------------------------------------------------------------------  350
Pal2   ------------------------TKVAKAKLASSIVNLDAGNNVELTLELKDQYDNLIIGVNGSDIMLENSYTAETIDN 1563
Eco1   --------------------------------------------------------------------------------  420
Yps7   ---------------------------------VSIEANGSDTSLVTLTLRDSNNNPVTGQTVALVSTLGTLGAVTEQAS  500
Yfr3   --------------------------------------------------------------------------------  425
Eco20  ------------------------------------KAPTVKMTTELTFTVKDAYGNPVTGLKPDAPVFSGAASTGSERP  521
Efe5   --------------------------------------------------------------------------------  406
Csu1   -------------------------------AKVSVVVPKKMLTKDTSITQGNSSYKPITATPSATDGTVSFTRITPTLP  475
Ahy1   --------------------------------------------------------------------------------  403
Plu1   --------------------------PSWVDADRKVSFEELSLSYQVTGVTVEVDKDKAHYNNGTDSYTFTATVVDGHGK  730
Cla1   --------------------------------------------------------------------------------  387
Pas1   --------------------------------------------------------------------------------  363
ruler  ......2810......2820......2830......2840......2850......2860......2870......2880
```

CLUSTAL X (1.81.1-alpha) MULTIPLE SEQUENCE ALIGNMENT

File: /Users/saierlab/Desktop/58cterm.ps   Date: Tue May 12 18:49:09 2009


```
Eca1   --------------------------------------------------------------------------------   53
Esp2   --------------------------------------------------------------------------------   53
Cko1   --------------------------------------------------------------------------------   53
Sen1   --------------------------------------------------------------------------------   53
Eco3   --------------------------------------------------------------------------------   49
Esa3   --------------------------------------------------------------------------------   54
Kpn1   --------------------------------------------------------------------------------   51
Pan1   --------------------------------------------------------------------------------   61
Eta2   --------------------------------------------------------------------------------   52
Spr1   --------------------------------------------------------------------------------   69
Eco25  SYEKDLVVIDQKLTLSASSPLIGVNSPTGATLTATLTSANGTPVEGQVINFSVTPEGATLS-------------------   1720
Eco15  -----------KLELTSSKPETTVHNSEGITLTATARNARGELMPGQIITFSVTPEGATLSN------------------   798
Yfr1   --------------------------------------------------------------------------------   296
Yfr5   --------------------------------------------------------------------------------   345
Yps4   IDTAFVAGAVATITLTTPVNGAVADGADSNSVQAVVSDSEGNAVTGAAVVFSSANATAQITTVIGTTGADGIATATLTNT   2147
Efe2   S----------IEN---------------------------NVFPG--------IQEGKFN-------------------   663
Efe3   ATYSIIDAQPVIDNSFVDTDKDSYQAGDDMLITVKLADSRGNALSGREAILNDAVEVGSATR------------------   1237
Eta1   EVGKVTLLKSGQTTITVKTSGNDQYDPAEASYQLKIDKADPQLQAGDGEPITAEWADGKVWS------------------   451
Eco26  RTGYITMKKAGMVTITASMPGNDKYSPGIASYNLIISKANPQISSDG--IINAVWGDKSEKI------------------   750
Pru1   NQGKVKLKKAGRTKIWARIDGNGVYKSAAANYELEVEKARPNLTLTSNSTISATWADNNNHP------------------   1468
Sgl1   --------------------------------------------------------------------------------   447
Pmi1   TSGQISMKKAGTTIITLQTLGNEQYPSAKNSYPLVIEKAPPKLKVTSPHTIQSIWNDGITHQ------------------   1448
Ymo1   ATGEIKLLKAGVVNISAVTLADNTYAMGTASYQLEVERADPKLNFTKRFSNVTWGQVAAKEQ------------------   562
Yen2   LNGELTLLKAGVVNISVLTLPTDTYTSGTANYQLTVEKADPGINFAVAKRDVKWMDSMSPQN------------------   1591
Sen2   --------------------------------------------------------------------------------   269
Efe4   --------------------------------------------------------------------------------   263
Bpe1   VTSDNDIFSGIIQVSARNPAGNASPEVRQDYRD---EFVPANAITNIETNS-----------------------------   642
Bav2   ATSDHDQPSGEIKVQATDAANNKSPEATKAYADGVDKTPPVVSISNVQAAD-----------------------------   814
Bpa2   ATSRADMIGGNVTVVATDAAGNAAAPVRAAYADTVDRTPPVLDTPVLSVASDSGRVTVTGVTEPGARVQVAIPGESLQTV   1029
Bav1   --------------------------------------------------------------------------------   347
Yin1   --------------------------------------------------------------------------------   269
Yfr4   --------------------------------------------------------------------------------   269
Yin2   --------------------------------------------------------------------------------   270
Ymo2   --------------------------------------------------------------------------------   289
Eco10  --------------------------------------------------------------------------------   291
Eal1   --------------------------------------------------------------------------------   290
Eco16  --------------------------------------------------------------------------------   295
Sty4   --------------------------------------------------------------------------------   290
Yfr2   --------------------------------------------------------------------------------   443
Ype5   --------------------------------------------------------------------------------   410
Eco6   GDTITVAVTLKDAHGNLVEGGESLLSGDNVTVEGAVRSGGRSETAGVYTATWSAQMAGDSHHATLKLSEWGSSKQSESYS   1831
Bbr1   --------------------------------------------------------------------------------   348
Yps2   --------------------------------------------------------------------------------   364
Eco14  --------------------------------------------------------------------------------   380
Sen3   SANNQPTNNEGQSIVTLASDDVVSCKGTATFNGLSKSTATIRFTADTTTEKVDTLNASKTEN------------------   753
Pal3   --------------------------------------------------------------------------------   86
Ybe1   --------------------------------------------------------------------------------   350
Pal2   SRLAWRMDSAGIYKASLPLTLVGKHKLSAVINKQRTSTADITVNALKGAANVSQVIITTGKN------------------   1625
Eco1   --------------------------------------------------------------------------------   420
Yps7   GVYTATLTAGTVAGVASLSVSVGGSALGVAPATVTLNGDSGNLSTTHSTLVAAPVSIEANGS------------------   562
Yfr3   --------------------------------------------------------------------------------   425
Eco20  SAGNWTEKGNGVYVATLTLGSAAGQLSVMPRVNGQNAVAQPLVLNVAGDASKAEIRDMTVKVN-----------------   584
Efe5   --------------------------------------------------------------------------------   406
Csu1   AGINLNSATGEITGTPTEESPLQVYTMTVRDGKSGVESEVDFELGVAPSFVAQQKTYQKVIG------------------   537
Ahy1   --------------------------------------------------------------------------------   403
Plu1   PVADKPIDIDWQTEPTVEGLKLTKQNNSVSNAQGQVTATLTSTAAVENVQVSAKTASQKTAEN-----------------   793
Cla1   --------------------------------------------------------------------------------   387
Pas1   --------------------------------------------------------------------------------   363
ruler  ......2890......2900......2910......2920......2930......2940......2950......2960
```

```
Eca1  --------------------------------------------------------------------------------   53
Esp2  --------------------------------------------------------------------------------   53
Cko1  --------------------------------------------------------------------------------   53
Sen1  --------------------------------------------------------------------------------   53
Eco3  --------------------------------------------------------------------------------   49
Esa3  --------------------------------------------------------------------------------   54
Kpn1  --------------------------------------------------------------------------------   51
Pan1  --------------------------------------------------------------------------------   61
Eta2  --------------------------------------------------------------------------------   52
Spr1  --------------------------------------------------------------------------------   69
Eco25 ---------------------------GGKVRTNSSGQAPVVLTSNKVGTYTVTASFHNGVTIQTQTIVKVTGNSSTAHV 1773
Eco15 ---------------------------TGEVLTDQSGQAKVTLTSDKVNVYTVTAIMGKDVPVQSQVTVAVKADAKTAHV  851
Yfr1  --------------------------------------------------------------------------------  296
Yfr5  --------------------------------------------------------------------------------  345
Yps4  VAGTSNVVATIGGITNNIDTAFVAGAVATITLTTPVNGAVADGTDSNSVQAVVSDSEGNAVAGAAVVFSSANATAQITTV 2227
Efe2  ----------------------------------------------------------EFDGELTSANIYGRVCVFEL--  683
Efe3  --------------------------KEGSIWTEDTQHKGTYTAYYMAQIAGQDSVKLALDDGVKSSDTYTIVAAAPVVK 1291
Eta1  -------------------------------IASKFGNIDAEN--SLTATYTSENTGIVTVAGN-GELQAVKPGSTRLTV  497
Eco26 -------------------------------ISVSFNNEDVASNIKPPVSFTSKDPSVVSIDNT-GKIKMVKPGSTLIEV  798
Pru1  -------------------------------IQSAFNHTDAAS---IPVEYISQDASIAQIDSASGAITQVKPGVTKLTI 1514
Sgl1  --------------------------------------------------------------------------------  447
Pmi1  -------------------------------ITAEFDNPEVKN---TPIHFSSSDTLVATIDSK-GNLTAIKPGKTKIVI 1493
Ymo1  -------------------------------PNVGNSDWANSQ---PQLKWSSSEPNVAKVDAN-GNITLLKAGDTSIKA  607
Yen2  -------------------------------FVLSNSDANQSD---IKTIWQTDSGKIATVDKG-GLVTLVKPGTTNVTV 1636
Sen2  --------------------------------------------------------------------------------  269
Efe4  --------------------------------------------------------------------------------  263
Bpe1  -------------------------------------------------------------RNGIVTVSGKTSPDATVVV  661
Bav2  -------------------------------------------------------------ATGIVTVTGHTEAGSTVQV  833
Bpa2  TADSAGRYRAVSPGDVLRSGTVRVIATDAHGNASAPRDVPYQDAVDRTAPPLTIEAAVEQARTGVVNVSGKTEPNLMVSA 1109
Bav1  --------------------------------------------------------------------------------  347
Yin1  --------------------------------------------------------------------------------  269
Yfr4  --------------------------------------------------------------------------------  269
Yin2  --------------------------------------------------------------------------------  270
Ymo2  --------------------------------------------------------------------------------  289
Eco10 --------------------------------------------------------------------------------  291
Eal1  --------------------------------------------------------------------------------  290
Eco16 --------------------------------------------------------------------------------  295
Sty4  --------------------------------------------------------------------------------  290
Yfr2  --------------------------------------------------------------------------------  443
Ype5  ------------------------------------------------VVVETTSAKTDENGKVTTNLSMTNVGGTISTV  442
Eco6  IHSGAPVQANSAIRTDKSAYIAGEPLTVTITLRDEFGNPALGLTSEVIESYIDSFAVGGATPDSMRWVEQNNGEYTIVWT 1911
Bbr1  -----------------------------------------------------------ATDHYLDAWTKQTLLGGKIRL  369
Yps2  ---------------------------------------------------ATFQLQMDNDVANNTQYEWSSSFTPNVSV  393
Eco14 -----------------------------------------------VTLTSTQAGQAVVNATVDGKQISAQSVTFTRTV  413
Sen3  --------------------------------------------------VVAGKDTITLEATVTDENGHPVADTTVHWG  783
Pal3  --------------------------------------------------------------------------------   86
Ybe1  ---------------------------------------------------------------LKTTLTSSTNGSSGTPV  367
Pal2  -------------------------TISVGEKTELALKVQDRFGNEVDDVLASDIDLTNTDSQIKTSVKWVKSPLTLGTY 1680
Eco1  --------------------------------------------------------------------------------  420
Yps7  -----------------------------------------------DTSLVTLTLRDSNNNPVTGQTVALVSTLGTLGA  595
Yfr3  --------------------------------------------------------------------------------  425
Eco20 -------------------------------------------------NQLANGQSANQITLTVVDSYGNPLQGQEVTL  615
Efe5  ---------------------------------------------------LSVPANIITDAAGRVSVPLTTVKAGEYTV  435
Csu1  -------------------------------------------------------VNSPVSFPICILSGGVPPYNVTITP  562
Ahy1  --------------------------------------------------------------------------------  403
Plu1  ---------------------------------------ANGKVSFTEFSASYYVASVTVEVDKDEKHYNNGTDSYIFTA  834
Cla1  ------------------------------------------------AKALLTSKTPFKTPIKIVTSGVGKNSNKVITY  419
Pas1  -----------------------------------------------------------------ERLDQERVEQEARDA  378
ruler ......2970......2980......2990......3000......3010......3020......3030......3040
```

```
 Eca1 --------------------------------------------------------------------------------   53
 Esp2 --------------------------------------------------------------------------------   53
 Cko1 --------------------------------------------------------------------------------   53
 Sen1 --------------------------------------------------------------------------------   53
 Eco3 --------------------------------------------------------------------------------   49
 Esa3 --------------------------------------------------------------------------------   54
 Kpn1 --------------------------------------------------------------------------------   51
 Pan1 --------------------------------------------------------------------------------   61
 Eta2 --------------------------------------------------------------------------------   52
 Spr1 --------------------------------------------------------------------------------   69
Eco25 ASFIADPSTIAATNSDLSTLKATVEDGSGNLIEGLTVYFALKS-GSATLTSLTAVTDQNGIATTSVRGAITGSVTVSAVT 1852
Eco15 VSVVASPDTITADGIDSSTITSRVEDDYGFPVEGVDISHGLDTKGSPVVNIPTTRTDQSGQVTATITSTLAETLTVNVQV  931
 Yfr1 --------------------------------------------------------------------------------  296
 Yfr5 --------------------------------------------------------------------------------  345
 Yps4 IGTTGADGIATATLTNTVAGTSNVVATIGSITNNIDTAFVAGAVATITLTTLVNGAVANGADSNSVQAVVSDSGGNVVAG 2307
 Efe2 --------------------------------------------------------------------------------  683
 Efe3 NSAIQRGEAATYTAGDTLTLTVTLQDDWGNPVSGMENILRDSVTLPEAGLQERGWQQTPSGEYEANWTAQKAGTSLTASL 1371
 Eta1 STPETDQFKPASVEVAYQLDKRTVDVSFKAAVIKTTDEE----EFTLQLPENALPSDAIFTWESADKKVLDISSSGIVQG  573
Eco26 RSEETEQFLQSSAIVTYVLDKGRRNITFKNSDVKMTVDE----KFSLQTPELNNITTSSKIWRSSNPKVIEINQDGSLPS  874
 Pru1 RSKETEQFLSESQEVTYNLAKARFNIDFAQKEQEITDEKG---HFALQNTQITVPSKADIVWTSGADSVVNLEKNGTLKD 1591
 Sgl1 --------------------------------------------------------------------------------  447
 Pmi1 QSDATDKFLADSQTVDYQQEKAALSYYFKKNYIVLTTDSAADKLLMAQSPTPDVPVEANATWYSSQPNIVEITPDGIIKN 1573
 Ymo1 TTAQTDQFKAAEAEYVLNINKANLQLSFNASVKQYSVDSKP--SNVQSLILPTDLPASAVRWSSSNPSAMNIDSNG-RVS  684
 Yen2 SFVGDERFKYGEASYELNVAKYKPTVSFANSLLTNKVSEKI--YVQKPDEKLSTYAHLETKWSSSDNAIVEVANDASYMS 1714
 Sen2 --------------------------------------------------------------------------------  269
 Efe4 --------------------------------------------------------------------------------  263
 Bpe1 SFPGGDEARTRAKGDGTFSVSSPMDIPRG--VVSLSTEVNGVQTVVATRTYEDKFTKGGLDFTAFLPYTPKFNGVSTSDT  739
 Bav2 TFPDGQAVNATVDQDGSYTARSSKDVTQSGDITATATDAAGNPSAPATQAYDDTVDRTAPPVPTISSVTGNAAGQVTVVG  913
 Bpa2 TFPDGQTAQARADGQGRYQLASPADVARSGRIAVSASDAAGN-RAAASAEFTDQVDKTAPAVTIRAVTEQAGTGVATVSG 1188
 Bav1 --------------------------------------------------------------------------------  347
 Yin1 --------------------------------------------------------------------------------  269
 Yfr4 --------------------------------------------------------------------------------  269
 Yin2 --------------------------------------------------------------------------------  270
 Ymo2 --------------------------------------------------------------------------------  289
Eco10 --------------------------------------------------------------------------------  291
 Eal1 --------------------------------------------------------------------------------  290
Eco16 --------------------------------------------------------------------------------  295
 Sty4 --------------------------------------------------------------------------------  290
 Yfr2 --------------------------------------------------------------------------------  443
 Ype5 TATMINSANVTSTQDKPVIFYPDFTKATLNTPANTYSGFNINSGFPTTGFKNTHFQLSPHGITGANSDYDWVSSHPNVSV  522
 Eco6 AWVAEENLVASLKLKTWATEIKSSLYGIQPGAAAKNQSTIVADKTIYIAGDSITVTVVLKDAQGNFITDGVVQLNEENVQ 1991
 Bbr1 LRPVARLLLSPGSMTYTEIAKSFDGSSLDGIVARFEPANGAPPQTAALLAAIKLHDPNYRLESNKMFIYLDTMNSDPYNR  449
 Yps2 NDQGQVTITYQTYSEVAVTAKSKKFPSYSVSYRFYPNRWIYDGGRSLVSSLEASRQCQGSDMSAVLESSRATNGTRAPDG  473
Eco14 RGVITVEKERVYPGTRQTVTLTLTDAAGNPVSGERVDWHVDSGNLWQTQGTTDAQGRTTTTWESTTPGTATITADAYGQQ  493
 Sen3 TDNSSGTFQPGDSSVTDSNGVATVTYSATKAVPTLIGAGINQSEKTITVNYIGNADTAKLSNIKPDKTKAVADNTELVTW  863
 Pal3 --------------------------------------------------------------------------------   86
 Ybe1 SKNNDVIFTVFTSPDSPVADYWGHMDETIIVDGVIIKRPKLAAEVAGSQQIDMNNEEWAYANFTNASAHCTLPTKEDLVA  447
 Pal2 ITDVQFDKVKSHTLIASVNGQTKMLQIHVQPLKGYSNVAAIALQTPAKIEVAEKTKLTLTLMDKFNNGVVGVEAQHIELL 1760
 Eco1 --------------------------------------------------------------------------------  420
 Yps7 VTEQASGVYTATLTAGTVAGVASLSVSVGGSALGVAPATVTLNGDSGNLSTTHSTLVAAPVNIEANGSDTSLVTLTLRDS  675
 Yfr3 --------------------------------------------------------------------------------  425
Eco20 TLPQGVTSKTGNTVTTNAAGKVDIELMSTVAGEHSITASVNNAQKTVTVKFKADFSTGQATLEVDGSTPKVANDNDAFTL  695
 Efe5 TARVTDGSNSVESGSVKLTFVPDAASAELTIGASKQQIVADGHENATVDIQMLDANNNAFAGDVNLTITPSTGASLTSSK  515
 Csu1 NLPNGLQLDATTGEISGSAKATSNQTTYTVNVTDANLSPRSPMTLALTVANAPTVTEVESNKVAVIGSYVNYTPLKATSA  642
 Ahy1 --------------------------------------------------------------------------------  403
 Plu1 TVKDGHGNLVVGQPVDIDWQTDPKADGLKLTKQNNSVSNAQGQVTATLTSTVVAIDVQVSAKTATQQTPVNVDKKVSFIS  914
 Cla1 DLRTFTPVITYPVFDSTLYGKHEKTIDYDTDYSILVKGLLPDSIIEPITNGVIKPKSNSVQVNNKGEATIAYHKISDFNT  499
 Pas1 WLRQEEQERLEQERLEQERLERERLEQERLEQERLEQERRDKILERRKGTYGDSDDDSDDDTYQESPETTKEPEKKPELQ  458
ruler ......3050......3060......3070......3080......3090......3100......3110......3120
```

```
Eca1   --------------------------------------------------------------------------------   53
Esp2   --------------------------------------------------------------------------------   53
Cko1   --------------------------------------------------------------------------------   53
Sen1   --------------------------------------------------------------------------------   53
Eco3   --------------------------------------------------------------------------------   49
Esa3   --------------------------------------------------------------------------------   54
Kpn1   --------------------------------------------------------------------------------   51
Pan1   --------------------------------------------------------------------------------   61
Eta2   --------------------------------------------------------------------------------   52
Spr1   --------------------------------------------------------------------------------   69
Eco25  TAGGMQTVDITLVAGPADASQSVLKNNRSSLKGDFTDSAELHLVLHDISGNPIKVSEGLEFVQSGTNAPYVQVSAIDYSK   1932
Eco15  PGTANQSATITLVAGTADESKSILKSDVDTLKADYQQSAKLTLTLQDKYGNPIVTSDHLEFVQSGPFVNFLKLSDIDYSQ   1011
Yfr1   --------------------------------------------------------------------------------   296
Yfr5   --------------------------------------------------------------------------------   345
Yps4   ATVVFSSTNATAQVTTVIGTTGADGIATATLTNTVAGTSNVVATIDTVNANIDTTFVAGAVATITLSVLVNDATADGADT   2387
Efe2   --------------------------------------------------------------------------------   683
Efe3   ELEGWRKETAPFAIVAGTPVQSESSLQTDKESYTVEDTLELTVTLRDAWKNPVSG-KLALVNDAVLTIDAASATDVFSEG   1450
Eta1   KVSK----GKTRLTLSITANDYYSASSNYYDMWIYSKPSVSIGKVTYISKGVMADKGV------WTPVFTDDKFSVTWST   643
Eco26  NKETPNDAGYSEISLIIPSDEYYHEERSSYNLHVYGQPAINIGKISYVGNAQVQNNGR------WTPVYADDVITINWSV   948
Pru1   LAKG-----EANLIMTVKANDYFEQTSGEYRVKVYTKPAISANIITYGNNGNRQDNTSN-----WSPVYTDDNLEINWSL   1661
Sgl1   --------------------------------------------------------------------------------   447
Pmi1   LNIG-----QTTLTLEVKNNNYFQDHEQSYNVIIQASPRITINSFEFLSAGESKSNTHLDDLTWQPLYTSDDQFIVKWSS   1648
Ymo1   GVSA----GDSNIKIEIDKNDFYAENSAKYLAEVYQKPSVSISSTNTVSKGVSGSNSSE-----WKPAFKGDQMAVNWRV   755
Yen2   PKGP----GKARITLQVVGNDWYEEQSSSYEQEVYATPKVSIRETTAISNSVKKVNERV-----WSPVFTNDNFGVTVDN   1785
Sen2   --------------------------------------------------------------------------------   269
Efe4   --------------------------------------------------------------------------------   263
Bpe1   SAVLLSIPRYFYSGG---------------------APVAKIMPPEAD--GSEFAKRAAAAMR-----AQVTPGRDSSEY   791
Bav2   TAEPGSTVTINFPGGSEKQVQLNADGQYSVTSD-GHIPTGDIKASATDREGNKSAEVSKAYMS-----APPAPVINSVTT   987
Bpa2   ATEPGATVSVTFPDGQVARATADRSGAYTVHSARDVTARGNISVIATDAAGNASPSVSRQFSNTVADTVPPSLTIAMVTT   1268
Bav1   --------------------------------------------------------------------------------   347
Yin1   --------------------------------------------------------------------------------   269
Yfr4   --------------------------------------------------------------------------------   269
Yin2   --------------------------------------------------------------------------------   270
Ymo2   --------------------------------------------------------------------------------   289
Eco10  --------------------------------------------------------------------------------   291
Eal1   --------------------------------------------------------------------------------   290
Eco16  --------------------------------------------------------------------------------   295
Sty4   --------------------------------------------------------------------------------   290
Yfr2   --------------------------------------------------------------------------------   443
Ype5   SNTGAITLQDNPGGKVTITATWKHDSSKVFTYDFTLNYWVGLYSSTNLSWAQANASCINAGMRLPTNREVSAGQDVRGVG   602
Eco6   VRNADPIQGNNWVYNGNGQYQRQYMAHFAEANLNAQLKMAGWSDANYSNNYTIKPGEVSPLGSQLRIREVLVVEGADLPV   2071
Bbr1   VPNGDYPVTLVLEDKATGAREATTMVLKVTGSTYGKAPVVPGANGVLGTGPGPSLGGSLLIGGEGGLLGS----------   519
Yps2   TLWGEWGSLTAYSSDWQSGEYWVKKTSTDFETMNMDTGALQPGPAYLAFPLCALSI------------------------   529
Eco14  YTAPVITVMPALTVSSVTGIDATGADGKNFGKRVPNSTWPGAKFRIDTENAAGTVTWTASSPAVSINGNVMTVKSNPAGV   573
Sen3   SVDVKDANGNILPGISVNWSSDDPDLTLAASSSVTNEHGVATITGRTLKARDAVVKATLSAGGQMLSAAKVTFIGDAKTA   943
Pal3   --------------------------------------------------------------------------------   86
Ybe1   LYNAYPTGSITNAHGWPSESNILYWSGTTEGSTVWATDMTLGRTVLITKEKIQLVTCL----------------------   505
Pal2   IGSTLQTATWVDNQDGSYHTEFALNQAGDTPLIVTVNKFTEMNSIHVNSPSGKDKVASIQLAATVTQVLPNTSTVLTLTL   1840
Eco1   --------------------------------------------------------------------------------   420
Yps7   NNNPVTGQTVALVSTLGTLGAVTEQASGIYTATLTAGTLTGTASLSVNVDGNSLGITPATVNVIPAPVDLTVSVDNARKN   755
Yfr3   --------------------------------------------------------------------------------   425
Eco20  TATVKDQYGNLLPGAVVVFNLPRGVKPLADGNIMVNADKEGKAELKVVSVTAGTYEITASAGNDQPSNAQSVTFVADKTT   775
Efe5   LQLDARGQATTQFTASKAGKYTIQAEYMLNGKRITASQNIDAVTDVKDAVLEITSDVSSAVVSDTSNLKFTLQLKSKSGE   595
Csu1   VTGKTVTFISVAPALPSGLSWRSYDGSIYGTPQDVTKTGQETFTFTIRDGGTGAEVQHSFNLSVVPKFTFSQTTYSKTLP   722
Ahy1   --------------------------------------------------------------------------------   403
Plu1   PDELASLTVSPDHVTEGEGEGHTYTFTATVKDFSGQAKSGITVAWSAANSKGVTITDKNLVTQVVGDGKTDADGKAQYQV   994
Cla1   KKIKPQFKYIKSGDTKETFTMNDINLYQYKLSISSDKTELVGDEIFKVTVRGGKPNASVEWTLTGDGKITSKDSKFNAKG   579
Pas1   GGNGSKPDIAPNLLLDHPLLNQVGDNSTLYTNNTDPNEEGNETDWED---------------------------------   505
ruler  ......3130......3140......3150......3160......3170......3180......3190......3200
```

```
Eca1   --------------------------------------------------------------------------------   53
Esp2   --------------------------------------------------------------------------------   53
Cko1   --------------------------------------------------------------------------------   53
Sen1   --------------------------------------------------------------------------------   53
Eco3   --------------------------------------------------------------------------------   49
Esa3   --------------------------------------------------------------------------------   54
Kpn1   --------------------------------------------------------------------------------   51
Pan1   --------------------------------------------------------------------------------   61
Eta2   --------------------------------------------------------------------------------   52
Spr1   --------------------------------------------------------------------------------   69
Eco25  NFSGEYKATVTG-GGEGIATLIPVLNGVHQAG--LSTTIQFTRAEDKIMSGTVLVNG-----ANLPTTTFPSQGFTG-AY 2003
Eco15  RNYGEYTVTVTG-GKEGTATLIPMLNGVHQAN--LSISLNLIQS-IKEMSGHVTANN-----HTFSTAKFPSEGFAG-AY 1081
Yfr1   --------------------------------------------------------------------------------  296
Yfr5   --------------------------------------------------------------------------------  345
Yps4   NQVDALVQDANGNAITGAAVVFSSANGATILS--STMNTGVNGVASTLLTHTVAGTSNVVATIDTVNANIDTAFVAG-AV 2464
Efe2   --------------------------------------------------------------------------------  683
Efe3   DTAGTYIRHFTLNSWRDSTQVSIRLQTWDRDA--TSNPYSVERRRLPMTGTIVANDGNFGLDEGFPTTGFIGAWFILSSD 1528
Eta1   ESSDELSKAKFVAIYLKDSS-GKALAQKLVDS--PAGTVTTTFEPKPRFWNESLHVELVAQGFDKLTNSEKSPG-INVKN  719
Eco26  ETSNEYKKPKKASIFFKDAANGADIYKKEILH--INGINVTELHANASLLGKKIKVVLVAEGELNLKSELEDKNEINVYE 1026
Pru1   NGSSEFDTAATIKAVVTEPN--GNTFEYPVTV--QAGIQKITLPPKREYFSQSGNLKVTISATGKSSHKLKNEFVVIDTP 1737
Sgl1   --------------------------------------------------------------------------------  447
Pmi1   SDTPKAINFELFDENGTSIALEERQNYAPKHN--IHDPVKIQVPKQYLTSPKQLTLEVTTFDSNNQPYVDPKKYKINIDY 1726
Ymo1   SGSGKFAGPKKVVVQFIEDGSGSKLFEKESTSNLASGSTVIEDTNVKNLVGKRGKFVVKTEGTSGLSS-QDQTKIIDVKR  834
Yen2   SQS-KYERADSVKVILLDG--TQELASKELG---ITTSSSFEFKPKPDWVGKSLKVKVVAKNDVRQENEVTLDHEVRVGT 1859
Sen2   --------------------------------------------------------------------------------  269
Efe4   --------------------------------------------------------------------------------  263
Bpe1   DVR-----------------VSWPAGTFTPADVGQR-------------------------------------ADLAVKV  817
Bav2   DANTGRVTVSGTAKPNGKVLVTFPGGSQREVSVSAGGDYTATSSDDEESGEIKAVLLEGAERS---RETKRNYADEAVRL 1064
Bpa2   DPGNGRITVSGVTEAGARVHVVFPGGASDTVTADSKGGYRATSAGDVGSGAVTAQATDASANRSPLKTYAYTDAWQPRQL 1348
Bav1   --------------------------------------------------------------------------------  347
Yin1   --------------------------------------------------------------------------------  269
Yfr4   --------------------------------------------------------------------------------  269
Yin2   --------------------------------------------------------------------------------  270
Ymo2   --------------------------------------------------------------------------------  289
Eco10  --------------------------------------------------------------------------------  291
Eal1   --------------------------------------------------------------------------------  290
Eco16  --------------------------------------------------------------------------------  295
Sty4   --------------------------------------------------------------------------------  290
Yfr2   --------------------------------------------------------------------------------  443
Ype5   SLLSLSIILCNCHRIKGDRSGGHRTR------------------------------------------------------  628
Eco6   SVLLVDDFGNPVDNGLDLLDDTVYLQNVEKKEGEKWRYVGDGIYERTYMAYQEGENLTSFMEIKGWRIYGQPSYTILPFV 2151
Bbr1   --------------------------------------------------------------------------------  519
Yps2   --------------------------------------------------------------------------------  529
Eco14  TLTGTDTDGQTVTLNMGGNWFAQSATKYEWLNSDYDGTAIYSCRQLGAQVASSGALQGVISEWGELETYEGWTNLKNNWL  653
Sen3   MLMSLNVDKFNVLANGGDAATYTAYVEDINHNIVPDATVSWRTTMNKLSSGTSKTNSSGKATVKLSGNSVGMVTVTATIN 1023
Pal3   --------------------------------------------------------------------------------   86
Ybe1   --------------------------------------------------------------------------------  505
Pal2   KDQYGNGVNNVLSKDISLSNSYTPENLTSPNWAEDGKQSGIYTVSVNLQKVTEHTLTAKVNNLDNILKITVQPFTEIQHV 1920
Eco1   --------------------------------------------------------------------------------  420
Yps7   IGQAISLTVTAKYKSTDVVAPNVKMTFEQVAVVNRQNNTVSISGAVQIAGVNYNSFTGMTDANGQLTVSVTDPNGIGVQT  835
Yfr3   --------------------------------------------------------------------------------  425
Eco20  ATISSIEVIGNRAVADGKTKQTYKVTVTDANNNLLKDSDVTLTASSENLVLDPKGTAKTNEQGQAVFTGSTTIAATYTLT  855
Efe5   ALSGRVVKISTTGPSKNGELVVDQSTVTTDESGKATVSVHGRTAGSYKLTATLDELGATTSAEKSFSLYADENNGVLSLS  675
Csu1   ANVAADITVLSITSGSGDYELTAPANFPSGITMSLEGTGAAQIVKVTGTPTSSQSEASYVFQVKDKKSGLSTGSRTLKLT  802
Ahy1   --------------------------------------------------------------------------------  403
Plu1   YSKSGGFVAVMVTAKVNDSSVGSKNKTVEIKANEQDVTDFFIMDYDTKDGKGPGRSVPKERMNFAWPKMRFEPEYLPGEL 1074
Cla1   EAYLNGQGKSPFNNSINVEAKAFNQILKASAKISIYESRGILAHPTGNKNGGNGRTALLGNDNGEDGQAFGNYTNGYSIF  659
Pas1   --------------------------------------------------------------------------------  505
ruler  ......3210......3220......3230......3240......3250......3260......3270......3280
```

```
Eca1   --------------------------------------------------------------------------------   53
Esp2   --------------------------------------------------------------------------------   53
Cko1   --------------------------------------------------------------------------------   53
Sen1   --------------------------------------------------------------------------------   53
Eco3   --------------------------------------------------------------------------------   49
Esa3   --------------------------------------------------------------------------------   54
Kpn1   --------------------------------------------------------------------------------   51
Pan1   --------------------------------------------------------------------------------   61
Eta2   --------------------------------------------------------------------------------   52
Spr1   --------------------------------------------------------------------------------   69
Eco25  YQLNNDNFAPGKTAADYEFSSSASWVDVDATGKVTFKNVGSKWER-ITATPKTGGPSYIYEIRVKSWWVNAGDAFMIYSL 2082
Eco15  YTLNNDNFEAGKTVDDYMFSSSQGWVSVDASGKVSFANIGDQTSVTISAVPRQGGTTYQTLIKLKGWWVNNGNHTNIWLA 1161
Yfr1   --------------------------------------------------------------------------------  296
Yfr5   --------------------------------------------------------------------------------  345
Yps4   ATITLTTPVNGAVANGADSNSVQAVVSDSEGNAVAGAAVVFSSANATAQITTVIGTTGVDGIATATLTNTVAGTSNVVAT 2544
Efe2   --------------------------------------------------------------------------------  683
Efe3   MQIEYDKTTCGGNENSCDWLHVNTMSPNSHEVSFIGQPTSETREVTINFISLSDDNIIYTYTFNLTAWFSTISPVQDTRQ 1608
Eta1   LPPNKIWETFTVRSKALIRSDNGNESTCREIAGPAGKEHWIDAIVD-------GGQVNFNGKTLISPMTITGLTDGEQNG  792
Eco26  LMPHEIWQNATITTEMSGWCR----ATWIDAPTSKTMKSNFSISLA-------SGKSLLFPMSVKVKASSNGGYGGFTYG 1095
Pru1   IQVTSPKEIGQQTLGHKVKYFLEGQTSERNICAYYLTDRTIDLVIAPTMNINTNGRRLLAPIYVKHEILSSEYSGTGAAG 1817
Sgl1   --------------------------------------------------------------------------------  447
Pmi1   FPANNAYKIISLDYNSKFIYTDTGALASSCQNGWSTTTTHLILTPR--LVFKGEPKNLLYPIYITTELYDVTLNPSDKLI 1804
Ymo1   LYPDEIWNKVRVKSGHSIYEKGKSVKDCRTSAGFWNDTHHVNLNWG----VGIDLGNNQLLEGMEVKLRIDKVSSSYISP  910
Yen2   LEPIDIWQNAIFTRNYSLHNNDGSKRDSCPIVNNLFYPNYARLNWR----MQLVLNKDMLHP-----MQITKLES-KTSK 1929
Sen2   --------------------------------------------------------------------------------  269
Efe4   --------------------------------------------------------------------------------  263
Bpe1   FDKG------------------------------TGKYMLLLAP-------------------VNFPY------------  836
Bav2   SAKDVEIENGDQAGFWLTVTHSVGSYGYSLLSIQTSGKVSVKLLPSDPSDRNQQELVKVIALQVSGPYAWSG-------A 1137
Bpa2   SAKDFSQSRYFVPLTTRGGTDNEPLLRITNQQIP-TAELRVALKPYKTEDRAGRLVREAITLKWGNAPGVGGDARALHFY 1427
Bav1   --------------------------------------------------------------------------------  347
Yin1   --------------------------------------------------------------------------------  269
Yfr4   --------------------------------------------------------------------------------  269
Yin2   --------------------------------------------------------------------------------  270
Ymo2   --------------------------------------------------------------------------------  289
Eco10  --------------------------------------------------------------------------------  291
Eal1   --------------------------------------------------------------------------------  290
Eco16  --------------------------------------------------------------------------------  295
Sty4   --------------------------------------------------------------------------------  290
Yfr2   --------------------------------------------------------------------------------  443
Ype5   --------------------------------------------------------------------------------  628
Eco6   EVELLSVNGVKFRATDGFPETGFDGAKFTLLLTHNMKNTDYNWTAGIYGINVDSNGEVTLSVLIRSEVTITGKPKNGKGN 2231
Bbr1   --------------------------------------------------------------------------------  519
Yps2   --------------------------------------------------------------------------------  529
Eco14  YSSTTVISGNGVKSTIAYLFPGGKEGLLTGIRGYYACR------------------------------------------  691
Sen3   NSSMSKSGVKFINVIEDTWYVKSGSSTYTSSAIKGYPDLGFVVASPTTGPTYLEWSPTGTSKVTTPVTLIDDAGQQYTVN 1103
Pal3   --------------------------------------------------------------------------------   86
Ybe1   --------------------------------------------------------------------------------  505
Pal2   NSLELAIDNNNITLGENVMFTLSTKDIYGNNVMIKPADIHLNNANGTVNQPTWKEQNGEYKGEMILSISGNYVITANVGT 2000
Eco1   --------------------------------------------------------------------------------  420
Yps7   TLKVRAESGDTGMENVIFNVLTSPNTSLANMWGYMSEVIINNGITFKRPLLKAEDTSGDSSANSSSNYANEIWAVFTNTA  915
Yfr3   --------------------------------------------------------------------------------  425
Eco20  AKVEQANGQVSTKTAESKFVADDKNAVLAASPERVDSLVADGKTTATMTVTLMAGVNPVGGSMWVDIEAPEGVTEKDYQF  935
Efe5   MEPGYVTDDGEPFGFIARLTDKFGNPMTGEAEFTAGNEKYLDSADKITMTPAKVTLSWTGHAYSEIRTYLPGRTWVKVKV  755
Csu1   IVGGTTWRRQIADSVMLDYIRKHQMQSKMVQDKIPSTVGLGIGGSATFKALSKEDKGKRKQNYTTSSLVSQPRSFVGNFE  882
Ahy1   --------------------------------------------------------------------------------  403
Plu1   TIAGYTGVYDSSNRNVVDVDGHYFQVKKAGTATLTTTFTHPESGRYLKYIIPDVKIDHFVIVDSSSTGPGIGSAYSKDRI 1154
Cla1   TLVGHNGNFDLGIYGYKNIEDIRNANIKITYTPAYGGNKQWNLNYFISSKIYYLGNTYLPFDMNHYTYLLFSKEEASIPT  739
Pas1   --------------------------------------------------------------------------------  505
ruler  ......3290......3300......3310......3320......3330......3340......3350......3360
```

```
Eca1   --------------------------------------------------------------------------------   53
Esp2   --------------------------------------------------------------------------------   53
Cko1   --------------------------------------------------------------------------------   53
Sen1   --------------------------------------------------------------------------------   53
Eco3   --------------------------------------------------------------------------------   49
Esa3   --------------------------------------------------------------------------------   54
Kpn1   --------------------------------------------------------------------------------   51
Pan1   --------------------------------------------------------------------------------   61
Eta2   --------------------------------------------------------------------------------   52
Spr1   --------------------------------------------------------------------------------   69
Eco25  AENFCSS--NGYTLPLGDHLNHSRS-RGIGSLYSEWGDMGHYTTEAGFHSNMYWSSSPANSNEQYVVSLATGDQSVFEKL   2159
Eco15  ANALCHAKNDGYNLPGITHLTSGENKRTQGSLYGEWGNVGAFSSNSQFTPGAYWTSESDDYSRHYYVQMLTGMTGSDADS   1241
Yfr1   --------------------------------------------------------------------------------   296
Yfr5   --------------------------------------------------------------------------------   345
Yps4   VDTVNANIDTAFVAGAVATITLTTPVNGAVANGADSNSVQAVVSDSGGNVVAGATVVFSSTNTTAQVTTVIGTTGADGIA   2624
Efe2   --------------------------------------------------------------------------------   683
Efe3   QAIDRCGGAENLPLLKDLTSATALGQPGIRGIGNLHGEWGNINRILNTSIFNVWTNEIFPPVYYYIVNVSVGIPNMQSEN   1688
Eta1   HYRNGQFPLTQRNIYSDLSVNFGTKQIARECWKGHDGS-----YFIGAKVNYNGENFEYWMVDPHNWTGKGVGTNKYYLD   867
Eco26  YFTFS--GLSEKNIVN--SSSWSGDDKKGECWKDYWGS-----YNTYMEVQYNQKNYIYRSDSARGWQGMDNNNGPYTDN   1166
Pru1   ISYPEISTPNTNDDYVYSNNDIYRKVLKNNCYENHSGS-----GNITTTLSFLGS--SDELKHHFSWNGKNN--------   1882
Sgl1   --------------------------------------------------------------------------------   447
Pmi1   GHINEKLTYPDKIIFSEHSKKIAEISPTSAHYALDEECRINDSGHGTLKANITIGNRSSYLKKEFHWDGQIGGYF-----   1879
Ymo1   KDIVTRNILKTFDEVSTPSSNYIAETLHTDCYDPHNDS-----VELVLDVEYLGKKTMYKAG-SVYWEGLGRTKKDFVEA   984
Yen2   HGINMTHIDSSTSEIFDSYDNKDDNRLINKCIKEKYGT-----YKTYMDIKYAGREYKYEAINDLYWEGEGDDRESDKSS   2004
Sen2   --------------------------------------------------------------------------------   269
Efe4   --------------------------------------------------------------------------------   263
Bpe1   --------------------------------------------------------------------------------   836
Bav2   VNVSADKYIDQNPPSWVPAGTYPVKIVVTQIATGATVEVSGTIVYSKTAV------------------------------   1187
Bpa2   VPDSAKAKLRKELTEVERSNDYKFWIEFTDTRFGGVARLYIKVISTL---------------------------------   1474
Bav1   --------------------------------------------------------------------------------   347
Yin1   --------------------------------------------------------------------------------   269
Yfr4   --------------------------------------------------------------------------------   269
Yin2   --------------------------------------------------------------------------------   270
Ymo2   --------------------------------------------------------------------------------   289
Eco10  --------------------------------------------------------------------------------   291
Eal1   --------------------------------------------------------------------------------   290
Eco16  --------------------------------------------------------------------------------   295
Sty4   --------------------------------------------------------------------------------   290
Yfr2   --------------------------------------------------------------------------------   443
Ype5   --------------------------------------------------------------------------------   628
Eco6   DVVFKFKIKKWFTSLGATSSNTWDIINTSCSYGQMPSSLELAQRPSGGVVPRKVGTLWGEYGNLKTYGNAFSGTDYWTST   2311
Bbr1   --------------------------------------------------------------------------------   519
Yps2   --------------------------------------------------------------------------------   529
Eco14  --------------------------------------------------------------------------------   691
Sen3   LKGNRTSDCSTRPLNAAVGCSSSAGYKAQFTWNINDNKSIPPGHYTGLIHFYGKDWHTAWAFEYRLTMDLTIN-------   1176
Pal3   --------------------------------------------------------------------------------   86
Ybe1   --------------------------------------------------------------------------------   505
Pal2   QISAPINLTVQAGTPVFATGKSQLSVNRDDLDENSSTNAIVTLELKDANGVAIKGKKPHIQATAGKIDRIMIETTDGVYT   2080
Eco1   --------------------------------------------------------------------------------   420
Yps7   ARAVCSSLPTRPELSGLYSNYPTGSLTTTHGWRTDFYRFRSNTPQSTGFSAVNFLNGTFSSADNTSYDYVSCKG------   989
Yfr3   --------------------------------------------------------------------------------   425
Eco20  LPSKADHFSGGKITRTFSTSKPGVYTFTFNALTYGGYEMTPVKVTINAVAAETENGEEEMP-------------------   996
Efe5   TRGDKTYEQTEETWVSELPKNPQQ--------------------------------------------------------   779
Csu1   KAILMMKLGGGITDSEWRVAWGRDVLFTNPLGRMPAWMYTTKGDQPWEEFFRKSGGPFFWTAFK----------------   946
Ahy1   --------------------------------------------------------------------------------   403
Plu1   DTSKPLPSCTRGNRIKESDLGGSIDYLVKDLNLNLIDKGLLGDPRASLNSNGITMGGLQINDESIQVHLLKEKDPNAIFY   1234
Cla1   KQNNGIYKFEDLNNKYIKPTYLHLKLP-----------------------------------------------------   766
Pas1   --------------------------------------------------------------------------------   505
ruler  ......3370......3380......3390......3400......3410......3420......3430......3440
```

```
   Eca1 --------------------------------------------------------------------------------   53
   Esp2 --------------------------------------------------------------------------------   53
   Cko1 --------------------------------------------------------------------------------   53
   Sen1 --------------------------------------------------------------------------------   53
   Eco3 --------------------------------------------------------------------------------   49
   Esa3 --------------------------------------------------------------------------------   54
   Kpn1 --------------------------------------------------------------------------------   51
   Pan1 --------------------------------------------------------------------------------   61
   Eta2 --------------------------------------------------------------------------------   52
   Spr1 --------------------------------------------------------------------------------   69
  Eco25 GFAYATCYKNL---------------------------------------------------------------------- 2170
  Eco15 SPQLTACRKSL---------------------------------------------------------------------- 1252
   Yfr1 --------------------------------------------------------------------------------  296
   Yfr5 --------------------------------------------------------------------------------  345
   Yps4 TATLTNTVAGTSNVVATVDTVNANIDTTFVAGAVATITLSVLVNDATADGADTNQVDALVQDANGNAITGAAVVFSSANG 2704
   Efe2 --------------------------------------------------------------------------------  683
   Efe3 AHINTICRTIL---------------------------------------------------------------------- 1699
   Eta1 TVTKERNRAAINGS------------------------------------------------------------------  881
  Eco26 MIYVK--------------------------------------------------------------------------- 1171
   Pru1 -------------------------------------------------------------------------------- 1882
   Sgl1 --------------------------------------------------------------------------------  447
   Pmi1 -------------------------------------------------------------------------------- 1879
   Ymo1 NIQRK---------------------------------------------------------------------------  989
   Yen2 GFKKVP-------------------------------------------------------------------------- 2010
   Sen2 --------------------------------------------------------------------------------  269
   Efe4 --------------------------------------------------------------------------------  263
   Bpe1 --------------------------------------------------------------------------------  836
   Bav2 -------------------------------------------------------------------------------- 1187
   Bpa2 -------------------------------------------------------------------------------- 1474
   Bav1 --------------------------------------------------------------------------------  347
   Yin1 --------------------------------------------------------------------------------  269
   Yfr4 --------------------------------------------------------------------------------  269
   Yin2 --------------------------------------------------------------------------------  270
   Ymo2 --------------------------------------------------------------------------------  289
  Eco10 --------------------------------------------------------------------------------  291
   Eal1 --------------------------------------------------------------------------------  290
  Eco16 --------------------------------------------------------------------------------  295
   Sty4 --------------------------------------------------------------------------------  290
   Yfr2 --------------------------------------------------------------------------------  443
   Ype5 --------------------------------------------------------------------------------  628
   Eco6 QLMGVHEKFNPETGISELGTGKSSGLCVEYY------------------------------------------------- 2342
   Bbr1 --------------------------------------------------------------------------------  519
   Yps2 --------------------------------------------------------------------------------  529
  Eco14 --------------------------------------------------------------------------------  691
   Sen3 -------------------------------------------------------------------------------- 1176
   Pal3 --------------------------------------------------------------------------------   86
   Ybe1 --------------------------------------------------------------------------------  505
   Pal2 ANFNNPVVGESTIYLDNASIDYSGSTP----------------------------------------------------- 2107
   Eco1 --------------------------------------------------------------------------------  420
   Yps7 --------------------------------------------------------------------------------  989
   Yfr3 --------------------------------------------------------------------------------  425
  Eco20 --------------------------------------------------------------------------------  996
   Efe5 --------------------------------------------------------------------------------  779
   Csu1 --------------------------------------------------------------------------------  946
   Ahy1 --------------------------------------------------------------------------------  403
   Plu1 VVLCEE-------------------------------------------------------------------------- 1240
   Cla1 --------------------------------------------------------------------------------  766
   Pas1 --------------------------------------------------------------------------------  505
  ruler ......3450......3460......3470......3480......3490......3500......3510......3520
```

```
Eca1   --------------------------------------------------------------------------------   53
Esp2   --------------------------------------------------------------------------------   53
Cko1   --------------------------------------------------------------------------------   53
Sen1   --------------------------------------------------------------------------------   53
Eco3   --------------------------------------------------------------------------------   49
Esa3   --------------------------------------------------------------------------------   54
Kpn1   --------------------------------------------------------------------------------   51
Pan1   --------------------------------------------------------------------------------   61
Eta2   --------------------------------------------------------------------------------   52
Spr1   --------------------------------------------------------------------------------   69
Eco25  --------------------------------------------------------------------------------   2170
Eco15  --------------------------------------------------------------------------------   1252
Yfr1   --------------------------------------------------------------------------------   296
Yfr5   --------------------------------------------------------------------------------   345
Yps4   ADIIAPTMNTGVNGVASTLLTHTMAGTSNVIATIDTVNANIDTTFVAGAVATITLSVPVNDATADGADTNQVDALVQDAN   2784
Efe2   --------------------------------------------------------------------------------   683
Efe3   --------------------------------------------------------------------------------   1699
Eta1   --------------------------------------------------------------------------------   881
Eco26  --------------------------------------------------------------------------------   1171
Pru1   --------------------------------------------------------------------------------   1882
Sgl1   --------------------------------------------------------------------------------   447
Pmi1   --------------------------------------------------------------------------------   1879
Ymo1   --------------------------------------------------------------------------------   989
Yen2   --------------------------------------------------------------------------------   2010
Sen2   --------------------------------------------------------------------------------   269
Efe4   --------------------------------------------------------------------------------   263
Bpe1   --------------------------------------------------------------------------------   836
Bav2   --------------------------------------------------------------------------------   1187
Bpa2   --------------------------------------------------------------------------------   1474
Bav1   --------------------------------------------------------------------------------   347
Yin1   --------------------------------------------------------------------------------   269
Yfr4   --------------------------------------------------------------------------------   269
Yin2   --------------------------------------------------------------------------------   270
Ymo2   --------------------------------------------------------------------------------   289
Eco10  --------------------------------------------------------------------------------   291
Eal1   --------------------------------------------------------------------------------   290
Eco16  --------------------------------------------------------------------------------   295
Sty4   --------------------------------------------------------------------------------   290
Yfr2   --------------------------------------------------------------------------------   443
Ype5   --------------------------------------------------------------------------------   628
Eco6   --------------------------------------------------------------------------------   2342
Bbr1   --------------------------------------------------------------------------------   519
Yps2   --------------------------------------------------------------------------------   529
Eco14  --------------------------------------------------------------------------------   691
Sen3   --------------------------------------------------------------------------------   1176
Pal3   --------------------------------------------------------------------------------   86
Ybe1   --------------------------------------------------------------------------------   505
Pal2   --------------------------------------------------------------------------------   2107
Eco1   --------------------------------------------------------------------------------   420
Yps7   --------------------------------------------------------------------------------   989
Yfr3   --------------------------------------------------------------------------------   425
Eco20  --------------------------------------------------------------------------------   996
Efe5   --------------------------------------------------------------------------------   779
Csu1   --------------------------------------------------------------------------------   946
Ahy1   --------------------------------------------------------------------------------   403
Plu1   --------------------------------------------------------------------------------   1240
Cla1   --------------------------------------------------------------------------------   766
Pas1   --------------------------------------------------------------------------------   505
ruler  ......3530......3540......3550......3560......3570......3580......3590......3600
```

```
   Eca1 --------------------------------------------------------------------------------   53
   Esp2 --------------------------------------------------------------------------------   53
   Cko1 --------------------------------------------------------------------------------   53
   Sen1 --------------------------------------------------------------------------------   53
   Eco3 --------------------------------------------------------------------------------   49
   Esa3 --------------------------------------------------------------------------------   54
   Kpn1 --------------------------------------------------------------------------------   51
   Pan1 --------------------------------------------------------------------------------   61
   Eta2 --------------------------------------------------------------------------------   52
   Spr1 --------------------------------------------------------------------------------   69
  Eco25 -------------------------------------------------------------------------------- 2170
  Eco15 -------------------------------------------------------------------------------- 1252
   Yfr1 --------------------------------------------------------------------------------  296
   Yfr5 --------------------------------------------------------------------------------  345
   Yps4 GNAITGAAVVFSSANGATILSSTMNTGVNGVASTLLTHTQSGVSNVVATIDTVNANIDTAFVAGAVATITLTTPVNGAVA 2864
   Efe2 --------------------------------------------------------------------------------  683
   Efe3 -------------------------------------------------------------------------------- 1699
   Eta1 --------------------------------------------------------------------------------  881
  Eco26 -------------------------------------------------------------------------------- 1171
   Pru1 -------------------------------------------------------------------------------- 1882
   Sgl1 --------------------------------------------------------------------------------  447
   Pmi1 -------------------------------------------------------------------------------- 1879
   Ymo1 --------------------------------------------------------------------------------  989
   Yen2 -------------------------------------------------------------------------------- 2010
   Sen2 --------------------------------------------------------------------------------  269
   Efe4 --------------------------------------------------------------------------------  263
   Bpe1 --------------------------------------------------------------------------------  836
   Bav2 -------------------------------------------------------------------------------- 1187
   Bpa2 -------------------------------------------------------------------------------- 1474
   Bav1 --------------------------------------------------------------------------------  347
   Yin1 --------------------------------------------------------------------------------  269
   Yfr4 --------------------------------------------------------------------------------  269
   Yin2 --------------------------------------------------------------------------------  270
   Ymo2 --------------------------------------------------------------------------------  289
  Eco10 --------------------------------------------------------------------------------  291
   Eal1 --------------------------------------------------------------------------------  290
  Eco16 --------------------------------------------------------------------------------  295
   Sty4 --------------------------------------------------------------------------------  290
   Yfr2 --------------------------------------------------------------------------------  443
   Ype5 --------------------------------------------------------------------------------  628
   Eco6 -------------------------------------------------------------------------------- 2342
   Bbr1 --------------------------------------------------------------------------------  519
   Yps2 --------------------------------------------------------------------------------  529
  Eco14 --------------------------------------------------------------------------------  691
   Sen3 -------------------------------------------------------------------------------- 1176
   Pal3 --------------------------------------------------------------------------------   86
   Ybe1 --------------------------------------------------------------------------------  505
   Pal2 -------------------------------------------------------------------------------- 2107
   Eco1 --------------------------------------------------------------------------------  420
   Yps7 --------------------------------------------------------------------------------  989
   Yfr3 --------------------------------------------------------------------------------  425
  Eco20 --------------------------------------------------------------------------------  996
   Efe5 --------------------------------------------------------------------------------  779
   Csu1 --------------------------------------------------------------------------------  946
   Ahy1 --------------------------------------------------------------------------------  403
   Plu1 -------------------------------------------------------------------------------- 1240
   Cla1 --------------------------------------------------------------------------------  766
   Pas1 --------------------------------------------------------------------------------  505
  ruler ......3610......3620......3630......3640......3650......3660......3670......3680
```

```
  Eca1 --------------------------------------------------------------------------------   53
  Esp2 --------------------------------------------------------------------------------   53
  Cko1 --------------------------------------------------------------------------------   53
  Sen1 --------------------------------------------------------------------------------   53
  Eco3 --------------------------------------------------------------------------------   49
  Esa3 --------------------------------------------------------------------------------   54
  Kpn1 --------------------------------------------------------------------------------   51
  Pan1 --------------------------------------------------------------------------------   61
  Eta2 --------------------------------------------------------------------------------   52
  Spr1 --------------------------------------------------------------------------------   69
 Eco25 -------------------------------------------------------------------------------- 2170
 Eco15 -------------------------------------------------------------------------------- 1252
  Yfr1 --------------------------------------------------------------------------------  296
  Yfr5 --------------------------------------------------------------------------------  345
  Yps4 DGANSNSVQAVVTDSGGNPVNGAAVVFSSANATAQITTVIGTTGADGIATATLTNTVAGTSNVAATIDTVNANIDTTFVA 2944
  Efe2 --------------------------------------------------------------------------------  683
  Efe3 -------------------------------------------------------------------------------- 1699
  Eta1 --------------------------------------------------------------------------------  881
 Eco26 -------------------------------------------------------------------------------- 1171
  Pru1 -------------------------------------------------------------------------------- 1882
  Sgl1 --------------------------------------------------------------------------------  447
  Pmi1 -------------------------------------------------------------------------------- 1879
  Ymo1 --------------------------------------------------------------------------------  989
  Yen2 -------------------------------------------------------------------------------- 2010
  Sen2 --------------------------------------------------------------------------------  269
  Efe4 --------------------------------------------------------------------------------  263
  Bpe1 --------------------------------------------------------------------------------  836
  Bav2 -------------------------------------------------------------------------------- 1187
  Bpa2 -------------------------------------------------------------------------------- 1474
  Bav1 --------------------------------------------------------------------------------  347
  Yin1 --------------------------------------------------------------------------------  269
  Yfr4 --------------------------------------------------------------------------------  269
  Yin2 --------------------------------------------------------------------------------  270
  Ymo2 --------------------------------------------------------------------------------  289
 Eco10 --------------------------------------------------------------------------------  291
  Eal1 --------------------------------------------------------------------------------  290
 Eco16 --------------------------------------------------------------------------------  295
  Sty4 --------------------------------------------------------------------------------  290
  Yfr2 --------------------------------------------------------------------------------  443
  Ype5 --------------------------------------------------------------------------------  628
  Eco6 -------------------------------------------------------------------------------- 2342
  Bbr1 --------------------------------------------------------------------------------  519
  Yps2 --------------------------------------------------------------------------------  529
 Eco14 --------------------------------------------------------------------------------  691
  Sen3 -------------------------------------------------------------------------------- 1176
  Pal3 --------------------------------------------------------------------------------   86
  Ybe1 --------------------------------------------------------------------------------  505
  Pal2 -------------------------------------------------------------------------------- 2107
  Eco1 --------------------------------------------------------------------------------  420
  Yps7 --------------------------------------------------------------------------------  989
  Yfr3 --------------------------------------------------------------------------------  425
 Eco20 --------------------------------------------------------------------------------  996
  Efe5 --------------------------------------------------------------------------------  779
  Csu1 --------------------------------------------------------------------------------  946
  Ahy1 --------------------------------------------------------------------------------  403
  Plu1 -------------------------------------------------------------------------------- 1240
  Cla1 --------------------------------------------------------------------------------  766
  Pas1 --------------------------------------------------------------------------------  505
 ruler ......3690......3700......3710......3720......3730......3740......3750......3760
```

```
  Eca1 --------------------------------------------------------------------------------   53
  Esp2 --------------------------------------------------------------------------------   53
  Cko1 --------------------------------------------------------------------------------   53
  Sen1 --------------------------------------------------------------------------------   53
  Eco3 --------------------------------------------------------------------------------   49
  Esa3 --------------------------------------------------------------------------------   54
  Kpn1 --------------------------------------------------------------------------------   51
  Pan1 --------------------------------------------------------------------------------   61
  Eta2 --------------------------------------------------------------------------------   52
  Spr1 --------------------------------------------------------------------------------   69
 Eco25 -------------------------------------------------------------------------------- 2170
 Eco15 -------------------------------------------------------------------------------- 1252
  Yfr1 --------------------------------------------------------------------------------  296
  Yfr5 --------------------------------------------------------------------------------  345
  Yps4 GAVATITLTTPVNGAVADGANSNSVQAVVSDSEGNPVNGATVVFSSINATAQITTVIGTTGVDGIATATLTNTVAGTSNV 3024
  Efe2 --------------------------------------------------------------------------------  683
  Efe3 -------------------------------------------------------------------------------- 1699
  Eta1 --------------------------------------------------------------------------------  881
 Eco26 -------------------------------------------------------------------------------- 1171
  Pru1 -------------------------------------------------------------------------------- 1882
  Sgl1 --------------------------------------------------------------------------------  447
  Pmi1 -------------------------------------------------------------------------------- 1879
  Ymo1 --------------------------------------------------------------------------------  989
  Yen2 -------------------------------------------------------------------------------- 2010
  Sen2 --------------------------------------------------------------------------------  269
  Efe4 --------------------------------------------------------------------------------  263
  Bpe1 --------------------------------------------------------------------------------  836
  Bav2 -------------------------------------------------------------------------------- 1187
  Bpa2 -------------------------------------------------------------------------------- 1474
  Bav1 --------------------------------------------------------------------------------  347
  Yin1 --------------------------------------------------------------------------------  269
  Yfr4 --------------------------------------------------------------------------------  269
  Yin2 --------------------------------------------------------------------------------  270
  Ymo2 --------------------------------------------------------------------------------  289
 Eco10 --------------------------------------------------------------------------------  291
  Eal1 --------------------------------------------------------------------------------  290
 Eco16 --------------------------------------------------------------------------------  295
  Sty4 --------------------------------------------------------------------------------  290
  Yfr2 --------------------------------------------------------------------------------  443
  Ype5 --------------------------------------------------------------------------------  628
  Eco6 -------------------------------------------------------------------------------- 2342
  Bbr1 --------------------------------------------------------------------------------  519
  Yps2 --------------------------------------------------------------------------------  529
 Eco14 --------------------------------------------------------------------------------  691
  Sen3 -------------------------------------------------------------------------------- 1176
  Pal3 --------------------------------------------------------------------------------   86
  Ybe1 --------------------------------------------------------------------------------  505
  Pal2 -------------------------------------------------------------------------------- 2107
  Eco1 --------------------------------------------------------------------------------  420
  Yps7 --------------------------------------------------------------------------------  989
  Yfr3 --------------------------------------------------------------------------------  425
 Eco20 --------------------------------------------------------------------------------  996
  Efe5 --------------------------------------------------------------------------------  779
  Csu1 --------------------------------------------------------------------------------  946
  Ahy1 --------------------------------------------------------------------------------  403
  Plu1 -------------------------------------------------------------------------------- 1240
  Cla1 --------------------------------------------------------------------------------  766
  Pas1 --------------------------------------------------------------------------------  505
 ruler ......3770......3780......3790......3800......3810......3820......3830......3840
```

```
 Eca1 --------------------------------------------------------------------------------   53
 Esp2 --------------------------------------------------------------------------------   53
 Cko1 --------------------------------------------------------------------------------   53
 Sen1 --------------------------------------------------------------------------------   53
 Eco3 --------------------------------------------------------------------------------   49
 Esa3 --------------------------------------------------------------------------------   54
 Kpn1 --------------------------------------------------------------------------------   51
 Pan1 --------------------------------------------------------------------------------   61
 Eta2 --------------------------------------------------------------------------------   52
 Spr1 --------------------------------------------------------------------------------   69
Eco25 -------------------------------------------------------------------------------- 2170
Eco15 -------------------------------------------------------------------------------- 1252
 Yfr1 --------------------------------------------------------------------------------  296
 Yfr5 --------------------------------------------------------------------------------  345
 Yps4 VATIDTVNANIDTTFVAGAVATITLTTLVNGAVADGANSNSVQAVVSDSGGNPVTGAAVVFSSANATAQITTVIGTTGVD 3104
 Efe2 --------------------------------------------------------------------------------  683
 Efe3 -------------------------------------------------------------------------------- 1699
 Eta1 --------------------------------------------------------------------------------  881
Eco26 -------------------------------------------------------------------------------- 1171
 Pru1 -------------------------------------------------------------------------------- 1882
 Sgl1 --------------------------------------------------------------------------------  447
 Pmi1 -------------------------------------------------------------------------------- 1879
 Ymo1 --------------------------------------------------------------------------------  989
 Yen2 -------------------------------------------------------------------------------- 2010
 Sen2 --------------------------------------------------------------------------------  269
 Efe4 --------------------------------------------------------------------------------  263
 Bpe1 --------------------------------------------------------------------------------  836
 Bav2 -------------------------------------------------------------------------------- 1187
 Bpa2 -------------------------------------------------------------------------------- 1474
 Bav1 --------------------------------------------------------------------------------  347
 Yin1 --------------------------------------------------------------------------------  269
 Yfr4 --------------------------------------------------------------------------------  269
 Yin2 --------------------------------------------------------------------------------  270
 Ymo2 --------------------------------------------------------------------------------  289
Eco10 --------------------------------------------------------------------------------  291
 Eal1 --------------------------------------------------------------------------------  290
Eco16 --------------------------------------------------------------------------------  295
 Sty4 --------------------------------------------------------------------------------  290
 Yfr2 --------------------------------------------------------------------------------  443
 Ype5 --------------------------------------------------------------------------------  628
 Eco6 -------------------------------------------------------------------------------- 2342
 Bbr1 --------------------------------------------------------------------------------  519
 Yps2 --------------------------------------------------------------------------------  529
Eco14 --------------------------------------------------------------------------------  691
 Sen3 -------------------------------------------------------------------------------- 1176
 Pal3 --------------------------------------------------------------------------------   86
 Ybe1 --------------------------------------------------------------------------------  505
 Pal2 -------------------------------------------------------------------------------- 2107
 Eco1 --------------------------------------------------------------------------------  420
 Yps7 --------------------------------------------------------------------------------  989
 Yfr3 --------------------------------------------------------------------------------  425
Eco20 --------------------------------------------------------------------------------  996
 Efe5 --------------------------------------------------------------------------------  779
 Csu1 --------------------------------------------------------------------------------  946
 Ahy1 --------------------------------------------------------------------------------  403
 Plu1 -------------------------------------------------------------------------------- 1240
 Cla1 --------------------------------------------------------------------------------  766
 Pas1 --------------------------------------------------------------------------------  505
ruler ......3850......3860......3870......3880......3890......3900......3910......3920
```

```
  Eca1 --------------------------------------------------------------------------------   53
  Esp2 --------------------------------------------------------------------------------   53
  Cko1 --------------------------------------------------------------------------------   53
  Sen1 --------------------------------------------------------------------------------   53
  Eco3 --------------------------------------------------------------------------------   49
  Esa3 --------------------------------------------------------------------------------   54
  Kpn1 --------------------------------------------------------------------------------   51
  Pan1 --------------------------------------------------------------------------------   61
  Eta2 --------------------------------------------------------------------------------   52
  Spr1 --------------------------------------------------------------------------------   69
 Eco25 -------------------------------------------------------------------------------- 2170
 Eco15 -------------------------------------------------------------------------------- 1252
  Yfr1 --------------------------------------------------------------------------------  296
  Yfr5 --------------------------------------------------------------------------------  345
  Yps4 GIATATLTNTVAGTSNVVATIGSITNNIDTAFVAGAVATITLTTPVNGAVADGANSNSVQAVVTDSGGNPVNGAAVVFSS 3184
  Efe2 --------------------------------------------------------------------------------  683
  Efe3 -------------------------------------------------------------------------------- 1699
  Eta1 --------------------------------------------------------------------------------  881
 Eco26 -------------------------------------------------------------------------------- 1171
  Pru1 -------------------------------------------------------------------------------- 1882
  Sgl1 --------------------------------------------------------------------------------  447
  Pmi1 -------------------------------------------------------------------------------- 1879
  Ymo1 --------------------------------------------------------------------------------  989
  Yen2 -------------------------------------------------------------------------------- 2010
  Sen2 --------------------------------------------------------------------------------  269
  Efe4 --------------------------------------------------------------------------------  263
  Bpe1 --------------------------------------------------------------------------------  836
  Bav2 -------------------------------------------------------------------------------- 1187
  Bpa2 -------------------------------------------------------------------------------- 1474
  Bav1 --------------------------------------------------------------------------------  347
  Yin1 --------------------------------------------------------------------------------  269
  Yfr4 --------------------------------------------------------------------------------  269
  Yin2 --------------------------------------------------------------------------------  270
  Ymo2 --------------------------------------------------------------------------------  289
 Eco10 --------------------------------------------------------------------------------  291
  Eal1 --------------------------------------------------------------------------------  290
 Eco16 --------------------------------------------------------------------------------  295
  Sty4 --------------------------------------------------------------------------------  290
  Yfr2 --------------------------------------------------------------------------------  443
  Ype5 --------------------------------------------------------------------------------  628
  Eco6 -------------------------------------------------------------------------------- 2342
  Bbr1 --------------------------------------------------------------------------------  519
  Yps2 --------------------------------------------------------------------------------  529
 Eco14 --------------------------------------------------------------------------------  691
  Sen3 -------------------------------------------------------------------------------- 1176
  Pal3 --------------------------------------------------------------------------------   86
  Ybe1 --------------------------------------------------------------------------------  505
  Pal2 -------------------------------------------------------------------------------- 2107
  Eco1 --------------------------------------------------------------------------------  420
  Yps7 --------------------------------------------------------------------------------  989
  Yfr3 --------------------------------------------------------------------------------  425
 Eco20 --------------------------------------------------------------------------------  996
  Efe5 --------------------------------------------------------------------------------  779
  Csu1 --------------------------------------------------------------------------------  946
  Ahy1 --------------------------------------------------------------------------------  403
  Plu1 -------------------------------------------------------------------------------- 1240
  Cla1 --------------------------------------------------------------------------------  766
  Pas1 --------------------------------------------------------------------------------  505
 ruler ......3930......3940......3950......3960......3970......3980......3990......4000
```

```
   Eca1 --------------------------------------------------------------------------------   53
   Esp2 --------------------------------------------------------------------------------   53
   Cko1 --------------------------------------------------------------------------------   53
   Sen1 --------------------------------------------------------------------------------   53
   Eco3 --------------------------------------------------------------------------------   49
   Esa3 --------------------------------------------------------------------------------   54
   Kpn1 --------------------------------------------------------------------------------   51
   Pan1 --------------------------------------------------------------------------------   61
   Eta2 --------------------------------------------------------------------------------   52
   Spr1 --------------------------------------------------------------------------------   69
  Eco25 --------------------------------------------------------------------------------  2170
  Eco15 --------------------------------------------------------------------------------  1252
   Yfr1 --------------------------------------------------------------------------------  296
   Yfr5 --------------------------------------------------------------------------------  345
   Yps4 ANATAQITTVIGTTGADGIATATLTNTVAGTSNVIATIDTVNANIDTTFVAGAVATITLTTPVNGAVADGADSNSVQAVV  3264
   Efe2 --------------------------------------------------------------------------------  683
   Efe3 --------------------------------------------------------------------------------  1699
   Eta1 --------------------------------------------------------------------------------  881
  Eco26 --------------------------------------------------------------------------------  1171
   Pru1 --------------------------------------------------------------------------------  1882
   Sgl1 --------------------------------------------------------------------------------  447
   Pmi1 --------------------------------------------------------------------------------  1879
   Ymo1 --------------------------------------------------------------------------------  989
   Yen2 --------------------------------------------------------------------------------  2010
   Sen2 --------------------------------------------------------------------------------  269
   Efe4 --------------------------------------------------------------------------------  263
   Bpe1 --------------------------------------------------------------------------------  836
   Bav2 --------------------------------------------------------------------------------  1187
   Bpa2 --------------------------------------------------------------------------------  1474
   Bav1 --------------------------------------------------------------------------------  347
   Yin1 --------------------------------------------------------------------------------  269
   Yfr4 --------------------------------------------------------------------------------  269
   Yin2 --------------------------------------------------------------------------------  270
   Ymo2 --------------------------------------------------------------------------------  289
  Eco10 --------------------------------------------------------------------------------  291
   Eal1 --------------------------------------------------------------------------------  290
  Eco16 --------------------------------------------------------------------------------  295
   Sty4 --------------------------------------------------------------------------------  290
   Yfr2 --------------------------------------------------------------------------------  443
   Ype5 --------------------------------------------------------------------------------  628
   Eco6 --------------------------------------------------------------------------------  2342
   Bbr1 --------------------------------------------------------------------------------  519
   Yps2 --------------------------------------------------------------------------------  529
  Eco14 --------------------------------------------------------------------------------  691
   Sen3 --------------------------------------------------------------------------------  1176
   Pal3 --------------------------------------------------------------------------------   86
   Ybe1 --------------------------------------------------------------------------------  505
   Pal2 --------------------------------------------------------------------------------  2107
   Eco1 --------------------------------------------------------------------------------  420
   Yps7 --------------------------------------------------------------------------------  989
   Yfr3 --------------------------------------------------------------------------------  425
  Eco20 --------------------------------------------------------------------------------  996
   Efe5 --------------------------------------------------------------------------------  779
   Csu1 --------------------------------------------------------------------------------  946
   Ahy1 --------------------------------------------------------------------------------  403
   Plu1 --------------------------------------------------------------------------------  1240
   Cla1 --------------------------------------------------------------------------------  766
   Pas1 --------------------------------------------------------------------------------  505
  ruler ......4010......4020......4030......4040......4050......4060......4070......4080
```

```
Eca1   --------------------------------------------------------------------------------   53
Esp2   --------------------------------------------------------------------------------   53
Cko1   --------------------------------------------------------------------------------   53
Sen1   --------------------------------------------------------------------------------   53
Eco3   --------------------------------------------------------------------------------   49
Esa3   --------------------------------------------------------------------------------   54
Kpn1   --------------------------------------------------------------------------------   51
Pan1   --------------------------------------------------------------------------------   61
Eta2   --------------------------------------------------------------------------------   52
Spr1   --------------------------------------------------------------------------------   69
Eco25  --------------------------------------------------------------------------------   2170
Eco15  --------------------------------------------------------------------------------   1252
Yfr1   --------------------------------------------------------------------------------   296
Yfr5   --------------------------------------------------------------------------------   345
Yps4   SDSEGNAVTGAAVVFSSANATAQITTVIGTTGADGIATATLTNTVAGTSNVVATIDTVNANIDTAFVAGELENIVVSIIN   3344
Efe2   --------------------------------------------------------------------------------   683
Efe3   --------------------------------------------------------------------------------   1699
Eta1   --------------------------------------------------------------------------------   881
Eco26  --------------------------------------------------------------------------------   1171
Pru1   --------------------------------------------------------------------------------   1882
Sgl1   --------------------------------------------------------------------------------   447
Pmi1   --------------------------------------------------------------------------------   1879
Ymo1   --------------------------------------------------------------------------------   989
Yen2   --------------------------------------------------------------------------------   2010
Sen2   --------------------------------------------------------------------------------   269
Efe4   --------------------------------------------------------------------------------   263
Bpe1   --------------------------------------------------------------------------------   836
Bav2   --------------------------------------------------------------------------------   1187
Bpa2   --------------------------------------------------------------------------------   1474
Bav1   --------------------------------------------------------------------------------   347
Yin1   --------------------------------------------------------------------------------   269
Yfr4   --------------------------------------------------------------------------------   269
Yin2   --------------------------------------------------------------------------------   270
Ymo2   --------------------------------------------------------------------------------   289
Eco10  --------------------------------------------------------------------------------   291
Eal1   --------------------------------------------------------------------------------   290
Eco16  --------------------------------------------------------------------------------   295
Sty4   --------------------------------------------------------------------------------   290
Yfr2   --------------------------------------------------------------------------------   443
Ype5   --------------------------------------------------------------------------------   628
Eco6   --------------------------------------------------------------------------------   2342
Bbr1   --------------------------------------------------------------------------------   519
Yps2   --------------------------------------------------------------------------------   529
Eco14  --------------------------------------------------------------------------------   691
Sen3   --------------------------------------------------------------------------------   1176
Pal3   --------------------------------------------------------------------------------   86
Ybe1   --------------------------------------------------------------------------------   505
Pal2   --------------------------------------------------------------------------------   2107
Eco1   --------------------------------------------------------------------------------   420
Yps7   --------------------------------------------------------------------------------   989
Yfr3   --------------------------------------------------------------------------------   425
Eco20  --------------------------------------------------------------------------------   996
Efe5   --------------------------------------------------------------------------------   779
Csu1   --------------------------------------------------------------------------------   946
Ahy1   --------------------------------------------------------------------------------   403
Plu1   --------------------------------------------------------------------------------   1240
Cla1   --------------------------------------------------------------------------------   766
Pas1   --------------------------------------------------------------------------------   505
ruler  ......4090......4100......4110......4120......4130......4140......4150......4160
```

```
  Eca1 --------------------------------------------------------------------------------   53
  Esp2 --------------------------------------------------------------------------------   53
  Cko1 --------------------------------------------------------------------------------   53
  Sen1 --------------------------------------------------------------------------------   53
  Eco3 --------------------------------------------------------------------------------   49
  Esa3 --------------------------------------------------------------------------------   54
  Kpn1 --------------------------------------------------------------------------------   51
  Pan1 --------------------------------------------------------------------------------   61
  Eta2 --------------------------------------------------------------------------------   52
  Spr1 --------------------------------------------------------------------------------   69
 Eco25 -------------------------------------------------------------------------------- 2170
 Eco15 -------------------------------------------------------------------------------- 1252
  Yfr1 --------------------------------------------------------------------------------  296
  Yfr5 --------------------------------------------------------------------------------  345
  Yps4 NNALANGADTNIVEAFVTDRFGNGVANQSLMFGTNGASIVGSSTVTTNIDGRVRVSATHTVAGSSNTVFAISGAHQGYTR 3424
  Efe2 --------------------------------------------------------------------------------  683
  Efe3 -------------------------------------------------------------------------------- 1699
  Eta1 --------------------------------------------------------------------------------  881
 Eco26 -------------------------------------------------------------------------------- 1171
  Pru1 -------------------------------------------------------------------------------- 1882
  Sgl1 --------------------------------------------------------------------------------  447
  Pmi1 -------------------------------------------------------------------------------- 1879
  Ymo1 --------------------------------------------------------------------------------  989
  Yen2 -------------------------------------------------------------------------------- 2010
  Sen2 --------------------------------------------------------------------------------  269
  Efe4 --------------------------------------------------------------------------------  263
  Bpe1 --------------------------------------------------------------------------------  836
  Bav2 -------------------------------------------------------------------------------- 1187
  Bpa2 -------------------------------------------------------------------------------- 1474
  Bav1 --------------------------------------------------------------------------------  347
  Yin1 --------------------------------------------------------------------------------  269
  Yfr4 --------------------------------------------------------------------------------  269
  Yin2 --------------------------------------------------------------------------------  270
  Ymo2 --------------------------------------------------------------------------------  289
 Eco10 --------------------------------------------------------------------------------  291
  Eal1 --------------------------------------------------------------------------------  290
 Eco16 --------------------------------------------------------------------------------  295
  Sty4 --------------------------------------------------------------------------------  290
  Yfr2 --------------------------------------------------------------------------------  443
  Ype5 --------------------------------------------------------------------------------  628
  Eco6 -------------------------------------------------------------------------------- 2342
  Bbr1 --------------------------------------------------------------------------------  519
  Yps2 --------------------------------------------------------------------------------  529
 Eco14 --------------------------------------------------------------------------------  691
  Sen3 -------------------------------------------------------------------------------- 1176
  Pal3 --------------------------------------------------------------------------------   86
  Ybe1 --------------------------------------------------------------------------------  505
  Pal2 -------------------------------------------------------------------------------- 2107
  Eco1 --------------------------------------------------------------------------------  420
  Yps7 --------------------------------------------------------------------------------  989
  Yfr3 --------------------------------------------------------------------------------  425
 Eco20 --------------------------------------------------------------------------------  996
  Efe5 --------------------------------------------------------------------------------  779
  Csu1 --------------------------------------------------------------------------------  946
  Ahy1 --------------------------------------------------------------------------------  403
  Plu1 -------------------------------------------------------------------------------- 1240
  Cla1 --------------------------------------------------------------------------------  766
  Pas1 --------------------------------------------------------------------------------  505
 ruler ......4170......4180......4190......4200......4210......4220......4230......4240
```

```
Eca1   --------------------------------------------------------------------------------    53
Esp2   --------------------------------------------------------------------------------    53
Cko1   --------------------------------------------------------------------------------    53
Sen1   --------------------------------------------------------------------------------    53
Eco3   --------------------------------------------------------------------------------    49
Esa3   --------------------------------------------------------------------------------    54
Kpn1   --------------------------------------------------------------------------------    51
Pan1   --------------------------------------------------------------------------------    61
Eta2   --------------------------------------------------------------------------------    52
Spr1   --------------------------------------------------------------------------------    69
Eco25  --------------------------------------------------------------------------------  2170
Eco15  --------------------------------------------------------------------------------  1252
Yfr1   --------------------------------------------------------------------------------   296
Yfr5   --------------------------------------------------------------------------------   345
Yps4   VTFVADASTAQLKLTSFLDNQLANGKAGNIAQALVTDAYDNPLANQSVSFALDNGAVIESRGDASSASGIVLMRFNNTLA  3504
Efe2   --------------------------------------------------------------------------------   683
Efe3   --------------------------------------------------------------------------------  1699
Eta1   --------------------------------------------------------------------------------   881
Eco26  --------------------------------------------------------------------------------  1171
Pru1   --------------------------------------------------------------------------------  1882
Sgl1   --------------------------------------------------------------------------------   447
Pmi1   --------------------------------------------------------------------------------  1879
Ymo1   --------------------------------------------------------------------------------   989
Yen2   --------------------------------------------------------------------------------  2010
Sen2   --------------------------------------------------------------------------------   269
Efe4   --------------------------------------------------------------------------------   263
Bpe1   --------------------------------------------------------------------------------   836
Bav2   --------------------------------------------------------------------------------  1187
Bpa2   --------------------------------------------------------------------------------  1474
Bav1   --------------------------------------------------------------------------------   347
Yin1   --------------------------------------------------------------------------------   269
Yfr4   --------------------------------------------------------------------------------   269
Yin2   --------------------------------------------------------------------------------   270
Ymo2   --------------------------------------------------------------------------------   289
Eco10  --------------------------------------------------------------------------------   291
Eal1   --------------------------------------------------------------------------------   290
Eco16  --------------------------------------------------------------------------------   295
Sty4   --------------------------------------------------------------------------------   290
Yfr2   --------------------------------------------------------------------------------   443
Ype5   --------------------------------------------------------------------------------   628
Eco6   --------------------------------------------------------------------------------  2342
Bbr1   --------------------------------------------------------------------------------   519
Yps2   --------------------------------------------------------------------------------   529
Eco14  --------------------------------------------------------------------------------   691
Sen3   --------------------------------------------------------------------------------  1176
Pal3   --------------------------------------------------------------------------------    86
Ybe1   --------------------------------------------------------------------------------   505
Pal2   --------------------------------------------------------------------------------  2107
Eco1   --------------------------------------------------------------------------------   420
Yps7   --------------------------------------------------------------------------------   989
Yfr3   --------------------------------------------------------------------------------   425
Eco20  --------------------------------------------------------------------------------   996
Efe5   --------------------------------------------------------------------------------   779
Csu1   --------------------------------------------------------------------------------   946
Ahy1   --------------------------------------------------------------------------------   403
Plu1   --------------------------------------------------------------------------------  1240
Cla1   --------------------------------------------------------------------------------   766
Pas1   --------------------------------------------------------------------------------   505
ruler  ......4250......4260......4270......4280......4290......4300......4310......4320
```

```
  Eca1 --------------------------------------------------------------------------------   53
  Esp2 --------------------------------------------------------------------------------   53
  Cko1 --------------------------------------------------------------------------------   53
  Sen1 --------------------------------------------------------------------------------   53
  Eco3 --------------------------------------------------------------------------------   49
  Esa3 --------------------------------------------------------------------------------   54
  Kpn1 --------------------------------------------------------------------------------   51
  Pan1 --------------------------------------------------------------------------------   61
  Eta2 --------------------------------------------------------------------------------   52
  Spr1 --------------------------------------------------------------------------------   69
 Eco25 -------------------------------------------------------------------------------- 2170
 Eco15 -------------------------------------------------------------------------------- 1252
  Yfr1 --------------------------------------------------------------------------------  296
  Yfr5 --------------------------------------------------------------------------------  345
  Yps4 GMTTVTATLDSTGQTETLEMHFVAGKAASIELTMTKDNAVANNIDTNEVQVLVTDADGNAINGAVVNLTSNSGMNITPNS 3584
  Efe2 --------------------------------------------------------------------------------  683
  Efe3 -------------------------------------------------------------------------------- 1699
  Eta1 --------------------------------------------------------------------------------  881
 Eco26 -------------------------------------------------------------------------------- 1171
  Pru1 -------------------------------------------------------------------------------- 1882
  Sgl1 --------------------------------------------------------------------------------  447
  Pmi1 -------------------------------------------------------------------------------- 1879
  Ymo1 --------------------------------------------------------------------------------  989
  Yen2 -------------------------------------------------------------------------------- 2010
  Sen2 --------------------------------------------------------------------------------  269
  Efe4 --------------------------------------------------------------------------------  263
  Bpe1 --------------------------------------------------------------------------------  836
  Bav2 -------------------------------------------------------------------------------- 1187
  Bpa2 -------------------------------------------------------------------------------- 1474
  Bav1 --------------------------------------------------------------------------------  347
  Yin1 --------------------------------------------------------------------------------  269
  Yfr4 --------------------------------------------------------------------------------  269
  Yin2 --------------------------------------------------------------------------------  270
  Ymo2 --------------------------------------------------------------------------------  289
 Eco10 --------------------------------------------------------------------------------  291
  Eal1 --------------------------------------------------------------------------------  290
 Eco16 --------------------------------------------------------------------------------  295
  Sty4 --------------------------------------------------------------------------------  290
  Yfr2 --------------------------------------------------------------------------------  443
  Ype5 --------------------------------------------------------------------------------  628
  Eco6 -------------------------------------------------------------------------------- 2342
  Bbr1 --------------------------------------------------------------------------------  519
  Yps2 --------------------------------------------------------------------------------  529
 Eco14 --------------------------------------------------------------------------------  691
  Sen3 -------------------------------------------------------------------------------- 1176
  Pal3 --------------------------------------------------------------------------------   86
  Ybe1 --------------------------------------------------------------------------------  505
  Pal2 -------------------------------------------------------------------------------- 2107
  Eco1 --------------------------------------------------------------------------------  420
  Yps7 --------------------------------------------------------------------------------  989
  Yfr3 --------------------------------------------------------------------------------  425
 Eco20 --------------------------------------------------------------------------------  996
  Efe5 --------------------------------------------------------------------------------  779
  Csu1 --------------------------------------------------------------------------------  946
  Ahy1 --------------------------------------------------------------------------------  403
  Plu1 -------------------------------------------------------------------------------- 1240
  Cla1 --------------------------------------------------------------------------------  766
  Pas1 --------------------------------------------------------------------------------  505
 ruler ......4330......4340......4350......4360......4370......4380......4390......4400
```

CLUSTAL X (1.81.1-alpha) MULTIPLE SEQUENCE ALIGNMENT

File: /Users/saierlab/Desktop/58cterm.ps   Date: Tue May 12 18:49:09 2009


```
  Eca1 --------------------------------------------------------------------------------   53
  Esp2 --------------------------------------------------------------------------------   53
  Cko1 --------------------------------------------------------------------------------   53
  Sen1 --------------------------------------------------------------------------------   53
  Eco3 --------------------------------------------------------------------------------   49
  Esa3 --------------------------------------------------------------------------------   54
  Kpn1 --------------------------------------------------------------------------------   51
  Pan1 --------------------------------------------------------------------------------   61
  Eta2 --------------------------------------------------------------------------------   52
  Spr1 --------------------------------------------------------------------------------   69
 Eco25 --------------------------------------------------------------------------------  2170
 Eco15 --------------------------------------------------------------------------------  1252
  Yfr1 --------------------------------------------------------------------------------  296
  Yfr5 --------------------------------------------------------------------------------  345
  Yps4 VTTGSDGTATATLTHTLAGSLPINARIDQVSKTINATFIADVSTAQIIASDMFIIVNDQVANGQAVNAVQARVTDSYGNP  3664
  Efe2 --------------------------------------------------------------------------------  683
  Efe3 --------------------------------------------------------------------------------  1699
  Eta1 --------------------------------------------------------------------------------  881
 Eco26 --------------------------------------------------------------------------------  1171
  Pru1 --------------------------------------------------------------------------------  1882
  Sgl1 --------------------------------------------------------------------------------  447
  Pmi1 --------------------------------------------------------------------------------  1879
  Ymo1 --------------------------------------------------------------------------------  989
  Yen2 --------------------------------------------------------------------------------  2010
  Sen2 --------------------------------------------------------------------------------  269
  Efe4 --------------------------------------------------------------------------------  263
  Bpe1 --------------------------------------------------------------------------------  836
  Bav2 --------------------------------------------------------------------------------  1187
  Bpa2 --------------------------------------------------------------------------------  1474
  Bav1 --------------------------------------------------------------------------------  347
  Yin1 --------------------------------------------------------------------------------  269
  Yfr4 --------------------------------------------------------------------------------  269
  Yin2 --------------------------------------------------------------------------------  270
  Ymo2 --------------------------------------------------------------------------------  289
 Eco10 --------------------------------------------------------------------------------  291
  Eal1 --------------------------------------------------------------------------------  290
 Eco16 --------------------------------------------------------------------------------  295
  Sty4 --------------------------------------------------------------------------------  290
  Yfr2 --------------------------------------------------------------------------------  443
  Ype5 --------------------------------------------------------------------------------  628
  Eco6 --------------------------------------------------------------------------------  2342
  Bbr1 --------------------------------------------------------------------------------  519
  Yps2 --------------------------------------------------------------------------------  529
 Eco14 --------------------------------------------------------------------------------  691
  Sen3 --------------------------------------------------------------------------------  1176
  Pal3 --------------------------------------------------------------------------------   86
  Ybe1 --------------------------------------------------------------------------------  505
  Pal2 --------------------------------------------------------------------------------  2107
  Eco1 --------------------------------------------------------------------------------  420
  Yps7 --------------------------------------------------------------------------------  989
  Yfr3 --------------------------------------------------------------------------------  425
 Eco20 --------------------------------------------------------------------------------  996
  Efe5 --------------------------------------------------------------------------------  779
  Csu1 --------------------------------------------------------------------------------  946
  Ahy1 --------------------------------------------------------------------------------  403
  Plu1 --------------------------------------------------------------------------------  1240
  Cla1 --------------------------------------------------------------------------------  766
  Pas1 --------------------------------------------------------------------------------  505
 ruler ......4410......4420......4430......4440......4450......4460......4470......4480
```

```
Eca1   --------------------------------------------------------------------------------   53
Esp2   --------------------------------------------------------------------------------   53
Cko1   --------------------------------------------------------------------------------   53
Sen1   --------------------------------------------------------------------------------   53
Eco3   --------------------------------------------------------------------------------   49
Esa3   --------------------------------------------------------------------------------   54
Kpn1   --------------------------------------------------------------------------------   51
Pan1   --------------------------------------------------------------------------------   61
Eta2   --------------------------------------------------------------------------------   52
Spr1   --------------------------------------------------------------------------------   69
Eco25  --------------------------------------------------------------------------------   2170
Eco15  --------------------------------------------------------------------------------   1252
Yfr1   --------------------------------------------------------------------------------   296
Yfr5   --------------------------------------------------------------------------------   345
Yps4   IQGQLVEFVLSNTGTIQYKLEETSVEGGVMVTFTNTLAGITNVTATVVSSRSSQNVDTTFIADVTTAHIAESDLMVIVDN   3744
Efe2   --------------------------------------------------------------------------------   683
Efe3   --------------------------------------------------------------------------------   1699
Eta1   --------------------------------------------------------------------------------   881
Eco26  --------------------------------------------------------------------------------   1171
Pru1   --------------------------------------------------------------------------------   1882
Sgl1   --------------------------------------------------------------------------------   447
Pmi1   --------------------------------------------------------------------------------   1879
Ymo1   --------------------------------------------------------------------------------   989
Yen2   --------------------------------------------------------------------------------   2010
Sen2   --------------------------------------------------------------------------------   269
Efe4   --------------------------------------------------------------------------------   263
Bpe1   --------------------------------------------------------------------------------   836
Bav2   --------------------------------------------------------------------------------   1187
Bpa2   --------------------------------------------------------------------------------   1474
Bav1   --------------------------------------------------------------------------------   347
Yin1   --------------------------------------------------------------------------------   269
Yfr4   --------------------------------------------------------------------------------   269
Yin2   --------------------------------------------------------------------------------   270
Ymo2   --------------------------------------------------------------------------------   289
Eco10  --------------------------------------------------------------------------------   291
Eal1   --------------------------------------------------------------------------------   290
Eco16  --------------------------------------------------------------------------------   295
Sty4   --------------------------------------------------------------------------------   290
Yfr2   --------------------------------------------------------------------------------   443
Ype5   --------------------------------------------------------------------------------   628
Eco6   --------------------------------------------------------------------------------   2342
Bbr1   --------------------------------------------------------------------------------   519
Yps2   --------------------------------------------------------------------------------   529
Eco14  --------------------------------------------------------------------------------   691
Sen3   --------------------------------------------------------------------------------   1176
Pal3   --------------------------------------------------------------------------------   86
Ybe1   --------------------------------------------------------------------------------   505
Pal2   --------------------------------------------------------------------------------   2107
Eco1   --------------------------------------------------------------------------------   420
Yps7   --------------------------------------------------------------------------------   989
Yfr3   --------------------------------------------------------------------------------   425
Eco20  --------------------------------------------------------------------------------   996
Efe5   --------------------------------------------------------------------------------   779
Csu1   --------------------------------------------------------------------------------   946
Ahy1   --------------------------------------------------------------------------------   403
Plu1   --------------------------------------------------------------------------------   1240
Cla1   --------------------------------------------------------------------------------   766
Pas1   --------------------------------------------------------------------------------   505
ruler  ......4490......4500......4510......4520......4530......4540......4550......4560
```

```
 Eca1 --------------------------------------------------------------------------------   53
 Esp2 --------------------------------------------------------------------------------   53
 Cko1 --------------------------------------------------------------------------------   53
 Sen1 --------------------------------------------------------------------------------   53
 Eco3 --------------------------------------------------------------------------------   49
 Esa3 --------------------------------------------------------------------------------   54
 Kpn1 --------------------------------------------------------------------------------   51
 Pan1 --------------------------------------------------------------------------------   61
 Eta2 --------------------------------------------------------------------------------   52
 Spr1 --------------------------------------------------------------------------------   69
Eco25 -------------------------------------------------------------------------------- 2170
Eco15 -------------------------------------------------------------------------------- 1252
 Yfr1 --------------------------------------------------------------------------------  296
 Yfr5 --------------------------------------------------------------------------------  345
 Yps4 AVANNSEKNEVHARVTDAKGNVLSGQTVIFTSGNGAAITTVNGISDGDGLTKATLTHTLAGTSVVTARVGNQVQSKDTTF 3824
 Efe2 --------------------------------------------------------------------------------  683
 Efe3 -------------------------------------------------------------------------------- 1699
 Eta1 --------------------------------------------------------------------------------  881
Eco26 -------------------------------------------------------------------------------- 1171
 Pru1 -------------------------------------------------------------------------------- 1882
 Sgl1 --------------------------------------------------------------------------------  447
 Pmi1 -------------------------------------------------------------------------------- 1879
 Ymo1 --------------------------------------------------------------------------------  989
 Yen2 -------------------------------------------------------------------------------- 2010
 Sen2 --------------------------------------------------------------------------------  269
 Efe4 --------------------------------------------------------------------------------  263
 Bpe1 --------------------------------------------------------------------------------  836
 Bav2 -------------------------------------------------------------------------------- 1187
 Bpa2 -------------------------------------------------------------------------------- 1474
 Bav1 --------------------------------------------------------------------------------  347
 Yin1 --------------------------------------------------------------------------------  269
 Yfr4 --------------------------------------------------------------------------------  269
 Yin2 --------------------------------------------------------------------------------  270
 Ymo2 --------------------------------------------------------------------------------  289
Eco10 --------------------------------------------------------------------------------  291
 Eal1 --------------------------------------------------------------------------------  290
Eco16 --------------------------------------------------------------------------------  295
 Sty4 --------------------------------------------------------------------------------  290
 Yfr2 --------------------------------------------------------------------------------  443
 Ype5 --------------------------------------------------------------------------------  628
 Eco6 -------------------------------------------------------------------------------- 2342
 Bbr1 --------------------------------------------------------------------------------  519
 Yps2 --------------------------------------------------------------------------------  529
Eco14 --------------------------------------------------------------------------------  691
 Sen3 -------------------------------------------------------------------------------- 1176
 Pal3 --------------------------------------------------------------------------------   86
 Ybe1 --------------------------------------------------------------------------------  505
 Pal2 -------------------------------------------------------------------------------- 2107
 Eco1 --------------------------------------------------------------------------------  420
 Yps7 --------------------------------------------------------------------------------  989
 Yfr3 --------------------------------------------------------------------------------  425
Eco20 --------------------------------------------------------------------------------  996
 Efe5 --------------------------------------------------------------------------------  779
 Csu1 --------------------------------------------------------------------------------  946
 Ahy1 --------------------------------------------------------------------------------  403
 Plu1 -------------------------------------------------------------------------------- 1240
 Cla1 --------------------------------------------------------------------------------  766
 Pas1 --------------------------------------------------------------------------------  505
ruler ......4570......4580......4590......4600......4610......4620......4630......4640
```

```
   Eca1 --------------------------------------------------------------------------------   53
   Esp2 --------------------------------------------------------------------------------   53
   Cko1 --------------------------------------------------------------------------------   53
   Sen1 --------------------------------------------------------------------------------   53
   Eco3 --------------------------------------------------------------------------------   49
   Esa3 --------------------------------------------------------------------------------   54
   Kpn1 --------------------------------------------------------------------------------   51
   Pan1 --------------------------------------------------------------------------------   61
   Eta2 --------------------------------------------------------------------------------   52
   Spr1 --------------------------------------------------------------------------------   69
  Eco25 -------------------------------------------------------------------------------- 2170
  Eco15 -------------------------------------------------------------------------------- 1252
   Yfr1 --------------------------------------------------------------------------------  296
   Yfr5 --------------------------------------------------------------------------------  345
   Yps4 IADRTTATIRASDLTITRSNALADGVATNAARVIVTDAYGNPVPSMLVSYTSENGATLTPTLGSTDSSGMLSTTFTHTIA 3904
   Efe2 --------------------------------------------------------------------------------  683
   Efe3 -------------------------------------------------------------------------------- 1699
   Eta1 --------------------------------------------------------------------------------  881
  Eco26 -------------------------------------------------------------------------------- 1171
   Pru1 -------------------------------------------------------------------------------- 1882
   Sgl1 --------------------------------------------------------------------------------  447
   Pmi1 -------------------------------------------------------------------------------- 1879
   Ymo1 --------------------------------------------------------------------------------  989
   Yen2 -------------------------------------------------------------------------------- 2010
   Sen2 --------------------------------------------------------------------------------  269
   Efe4 --------------------------------------------------------------------------------  263
   Bpe1 --------------------------------------------------------------------------------  836
   Bav2 -------------------------------------------------------------------------------- 1187
   Bpa2 -------------------------------------------------------------------------------- 1474
   Bav1 --------------------------------------------------------------------------------  347
   Yin1 --------------------------------------------------------------------------------  269
   Yfr4 --------------------------------------------------------------------------------  269
   Yin2 --------------------------------------------------------------------------------  270
   Ymo2 --------------------------------------------------------------------------------  289
  Eco10 --------------------------------------------------------------------------------  291
   Eal1 --------------------------------------------------------------------------------  290
  Eco16 --------------------------------------------------------------------------------  295
   Sty4 --------------------------------------------------------------------------------  290
   Yfr2 --------------------------------------------------------------------------------  443
   Ype5 --------------------------------------------------------------------------------  628
   Eco6 -------------------------------------------------------------------------------- 2342
   Bbr1 --------------------------------------------------------------------------------  519
   Yps2 --------------------------------------------------------------------------------  529
  Eco14 --------------------------------------------------------------------------------  691
   Sen3 -------------------------------------------------------------------------------- 1176
   Pal3 --------------------------------------------------------------------------------   86
   Ybe1 --------------------------------------------------------------------------------  505
   Pal2 -------------------------------------------------------------------------------- 2107
   Eco1 --------------------------------------------------------------------------------  420
   Yps7 --------------------------------------------------------------------------------  989
   Yfr3 --------------------------------------------------------------------------------  425
  Eco20 --------------------------------------------------------------------------------  996
   Efe5 --------------------------------------------------------------------------------  779
   Csu1 --------------------------------------------------------------------------------  946
   Ahy1 --------------------------------------------------------------------------------  403
   Plu1 -------------------------------------------------------------------------------- 1240
   Cla1 --------------------------------------------------------------------------------  766
   Pas1 --------------------------------------------------------------------------------  505
  ruler ......4650......4660......4670......4680......4690......4700......4710......4720
```

```
  Eca1 --------------------------------------------------------------------------------   53
  Esp2 --------------------------------------------------------------------------------   53
  Cko1 --------------------------------------------------------------------------------   53
  Sen1 --------------------------------------------------------------------------------   53
  Eco3 --------------------------------------------------------------------------------   49
  Esa3 --------------------------------------------------------------------------------   54
  Kpn1 --------------------------------------------------------------------------------   51
  Pan1 --------------------------------------------------------------------------------   61
  Eta2 --------------------------------------------------------------------------------   52
  Spr1 --------------------------------------------------------------------------------   69
 Eco25 -------------------------------------------------------------------------------- 2170
 Eco15 -------------------------------------------------------------------------------- 1252
  Yfr1 --------------------------------------------------------------------------------  296
  Yfr5 --------------------------------------------------------------------------------  345
  Yps4 GISKVTATIVTMGISQAKDAVFIADRTTAHVSALTVEKNDSLANNSDRNIVQAHIQDAHGNVITGMNVNFSATENVTLAA 3984
  Efe2 --------------------------------------------------------------------------------  683
  Efe3 -------------------------------------------------------------------------------- 1699
  Eta1 --------------------------------------------------------------------------------  881
 Eco26 -------------------------------------------------------------------------------- 1171
  Pru1 -------------------------------------------------------------------------------- 1882
  Sgl1 --------------------------------------------------------------------------------  447
  Pmi1 -------------------------------------------------------------------------------- 1879
  Ymo1 --------------------------------------------------------------------------------  989
  Yen2 -------------------------------------------------------------------------------- 2010
  Sen2 --------------------------------------------------------------------------------  269
  Efe4 --------------------------------------------------------------------------------  263
  Bpe1 --------------------------------------------------------------------------------  836
  Bav2 -------------------------------------------------------------------------------- 1187
  Bpa2 -------------------------------------------------------------------------------- 1474
  Bav1 --------------------------------------------------------------------------------  347
  Yin1 --------------------------------------------------------------------------------  269
  Yfr4 --------------------------------------------------------------------------------  269
  Yin2 --------------------------------------------------------------------------------  270
  Ymo2 --------------------------------------------------------------------------------  289
 Eco10 --------------------------------------------------------------------------------  291
  Eal1 --------------------------------------------------------------------------------  290
 Eco16 --------------------------------------------------------------------------------  295
  Sty4 --------------------------------------------------------------------------------  290
  Yfr2 --------------------------------------------------------------------------------  443
  Ype5 --------------------------------------------------------------------------------  628
  Eco6 -------------------------------------------------------------------------------- 2342
  Bbr1 --------------------------------------------------------------------------------  519
  Yps2 --------------------------------------------------------------------------------  529
 Eco14 --------------------------------------------------------------------------------  691
  Sen3 -------------------------------------------------------------------------------- 1176
  Pal3 --------------------------------------------------------------------------------   86
  Ybe1 --------------------------------------------------------------------------------  505
  Pal2 -------------------------------------------------------------------------------- 2107
  Eco1 --------------------------------------------------------------------------------  420
  Yps7 --------------------------------------------------------------------------------  989
  Yfr3 --------------------------------------------------------------------------------  425
 Eco20 --------------------------------------------------------------------------------  996
  Efe5 --------------------------------------------------------------------------------  779
  Csu1 --------------------------------------------------------------------------------  946
  Ahy1 --------------------------------------------------------------------------------  403
  Plu1 -------------------------------------------------------------------------------- 1240
  Cla1 --------------------------------------------------------------------------------  766
  Pas1 --------------------------------------------------------------------------------  505
 ruler ......4730......4740......4750......4760......4770......4780......4790......4800
```

```
 Eca1 --------------------------------------------------------------------------------   53
 Esp2 --------------------------------------------------------------------------------   53
 Cko1 --------------------------------------------------------------------------------   53
 Sen1 --------------------------------------------------------------------------------   53
 Eco3 --------------------------------------------------------------------------------   49
 Esa3 --------------------------------------------------------------------------------   54
 Kpn1 --------------------------------------------------------------------------------   51
 Pan1 --------------------------------------------------------------------------------   61
 Eta2 --------------------------------------------------------------------------------   52
 Spr1 --------------------------------------------------------------------------------   69
Eco25 -------------------------------------------------------------------------------- 2170
Eco15 -------------------------------------------------------------------------------- 1252
 Yfr1 --------------------------------------------------------------------------------  296
 Yfr5 --------------------------------------------------------------------------------  345
 Yps4 NMVTTNAQGYAENTLRHNAPVTSAVTATVATDLVGLTEDVRFVAGAGARIELFRLNDGAVADGIQTNRVEARVYDVSDNL 4064
 Efe2 --------------------------------------------------------------------------------  683
 Efe3 -------------------------------------------------------------------------------- 1699
 Eta1 --------------------------------------------------------------------------------  881
Eco26 -------------------------------------------------------------------------------- 1171
 Pru1 -------------------------------------------------------------------------------- 1882
 Sgl1 --------------------------------------------------------------------------------  447
 Pmi1 -------------------------------------------------------------------------------- 1879
 Ymo1 --------------------------------------------------------------------------------  989
 Yen2 -------------------------------------------------------------------------------- 2010
 Sen2 --------------------------------------------------------------------------------  269
 Efe4 --------------------------------------------------------------------------------  263
 Bpe1 --------------------------------------------------------------------------------  836
 Bav2 -------------------------------------------------------------------------------- 1187
 Bpa2 -------------------------------------------------------------------------------- 1474
 Bav1 --------------------------------------------------------------------------------  347
 Yin1 --------------------------------------------------------------------------------  269
 Yfr4 --------------------------------------------------------------------------------  269
 Yin2 --------------------------------------------------------------------------------  270
 Ymo2 --------------------------------------------------------------------------------  289
Eco10 --------------------------------------------------------------------------------  291
 Eal1 --------------------------------------------------------------------------------  290
Eco16 --------------------------------------------------------------------------------  295
 Sty4 --------------------------------------------------------------------------------  290
 Yfr2 --------------------------------------------------------------------------------  443
 Ype5 --------------------------------------------------------------------------------  628
 Eco6 -------------------------------------------------------------------------------- 2342
 Bbr1 --------------------------------------------------------------------------------  519
 Yps2 --------------------------------------------------------------------------------  529
Eco14 --------------------------------------------------------------------------------  691
 Sen3 -------------------------------------------------------------------------------- 1176
 Pal3 --------------------------------------------------------------------------------   86
 Ybe1 --------------------------------------------------------------------------------  505
 Pal2 -------------------------------------------------------------------------------- 2107
 Eco1 --------------------------------------------------------------------------------  420
 Yps7 --------------------------------------------------------------------------------  989
 Yfr3 --------------------------------------------------------------------------------  425
Eco20 --------------------------------------------------------------------------------  996
 Efe5 --------------------------------------------------------------------------------  779
 Csu1 --------------------------------------------------------------------------------  946
 Ahy1 --------------------------------------------------------------------------------  403
 Plu1 -------------------------------------------------------------------------------- 1240
 Cla1 --------------------------------------------------------------------------------  766
 Pas1 --------------------------------------------------------------------------------  505
ruler ......4810......4820......4830......4840......4850......4860......4870......4880
```

```
   Eca1 --------------------------------------------------------------------------------   53
   Esp2 --------------------------------------------------------------------------------   53
   Cko1 --------------------------------------------------------------------------------   53
   Sen1 --------------------------------------------------------------------------------   53
   Eco3 --------------------------------------------------------------------------------   49
   Esa3 --------------------------------------------------------------------------------   54
   Kpn1 --------------------------------------------------------------------------------   51
   Pan1 --------------------------------------------------------------------------------   61
   Eta2 --------------------------------------------------------------------------------   52
   Spr1 --------------------------------------------------------------------------------   69
  Eco25 -------------------------------------------------------------------------------- 2170
  Eco15 -------------------------------------------------------------------------------- 1252
   Yfr1 --------------------------------------------------------------------------------  296
   Yfr5 --------------------------------------------------------------------------------  345
   Yps4 VPNSNVVFSADNGGQLVQNDVQTDALGSAYVTVSNINTGVTKVSVTADGVSASTTTTFIADKDTVTLRADLFLITHDNAV 4144
   Efe2 --------------------------------------------------------------------------------  683
   Efe3 -------------------------------------------------------------------------------- 1699
   Eta1 --------------------------------------------------------------------------------  881
  Eco26 -------------------------------------------------------------------------------- 1171
   Pru1 -------------------------------------------------------------------------------- 1882
   Sgl1 --------------------------------------------------------------------------------  447
   Pmi1 -------------------------------------------------------------------------------- 1879
   Ymo1 --------------------------------------------------------------------------------  989
   Yen2 -------------------------------------------------------------------------------- 2010
   Sen2 --------------------------------------------------------------------------------  269
   Efe4 --------------------------------------------------------------------------------  263
   Bpe1 --------------------------------------------------------------------------------  836
   Bav2 -------------------------------------------------------------------------------- 1187
   Bpa2 -------------------------------------------------------------------------------- 1474
   Bav1 --------------------------------------------------------------------------------  347
   Yin1 --------------------------------------------------------------------------------  269
   Yfr4 --------------------------------------------------------------------------------  269
   Yin2 --------------------------------------------------------------------------------  270
   Ymo2 --------------------------------------------------------------------------------  289
  Eco10 --------------------------------------------------------------------------------  291
   Eal1 --------------------------------------------------------------------------------  290
  Eco16 --------------------------------------------------------------------------------  295
   Sty4 --------------------------------------------------------------------------------  290
   Yfr2 --------------------------------------------------------------------------------  443
   Ype5 --------------------------------------------------------------------------------  628
   Eco6 -------------------------------------------------------------------------------- 2342
   Bbr1 --------------------------------------------------------------------------------  519
   Yps2 --------------------------------------------------------------------------------  529
  Eco14 --------------------------------------------------------------------------------  691
   Sen3 -------------------------------------------------------------------------------- 1176
   Pal3 --------------------------------------------------------------------------------   86
   Ybe1 --------------------------------------------------------------------------------  505
   Pal2 -------------------------------------------------------------------------------- 2107
   Eco1 --------------------------------------------------------------------------------  420
   Yps7 --------------------------------------------------------------------------------  989
   Yfr3 --------------------------------------------------------------------------------  425
  Eco20 --------------------------------------------------------------------------------  996
   Efe5 --------------------------------------------------------------------------------  779
   Csu1 --------------------------------------------------------------------------------  946
   Ahy1 --------------------------------------------------------------------------------  403
   Plu1 -------------------------------------------------------------------------------- 1240
   Cla1 --------------------------------------------------------------------------------  766
   Pas1 --------------------------------------------------------------------------------  505
  ruler ......4890......4900......4910......4920......4930......4940......4950......4960
```

```
  Eca1 -------------------------------------------------------------------------------- 53
  Esp2 -------------------------------------------------------------------------------- 53
  Cko1 -------------------------------------------------------------------------------- 53
  Sen1 -------------------------------------------------------------------------------- 53
  Eco3 -------------------------------------------------------------------------------- 49
  Esa3 -------------------------------------------------------------------------------- 54
  Kpn1 -------------------------------------------------------------------------------- 51
  Pan1 -------------------------------------------------------------------------------- 61
  Eta2 -------------------------------------------------------------------------------- 52
  Spr1 -------------------------------------------------------------------------------- 69
 Eco25 -------------------------------------------------------------------------------- 2170
 Eco15 -------------------------------------------------------------------------------- 1252
  Yfr1 -------------------------------------------------------------------------------- 296
  Yfr5 -------------------------------------------------------------------------------- 345
  Yps4 ANGVTENRVLLQLLDANDNKVSGVEVNFTATNGASINASAITDTNGLAIGVLTNTLSGPSDVTVTLVTPGGTESLTVTPQ 4224
  Efe2 -------------------------------------------------------------------------------- 683
  Efe3 -------------------------------------------------------------------------------- 1699
  Eta1 -------------------------------------------------------------------------------- 881
 Eco26 -------------------------------------------------------------------------------- 1171
  Pru1 -------------------------------------------------------------------------------- 1882
  Sgl1 -------------------------------------------------------------------------------- 447
  Pmi1 -------------------------------------------------------------------------------- 1879
  Ymo1 -------------------------------------------------------------------------------- 989
  Yen2 -------------------------------------------------------------------------------- 2010
  Sen2 -------------------------------------------------------------------------------- 269
  Efe4 -------------------------------------------------------------------------------- 263
  Bpe1 -------------------------------------------------------------------------------- 836
  Bav2 -------------------------------------------------------------------------------- 1187
  Bpa2 -------------------------------------------------------------------------------- 1474
  Bav1 -------------------------------------------------------------------------------- 347
  Yin1 -------------------------------------------------------------------------------- 269
  Yfr4 -------------------------------------------------------------------------------- 269
  Yin2 -------------------------------------------------------------------------------- 270
  Ymo2 -------------------------------------------------------------------------------- 289
 Eco10 -------------------------------------------------------------------------------- 291
  Eal1 -------------------------------------------------------------------------------- 290
 Eco16 -------------------------------------------------------------------------------- 295
  Sty4 -------------------------------------------------------------------------------- 290
  Yfr2 -------------------------------------------------------------------------------- 443
  Ype5 -------------------------------------------------------------------------------- 628
  Eco6 -------------------------------------------------------------------------------- 2342
  Bbr1 -------------------------------------------------------------------------------- 519
  Yps2 -------------------------------------------------------------------------------- 529
 Eco14 -------------------------------------------------------------------------------- 691
  Sen3 -------------------------------------------------------------------------------- 1176
  Pal3 -------------------------------------------------------------------------------- 86
  Ybe1 -------------------------------------------------------------------------------- 505
  Pal2 -------------------------------------------------------------------------------- 2107
  Eco1 -------------------------------------------------------------------------------- 420
  Yps7 -------------------------------------------------------------------------------- 989
  Yfr3 -------------------------------------------------------------------------------- 425
 Eco20 -------------------------------------------------------------------------------- 996
  Efe5 -------------------------------------------------------------------------------- 779
  Csu1 -------------------------------------------------------------------------------- 946
  Ahy1 -------------------------------------------------------------------------------- 403
  Plu1 -------------------------------------------------------------------------------- 1240
  Cla1 -------------------------------------------------------------------------------- 766
  Pas1 -------------------------------------------------------------------------------- 505
 ruler ......4970......4980......4990......5000......5010......5020......5030......5040
```

```
   Eca1 --------------------------------------------------------------------------------   53
   Esp2 --------------------------------------------------------------------------------   53
   Cko1 --------------------------------------------------------------------------------   53
   Sen1 --------------------------------------------------------------------------------   53
   Eco3 --------------------------------------------------------------------------------   49
   Esa3 --------------------------------------------------------------------------------   54
   Kpn1 --------------------------------------------------------------------------------   51
   Pan1 --------------------------------------------------------------------------------   61
   Eta2 --------------------------------------------------------------------------------   52
   Spr1 --------------------------------------------------------------------------------   69
  Eco25 -------------------------------------------------------------------------------- 2170
  Eco15 -------------------------------------------------------------------------------- 1252
   Yfr1 --------------------------------------------------------------------------------  296
   Yfr5 --------------------------------------------------------------------------------  345
   Yps4 FIADINTARIANGDFVIIDDGAVANSVDANEVRARVTDNQGNAIAGYSVTFASQNGATITTSGITGVDGWASAKLTHTKA 4304
   Efe2 --------------------------------------------------------------------------------  683
   Efe3 -------------------------------------------------------------------------------- 1699
   Eta1 --------------------------------------------------------------------------------  881
  Eco26 -------------------------------------------------------------------------------- 1171
   Pru1 -------------------------------------------------------------------------------- 1882
   Sgl1 --------------------------------------------------------------------------------  447
   Pmi1 -------------------------------------------------------------------------------- 1879
   Ymo1 --------------------------------------------------------------------------------  989
   Yen2 -------------------------------------------------------------------------------- 2010
   Sen2 --------------------------------------------------------------------------------  269
   Efe4 --------------------------------------------------------------------------------  263
   Bpe1 --------------------------------------------------------------------------------  836
   Bav2 -------------------------------------------------------------------------------- 1187
   Bpa2 -------------------------------------------------------------------------------- 1474
   Bav1 --------------------------------------------------------------------------------  347
   Yin1 --------------------------------------------------------------------------------  269
   Yfr4 --------------------------------------------------------------------------------  269
   Yin2 --------------------------------------------------------------------------------  270
   Ymo2 --------------------------------------------------------------------------------  289
  Eco10 --------------------------------------------------------------------------------  291
   Eal1 --------------------------------------------------------------------------------  290
  Eco16 --------------------------------------------------------------------------------  295
   Sty4 --------------------------------------------------------------------------------  290
   Yfr2 --------------------------------------------------------------------------------  443
   Ype5 --------------------------------------------------------------------------------  628
   Eco6 -------------------------------------------------------------------------------- 2342
   Bbr1 --------------------------------------------------------------------------------  519
   Yps2 --------------------------------------------------------------------------------  529
  Eco14 --------------------------------------------------------------------------------  691
   Sen3 -------------------------------------------------------------------------------- 1176
   Pal3 --------------------------------------------------------------------------------   86
   Ybe1 --------------------------------------------------------------------------------  505
   Pal2 -------------------------------------------------------------------------------- 2107
   Eco1 --------------------------------------------------------------------------------  420
   Yps7 --------------------------------------------------------------------------------  989
   Yfr3 --------------------------------------------------------------------------------  425
  Eco20 --------------------------------------------------------------------------------  996
   Efe5 --------------------------------------------------------------------------------  779
   Csu1 --------------------------------------------------------------------------------  946
   Ahy1 --------------------------------------------------------------------------------  403
   Plu1 -------------------------------------------------------------------------------- 1240
   Cla1 --------------------------------------------------------------------------------  766
   Pas1 --------------------------------------------------------------------------------  505
  ruler ......5050......5060......5070......5080......5090......5100......5110......5120
```

```
  Eca1 --------------------------------------------------------------------------------   53
  Esp2 --------------------------------------------------------------------------------   53
  Cko1 --------------------------------------------------------------------------------   53
  Sen1 --------------------------------------------------------------------------------   53
  Eco3 --------------------------------------------------------------------------------   49
  Esa3 --------------------------------------------------------------------------------   54
  Kpn1 --------------------------------------------------------------------------------   51
  Pan1 --------------------------------------------------------------------------------   61
  Eta2 --------------------------------------------------------------------------------   52
  Spr1 --------------------------------------------------------------------------------   69
 Eco25 -------------------------------------------------------------------------------- 2170
 Eco15 -------------------------------------------------------------------------------- 1252
  Yfr1 --------------------------------------------------------------------------------  296
  Yfr5 --------------------------------------------------------------------------------  345
  Yps4 GESGILARISRPGSMVQVLTPYFIADVSTATLQLFNFNPIPIIADGVMQFFVLGRVFDANQNPVGGQQVAFSATNEVTLT 4384
  Efe2 --------------------------------------------------------------------------------  683
  Efe3 -------------------------------------------------------------------------------- 1699
  Eta1 --------------------------------------------------------------------------------  881
 Eco26 -------------------------------------------------------------------------------- 1171
  Pru1 -------------------------------------------------------------------------------- 1882
  Sgl1 --------------------------------------------------------------------------------  447
  Pmi1 -------------------------------------------------------------------------------- 1879
  Ymo1 --------------------------------------------------------------------------------  989
  Yen2 -------------------------------------------------------------------------------- 2010
  Sen2 --------------------------------------------------------------------------------  269
  Efe4 --------------------------------------------------------------------------------  263
  Bpe1 --------------------------------------------------------------------------------  836
  Bav2 -------------------------------------------------------------------------------- 1187
  Bpa2 -------------------------------------------------------------------------------- 1474
  Bav1 --------------------------------------------------------------------------------  347
  Yin1 --------------------------------------------------------------------------------  269
  Yfr4 --------------------------------------------------------------------------------  269
  Yin2 --------------------------------------------------------------------------------  270
  Ymo2 --------------------------------------------------------------------------------  289
 Eco10 --------------------------------------------------------------------------------  291
  Eal1 --------------------------------------------------------------------------------  290
 Eco16 --------------------------------------------------------------------------------  295
  Sty4 --------------------------------------------------------------------------------  290
  Yfr2 --------------------------------------------------------------------------------  443
  Ype5 --------------------------------------------------------------------------------  628
  Eco6 -------------------------------------------------------------------------------- 2342
  Bbr1 --------------------------------------------------------------------------------  519
  Yps2 --------------------------------------------------------------------------------  529
 Eco14 --------------------------------------------------------------------------------  691
  Sen3 -------------------------------------------------------------------------------- 1176
  Pal3 --------------------------------------------------------------------------------   86
  Ybe1 --------------------------------------------------------------------------------  505
  Pal2 -------------------------------------------------------------------------------- 2107
  Eco1 --------------------------------------------------------------------------------  420
  Yps7 --------------------------------------------------------------------------------  989
  Yfr3 --------------------------------------------------------------------------------  425
 Eco20 --------------------------------------------------------------------------------  996
  Efe5 --------------------------------------------------------------------------------  779
  Csu1 --------------------------------------------------------------------------------  946
  Ahy1 --------------------------------------------------------------------------------  403
  Plu1 -------------------------------------------------------------------------------- 1240
  Cla1 --------------------------------------------------------------------------------  766
  Pas1 --------------------------------------------------------------------------------  505
 ruler ......5130......5140......5150......5160......5170......5180......5190......5200
```

```
  Eca1 --------------------------------------------------------------------------------   53
  Esp2 --------------------------------------------------------------------------------   53
  Cko1 --------------------------------------------------------------------------------   53
  Sen1 --------------------------------------------------------------------------------   53
  Eco3 --------------------------------------------------------------------------------   49
  Esa3 --------------------------------------------------------------------------------   54
  Kpn1 --------------------------------------------------------------------------------   51
  Pan1 --------------------------------------------------------------------------------   61
  Eta2 --------------------------------------------------------------------------------   52
  Spr1 --------------------------------------------------------------------------------   69
 Eco25 --------------------------------------------------------------------------------  2170
 Eco15 --------------------------------------------------------------------------------  1252
  Yfr1 --------------------------------------------------------------------------------  296
  Yfr5 --------------------------------------------------------------------------------  345
  Yps4 ESNGSISTPEGSVLLSVTSTQAGVHPITGTLVSNNYTDTFGATFIANKNTAQLSTLMVVDNNALADGVTRNQVRAHVVDS  4464
  Efe2 --------------------------------------------------------------------------------  683
  Efe3 --------------------------------------------------------------------------------  1699
  Eta1 --------------------------------------------------------------------------------  881
 Eco26 --------------------------------------------------------------------------------  1171
  Pru1 --------------------------------------------------------------------------------  1882
  Sgl1 --------------------------------------------------------------------------------  447
  Pmi1 --------------------------------------------------------------------------------  1879
  Ymo1 --------------------------------------------------------------------------------  989
  Yen2 --------------------------------------------------------------------------------  2010
  Sen2 --------------------------------------------------------------------------------  269
  Efe4 --------------------------------------------------------------------------------  263
  Bpe1 --------------------------------------------------------------------------------  836
  Bav2 --------------------------------------------------------------------------------  1187
  Bpa2 --------------------------------------------------------------------------------  1474
  Bav1 --------------------------------------------------------------------------------  347
  Yin1 --------------------------------------------------------------------------------  269
  Yfr4 --------------------------------------------------------------------------------  269
  Yin2 --------------------------------------------------------------------------------  270
  Ymo2 --------------------------------------------------------------------------------  289
 Eco10 --------------------------------------------------------------------------------  291
  Eal1 --------------------------------------------------------------------------------  290
 Eco16 --------------------------------------------------------------------------------  295
  Sty4 --------------------------------------------------------------------------------  290
  Yfr2 --------------------------------------------------------------------------------  443
  Ype5 --------------------------------------------------------------------------------  628
  Eco6 --------------------------------------------------------------------------------  2342
  Bbr1 --------------------------------------------------------------------------------  519
  Yps2 --------------------------------------------------------------------------------  529
 Eco14 --------------------------------------------------------------------------------  691
  Sen3 --------------------------------------------------------------------------------  1176
  Pal3 --------------------------------------------------------------------------------   86
  Ybe1 --------------------------------------------------------------------------------  505
  Pal2 --------------------------------------------------------------------------------  2107
  Eco1 --------------------------------------------------------------------------------  420
  Yps7 --------------------------------------------------------------------------------  989
  Yfr3 --------------------------------------------------------------------------------  425
 Eco20 --------------------------------------------------------------------------------  996
  Efe5 --------------------------------------------------------------------------------  779
  Csu1 --------------------------------------------------------------------------------  946
  Ahy1 --------------------------------------------------------------------------------  403
  Plu1 --------------------------------------------------------------------------------  1240
  Cla1 --------------------------------------------------------------------------------  766
  Pas1 --------------------------------------------------------------------------------  505
 ruler ......5210......5220......5230......5240......5250......5260......5270......5280
```

```
Eca1   --------------------------------------------------------------------------------   53
Esp2   --------------------------------------------------------------------------------   53
Cko1   --------------------------------------------------------------------------------   53
Sen1   --------------------------------------------------------------------------------   53
Eco3   --------------------------------------------------------------------------------   49
Esa3   --------------------------------------------------------------------------------   54
Kpn1   --------------------------------------------------------------------------------   51
Pan1   --------------------------------------------------------------------------------   61
Eta2   --------------------------------------------------------------------------------   52
Spr1   --------------------------------------------------------------------------------   69
Eco25  --------------------------------------------------------------------------------   2170
Eco15  --------------------------------------------------------------------------------   1252
Yfr1   --------------------------------------------------------------------------------   296
Yfr5   --------------------------------------------------------------------------------   345
Yps4   TGNSVADIAVTFTANHGAQLSHVTVLTDDNGDAVNTLTNSLVGVTVVTAKLGTAGTPLTVDTVFTAGPLATLTLVTMVDN   4544
Efe2   --------------------------------------------------------------------------------   683
Efe3   --------------------------------------------------------------------------------   1699
Eta1   --------------------------------------------------------------------------------   881
Eco26  --------------------------------------------------------------------------------   1171
Pru1   --------------------------------------------------------------------------------   1882
Sgl1   --------------------------------------------------------------------------------   447
Pmi1   --------------------------------------------------------------------------------   1879
Ymo1   --------------------------------------------------------------------------------   989
Yen2   --------------------------------------------------------------------------------   2010
Sen2   --------------------------------------------------------------------------------   269
Efe4   --------------------------------------------------------------------------------   263
Bpe1   --------------------------------------------------------------------------------   836
Bav2   --------------------------------------------------------------------------------   1187
Bpa2   --------------------------------------------------------------------------------   1474
Bav1   --------------------------------------------------------------------------------   347
Yin1   --------------------------------------------------------------------------------   269
Yfr4   --------------------------------------------------------------------------------   269
Yin2   --------------------------------------------------------------------------------   270
Ymo2   --------------------------------------------------------------------------------   289
Eco10  --------------------------------------------------------------------------------   291
Eal1   --------------------------------------------------------------------------------   290
Eco16  --------------------------------------------------------------------------------   295
Sty4   --------------------------------------------------------------------------------   290
Yfr2   --------------------------------------------------------------------------------   443
Ype5   --------------------------------------------------------------------------------   628
Eco6   --------------------------------------------------------------------------------   2342
Bbr1   --------------------------------------------------------------------------------   519
Yps2   --------------------------------------------------------------------------------   529
Eco14  --------------------------------------------------------------------------------   691
Sen3   --------------------------------------------------------------------------------   1176
Pal3   --------------------------------------------------------------------------------   86
Ybe1   --------------------------------------------------------------------------------   505
Pal2   --------------------------------------------------------------------------------   2107
Eco1   --------------------------------------------------------------------------------   420
Yps7   --------------------------------------------------------------------------------   989
Yfr3   --------------------------------------------------------------------------------   425
Eco20  --------------------------------------------------------------------------------   996
Efe5   --------------------------------------------------------------------------------   779
Csu1   --------------------------------------------------------------------------------   946
Ahy1   --------------------------------------------------------------------------------   403
Plu1   --------------------------------------------------------------------------------   1240
Cla1   --------------------------------------------------------------------------------   766
Pas1   --------------------------------------------------------------------------------   505
ruler  ......5290......5300......5310......5320......5330......5340......5350......5360
```

```
  Eca1 --------------------------------------------------------------------------------   53
  Esp2 --------------------------------------------------------------------------------   53
  Cko1 --------------------------------------------------------------------------------   53
  Sen1 --------------------------------------------------------------------------------   53
  Eco3 --------------------------------------------------------------------------------   49
  Esa3 --------------------------------------------------------------------------------   54
  Kpn1 --------------------------------------------------------------------------------   51
  Pan1 --------------------------------------------------------------------------------   61
  Eta2 --------------------------------------------------------------------------------   52
  Spr1 --------------------------------------------------------------------------------   69
 Eco25 -------------------------------------------------------------------------------- 2170
 Eco15 -------------------------------------------------------------------------------- 1252
  Yfr1 --------------------------------------------------------------------------------  296
  Yfr5 --------------------------------------------------------------------------------  345
  Yps4 AFADNSATNTVQATLKDATGNPIVGEVVAFAASNGATITATDGGVSNANGIVLATLTNGAAGVSTVTATIETLTATTETT 4624
  Efe2 --------------------------------------------------------------------------------  683
  Efe3 -------------------------------------------------------------------------------- 1699
  Eta1 --------------------------------------------------------------------------------  881
 Eco26 -------------------------------------------------------------------------------- 1171
  Pru1 -------------------------------------------------------------------------------- 1882
  Sgl1 --------------------------------------------------------------------------------  447
  Pmi1 -------------------------------------------------------------------------------- 1879
  Ymo1 --------------------------------------------------------------------------------  989
  Yen2 -------------------------------------------------------------------------------- 2010
  Sen2 --------------------------------------------------------------------------------  269
  Efe4 --------------------------------------------------------------------------------  263
  Bpe1 --------------------------------------------------------------------------------  836
  Bav2 -------------------------------------------------------------------------------- 1187
  Bpa2 -------------------------------------------------------------------------------- 1474
  Bav1 --------------------------------------------------------------------------------  347
  Yin1 --------------------------------------------------------------------------------  269
  Yfr4 --------------------------------------------------------------------------------  269
  Yin2 --------------------------------------------------------------------------------  270
  Ymo2 --------------------------------------------------------------------------------  289
 Eco10 --------------------------------------------------------------------------------  291
  Eal1 --------------------------------------------------------------------------------  290
 Eco16 --------------------------------------------------------------------------------  295
  Sty4 --------------------------------------------------------------------------------  290
  Yfr2 --------------------------------------------------------------------------------  443
  Ype5 --------------------------------------------------------------------------------  628
  Eco6 -------------------------------------------------------------------------------- 2342
  Bbr1 --------------------------------------------------------------------------------  519
  Yps2 --------------------------------------------------------------------------------  529
 Eco14 --------------------------------------------------------------------------------  691
  Sen3 -------------------------------------------------------------------------------- 1176
  Pal3 --------------------------------------------------------------------------------   86
  Ybe1 --------------------------------------------------------------------------------  505
  Pal2 -------------------------------------------------------------------------------- 2107
  Eco1 --------------------------------------------------------------------------------  420
  Yps7 --------------------------------------------------------------------------------  989
  Yfr3 --------------------------------------------------------------------------------  425
 Eco20 --------------------------------------------------------------------------------  996
  Efe5 --------------------------------------------------------------------------------  779
  Csu1 --------------------------------------------------------------------------------  946
  Ahy1 --------------------------------------------------------------------------------  403
  Plu1 -------------------------------------------------------------------------------- 1240
  Cla1 --------------------------------------------------------------------------------  766
  Pas1 --------------------------------------------------------------------------------  505
 ruler ......5370......5380......5390......5400......5410......5420......5430......5440
```

```
Eca1   --------------------------------------------------------------------------------   53
Esp2   --------------------------------------------------------------------------------   53
Cko1   --------------------------------------------------------------------------------   53
Sen1   --------------------------------------------------------------------------------   53
Eco3   --------------------------------------------------------------------------------   49
Esa3   --------------------------------------------------------------------------------   54
Kpn1   --------------------------------------------------------------------------------   51
Pan1   --------------------------------------------------------------------------------   61
Eta2   --------------------------------------------------------------------------------   52
Spr1   --------------------------------------------------------------------------------   69
Eco25  --------------------------------------------------------------------------------   2170
Eco15  --------------------------------------------------------------------------------   1252
Yfr1   --------------------------------------------------------------------------------   296
Yfr5   --------------------------------------------------------------------------------   345
Yps4   FIAMKNLDVTVGDTTFDGDAGFPTTGFVGAAFKVNSGGDNSLYDWSSSAPALVSVSGEGVVTFNAVFPTGTPAITISATP   4704
Efe2   --------------------------------------------------------------------------------   683
Efe3   --------------------------------------------------------------------------------   1699
Eta1   --------------------------------------------------------------------------------   881
Eco26  --------------------------------------------------------------------------------   1171
Pru1   --------------------------------------------------------------------------------   1882
Sgl1   --------------------------------------------------------------------------------   447
Pmi1   --------------------------------------------------------------------------------   1879
Ymo1   --------------------------------------------------------------------------------   989
Yen2   --------------------------------------------------------------------------------   2010
Sen2   --------------------------------------------------------------------------------   269
Efe4   --------------------------------------------------------------------------------   263
Bpe1   --------------------------------------------------------------------------------   836
Bav2   --------------------------------------------------------------------------------   1187
Bpa2   --------------------------------------------------------------------------------   1474
Bav1   --------------------------------------------------------------------------------   347
Yin1   --------------------------------------------------------------------------------   269
Yfr4   --------------------------------------------------------------------------------   269
Yin2   --------------------------------------------------------------------------------   270
Ymo2   --------------------------------------------------------------------------------   289
Eco10  --------------------------------------------------------------------------------   291
Eal1   --------------------------------------------------------------------------------   290
Eco16  --------------------------------------------------------------------------------   295
Sty4   --------------------------------------------------------------------------------   290
Yfr2   --------------------------------------------------------------------------------   443
Ype5   --------------------------------------------------------------------------------   628
Eco6   --------------------------------------------------------------------------------   2342
Bbr1   --------------------------------------------------------------------------------   519
Yps2   --------------------------------------------------------------------------------   529
Eco14  --------------------------------------------------------------------------------   691
Sen3   --------------------------------------------------------------------------------   1176
Pal3   --------------------------------------------------------------------------------   86
Ybe1   --------------------------------------------------------------------------------   505
Pal2   --------------------------------------------------------------------------------   2107
Eco1   --------------------------------------------------------------------------------   420
Yps7   --------------------------------------------------------------------------------   989
Yfr3   --------------------------------------------------------------------------------   425
Eco20  --------------------------------------------------------------------------------   996
Efe5   --------------------------------------------------------------------------------   779
Csu1   --------------------------------------------------------------------------------   946
Ahy1   --------------------------------------------------------------------------------   403
Plu1   --------------------------------------------------------------------------------   1240
Cla1   --------------------------------------------------------------------------------   766
Pas1   --------------------------------------------------------------------------------   505
ruler  ......5450......5460......5470......5480......5490......5500......5510......5520
```

```
   Eca1 --------------------------------------------------------------------------------   53
   Esp2 --------------------------------------------------------------------------------   53
   Cko1 --------------------------------------------------------------------------------   53
   Sen1 --------------------------------------------------------------------------------   53
   Eco3 --------------------------------------------------------------------------------   49
   Esa3 --------------------------------------------------------------------------------   54
   Kpn1 --------------------------------------------------------------------------------   51
   Pan1 --------------------------------------------------------------------------------   61
   Eta2 --------------------------------------------------------------------------------   52
   Spr1 --------------------------------------------------------------------------------   69
  Eco25 -------------------------------------------------------------------------------- 2170
  Eco15 -------------------------------------------------------------------------------- 1252
   Yfr1 --------------------------------------------------------------------------------  296
   Yfr5 --------------------------------------------------------------------------------  345
   Yps4 KGGGSPLSYSFRVNQWFINNNGVALNRADAATYCANAGYTTVSSSQVTNAIVWGMGTRAMGNLWSEWGDFNNYNVPGWEP 4784
   Efe2 --------------------------------------------------------------------------------  683
   Efe3 -------------------------------------------------------------------------------- 1699
   Eta1 --------------------------------------------------------------------------------  881
  Eco26 -------------------------------------------------------------------------------- 1171
   Pru1 -------------------------------------------------------------------------------- 1882
   Sgl1 --------------------------------------------------------------------------------  447
   Pmi1 -------------------------------------------------------------------------------- 1879
   Ymo1 --------------------------------------------------------------------------------  989
   Yen2 -------------------------------------------------------------------------------- 2010
   Sen2 --------------------------------------------------------------------------------  269
   Efe4 --------------------------------------------------------------------------------  263
   Bpe1 --------------------------------------------------------------------------------  836
   Bav2 -------------------------------------------------------------------------------- 1187
   Bpa2 -------------------------------------------------------------------------------- 1474
   Bav1 --------------------------------------------------------------------------------  347
   Yin1 --------------------------------------------------------------------------------  269
   Yfr4 --------------------------------------------------------------------------------  269
   Yin2 --------------------------------------------------------------------------------  270
   Ymo2 --------------------------------------------------------------------------------  289
  Eco10 --------------------------------------------------------------------------------  291
   Eal1 --------------------------------------------------------------------------------  290
  Eco16 --------------------------------------------------------------------------------  295
   Sty4 --------------------------------------------------------------------------------  290
   Yfr2 --------------------------------------------------------------------------------  443
   Ype5 --------------------------------------------------------------------------------  628
   Eco6 -------------------------------------------------------------------------------- 2342
   Bbr1 --------------------------------------------------------------------------------  519
   Yps2 --------------------------------------------------------------------------------  529
  Eco14 --------------------------------------------------------------------------------  691
   Sen3 -------------------------------------------------------------------------------- 1176
   Pal3 --------------------------------------------------------------------------------   86
   Ybe1 --------------------------------------------------------------------------------  505
   Pal2 -------------------------------------------------------------------------------- 2107
   Eco1 --------------------------------------------------------------------------------  420
   Yps7 --------------------------------------------------------------------------------  989
   Yfr3 --------------------------------------------------------------------------------  425
  Eco20 --------------------------------------------------------------------------------  996
   Efe5 --------------------------------------------------------------------------------  779
   Csu1 --------------------------------------------------------------------------------  946
   Ahy1 --------------------------------------------------------------------------------  403
   Plu1 -------------------------------------------------------------------------------- 1240
   Cla1 --------------------------------------------------------------------------------  766
   Pas1 --------------------------------------------------------------------------------  505
  ruler ......5530......5540......5550......5560......5570......5580......5590......5600
```

```
  Eca1 -------------------------------------------    53
  Esp2 -------------------------------------------    53
  Cko1 -------------------------------------------    53
  Sen1 -------------------------------------------    53
  Eco3 -------------------------------------------    49
  Esa3 -------------------------------------------    54
  Kpn1 -------------------------------------------    51
  Pan1 -------------------------------------------    61
  Eta2 -------------------------------------------    52
  Spr1 -------------------------------------------    69
 Eco25 -------------------------------------------  2170
 Eco15 -------------------------------------------  1252
  Yfr1 -------------------------------------------   296
  Yfr5 -------------------------------------------   345
  Yps4 AEFFWLSDNYNATDGLAASLSHGVLTTMGDPMAMIHVMCTRPI  4827
  Efe2 -------------------------------------------   683
  Efe3 -------------------------------------------  1699
  Eta1 -------------------------------------------   881
 Eco26 -------------------------------------------  1171
  Pru1 -------------------------------------------  1882
  Sgl1 -------------------------------------------   447
  Pmi1 -------------------------------------------  1879
  Ymo1 -------------------------------------------   989
  Yen2 -------------------------------------------  2010
  Sen2 -------------------------------------------   269
  Efe4 -------------------------------------------   263
  Bpe1 -------------------------------------------   836
  Bav2 -------------------------------------------  1187
  Bpa2 -------------------------------------------  1474
  Bav1 -------------------------------------------   347
  Yin1 -------------------------------------------   269
  Yfr4 -------------------------------------------   269
  Yin2 -------------------------------------------   270
  Ymo2 -------------------------------------------   289
 Eco10 -------------------------------------------   291
  Eal1 -------------------------------------------   290
 Eco16 -------------------------------------------   295
  Sty4 -------------------------------------------   290
  Yfr2 -------------------------------------------   443
  Ype5 -------------------------------------------   628
  Eco6 -------------------------------------------  2342
  Bbr1 -------------------------------------------   519
  Yps2 -------------------------------------------   529
 Eco14 -------------------------------------------   691
  Sen3 -------------------------------------------  1176
  Pal3 -------------------------------------------    86
  Ybe1 -------------------------------------------   505
  Pal2 -------------------------------------------  2107
  Eco1 -------------------------------------------   420
  Yps7 -------------------------------------------   989
  Yfr3 -------------------------------------------   425
 Eco20 -------------------------------------------   996
  Efe5 -------------------------------------------   779
  Csu1 -------------------------------------------   946
  Ahy1 -------------------------------------------   403
  Plu1 -------------------------------------------  1240
  Cla1 -------------------------------------------   766
  Pas1 -------------------------------------------   505
 ruler ......5610......5620......5630......5640...
```
