# Supplementary figures and images for: The Bacterial Intimins and Invasins: A Large and Novel Family of Secreted Proteins
Source: PLoS One. 2010 Dec 22;5(12):e14403. doi: 10.1371/journal.pone.0014403 (PMC3008723; doi:10.1371/journal.pone.0014403)

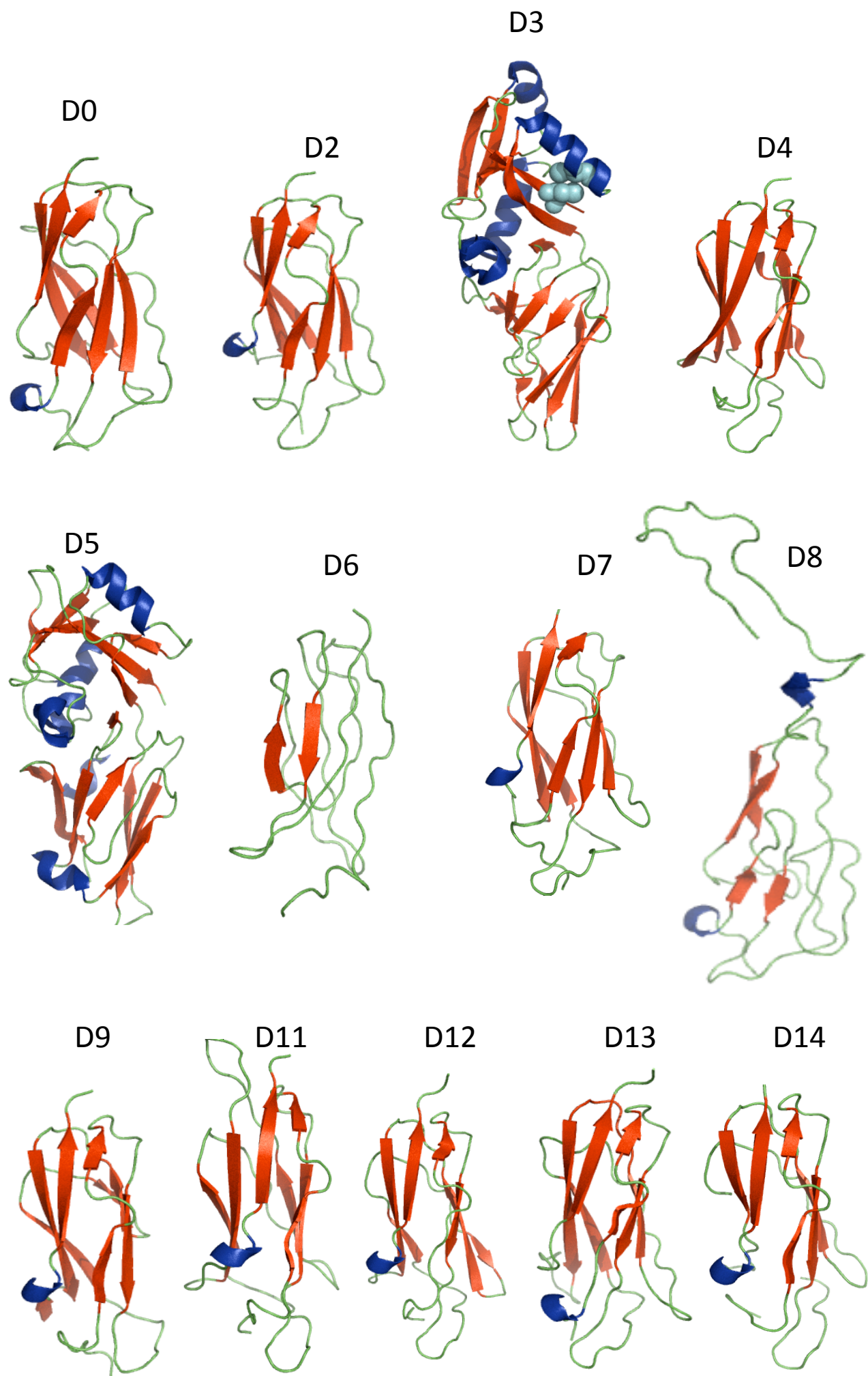

Supplement: Figure S5 — Tertiary structural predictions of the Big subdomains comprising the C-terminal passenger domains. (0.78 MB PDF) [file pone.0014403.s005.pdf]
