# Supplementary material for: The Bacterial Intimins and Invasins: A Large and Novel Family of Secreted Proteins
Source: PLoS One. 2010 Dec 22;5(12):e14403. doi: 10.1371/journal.pone.0014403 (PMC3008723; doi:10.1371/journal.pone.0014403)
Supplement: Figure S6 — Multiple alignment of passenger subdomain D0. (0.02 MB PDF) [file pone.0014403.s006.pdf]

```
Esa2            -------VADMTLATIKNNSPADGTSADTVTLHAVTAD-GKPAAHAAIVWTVSGNAA---
Yps4            -------MVISHLATTVDNATANGIAANTVQATVTDGD-GQPIIGQIINFAVNTQAT---
Yps2            -------QLTLTAAVIGDGAPANGKTAITVEFTVADFE-GKPLAGQEVVITTNNGAL---
Ype5            ---HGVNTNASGLTAAPEILPANASASSVIEFNIKDNA-NQPITGIADELAFSLELVELP
Yps7            ---QKINIANSTLVAAPVNIEANNIDTSVVTLTLKDDN-NIPVP----------------
Yfr3            ---QSVSHLNSTLQVSPTEISADGVAVSLITLNLKDDN-NLPVKGMGDQLTLDLHFTP--
Yfr2            ---QNLSTGNSLMTATPSILVADGVSTSQITITLRDSH-NQPVIGASRNIILASNFVVDG
Eco26           ----DIDPTISSLDPKNISLPADGQSQKKLTLKIKNRN-GLFEDINPDDINVLKTSEENF
Eta1            ---AAIDASMSPVTPEKITLPADGKTQQLLLLKINDRD-GKPVDIAESEISVLREAKLRT
Pru1            ---PAIYPIKSALTPRKIDLIADGKNTKKLSLSIRDKA-GNYIDLAANEIGIEKVVANKT
Pmi1            ---SLINTRNSLFSPKQSQLFANGEATQKLILSIVDND-NLPVDIDSKEITLQQQSDTEK
Sgl1            ---PAINTTTSEFTPVKSTLPADTVAQQTLTLKVKDIQ-GNPVDIGEDEITVTSNNAQEN
Yen2            ---VNINRINSTISLNPATLPANGTSRSTIQLKLNTDA-GQAVSGASGQMTFAIRDSSGR
Ymo1            ---VNINSVKSTITLTPATLPANATSRSTLQLKLNTDA-GNAVSGAARQITFAVRDVSGN
Ybe1            ---DTPATISGTWTPAASTKPADGNSAVLLTLTLKDAA-GNPLIGQANNITLKKNTLGGS
Eco20           ---SADRTALTLDGQSRIQMLANGNEQRPLVLSLRDAE-GQPVTGMKDQIKTELAFKPAG
Efe5            SNVSAGDSSFTLDGNNNAQISADGQSTYPVTLNLKDSN-GKPLTGLENDIEMSLEFTPDN
Eco15           ---PTLSQKDSLLSVNPLTVAADKKSTTTLTVTAHDSD-GTPVPGLALQTRSEGVQD---
Eco25           ---PTLSQKDSSVSLSSQTLSADSHSTATLTFIAHDAA-GNPVIGLVLSTRHEGVQD---
Eco6            ---PTLSLTDSTLSVDQQILLADGKSTSTLTYTARDSS-GKPIPGMTLKTQVKGLQD---
Efe2            ---PQLSAEDSEVTSDKPILKPDGVDTAHLTFLARDTD-GKAVSGLKVTAGFTAPEGLNF
Efe3            --------QHSQVTSDKTTLKPDGTEKAVLTFRAQDAE-GKAVSALTVSTSFTAPQGMAL
Yfr1            -------QISLTLSGGSNTAVADGNAPVSYIATVVDTSGTVATPLAGMNIAFDSTVG---
Yfr5            ---------ALNVVGSSTSATADGSALVTYRASVVDTANGANTPMAGMNVAFNATIG---
Eco14           -------QQQLAVSTDKTTATADGADSVRYTLTVTGSD-GKPVSGQAVRWEHNGG-----
Csu1            ---------SGTINILKNNQPANNQDQAEVIFTIVDAH-NNPIPNFAVTATASNQAT---
Eco1            -------VGVTDFTADKTSAKADGTEAITYTATVKKNG--VAQANVPVSFNIVSGTAT--
Sen3            ---FDLHQITSATTTTSATLPADGVSTTQVTVTVTSGN-GVKITGLANDLSAQLMRSNSS
Plu1            -------RNISLSVNTTDPLIADGNAKYVYTATLLGADKKTPIENAKLIWDTDKKDPGLK
Pal2            ------SISPRDMKFHGKG-LANGNDINGLTAIARDSL-GHAIPNTKVVFVLPNALT---
                                       .:

Esa2            -----------------LSSTNSVTDANGNTSVNLT-NTTAGQVIVTATSGS--VVRTTS
Yps4            -----------------LSTTEARTGANGIASTTLT-HTVAGVSAVSATLGS--SSRSVN
Yps2            -----------------PNKITEKTDANGVARIALT-NTTDGVTVVTAEVEG--QRQSVD
Ype5            EELAKAKARSVPLKTVSHTLTKITESAPGIYQATLTSGSKPQLINITAQING-VPLADVQ
Yps7            -------GQNVTFLSPLGTLSAMTDSGNGVYTATLTAGTVSGTTAVSSNING-IALDMTP
Yfr3            -------DTTATQPVSYPLLSTVTETAPGIYTYQLTAGFTQGEANIAPTIQN-ISLASAK
Yfr2            -----STSPIRAAAASEMKISDVSEAMGGVYNATLTAGTQPGTATITSSING-MALNAVN
Eco26           ----DVS----------SKITRFSRQEAGIYTATLTAGTKSERFTITPMIYN-IKLPPST
Eta1            ----AGS----------TTITAFSRSAAGEYVATLTAGTLPESFTLIPTARN-VRFAPIS
Pru1            ----AARSVNSANTANLTTVSGFTRIAAGQYEAILTSGTTPENFVLVSKARN-AVFPEIK
Pmi1            ----GNS-----------RISTFSRLAAGKYQLTVTAGSIPEKLTLTPVFRD-NTFNSAT
Sgl1            ----SGA-----------KVSALQRQDSGIYTLVVTAGTGTDVIKITPSARG-ANFASAS
Yen2            ----VFKAR---TSLQPVVISDVQEVQTGVYEASITSGFLTGRFEITPTVRG-VQLNPII
Ymo1            ----VPRAR---SFAQPVVIADAQEIQTGVYETSVTSGNLVGRFEITPTVRG-VQLNPII
Ybe1            --------------GSEPTLSALTETGPGIYQTTATAGTNFGVLTLTPEIQG-TQLAPAT
Eco20           N--IVTRSLKATKSQAKPTLGEFTETEAGVYQSVFTTGTQSGEATITVSVDGMSKTVTAE
Efe5            N--TQR-----SRSVSGPQLGKVQEISAGVYRSMLTAGSQSGTARVTAKVLG--KTFTLN
Eco15           -----------------ITLSDWTDNGDGSYTQILTAGTTSGSVTLTPQINGESAVKESI
Eco25           -----------------ITLSDWKDNGDGSYTQILTTGAMSGTLTLMPQLNGVDAAKAPA
Eco6            -----------------FALSEWKDNGNGTYTQIVTAGKTSGALSLMPQFNGDDIAKTPA
Efe2            S----------------LSEFFTETKTPGEYTAGLK-GSMKGTVSVMPLVDGKPATKAPV
Efe3            V----------------LSDTFTETETKGTYTAELK-GTMPGEVRVMPQVAGKDAAKDAV
Yfr1            -----------------NVLTPNAVTDNQGKANITIKSTLAGGGHIHGVLDN-GNRAQAP
Yfr5            -----------------DVVTPVGTTDNMGRATVSVKSTLAGAGQVHAVLDN-GNRAQAA
Eco14           -----------------TLNGENTTNADGVATATLT-SQTAGIIRVTATTRN-QTAKAAD
Csu1            -----------------LDTVPSTTDASGQIRVSLK-NNRSGITEVTATSNN--SSSTAK
```

```
Eco1            -----------------LGANSAKTDANGKATVTLK-SSTPGQVVVSAKTAEMTSALNAS
Sen3            K--------GLAADTVQEKISAFKEQSPGVYVSTFTSGTLAGVVTVQPYYNQTSKLSSTT
Plu1            -----------------LKPDSEATDKNGQQTATLTSTTPLSDIQVSVRING----ERVT
Pal2            -----------------LASRKTKTTTNSSVQNSLR--KKMQTMKVAKPWEYITTTNDKG
                                                             :

Esa2            AAFNLLV---
Yps4            TTFVADE---
Yps2            THFVKGT---
Ype5            TKVTLIA---
Yps7            ATVTLNGNS-
Yfr3            VILTASD---
Yfr2            VIFTANA---
Eco26           VTISKDR---
Eta1            VELTAND---
Pru1            VSVLADG---
Pmi1            VTLIADN---
Sgl1            VTVEADG---
Yen2            LTQSADK---
Ymo1            LTQSADA---
Ybe1            ITYTDPS---
Eco20           LRATMM----
Efe5            IKQTVAEE--
Eco15           VVNIVPVV--
Eco25           VVNIISVSSS
Eco6            LIAIVA----
Efe2            TVTLSDVV--
Efe3            TVTLVNTTP-
Yfr1            LIFIAD----
Yfr5            VMFMA-----
Eco14           VTFV------
Csu1            LEFLPV----
Eco1            AVIFV-----
Sen3            ITLTPV----
Plu1            ----------
Pal2            EALVQF----
```
