# Supplementary material for: The Bacterial Intimins and Invasins: A Large and Novel Family of Secreted Proteins
Source: PLoS One. 2010 Dec 22;5(12):e14403. doi: 10.1371/journal.pone.0014403 (PMC3008723; doi:10.1371/journal.pone.0014403)
Supplement: Figure S7 — Multiple alignment of passenger subdomain D4. (0.01 MB PDF) [file pone.0014403.s007.pdf]

```
Eco15           KEMSGHVTANNHTFS-TAKFPSEGFAGAYYTLNNDNFEAGKTVDDYMFSSSQ-GWVSVDA
Eco25           KIMSGTVLVNGANLP-TTTFPSQGFTGAYYQLNNDNFAPGKTAADYEFSSSA-SWVDVDA
Yfr2            -PVFTGIIAGGYDFALNDGFPKTGFNQATFTIN-----MNG-TADYSWSSNQPAAVTVNS
Yps4            -MKNLDVTVGDTTFDGDAGFPTTGFVGAAFKVN-----SGGDNSLYDWSSSAPALVSVSG
Ype5            -ATLNTPANTYSGFNINSGFPTTGFKNTHFQLSPHG--ITGANSDYDWVSSH-PNVSVSN
Yps2            -PTLTGILVNGQNFATDKGFPKTIFKNATFQLQMDN--DVANNTQYEWSSSFTPNVSVND
Eco6            -VEVELLSVNGVKFRATDGFPETGFDGAKFTLLLT---HNMKNTDYNWTAGI-YGINVDS
                             :     **   *  : : :             * : :.    : *.

Eco15           SGKVSFANI--GDQTSVTISAVPRQ-GGTTYQTLIKLK
Eco25           TGKVTFKNV--GSKWER-ITATPKT-GGPSYIYEIRVK
Yfr2            SGQVTFNGP-PSGT--VTITATPNN-GGSPQSYSFTVE
Yps4            EGVVTFNAVFPTGTPAITISATPKG-GGSPLSYSFRV-
Ype5            TGAITLQDN-PGGK--VTITATWKHDSSKVFTYDFTL-
Yps2            QGQVTITYQ-TYSE--VAVTAKSKKFPSYSVSYRFYP-
Eco6            NGEVTLSVL---IRSEVTITGKPKNGKGNDVVFKFKI-
                 * :::            ::.  .   .      :
```
