# Supplementary material for: The Bacterial Intimins and Invasins: A Large and Novel Family of Secreted Proteins
Source: PLoS One. 2010 Dec 22;5(12):e14403. doi: 10.1371/journal.pone.0014403 (PMC3008723; doi:10.1371/journal.pone.0014403)
Supplement: Figure S8 — Multiple alignment of passenger subdomain D5. (0.01 MB PDF) [file pone.0014403.s008.pdf]

```
Eco15           --GWWVNNGNHTN-IWLAANALCHAKNDGYNLPGITHLTS----GENKRTQGSLYGEWGN
Yfr2            --ENWFEGDSTSR-NWGNANADCNSRSE--VLPTIAQLTS----GEYIRQIGSLFGEWGD
Eco25           --SWWVNAGDAFM-IYSLAENFCS--SNGYTLPLGDHLNH----SRS-RGIGSLYSEWGD
Yps4            VNQWFINNNGVAL-NRADAATYCA--NAGYTTVSSSQVTNAIVWGMGTRAMGNLWSEWGD
Eco6            -KKWFTSLGATSSNTWDIINTSCSYGQMPSSLELAQRPSG----GVVPRKVGTLWGEYGN
Yps2            -PNRWIYDGGRSLVSSLEASRQCQGSDMSAVLESSRATNG------TRAPDGTLWGEWGS
                    :   .             *   .           .            *.*:.*:*.

Eco15           VGAFSSNSQFTPGAYWTSESDDYSRHYYVQMLTGMTGSDAD-SSPQLTACR---
Yfr2            MMDYG----FPAANNWSSEQSGTSNYYYVVNSSRGRSSQAL-YTPGGTSVL---
Eco25           MGHYTTEAGFHSNMYWSSSPANSNEQYVVSLATGDQSVFEK-LGFAYATC----
Yps4            FNNYNVPGWEPAEFFWLSDNYNATDGLAASLSHGVLTTMGDPMAMIHVMCTRPI
Eco6            LKTYG--NAFSGTDYWTSTQLMGVHEKFNPETGISELGTGKSSGLCVEYY----
Yps2            LTAYS--SDWQSGEYWVKKTSTDFETMNMDTGALQPGPAYLAFPLCALSI----
                .  :           * .
```
