# Supplementary material for: The Bacterial Intimins and Invasins: A Large and Novel Family of Secreted Proteins
Source: PLoS One. 2010 Dec 22;5(12):e14403. doi: 10.1371/journal.pone.0014403 (PMC3008723; doi:10.1371/journal.pone.0014403)
Supplement: Figure S9 — Multiple alignment of passenger subdomain D6. (0.01 MB PDF) [file pone.0014403.s009.pdf]

```
Yin1                ATGAAVPLVT-GSACPLDASGNPEPCIRLKNTQVEVRDGVNYYVLELVSTVMGTFSLQAD
Ymo2                -ARTPIELVN-ANQCPLNAAGKVEPCLRIKDDRTEVRDGVNYHILELVSTLAGNFTISSD
Yfr4                -SGTPVAWVAPGSSCPVDALGLPQPCVQMVQVQTEVRSGINHYVAELVSTQLGTFLVQAD
Yin2                YSGAVVPLVT-GPTCPTDEDGFPDACLRVVHTATEVRDGITYYVNSLISNQPGTFIITTD
                     : : :  *  .  ** :  *  :.*::: .  .***.*:.::: .*:*.  *.* : :*

Yin1                FNHYGRSNVQIVTF-SNPGGRDNVARAEIVDSA
Ymo2                MGVYGVSNSQTFVFNSTISSIENLTGGIFLA--
Yfr4                LGVYGVTAPQTVSFTSSGP--TLVTRAEIQDPS
Yin2                LGAYGVTNAKTITFTSASPMETVVARAEIRDPA
                    :. ** :  : . * *       :: . :
```
