# Supplementary material for: The Bacterial Intimins and Invasins: A Large and Novel Family of Secreted Proteins
Source: PLoS One. 2010 Dec 22;5(12):e14403. doi: 10.1371/journal.pone.0014403 (PMC3008723; doi:10.1371/journal.pone.0014403)
Supplement: Figure S10 — Multiple alignment of passenger subdomain D7. (0.01 MB PDF) [file pone.0014403.s010.pdf]

```
Eal1            HSHEASAQADGVDGVVMDLDVTDSFGDDTDGKGNVLPEDNLNPQLYDAQDKKVTLANKPC
Eco10           HSHEASAQADGVDGVVMDLDVTDSFGDNTDRNGDALPEDNLTPQLYDAQDKRVTLTNKPC
Eco16           VVEITVQQDRKIELIVNNIANVPEENNHSHEASAQADGVDGVVMDLDVTDSFGDNTDRNG
Sty4            HSHEASALADGEDGVVMDLLITDSFGDSTDRNGNELVDDAMTPVLYDSNDKKVTLAQTPC
                  . :       : :* ::  . . .: :.  .             *  *.    ::

Eal1            TTEVPCVFIAEKNKEKGTVTLASTLPGTFRWKAKAA
Eco10           STDNPCVFIAKQDKEKGTVTLSSTLPGTYRWKAKAA
Eco16           DALPEDNLTPQLYDAQDKRVTLTNKPCSTDNPCVFI
Sty4            TTETPCVFIASRDKEAGTVTLSSTLPGTFRWKAKED
                 :     : ..  .  .. .  :. * :    .
```
