# Supplementary material for: The Bacterial Intimins and Invasins: A Large and Novel Family of Secreted Proteins
Source: PLoS One. 2010 Dec 22;5(12):e14403. doi: 10.1371/journal.pone.0014403 (PMC3008723; doi:10.1371/journal.pone.0014403)
Supplement: Figure S12 — Multiple alignment of passenger subdomain D9. (0.01 MB PDF) [file pone.0014403.s012.pdf]

```
Eco26           GTINTNLSFDNKTINETYKGRAVLIQQVIGLPESNH--PPVTWATSDPLVAEIDSRTGYI
Ymo1            GNIIPQLSFDNKNEKQTYRKTPFAKQALKGLPQS----VIAHWNSDNSDVAKIDPATGEI
Pmi1            AIPKSEITFEKPIQQEIYKSTVIDALSYKGVPQN----MQVIWSSSDPTVASIDTTSGQI
Eta1            GTTTPELGFATAQHNVTWTKNFSDSQAVSGVPEG----VEQQWSSSDNSVATVN-EVGKV
Yen2            EKADPGINFAVAKRDVKWMDSMSPQNFVLSNSDANQSDIKTIWQTDSGKIATVD-KGGLV
                    . : *     .  :           . .:         * :..  :* ::   * :

Eco26           TMKKAGMVTITASMPGNDKYSPGIASYNLIISKANPQ
Ymo1            KLLKAGVVNISAVTLADNTYAMGTASYQLEVERADPK
Pmi1            SMKKAGTTIITLQTLGNEQYPSAKNSYPLVIEKAPPK
Eta1            TLLKSGQTTITVKTSGNDQYDPAEASYQLKIDKADPQ
Yen2            TLVKPGTTNVTVSFVGDERFKYGEASYELNVAKYKPT
                .: *.* . ::    .:: :  .  ** * : :  *
```
