# Supplementary material for: The Bacterial Intimins and Invasins: A Large and Novel Family of Secreted Proteins
Source: PLoS One. 2010 Dec 22;5(12):e14403. doi: 10.1371/journal.pone.0014403 (PMC3008723; doi:10.1371/journal.pone.0014403)
Supplement: Figure S13 — Multiple alignment of passenger subdomain D10. (0.01 MB PDF) [file pone.0014403.s013.pdf]

```
Eco26           KVVLVAEGELNLKSELEDKNEINVYELMPHEIWQNATITTEMSGWCR--ATWIDAPTS--
Yen2            KVKVVAKNDVRQENEVTLDHEVRVGTLEPIDIWQNAIFTRNYSLHNNDGSKRDSCPIVNN
Ymo1            --FVVKTEGTSGLSSQDQTKIIDVKRLYPDEIWNKVRVKSGHSIYEKGKSVKDCRTSAGF
Eta1            -HVELVAQGFDKLTNSEKSPGINVKNLPPNKIWETFTVRSKALIRSDNGNESTCREIAGP
                    :        ..      : *  * * .**:.  .

Eco26           ----KTMKSNFS-ISLASGKS-LLFPMSVKVKASSNG-GYGGFTYGYFTFSGLSEKNIVN
Yen2            LFYPNYARLNWR-MQLVLNKD-MLHPMQITKLESKTS-KHG----INMTHIDSSTSEIFD
Ymo1            WNDTHHVNLNWG-VGIDLGNNQLLEGMEVKLRIDKVSSSYISPKDIVTRNILKTFDEVST
Eta1            AGKEHWIDAIVDGGQVNFNGKTLISPMTITGLTDGEQNGHYR----NGQFPLTQRNIYSD
                    :          :  . . ::  * :.   .     :               .

Eco26           SSSWSGDDKKG-ECWKDYWGSYNTYMEVQYNQKNYIYRSDSARGWQGMDNNNGPYTDNMI
Yen2            SYDNKDDNRLINKCIKEKYGTYKTYMDIKYAGREYKYEAINDLYWEGEGDDRESDKSSGF
Ymo1            PSSNYIAETLHTDCYDPHNDSVELVLDVEYLGKKTMYKAG-SVYWEGLGRTKK-------
Eta1            LSVNFGTKQIARECWKGHDGSYFIGAKVNYNGENFEYWMVDPHNWTGKGVGTNKYYLDTV
                       .    .* .   .:     .::*  .:  *       * * .

Eco26           YVK
Yen2            KKV
Ymo1            ---
Eta1            TKE
```
