# Supplementary material for: The Bacterial Intimins and Invasins: A Large and Novel Family of Secreted Proteins
Source: PLoS One. 2010 Dec 22;5(12):e14403. doi: 10.1371/journal.pone.0014403 (PMC3008723; doi:10.1371/journal.pone.0014403)
Supplement: Figure S14 — Multiple alignment of passenger subdomain D12. (0.01 MB PDF) [file pone.0014403.s014.pdf]

```
Bav2          APSITKVSTDATTGRVTVSGSAEPDVVVTVMFPDGTEKTVPTNDDGTYRATSDGN-MVSG
Bpe1          APTIANVATDATSGRVTAAGMAEPGANVTVNFPDGTRKTVVAGGDGAYTATSDRD-MVSG
Bbr1          VPPTVEVATDSSSGRVTVSGKATPRAKVKVDFPGGTSKTVTADADGRYRATSDGD-VPGG
Bpa2          MTPTVRHATDAQTGRVTVMGRTEAGALVTVQFPDGSSKTVRAQNDGGYAATSDTD-MVSG
Bav1          PQATITSVIPSTLGELTVSGLTQANAEVYVQFPDGSSTTVNADASGNYTAVSTSKSMPSG
Ahy1          GAPTLTAADSNGDDKPEVTGKAEPESTVTITWPDGTSTTTADVDGNYTLEAPTV-QGSG
                .          .. . * : .   * : :*.*: .*. :  .* *   :      .*

Bav2          DILAHATDRAKNRSPDTRYAYADAVAPA
Bpe1          DIRVQATDKAGNQSPEATRAYADAVDRT
Bbr1          DIVVTQTGMPGAAGKPVRRPYVDTVAPT
Bpa2          PIVVSAGDADGNRTPAQQVMYTDTLDKT
Bav1          EIMVIATGRSAGVGSAATQTYTRNPPTA
Ahy1          TITATATDKSGNTGPATSVNYIDSTVPG
               *  .   .            *
```
