# Supplementary material for: The Bacterial Intimins and Invasins: A Large and Novel Family of Secreted Proteins
Source: PLoS One. 2010 Dec 22;5(12):e14403. doi: 10.1371/journal.pone.0014403 (PMC3008723; doi:10.1371/journal.pone.0014403)
Supplement: Figure S15 — Multiple alignment of passenger subdomain D13. (0.01 MB PDF) [file pone.0014403.s015.pdf]

```
Efe4                TVNDDNGSIIEWHYVRVRSKDEWKSLKPENVEYSTHSPGLSFKSLGGEERDGQWIEKVQL
Sen2                LPAGEEGKVIEWHYVRERSEEEWASLKPRNIKYQSDTPGLSFKALGGTERDGHWVERVLV
                       .::*.:******* **::** ****.*::*.:.:******:*** ****:*:*:* :

Efe4                VVKDPTARMTLAAMELNISATGPGGTHPVNGTIRMTPVMNL
Sen2                THVGDDAR----SFKLHIEASGPDDKHPVKGSVLLQAQSDS
                    .  .  **    :::*:*.*:**...***:*:: : .  :
```
