# Supplementary material for: The Bacterial Intimins and Invasins: A Large and Novel Family of Secreted Proteins
Source: PLoS One. 2010 Dec 22;5(12):e14403. doi: 10.1371/journal.pone.0014403 (PMC3008723; doi:10.1371/journal.pone.0014403)
Supplement: Figure S16 — Multiple alignment of passenger subdomain D14. (0.01 MB PDF) [file pone.0014403.s016.pdf]

```
Efe4            TEELNGSTNAPVVGSTLQAKTTCDTDKDCSSLFRYQWEISPDGNRWYDVPGATGQSWLMP
Sen2            NDEANGSVTAPVVGTEMRARTLCINNTDCTDAFNYQWEISDEMKSWKSVPGATKATWLLP
                .:* ***..*****: ::*:* * .:.**:. *.****** : : * .*****  :**:*

Efe4            AVMDGHSLQNKQVRVRVVS--ENVPTH-------
Sen2            YSLNGESLQNKYIRVRIISDKENAESNNATSAAN
                  ::*.***** :***::*  **. ::
```
