# Supplementary material for: The Bacterial Intimins and Invasins: A Large and Novel Family of Secreted Proteins
Source: PLoS One. 2010 Dec 22;5(12):e14403. doi: 10.1371/journal.pone.0014403 (PMC3008723; doi:10.1371/journal.pone.0014403)
Supplement: Table S1 — The sixty-nine proteins of the Intimin/Invasin (Int/Inv) family included in this study, listed according to phylogenetic cluster and position within that cluster. Cluster designations refer to the clustering patterns in the phylogenetic tree shown in Fig 1A. Protein sizes are presented in numbers of amino acyl residues (aas). Greek letters refer to the subcategory (order) of the proteobacteria. Other columns are self-explanatory. (0.12 MB DOC) [file pone.0014403.s017.doc]

# Supporting Information Table 1.

# Intimins and Invasins According to Phylogenetic Cluster

| **Cluster and**  **Protein**  **Abbreviation** | **Organismal**  **Source** | **Protein**  **Size (no.**  **of aas)** | **Organismal**  **Type** | **gi#** |
| --- | --- | --- | --- | --- |
| Cluster A |  |  |  |  |
| Yin2 | *Yersinia intermedia* ATCC 29909 | 753 | γ | 77977839 |
| Yin1 | *Yersinia intermedia* ATCC 29909 | 751 | γ | 77979954 |
| Yfr4 | *Yersinia frederiksenii* ATCC 33641 | 749 | γ | 77974432 |
| Ymo2 | *Yersinia mollaretii* ATCC 43969 | 686 | γ | 77963537 |
| Cluster B |  |  |  |  |
| Eta2 | *Erwinia tasmaniensis* Et1/99 | 480 | γ | 188533998 |
| Pan1 | *Pantoea ananatis* | 490 | γ | 188035728 |
| Esp2 | *Enterobacter* sp. 638 | 468 | γ | 146311965 |
| Eca1 | *Enterobacter cancerogenus* ATCC 35316 | 468 | γ | 209908385 |
| Cko1 | *Citrobacter koseri* ATCC BAA-895 | 454 | γ | 157145552 |
| Eco3 | *Escherichia coli* O157:H7 EDL933 | 417 | γ | 15801451 |
| Sen1 | *Salmonella enterica* subsp. *arizonae serovar* 62:z4,z23:-- | 460 | γ | 161503120 |
| Esa3 | *Enterobacter sakazakii* ATCC BAA-894 | 471 | γ | 156933694 |
| Kpn1 | *Klebsiella pneumoniae* subsp*. pneumoniae* MGH 78578 | 460 | γ | 152970765 |
| Spr1 | *Serratia proteamaculans* 568 | 497 | γ | 157371141 |
| Cluster C |  |  |  |  |
| Pal2 | *Providencia alcalifaciens* DSM 30120 | 2521 | γ | 212710278 |
| Cluster D |  |  |  |  |
| Efe4 | *Escherichia fergusonii* ATCC 35469 | 672 | γ | 218548924 |
| Sen2 | *Salmonella enterica* subsp. *arizonae serovar* 62:z4,z23:-- | 660 | γ | 161503245 |
| Cluster E |  |  |  |  |
| Pma3 | *Prochlorococcus marinus* str. MIT 9215 | 379 | Cyanobacteria | 157413600 |
| Pma1 | *Prochlorococcus marinus* str. MIT 9312 | 372 | Cyanobacteria | 78779562 |
| Pma5 | uncultured *Prochlorococcus marinus* clone ASNC3046 | 373 | Cyanobacteria | 91069978 |
| Pma4 | *Prochlorococcus marinus* subsp. *marinus* str. CCMP1375 | 410 | Cyanobacteria | 33240740 |
| Ssp1 | *Synechococcus* sp. RCC307 | 428 | Cyanobacteria | 148243547 |
| Ssp2 | *Synechococcus* sp. RCC307 | 436 | Cyanobacteria | 148241686 |
| Cluster F |  |  |  |  |
| Bpa2 | *Bordetella parapertussis* 12822 | 1937 | β | 33594824 |
| Bbr1 | *Bordetella bronchiseptica* RB50 | 969 | β | 33599102 |
| Bav2 | *Bordetella avium* 197N | 1654 | β | 187477403 |
| Bpe1 | *Bordetella pertussis* Tohama I | 1308 | β | 33592249 |
| Bav1 | *Bordetella avium* 197N | 747 | β | 187477361 |
| Cluster G |  |  |  |  |
| Psp2 | *Candidatus Pelagibacter* sp. HTCC7211 | 251 | α | 207082844 |
| Mba1 | *Methylophilales bacterium* HTCC2181 | 331 | β | 118594627 |
| Cluster H |  |  |  |  |
| Plu2 | *Pelodictyon luteolum* DSM 273 | 302 | *Chlorobi* | 78186442 |
| Cluster I |  |  |  |  |
| Cla1 | *Campylobacter lari* RM2100 | 1459 | ε | 222778515 |
| Cluster J |  |  |  |  |
| Rba1 | *Rhodobacterales bacterium* HTCC225*5* | 327 | α | 114770327 |
| Cluster K |  |  |  |  |
| Csu1 | *Chlamydia suis* | 1305 | *Chlamydiae* | 69204798 |
| Cluster L |  |  |  |  |
| Pas1 | *Photorhabdus asymbiotica* subsp. *asymbiotica* ATCC 43949 | 924 | γ | 211638304 |
| Cluster M |  |  |  |  |
| Plu1 | *Photorhabdus luminescens* subsp. *laumondii* TTO1 | 1695 | γ | 37525974 |
| Cluster N |  |  |  |  |
| Yen2 | *Yersinia enterocolitica* subsp. *enterocolitica* 8081 | 2484 | γ | 123441053 |
| Ymo1 | *Yersinia mollaretii* ATCC 43969 | 1424 | γ | 77962119 |
| Cluster O |  |  |  |  |
| Eco1 | *Escherichia coli* | 937 | γ | 215489850 |
| Cluster P |  |  |  |  |
| Sgl1 | *Sodalis glossinidius* str. ‘morsitans’ | 934 | γ | 85058580 |
| Eta1 | *Erwinia tasmaniensis* Et1/99 | 1400 | γ | 188535206 |
| Pal3 | *Providencia alcalifaciens* DSM 30120 | 542 | γ | 212711721 |
| Pru1 | *Providencia rustigianii* DSM 4541 | 2373 | γ | 212706386 |
| Eco26 | *Escherichia coli* 53638 | 1653 | γ | 188574072 |
| Pmi1 | *Proteus mirabilis* HI4320 | 2358 | γ | 197283938 |
| Cluster Q |  |  |  |  |
| Yfr2 | *Yersinia frederiksenii* ATCC 33641 | 906 | γ | 77974284 |
| Yps7 | *Yersinia pseudotuberculosis* | 1486 | γ | 218534673 |
| Yfr3 | *Yersinia frederiksenii* ATCC 33641 | 851 | γ | 77971991 |
| Yfr5 | *Yersinia frederiksenii* ATCC 33641 | 780 | γ | 77972649 |
| Yfr1 | *Yersinia frederiksenii* ATCC 33641 | 770 | γ | 77972786 |
| Yps4 | *Yersinia pseudotuberculosis* YPIII | 5337 | γ | 170022406 |
| Yps2 | *Yersinia pseudotuberculosis* | 985 | γ | 155441 |
| Cluster R |  |  |  |  |
| Ybe1 | *Yersinia bercovieri* ATCC 43970 | 945 | γ | 77959044 |
| Ype5 | *Yersinia pestis* KIM | 1050 | γ | 22126485 |
| Cluster S |  |  |  |  |
| Sen3 | *Salmonella enterica* subsp. *arizonae serovar* 62:z4,z23:-- | 1812 | γ | 161505715 |
| Esa2 | *Enterobacter sakazakii* ATCC BAA-894 | 1027 | γ | 156933176 |
| Eco14 | *Escherichia coli* E24377A | 1084 | γ | 157157710 |
| Cluster T |  |  |  |  |
| Ahy1 | *Aeromonas hydrophila subsp. hydrophila* ATCC 7966 | 916 | γ | 117619710 |
| Cluster U |  |  |  |  |
| Eco20 | *Escherichia coli* | 1418 | γ | 71979957 |
| Cluster V |  |  |  |  |
| Efe3 | *Escherichia fergusonii* ATCC 35469 | 2104 | γ | 218551120 |
| Efe2 | *Escherichia fergusonii* ATCC 35469 | 1089 | γ | 218549138 |
| Eco15 | *Escherichia coli* O157:H7 EDL933 | 1700 | γ | 15804908 |
| Eco25 | *Escherichia coli* O157:H7 | 2660 | γ | 20455388 |
| Eco6 | *Escherichia coli* IAI39 | 2836 | γ | 218702502 |
| Cluster W |  |  |  |  |
| Eal1 | *Escherichia albertii* TW07627 | 725 | γ | 170767304 |
| Eco10 | *Escherichia coli* | 734 | γ | 32186995 |
| Sty4 | *Salmonella typhi* | 626 | γ | 7644406 |
| Eco16 | *Escherichia coli* O127:H6 str. E2348/69 | 711 | γ | 215487854 |
| Cluster X |  |  |  |  |
| Efe5 | *Escherichia fergusonii* ATCC 35469 | 1185 | γ | 218549119 |

# The sixty-nine proteins of the Intimin/Invasin (Int/Inv) family included in this study, listed according to phylogenetic cluster and position within that cluster. Cluster designations refer to the clustering patterns in the phylogenetic tree shown in Fig 1A. Protein sizes are presented in numbers of amino acyl residues (aas). Greek letters refer to the subcategory (order) of the proteobacteria. Other columns are self-explanatory.
