# Supplementary material for: The Bacterial Intimins and Invasins: A Large and Novel Family of Secreted Proteins
Source: PLoS One. 2010 Dec 22;5(12):e14403. doi: 10.1371/journal.pone.0014403 (PMC3008723; doi:10.1371/journal.pone.0014403)
Supplement: Figure S11 — Multiple alignment of passenger subdomain D8. (0.02 MB PDF) [file pone.0014403.s011.pdf]

```
Eal1            DERIPTDSTYRFVLWRDKNKDGIFQQSEKLTDEEMAQYDYQWEFTGQS---AHGETGAQA
Eco10           KEPLPLNNTYRFVLWRDNNKDGVFQQVEKLTDEEMVQYDYKWEFTGKS---INGEVGAQA
Eco16           AKPIPLKSGYRFVMWRDSNNDGIYQQSEKLTEEEKARYDYEWEFTGKS---AHGSTGAKA
Sty4            DKHPTVNNTYRFLLWRDKNKDGVFQMSEQLTEEEMALYDYQWEFTGQS---TNGHTGALA
Yfr4            GVDLLTSGAHPQVGVVYTVK--LFDAANTDITATIPATEVHWALDGTN---TAG-CAITL
Yin1            GTDVLVSGNHPQVGVTYTVK--LYNAANVDITSTLPADSLHWALDGAN---TAG-CAITL
Yin2            GEDLLTTHNAPQVGVTYTVV--LFDENDVDVTETLPADEVHWALDGTN---TAG-CAITL
Ymo2            GTPLKVGETYRFIAWSDRQGNGKWSEGDEEVTSSLQS--IQWYLDGTNSSATGGSSGITL
                            :          :.  :            .* : * .     *  .

Eal1            NTTN-EDIVIPATNQEAAQKFSAQAGDGVQGYGLQVK---
Eco10           NTSN-EDIVIPATNREAAQTYGAQAGDGLQGYGLRVL---
Eco16           NTIN-EDLILPATNKEAASKFDAEERDGVQGYGIRVVYSE
Sty4            NTIN-EDLVLPVTNKEAAQKFAANVEDGVQGYGIRVTY--
Yfr4            NNHD-TGVTGYQFTPRTNGSSNSGVACGDQGFGLKVN---
Yin1            NNFD-TGATGYSFTPRPNGSSNSGVACGDQGFGLKVN---
Yin2            DNHD-TGMTGYTFTPRPNASSNSGVVCGDQGFGLKVN---
Ymo2            VDHEIAGATTDHYTVAVNHASSSGQTAGDQGFKLKVG---
                   :  .      .        :    * **: ::*
```
